# Divergent synthesis of benzazepines and bridged polycycloalkanones via dearomative rearrangement

**DOI:** 10.1038/s41467-022-31920-1

**Published:** 2022-07-29

**Authors:** Qiu Shi, Zhehui Liao, Zhili Liu, Jiajia Wen, Chenguang Li, Jiamin He, Jiazhen Deng, Shan Cen, Tongxiang Cao, Jinming Zhou, Shifa Zhu

**Affiliations:** 1 Key Laboratory of Functional Molecular Engineering of Guangdong Province, School of Chemistry and Chemical Engineering, South China University of Technology, Guangzhou, 510640 China; 2 Institute of Medicinal Biotechnology, Chinese Academy of Medical Science, Beijing, 100050 China; 3 Key Laboratory of the Ministry of Education for Advanced Catalysis Materials, Department of Chemistry, Zhejiang Normal University, Jinhua, 321004 China

**Keywords:** Diversity-oriented synthesis, Synthetic chemistry methodology

## Abstract

The dearomative functionalization of aromatic compounds represents a fascinating but challenging transformation, as it typically needs to overcome a great kinetic barrier. Here, a catalyst-free dearomative rearrangement of *o*-nitrophenyl alkyne is successfully established by leveraging the remote oxygen transposition and a weak N-O bond acceleration. This reaction features high atom-, step- and redox-economy, which provides a divergent entry to a series of biologically important benzazepines and bridged polycycloalkanones. The reaction is proposed to proceed through a tandem oxygen transfer cyclization/(3 + 2) cycloaddition/(homo-)hetero-Claisen rearrangement reaction. The resulting polycyclic system is richly decorated with transformable functionalities, such as carbonyl, imine and diene, which enables diversity-oriented synthesis of alkaloid-like polycyclic framework.

## Abstract (web summary)

The dearomative functionalization of aromatic compounds represents a challenging transformation, as it typically needs to overcome a great kinetic barrier. Here, the authors disclose a weak-bond-accelerated, catalyst-free dearomative [3,3]-rearrangement of o-nitrophenyl alkyne for the divergent synthesis of benzazepines and bridged polycycloalkanones via remote oxygen transposition.

## Introduction

**Fig. 1:**
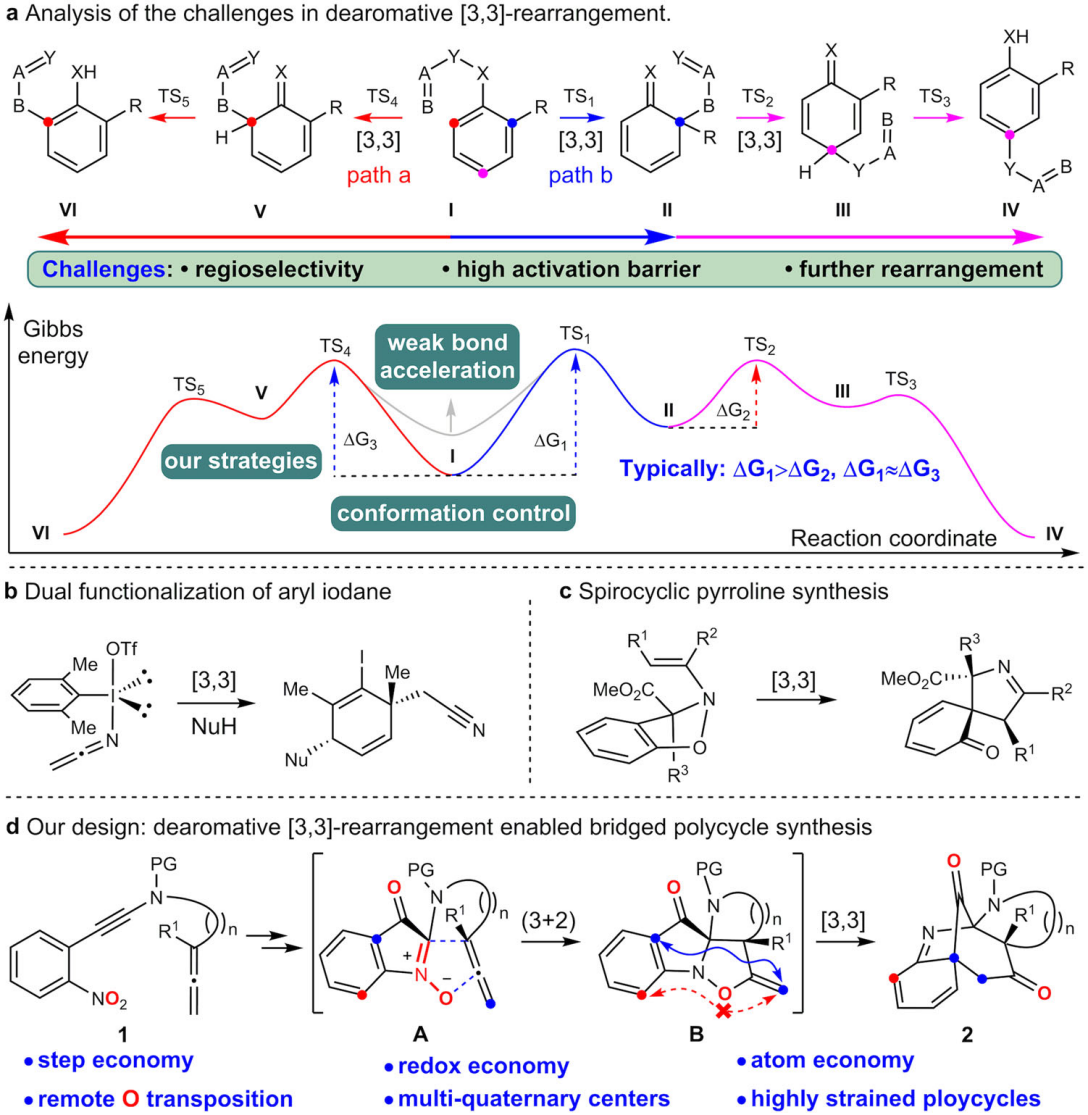
Dearomative [3,3]-rearrangement in efficient synthesis. **a** Analysis of the challenges in dearomative [3,3]-rearrangement. **b** Dual functionalization of aryl iodane. **c** Spirocyclic pyrroline synthesis. **d** Our design: dearomative [3,3]-rearrangement enabled bridged polycycle synthesis. PG = protective group.

The dearomative functionalization of aromatic compounds represents a fascinating transformation, which could rapidly build up stereocomplex 3-D alicyclic architectures with transformable C=C bonds from planar arenes in one step. Consequently, many successful approaches have been established during the last few decades^1–5^. From the perspective of arene substrates, most methods typically rely on low aromaticity arenes, such as phenol^6,7^, π-extended (hetero)arenes^8,9^, and hapto-coordinated arenes^10^. In terms of the thermodynamic profiles, highly active species such as cation^11^, carbene^12–15^, radical^16^, photoexcited intermediates^17,18^, and allyl organometallics;^19,20^ were usually applied in order to compensate the enthalpic penalty in breaking an aromatic system. In this vein, catalytic asymmetric dearomatization has become a powerful method in achieving optically pure molecules from arenes^2,21–24^. Additionally, redox manipulation of the arene could also lead the loss of aromaticity, but stoichiometric strong oxidants^7,25,26^ or reductants^27–29^ were required in many cases. Strangely, in spite of these achievements, there are only a few reports of dearomative arene functionalization by leveraging an aromatic [3,3]-sigmatropic rearrangement strategy^5,30–35^, which proceed through a dearomative intermediate. Several inherent challenges might result in the scarcity of such a dearomative [3,3]-rearrangement (Fig. 1a). Firstly, it is well-known that dearomatization processes have to overcome a prominent activation barrier (Fig. 1a, ΔG_1_ and ΔG_3_), which could not be sufficiently compensated from the enthalpy of sigma bond interchange during [3,3]-sigmatropic rearrangement. Secondly, the potential regioselectivity issues should be considered in developing such a reaction (Fig. 1a, paths a and b), so symmetrical or reactivity-differentiated substrates were usually applied^36–38^. Finally, the transiently formed dearomative intermediates are unstable and would easily collapse via rearomatization or consecutive [3,3]-sigmatropic rearrangement (Fig. 1a, product **IV** and **VI**)^39^. Recently, several excellent studies aiming to address the activity issues were achieved by developing new catalytic model^8,32^ or using energetic intermediates and substrates^37,38,40,41^. For example, Peng group disclosed a dearomative dual functionalization of aryl iodanes by integration a iodonio-Claisen rearrangement with a subsequent nucleophilic interception of the dearomative intermediate (Fig. 1b)^38^. Anderson and coworkers reported a tandem of (3 + 2) cycloaddition and hetero-Claisen rearrangement for the dearomative synthesis of spirocyclic 1-pyrrolines by exploiting the activity of nitrones and arynes (Fig. 1c)^40^. However, to the best of our knowledge, access to a bridged polycyclic system via dearomative [3,3]-sigmatropic rearrangement is still a yet unmet challenge. Inspired by these intriguing achievements and our successful experiences in dearomative Claisen rearrangement^42–44^, we intend to use the weak N-O bond (N-O: 53 kcal/mol vs C-O: 86 kcal/mol) as leverage to design the dearomative [3,3]-sigmatropic rearrangement, aiming for the synthesis of bridged alicyclic systems^45^.

In the past few decades, the (3 + 2) cycloadditions of nitrone have become a valid approach to assemble the N-O bond containing rings^45^, especially integrated with the alkyne chemistry^46–51^. For example, the nitroalkyne-based oxygen transfer could efficiently generate the nitrone, which can then be intercepted by an intermolecular alkene to give a polycyclic system^48–50^. Similarly, we surmised that an in-situ generated nitrone bearing a well-situated allene would undergo selective cycloaddition and the conformation of the newly formed ring would ensure the consequent dearomative sigmatropic rearrangement occurring selectively to give a stereochemically and functionally rich bridged polycyclic system (Fig. 1d).

In this work, we disclose a catalyst-free dearomative [3,3]-rearrangement of *o*-nitrophenyl alkyne for the divergent synthesis of benzazepines and bridged polycycloalkanones via remote oxygen transposition (over 11 atoms). This reaction features high atom-, step- and redox-economy. In addition, the resulting polycyclic system is constituted by four rings, two bridge-head quaternary centers, and transformable functionalities of cyclohexadiene and cyclodiketone, which enables the diversity-oriented synthesis of alkaloid-like fused polycyclic frameworks.

## Results and discussion

### Serendipitous discovery and rationalization

**Fig. 2:**
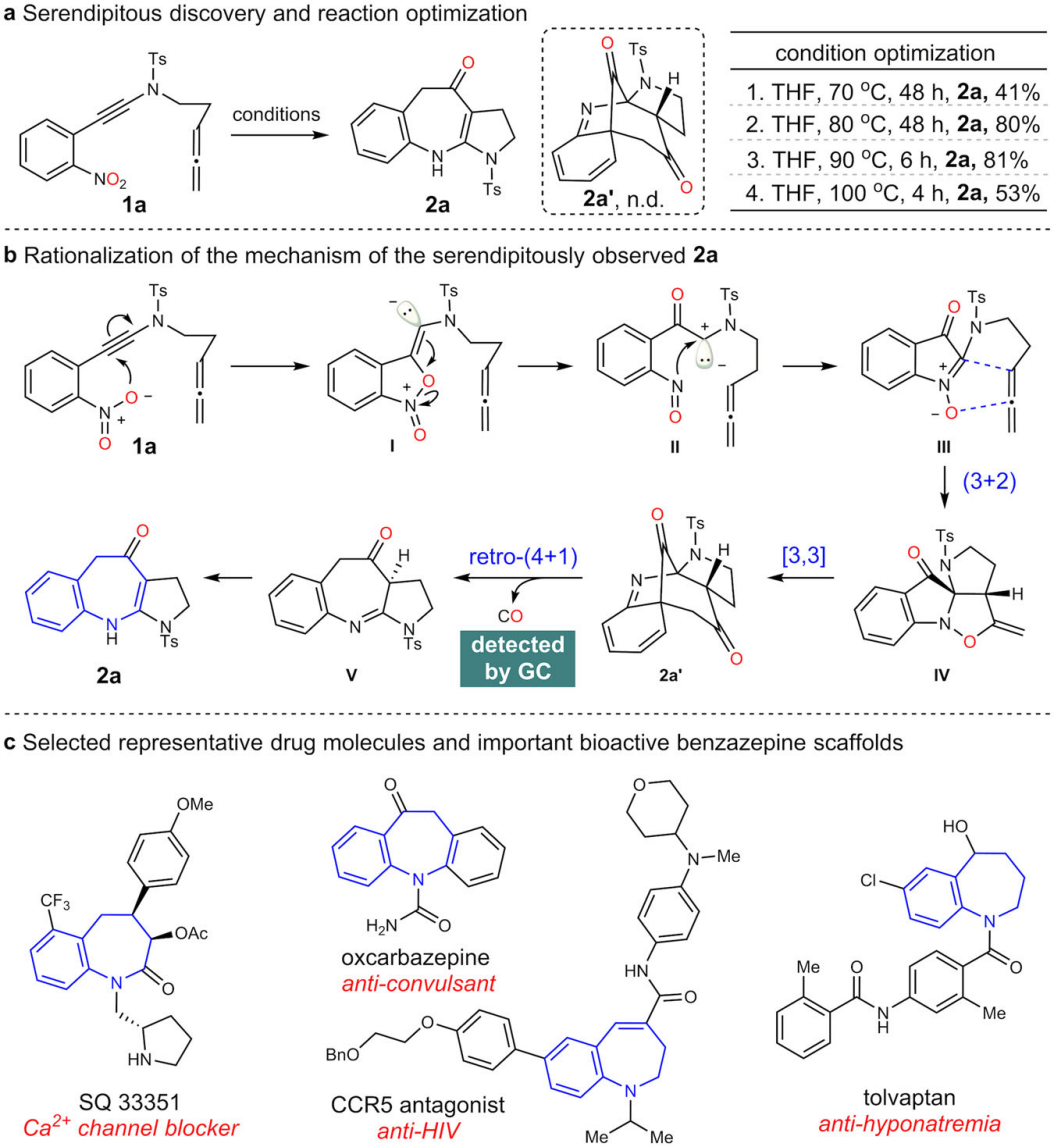
Reaction optimization and mechanistic rationalization. **a** Serendipitous discovery and reaction optimization. **b** Rationalization of the mechanism of the serendipitously observed **2a**. **c** Selected representative drug molecules and important bioactive benzazepine scaffolds. Ts = *p*-toluenesulfonyl. Bn = benzyl.

**Fig. 3:**
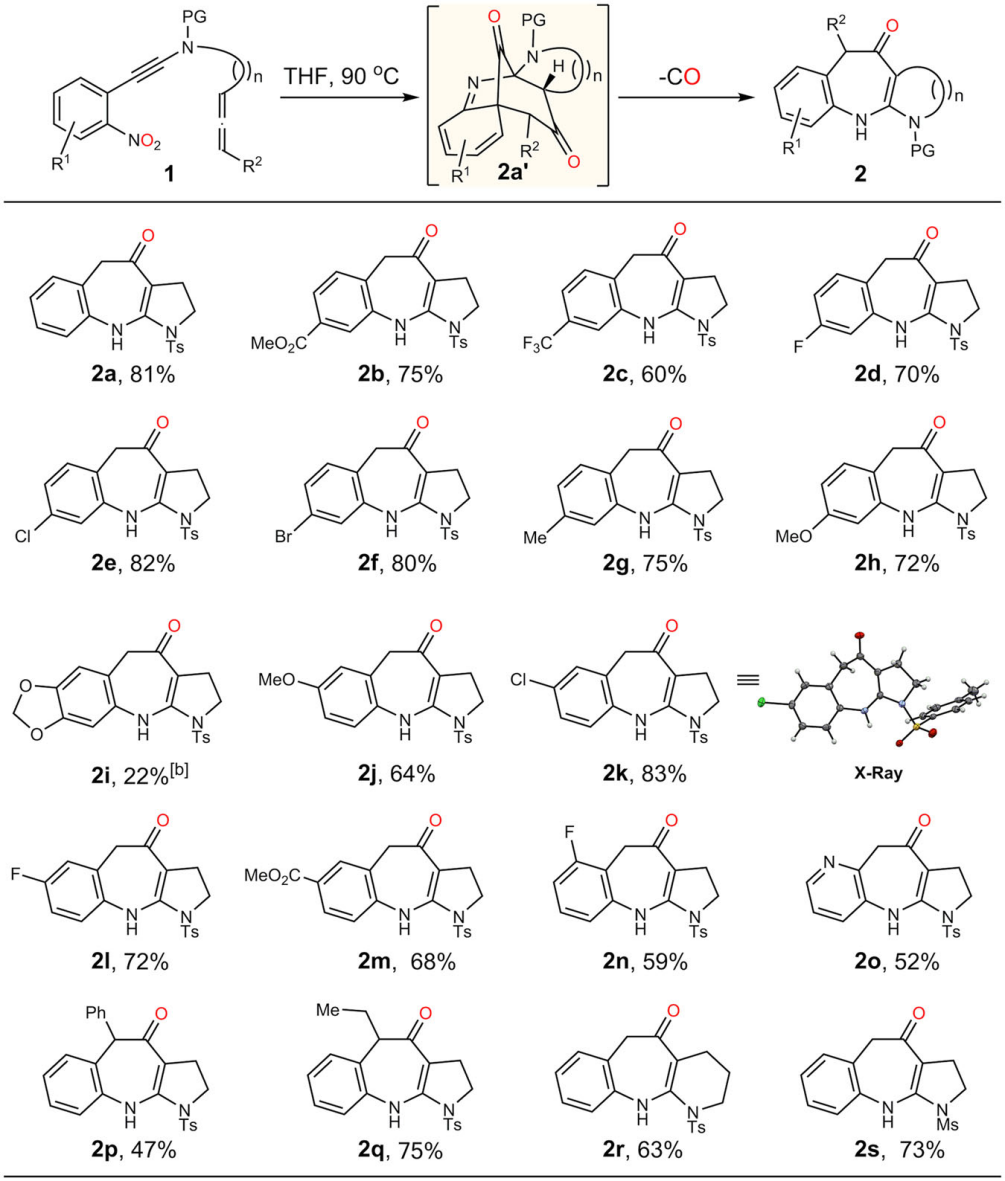
Evaluation of scope of benzazepines^a^. ^a^Reaction conditions: The solution of **1** in THF (0.03 M) was stirred under N_2_ atmosphere at 90 ^o^C for 6 h. ^b^Heating the resulting filtrate of **1** after the ynamide formation. PG = protective group. Ts = *p*-toluenesulfonyl. Ms = methanesulfonyl.

To verify our hypothesis, we set out the investigation with the easily available ynamide **1a** as model substrate. Surprisingly, under thermolysis conditions, the desired bridged cycloalkanone was not detected, but an unknown compound with one carbon atom less (identified by NMR) was isolated in 81% yield (Fig. 2a, entry 3). The temperature has great impact on the reaction yields (Fig. 2a). The structure was tentatively assigned to benzazepine **2a** by analysing of the NMR spectra, which was further confirmed later by the X-ray crystal diffraction analysis of the analogue **2k** (Fig. 3). The formation of benzazepine **2a** can be rationalized as shown in Fig. 2b. The thermal-triggered intramolecular oxygen transfer^48–50^ of **1a** initially gave the nitrone **III** (Fig. 2b), which could be effectively trapped by the tethered allene with complete diastereoselectivity. The resulting (3 + 2) cycloaddition adduct **IV** then underwent the dearomative [3,3]-sigmatropic rearrangement to afford the bridged cycloalkanone **2a’**, one molecule of carbon monoxide (CO) was further extruded through a cheletropic decarbonylation reaction (retro (4 + 1) reaction for simplicity)^52–56^ to regenerate the benzene ring and followed by isomerization to give the thermodynamically favourable product **2a**. It is noteworthy that the CO gas could be detected by GC analysis of the gas in the reaction flask (see SI for details). The extrusion of CO was presumably due to the high tendency of rearomatization and high ring strain of the intermediate **2a’**.

### Scope of the benzazepines

Encouraged by our initial discovery and the important bioactivity of benzazepines (Fig. 2c)^57–60^, we then started to test the generality of this reaction under the optimized conditions (Fig. 2a, entry 3). As shown in Fig. 3, the electronic nature of the arene has little influence on the formation of the benzazepines (**2a-n**) except the methylenedioxy-substituted product **2i**. Both electron-rich and electron-deficient benzazepines were isolated in similar yields (about 70%). It is worth noting that the sterically hindered product **2n** could obtained in 59% yield as well. Interestingly, the otherwise difficult to access pyridine-fused azepine **2o** was also achieved in moderate yield (52%). In addition, the internal allene tethered substrates were subjected to the standard reaction conditions as well, giving the desired benzazepines **2p** and **2q** in 47% and 75% yield, respectively. Finally, the piperidine-fused benzazepine **2r** and methylsulfonyl-protected benzazepine **2s** could also be obtained in satisfactory yields.

**Fig. 4:**
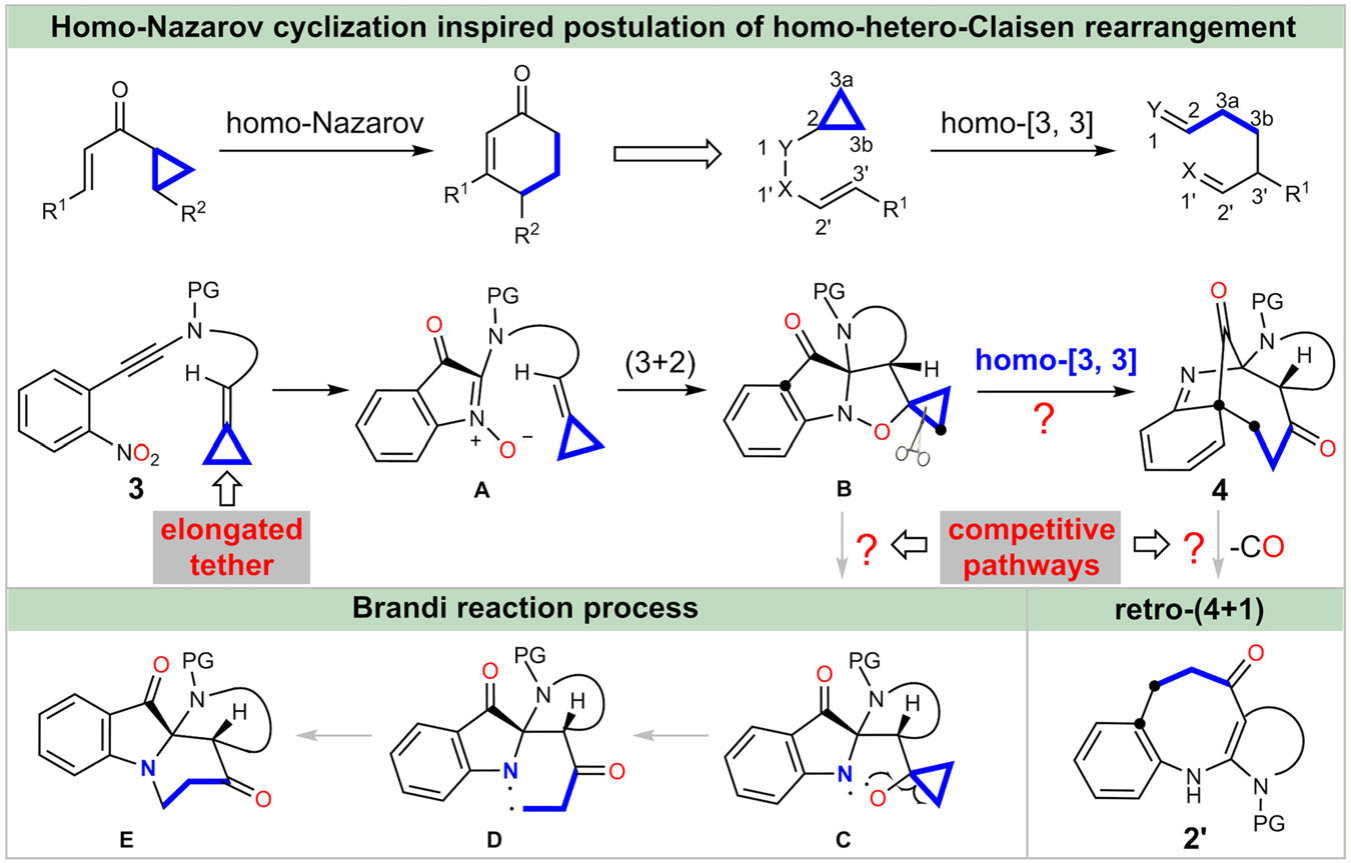
Minimization of ring strain in achieving bridged polyclcloalkanone. The homo-hetero-Claisen rearrangement postulation inspired by mechanistic insight of Claisen rearrangement and the homo-Nazarov cyclization. PG = protective group.

Having realized the formation of benzazepines, we wondered if the originally desired bridged polycyclic system could be accessed through the stabilization of the transient dearomatized intermediate via minimization of ring strain (Fig. 4). As the ring strain will drastically reduce with the increasing of ring size, therefore, we tried to extend the [3.2.1]-bridged system to a [n.2.1]-bridged one (n > 3). Inspired by the homo-Nazarov cyclization reaction^61–63^ and also the mechanistic fact that the [3,3]-sigmatropic rearrangement might involve a diradical species^39^, replacing one C=C bond of the allene with a cyclopropane moiety^64^ may enable a homo-Claisen rearrangement and lead to the desired [4.2.1]-bridged polycyclic product **4** without CO extrusion. It is worthy to note that a radical mediated Brandi rearrangement^65–67^, in addition to the competitive retro (4 + 1) addition reaction, might be involved as well, which further complicated the reaction system.

### Scope of the bridged polyclcloalkanones

**Fig. 5:**
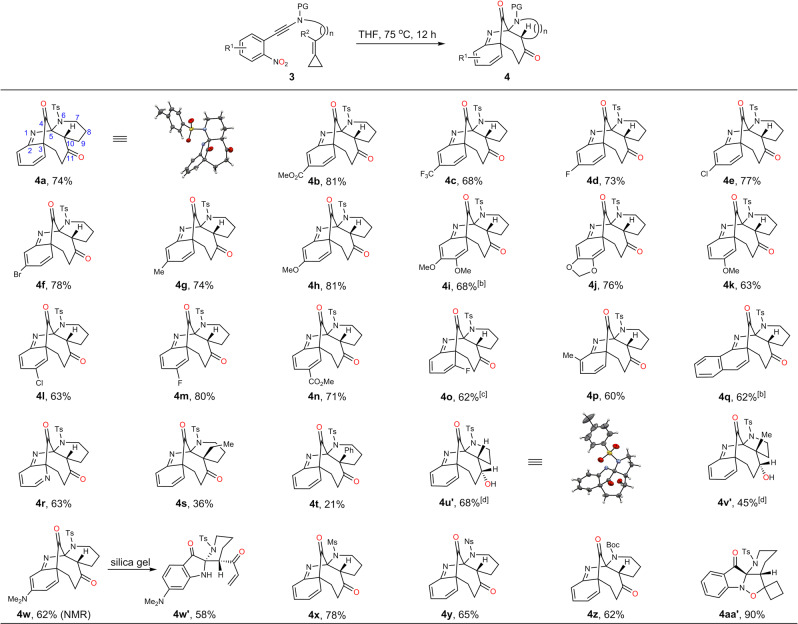
Evaluation of scope of benzazepines^a^. ^a^Reaction conditions: The solution of **3** in THF (0.03 M) was stirred under N_2_ atmosphere at 75 ^o^C for 12 h; ^b^Heating the resulting filtrate of **3** after the ynamide formation; ^c^80 °C for 36 h; ^d^NaBH_4_ (3 eq) and 0.5 mL MeOH were added when the rearrangement reaction mixture cooling to room temperature, then stirred for another 1 h. Note: these imine-containing bridged skeletons are quite stable, without obvious decomposition when placed under ambient atmosphere and temperature for more than 2 months. PG = protective group. Ts = *p*-toluenesulfonyl. Ms = methanesulfonyl. Ns = 2-nitrobenzenesulfonyl. Boc = t-butoxycarbonyl. THF = tetrahydrofuran.

With these considerations in mind, we then set out to validate the feasibility of such hypothesis. Fortunately, when nitroalkyne **3a** with a tailored methylenecyclopropane was applied as substrate, the envisioned dearomative bridged polycyclic product **4a** was obtained in 74% yield, which was unambiguously confirmed by X-ray crystal diffraction analysis (Fig. 5). To the best of our knowledge, it is the first homo-hetero-Claisen rearrangement reaction. It is also noteworthy that in this cascade process the two oxygen atoms of the nitro group were transferred over 4 and 11 atoms, giving two transformable ketone functionalities in a redox-neutral manner. In line with our previous observation, the electronic properties and substituted patterns of the arene have little effect on the reaction performance (**4a**-**p**, 60-81%). For example, the nitrobenzenes with -CO_2_Me or -OMe group at the meta-position could deliver the desired products **4b** and **4h** in excellent yields. In addition, the methylenedioxy substituted product **4j** could be obtained in 76% yield, which is in stark contrast to that of **2i** (22%). Notably the formation of **4o** needed a slightly elevated temperature and prolonged reaction time (80 ^o^C, 36 h), which might be attributed to the steric hindrance. The practicability of the naphthalene and pyridine-based substrates were also tested to give the corresponding products **4q** and **4r** in satisfactory yields. The reaction was highly sensitive to the size of R^2^ as the yields decreased sharply when the group R^2^ was replaced by ethyl and phenyl, furnishing the corresponding products **4s** and **4t** in only 36% and 21% yield, respectively. When ynamide with a shorter tether were used as substrates, the desired products decomposed quickly upon chromatography isolation on silica gel. Fortunately, these in situ generated polycyclic diketones could be selectively reduced to the corresponding alcohols **4u’** and **4v’** in 68% and 45% yield, respectively. It is worthy to mention that the dimethyl amine substituted product **4v** could be observed by NMR in 62% yield, but it was sensitive to silica gel and transformed to the ring fragmentation product **4w’** in 58% yield. In addition, methylsulfonyl- and *o*-nitrobenzenesufonyl protected polycyclic product **4x** and **4y** could also be obtained in good yield. In sharp contrast to the sulfonyl protected counterparts, the carbamate protected substrate **3z** required more forcing conditions (100 ^o^C for 120 h), delivering the desired bridged adduct **4z** in 62% yield with the thermolabile tert-butoxycarbamate moiety survived. Interestingly, when the cyclobutene-containing substrate was tested, however, only the (3 + 2) adduct **4aa’** was isolated in excellent yield, which couldn’t be further transferred to the desired bridged cyclic product even elevating the reaction temperature to 200 ^o^C, which indicated that the weak N-O bond is thermally stable and the cyclopropane is critical for the following rearrangement.

### The compatibility of other type substrates

**Fig. 6:**
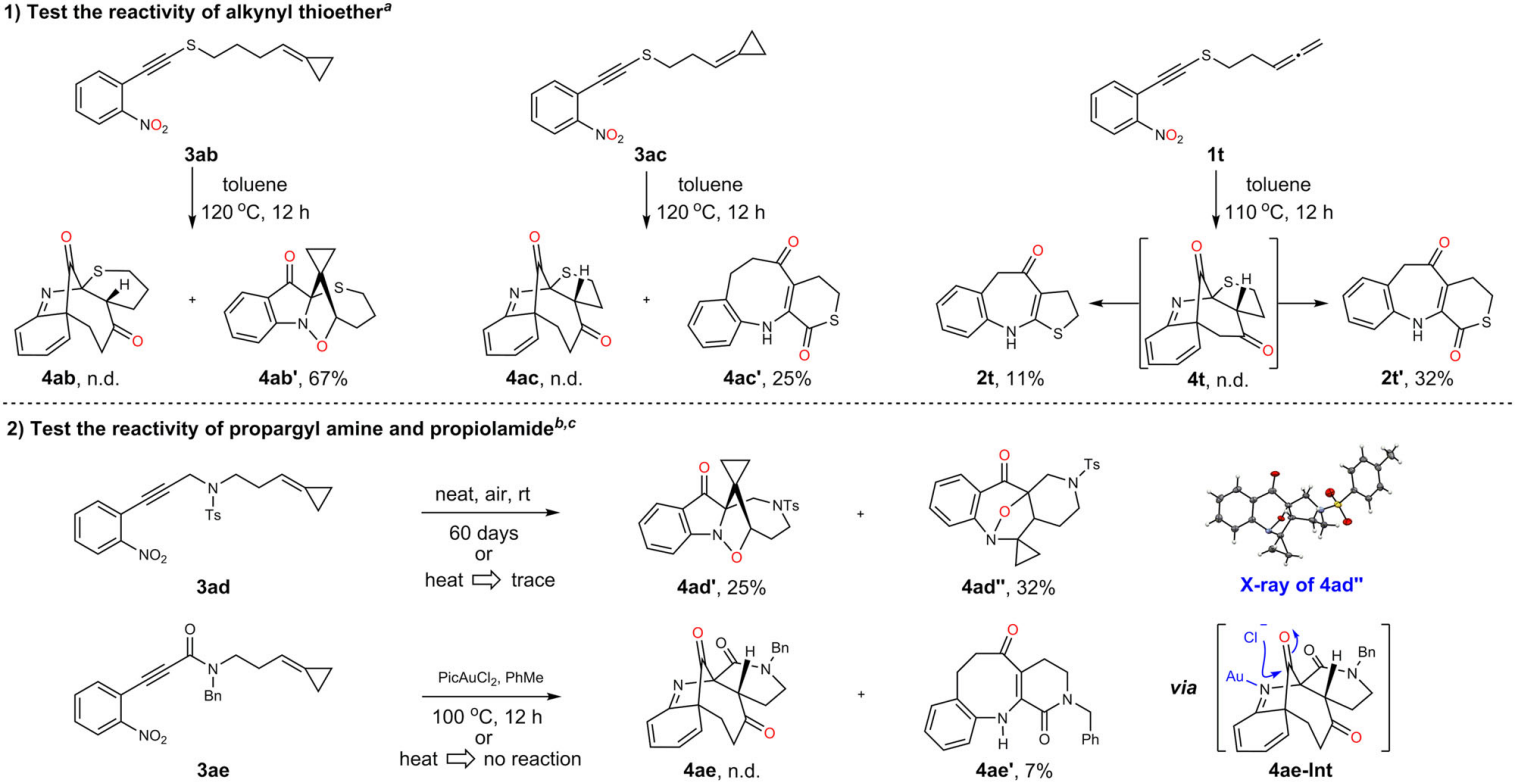
Test the compatibility of other type substrates. ^a^The solution of **3** in toluene (0.03 M) was stirred under N_2_ atmosphere at 110 or 120 ^o^C for 12 h; ^b^The viscous oil **3ad** (50 mg) in a 25 mL round bottom flask with a stopper was stood at room temperature for 60 days; ^c^To the solution of **3ae** in PhMe (0.03 M) was added PicAuCl_2_ (0.2 eq) under nitrogen atmosphere, then stirred at 100 °C for 12 h. n.d. means not detected. Pic = 2-pyridinecarboxylate. Ts = *p*-toluenesulfonyl. Bn = benzyl.

To further explore the reaction scope, other type of nitroalkynes were tested as substrates as well (Fig. 6). Unexpectedly, a low conversion was achieved when the alkynyl thioethers were tested as the substrates under the standard conditions, which was in striking contrast to Verniest’s results^48^. When the reaction temperature was increased from 75 ^o^C to 120 ^o^C, the thioether **3ab** exclusively afforded the *endo*-cycloaddition adduct **4ab’** rather than the desired bridged dione **4ab**. Such selectivity inversion indicated that the steric repulsion between the Ts group and cyclopropane moiety is critical in ensuring the *exo*-cycloaddition and the subsequent rearrangement processes. We further envisioned that a shorter tether might alter the reaction selectivity in favoring *exo*-cycloaddition as the ring tension would increase dramatically for the *endo*-selectivity. Surprisingly, the thioether **3ac** with one less carbon atom delivered a bridged-carbonyl group transposed product **4ac’** without observing **4ab’**-like *endo*-adduct. In addition, the allene thioether **1t** was also examined, which gave the benzazepines **2t** and **2t’**, which might arise from an asynchronous heterolysis^54^ of transient bridged product **4t**. In addition to the substrates with heteroatom-modified and electronically biased carbon-carbon triple bond (ynamide and thioalkyne), the nitroalkynes **3ad** and **3ae** with carbon atom-capped carbon-carbon triple bond were explored as well. Interestingly, the substrate **3ad** could transfer to **4ad’** and **4ad”** upon exposure to air at room temperature over a period about two months. The structure of **4ad”** was unambiguous validated by X-ray diffraction analysis. However, under thermal conditions, the substrate decomposed dramatically (>150 °C) with only trace amount of the desired products were observed by NMR analysis of the reaction mixture. The formation of the *endo*-adduct **4ad’** confirmed again the steric effect is very important, which is in line with the observation in **3ab**. For the propiolamide **3a**, the reaction is sluggish under heating conditions (150 ^o^C for 8 h). A cooperative strategy by combined the gold catalysis^49,50^ and the thermolysis process was also applied. However, we could not detect the desired product **4ae**, but with its descendant **4ae’** being isolated in 7% yield, which might proceed through the intermediate **4ae-Int** with the assistance of gold coordination and nucleophilic chloride.

### Mechanistic study

**Fig. 7:**
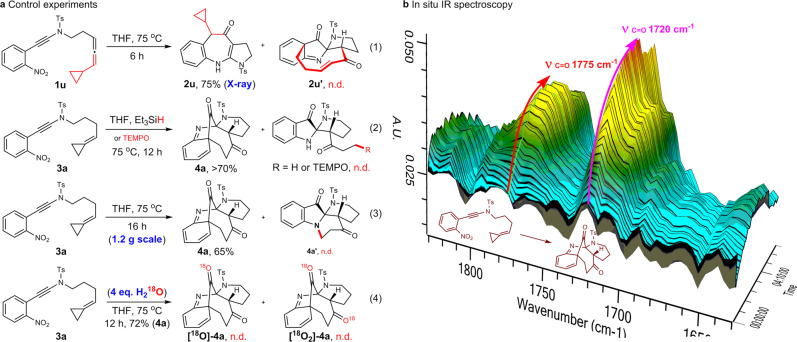
Mechanistic investigation and gram-scale synthesis. **a** Control experiments: The Eqs. 1 and 2 indicate that no radical is involved in the (homo-)hetero-Claisen rearrangement. Eq. 3 is robust and radical mediated Brandi cyclization occurred, which imply the absence of radical species in the homo-hetero-Claisen rearrangement. **b** Investigation of the cascade reaction profiles by the in situ IR spectroscopy. THF = tetrahydrofuran. Ts = *p*-toluenesulfonyl.

To better understand the reaction mechanism, several control reactions were then conducted. Intuitively, we think that a loose radical pair involved pathway^39^ might be more feasible as tremendous transannular strain will built-up in achieving a synchronic [3,3]-sigmatropic rearrangement transition state. Therefore, the cyclopropane substituted substrate **1u** was applied to probe the intermediacy of such radical pair. However, no ring opening products, such as **2u’**, were detected, but with the normal benzazepine **2u** being isolated in good yield (Fig. 7, Eq. 1). In addition, the attempt to intercept or terminate the envisioned radical species with TEMPO and Et_3_SiH also failed (Eq. 2). Finally, **3a** was conducted on a gram scale in order to detect the potential Brandi cyclization product **4a’**, which typically proceeded through a radical pathway^65–67^. Similarly, the normal rearrangement product **4a** was obtained in 65% yield, while no desired Brandi cyclization product **4a’** was isolated even in trace amount (Eq. 3). These results indicated that the postulated radical pair either reluctant to be perturbated from outside or not existed. Introducing 4 equivalents of H_2_^18^O to the reaction system has no effect on the reaction outcome (72%), which further confirmed its robustness. The HRMS and ^13^C-NMR of resulting product revealed that no ^18^O was incorporated (Eq. 4), which corroborated that the carbonyl groups came from the transposition of oxygen of nitroxide rather than external water. In addition, the reaction profiles were monitored by in situ IR spectroscopy, which characterized by the stretching vibration absorption of two carbonyl groups at 1720 cm^−1^ and 1775 cm^−1^, while no obvious absorption of intermediates were accumulated throughout, indicating their metastable character. Taken together, we are preferred more to a concerted but asynchronous [3,3]-sigmatropic rearrangement process wherein the N-O cleavage more advanced to relieve the incoming transannular strain.

**Fig. 8:**
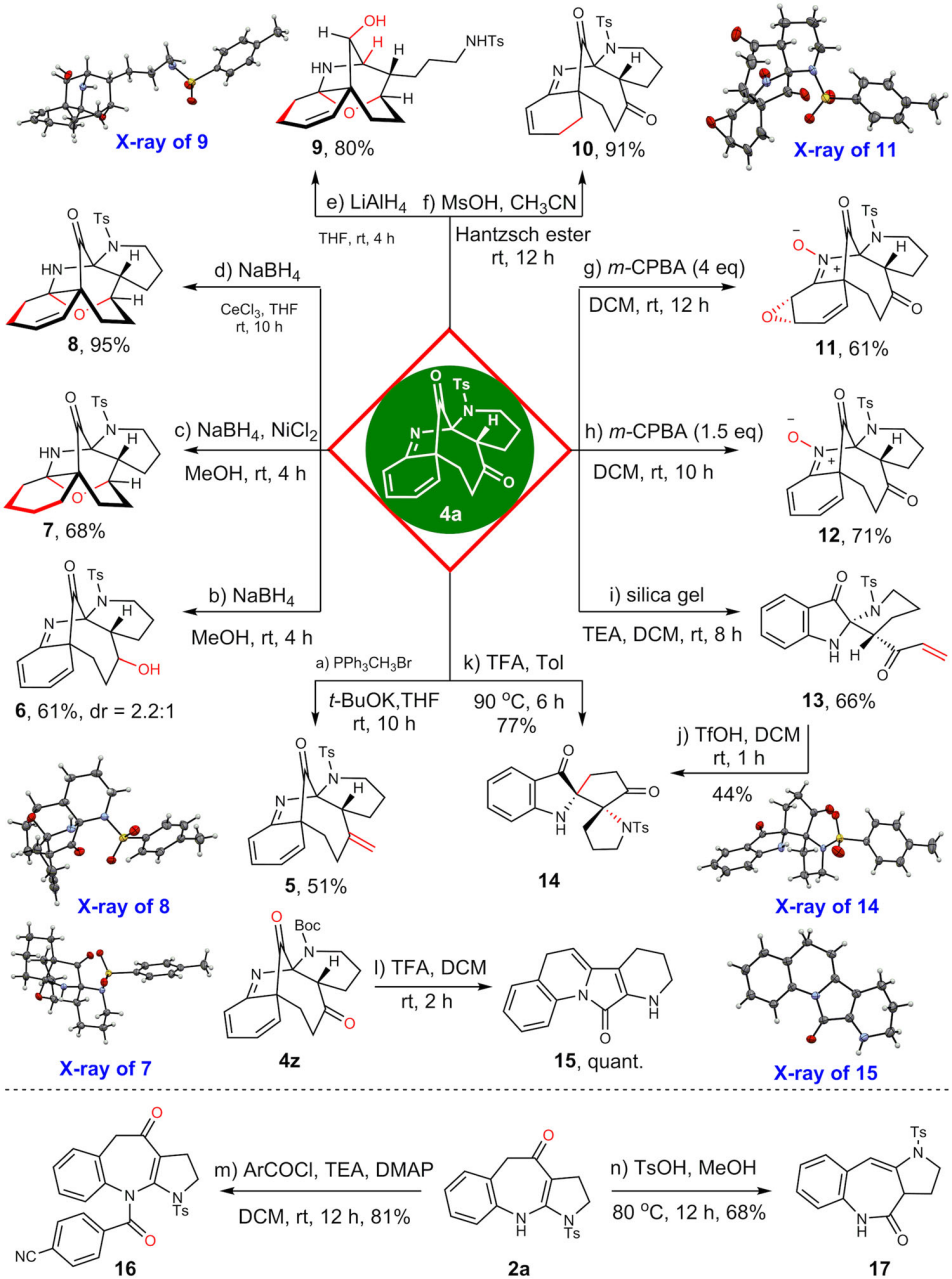
Late-stage diversification of 4a and 2a. Selective olefination and reduction of the dione to give **5** and **6**. Reductive acetalation companied with entirely reduction of the diene to afford the cage product **7**. Luche reduction conditions could also produce the cage product **8** with the cyclohexadiene partially hydrogenated. The bridged carbonyl group could be reduced with lithium aluminium hydride to furnish **9**. One C = C double bond of the cyclohexadiene was selectively reduced by Hantzsch ester to provide **10**. Epoxidation product **11** was obtained with *m*-CPBA. Kinetically oxidation of the imine moiety to give nitrone **12**. Retro Michael addition to give the fragmentation oxindole **13**, which could transform to the spiral dione **14**. Under the acidic deprotection conditions the three-dimensional architecture of **4z** collapsed entirely to form a unique [6-6-5-6] tetracyclic architecture **15**. Furthermore, the benzoyl functionality could be easily installed on free nitrogen atom of **2a** to give product **16**. Interestingly, compound **2a** has been structurally reorganized into a [6-7-5] tricyclic architecture **17** by refluxing in TsOH/methanol solution. *m*-CPBA = *m*-chloroperoxybenzoic acid. TEA = triethylamine. TFA = trifluoroacetic Acid. DMAP = 4-(dimethylamino)pyridine. Ms = methylsulfonyl. Ts = *p*-toluenesulfonyl. Boc = *t*-butoxycarbonyl. NaBH_4_ = sodium borohydride. LiAlH_4_ = lithium aluminum hydride.

With the bridged polycyclic compounds in hand, efforts to demonstrate the synthetic utility of them were then conducted (Fig. 8). The stereochemically and functionally rich polycyclic systems could be successfully converted into a series of alkaloid-like frameworks (**5-14**) by using very simple and practical procedures (6-7 steps in total from the commercially available starting material). Taking **4a** as an example, the two carbonyl groups could be selectively differentiated under the Wittig reaction conditions to give the olefin **5** in 51% yield. Interestingly, the reduction of **4a** with sodium borohydride gave alcohol **6** in 2.2:1 dr, while under the otherwise identical conditions except nickel chloride, a cage product **7** was obtained in high yield, which might arise from the selective ketone reduction, C=C bond hydration and acetalation processes. In addition, under the Luche reduction conditions, a similar cage product **8** with one C=C reserved was furnished in excellent yield. Under more forcing conditions, another cage compound **9** with the bridged carbonyl group being reduced could be isolated in 80% yield. These complex architectures were confirmed by the X-ray analysis (**7**, **8** and **9**). In addition, selective reduction of one C=C bond of the cyclohexadiene could also be achieved with the Hantzsch ester as hydride donor, giving the product **10** in 91% yield. Epoxidation of the less sterically hindered C=C bond was feasible, but accompanied by the oxidation of imine motif. The stereochemistry of the epoxide **11** was also established by X-ray diffraction analysis. Reducing the loading of *m-*CPBA would selectively produce the kinetic product **12** in good yield. Under basic conditions in the presence of silica gel, a ring fragmentation product **13** was obtained in moderate yield. Exposing **13** to the acid condition led to a bis-spiral system **14**, which was also confirmed by X-ray diffraction analysis. Interestingly, the spiral indolone **14** could also be obtained in 77% yield by treating **4a** with trifluoroacetic acid at 90 ^o^C for 6 h. The Boc-protected product **4z** was labile to acidic condition, with the bridged architecture collapse to a planar molecule **15** at room temperature in quantitative yield. Finally, further transformation of benzazepine **2a** was conducted to demonstrate its versatile capacity in diversification modification the benzazepine skeleton. As many of the vaptan class of drugs contain a N-linked benzoyl moiety^57,68,69^, the benzoylation product **16** was synthesized preferentially. Interestingly, the peripheral pyrrole unit could shift to another position under refluxing acidic environment to give **17** in good yield.

### The antiviral activity

**Fig. 9:**
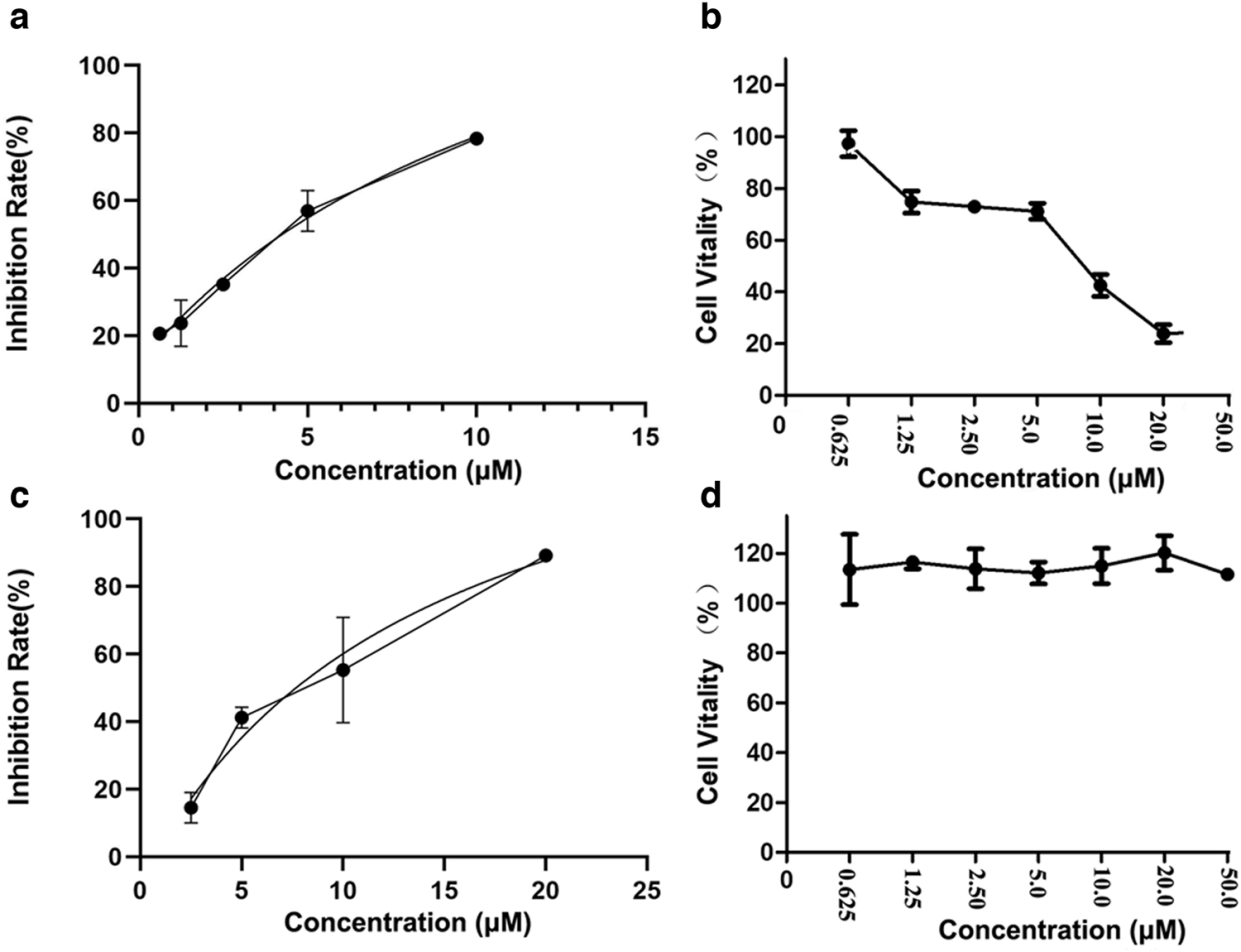
The antiviral activity. **a 13** inhibited the growth of HIV-1 virus in dose dependent manner in SupT1 cell; **b 13** exhibited moderate cytotoxicity with CC_50_ of 8.22 µM for SupT1 cell; **c 4a** inhibited the growth of IAV virus in dose dependent manner in 293T-GLUC cell; **d 4a** exhibited low cytotoxicity for 293T-GLUC cell. (*n* = 3). Bar = mean. Error bars = ±SEM. Data are from at least three independent experiments and evaluated via Prism (version 5.01, GraphPad Software, San Diego, CA, USA).

The approved anti-HIV drug, nevirapine, is a benzazepine analogue. Therefore, we firstly evaluated the chemotype **2** compounds (with benzazepine scaffold) using as nevirapine the positive control. As results, the chemotype **2** compounds only showed modest anti-HIV activity in SupT1 cells at 10 µM concentration (Table S3). To explore whether these unique architectures of **4** and its derivatives could serve as potential bioactive agents with novel core structure, we performed chemical structure similarity search in therapeutic target database (TTD) using the core structure of this series compound as the template, and four compounds were obtained with the Tanimoto similarity more than 0.7. The most similar compound Spiro[pyrrolidine-2,2-adamantane] (Tanimoto similarity was 0.745) was identified with the antiviral activity against influenza A virus (IAV) and the human immunodeficiency virus type 1 (HIV-1) and type 2 (HIV-2). Therefore, we explored the bioactivity of these compounds via testing the antiviral activity of IAV and HIV-1. As results, preliminary anti-HIV-1 assay indicated that compound **13** displayed the best anti-HIV effect with 97% reduction of VSV-G-pseudotyped HIV-1 virus in SupT1 cells at 10 µM concentration (Table S4). Further testing showed that **13** inhibited HIV-1 in a dose dependent manner (EC_50_ = 4.24 µM, Fig. 9a). However, the antiviral potency was measured in a Luc reporter assay which could not separate inhibition from the cytotoxicity. Therefore, the observed Luc reduction shown in Fig. 9a may largely reflect the cytotoxicity of **13** in SupT1 cell lines (CC_50_ = 8.22 µM, Fig. 9b). As for the antiviral activity against IAV, most of the compounds under preliminary test exhibited anti-IAV activity in 293T-GLUC cell at 10 µM concentration (Table S5), and **4a** displayed the best anti-IAV effect with 92% reduction of IAV virus. Further activity inspection indicated **4a** could inhibit the growth of IAV virus in dose dependent manner with the EC_50_ of 7.73 µM (Fig. 9c). Importantly, **4a** exhibited low cytotoxicity (not showing cytotoxicity up to 50 µM) for 293T-GLUC cell (Fig. 9d), suggesting that the observed antiviral effect was not due to cytotoxicity. Taken together, this series compound would become a type of promising anti-virus agents with brand new scaffold.

In summary, we disclosed a dearomative rearrangement, which enable divergent accesses to a series of unique benzazepines and bridged polycycloalkanones in a redox-neutral, step- and atom-economic manner. The reaction was proposed to proceed through a tandem oxygen transfer cyclization/(3 + 2) cycloaddition/(homo-)hetero-Claisen rearrangement reaction, wherein an oxygen atom was eventually transposed over 11 atoms. The resulting bridged polycycloalkanones could be successfully converted into several alkaloid-like frameworks via late-stage diversification. The preliminary bioactivity investigation indicated that these unique bridged frameworks hold great potential to be a type of promising anti-virus agents with brand new scaffold.

## Methods

### General procedure for the synthesis of benzazepines and bridged polycycloalkanones

A dried 25 mL Schlenk tube was flushed with nitrogen three times. The solution of **1** (or **3**) in the THF (0.03 M) was added to the tube under nitrogen atmosphere. The resulting mixture was put in a 90 °C (or 75 °C for **3**) oil bath and stirred for 6 h (or 12 h for **3**). After cooled to room temperature, the mixture was transferred to a 25 mL round bottom flask and evaporated under reduced pressure. The residue was purified by column chromatography on silica gel (petroleum ether/EtOAc as eluent) to give desired cyclization product **2** (or **4**).

### Reporting summary

Further information on research design is available in the Nature Research Reporting Summary linked to this article.

## Supplementary information

Supplementary Information

Reporting Summary

## Data Availability

The materials and methods, experimental procedures, characterization data, ^1^H, ^13^C, ^19^F NMR spectra and mass spectrometry data are available in the Supplementary Information. The X-ray crystallographic coordinates for structures reported in this study have been deposited at the Cambridge Crystallographic Data Centre (CCDC), under deposition numbers CCDC 2092437 (**2k**), CCDC 2092438 (**2****u**), CCDC 2092439 (**4a**), CCDC 2092440 (**4t**), CCDC 2131908 (**4ad’**), CCDC 2092441 (**7**), CCDC 2092442 (**8**), CCDC 2092443 (**9**), CCDC 2092444 (**11**) and CCDC 2092445 (**14**) and CCDC 2131952 (**15**). These data can be obtained free of charge from The Cambridge Crystallographic Data Centre via http://www.ccdc.cam.ac.uk/data_request/cif. The chemical structure similarity search is conducted in therapeutic target database (TTD) via http://db.idrblab.net/ttd/.
