# Supplementary material for: Divergent synthesis of benzazepines and bridged polycycloalkanones via dearomative rearrangement
Source: Nat Commun. 2022 Jul 29;13:4402. doi: 10.1038/s41467-022-31920-1 (PMC9338057; doi:10.1038/s41467-022-31920-1)
Supplement: Supplementary file 1 — Supplementary Information [file 41467_2022_31920_MOESM1_ESM.pdf]

Supplementary Information

Divergent Synthesis of Benzazepines and Bridged Polycycloalkanones via Dearomative Rearrangement

Qiu Shi[1], Zhehui Liao[1], Zhili Liu[1], Jiajia Wen[2], Chenguang Li[1], Jiamin He[1], Jiazhen Deng[1], Shan Cen[2], Tongxiang Cao[1], Jinming Zhou[3]*, Shifa Zhu[1]**

[1]Key Laboratory of Functional Molecular Engineering of Guangdong Province, School of Chemistry and Chemical Engineering, South China University of Technology, Guangzhou 510640, China;

[2]Institute of Medicinal Biotechnology, Chinese Academy of Medical Science, Beijing 100050, China;

[3]Key Laboratory of the Ministry of Education for Advanced Catalysis Materials, Department of Chemistry, Zhejiang Normal University, 688 Yingbin Road, Jinhua 321004, China;Correspondence and requests for materials should be addressed to: S. Zhu (email: zhusf@scut.edu.cn); J. Zhou (email: zhoujinming@zjnu.edu.cn); T. Cao (email: caotx@scut.edu.cn).

Table of contents

I. Supplementary Methods 2
General information 2
Optimization of reaction conditions 3
Experimental procedures 5
II. Supplementary Discussion 52
Control experiment 52
In situ IR spectroscopy 53
CO detection 54
Biological activity tests 55
Cytotoxicity analysis 55
Statistical analysis. 55
X-Ray diffraction analysis 59
III. Supplementary NMR Spectra 70
IV. Supplementary References 328

I. Supplementary Methods

General information

All reagents were purchased from commercial suppliers (such as Energy Chemical, MACKLIN, J&K Scientific) and used without further purification or prepared as described in the literature. Dichloromethane was distilled from calcium hydride. Toluene and THF was distilled from sodium. Column chromatography was carried out with silica gel (200-300 mesh). Analytical TLC was performed with silica gel GF254 plates, and the products were visualized by UV detection or potassium permanganate stain. All reactions were carried out under N_2 in a Schlenk tube unless otherwise noted. All reactions that required heating were proceeded in oil bath. ^{1}H, ^{13}C, ^{19}F NMR spectra were recorded on a Bruker AVANCE 400 and Bruker AVANCE 500, ^{1}H NMR and ^{13}C NMR chemical shifts were determined relative to internal standard TMS at δ 0.0 or chloroform at δ 7.26 for hydrogen, δ 77.16 for carbon. Chemical shifts (δ) are reported in ppm, and coupling constants (J) are in Hertz (Hz). The following abbreviations were used to explain the multiplicities: s = singlet, d = doublet, t = triplet, q = quartet, p = pentet, dd = doublet of doublets, dt = doublet of triplets, dq = doublet of quartets, td = triplets of doublets, tt = triplet of triplets, tp = triplet of pentets, ddd = doublet of doublet of doublets, ddt = doublet of doublet of triplets, dddd = doublet of doublet of doublet of doublets, m = multiplet, brs = broad. Infrared (IR) spectra are recorded on a Nicolet 210 spectrophotometer and were recorded in potassium bromide (KBr) pellet. Infrared (IR) spectra are recorded on a Nicolet 210 spectrophotometer and were recorded in potassium bromide (KBr) pellet. Mass spectra (MS) were obtained using ESI, DART mass spectrometer. Melting points were determined using a hot stage apparatus. Data for the single crystal structure determination were collected with an Agilent SuperNova, Dual, Cu at zero, AtlasS2 diffractometer. Operando IR analysis of the reaction mixture was conducted using a Mettler-Toledo ReactIR 15 instrument equipped with a 6.35 mm diameter DiComp probe. The cell lines HEK293T (ATCC CRL-3216), and SupT1 (ATCC CRL-1942) cells were from ATCC, and authenticated by STR profiling. The biological data were from at least three independent experiments, and all attempts for replications are successful. The data were collected from the platform system of Centro XS3 LB 960 and analyzed by using Prism (version 5.01, GraphPad Software, San Diego, CA, USA) without excluding any data.

Optimization of reaction conditions

Supplementary Table 1. Optimization of 2a[a]

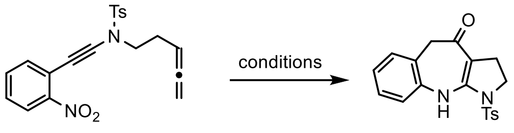

1a **2a**

entry	Time	T/(°C)	solvent	c/(mol/L)	**2a** (%)
1	48h	70	THF	0.025	41
2	48h	80	THF	0.025	80
3	6h	90	THF	0.025	81
4	4h	100	THF	0.025	53
5	6h	90	CH_3CN	0.025	64
6	6h	90	DCE	0.025	57
7	6h	90	PhMe	0.025	61
**8**	**6h**	**90**	**THF**	**0.033**	**81**
9	6h	90	THF	0.1	72
10	3h	90	THF	0.033	79
11	24h	90	THF	0.033	78

[a]**1a** (0.15 mmol) in solvent; under N_2; isolated yield.

Supplementary Table 2. Optimization of 4a[a]

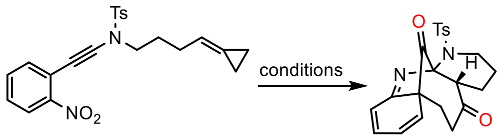

3a → **4a**

entry	cat. (mol %)	T/(°C)	solvent	**4a** (%)
1	--	90	Toluene	65
2	--	90	DMSO	63
3	--	90	THF	66
4	--	90	DMF	0
5	--	90	DCE	64
6	--	90	1,4-dioxane	49
7	$Rh_2(OAc)_4$	90	THF	64
8	$RuPPh_3Cl$	90	THF	61
9	$Cu(OAc)_2$	90	THF	62
10 [b]	--	90	THF	0
11 [c]	--	90	THF	70
12 [c]	--	110	THF	39
**13** [c]	**--**	**75**	**THF**	**74**
14 [c]	--	80	THF	71
15 [c,d]	--	60	THF	72

[a] **3a** (0.15 mmol) in 4.5 ml solvent was added under N_2 and stirred for 12 h; isolated yield. [b] The reaction was performed under air for 12 h; [c] The reaction was in normal THF; [d] The reaction was performed under N_2 for 60 h; **3a** (0.15 mmol) in 4.5 ml THF.

Experimental procedures

Typical procedure for the synthesis of S1[1]

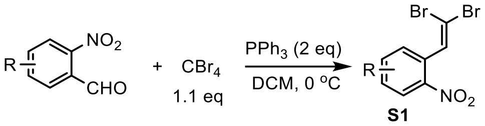

Dissolve the 2-nitrobenzaldehyde (1.00 g, 6.62 mmol) and CBr_4 (2.3 g, 7.2 mmol) in DCM (35 mL). Cool the solution to 0 °C in ice bath and PPh_3 (3.64 g, 13.2 mmol) was added in several portions to the mixture. The reaction was monitored by TLC until fully consumption of nitrobenzaldehyde (30 minutes). Hexanes (50 mL) was added to the mixture, and the solution was collected after filtration. The filtrate was concentrated under reduced pressure and the residue was chromatographed to give the desired 2-(2,2-dibromovinyl)-1-nitro-benzene(**S1**, EtOAc/petroleum ether = 1:20).

Typical procedure for preparation of S2[2]

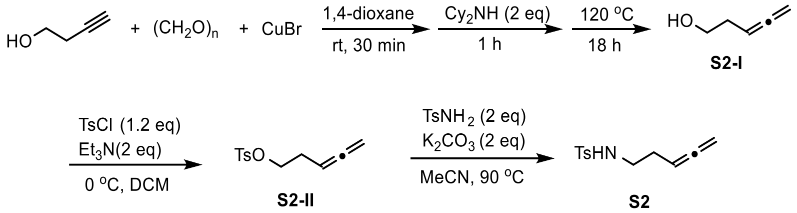

3-butyn-1-ol (50 mmol), dioxane (100 mL), paraformaldehyde (100 mmol) and CuBr (35 mmol) were added to a 250 mL two-neck flask equipped with a reflux condenser. After the mixture stirring at r.t. for 30 min, 2.0 eq Cy_2NH (100 mmol) was added and stirred for another 1 h at r.t. Then the mixture was heated to 120 ^oC for 18 h. The resulting mixture was cooled to r.t., then quenched with 8M HCl (aq) to pH≈1~2 (about 15 mL) and extracted with EtOAc (3*50 mL). The organic phases were collected and washed with brine (50 mL), dried with Na_2SO_4 and concentrated under reduced pressure. The residue was chromatographed with EtOAc/petroleum ether = 1:4 as the eluent to give the desired product **S2-I** (2.8g, 60%) as a colorless oil.

To a solution of **S2-I** (1 eq, 2.8 g) in DCM (60 mL), TEA (2 eq, 9.2 mL) and TsCl (1.2 eq, 7.6 g) was added in sequence at 0 ^oC. The resulting mixture was stirred at room temperature until totally consumption of starting metarial (about 12 h, monitored by TLC). The reaction mixture was quenched with water (60 mL) and extracted with DCM (3*40 mL). The organic phases were collected and washed with brine (40 mL), dried with Na_2SO_4 and concentrated under reduced pressure. The residue was used for next step without purification.

To a 100 mL Schlenk tube with a magnetic bar were added **S2-II**, $TsNH_2$ (2.0 eq, 11.4 g), CH_3CN (50 mL) and K_2CO_3 (2 eq, 9.2 g). The mixture was stirred at 90 ^oC for 12 h. After the reaction was finished (monitored by TLC), the reaction mixture was filtrated and filtrate washed with water (80 mL), extracted with EtOAc (3*50 mL). The combined organic layers were dried over Na_2SO_4, filtrated, concentrated in *vacuo*. The crude product was purified by silica gel column chromatography (EtOAc/petroleum ether = 1:5) to afford **S2** as colorless oil (60% yield).

Typical procedure for preparation of S3[3]

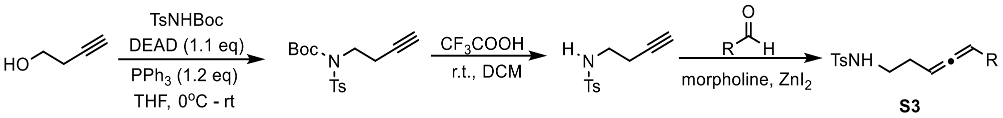

But-3-yn-1-ol (1.0 eq, 30 mmol), TsNHBoc (1.0 eq, 30 mmol) and PPh_3 (1.1 eq, 33 mmol) in 60 mL dry THF was stirred for 10 min. DEAD (1.1 eq) was then added at 0 °C. The mixture was warmed to r.t. and stirred for 8 h (monitored by TLC). After completion, the solvent was evaporated under reduced pressure and the residue was purified via silica gel column chromatography with EtOAc/petroleum ether = 1:5 as the eluent to afford the desired product tert-butyl but-3-yn-1-yl(tosyl)carbamate (4.2 g).

The tert-butyl but-3-yn-1-yl(tosyl)carbamate(1 g, 3.1 mmol) was dissolved in 50 mL DCM. TFA (1.15 mL, 5.0 eq) was added dropwisely to the mixture at 0 °C. The reaction was warmed to r.t. and stirred for 3 h. The reaction was quenched by $NaHCO_3$ (aq) (20 mL) and extracted with DCM (3*50 mL). The organic phase was washed with brine (50 mL), dried with Na_2SO_4 and concentrated under reduced pressure. The residue was chromatographed with EtOAc/petroleum ether =1:5 as the eluent to give the desired N-(but-3-yn-1-yl)-4-methylbenzenesulfonamide as a colorless oil (0.66 g).

A mixture of N-(but-3-yn-1-yl)-4-methylbenzenesulfonamide (5 mmol, 1.0 eq), aldehyde (10 mmol, 2.0 eq), morpholine (7.5 mmol, 1.5 eq) and ZnI_2 (4 mmol, 0.8 eq) in 70 mL toluene was heated at 130 °C. The reaction was monitored by TLC until completion (3 h). The solvent was removed under reduced pressure. The residue was chromatographed with EtOAc/petroleum ether = 1:5 as the eluent to give **S3** (10%-30%).

Typical procedure for preparation of S4

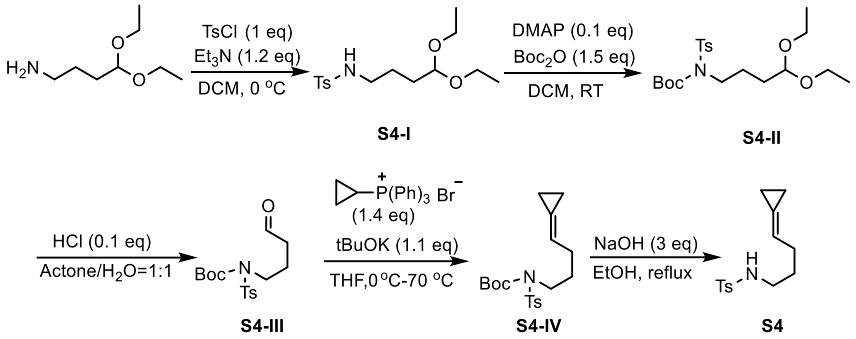

To a solution of 4,4-diethoxybutan-1-amine (3.4 ml, 20 mmol) and Et_3N (3.5 ml, 24 mmol) in DCM (100 ml) was added tosyl chloride (3.8 g, 20 mmol) over 10 min at 0 °C. The reaction mixture was warmed to r.t. and stirred for 1 h. After completion (1 h, monitored by TLC), the reaction was quenched with saturated $NaHCO_3$ aqueous solution (100 mL). The organic phase was collected, dried with Na_2SO_4 and concentrated under reduced pressure. The residue (**S4-I**) was used in the next step without further purification.

To a solution of crude **S4-I** in 50 mL DCM was added Boc_2O (30 mmol) and DMAP (2 mmol) at r.t. The reaction mixture was stirred for 0.5 h. After completion (0.5 h, monitored by TLC), the reaction was

quenched with water (50 mL). The organic phase was collected, dried with Na_2SO_4 and concentrated under reduced pressure. The residue (**S4-II**) was used in the next step without further purification.

To a 100 mL round-bottom flask with a magnetic bar were added **S4-II**, the mixed solvent (acetone/ water = 1:1, 50 mL) and 8M HCl solution (0.1 eq, 0.25 mL). The mixture was stirred at r.t. for 12 h. After the reaction was finished (12 h, monitored by TLC), the reaction mixture was quenched with saturated $NaHCO_3$ aqueous solution (40 mL), extracted with EtOAc (3*40 mL) . The combined organic phase was dried over Na_2SO_4, filtrated and concentrated in vacuo. The crude product was purified by column chromatography (silica gel, EtOAc/petroleum ether = 1:4) to afford **S4-III** as white solid (5.8 g).

A suspension of Wittig reagent (1.4 eq, 9 g) in dry THF (70 ml) was prepared under N_2 atmosphere. Potassium tert-butoxide (1.1 eq) solution (1M in THF, 18.6 mL) was then added slowly to the suspension using a syringe over 15 minutes at 0 ^oC. The mixture was warmed to r.t. and stirred for 1 h. **S4-III** (1 eq, 16.9 mmol) in THF solution was added slowly to the suspension using a syringe over 15 minutes. After stirring for 1 h at r.t., the reaction was heated to 70 ^oC in oil bath. After completion (12 h, monitored by TLC), the solution was filtrated through a pad of silica gel. The organic phases were concentrated in vacuo and purified over silica gel column chromatography (EtOAc/petroleum ether = 1:5) to give S4-IV 3.82 g (62%).

The isolated **S4-IV** (1 eq, 10.5 mmol) was dissolved in 30 mL EtOH, following by adding 3.0 eq NaOH (1.2 g). The mixture was reflux at 80 ^oC until completion (12 h, monitored by TLC). The reaction mixture was quenched with water (50 mL). The organic phase was collected, dried with Na_2SO_4 and concentrated under reduced pressure. The residue was purified by column chromatography (silica gel, EtOAc/petroleum ether = 1:4) to afford **S4** as yellow oil. (2.5 g, 90 %)

Typical procedure for preparation of α-substituded S5[4]

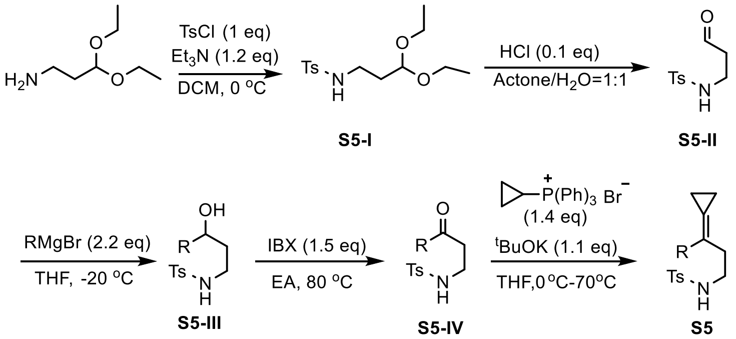

To a solution of 3, 3-diethoxypropan-1-amine (3.4 ml, 20 mmol) and tri-ethylamine (3.5 ml, 24 mmol) in DCM (100 ml) was added tosyl chloride (3.8 g, 20 mmol) over 10 min at 0 °C. The reaction mixture was warmed to r.t. and stirred for 1 h. After completion (1 h, monitored by TLC), the reaction was quenched with $NaHCO_3$ (aq) (100 ml). The organic phase was collected, dried with Na_2SO_4 and concentrated under reduced pressure. The residue (**S5-I**) was used in the next step without further purification.

To a 100 mL round-bottom flask with a magnetic bar were added **S5-I**, the mixed solvent (acetone/ water = 1:1, 50 mL) and 8M HCl solution (0.1 eq, 0.25 mL). The mixture was stirred at r.t. for 12 h. After

the reaction was finished (12 h, monitored by TLC), the reaction mixture was quenched with saturated $NaHCO_3$ aqueous solution (40 mL), extracted with EtOAc (3*40 mL). The combined organic phase was dried over Na_2SO_4, filtrated and concentrated in *vacuo*. The crude product was purified by column chromatography (silica gel, EtOAc/petroleum ether = 1/4) to afford **S5-II** as colorless oil (4.3 g, 94% yield).

Under nitrogen atmosphere, a solution of **S5-II** (1.0 eq 4.3 g, 18.8 mmol) in THF (60 mL) was cooled to -20 °C. The solution of RMgBr (2.2 eq, 41 mL, 1 mol/L in THF) was added dropwisely within 15 min at -20 °C. After reacting at -20 °C for 1 h, the solution was slowly warmed to 0 °C. Then the mixture was quenched with saturated NH_4Cl aqueous solution (30 mL), and extracted with EtOAc (3*30 mL). The extract was dried over Na_2SO_4 and evaporated under reduced pressure. The crude product **S5-III** was used in the next step without further purification.

To a 100 mL round-bottom flask with a magnetic bar were added **S5-III** (18.8 mmol), IBX (28.2 mmol, 1.5 eq) and EtOAc (50 mL). The mixture was stirred at 80 °C for 12 h. The mixture was filtrated through short celite. The solvent was evaporated in reduced pressure and purified via chromatography to afford the desired ketone **S5-IV** (silica gel, EtOAc/petroleum ether = 1:2, Two-step total yield: 72%).

A suspension of Wittig reagent (18.9 mmol, 1.4 eq) in dry THF (20 ml) was prepared under N_2 atmosphere. Potassium tert-butoxide solution (14.9 mL, 1.1 eq, 1M in THF) was then added slowly to the suspension using a syringe pump over 15 minutes at 0 °C. The mixture was warmed to r.t. and stirred for 1 h. **S5-IV** (1 eq, 13.5 mmol) in THF solution (20 mL) was added slowly to the suspension using a syringe pump over 15 minutes. After stirring for 1 h at r.t., the reaction was heated to 70 °C in oil bath. After completion (12 h, monitored by TLC), the solution was filtrated through a pad of silica gel. The organic phases were concentrated in vacuo and purified over silica gel column chromatography (EtOAc/petroleum ether = 1:5, 640mg, 18%).

Typical procedure for preparation of α-substituded S6

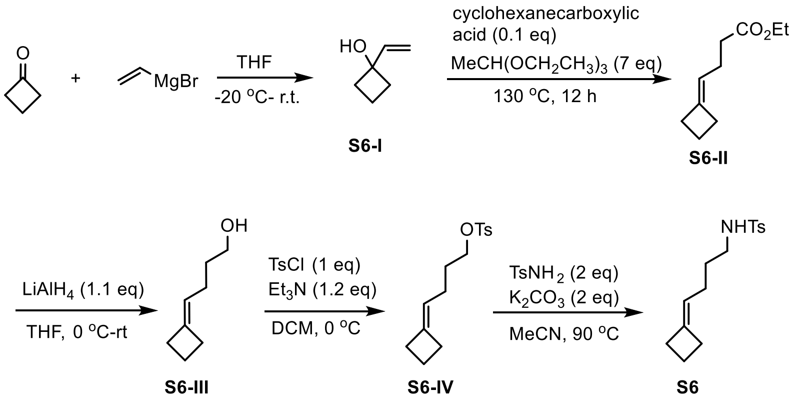

Under nitrogen atmosphere, a solution of cyclobutanone (1.0 eq, 1.4 g, 20 mmol) in THF (60 mL) was cooled to -20 °C. The solution of CH_2=CHMgBr (1.1 eq, 1 mol/L, 22 mL) was added dropwisely within 15 min at -20 °C. After reacting at -20 °C for 1 h, the solution was slowly warmed to 0 °C and stirred for 45 min. Then the mixture was quenched with saturated NH_4Cl aqueous solution (40 mL), and extracted with EtOAc (3*30 mL). The organic phases were dried over Na_2SO_4, filtrated and concentrated in vacuo. The residue was purified over silica gel column chromatography (petroleum ether/EtOAc = 10:1) to give **S6-I** (1.6 g, 82%).

According to a literature procedure [5], a solution of **S6-I** (1.0 eq, 1.6 g), triethyl orthoacetate (7 eq, 21 mL) and cyclohexanecarboxylic acid (0.1 eq, 0.2 g) was stirred under reflux conditions for 12 h. The reaction mixture was then cooled to room temperature and extracted three times with EtOAc (100 mL). The combined organic layers were washed with 10 % aqueous HCl (30 mL), saturated $NaHCO_3$ aqueous solution (30 mL), water and brine (30 mL). The organic layer was dried with Na_2SO_4 and concentrated under reduced pressure. The residue was purified by silica gel column chromatography using petroleum ether/EtOAc = 20:1 as eluent to afford the corresponding ester **S6-II** (0.94 g, 34%).

To a 25 mL Schlenk tube with a magnetic bar were added **S6-II** (1.0 eq, 0.94 g) and $LiAlH_4$ (6.1 mL, 1M in THF, 1.1 eq) in THF at 0 ^oC. Then the solution was warmed to r.t. After the reaction was finished (1 h, monitored by TLC), the mixture was quenched with water (10 mL) at 0 ^oC slowly, extracted with EtOAc (3*20 mL), dried over Na_2SO_4, filtrated and concentrated in *vacuo*. The crude product was purified by column chromatography (silica gel, petroleum ether/EtOAc = 6:1) to afford **S6-III** as colorless liquid (0.58 g, 82% yield).

To a solution of **S6-III** (1.0 eq, 0.58 g) and triethylamine (1.2 eq, 0.8 mL) in DCM (30 mL) was added tosyl chloride (1 eq, 0.86 g) over 10 min at 0 °C. The reaction mixture was warmed to r.t. and stirred for 1 h. After completion (1 h, monitored by TLC), the reaction was quenched with saturated $NaHCO_3$ (20 mL). The organic phase was collected, dried with Na_2SO_4 and concentrated under reduced pressure. The residue was used in the next step without further purification.

To a 100 mL Schlenk tube charged with a magnetic bar were added **S6-IV** (1.0 eq, 1.2 g), $TsNH_2$ (2.0 eq, 1.56 g), CH_3CN and K_2CO_3 (2 eq, 1.23 g). The mixture was stirred at 90 ^oC for 12 h. After the reaction was finished, the reaction mixture was washed with water (30 mL), extracted with EtOAc (3*30 mL), dried over Na_2SO_4, and concentrated in *vacuo*. The crude product was purified by column chromatography (silica gel, petroleum ether/EtOAc = 5:1) to afford **S6** as colorless oil (0.8 g, 65% yield).

Typical procedure for preparation of 1

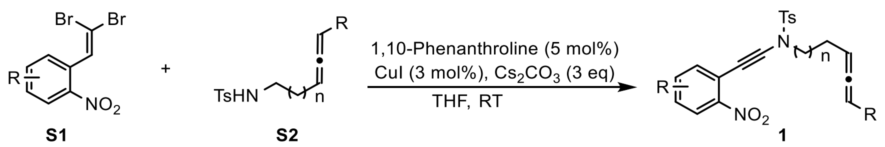

A mixture of dibromoalkene **S1** (1.1 eq), **S2** (1 eq), CuI (3 mol%), 1,10-phenantroline (5 mol%), caesium carbonate (3 eq) in THF (0.1 M) were stirred at r.t. for 12 h under N_2 atmosphere in a 25 mL Schlenk tube. After completion (monitored by TLC), the mixture was filtrated through celite, the solvent was evaporated in *vauco* at r.t. (Note：The ynamide is easily converted into a nitrone intermediate under heating). The residue was purified via chromatography to afford the desired product **1** in 30-90% yield.

4-methyl-N-((2-nitrophenyl)ethynyl)-N-(penta-3,4-dien-1-yl)benzenesulfonamide (1a)

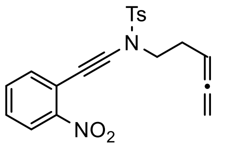

1a

Yield: 83% (311 mg), green liquid, R_f = 0.3 (EtOAc/ petroleum ether = 1:8). **^{1}H NMR (500 MHz, $CDCl_3$)** δ 8.08 (d, J = 8.5 Hz, 1H), 7.88 (d, J = 8.3 Hz, 2H), 7.59 – 7.52 (m, 2H), 7.34-7.38 (m, 3H), 5.09 (p, J = 6.8 Hz, 1H), 4.69 (dt, J = 6.5, 3.1 Hz, 2H), 3.58 (t, J = 7.3 Hz, 2H), 2.48 (qt, *J* = 7.2, 3.1 Hz, 2H), 2.44 (s, 3H). **^{13}C{^{1}H} NMR (126 MHz, $CDCl_3$)** δ 209.1, 147.6, 145.1, 134.6, 133.2, 133.1, 130.0 (2C), 127.7 (2C), 127.2, 124.8, 119.5, 91.3, 85.8, 75.9, 69.5, 51.1, 26.7, 21.7. **IR (KBr, cm^{-1}):** 2973, 2922, 2207, 1602, 1568 1365, 1340, 1165, 1084, 946, 851, 776, 743, 658, 573. **HRMS (ESI – TOF)/m/z:** $[M + H]^+$ calcd for $C_{20}H_{19}N_2O_4S$ 383.1060; found 383.1053.

Methyl 4-(((4-methyl-N-(penta-3,4-dien-1-yl)phenyl)sulfonamido)ethynyl)-3- nitrobenzoate (1b)

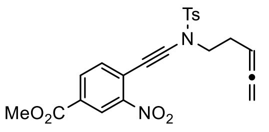

1b

Yield: 88% (232 mg), yellow oil, R_f = 0.4 (EtOAc/ petroleum ether = 1:4). **^{1}H NMR (500 MHz, $CDCl_3$)** δ 8.74 (d, J = 1.7 Hz, 1H), 8.17 (dd, J = 8.2, 1.7 Hz, 1H), 7.88 (d, J = 8.4 Hz, 2H), 7.60 (d, J = 8.3 Hz, 1H), 7.38 (d, J = 8.1 Hz, 2H), 5.08 (p, J = 6.7 Hz, 1H), 4.69 (dt, J = 6.5, 3.2 Hz, 2H), 3.97 (s, 3H), 3.61 (t, J = 7.3 Hz, 2H), 2.49 (qt, *J* = 6.9, 3.2 Hz, 2H), 2.45 (s, 3H). **^{13}C{^{1}H} NMR (126 MHz, $CDCl_3$)** δ 209.1, 164.7, 147.0, 145.3, 134.5, 133.3, 132.9, 130.1 (2C), 128.6, 127.7 (2C), 126.1, 123.9, 95.6, 85.7, 75.9, 70.7, 52.8, 51.1, 26.8, 21.7. **IR (KBr, cm^{-1}):** 2953, 2216, 1956, 1726, 1616, 1497, 1372, 1343, 1170, 1113, 904, 711, 669, 569, 542. **HRMS (ESI – TOF)/m/z:** $[M + Na]^+$ calcd for $C_{22}H_{20}N_2O_6SNa$ 463.0934; found 463.0930.

4-methyl-N-((2-nitro-4-(trifluoromethyl)phenyl)ethynyl)-N-(penta-3,4-dien-1-yl)benzenesulfonamide(1c)

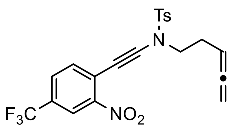

1c

Yield: 70% (189 mg), yellow oil, R_f = 0.9 (EtOAc/ petroleum ether = 1:4). **^{1}H NMR (500 MHz, $CDCl_3$)** δ 8.38 (s, 1H), 7.88 (d, J = 8.1 Hz, 2H), 7.78 (d, J = 8.2 Hz, 1H), 7.67 (d, J = 8.2 Hz, 1H), 7.38 (d, J = 8.1 Hz, 2H), 5.08 (p, J = 6.8 Hz, 1H), 4.69 (dt, J = 6.4, 3.1 Hz, 2H), 3.61 (t, J = 7.3 Hz, 2H), 2.49 (qt, *J* = 6.9, 3.2 Hz, 2H), 2.45 (s, 3H). **^{13}C{^{1}H} NMR (126 MHz, $CDCl_3$)** δ 209.1, 146.9, 145.4, 134.5, 133.6, 130.1 (2C), 129.4 (q, J = 3.4 Hz), 128.9 (q, J = 34.4 Hz), 127.7 (2C), 123.5, 122.8 (q, *J* = 272.5 Hz), 122.3 (q, J = 4.0 Hz), 121.7, 95.4, 85.7, 75.9, 70.1, 51.1, 26.8, 21.7. ^{19}F NMR (471 MHz, $CDCl_3$) δ -62.86. **IR (KBr, cm^{-1}):** 2218, 1628, 1535, 1363, 1323, 1170, 1136, 1086, 966, 806, 750, 668, 574. **HRMS (ESI – TOF)/m/z:** $[M + H]^+$ calcd for $C_{21}H_{18}F_3N_2O_4S$ 451.0934; found 451.0925

N-(4-cyclopropylidenebutyl)-4-methyl-N-((6-nitrobenzo[1][1,3]dioxol-5yl)ethynyl)benzenesulfonamide (1d)

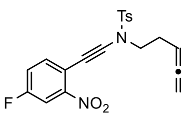

1d

Yield: 69% (166 mg), yellow oil, R_f = 0.8 (EtOAc/ petroleum ether = 1:5). **^{1}H NMR (500 MHz, $CDCl_3$)** δ 7.87 (d, J = 8.2 Hz, 2H), 7.82 (dd, J = 8.4, 2.7 Hz, 1H), 7.55 (dd, J = 8.8, 5.4 Hz, 1H), 7.37 (d, J = 8.1 Hz, 2H), 7.35 – 7.27 (m, 1H), 5.08 (p, J = 6.8 Hz, 1H), 4.69 (dt, J = 6.4, 3.1 Hz, 2H), 3.57 (t, J = 7.4 Hz, 2H), 2.51 – 2.41 (m, 5H). **^{13}C{^{1}H} NMR (126 MHz, $CDCl_3$)** δ 209.1, 160.3 (d, J = 253.3 Hz), 148.2 (d, J = 8.8 Hz), 145.1, 134.9, 134.7 (d, J = 7.6 Hz), 130.0 (2C), 127.7 (2C), 121.05 (d, J = 22.7 Hz), 115.81 (d, J = 3.8 Hz), 112.3 (d, J = 27.7 Hz), 91.0, 85.8, 75.9, 68.4, 51.0, 26.7, 21.7. **^{19}F NMR (471 MHz, $CDCl_3$)** δ -109.74. **IR (KBr, cm^{-1}):** 3094, 2926, 2226, 1956, 1597, 1579, 1369, 1210, 1113, 1091, 970, 812, 667, 545, 476. **HRMS (ESI – TOF)/m/z:** $[M + Na]^+$ calcd for $C_{20}H_{17}N_2FO_4SNa$ 423.0785; found 423.0781.

N-((4-chloro-2-nitrophenyl)ethynyl)-4-methyl-N-(penta-3,4-dien-1-yl)benzenesulfonamide (1e)

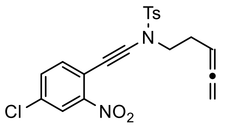

1e

Yield: 85% (213 mg), yellow oil, R_f = 0.8 (EtOAc/ petroleum ether = 1:5). **^{1}H NMR (500 MHz, $CDCl_3$)** δ 8.09 (d, J = 2.1 Hz, 1H), 7.87 (d, J = 8.1 Hz, 2H), 7.55 – 7.46 (m, 2H), 7.37 (d, J = 8.1 Hz, 2H), 5.08 (p, J = 6.8 Hz, 1H), 4.69 (dt, J = 6.5, 3.1 Hz, 2H), 3.58 (t, J = 7.3 Hz, 2H), 2.50 – 2.45 (m, 5H). **^{13}C{^{1}H} NMR (126 MHz, $CDCl_3$)** δ 209.1, 147.8, 145.2, 134.5, 134.0, 133.3, 132.8, 130.0 (2C), 127.7 (2C), 124.9, 118.2, 92.5, 85.7, 75.9, 69.0, 51.0, 26.8, 21.7. **IR (KBr, cm^{-1}):** 2827, 2208, 1695, 1596, 1525, 1367, 1097, 967, 1166, 1097, 967, 816, 722, 665, 573, 546. **HRMS (ESI – TOF)/m/z:** $[M + Na]^+$ calcd for $C_{20}H_{17}ClN_2O_4SNa$ 439.0490; found 439.0485.

N-((4-bromo-2-nitrophenyl)ethynyl)-4-methyl-N-(penta-3,4-dien-1-yl)benzenesulfonamide (1f)

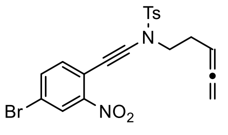

1f

Yield: 87% (242 mg), yellow solid, m. p. 80.6- 81.5 ℃. R_f = 0.5 (EtOAc/ petroleum ether = 1:4). **^{1}H NMR (500 MHz, $CDCl_3$)** δ 8.24 (d, J = 2.0 Hz, 1H), 7.86 (d, J = 8.3 Hz, 2H), 7.66 (dd, J = 8.4, 2.1 Hz, 1H), 7.41 (d, *J* = 8.4 Hz, 1H), 7.37 (d, *J* = 8.1 Hz, 2H), 5.07 (p, J = 6.8 Hz, 1H), 4.69 (dt, J = 6.4, 3.0 Hz, 2H), 3.57 (t, J = 7.3 Hz, 2H), 2.50 – 2.43 (m, 5H). **^{13}C{^{1}H} NMR (126 MHz, $CDCl_3$)** δ 209.1, 147.8, 145.2, 136.2, 134.5, 134.1, 130.0 (2C), 127.8, 127.67 (2C), 120.1, 118.6, 92.8, 85.7, 75.9, 69.2, 51.0, 26.8, 21.7. **IR (KBr, cm^{-1}):** 2984, 2221, 1955, 1598, 1549, 1523, 1369, 1340, 1170, 1090, 712, 666, 571, 543. **HRMS (ESI – TOF)/m/z:** $[M + Na]^+$ calcd for $C_{20}H_{17}BrN_2O_4SNa$ 482.9985; found 482.9980.

4-methyl-N-((4-methyl-2-nitrophenyl)ethynyl)-N-(penta-3,4-dien-1-yl)benzenesulfonamide (1g)

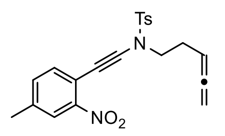

1g

Yield: 78% (175 mg), yellow oil, R_f = 0.6 (EtOAc/ petroleum ether = 1:4). **^{1}H NMR (500 MHz, $CDCl_3$)** δ 7.88 (d, J = 8.3 Hz, 3H), 7.43 (d, J = 7.9 Hz, 1H), 7.36 (d, J = 8.2 Hz, 3H), 5.09 (p, J = 6.8 Hz, 1H), 4.72 – 4.66 (m, 2H), 3.56 (t, J = 7.4 Hz, 2H), 2.47 (qt, *J* = 7.0, 3.5 Hz, 2H), 2.44 (s, 3H), 2.42 (s, 3H). **^{13}C{^{1}H} NMR (126 MHz, $CDCl_3$)** δ 209.1, 147.7, 145.0, 138.1, 134.6, 134.0, 133.1, 130.0 (2C), 127.7 (2C), 125.0, 116.5, 90.0, 85.9, 75.8, 69.1, 51.1, 26.7, 21.7, 21.1. **IR (KBr, cm^{-1}):** 2928, 2225, 1599, 1526, 1445, 1363, 1220, 1169, 1122, 1086, 986, 913, 963, 913, 815, 739, 666, 575, 551. **HRMS (ESI – TOF)/m/z:** $[M + Na]^+$ calcd for $C_{21}H_{20}N_2O_4SNa$ 419.1036; found 419.1032.

N-((4-methoxy-2-nitrophenyl)ethynyl)-4-methyl-N-(penta-3,4-dien-1-yl)benzenesulfonamide (1h)

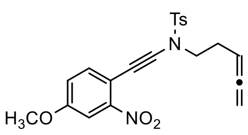

1h

Yield: 63% (147 mg), yellow oil, R_f = 0.6 (EtOAc/ petroleum ether = 1:4). **^{1}H NMR (400 MHz, $CDCl_3$)** δ 7.89 (d, J = 6.4 Hz, 2H), 7.58 (s, 1H), 7.48 (d, J = 6.9 Hz, 1H), 7.37 (d, J = 7.9 Hz, 2H), 7.12 (d, J = 8.7 Hz, 1H), 5.10 (p, *J* = 6.6 Hz, 1H), 4.74 – 4.67 (m, 2H), 3.89 (s, 3H), 3.56 (t, J = 7.3 Hz, 2H), 2.53 – 2.42 (m, 5H). **^{13}C{^{1}H} NMR (101 MHz, $CDCl_3$)** δ 209.1, 158.7, 149.0, 144.9, 134.7, 134.5, 129.9 (2C), 127.7 (2C), 120.4, 111.5, 109.0, 88.8, 85.9, 75.8, 68.3, 56.0, 51.1, 26.7, 21.6. **IR (KBr, cm^{-1}):** 1634, 1399, 1299, 1165, 1090, 970, 913, 739, 665, 586. **HRMS (ESI – TOF)/m/z:** $[M + Na]^+$ calcd for $C_{21}H_{20}N_2O_5SNa$ 435.0985; found 435.0978.

N-((5-methoxy-2-nitrophenyl)ethynyl)-4-methyl-N-penta-3,4-dien-1-yl)benzenesulfonamide (1j)

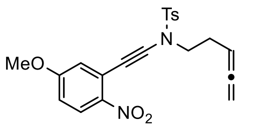

1j

Yield: 79% (195 mg), yellow oil, R_f = 0.7 (EtOAc/ petroleum ether = 1:5). **^{1}H NMR (500 MHz, $CDCl_3$)** δ 8.12 (d, J = 9.3 Hz, 1H), 7.88 (d, J = 8.2 Hz, 2H), 7.36 (d, J = 8.1 Hz, 2H), 6.95 (d, J = 2.7 Hz, 1H), 6.83 (dd, J = 9.3, 2.8 Hz, 1H), 5.09 (p, J = 6.8 Hz, 1H), 4.69 (dt, J = 6.5, 3.1 Hz, 2H), 3.90 (s, 3H), 3.58 (t, J = 7.4 Hz, 2H), 2.49 (qt, *J* = 7.3, 3.5 Hz, 2H), 2.44 (s, 3H). **^{13}C{^{1}H} NMR (126 MHz, $CDCl_3$)** δ 209.1, 163.1, 145.1, 140.9, 134.61, 130.0 (2C), 127.7 (2C), 127.3, 121.9, 116.7, 113.4, 91.4, 85.9, 75.8, 70.2, 56.1, 51.1, 26.7, 21.7. **IR (KBr, cm^{-1}):** 3064, 2940, 2224, 1956, 1659, 1604, 1577, 1415, 1169, 1076, 737.5, 672.4, 571.9, 546. **HRMS (ESI – TOF) /m/z:** $[M + Na]^+$ calcd for $C_{21}H_{20}N_2O_5SNa$ 435.0985; found 435.0983.

N-((5-chloro-2-nitrophenyl)ethynyl)-4-methyl-N-(penta-3,4-dien-1-yl)benzenesulfonamide (1k)

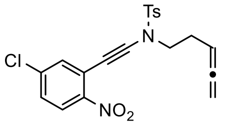

1k

Yield: 86% (215 mg), yellow solid, m.p. 87.0 – 89.1 ℃. R_f = 0.8 (EtOAc/ petroleum ether = 1:5). **^{1}H NMR (500 MHz, $CDCl_3$)** δ 8.06 (d, *J* = 8.9 Hz, 1H), 7.88 (d, *J* = 8.1 Hz, 2H), 7.49 (d, *J* = 2.3 Hz, 1H), 7.38 (d, *J* = 8.0 Hz, 2H), 7.30 (dd, *J* = 8.9, 2.2 Hz, 1H), 5.08 (p, *J* = 6.8 Hz, 1H), 4.69 (dt, *J* = 6.5, 2.8 Hz, 2H), 3.59 (t, *J* = 7.3 Hz, 2H), 2.51 – 2.43 (m, 5H). **^{13}C{^{1}H} NMR (126 MHz, $CDCl_3$)** δ 209.1, 145.6, 145.3, 139.7, 134.5, 132.4, 130.1 (2C), 127.7 (2C), 127.2, 126.2, 121.4, 93.2, 85.7, 75.9, 69.3, 51.0, 26.8, 21.7. **IR(KBr, cm^{-1}):** 2928, 2208, 1600, 1559, 1521, 1404, 1367, 1169, 1125, 1092, 975, 866, 815, 749, 713, 667, 568, 548. **HRMS (ESI – TOF) /m/z:** $[M + Na]^+$ calcd for $C_{20}H_{17}ClN_2O_4SNa$ 439.0490; found 439.0486.

N-((5-fluoro-2-nitrophenyl)ethynyl)-4-methyl-N-(penta-3,4-dien-1-yl)benzenesulfonamide (1l)

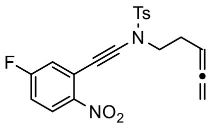

1l

Yield: 84% (168 mg), yellow oil, R_f = 0.6 (EtOAc/ petroleum ether = 1:4). **^{1}H NMR (500 MHz, $CDCl_3$)** δ 8.16 (dd, *J* = 9.2, 5.1 Hz, 1H), 7.88 (d, *J* = 8.0 Hz, 2H), 7.38 (d, *J* = 8.0 Hz, 2H), 7.20 (dd, *J* = 8.7, 2.7 Hz, 1H), 7.04 (ddd, *J* = 9.6, 7.3, 2.8 Hz, 1H), 5.08 (p, *J* = 6.8 Hz, 1H), 4.69 (dt, *J* = 6.5, 3.1 Hz, 2H), 3.59 (t, *J* = 7.3 Hz, 2H), 2.48 (qt, *J* = 7.0, 3.2 Hz, 2H), 2.45 (s, 3H). **^{13}C{^{1}H} NMR (101 MHz, $CDCl_3$)** 209.1, 164.7 (d, J = 257.3 Hz), 145.2, 143.7, 134.6, 130.0 (2C), 127.7 (2C), 127.6, 122.5 (d, J = 11.5 Hz), 119.2 (d, J = 25.1 Hz), 114.5 (d, J = 23.5 Hz), 93.3, 85.7, 75.9, 69.5, 51.1, 26.8, 21.7. ^{19}F NMR (471 MHz, $CDCl_3$) δ -103.93. **IR (KBr, cm^{-1}):** 2925, 2222, 1616, 1581, 1427, 1350, 1169, 912, 740, 669, 577, 555. **HRMS (ESI – TOF)/m/z:** $[M + Na]^+$ calcd for $C_{20}H_{17}FN_2O_4SNa$ 423.0785; found 423.0780.

Methyl 3-(((4-methyl-N-(penta-3,4-dien-1-yl)phenyl)sulfonamido)ethynyl)-4- nitrobenzoate (1m)

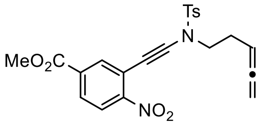

1m

Yield: 55% (145 mg), yellow solid, m.p. 78.6 – 80.5 °C, R_f = 0.5 (EtOAc/ petroleum ether = 1:4). **^{1}H NMR (500 MHz, $CDCl_3$)** δ 8.16 (s, 1H), 8.12 (d, *J* = 8.6 Hz, 1H), 7.97 (dd, *J* = 8.6, 1.5 Hz, 1H), 7.89 (d, J = 8.0 Hz, 2H), 7.38 (d, J = 8.1 Hz, 2H), 5.09 (p, J = 6.8 Hz, 1H), 4.70 (dt, J = 6.4, 3.0 Hz, 2H), 3.99 (s, 3H), 3.60 (t, J = 7.3 Hz, 2H), 2.52 – 2.44 (m, 5H). **^{13}C{^{1}H} NMR (126 MHz, $CDCl_3$)** δ 209.1, 164.8, 149.8, 145.2, 134.5, 134.3, 134.1, 130.1 (2C), 127.8, 127.7 (2C), 124.9, 119.8, 92.5, 85.7, 75.9, 69.0, 52.9, 51.1, 26.8, 21.7. **IR (KBr, cm^{-1}):** 2222, 1727, 1526, 1365, 1274, 1170, 1121, 667, 548. **HRMS (ESI – TOF) /m/z:** $[M + H]^+$ calcd for $C_{22}H_{21}N_2O_6S$ 441.1115; found 441.1107.

N-((2-fluoro-6-nitrophenyl)ethynyl)-4-methyl-N-(penta-3,4-dien-1-yl)benzenesulfonamide (1n)

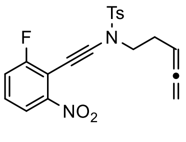

1n

Yield: 95 % (228 mg), yellow oil, R_f = 0.6 (EtOAc/ petroleum ether = 1:4). **^{1}H NMR (500 MHz, $CDCl_3$)** δ 7.93 (d, J = 8.0 Hz, 3H), 7.40 – 7.30 (m, 4H), 5.09 (p, J = 6.7 Hz, 1H), 4.69 (dt, *J* = 6.0, 2.8 Hz, 2H), 3.62 (t, *J* = 7.3 Hz, 2H), 2.49 (qt, *J* = 7.0, 3.2 Hz, 2H), 2.45 (s, 3H). **^{13}C{^{1}H} NMR (126 MHz, $CDCl_3$)** δ 209.1, 162.2 (d, *J* = 253.1 Hz), 148.3, 145.1, 134.4, 123.0(2C), 127.8 (2C), 127.1 (d, *J* = 8.6 Hz), 120.5 (d, *J* = 3.4 Hz), 120.1 (d, *J* = 21.8 Hz), 109.6 (d, *J* = 20.3 Hz), 96.6 (d, *J* = 4.3 Hz), 85.8, 75.9, 63.1, 51.2, 26.7, 21.7. **^{19}F NMR (471 MHz, $CDCl_3$)** δ -106.46. **IR (KBr, cm^{-1}):** 2926, 2226, 1632, 1530, 1361, 1171, 1087, 852, 746, 672, 573. **HRMS (ESI – TOF) /m/z:** $[M + Na]^+$ calcd for $C_{20}H_{17}FN_2O_4SNa$ 423.0785; found 423.0780.

4-methyl-N-((2-nitropyridin-3-yl)ethynyl)-N-(penta-3,4-dien-1-yl)benzenesulfonamide (1o)

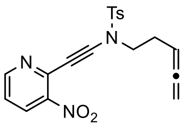

1o

Yield: 35% (40 mg), yellow oil, R_f = 0.5 (EtOAc/ petroleum ether = 1:4). **^{1}H NMR (500 MHz, $CDCl_3$)** δ 8.76 (d, J = 4.6 Hz, 1H), 8.39 (d, J = 8.4 Hz, 1H), 7.95 (d, J = 7.6 Hz, 2H), 7.37 (d, J = 8.0 Hz, 2H), 7.34 (dd, J = 8.4, 4.7 Hz, 1H), 5.08 (p, J = 6.7 Hz, 1H), 4.72 – 4.65 (m, 2H), 3.65 (t, J = 7.5 Hz, 2H), 2.48 (qt, J = 6.6, 3.2 Hz, 2H), 2.44 (s, 3H). **^{13}C{^{1}H} NMR (126 MHz, $CDCl_3$)** δ 209.1, 153.8, 145.3, 144.2, 138.2, 134.5, 132.6, 130.04 (2C), 127.8 (2C), 121.2, 92.7, 85.7, 76.0, 71.7, 51.2, 26.9, 21.7. IR (KBr, cm^{-1}): 2978, 2864, 1718, 1591, 1525, 1455, 1356, 1266, 1167, 1082, 918, 816, 756, 668. **HRMS (ESI – TOF) /m/z:** $[M + H]^+$ calcd for $C_{19}H_{18}N_3O_4S$ 384.1013; found 384.1014.

4-methyl-N-((2-nitrophenyl)ethynyl)-N-(5-phenylpenta-3,4-dien-1-yl) benzenesulfonamide (1p)

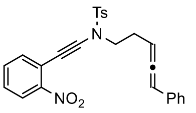

1p

Yield: 35% (160 mg), yellow oil, R_f = 0.7 (EtOAc/ petroleum ether = 1:4). **^{1}H NMR (500 MHz, $CDCl_3$)** δ 8.06 (d, J = 8.3 Hz, 1H), 7.87 (d, J = 8.3 Hz, 2H), 7.53 – 7.47 (m, 2H), 7.37 – 7.30 (m, 3H), 7.25 (d, J = 4.3 Hz, 4H), 7.16 (q, J = 4.3 Hz, 1H), 6.17 – 6.11 (m, 1H), 5.56 (q, J = 6.7 Hz, 1H), 3.72 – 3.57 (m, 2H), 2.62 (qd, J = 7.1, 2.8 Hz, 2H), 2.41 (s, 3H). **^{13}C{^{1}H} NMR (126 MHz, $CDCl_3$)** δ 205.9, 147.6, 145.1, 134.6, 134.2, 133.2, 133.0, 130.0 (2C), 128.5 (2C), 127.7 (2C), 127.1, 127.0, 126.8 (2C), 124.8, 119.5, 95.7, 91.3, 90.8, 69.7, 51.1, 27.4, 21.7. **IR (KBr, cm^{-1}):** 2987, 2209, 1763, 1594, 123, 1367, 1343, 1245, 1168, 965, 915, 748. **HRMS (ESI – TOF) /m/z:** $[M + H]^+$ calcd for $C_{26}H_{23}N_2O_4S$ 459.1373; found 459.1363.

N-(hepta-3,4-dien-1-yl)-4-methyl-N-((2-nitrophenyl) ethynyl) benzenesulfonamide (1q)

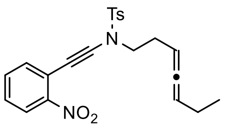

1q

Yield: 65% (160 mg), yellow oil, R_f = 0.7 (EtOAc/ petroleum ether = 1:4). **^{1}H NMR (500 MHz, $CDCl_3$)** δ 8.08 (d, J = 8.4 Hz, 1H), 7.88 (d, J = 8.0 Hz, 2H), 7.55 (d, J = 4.4 Hz, 2H), 7.36 (m, 3H), 5.21 – 5.15 (m, 1H), 5.09 (dq, *J* = 6.6, 3.3 Hz, 1H), 3.56 (t, *J* = 7.4 Hz, 2H), 2.47 (qd, *J* = 6.8, 2.7 Hz, 2H), 2.44 (s, 3H), 1.97 (pd, *J* = 7.2, 3.0 Hz, 2H), 0.98 (t, J = 7.4 Hz, 3H). **^{13}C{^{1}H} NMR (126 MHz, $CDCl_3$)** δ 204.3, 147.6, 145.0, 134.6, 133.2, 133.0, 130.0 (2C), 127.7 (2C), 127.1, 124.8, 119.6, 94.0, 91.5, 87.2, 69.5, 51.2, 27.5, 21.8, 21.68, 13.4. **IR (KBr, cm^{-1}):** 2966, 2927, 2870, 2199, 1603, 1523, 1367, 1343, 1170, 966, 748, 666, 580, 545. **HRMS (ESI – TOF) /m/z:** $[M + H]^+$ calcd for $C_{22}H_{23}N_2O_4S$ 411.1373; found 411.1365.

N-(hexa-4,5-dien-1-yl)-4-methyl-N-((2-nitrophenyl)ethynyl)benzenesulfonamide (1r)

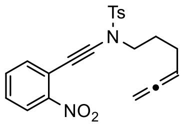

1r

Yield: 65 % (112.6 mg), yellow oil, R_f = 0.6 (EtOAc/ petroleum ether = 1:4). **^{1}H NMR (500 MHz, $CDCl_3$)** δ 8.09 (d, J = 8.3 Hz, 1H), 7.88 (d, J = 8.1 Hz, 2H), 7.55 (d, J = 4.3 Hz, 2H), 7.39 – 7.32 (m, 3H), 5.11 (p, J = 6.6 Hz, 1H), 4.70 (dt, J = 6.6, 3.3 Hz, 2H), 3.53 (t, J = 7.1 Hz, 2H), 2.44 (s, 3H), 2.09 (qt, J = 6.7, 3.1 Hz, 2H), 1.93 (p, J = 7.2 Hz, 2H). **^{13}C{^{1}H} NMR (126 MHz, $CDCl_3$)** δ 208.6, 147.6, 145.0, 134.6, 133.2, 133.0, 130.0 (2C), 127.7 (2C), 127.1, 124.8, 119.6, 91.6, 88.7, 75.6, 69.3, 51.2, 27.0, 24.8, 21.7. **IR (KBr, cm^{-1}):** 2926, 2225, 1632, 1530, 1361, 1295, 1257, 1171, 852, 810, 746, 573. **HRMS (ESI – TOF) /m/z:** [M + Na]$^+$ calcd for $C_{21}H_{20}N_2O_4SNa$ 419.1036; found 419.1031.

N-((2-nitrophenyl)ethynyl)-N-(penta-3,4-dien-1-yl)methanesulfonamide (1s)

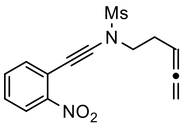

1s

Yield: 59% (90.6 mg), yellow oil, R_f = 0.5 (EtOAc/ petroleum ether = 1:4). **^{1}H NMR (500 MHz, $CDCl_3$)** δ 8.11 (d, J = 8.3 Hz, 1H), 7.61 – 7.53 (m, 2H), 7.40 (t, J = 6.3 Hz, 1H), 5.18 (p, J = 6.8 Hz, 1H), 4.76 (dt, J = 6.4, 3.0 Hz, 2H), 3.72 (t, J = 7.1 Hz, 2H), 3.25 (s, 3H), 2.57 (dt, J = 6.9, 3.3 Hz, 2H). **^{13}C{^{1}H} NMR (126 MHz, $CDCl_3$)** δ 209.2, 147.8, 133.2 (2C), 127.5, 124.9, 119.1, 90.3, 85.9, 76.0, 69.8, 51.0, 39.1, 27.2. **IR (KBr, cm^{-1}):** 2931, 2208, 1606, 1565, 1356, 1161, 962, 912, 776, 550. **HRMS (ESI – TOF) /m/z:** [M-H]$^-$ calcd for $C_{14}H_{13}N_2O_4S$ 305.0601; found 305.0603.

Typical procedure for preparation of 1t

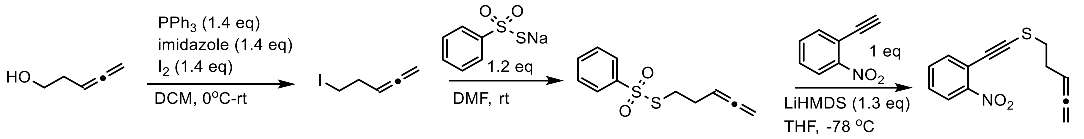

Dissolve triphenylphosphine (1.4 eq, 7 mmol) and imidazole (1.4 eq, 7 mmol) in DCM (20 mL). Cool the solution to 0 °C, then iodine (1.4 eq, 7 mmol) was added slowly to the stirring mixture and reacted for 15 min. Add penta-3,4-dien-1-ol (1 eq, 5 mmol) slowly at 0 °C. Warm the mixture to room temperature and react for 3 h. Add petroleum ether (20 mL) to the reaction solution. Filter the suspension through celite and concentrate the solution in vacuo at r.t. and purified over silica gel column chromatography (petroleum ether, 0.79 g, 82%).

To a solution of benzenesulfonic acid sodium salt (1.2 eq, 4.92 mmol) in DMF (20 mL) was slowly added the solution of 5-iodopenta-1,2-diene (1 eq, 4.1 mmol) at r.t. and stirred for 3 h. Upon completion (monitored by TLC), water was added (200 mL) and extracted with EtOAc (3*20 mL). The combined organic phases were washed with brine (50 mL). The organic phase was dried over Na_2SO_4, filtered and concentrated under reduced pressure. The resulting crude mixture was purified via silica gel column chromatography (silica gel, EtOAc/petroleum ether = 1:10, 0.8 g, 81%).

LiHMDS (4.3 mL, 1 M in THF, 1.3 eq) was slowly added to a stirred solution of alkyne (1 eq, 0.45 g, 3.3 mmol) in dry THF (0.2 M) over 10 min at -78 °C under nitrogen atmosphere. After 15 min, the lithium acetaminide was treated with the corresponding benzene thiosulfonate(1.1 eq, 0.8 g) in dry THF (1.1 M) which was added drop-wise over 15 min and the solution was then stirred for a further 15 min at -78 °C. The mixture was allowed to warm to room temperature and stirred for 1 h. The reaction mixture was quenched with saturated NH_4Cl (aq) (10 mL) and then extracted with THF (2*10 mL). The combined

organic layers were dried over Na_2SO_4, filtered and concentrated under reduced pressure to give a residue which was purified by silica gel column chromatography to afford the desired alkynyl thioethers (EtOAc/petroleum ether = 1:30, 0.57 g, **1t**: 78%).

((2-nitrophenyl)ethynyl)(penta-3,4-dien-1-yl)sulfane (1t)

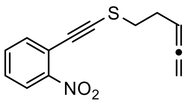

1t

Yield: 78% (570 mg), green oil, R_f = 0.3 (EtOAc/ petroleum ether = 1:30). **^{1}H NMR (500 MHz, $CDCl_3$)** δ 8.10 (d, *J* = 8.4 Hz, 1H), 7.58 – 7.53 (m, 2H), 7.44 – 7.35 (m, 1H), 5.26 (p, *J* = 6.6 Hz, 1H), 4.78 (dt, *J* = 6.5, 3.2 Hz, 2H), 2.99 (t, *J* = 7.2 Hz, 2H), 2.60 (qt, *J* = 6.9, 3.2 Hz, 2H). **^{13}C{^{1}H} NMR (126 MHz, $CDCl_3$)** δ 208.8, 148.3, 133.5, 133.0, 127.5, 124.8, 119.3, 90.6, 90.5, 87.8, 76.1, 35.3, 28.0. **IR (KBr, cm^{-1}):** 3141, 1638, 1522, 1401, 1258, 1219, 1085, 844, 743, 615. **HRMS (ESI – TOF)/m/z:** $[M+H]^+$ calcd for $C_{13}H_{12}NO_2S$ 246.0583; found 246.0579.

N-(5-cyclopropylpenta-3,4-dien-1-yl)-4-methyl-N-((2-nitrophenyl)ethynyl)benzenesulfonamide (1u)

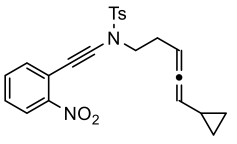

1u

Yield: 78% (67 mg), geeen oil. Rf = 0.3 (EtOAc/ petroleum ether = 1:8). **^{1}H NMR (400 MHz, $CDCl_3$)** δ 8.01 (d, *J* = 8.3 Hz, 1H), 7.80 (d, *J* = 8.3 Hz, 2H), 7.47 (d, *J* = 4.0 Hz, 2H), 7.32 – 7.25 (m, 3H), 5.07 (q, *J* = 5.9 Hz, 1H), 4.90 (ddt, *J* = 7.5, 6.1, 2.8 Hz, 1H), 3.48 (td, *J* = 7.5, 2.0 Hz, 2H), 2.43 – 2.33 (m, 5H), 1.12 (qtd, *J* = 8.2, 4.8, 1.5 Hz, 1H), 0.64 – 0.51 (m, 2H), 0.27 – 0.17 (m, 2H). **^{13}C{^{1}H} NMR (101 MHz, $CDCl_3$)** δ 204.2, 147.6, 145.0, 134.6, 133.2, 133.0, 130.0 (2C), 127.7 (2C), 127.1, 124.8, 119.6, 96.6, 91.4, 88.4, 69.5, 51.1, 27.6, 21.7, 9.4, 6.8, 6.7. **IR (KBr, cm^{-1}):** 2920, 2815, 2203, 1647, 1590, 1521, 1385, 1349, 1165, 1085, 569, 542. **HRMS(ESI-TOF)/m/z:** $[M+H]^+$ calcd for $C_{23}H_{23}N_2O_4S$ 423.1373; found 423.1365.

Typical procedure for preparation of 2

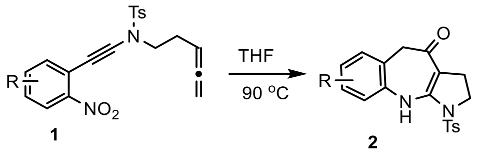

1 **2**

A dried 25 mL Schlenk tube was flushed with N_2 three times. The solution of **1** (1 eq) in the THF (0.03 M) was added to the tube under nitrogen atmosphere. The resulting mixture was put in a 90 °C oil bath and stirred for 6 h. After cooled to room temperature, the mixture was transferred to a 25 mL round bottom flask and evaporated under reduced pressure. The residue was purified by column chromatography (silica gel, petroleum ether/EtOAc = 1:1) to give **2**.

1-tosyl-2,3,5,10-tetrahydrobenzo²pyrrolo[3,2-f]azepin-4(1H)-one (2a)

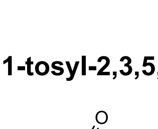

2a

Yield: 81% (36.7 mg), gray solid, m.p. 164.0 – 164.6 °C, R_f = 0.6 (EtOAc/ petroleum ether = 1:1). **^{1}H NMR (500 MHz, $CDCl_3$)** δ 9.00 (s, 1H), 7.68 (d, J = 8.0 Hz, 2H), 7.31 (d, J = 7.9 Hz, 3H), 7.23 (d, J = 6.5 Hz, 2H), 7.16 (d, J = 7.9 Hz, 1H), 3.71 (t, J = 8.4 Hz, 2H), 3.60 (s, 2H), 2.52 (t, J = 8.4 Hz, 2H), 2.42 (s, 3H). **^{13}C{^{1}H} NMR (126 MHz, $CDCl_3$)** δ 187.1, 150.5, 145.5, 138.2, 133.2, 130.8, 130.4 (2C), 127.60, 127.2 (2C), 126.2, 124.8, 119.5, 102.6, 48.2, 47.8, 25.1, 21.7. IR (KBr, cm^{-1}): 2924, 1727, 1627, 1402, 1266, 1160, 915, 754, 588, 546. **HRMS (ESI – TOF)/m/z:** $[M + H]^+$ calcd for $C_{19}H_{19}N_2O_3S$ 355.1111; found 355.1112

Methyl 4-oxo-1-tosyl-1,2,3,4,5,10-hexahydrobenzo[2]pyrrolo[3,2-f]azepine-8-carboxylate (2b)

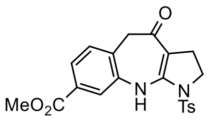

2b

Yield: 75% (64.1 mg), brown oil. R_f = 0.3 (EtOAc/ petroleum ether = 1:1). **^{1}H NMR (500 MHz, $CDCl_3$)** δ 9.12 (s, 1H), 7.87 (d, J = 7.9 Hz, 1H), 7.85 (s, 1H), 7.70 (d, J = 8.1 Hz, 2H), 7.33 (d, J = 8.1 Hz, 2H), 7.30 (d, J = 7.9 Hz, 1H), 3.96 (s, 3H), 3.72 (t, J = 8.4 Hz, 2H), 3.66 (s, 2H), 2.54 (t, J = 8.5 Hz, 2H), 2.41 (s, 3H). **^{13}C{^{1}H} NMR (126 MHz, $CDCl_3$)** δ 186.0, 166.1, 150.6, 145.6, 138.3, 133.1, 131.0, 130.4 (2C), 129.8, 129.6, 127.2 (2C), 127.1, 120.8, 102.9, 52.4, 48.4, 47.8, 25.1, 21.7. **IR (KBr, cm^{-1}):** 2953, 1727, 1602, 1541, 1497, 1437, 1355, 1295, 1226, 1159, 1108, 814, 761, 667, 592, 542. **HRMS (ESI – TOF) /m/z:** [M + Na]$^+$ calcd for $C_{21}H_{20}N_2O_5SNa$ 435.0985; found 435.0980.

1-tosyl-8-(trifluoromethyl)-2,3,5,10-tetrahydrobenzo[2]pyrrolo[3,2-f]azepin-4(1H)-one (2c)

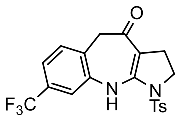

2c

Yield: 60% (42.3 mg), gray solid, m.p. 122.3 -122.6 °C. R_f = 0.4 (EtOAc/ petroleum ether = 1:1). **^{1}H NMR (500 MHz, $CDCl_3$)** δ 9.11 (s, 1H), 7.69 (d, J = 8.1 Hz, 2H), 7.47 (d, J = 8.0 Hz, 1H), 7.40 (s, 1H), 7.37 (d, J = 8.3 Hz, 1H), 7.34 (d, J = 8.2 Hz, 2H), 3.73 (t, J = 8.5 Hz, 2H), 3.66 (s, 2H), 2.56 (t, J = 8.5 Hz, 2H), 2.44 (s, 3H). **^{13}C{^{1}H} NMR (126 MHz, $CDCl_3$)** δ 185.9, 150.4, 145.8, 138.6, 133.1, 131.5, 130.5 (2C), 130.2 (q, J = 33.2 Hz), 128.5, 127.2 (2C), 122.7 (q, J = 3.7 Hz), 122.5 (q, J = 271.5 Hz), 116.7 (q, J = 3.7 Hz), 103.2, 48.1, 47.8, 25.0, 21.7. **^{19}F NMR (471 MHz, $CDCl_3$)** δ -116.16. **IR (KBr, cm^{-1}):** 3059, 2970, 1584, 1545, 1502, 1404, 1334, 1267, 1164, 1123, 1080, 936, 887, 819, 741, 587. **HRMS (ESI – TOF) /m/z:** [M + H]$^+$ calcd for $C_{20}H_{18}F_3N_2O_3S$ 423.0985; found 423.0986.

8-fluoro-1-tosyl-2,3,5,10-tetrahydrobenzo[2]pyrrolo[3,2-f]azepin-4(1H)-one (2d)

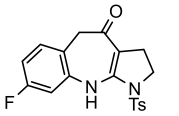

2d

Yield: 70% (46 mg), gray solid, m.p. 155.8– 156.6 °C. R_f = 0.4 (EtOAc/ petroleum ether = 1:1). **^{1}H NMR (500 MHz, $CDCl_3$)** δ 8.96 (s, 1H), 7.67 (d, J = 8.1 Hz, 2H), 7.34 (d, J = 8.1 Hz, 2H), 7.18 (dd, J = 8.5, 6.0 Hz, 1H), 6.94 (td, J = 8.3, 2.5 Hz, 1H), 6.89 (dd, J = 9.1, 2.5 Hz, 1H), 3.71 (t, J = 8.5 Hz, 2H), 3.57 (s, 2H), 2.52 (t, J = 8.5 Hz, 2H), 2.44 (s, 3H). **^{13}C{^{1}H} NMR (126 MHz, $CDCl_3$)** δ 187.0, 161.8 (d, *J* = 246.6 Hz), 150.2, 145.6, 139.3 (d, *J* = 10.0 Hz), 133.1, 132.1 (d, *J* = 9.0 Hz), 130.5 (2C), 127.2 (2C), 120.6 (d, *J* = 2.9 Hz), 113.2 (d, *J* = 21.4 Hz), 106.6 (d, *J* = 24.5 Hz), 102.8, 47.8, 47.5, 25.1, 21.7. **^{19}F NMR (471 MHz, $CDCl_3$)** δ -114.57. **IR (KBr, cm^{-1}):** 3327, 3057, 2976, 2923, 2246, 1729, 1614, 1496, 1400, 1266, 1164, 971, 745, 583, 543. **HRMS (ESI – TOF) /m/z:** [M + Na]$^+$ calcd for $C_{19}H_{17}FN_2O_3SNa$ 395.0836; found 395.0832.

8-chloro-1-tosyl-2,3,5,10-tetrahydrobenzo[2]pyrrolo[3,2-f]azepin-4(1H)-one (2e)

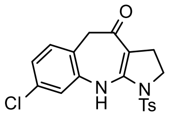

2e

Yield: 82% (52.9 mg), brown solid, m.p. 151.2– 152.6 °C. R_f = 0.6 (EtOAc/ petroleum ether = 1:1). **^{1}H NMR (500 MHz, $CDCl_3$)** δ 8.97 (s, 1H), 7.68 (d, J = 7.9 Hz, 2H), 7.34 (d, J = 7.9 Hz, 2H), 7.21 – 7.12 (m, 3H), 3.70 (t, J = 8.4 Hz, 2H), 3.57 (s, 2H), 2.52 (t, J = 8.5 Hz, 2H), 2.44 (s, 3H). **^{13}C{^{1}H} NMR (126 MHz, $CDCl_3$)** δ 186.7, 150.2, 145.7, 139.2, 133.1, 133.0, 131.9, 130.5 (2C), 127.2 (2C), 126.2, 123.3, 119.5, 102.9, 47.8, 47.7, 25.1, 21.7. **IR (KBr, cm^{-1}):** 1606, 1533, 1485, 1401, 1352, 1241, 1159, 1096, 811, 737, 668, 587. **HRMS (ESI – TOF) /m/z:** [M + Na]$^+$ calcd for $C_{19}H_{17}ClN_2O_3SNa$ 411.0541; found 411.0536.

8-bromo-1-tosyl-2,3,5,10-tetrahydrobenzo^{2}pyrrolo[3,2-f]azepin-4(1H)-one (2f)

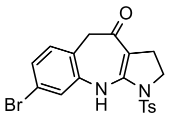

2f

Yield: 80% (61.5 mg), brown solid, m.p. 162.5– 163.9 °C. R_f = 0.5 (EtOAc/ petroleum ether = 1:1). **^{1}H NMR (500 MHz, $CDCl_3$)** δ 8.96 (s, 1H), 7.68 (d, J = 8.1 Hz, 2H), 7.37 – 7.30 (m, 4H), 7.09 (d, J = 8.0 Hz, 1H), 3.70 (t, J = 8.5 Hz, 2H), 3.55 (s, 2H), 2.52 (t, J = 8.5 Hz, 2H), 2.44 (s, 3H). **^{13}C{^{1}H} NMR (126 MHz, $CDCl_3$)** δ 186.5, 150.2, 145.7, 139.5, 133.1, 132.1, 130.5 (2C), 129.1, 127.2 (2C), 123.8, 122.4, 120.6, 102.9, 47.8 (2C), 25.1, 21.7. **IR (KBr, cm^{-1}):** 2979, 1603, 1573, 1531, 1484, 1354, 1402, 1159, 1114, 1086, 1159, 1114, 1086, 741, 667, 591. **HRMS (ESI – TOF) /m/z:** $[M + H]^+$ calcd for $C_{19}H_{18}BrN_2O_3S$ 433.0216; found 433.0211.

8-methyl-1-tosyl-2,3,5,10-tetrahydrobenzo^{2}pyrrolo[3,2-f]azepin-4(1H)-one (2g)

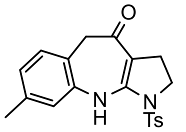

2g

Yield: 75 % (51.5 mg), brown solid, m.p. 101.8 - 102.1 °C, R_f = 0.4 (EtOAc/ petroleum ether = 1:1). **^{1}H NMR (500 MHz, $CDCl_3$)** δ 8.94 (s, 1H), 7.69 (d, J = 8.1 Hz, 2H), 7.32 (d, J = 8.1 Hz, 2H), 7.10 (d, J = 7.8 Hz, 1H), 7.03 (d, J = 7.8 Hz, 1H), 6.96 (s, 1H), 3.69 (t, J = 8.4 Hz, 2H), 3.55 (s, 2H), 2.51 (t, J = 8.5 Hz, 2H), 2.42 (s, 3H), 2.38 (s, 3H). **^{13}C{^{1}H} NMR (126 MHz, $CDCl_3$)** δ 187.4, 150.4, 145.5, 138.0, 137.7, 133.2, 130.6, 130.4 (2C), 127.3 (2C), 127.1, 121.7, 119.9, 102.3, 47.8 (2C), 25.1, 21.7, 20.9. **IR (KBr, cm^{-1}):** 2922, 1605, 1499, 1402, 1351, 1240, 1160, 1115, 814, 735, 670, 587. **HRMS (ESI – TOF) /m/z:** $[M + Na]^+$ calcd for $C_{20}H_{20}N_2O_3SNa$ 391.1087; found 391.1082.

8-methoxy-1-tosyl-2,3,5,10-tetrahydrobenzo^{2}pyrrolo[3,2-f]azepin-4(1H)-one (2h)

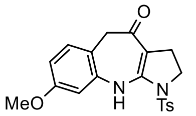

2h

Yield: 72% (45.1 mg), brown oil, R_f = 0.4 (EtOAc/ petroleum ether = 1:1). **^{1}H NMR (500 MHz, $CDCl_3$)** δ 8.92 (s, 1H), 7.69 (d, J = 8.1 Hz, 2H), 7.32 (d, J = 8.0 Hz, 2H), 7.12 (d, J = 8.4 Hz, 1H), 6.78 (dd, J = 8.4, 2.4 Hz, 1H), 6.67 (d, J = 2.4 Hz, 1H), 3.83 (s, 3H), 3.69 (t, J = 8.4 Hz, 2H), 3.52 (s, 2H), 2.51 (t, J = 8.4 Hz, 2H), 2.43 (s, 3H). **^{13}C{^{1}H} NMR (126 MHz, $CDCl_3$)** δ 187.7, 159.2, 150.3, 145.5, 139.0, 133.1, 131.6, 130.4 (2C), 127.2 (2C), 116.9, 112.2, 104.8, 102.4, 55.7, 47.8, 47.3, 25.2, 21.7. **IR (KBr, cm^{-1}):** 2963, 1608, 1499, 1405, 1351, 1277, 1116, 1034, 812, 739, 670, 582, 541. **HRMS (ESI – TOF) /m/z:** $[M + Na]^+$ calcd for $C_{20}H_{20}N_2O_4SNa$ 407.1036; found 407.1030.

Procedure for preparation of 2i (Note: **1i** will deteriorate during separation)

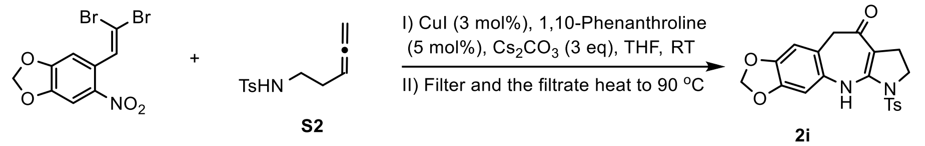

A mixture of 5-(2,2-dibromovinyl)-6-nitrobenzo[d][1,3]dioxole (1.1 eq), **S2** (1 eq), CuI (3 mol%), 1,10-phenantroline (5 mol%), caesium carbonate (3 eq) in THF (0.1 M) were stirred at r.t. for 12 h under N_2 atmosphere in a 25 mL Schlenk tube. After completion (12 h, monitored by TLC), the mixture was filtrated through celite, the resulting filtrate was added to a dried 25 mL schlenk tube under nitrogen atmosphere and put in a 90 °C oil bath stirring for 6 h. After cooled to room temperature, the mixture was transfered to a 25 mL round bottom flask and evaporated under reduced pressure. The residue was purified by column chromatography (silica gel, petroleum ether/EtOAc = 1:1) to give **2i**.

6-tosyl-5,7,8,10-tetrahydro-[1,3]dioxolo[4',5':4,5]benzo[1,2-b]pyrrolo[3,2-f]azepin-9(6H)-one (2i)

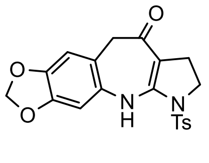

2i

Yield: 22% (16.8 mg), brown oil. R_f = 0.8 (EtOAc/ petroleum ether = 1:2). **^{1}H NMR (500 MHz, $CDCl_3$)** δ 8.79 (s, 1H), 7.67 (d, *J* = 8.1 Hz, 2H), 7.33 (d, *J* = 8.0 Hz, 2H), 6.67 (s, 2H), 6.00 (s, 2H), 3.69 (t, *J* = 8.4 Hz, 2H), 3.46 (s, 2H), 2.49 (t, *J* = 8.4 Hz, 2H), 2.43 (s, 3H). **^{13}C{^{1}H} NMR (126 MHz, $CDCl_3$)** δ 186.8, 150.6, 147.2, 146.3, 145.5, 133.3, 132.2, 130.4 (2C), 127.2 (2C), 118.1, 109.5, 102.1, 101.8, 100.6, 47.9, 47.7, 25.1, 21.7. **IR (KBr, cm^{-1}):** 2981, 2917, 1733, 1649, 1590, 1481, 1351, 1247, 1164, 1037, 938, 813, 669, 590. **HRMS (ESI – TOF)/m/z:** $[M + H]^+$ calcd for $C_{20}H_{19}N_2O_5S$ 399.1009; found 399.1007.

7-methoxy-1-tosyl-2,3,5,10-tetrahydrobenzo[2]pyrrolo[3,2-f]azepin-4(1H)-one (2j)

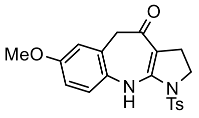

2j

Yield: 64% (29.8 mg), brown solid, m.p. 143.6 – 144.0 °C. R_f = 0.8 (EtOAc/ petroleum ether = 1:2). **^{1}H NMR (500 MHz, $CDCl_3$)** δ 8.88 (s, 1H), 7.67 (d, J = 8.1 Hz, 2H), 7.31 (d, J = 8.0 Hz, 2H), 7.08 (d, J = 8.7 Hz, 1H), 6.85 (dd, J = 8.7, 2.8 Hz, 1H), 6.75 (d, J = 2.7 Hz, 1H), 3.80 (s, 3H), 3.70 (t, J = 8.4 Hz, 2H), 3.56 (s, 2H), 2.50 (t, J = 8.4 Hz, 2H), 2.42 (s, 3H). **^{13}C{^{1}H} NMR (126 MHz, $CDCl_3$)** δ 186.7, 158.1, 150.8, 145.5, 133.3, 131.5, 130.4 (2C), 127.2 (2C), 126.4, 120.6, 114.5, 113.8, 101.8, 55.6, 48.4, 47.9, 25.1, 21.7. **IR (KBr, cm^{-1}):** 3337, 2918, 2215, 1586, 1532, 1500, 1353, 1248, 1158, 1035, 812, 666, 585. **HRMS (ESI – TOF) /m/z:** $[M + Na]^+$ calcd for $C_{20}H_{20}N_2O_4SNa$ 407.1036; found 407.1031.

7-chloro-1-tosyl-2,3,5,10-tetrahydrobenzo[2]pyrrolo[3,2-f]azepin-4(1H)-one (2k)

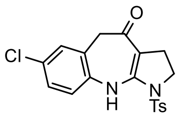

2k

Yield: 83% (64.5 mg), brown solid, m.p. 149.5 – 152.2 °C. R_f = 0.6 (EtOAc/ petroleum ether = 1:1). **^{1}H NMR (500 MHz, $CDCl_3$)** δ 8.97 (s, 1H), 7.66 (d, J = 8.0 Hz, 2H), 7.33 (d, J = 8.0 Hz, 2H), 7.29 – 7.27 (m, 1H), 7.22 (s, 1H), 7.10 (d, J = 8.4 Hz, 1H), 3.70 (t, J = 8.4 Hz, 2H), 3.56 (s, 2H), 2.51 (t, J = 8.4 Hz, 2H), 2.43 (s, 3H). **^{13}C{^{1}H} NMR (126 MHz, $CDCl_3$)** δ 186.2, 150.4, 145.7, 136.9, 133.1, 131.3, 130.5, 130.4 (2C), 127.6, 127.2 (2C), 126.4, 120.7, 102.8, 47.9, 47.8, 25.1, 21.7. **IR (KBr, cm^{-1}):** 2953, 1727, 1602, 1541, 1497, 1437, 1355, 1295, 1226, 1159, 1108, 814, 761, 667, 592, 542. **HRMS (ESI – TOF)/m/z:** $[M + Na]^+$ calcd for $C_{19}H_{17}ClN_2O_3SNa$ 411.0541; found 411.0537.

7-fluoro-1-tosyl-2,3,5,10-tetrahydrobenzo[2]pyrrolo[3,2-f]azepin-4(1H)-one (2l)

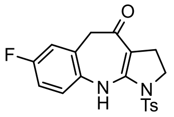

2l

Yield: 72% (44.1 mg), gray solid, m.p. 93.5 - 94.7 °C, R_f = 0.4 (EtOAc/ petroleum ether = 1:1). **^{1}H NMR (500 MHz, $CDCl_3$)** δ 8.94 (s, 1H), 7.67 (d, J = 8.1 Hz, 2H), 7.33 (d, J = 8.0 Hz, 2H), 7.13 (dd, J = 8.7, 4.7 Hz, 1H), 7.01 (td, *J* = 8.1, 2.7 Hz, 1H), 6.95 (dd, *J* = 8.7, 2.8 Hz, 1H), 3.70 (t, J = 8.4 Hz, 2H), 3.57 (s, 2H), 2.51 (t, J = 8.4 Hz, 2H), 2.43 (s, 3H). **^{13}C{^{1}H} NMR (126 MHz, $CDCl_3$)** δ 186.2, 160.8 (d, J = 245.4 Hz), 150.7, 145.6, 134.4 (d, J = 2.6 Hz), 133.1, 130.4 (2C), 127.2 (2C), 127.0 (d, J = 8.3 Hz), 120.9 (d, J = 8.7 Hz), 116.9 (d, J = 23.0 Hz), 114.5 (d, J = 23.3 Hz), 102.4, 48.0, 47.8, 25.1, 21.7. **^{19}F NMR (471 MHz, $CDCl_3$)** δ -116.91. **IR (KBr, cm^{-1}):** 2923, 1586, 1497, 1402, 1353, 1246, 1159, 1105, 814, 737, 667, 587, 546. **HRMS (ESI – TOF) /m/z:** $[M + Na]^+$ calcd for $C_{19}H_{17}FN_2O_3SNa$ 395.0836; found 395.0834

Methyl 4-oxo-1-tosyl-1,2,3,4,5,10-hexahydrobenzo^{2}pyrrolo[3,2-f]azepine-7-carboxylate (2m)

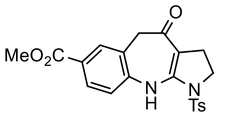

2m

Yield: 68% (40.9 mg), gray solid, m.p. 128.4 – 129.5 °C. R_f = 0.3 (EtOAc/ petroleum ether = 1:1). **^{1}H NMR (500 MHz, $CDCl_3$)** δ 9.14 (s, 1H), 7.98 (dd, J = 8.3, 1.9 Hz, 1H), 7.94 (s, 1H), 7.67 (d, J = 8.1 Hz, 2H), 7.32 (d, J = 8.1 Hz, 2H), 7.21 (d, J = 8.3 Hz, 1H), 3.92 (s, 3H), 3.72 (t, J = 8.5 Hz, 2H), 3.65 (s, 2H), 2.53 (t, J = 8.5 Hz, 2H), 2.43 (s, 3H). **^{13}C{^{1}H} NMR (126 MHz, $CDCl_3$)** δ 186.4, 166.2, 149.7, 145.7, 142.1, 133.0, 132.7, 130.5 (2C), 129.1, 127.8, 127.2 (2C), 124.2, 119.5, 103.6, 52.2, 48.1, 47.8, 25.2, 21.7. **IR (KBr, cm^{-1}):** 2953, 1726, 1606, 1528, 1442, 1365, 1726, 1167, 1121, 1091, 965, 912, 821, 667, 549. **HRMS (ESI – TOF) /m/z:** [M + H]$^+$ calcd for $C_{21}H_{21}N_2O_5S$ 413.1166; found 413.1164.

6-fluoro-1-tosyl-2,3,5,10-tetrahydrobenzo^{2}pyrrolo[3,2-f]azepin-4(1H)-one (2n)

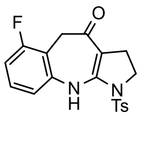

2n

Yield: 59% (25.8 mg), brown oil, R_f = 0.4 (EtOAc/ petroleum ether = 1:1). **^{1}H NMR (500 MHz, $CDCl_3$)** δ 9.03 (s, 1H), 7.67 (d, J = 8.1 Hz, 2H), 7.32 (d, J = 8.0 Hz, 2H), 7.26 – 7.22 (m, 1H), 7.02 – 6.93 (m, 2H), 3.71 (t, J = 8.5 Hz, 2H), 3.64 (s, 2H), 2.52 (t, J = 8.5 Hz, 2H), 2.43 (s, 3H). **^{13}C{^{1}H} NMR (126 MHz, $CDCl_3$)** δ 186.3, 160.5 (d, J = 247.1 Hz), 150.2, 145.6, 140.4 (d, J = 5.6 Hz), 133.0, 130.4 (2C), 128.2 (d, J = 9.7 Hz), 127.2 (2C), 115.0 (d, J = 3.4 Hz), 112.9 (d, J = 19.8 Hz), 112.3 (d, J = 23.1 Hz), 103.5, 47.8, 38.5 (d, J = 4.1 Hz), 25.2, 21.7. **^{19}F NMR (471 MHz, $CDCl_3$)** δ -116.16. **IR (KBr, cm^{-1}):** 2923, 1614, 1476, 1399, 1354, 1261, 1161, 1984, 782, 669, 586. **HRMS (ESI – TOF) /m/z:** [M + Na]$^+$ calcd for $C_{19}H_{17}FN_2O_3SNa$ 395.0836; found 395.0831.

6-tosyl-5,7,8,10-tetrahydropyrido[3,2-b]pyrrolo[3,2-f]azepin-9(6H)-one (2o)

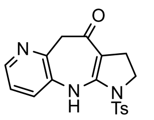

2o

Yield: 52% (20 mg), gray solid, m.p. 161.7-162.4 °C. R_f = 0.6 (EtOAc/ petroleum ether = 1:1). **^{1}H NMR (500 MHz, $CDCl_3$)** δ 8.87 (s, 1H), 8.48 (d, J = 4.6 Hz, 1H), 7.66 (d, J = 8.3 Hz, 2H), 7.48 (s, 1H), 7.33 (d, J = 8.1 Hz, 2H), 7.29 – 7.27 (m, 1H), 3.90 (s, 2H), 3.72 (t, J = 8.5 Hz, 2H), 2.56 (t, J = 8.5 Hz, 2H), 2.44 (s, 3H). **^{13}C{^{1}H} NMR (126 MHz, $CDCl_3$)** δ 185.4, 149.5, 147.3, 145.7, 144.0, 134.0, 133.0, 130.5 (2C), 127.2 (2C), 126.9, 122.2, 103.6, 51.7, 47.8, 25.2, 21.7. **IR (KBr, cm^{-1}):** 2922, 1733, 1598, 1568, 1489, 1352, 1268, 1160, 1113, 1083, 810, 752, 668, 587, 544. **HRMS (ESI – TOF) /m/z:** [M + H]$^+$ calcd for $C_{18}H_{18}N_3O_3S$ 356.1064; found 356.1068.

5-phenyl-1-tosyl-2,3,5,10-tetrahydrobenzo^{2}pyrrolo[3,2-f]azepin-4(1H)-one (2p)

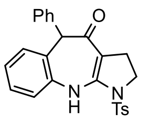

2p

Yield: 47% (23.8 mg), gray solid, m.p. 134.4 -134.8 °C. R_f = 0.4 (EtOAc/ petroleum ether = 1:4). **^{1}H NMR (500 MHz, $CDCl_3$)** δ 9.09 (s, 1H), 7.42 (t, J = 7.6 Hz, 1H), 7.37 – 7.27 (m, 4H), 7.23 – 7.16 (m, 4H), 7.14 (d, J = 7.9 Hz, 2H), 6.88 (d, J = 7.5 Hz, 2H), 5.03 (s, 1H), 3.75 (td, J = 10.4, 3.8 Hz, 1H), 3.32 (q, J = 10.0 Hz, 1H), 2.72 – 2.56 (m, 2H), 2.39 (s, 3H). **^{13}C{^{1}H} NMR (126 MHz, $CDCl_3$)** δ 188.5, 148.5, 145.0, 138.1, 137.6, 132.9, 132.7, 130.3 (2C), 128.4, 128.2 (2C), 127.0 (2C), 126.7, 126.6 (2C), 126.3, 125.5, 120.5, 100.8, 63.9, 47.4, 25.2, 21.6. **IR (KBr, cm^{-1}):** 2958, 2862, 1575, 1542, 1493, 1450, 1409, 1352, 1303, 1160, 1120, 1080, 912, 912, 753, 668, 628, 586. **HRMS (ESI – TOF) /m/z:** [M + H]$^+$ calcd for $C_{25}H_{23}N_2O_3S$ 431.1424; found 431.1421.

5-ethyl-1-tosyl-2,3,5,10-tetrahydrobenzo^{2}pyrrolo[3,2-f]azepin-4(1H)-one (2q)

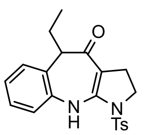

2q

Yield: 75% (35.7 mg), brown oil, R_f = 0.4 (EtOAc/ petroleum ether = 1:4). **^{1}H NMR (500 MHz, $CDCl_3$)** δ 9.02 (s, 1H), 7.67 (d, *J* = 7.9 Hz, 2H), 7.34 – 7.28 (m, 3H), 7.22 – 7.11 (m, 3H), 3.77 (td, *J* = 10.6, 4.7 Hz, 1H), 3.68 (q, *J* = 9.8 Hz, 1H), 3.43 (t, *J* = 8.0 Hz, 1H), 2.58 (td, *J* = 9.6, 4.7 Hz, 1H), 2.54 – 2.46 (m, 1H), 2.41 (s, 3H), 1.77 (dt, *J* = 14.3, 7.3 Hz, 1H),

1.60 (dt, J = 14.9, 7.8 Hz, 1H), 0.76 (t, J = 7.4 Hz, 3H). **^{13}C NMR{^{1}H} (126 MHz, CDCl$_3$)** δ 190.8, 148.4, 145.5, 136.8, 133.2, 131.4, 130.3 (2C), 127.6, 127.4, 127.2 (2C), 125.8, 120.0, 100.9, 60.9, 47.8, 25.4, 23.3, 21.6, 12.0. **IR (KBr, cm^{-1}):** 2964, 2926, 2871, 1604, 1539, 1490, 1402, 1352, 1225, 1161, 1093, 1052, 755, 669, 587. **HRMS (ESI – TOF) /m/z:** [M + H]$^+$ calcd for $C_{21}H_{23}N_2O_3S$ 383.1424; found 383.1430.

1-tosyl-1,2,3,4,6,11-hexahydro-5H-benzo^{2}pyrido[3,2-f]azepin-5-one (2r)

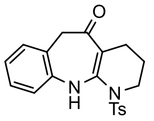

2r

Yield: 63% (36.7 mg), brown solid, m.p. 102.6 - 102.9 °C. R$_f$ = 0.6 (EtOAc/ petroleum ether = 1:1). **^{1}H NMR (500 MHz, CDCl$_3$)** δ 8.71 (s, 1H), 7.57 (d, J = 8.1 Hz, 2H), 7.35 – 7.27 (m, 3H), 7.24 (d, J = 8.0 Hz, 2H), 3.72 (t, J = 5.8 Hz, 2H), 3.69 (s, 2H), 2.38 (s, 3H), 2.03 (t, J = 7.3 Hz, 2H), 1.27 (p, J = 6.7 Hz, 3H). **^{13}C{^{1}H} NMR (126 MHz, CDCl$_3$)** δ 189.3, 149.4, 145.1, 138.8, 135.5, 130.3 (2C), 129.1, 128.4, 127.5, 127.0 (2C), 126.6, 119.8, 105.1, 48.2, 46.9, 21.6, 21.1, 20.8. **IR (KBr, cm^{-1}):** 2927, 1580, 1524, 1490, 1369, 1156, 1089, 1009, 756, 669, 560. **HRMS (ESI – TOF) /m/z:** [M + Na]$^+$ calcd for $C_{20}H_{20}N_2O_3SNa$ 391.1087; found 391.1085.

1-(methylsulfonyl)-2,3,5,10-tetrahydrobenzo^{2}pyrrolo[3,2-f]azepin-4(1H)-one (2s)

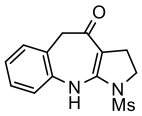

2s

Yield: 73% (33.8 mg), gray solid, m.p. 135.3 – 136.5 °C. Rf = 0.4 (EtOAc/ petroleum ether = 1:4).**^{1}H NMR (500 MHz, CDCl$_3$)** δ 8.67 (s, 1H), 7.26 – 7.18 (m, 3H), 7.04 (d, J = 7.7 Hz, 1H), 3.87 (t, J = 8.6 Hz, 2H), 3.65 (s, 2H), 3.07 (s, 3H), 2.84 (t, J = 8.6 Hz, 2H). **^{13}C{^{1}H} NMR (126 MHz, CDCl$_3$)** δ 187.20, 150.08, 137.92, 130.83, 127.60, 126.37, 124.80, 119.57, 101.92, 48.31, 48.22, 38.04, 25.35. **IR (KBr, cm^{-1}):** 3054, 3008, 1647, 1607, 1575, 1538, 1409, 1346, 1240, 1154, 1115, 1068, 1036, 965, 755. **HRMS (ESI – TOF) /m/z:** [M + H]$^+$ calcd for $C_{13}H_{15}N_2O_3S$ 279.0798; found 279.0798.

Typical procedure for preparation of 2t, 2t'.

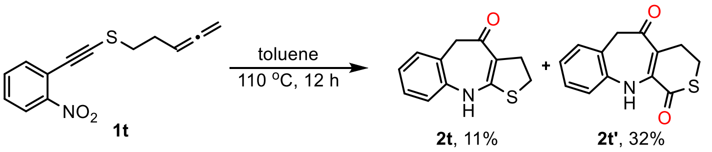

1t **2t**, 11% **2t'**, 32%

To a 25 mL Schlenk tube with a magnetic bar were added **1t** (0.2 mmol, 1.0 eq) and PhMe (6 mL) at r.t. under N_2 atmosphere. The mixture was stirred at 110 °C for 12 h. After the reaction was completed (monitored by TLC), the solvent was concentrated in vacuo. The crude product was purified by column chromatography (silica gel, petroleum ether/EtOAc = 2:1) to afford **2t** (11%) and **2t'** (32%).

2,3,5,10-tetrahydro-4H-benzo[b]thieno[3,2-f]azepin-4-one (2t)

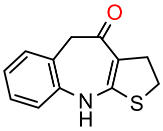

2t

Yield: 11% (4.8 mg), yellow oil, R$_f$ = 0.3 (EtOAc/ petroleum ether = 1:1). **^{1}H NMR (500 MHz, CDCl$_3$)** δ 7.25 – 7.16 (m, 3H), 7.07 (s, 1H), 6.96 (d, J = 7.5 Hz, 1H), 3.66 (s, 2H), 3.23 – 3.15 (m, 4H). **^{13}C{^{1}H} NMR (126 MHz, CDCl$_3$)** δ 184.4, 159.7, 139.8, 130.6, 127.2, 126.0, 125.0, 118.8, 113.4, 48.3, 36.1, 31.1. **IR (KBr, cm^{-1}):** 3120, 1712, 1637, 1398, 1276, 1047, 969, 825, 730. **HRMS (ESI – TOF)/m/z:** [M+H]$^+$ calcd for $C_{12}H_{12}NOS$ 218.0634; found 218.0629.

3,4,6,11-tetrahydrobenzo[b]thiopyrano[4,3-f]azepine-1,5-dione (2t')

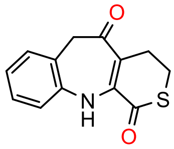

2t'

Yield: 32% (15.7 mg), yellow oil, R_f = 0.3 (EtOAc/ petroleum ether = 1:3). **^{1}H NMR (500 MHz, $CDCl_3$)** δ 8.15 (s, 1H), 7.30 – 7.24 (m, 3H), 7.10 (d, *J* = 7.4 Hz, 1H), 3.67 (s, 2H), 3.17 (t, *J* = 6.1 Hz, 2H), 3.06 (t, *J* = 6.1 Hz, 2H). **^{13}C{^{1}H} NMR (126 MHz, $CDCl_3$)** δ 191.8, 189.0, 141.0, 138.7, 129.6, 127.8, 126.8, 124.4, 120.4, 119.9, 49.1, 27.9, 23.4. **IR (KBr, cm^{-1}):** 3144, 1637, 1578, 1402, 1261, 1166, 1087, 759, 615. **HRMS (ESI – TOF)/m/z:** [M+H]$^+$ calcd for $C_{13}H_{12}NO_2S$ 246.0583; found 246.0580.

5-cyclopropyl-1-tosyl-2,3,5,10-tetrahydrobenzo[b]pyrrolo[3,2-f]azepin-4(1H)-one (2u)

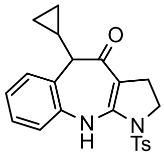

2u

Yield: 75% (42 mg), white solid, m.p. 119.1 – 120.5 °C. R_f = 0.4 (EtOAc/ petroleum ether = 1:3). **^{1}H NMR (500 MHz, $CDCl_3$)** δ 9.01 (s, 1H), 7.67 (d, J = 7.8 Hz, 2H), 7.33 (t, J = 7.8 Hz, 1H), 7.28 (d, J = 8.0 Hz, 2H), 7.23 (t, J = 6.8 Hz, 2H), 7.17 (d, J = 7.9 Hz, 1H), 3.82 – 3.68 (m, 2H), 2.62 (d, J = 10.2 Hz, 2H), 2.57 – 2.47 (m, 1H), 2.40 (s, 3H), 1.28 (m, 1H), 0.52 (m, 1H), 0.40 (m, 2H), 0.11 – 0.05 (m, 1H). **^{13}C{^{1}H} NMR (126 MHz, $CDCl_3$)** δ 190.0, 148.9, 145.5, 137.0, 133.2, 130.8, 130.3 (2C), 128.1, 127.6, 127.3 (2C), 125.9, 119.9, 101.2, 63.7, 47.9, 25.4, 21.6, 11.9, 4.8, 4.1. **IR (KBr, cm^{-1}):** 918, 2853, 1806, 1651, 1573, 1541, 1456, 1391, 1345, 1161, 810, 669, 589, 545. **HRMS (ESI – TOF) /m/z:** [M + H]$^+$ calcd for $C_{22}H_{23}N_2O_3S$ 395.1424; found 395.1419.

Typical procedure for preparation of 3

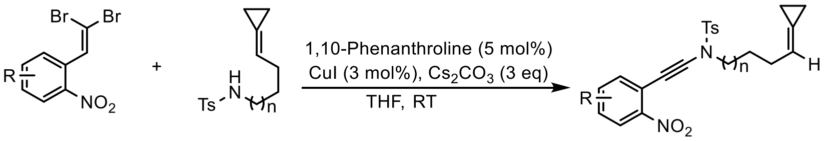

S1 S3 3

A mixture of dibromoalkene **S1** (1.1 eq), tosyl amide **S3** (1 eq), copper iodide (3 mol%), 1,10-phenantroline (5 mol%) and caesium carbonate (3 eq) in THF (0.1 M) were stirred at r.t. for 12 h under N_2 atmosphere in a 25 mL Schlenk tube. After completion (12 h, monitored by TLC), the mixture was filtrated through a short pad of celite. The solvent was evaporated in vauco at r.t.(The solvent needs to be removed under vacuum at room temperature, the ynamide is easily converted into an nitrone intermediate under heating) and purified via column chromatography to afford the desired ynamide **3** (40-90% yield).

N-(4-cyclopropylidenebutyl)-4-methyl-N-((2-nitrophenyl)ethynyl)benzenesulfonamide (3a)

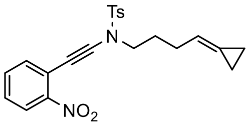

3a

Yield: 88% (639 mg), green oil, R_f = 0.3 (EtOAc/ petroleum ether = 1:8). **^{1}H NMR (500 MHz, $CDCl_3$)** δ 8.08 (d, J = 8.3 Hz, 1H), 7.87 (d, J = 8.3 Hz, 2H), 7.54 (d, J = 4.1 Hz, 2H), 7.38 – 7.33 (m, 3H), 5.75 (tp, J = 6.6, 2.3 Hz, 1H), 3.49 (t, J = 7.2 Hz, 2H), 2.44 (s, 3H), 2.26 (q, J = 7.0 Hz, 2H), 1.96 (p, J = 7.3 Hz, 2H), 1.02 (s, 4H). **^{13}C{^{1}H} NMR (126 MHz, $CDCl_3$)** δ 147.7, 145.1, 134.7, 133.3, 133.1, 130.1 (2C), 127.8, 127.2 (2C), 124.9, 122.7, 119.8, 116.5, 91.8, 69.3, 51.5, 28.6, 27.4, 21.8, 2.3, 2.1. **IR (KBr, cm^{-1}):** 2959, 2924, 1772, 1718, 1637, 1542, 1490, 1386, 1170, 746, 661, 587. **HRMS (ESI-TOF) / m/z: [M+Na]$^+$** calcd for $C_{22}H_{22}N_2O_4SNa$ 433.1192; found 433.1189.

Methyl 4-(((N-(4-cyclopropylidenebutyl)-4-methylphenyl)sulfonamido)ethynyl)-3- nitrobenzoate (3b)

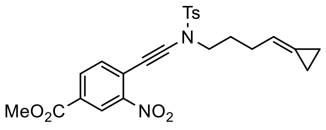

3b

Yield: 71% (216 mg), green oil, R_f = 0.3 (EtOAc/ petroleum ether = 1:8). **^{1}H NMR (500 MHz, $CDCl_3$)** δ 8.74 (s, 1H), 8.16 (d, J = 8.2 Hz, 1H), 7.86 (d, J = 8.2 Hz, 2H), 7.59 (d, J = 8.2 Hz, 1H), 7.36 (d, J = 8.1 Hz, 2H), 5.74 (tp ≈ t, J = 6.5 Hz, 1H), 3.96 (s, 3H), 3.52 (t, J = 7.2 Hz, 2H), 2.44 (s, 3H), 2.25 (q, J = 6.9 Hz, 2H), 1.95 (p, J = 7.3 Hz, 2H), 1.02 (s, 4H). **^{13}C{^{1}H} NMR (126 MHz, $CDCl_3$)** δ 165.1, 147.4, 145.6, 134.9, 133.7, 133.4, 130.4, 128.9, 128.0, 126.5, 124.4, 123.1, 116.6, 96.3, 70.9, 53.1, 51.8, 28.8, 27.7, 22.1, 2.6, 2.4. **IR (KBr, cm^{-1}):** 2953, 1766, 1624, 1529, 1442, 1293, 1167, 1109, 968, 812, 765, 664. **HRMS (ESI-TOF)/m/z:** $[M+Na]^+$ calcd for $C_{24}H_{24}N_2O_6SNa$ 491.1247; found 491.1248.

N-(4-cyclopropylidenebutyl)-4-methyl-N-((2-nitro-4-(trifluoromethyl)phenyl) ethynyl)benzenesulfonamide (3c)

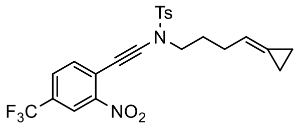

3c

Yield: 82% (287 mg), green oil, R_f = 0.3 (EtOAc/ petroleum ether = 1:8). **^{1}H NMR (500 MHz, $CDCl_3$)** δ 8.37 (s, 1H), 7.86 (d, J = 8.3 Hz, 2H), 7.77 (d, J = 8.1 Hz, 1H), 7.66 (d, J = 8.2 Hz, 1H), 7.37 (d, J = 8.1 Hz, 2H), 5.74 (tp, J = 6.6, 1.9 Hz, 1H), 3.52 (t, J = 7.2 Hz, 2H), 2.44 (s, 3H), 2.25 (q, J = 7.0 Hz, 2H), 1.95 (p, J = 7.3 Hz, 2H), 1.01 (s, 4H). **^{13}C{^{1}H} NMR (126 MHz, $CDCl_3$)** δ 147.0, 145.4, 134.6, 133.8, 130.2 (2C), 129.5 (q, J = 3.2 Hz), 128.9 (q, J = 34.5 Hz), 127.8 (2C), 123.7, 122.9 (q, J = 272.5 Hz), 122.9, 122.4 (q, J = 3.9 Hz), 116.3, 95.8, 70.0, 51.5, 28.6, 27.4, 21.8, 2.3, 2.1. **^{19}F NMR (471 MHz, $CDCl_3$)** δ -62.8. **IR (KBr, cm^{-1}):** 2957, 2921, 2858, 1635, 1454, 1326, 1245, 1139, 1088, 970, 853, 661, 587. **HRMS (ESI-TOF)/m/z:** $[M+Na]^+$ calcd for $C_{23}H_{21}F_3N_2O_4SNa$ 501.1066; found 501.1065.

N-(4-cyclopropylidenebutyl)-N-((4-fluoro-2-nitrophenyl)ethynyl)-4-methylbenzenesulfonamide. (3d)

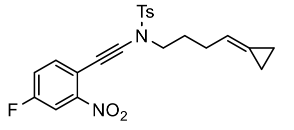

3d

Yield: 78% (284 mg), green oil, R_f = 0.3 (EtOAc/ petroleum ether = 1:5). **^{1}H NMR (500 MHz, $CDCl_3$)** δ 7.86 (d, *J* = 8.3 Hz, 2H), 7.81 (dd, *J* = 8.4, 2.6 Hz, 1H), 7.55 (dd, *J* = 8.7, 5.4 Hz, 1H), 7.37 (d, *J* = 8.1 Hz, 2H), 7.31 (ddd, *J* = 8.6, 7.4, 2.7 Hz, 1H), 5.74 (tp, *J* = 6.6, 2.1 Hz, 1H), 3.49 (t, *J* = 7.2 Hz, 2H), 2.44 (s, 3H), 2.26 (q, *J* = 7.0 Hz, 2H), 1.94 (p, *J* = 7.3 Hz, 2H), 1.02 (s, 4H). **^{13}C{^{1}H} NMR (126 MHz, $CDCl_3$)** δ 160.6 (d, J = 252.6 Hz), 148.5 (d, J = 8.2 Hz), 145.4, 135.2 (d, J = 7.8 Hz), 134.9, 130.3 (2C), 128.0 (2C), 123.0, 121.4 (d, J = 22.1 Hz), 116.7, 116.2 (d, J = 3.7 Hz), 112.6 (d, J = 26.9 Hz), 91.7, 68.5, 51.7, 28.8, 27.6, 22.0, 2.5, 2.3. **^{19}F NMR (471 MHz, $CDCl_3$)** δ -109.9. **IR (KBr, cm^{-1}):** 2928, 1777, 1645, 1575, 1454, 1366, 1269, 1020, 860, 812, 740, 666, 545. **HRMS (ESI-TOF)/m/z:** $[M+Na]^+$ calcd for $C_{22}H_{21}FN_2O_4SNa$ 451.1098; found 451.1094.

N-((4-chloro-2-nitrophenyl)ethynyl)-N-(4-cyclopropylidenebutyl)-4-methylbenzenesulfonamide (3e)

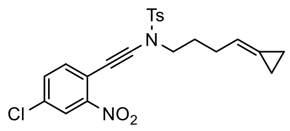

3e

Yield: 83% (216 mg), green oil, R_f = 0.3 (EtOAc/ petroleum ether = 1:12). **^{1}H NMR (500 MHz, $CDCl_3$)** δ 8.09 (d, J = 1.7 Hz, 1H), 7.85 (d, J = 8.2 Hz, 2H), 7.51 (dd, J = 8.4, 1.7 Hz, 1H), 7.48 (d, J = 8.4 Hz, 1H), 7.36 (d, J = 8.1 Hz, 2H), 5.73 (tp ≈ t, J = 6.5 Hz, 1H), 3.48 (t, J = 7.2 Hz, 2H), 2.44 (s, 3H), 2.25 (q, J = 6.9 Hz, 2H), 1.93 (p, J = 7.3 Hz, 2H), 1.01 (s, 4H).**^{13}C{^{1}H} NMR (126 MHz,**

$CDCl_3$) δ 148.1, 145.5, 134.9, 134.4, 133.7, 133.1, 130.4 (2C), 128.0 (2C), 125.3, 123.0, 118.6, 116.7, 93.2, 69.1, 51.7, 28.8, 27.6, 22.1, 2.6, 2.4. **IR (KBr, cm^{-1}):** 2984, 1765, 1633, 1525, 1371, 1242, 1098, 966, 814, 727, 661. **HRMS(ESI-TOF)/m/z: [M+Na]$^+$** calcd for $C_{22}H_{21}ClN_2O_4SNa$ 467.0803; found 467.0798.

N-((4-bromo-2-nitrophenyl)ethynyl)-N-(4-cyclopropylidenebutyl)-4-methylbenzenesulfonamide (3f)

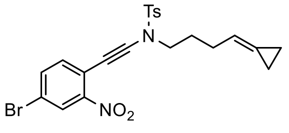

3f

Yield: 83% (284 mg), green oil, R_f = 0.3 (EtOAc/ petroleum ether = 1:5). **^{1}H NMR (500 MHz, $CDCl_3$)** δ 8.23 (d, J = 1.9 Hz, 1H), 7.84 (d, J = 8.3 Hz, 2H), 7.65 (dd, J = 8.4, 1.9 Hz, 1H), 7.40 (d, J = 8.4 Hz, 1H), 7.35 (d, J = 8.1 Hz, 2H), 5.73 (tp, *J* = 6.6, 2.3 Hz, 1H), 3.48 (t, J = 7.2 Hz, 2H), 2.44 (s, 3H), 2.24 (q, J = 7.0 Hz, 2H), 1.93 (p, J = 7.3 Hz, 2H), 1.01 (s, 4H). **$^{13}C\{^{1}H\}$ NMR (126 MHz, $CDCl_3$)** δ 147.9, 145.2, 136.2, 134.6, 134.2, 130.1(2C), 127.9 (2C), 127.7, 122.8, 120.1, 118.8, 116.4, 93.2, 69.0, 51.5, 28.6, 27.4, 21.8, 2.3, 2.1. **IR (KBr, cm^{-1}):** 2923, 2832, 1774, 1525, 1400, 1339, 1169, 1089, 962, 801, 665, 579. **HRMS (ESI-TOF)/m/z:** [M+Na]$^+$ calcd for $C_{22}H_{21}BrN_2O_4SNa$ 511.0298; found 511.0292.

N-(4-cyclopropylidenebutyl)-4-methyl-N-((4-methyl-2-nitrophenyl)ethynyl)benzenesulfonamide (3g)

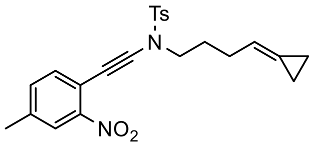

3g

Yield: 71% (216 mg), green oil, R_f = 0.3 (EtOAc/ petroleum ether = 1:8). **^{1}H NMR (500 MHz, $CDCl_3$)** δ 7.88 (s, 1H), 7.86 (d, *J* = 8.2 Hz, 2H), 7.43 (d, *J* = 7.9 Hz, 1H), 7.35 (d, *J* = 8.1 Hz, 3H), 5.74 (tp, *J* = 6.6, 2.3 Hz, 1H), 3.47 (t, *J* = 7.2 Hz, 2H), 2.43 (s, 3H), 2.42 (s, 3H), 2.25 (q, *J* = 7.0 Hz, 2H), 1.94 (p, *J* = 7.3 Hz, 2H), 1.01 (s, 4H).**$^{13}C\{^{1}H\}$ NMR (126 MHz, $CDCl_3$)** δ 148.0, 145.2, 138.4, 135.0, 134.3, 133.5, 130.3 (2C), 128.0 (2C), 125.4, 122.9, 117.0, 116.8, 90.7, 69.2, 51.7, 28.9, 27.6, 22.0, 21.5, 2.6, 2.3 **IR (KBr, cm^{-1}):** 2957, 1769, 1708, 1634, 1448, 1335, 1269, 1160, 1008, 856, 748, 586. **HRMS (ESI-TOF)/m/z:** [M+Na]$^+$ calcd for $C_{23}H_{24}N_2O_4SNa$ 447.1349; found 447.1345.

N-(4-cyclopropylidenebutyl)-N-((4-methoxy-2-nitrophenyl)ethynyl)-4-methylbenzenesulfonamide (3h)

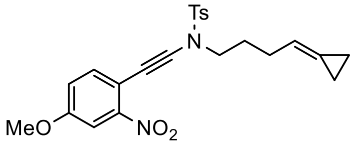

3h

Yield: 81% (276 mg), green oil, R_f = 0.3 (EtOAc/ petroleum ether = 1:9). **^{1}H NMR (500 MHz, $CDCl_3$)** δ 7.86 (d, J = 8.3 Hz, 2H), 7.56 (d, J = 2.6 Hz, 1H), 7.45 (d, J = 8.7 Hz, 1H), 7.36 (s, 2H), 7.11 (dd, J = 8.7, 2.7 Hz, 1H), 5.75 (tp, J = 6.7, 2.3 Hz, 1H), 3.87 (s, 3H), 3.46 (t, J = 7.2 Hz, 2H), 2.44 (s, 3H), 2.26 (q, J = 7.0 Hz, 2H), 1.94 (p, J = 7.3 Hz, 2H), 1.02 (s, 4H). **$^{13}C\{^{1}H\}$ NMR (126 MHz, $CDCl_3$)** δ 158.9, 149.2, 145.2, 134.9, 134.8, 130.2 (2C), 128.0 (2C), 122.8, 120.7, 116.8, 111.8, 109.3, 89.4, 68.4, 56.3, 51.7, 28.8, 27.6, 22.0, 2.5, 2.3. **IR (KBr, cm^{-1}):** 2941, 2845, 1767, 1636, 1529, 1452, 1238, 1031, 926, 810, 699, 561. **HRMS (ESI-TOF)/m/z:** [M+Na]$^+$ calcd for $C_{23}H_{24}N_2O_5SNa$ 463.1298; found 463.1296.

N-(4-cyclopropylidenebutyl)-4-methyl-N-((6-nitrobenzo[1][1,3]dioxol-5yl)ethynyl)benzenesul fonamide (3j')

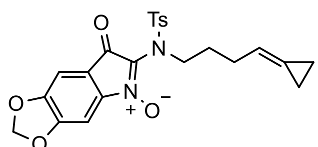

3j'

(After workin-up and purification, a nitrone intermediate was obtained rather than the desired ynamide)Yield: 81% (284 mg), red oil, R_f = 0.3 (EtOAc/ petroleum ether = 1:10). **^{1}H NMR (500 MHz, $CDCl_3$)** δ 7.91 (d, J = 8.2 Hz,

2H), 7.34 (d, J = 8.1 Hz, 2H), 7.11 (s, 1H), 7.02 (s, 1H), 6.16 (s, 2H), 5.56 (tp, J = 6.5, 2.1 Hz, 1H), 3.53 (t, J = 7.4 Hz, 2H), 2.45 (s, 3H), 2.07 (2.06 (q, J = 7.3 Hz, 2H), 1.53 (p, J = 7.4 Hz, 2H), 0.97 – 0.92 (m, 2H), 0.92 – 0.87 (m, 2H). **^{13}C{^{1}H} NMR (126 MHz, $CDCl_3$)** δ 182.2, 153.1, 150.6, 144.8, 142.1, 136.6, 131.8, 130.1 (2C), 128.5 (2C), 122.7, 116.7, 115.9, 103.6, 103.1, 98.0, 47.8, 28.7, 28.7, 22.0, 2.5, 2.2. **IR (KBr, cm^{-1}):** 2924, 2856, 1769, 1636, 1536, 1468, 1332, 1201, 1085, 962, 815, 582. **HRMS(ESI-TOF)/m/z:** $[M+Na]^+$ calcd for $C_{23}H_{22}N_2O_6SNa$ 477.1091; found 477.1086.

N-(4-cyclopropylidenebutyl)-N-((5-methoxy-2-nitrophenyl)ethynyl)-4-methylbenzenesulfonamide. (3k)

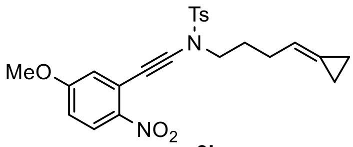

3k

Yield: 88% (213 mg), blue oil, R_f = 0.3 (EtOAc/ petroleum ether = 1:8). **^{1}H NMR (500 MHz, $CDCl_3$)** δ 8.11 (d, J = 9.3 Hz, 1H), 7.87 (d, J = 8.3 Hz, 2H), 7.36 (d, J = 8.1 Hz, 2H), 6.93 (d, J = 2.7 Hz, 1H), 6.82 (dd, J = 9.3, 2.8 Hz, 1H), 5.75 (tp, J = 6.4, 2.1 Hz, 1H), 3.89 (s, 3H), 3.50 (t, J = 7.2 Hz, 2H), 2.44 (s, 3H), 2.26 (q, J = 7.0 Hz, 2H), 1.96 (p, J = 7.3 Hz, 2H), 1.02 (s, 4H). **^{13}C{^{1}H} NMR (126 MHz, $CDCl_3$)** δ 163.4, 145.3, 141.2, 135.0, 130.3 (2C), 128.0 (2C), 127.6, 122.9, 122.3, 117.1, 116.8, 113.6, 92.1, 70.3, 56.4, 51.8, 28.8, 27.6, 22.0, 2.5, 2.3. **IR (KBr, cm^{-1}):** 2927, 2845 1658, 1515, 1460, 1363, 1241, 1087, 989, 815, 717, 574. **HRMS (ESI-TOF) /m/z:** $[M+Na]^+$ calcd for $C_{23}H_{24}N_2O_5SNa$ 463.1298; found 463.1293.

N-((5-chloro-2-nitrophenyl)ethynyl)-N-(4-cyclopropylidenebutyl)-4-methylbenzenesulfonamide (3l)

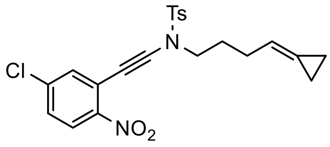

3l

Yield: 61% (210 mg), green oil, R_f = 0.3 (EtOAc/ petroleum ether = 1:8). **^{1}H NMR (500 MHz, $CDCl_3$)** δ 8.04 (d, J = 8.9 Hz, 1H), 7.86 (d, J = 7.7 Hz, 2H), 7.48 (s, 1H), 7.37 (d, J = 7.8 Hz, 2H), 7.29 (d, J = 8.9 Hz, 1H), 5.74 (tp ≈ t, J = 6.7 Hz, 1H), 3.50 (t, J = 7.1 Hz, 2H), 2.44 (s, 3H), 2.26 (q, J = 7.3 Hz, 2H), 1.94 (p, J = 7.1 Hz, 2H), 1.02 (s, 4H). **^{13}C{^{1}H} NMR (101 MHz, $CDCl_3$)** δ 145.8, 145.3, 139.8, 134.7, 132.5, 130.1 (2C), 127.8 (2C), 127.2, 126.3, 122.8, 121.6, 116.4, 93.6, 69.2, 51.5, 28.6, 27.4, 21.8, 2.3, 2.1. **IR (KBr, cm^{-1}):** 2928, 1764, 1611, 1521, 1369, 1242, 1121, 1094, 969, 869, 722, 581. **HRMS (ESI-TOF) /m/z:** $[M+Na]^+$ calcd for $C_{22}H_{21}ClN_2O_4SNa$ 467.0803; found 467.0798.

N-(4-cyclopropylidenebutyl)-N-((5-fluoro-2-nitrophenyl)ethynyl)-4-methylbenzenesulfonamide (3m)

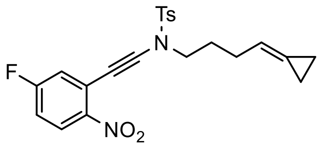

3m

Yield: 81% (276 mg), green oil, R_f = 0.3 (EtOAc/ petroleum ether = 1:9). **^{1}H NMR (500 MHz, $CDCl_3$)** δ 8.14 (dd, J = 7.4, 6.8 Hz, 1H), 7.86 (d, J = 7.8 Hz, 2H), 7.36 (d, J = 7.8 Hz, 2H), 7.18 (d, J = 8.6 Hz, 1H), 7.02 (t, J = 8.1 Hz, 1H), 5.74 (tp ≈ t, J = 6.5 Hz, 1H), 3.51 (t, J = 7.1 Hz, 2H), 2.44 (s, 3H), 2.25 (q, J = 7.4 Hz, 2H), 1.95 (p, J = 7.1 Hz, 2H), 1.02 (s, 4H). **^{13}C{^{1}H} NMR (101 MHz, $CDCl_3$)** δ 164.8 (d, J = 257.2 Hz), 145.3, 143.8, 134.7, 130.1 (2C), 127.8 (2C), 127.8 (d, J = 10.4 Hz), 122.8, 122.8 (d, J = 11.7 Hz), 119.4 (d, J = 25.1 Hz), 116.4, 114.6 (d, J = 23.7 Hz), 93.8, 69.4 (d, J = 2.2 Hz), 51.5, 28.6, 27.4, 21.8, 2.3, 2.1. **^{19}F NMR (376 MHz, $CDCl_3$)** δ -104.1. **IR (KBr, cm^{-1}):** 2985, 1764, 1629, 1526, 1371, 1243, 1078, 919, 818, 737. **HRMS(ESI-TOF)/m/z:** $[M+Na]^+$ calcd for $C_{22}H_{21}FN_2O_4SNa$ 451.1098; found 451.1095.

Methyl 3-(((N-(4-cyclopropylidenebutyl)-4-methylphenyl)sulfonamido)ethynyl)-4-nitrobenzoate (3n)

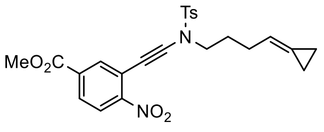

3n

Yield: 78% (275 mg), green oil, R_f = 0.3 (EtOAc/ petroleum ether = 1:8). **^{1}H NMR (500 MHz, $CDCl_3$)** δ 8.14 (d, J = 1.5 Hz, 1H), 8.10 (d, J = 8.6 Hz, 1H), 7.95 (dd, J = 8.6, 1.6 Hz, 1H), 7.87 (d, J = 8.2 Hz, 2H), 7.36 (d, J = 8.1 Hz, 2H), 5.74 (tp ≈ t, J = 6.5 Hz, 1H), 3.97 (s, 3H), 3.50 (t, J = 7.2 Hz, 2H), 2.44 (s, 3H), 2.25 (q, J = 6.9 Hz, 2H), 1.94 (p, J = 7.3 Hz, 2H), 1.01 (s, 4H). **^{13}C{^{1}H} NMR (126 MHz, $CDCl_3$)** δ 165.1, 150.2, 145.5, 134.9, 134.7, 134.5, 130.4 (2C), 128.1 (2C), 125.2, 123.0, 120.2, 116.7, 93.2, 69.1, 53.3, 51.8, 28.8, 27.7, 22.0, 2.5, 2.3. **IR (KBr, cm^{-1}):** 2955, 2869, 1773, 1631, 1441, 1334, 1272, 1088, 968, 813, 743, 663. **HRMS (ESI-TOF)/m/z:** $[M+H]^+$ calcd for $C_{24}H_{25}N_2O_6S$ 469.1428; found 469.1431.

N-(4-cyclopropylidenebutyl)-N-((2-fluoro-6-nitrophenyl)ethynyl)-4-methylbenzenesulfonamide (3o)

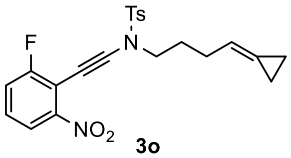

3o

Yield: 75% (240 mg), green oil, R_f = 0.3 (EtOAc/ petroleum ether = 1:10). **^{1}H NMR (500 MHz, $CDCl_3$)** δ 7.91 (d, J = 7.8 Hz, 3H), 7.39 – 7.27 (m, 4H), 5.75 (tp ≈ t, J = 6.6 Hz, 1H), 3.53 (t, J = 7.1 Hz, 2H), 2.26 (q, J = 6.6 Hz, 2H), 1.96 (p, J = 7.2 Hz, 2H), 1.02 (s, 4H). **^{13}C{^{1}H} NMR (101 MHz, $CDCl_3$)** δ 162.4 (d, *J* = 253.2 Hz), 148.6, 145.1, 134.6, 130.0 (2C), 127.9 (2C), 127.1 (d, *J* = 8.6 Hz), 122.8, 120.6 (d, *J* = 3.5 Hz), 120.1 (d, *J* = 21.8 Hz), 116.5, 109.8 (d, *J* = 20.4 Hz), 97.1 (d, *J* = 4.3 Hz), 62.9, 51.6, 28.6, 27.4, 21.8, 2.3, 2.1. **^{19}F NMR** (376 MHz, $CDCl_3$) δ -106.5. **IR (KBr, cm^{-1}):** 2940, 2870, 1777, 1716, 1618, 1486, 1360, 1255, 1121, 1085, 966, 812, 666. **HRMS (ESI-TOF)/m/z:** $[M+Na]^+$ calcd for $C_{22}H_{21}FN_2O_4SNa$ 451.1098; found 451.1093.

N-(4-cyclopropylidenebutyl)-4-methyl-N-((3-nitropyridin-2-yl)ethynyl)benzenesulfonamide (3p)

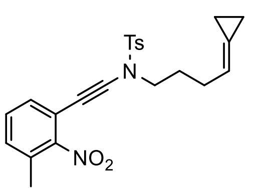

3p

Yield: 75% (120 mg), green oil, R_f = 0.5 (EtOAc/ petroleum ether = 1:10). **^{1}H NMR (400 MHz, $CDCl_3$)** δ 7.73 (d, *J* = 8.3 Hz, 2H), 7.28 (d, *J* = 8.1 Hz, 2H), 7.25 – 7.18 (m, 2H), 7.10 (d, *J* = 7.0 Hz, 1H), 5.65 (tp, *J* = 6.5, 1.9 Hz, 1H), 3.31 (t, *J* = 7.2 Hz, 2H), 2.36 (s, 3H), 2.24 (s, 3H), 2.14 (q, *J* = 7.0 Hz, 2H), 1.75 (p, *J* = 7.3 Hz, 2H), 0.94 (d, *J* = 1.4 Hz, 4H). **^{13}C NMR (101 MHz, CDCl3)** δ 151.9, 145.0, 134.5, 130.4, 130.3, 130.2, 130.1, 130.0 (2C), 127.7 (2C), 122.7, 116.8, 116.5, 88.6, 65.5, 51.3, 28.5, 27.3, 21.8, 17.7, 2.3, 2.1. **IR (KBr, cm^{-1}):** 2925, 1742, 1707, 1646, 1546, 1531, 1462, 1340, 1161, 1087, 754, 585, 545. **HRMS (ESI-TOF)/m/z:** $[M+H]^+$ calcd for $C_{23}H_{25}N_2O_4S$ 425.1530; found 425.1529.

N-(4-cyclopropylidenebutyl)-4-methyl-N-((3-nitropyridin-2-yl)ethynyl)benzenesulfonamide (3r)

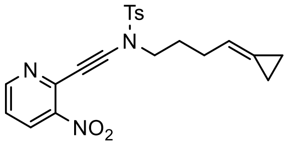

3r

Yield: 18% (40 mg), green oil, R_f = 0.3 (EtOAc/ petroleum ether = 1:5). **^{1}H NMR (500 MHz, $CDCl_3$)** δ 8.75 (d, J = 4.6 Hz, 1H), 8.37 (d, J = 8.4 Hz, 1H), 7.93 (d, J = 8.2 Hz, 2H), 7.36 (d, J = 8.1 Hz, 2H), 7.31 (dd, J = 8.4, 4.7 Hz, 1H), 5.73 (tp ≈ t, J = 6.4 Hz, 1H), 3.55 (t, J = 7.3 Hz, 2H), 2.43 (s, 3H), 2.25 (q, J = 6.9 Hz, 2H), 1.96 (p, J = 7.3 Hz, 2H), 1.01 (s, 4H). **^{13}C{^{1}H} NMR (126 MHz, $CDCl_3$)** δ 154.1, 145.5, 144.6, 138.7, 135.0, 132.9, 130.4 (2C), 128.2 (2C), 123.1, 121.4, 116.7, 93.5, 71.9, 52.0, 28.9, 27.9, 22.1, 2.6, 2.4. **IR (KBr, cm^{-1}):** 2924, 1635, 1523, 1379, 1268, 1165, 1087, 969, 755, 659, 586. **HRMS (ESI-TOF) /m/z:** $[M+H]^+$ calcd for $C_{21}H_{22}N_3O_4S$ 412.1326; found 412.1324.

N-(4-cyclopropylidenehexyl)-4-methyl-N-((2-nitrophenyl)ethynyl)benzenesulfonamide. (3s)

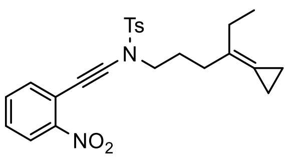

3s

Yield: 83% (40 mg), green oil, R_f = 0.3 (EtOAc/ petroleum ether = 1:8). **^{1}H NMR (500 MHz, $CDCl_3$)** δ 8.08 (d, J = 8.3 Hz, 1H), 7.87 (d, J = 8.0 Hz, 2H), 7.54 (d, J = 4.0 Hz, 2H), 7.36 (d, J = 7.7 Hz, 3H), 3.46 (t, J = 7.3 Hz, 2H), 2.44 (s, 3H), 2.21 (t, J = 7.2 Hz, 2H), 2.14 (q, J = 7.1 Hz, 2H), 1.99 (p, J = 7.3 Hz, 2H), 1.04 (t, J = 7.5 Hz, 3H), 1.00 (d, J = 5.9 Hz, 2H), 0.97 (d, J = 6.1 Hz, 2H). **^{13}C{^{1}H} NMR (126 MHz, $CDCl_3$)** δ 147.7, 145.1, 134.7, 133.3, 133.1, 130.1 (2C), 127.9, 127.8 (2C), 127.2, 124.9, 119.8, 115.1, 91.8, 69.3, 51.9, 31.7, 28.2, 25.9, 21.8, 12.6, 2.0, 1.7. **IR (KBr, cm^{-1}):** 2930, 1768, 1714, 1599, 1521, 1454, 1367, 1168, 1088, 971, 812, 741, 668. **HRMS(ESI-TOF)/m/z:** $[M+Na]^+$ calcd for $C_{24}H_{26}N_2O_4SNa$ 461.1505; found 461.1498.

N-(4-cyclopropylidene-4-phenylbutyl)-4-methyl-N-((2-nitrophenyl)ethynyl)benzenesulfonamide.(3t)

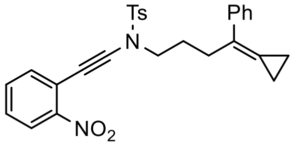

3t

Yield: 66% (30 mg), green oil, R_f = 0.3 (EtOAc/ petroleum ether = 1:8).**^{1}H NMR (500 MHz, $CDCl_3$)** δ 8.08 (d, *J* = 8.1 Hz, 1H), 7.81 (d, *J* = 8.2 Hz, 2H), 7.56 (d, *J* = 7.6 Hz, 2H), 7.52 (t, *J* = 7.7 Hz, 1H), 7.47 (d, *J* = 6.8 Hz, 1H), 7.35 (t, *J* = 7.2 Hz, 1H), 7.32 – 7.27 (m, 4H), 7.20 (t, *J* = 7.3 Hz, 1H), 3.50 (t, *J* = 7.2 Hz, 2H), 2.74 (t, *J* = 7.4 Hz, 2H), 2.41 (s, 3H), 2.03 (p, *J* = 7.3 Hz, 2H), 1.39 (dd ≈ t, *J* = 7.0, 2H), 1.18 (dd ≈ t, *J* = 7.0 Hz, 2H). **^{13}C{^{1}H} NMR (126 MHz, $CDCl_3$)** δ 147.9, 145.3, 140.0, 134.9, 133.6, 133.4, 130.3 (2C), 128.6 (2C), 128.0 (2C), 127.4, 126.9, 126.4 (2C), 126.0, 125.2, 122.3, 120.0, 91.9, 69.8, 51.8, 30.9, 26.5, 22.1, 5.2, 1.8. **IR (KBr, cm^{-1}):** 2925, 1741, 1707, 1692, 1646, 1462, 1160, 815, 756, 704, 592, 467. **HRMS (ESI-TOF)/m/z:** $[M+H]^+$ calcd for $C_{28}H_{27}N_2O_4S$ 487.1686; found 487.1692.

N-(3-cyclopropylidenepropyl)-4-methyl-N-((2-nitrophenyl)ethynyl)benzenesulfonamide (3u')

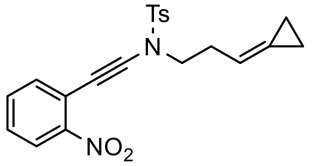

3u'

Yield: 78% (230 mg), green oil, R_f = 0.3 (EtOAc/ petroleum ether = 1:8). **^{1}H NMR** (500 MHz, $CDCl_3$) δ 8.08 (d, J = 8.3 Hz, 1H), 7.87 (d, J = 8.3 Hz, 2H), 7.54 (d, J = 4.1 Hz, 2H), 7.36 (d, J = 8.2 Hz, 2H), 7.35 (t, J = 8.5 Hz, 1H), 5.71 (tp, J = 6.6, 1.9 Hz, 1H), 3.61 (t, J = 7.4 Hz, 2H), 2.65 (q, J = 7.0 Hz, 2H), 2.44 (s, 3H), 1.01 (d, J = 1.2 Hz, 4H). **^{13}C{^{1}H} NMR (126 MHz, $CDCl_3$)** δ 147.8, 145.0, 134.9, 133.3, 133.1, 130.0 (2C), 127.8 (2C), 127.1, 125.3, 124.9, 119.8, 113.3, 91.8, 69.6, 51.5, 30.4, 21.8, 2.6, 2.1. **IR (KBr, cm^{-1}):** 2987, 2830, 1625, 1491, 1399, 1162, 1006, 793, 704, 667, 591. **HRMS(ESI-TOF)/m/z:** $[M+H]^+$ calcd for $C_{21}H_{21}N_2O_4S$ 397.1217; found 397.1216.

N-(3-cyclopropylidenebutyl)-4-methyl-N-((2-nitrophenyl)ethynyl)benzenesulfonamide (3v)

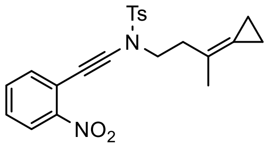

3v

Yield: 72% (60 mg), green oil, R_f = 0.3 (EtOAc/ petroleum ether = 1:8). **^{1}H NMR (500 MHz, $CDCl_3$)** δ 8.08 (d, J = 8.3 Hz, 1H), 7.86 (d, J = 8.2 Hz, 2H), 7.58 – 7.51 (m, 2H), 7.38 – 7.32 (m, 3H), 3.68 (t, J = 7.5 Hz, 2H), 2.61 (t, J = 7.5 Hz, 2H), 2.43 (s, 3H), 1.83 (s, 3H), 1.02 (t, J = 6.9 Hz, 2H), 0.92 (t, J = 7.1 Hz, 2H). **^{13}C{^{1}H} NMR (126 MHz, $CDCl_3$)** δ 147.7, 145.0, 134.9, 133.2, 133.1, 130.0 (2C), 127.8 (2C), 127.1, 124.9, 120.0, 119.8, 118.8, 91.8, 69.5, 50.2, 34.9, 21.8, 20.7, 3.1, 1.9. **IR (KBr, cm^{-1}):** 2970, 1779, 1639, 1523, 1342, 1164, 1023, 853, 739, 666. **HRMS(ESI-TOF)/m/z:** $[M+H]^+$ calcd for $C_{22}H_{23}N_2O_4S$ 411.1373; found 411.1367.

N-(4-cyclopropylidenebutyl)-N-((4-(dimethylamino)-2-nitrophenyl)ethynyl)-4-methylbenzenesulfonamide (3w)

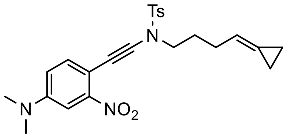

3w

Yield: 84% (87 mg), red oil, R_f = 0.3 (EtOAc/ petroleum ether = 1:10). **^{1}H NMR (500 MHz, $CDCl_3$)** δ 7.85 (d, *J* = 8.2 Hz, 2H), 7.35 (d, *J* = 9.4 Hz, 3H), 7.23 (d, *J* = 2.5 Hz, 1H), 6.81 (dd, *J* = 8.8, 2.6 Hz, 1H), 5.74 (tp ≈ t, *J* = 6.4 Hz, 1H), 3.42 (t, *J* = 7.2 Hz, 2H), 3.02 (s, 6H), 2.43 (s, 3H), 2.24 (q, *J* = 6.9 Hz, 2H), 1.91 (p, *J* = 7.3 Hz, 2H), 1.01 (s, 4H). **^{13}C{^{1}H} NMR (126 MHz, $CDCl_3$)** δ 150.0, 149.4, 144.7, 134.8, 134.8, 129.9 (2C), 127.8 (2C), 122.5, 116.7, 116.2, 106.8, 105.1, 86.9, 68.0, 51.5, 40.3 (2C), 28.6, 27.3, 21.7, 2.3, 2.1. **IR (KBr, cm^{-1}):** 2927, 1633, 1539, 1372, 1271, 1091, 967, 809, 747, 663. **HRMS(ESI-TOF)/m/z:** $[M+H]^+$ calcd for $C_{24}H_{28}N_3O_4S$ 454.1795; found 454.1796.

N-(4-cyclopropylidenebutyl)-N-((2-nitrophenyl)ethynyl)methanesulfonamide (3x)

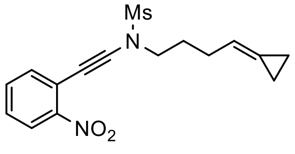

3x

Yield: 85% (180 mg), green oil, R_f = 0.3 (EtOAc/ petroleum ether = 1:8). **^{1}H NMR (500 MHz, $CDCl_3$)** δ 8.09 (d, J = 8.3 Hz, 1H), 7.57 – 7.52 (m, 2H), 7.37 (ddd, J = 8.6, 5.7, 3.1 Hz, 1H), 5.78 (tp ≈ t, J = 6.4 Hz, 1H), 3.62 (t, J = 7.3 Hz, 2H), 3.21 (s, 3H), 2.32 (q, J = 6.9 Hz, 2H), 2.03 (p, J = 7.3 Hz, 2H), 1.03 (s, 4H). **^{13}C{^{1}H} NMR (126 MHz, $CDCl_3$)** δ 147.9, 133.3, 133.2, 127.5, 124.9, 122.9, 119.3, 116.3, 90.7, 69.6, 51.5, 39.0, 28.6, 27.8, 2.3, 2.1. **IR (KBr, cm^{-1}):** 3048, 2941, 1715, 1579, 1445, 1271, 1148, 1012, 904, 799, 663. **HRMS(ESI-TOF)/m/z:** $[M+H]^+$ calcd for $C_{16}H_{19}N_2O_4S$ 335.1060; found 335.1057.

Procedure for preparation of 3y

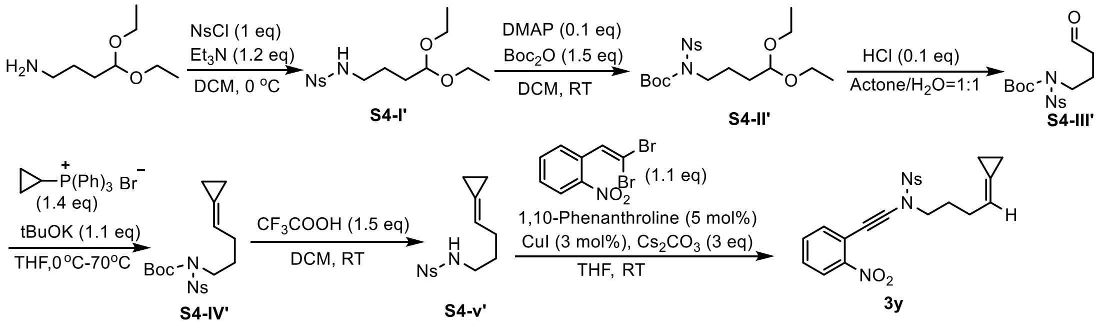

The synthesis of **3y** (total yield: 23%) are the same as the above operations. The substrate was synthesized according to the procedures of **S4** above, using NsCl instead of TsCl. (Note: The Ns protecting group is unstable under basic conditions while heating, the operation of removing Boc protection is under acidic conditions)

N-(4-cyclopropylidenebutyl)-4-nitro-N-((2-nitrophenyl)ethynyl)benzenesulfonamide(3y)

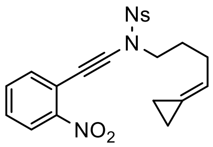

3y

Yield: 58% (153 mg), green oil, R_f = 0.4 (EtOAc/ petroleum ether = 1:4). **^{1}H NMR (500 MHz, $CDCl_3$)** δ 8.35 (dd, *J* = 5.7, 3.6 Hz, 1H), 8.09 (d, *J* = 8.4 Hz, 1H), 7.80 (dd, *J* = 5.9, 3.3 Hz, 2H), 7.75 – 7.71 (m, 1H), 7.58 – 7.51 (m, 2H), 7.38 (t, *J* = 6.8 Hz, 1H), 5.79 (tp ≈ t, *J* = 6.5 Hz, 1H), 3.76 (t, *J* = 7.2 Hz, 2H), 2.34 (q, *J* = 7.0 Hz, 2H), 2.06 (p, *J* = 7.2 Hz, 2H), 1.04 (s, 4H). **^{13}C{^{1}H} NMR (126 MHz, $CDCl_3$)** δ 148.1, 147.8, 135.0, 133.4, 133.2, 132.5, 132.2, 130.8, 127.6, 124.9, 124.6, 122.9, 119.2, 116.4, 89.8, 70.2, 52.5, 28.6, 27.9, 2.3, 2.1 **IR (KBr, cm^{-1}):** 3160, 1771, 1578, 1344, 1279, 1124, 782, 750, 507. **HRMS (ESI – TOF)/m/z:** $[M+Na]^+$ calcd for $C_{21}H_{19}N_3O_6SNa$ 463.0934; found 463.0930.

Procedure for preparation of 3z

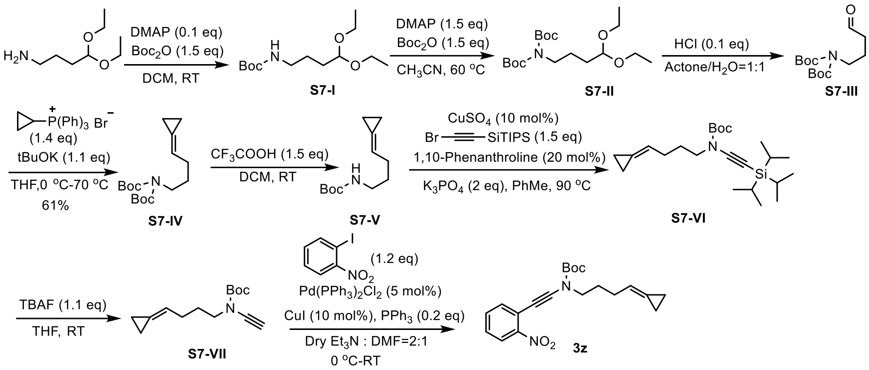

To a solution of 4,4-diethoxybutan-1-amine (3.4 mL, 20 mmol) and Boc_2O (6.9 mL, 30 mmol) in DCM (50 mL) was added DMAP (0.24 g, 2 mmol) slowly at r.t. and stirred for 1 h. After completion, the reaction was quenched with water (100 mL) and extracted with DCM (3*50 mL). The organic phase was collected, dried with Na_2SO_4 and concentrated under reduced pressure. The yellow oil (**S7-I**) was used in the next step without further purification.

To a solution of crude **S7-I** in 50 mL CH_3CN was added Boc_2O (30 mmol) and DMAP (30 mmol) at r.t. The reaction mixture was stirred at 60 °C for 10 h. After completion, the reaction was quenched with water (50 mL) and extracted with EtOAc (2*50 mL). The organic phase was collected, dried with Na_2SO_4 and concentrated under reduced pressure. The residue (**S7-II**) was used in the next step without further purification.

To a 100 mL round-bottom flask with a magnetic bar were added **S7-II**, the mixed solvent (acetone/ water = 1:1, 60 mL) and 8 M HCl solution (0.1 eq, 0.25 mL). The mixture was stirred at r.t. for 12 h. After the reaction was finished (the reaction can be monitored by $KMnO_4$ on TLC), the reaction mixture was washed with $NaHCO_3$ solution (50 mL) and water (50 mL), extracted with EtOAc (50 mL*2), dried with Na_2SO_4 and concentrated in vacuo. The crude product was purified by column chromatography (silica gel, EtOAc/petroleum ether = 1:4) to afford **S7-III** as yellow oil (Three-step total yield: 79.4%).

A suspension of Wittig reagent (1.4 eq, 22.4 mmol) in dry THF (120 mL) was prepared under N_2 atmosphere. Potassium tert-butoxide (1.1 eq, 17.6 mL, 1 M in THF) was then added slowly to the suspension using a syringe pump over 5 minutes at 0 °C. The mixture was warmed to r.t. for 1 h. **S7-III** (1 eq, 16 mmol) in THF (10 mL) solution was added slowly to the suspension using a syringe pump over 15 minutes. After stirring for 1 h at r.t., the reaction was heated to 70 °C in oil bath. After completion, the solution was filtrated through a pad of silica gel. The organic phases were concentrated in vacuo and purified over silica gel (EtOAc/petroleum ether = 1:30, 65%).

The isolated **S7-IV** (1 eq, 10.4 mmol) was dissolved in 20 mL DCM, following by adding CF_3COOH (1.5 eq, 15.6 mmol). The mixture was stirred at rt for 5 h. After completion, the reaction was quenched with $NaHCO_3$ solution (50 mL) and extracted with DCM (50 mL*2). The organic phase was collected, dried with Na_2SO_4 and concentrated under reduced pressure. The residue was purified by column chromatography (silica gel, EtOAc/petroleum ether = 1:6) to afford **S7-V** as yellow oil (99%).

Add K_3PO_4 (2.0 eq, 4 mmol) (Note: K_3PO_4 must be dried), $CuSO_4$ (10 mol%, 0.2 mmol), 1,10-phenanthroline (20 mol%, 0.4 mmol) and 1-bromo-2-triisopropyisilylacetylene (1.25 eq, 2.5 mmol) to a stirred solution of amide (1.0 eq, 2 mmol, 1.0 M in toluene) under nitrogen atmosphere and then stirred at 90 ^{o}C for 96 h. Monitor the reaction by TLC analysis. After completion, cool the reaction mixture to room temperature, diluted with EtOAc (20 mL) and filtrated through celite. The organic phases were concentrated in vacuo and purified the crude product by silica gel chromatography on a silica gel column with petroleumether(PE) and ethyl acetate (EA) as eluents (EtOAc/petroleum ether = 1:30) to obtain the product **S7-VI** (57%).

The **S7-VI** (1 eq, 1.14 mmol) was dissolved in THF (0.5 M), and tetrabutylammonium fluoride (1.1 eq, 1.25 mmol) was added and stirred at r.t. for 30 minutes. The mixture was diluted with H_2O (10 mL), extracted with EtOAc (10 mL*3). The extract was dried over Na_2SO_4 and evaporated under reduced pressure. The yellow oil (**S7-VII**) was used in the next step without further purification.

The sonogashira coupling conditions was according to a reported procedure[6].

To a solution of alkyne **S7-VII** (1 eq, 0.39 mmol), o-iodonitrobenzene (1.3 eq, 0.5 mmol) in Et_3N:DMF (2:1, 6 mL) were added PPh_3 (0.2 eq, 0.08 mmol), $Pd(PPh_3)_2Cl_2$ (0.05 eq, 0.02 mmol). The reaction mixture was degassed with N_2 for 10 min and CuI (0.1 eq, 0.04 mmol) was added and degassed again and the contents stirred at room temperature for 10 h. The reaction mixture was partitioned between ethyl acetate and water and the aqueous layer was extracted with ethyl acetate (2*25 mL). Combined ethyl acetate layer was washed with brine (10 mL), dried over Na_2SO_4, concentrated and the residue obtained was purified by silica gel column chromatography to afford the **3z**.

tert-butyl (4-cyclopropylidenebutyl)((2-nitrophenyl)ethynyl)carbamate (3z)

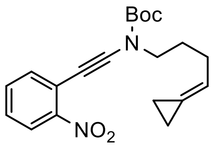

3z

Yield: 43% (60 mg), green liquid, R_f = 0.3 (EtOAc/ petroleum ether = 1:20). **^{1}H NMR (500 MHz, $CDCl_3$)** δ 8.08 (d, J = 8.3 Hz, 1H), 7.55 – 7.46 (m, 2H), 7.32 (t, J = 7.9 Hz, 1H), 5.81 (tp ≈ t, J = 6.3 Hz, 1H), 3.60 (t, J = 7.3 Hz, 2H), 2.29 (q, J = 6.8 Hz, 2H), 1.96 (p, J = 7.3 Hz, 2H), 1.57 (s, 9H), 1.03 (s, 4H). **^{13}C{^{1}H} NMR (126 MHz, $CDCl_3$)**) δ 153.7, 147.7, 135.0, 133.0, 126.7, 124.9, 122.3, 120.5, 117.0, 83.3, 82.7, 49.3, 28.9, 28.2, 28.1, 27.5, 2.3, 2.1 1. **IR (KBr, cm^{-1}):** 3158, 2920, 2852, 1737, 1618, 1580, 1436, 1270, 780, 753. **HRMS (ESI – TOF)/m/z:** $[M+H]^+$ calcd for $C_{20}H_{25}N_2O_4$ 357.1809; found 357.1803.

N-(4-cyclobutylidenebutyl)-4-methyl-N-((2-nitrophenyl)ethynyl)benzenesulfonamide (3aa)

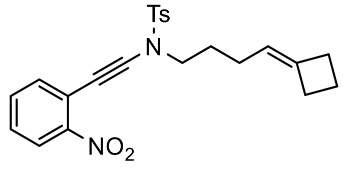

3aa

Yield: 88% (225 mg), green oil, R_f = 0.3 (EtOAc/ petroleum ether = 1:5). **^{1}H NMR (500 MHz, $CDCl_3$)** δ 8.08 (d, *J* = 8.3 Hz, 1H), 7.87 (d, *J* = 8.1 Hz, 2H), 7.54 (d, *J* = 4.2 Hz, 2H), 7.38 – 7.31 (m, 3H), 5.01 (t, *J* = 7.4 Hz, 1H), 3.46 (t, *J* = 7.2 Hz, 2H), 2.62 (t, *J* = 7.8 Hz, 2H), 2.58 (t, *J* = 8.0 Hz, 2H), 2.43 (s, 3H), 1.98 – 1.85 (m, 4H), 1.80 (p, *J* = 7.2 Hz, 2H). **^{13}C{^{1}H} NMR (126 MHz, $CDCl_3$)** δ 147.7, 145.1, 141.6, 134.7, 133.3, 133.1, 130.1 (2C), 127.8 (2C), 127.2, 124.9, 119.8, 118.5, 91.7, 69.4, 51.4, 31.0, 29.4, 27.8, 24.8, 21.8, 17.1. **IR (KBr, cm^{-1}):** 3058, 1732, 1597, 1474, 1338, 1162, 1023, 808, 666, 546. **HRMS (ESI-TOF) /m/z:** $[M+H]^+$ calcd for $C_{23}H_{25}N_2O_4S$ 425.1530; found 425.1527.

Procedure for preparation of 3ab and 3ac

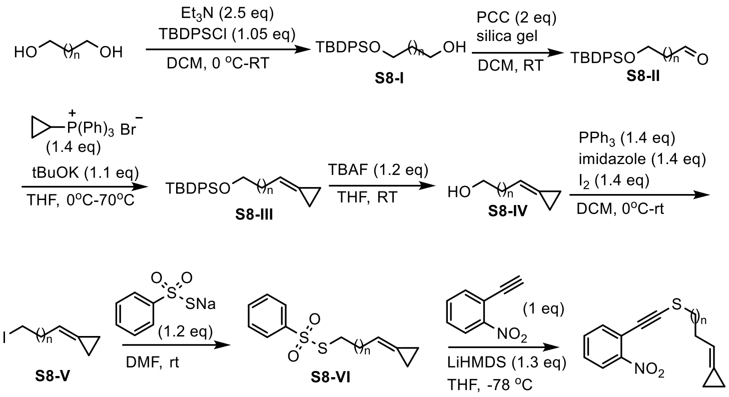

The alcohol (40 mmol) was added to a solution of Et_3N (100 mmol) in DCM (80 mL). TBDPSCl (41 mmol) was added dropwise at 0 °C. The mixture was stirred for 10 h at room temperature. Then, the reaction mixture was quenched with NH_4Cl (aq.) (60 mL), extracted with DCM (3*50 mL). The combined organic layers were washed with brine (50 mL) and dried over Na_2SO_4, filtered and concentrated. The yellow oil (**S8-I**) was used in the next step without further purification.

To a solution of **S8-I** (1 eq, 40 mmol) in DCM (100 mL) was added the mixture of PCC (2 eq, 80 mmol) and silica gel (weight ratio = 1:1) at r.t. and stirred for 3 h. After completion, the solution was filtrated through a pad of silica gel. The organic phase was collected, dried with Na_2SO_4 and concentrated under reduced pressure. The organic layer was concentrated and the residue was purified by chromatography on silica gel (PE: EtOAc = 30:1) to afford **S8-II** (Two-step total yield: 84%).

A suspension of Wittig reagent (1.4 eq, 47 mmol) in dry THF (120 mL) was prepared under N_2 atmosphere. Potassium tert-butoxide (1.1 eq, 37 mL, 1.0 M in THF) solution was then added slowly to the suspension using a syringe pump over 15 minutes at 0 °C. The mixture was warmed to r.t. for 1 h. **S8-II** (1 eq, 33.6 mmol) in THF (10 mL) solution was added slowly to the suspension using a syringe pump over 15 minutes. After stirring for 1 h at r.t., the reaction was heated to 70 °C in oil bath. After completion, the solution was filtrated through a pad of silica gel. The organic phases were concentrated in vacuo and purified over silica gel (EtOAc/petroleum ether = 1:30, n=1, Yield:17%, n=2, Yield: 52%).

The **S8-III** (1 eq, 5 mmol) was dissolved in THF (0.5 M), and tetrabutylammonium fluoride (1.1 eq, 5.5 mmol) was added and stirred for 30 minutes. The mixture was diluted with H_2O (10 mL), extracted with EtOAc (10 mL*3). The extract was dried over Na_2SO_4 and evaporated under reduced pressure. The yellow oil (**S8-IV**) was used in the next step without further purification.

Dissolve triphenylphosphine (1.4 eq, 7mmol) and imidazole (1.4 eq, 7 mmol) in DCM (20 mL). Cool the solution to 0 °C. Add iodine (1.4 eq, 7 mmol) slowly and stir the reaction for 15 min. Add **S8-IV** (1 eq, 5 mmol) slowly at 0 °C. Warm the mixture to room temperature for 3 h. Add petroleum ether (20 mL) to the reaction solution. Filter the suspension through celite and concentrate the solution in vacuo at RT (Note: This iodine has a low b. p.) and purified over silica gel (petroleum ether, Two-step total yield: 82%).

To a solution of benzenesulfonic acid sodium salt (1.2 eq, 4.8 mmol) in DMF (10 mL) was slowly added the solution of **S8-V** (4 mmol) at r.t. and stirred for 3 h. Upon completion, water was added (50 mL)

and extracted with EtOAc (3*50 mL). The combined organic phases were washed with brine (50 mL). The organic phase was dried over Na_2SO_4, filtered and concentrated in vacuo. The resulting crude mixture was purified via silica gel column chromatography (silica gel, EtOAc/petroleum ether = 1:10, 81%).

LiHMDS (1 M in THF, 1.3 eq, 1.4 mL) was slowly added to a stirred solution of alkyne (1 eq, 1.1 mmol) in dry THF (0.2 M) over 10 min at -78 °C. After 15 min, the lithium acetaminide was treated with the corresponding benzene thiosulfonate(1.1 eq, 1.2 mmol) in dry THF (0.1M) which was added drop-wise over 15 min and the solution was then stirred for a further 15 min at -78 °C. The mixture was allowed to warm to room temperature and stirred for 1 h. The reaction mixture was quenched with saturated NH_4Cl (aq) (10 mL) and then extracted with THF (2*10 mL). The combined organic layers were dried over Na_2SO_4, filtered and concentrated under reduced pressure to give a residue which was purified by silica gel column chromatography to afford the desired alkynyl thioethers (**3ab**: 75%, **3ac**: 82%).

(4-cyclopropylidenebutyl)((2-nitrophenyl)ethynyl)sulfane (3ab)

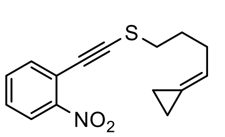

3ab

Yield: 75% (232 mg), green oil, R_f = 0.4 (EtOAc/ petroleum ether = 1:30). **^{1}H NMR (500 MHz, CDCl$_3$)** δ 8.07 (d, *J* = 8.3 Hz, 1H), 7.53 (d, *J* = 4.2 Hz, 2H), 7.40 – 7.33 (m, 1H), 5.78 (tp ≈ t, *J* = 6.1 Hz, 1H), 2.90 (t, *J* = 7.2 Hz, 2H), 2.37 (q, *J* = 6.6 Hz, 2H), 2.04 (p, *J* = 7.1 Hz, 2H), 1.04 (s, 4H). **^{13}C{^{1}H} NMR (126 MHz, CDCl$_3$)** δ 148.4, 133.6, 133.1, 127.5, 124.9, 122.8, 119.6, 116.7, 91.3, 90.2, 35.8, 30.4, 28.9, 2.4, 2.1. **IR (KBr, cm^{-1}):** 3286, 2928, 1728, 1635, 1476, 1254, 999, 850, 744, 664, 578. **HRMS (ESI – TOF)/m/z:** $[M+H]^+$ calcd for $C_{15}H_{16}NO_2S$ 274.0896; found 274.0892.

(3-cyclopropylidenepropyl)((2-nitrophenyl)ethynyl)sulfane (3ac)

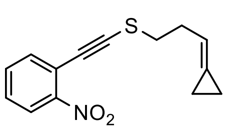

3ac

Yield: 82% (233 mg), green oil, R_f = 0.3 (EtOAc/ petroleum ether = 1:30). **^{1}H NMR (500 MHz, CDCl$_3$)** δ 7.99 (d, *J* = 8.4 Hz, 1H), 7.48 – 7.43 (m, 2H), 7.32 – 7.26 (m, 1H), 5.77 (tp ≈ t, *J* = 6.4 Hz, 1H), 2.94 (t, *J* = 7.3 Hz, 2H), 2.67 (q, *J* = 6.9 Hz, 2H), 1.00 (s, 4H). **^{13}C{^{1}H} NMR (126 MHz, CDCl$_3$)** δ 148.3, 133.5, 133.0, 127.5, 124.8, 124.4, 119.5, 115.1, 91.2, 90.4, 35.9, 31.7, 2.4, 2.2. **IR (KBr, cm^{-1}):** 3047, 2920, 1697, 1611, 1340, 1257, 961, 840, 743, 616. **HRMS (ESI – TOF)/m/z:** $[M+H]^+$ calcd for $C_{14}H_{14}NO_2S$ 260.0740; found 260.0735.

Typical procedure for preparation of 3ad

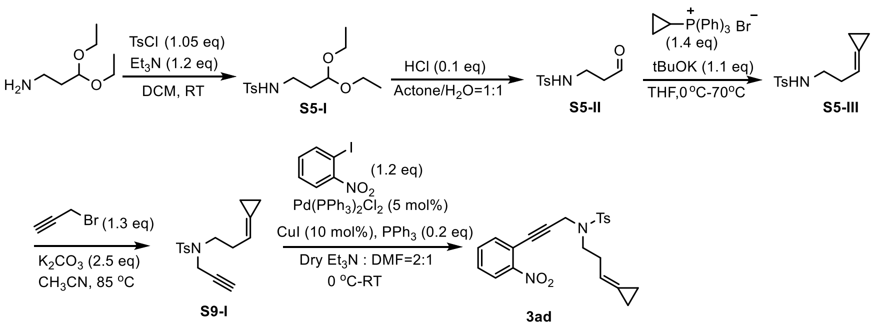

The synthesis of the three **S5-I** to **S5-III** are the same as the above operations.

To the solution of **S5-III** (0.5 g, 2 mmol) in CH_3CN (10 mL) was added K_2CO_3 (0.7 g, 5 mmol) and 3-bromoprop-1-yne (0.31 g, 2.6 mmol), the mixture was stirred for 12 h at 85 ℃. Upon completion, water was added (20 mL) and extracted with EtOAc (3 * 20 mL). The organic layer was washed with brine (10

mL), dried over Na_2SO_4, concentrated and the residue was purified by chromatography on silica gel (PE: EtOAc = 5:1) to afford 0.46 g **S9-I** in 81% yield.

To a solution of alkyne **S9-I** (1 eq, 1.62 mmol), o-iodonitrobenzene (1.3 eq, 2.1 mmol) in Et_3N:DMF (2:1, 18 mL), PPh_3 (0.2 eq, 0.32 mmol) was added. To this solution, $Pd(PPh_3)_2Cl_2$ (0.05 eq, 0.08 mmol) was added and the reaction mixture was degassed with N_2 for 10 min and CuI (0.1 eq, 0.16 mmol) was added and degassed again and the contents stirred at room temperature for 10 h. The reaction mixture was partitioned between ethyl acetate and water and the aqueous layer was extracted with ethyl acetate (2*25 mL). Combined ethyl acetate layer was washed with brine (50 mL), dried over Na_2SO_4, concentrated and the residue obtained was purified by silica gel column chromatography to afford the **3ad** (63%).

N-(3-cyclopropylidenepropyl)-4-methyl-N-(3-(2-nitrophenyl)prop-2-yn-1-yl)benzenesulfonamide (3ad)

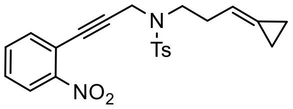

3ad

Yield: 63% (418 mg), green liquid, R_f = 0.3 (EtOAc/ petroleum ether = 1:6). **^{1}H NMR (500 MHz, CDCl$_3$, *major*)** δ 8.01 – 7.95 (m, 1H), 7.73 (d, J = 8.2 Hz, 2H), 7.51 (td, J = 7.6, 1.1 Hz, 1H), 7.47 – 7.40 (m, 1H), 7.26 (d, J = 6.7 Hz, 1H), 7.17 (d, J = 8.1 Hz, 2H), 5.79 (tp, J = 6.6, 1.9 Hz, 1H), 4.42 (s, 2H), 3.48 – 3.41 (m, 2H), 2.53 (q, J = 7.0 Hz, 2H), 2.25 (s, 3H), 1.05 (s, 4H). **^{13}C NMR (126 MHz, CDCl$_3$, major)** δ 143.5, 136.0, 134.83, 134.81, 132.8, 129.5 (2C), 129.0, 127.9 (2C), 124.7, 124.4, 117.8, 114.2, 90.5, 80.9, 46.3, 37.4, 30.4, 21.5, 2.7, 2.0.. **IR (KBr, cm^{-1}):** 3128, 2924, 1638, 1568, 1401, 1161, 1091, 964, 815, 670. **HRMS (ESI – TOF)/m/z:** $[M + H]^+$ calcd for $C_{22}H_{23}N_2O_4S$ 411.1373; found 411.1366.

Typical procedure for preparation of 3ae

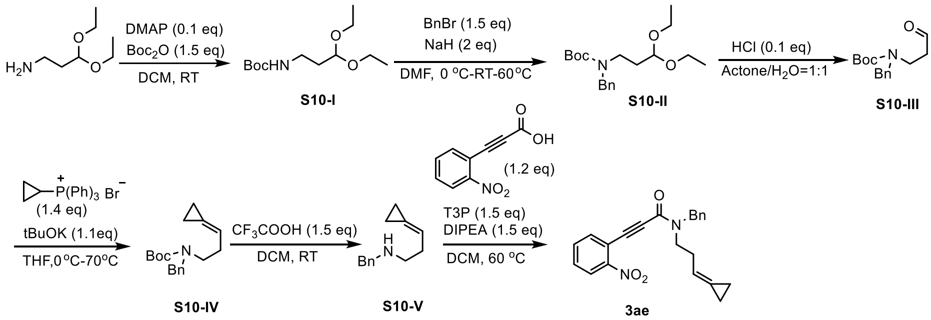

To a solution of 3,3-diethoxypropan-1-amine (3.4 mL, 20 mmol) and Boc_2O (6.9 mL, 30 mmol) in DCM (50 mL) was added DMAP (0.24 g, 2 mmol) slowly at r.t. and stirred for 1 h. After completion, the reaction was quenched with water (100 mL) and extracted with DCM (3 x 50 mL). The organic phase was collected, dried with Na_2SO_4 and concentrated under reduced pressure. The yellow oil (**S10-I**) was used in the next step without further purification.

To a suspension of sodium hydride (60%, 1.60 g, 2 eq) in anhydrous DMF (40 mL) at 0 °C was added **S10-I** (1 eq, 20 mmol). After 30 mins, BnBr (5.13 g, 30 mmol) was added to the reaction mixture at 0 ^{o}C. The reaction mixture was stirred at room temperature for 1h, then was stirred at 60 ^{o}C additional 10 h. The reaction mixture was quenched with *satd*. NH_4Cl(aq) (50 mL) and then extracted with EtOAc (3 × 50 mL). And the combined organic fractions were washed with brine (2 × 50 mL). The solution was dried

over Na_2SO_4, filtered and concentrated under reduced pressure. The yellow oil (**S10-II**) was used in the next step without further purification.

To a 100 mL round-bottom flask with a magnetic bar were added **S10-II** (20 mmol), the mixed solvent (acetone/ water = 1:1, 50 mL) and 8M HCl solution (0.1 eq, 0.25 mL). The mixture was stirred at r.t. for 12 h. After the reaction was finished (the reaction can be monitored by $KMnO_4$ on TLC), the reaction mixture was washed with $NaHCO_3$ solution (40 mL), extracted with EtOAc (40 mL*3), dried with Na_2SO_4 and concentrated in vacuo. The crude product was purified by column chromatography (silica gel, EtOAc/petroleum ether = 1:4) to afford **S10-III** as yellow oil (Three-step total yield: 72%).

A suspension of Wittig reagent (1.4 eq, 19.6 mmol) in dry THF (70 mL) was prepared under N_2 atmosphere. Potassium tert-butoxide (1.1 eq, 1M in THF, 15.4 mL) solution was then added slowly to the suspension using a syringe pump over 15 minutes at 0 ^{o}C. The mixture was warmed to r.t. for 1 h. **S10-III** (1 eq, 14 mmol) in THF (10 mL) solution was added slowly to the suspension using a syringe pump over 15 minutes. After stirring for 1 h at r.t., the reaction was heated to 70 ^{o}C in oil bath. After completion, the solution was filtrated through a pad of silica gel. The organic phases were concentrated in vacuo and purified over silica gel (EtOAc/petroleum ether = 1:30, 62%).

The isolated **S10-IV** (1 eq, 8.4 mmol) was dissolved in 20 mL DCM, following by adding CF_3COOH (1.5 eq, 12.6 mmol). The mixture was stirred at rt for 5 h. After completion, the reaction was quenched with $NaHCO_3$ solution (50 mL) and extracted with DCM (20 mL*3). The organic phase was collected, dried with Na_2SO_4 and concentrated under reduced pressure. The residue was purified by column chromatography (silica gel, EtOAc/petroleum ether = 1:6) to afford **S10-V** as yellow oil (94%).

The amide condensation conditions are according to a reported procedure[7].

A round-bottom flask was charged with a carboxylic acid (1.2 eq, 0.9 mmol) which was dissolved in DCM (0.1 M). Subsequently, DIPEA (1.5 eq, 1.13 mmol), T3P (n-propanephosphonic acid anhydride; 50% in EtOAc, 1.5 eq, 1.13 mmol) and the **S10-V** (1 eq, 0.75 mmol) were added. The resulting mixture was heated to 60 °C and allowed to stir for 16 hours. Upon completion, water was added (18 mL) and extracted with EtOAc (3x10 mL). The combined organic phases were washed with brine (10 mL). The organic phase was dried over Na_2SO_4, filtered and concentrated in vacuo. The resulting crude mixture was purified via silica gel column chromatography (silica gel, EtOAc/petroleum ether = 1:10, 70%).

N-benzyl-N-(3-cyclopropylidenepropyl)-3-(2-nitrophenyl)propiolamide(3ae)

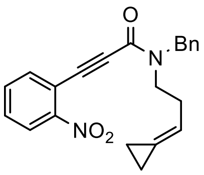

3ae

Yield: 70% (189.3 mg), yellow oil, R_f = 0.3 (EtOAc/ petroleum ether = 1:6). **^{1}H NMR (500 MHz, $CDCl_3$)** δ 8.05 (t, *J* = 8.5 Hz, 1H), 7.75 and 7.71 (d, *J* = 7.7 Hz, 1H), 7.65 - 7.51 (m, 1H), 7.54 – 7.47 (m, 1H), 7.32 – 7.20 (m, 5H), 5.72 and 5.65 (tp ≈ t, *J* = 6.6 Hz, 1H), 4.86 and 4.62 (s, 2H), 3.67 and 3.41 (t, *J* = 7.2 Hz, 2H), 2.46 and 2.36 (q, *J* = 7.0 Hz, 2H), 1.01 – 0.86 (m, 4H). **^{13}C{^{1}H} NMR (126 MHz, $CDCl_3$)** δ154.3, 154.1, 149.5, 149.4, 136.6, 136.4, 136.1, 136.1, 133.4, 130.6, 128.9, 128.8, 128.4, 128.0, 127.8, 127.8, 125.1, 125.0, 124.9, 124.3, 116.5, 116.5, 114.4, 114.0, 88.5, 88.4, 84.4, 84.3, 52.8, 47.7, 47.6, 44.1, 31.1, 29.6, 2.7, 2.6, 2.0, 1.8. **IR (KBr, cm^{-1}):** 3032, 2927, 1529, 1426, 1272, 1029, 1001, 933, 745, 666. **HRMS (ESI – TOF)/m/z:** $[M+H]^+$ calcd for $C_{22}H_{21}N_2O_3$ 361.1547; found 361.1548.

Typical procedure for preparation of 4

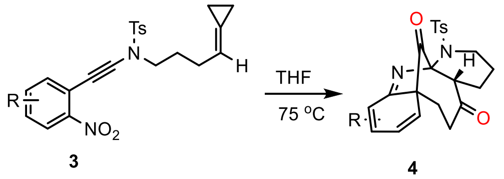

A dried 25 mL Schlenk tube was flushed with N_2 three times. The solution of **3** (1 eq) in the THF (0.03 M) was added to the tube under nitrogen atmosphere. The resulting mixture was put in a 75 °C oil bath and stirred for 12 h. After cooled to room temperature, the mixture was transfered to a 25 mL round bottom flask and evaporated under reduced pressure. The crude product was purified by column chromatography (silica gel, petroleum ether/EtOAc = 2:1) to give **4**.

1-tosyl-2,3,4,4a,6,7-hexahydro-7a,12a-methanobenzo[2]pyrido[3,2-g]azocine-5,13(1H)-dione (4a)

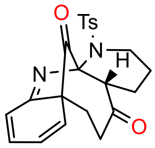

4a

Yield: 74% (60 mg), pink solid, m.p. 199.0 – 201.6 °C. R_f = 0.3 (EtOAc/ petroleum ether = 1:2). **^{1}H NMR (500 MHz, $CDCl_3$)** δ 7.69 (d, J = 8.2 Hz, 2H), 7.28 (d, J = 4.9 Hz, 2H), 6.73 (dd, J = 9.7, 5.6 Hz, 1H), 6.54 (d, J = 9.2 Hz, 1H), 6.51 (d, J = 9.7 Hz, 1H), 6.29 (dd, J = 9.2, 5.6 Hz, 1H), 3.30 (d, J = 8.3 Hz, 2H), 2.93 (t, J = 8.3, 7.9 Hz, 1H), 2.59 (ddd, J = 12.9, 9.2, 6.1 Hz, 1H), 2.49 – 2.43 (m, 1H), 2.41 (s, 3H), 2.00 – 1.86 (m, 4H), 1.81 – 1.74 (m, 1H), 1.70 – 1.58 (m, 1H). **^{13}C{^{1}H} NMR (126 MHz, $CDCl_3$)** δ 210.5, 205.6, 179.6, 144.1, 135.9, 135.4, 132.9, 129.4 (2C), 128.5 (2C), 122.7, 121.4, 84.7, 59.2, 58.0, 44.3, 40.3, 30.9, 23.4, 22.0, 21.6. **IR (KBr, cm^{-1}):** 2959, 2924, 1772, 1718, 1637, 1542, 1490, 1386, 1170, 746, 661, 587. **HRMS (ESI-TOF)/m/z:** [M+H]$^+$ calcd for $C_{22}H_{23}N_2O_4S$ 411.1373; found 411.1372.

Methyl-5,13-dioxo-1-tosyl-1,2,3,4,4a,5,6,7-octahydro-7a,12a-methanobenzo[2]pyrido[3,2-g]azocine-10-carboxylate (4b)

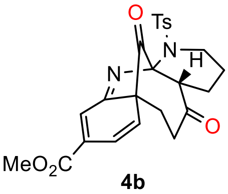

4b

Yield: 81% (60 mg), pink solid, m.p. 101.5 - 102.7 °C. R_f = 0.3 (EtOAc/ petroleum ether = 1:2). **^{1}H NMR (500 MHz, $CDCl_3$)** δ 7.70 (d, J = 7.6 Hz, 2H), 7.33 (s, 1H), 7.28 (d, J = 7.9 Hz, 2H), 6.75 (d, J = 9.5 Hz, 1H), 6.57 (d, J = 9.5 Hz, 1H), 3.87 (s, 3H), 3.37 (t, J = 12.0 Hz, 1H), 3.26 (d, J = 10.7 Hz, 1H), 2.99 – 2.91 (m, 1H), 2.65 – 2.55 (m, 1H), 2.46 – 2.42 (m, 1H), 2.41 (s, 3H), 2.01 – 1.85 (m, 4H), 1.74 – 1.59 (m, 2H). **^{13}C{^{1}H} NMR (101 MHz, $CDCl_3$)** δ 209.6, 204.9, 179.0, 165.1, 144.2, 136.7, 135.4, 132.7, 129.5 (2C), 128.3 (2C), 125.9, 121.1, 85.2, 58.9, 58.2, 52.7, 44.2, 40.2, 30.5, 23.3, 21.9, 21.6. **IR (KBr, cm^{-1}):** 2925, 1770, 1634, 1399, 1251, 1086, 972, 852, 728, 617. **HRMS(ESI-TOF)/m/z:** [M+Na]$^+$ calcd for $C_{24}H_{24}N_2O_6SNa$ 491.1247; found 491.1246.

1-tosyl-10-(trifluoromethyl)-2,3,4,4a,6,7-hexahydro-7a,12a-methanobenzo[2]pyrido[3,2-g]azocine-5,13(1H)-dione (4c)

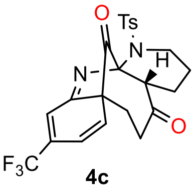

4c

Yield: 68% (47 mg), pink solid, m.p. 108.9 - 109.1 °C. R_f = 0.3 (EtOAc/ petroleum ether = 1:4). **^{1}H NMR (500 MHz, $CDCl_3$)** δ 7.68 (d, J = 8.1 Hz, 2H), 7.28 (d, J = 8.1 Hz, 2H), 6.89 (s, 1H), 6.68 (d, J = 9.5 Hz, 1H), 6.35 (d, J = 9.6 Hz, 1H), 3.35 (t, J = 11.5 Hz, 1H), 3.29 (d, J = 9.8 Hz, 1H), 2.97 (t, J = 8.0 Hz, 1H), 2.64 (ddd, J = 12.6, 10.0, 6.5 Hz, 1H), 2.47 – 2.42 (m, 1H), 2.42 (s, 3H), 2.02 – 1.93 (m, 2H), 1.93 – 1.85 (m, 2H), 1.76 (ddd, J = 13.9, 9.9, 6.0 Hz, 1H), 1.64 – 1.55 (m, 1H). **^{13}C{^{1}H} NMR (126 MHz, $CDCl_3$)** δ 208.8, 204.6, 177.2, 144.4, 136.54 (q, J = 34.2 Hz), 135.2, 134.7, 129.5 (2C), 128.3 (2C),

121.15(q, J = 273.1Hz), 120.59 (q, J = 5.5 Hz), 118.2(q, J = 2.3Hz), 84.9, 58.9, 58.2, 44.3, 40.1, 30.1, 23.2, 21.7, 21.6. **^{19}F NMR (471 MHz, $CDCl_3$)** δ -68.4. **IR (KBr, cm^{-1}):** 2932, 2867, 1773, 1634, 1445, 1280, 1140, 976, 863, 663, 582. **HRMS(ESI-TOF)/m/z:** $[M+H]^+$ calcd for $C_{23}H_{22}F_3N_2O_4S$ 479.1247; found 479.1241.

10-fluoro-1-tosyl-2,3,4,4a,6,7-hexahydro-7a,12a-methanobenzo[2]pyrido[3,2-g]azocine-5,13(1H)-dione. (4d)

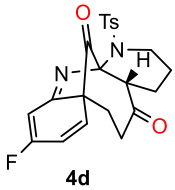

4d

Yield: 73% (48 mg), pink solid, m.p. 130.1 – 131.9 °C. R_f = 0.3 (EtOAc/ petroleum ether = 1:2). **^{1}H NMR (500 MHz, $CDCl_3$)** δ 7.71 (d, J = 8.1 Hz, 2H), 7.29 (d, J = 8.1 Hz, 2H), 6.70 (dd, J = 9.8, 6.5 Hz, 1H), 6.24 (t, J = 9.1 Hz, 1H), 6.15 (d, J = 10.0 Hz, 1H), 3.32 (t, J = 11.5 Hz, 1H), 3.26 (d, J = 9.5 Hz, 1H), 2.90 (dd, J = 11.2, 4.8 Hz, 1H), 2.60 (ddd, J = 14.6, 9.0, 5.9 Hz, 1H), 2.48 (dt, J = 12.8, 6.2 Hz, 1H), 2.42 (s, 3H), 2.01 – 1.94 (m, 1H), 1.94 – 1.88 (m, 2H), 1.82 (ddd, J = 14.0, 8.9, 5.7 Hz, 1H), 1.66 – 1.58 (m, 2H). **^{13}C{^{1}H} NMR (126 MHz, $CDCl_3$)** δ 209.1, 205.4, 178.1 (d, *J* = 15.2 Hz), 166.4 (d, *J* = 269.8 Hz), 144.3, 135.9 (d, *J* = 11.7 Hz), 135.3, 129.5 (2C), 128.4 (2C), 119.6 (d, *J* = 35.0 Hz), 101.3 (d, *J* = 20.6 Hz), 85.1, 59.2, 57.9, 44.2, 40.1, 31.5, 23.4, 22.1, 21.6. **^{19}F NMR (471 MHz, $CDCl_3$)** δ -94.1a. **IR (KBr, cm^{-1}):** 2956, 2923, 1780, 1644, 1491, 1330, 1154, 1010, 920, 785, 658. **HRMS(ESI-TOF)/m/z:** $[M+Na]^+$ calcd for $C_{22}H_{21}FN_2O_4SNa$ 451.1098; found 451.1099.

10-chloro-1-tosyl-2,3,4,4a,6,7-hexahydro-7a,12a-methanobenzo[2]pyrido[3,2-g]azocine-5,13(1H)-dione. (4e)

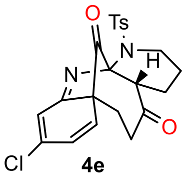

4e

Yield: 77% (60 mg), pink solid, m.p. 129.6 - 130.8 °C. R_f = 0.3 (EtOAc/ petroleum ether = 1:3). **^{1}H NMR (500 MHz, $CDCl_3$)** δ 7.70 (d, *J* = 7.8 Hz, 2H), 7.29 (s, 2H), 6.62 (s, 1H), 6.57 (d, *J* = 9.6 Hz, 1H), 6.25 (d, *J* = 9.6 Hz, 1H), 3.33 (t, *J* = 12.3 Hz, 1H), 3.26 (d, *J* = 9.2 Hz, 1H), 2.91 (t, *J* = 7.3 Hz, 1H), 2.65 – 2.53 (m, 1H), 2.50 – 2.43 (m, 1H), 2.42 (s, 3H), 2.02 – 1.87 (m, 4H), 1.84 – 1.75 (m, 1H), 1.63 – 1.52 (m, 1H). **^{13}C{^{1}H} NMR (101 MHz, $CDCl_3$)** δ 209.1, 205.1, 177.4, 144.2, 142.6, 135.4, 133.6, 129.5 (2C), 128.4 (2C), 126.1, 119.2, 85.0, 58.4, 58.0, 44.2, 40.1, 31.2, 23.4, 22.0, 21.6. **IR (KBr, cm^{-1}):** 2925, 2859, 1764, 1654, 1397, 1239, 1094, 788, 675, 522. H**RMS(ESI-TOF)/m/z:** $[M+Na]^+$ calcd for $C_{22}H_{21}ClN_2O_4SNa$ 467.0803; found 467.0802.

10-bromo-1-tosyl-2,3,4,4a,6,7-hexahydro-7a,12a-methanobenzo[2]pyrido[3,2-g]azocine-5,13(1H)-dione (4f)

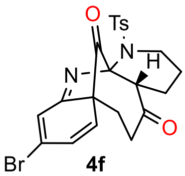

4f

Yield: 78% (53 mg), pink solid, m.p. 120.4 - 121.1 °C. R_f = 0.3 (EtOAc/ petroleum ether = 1:3). **^{1}H NMR (500 MHz, $CDCl_3$)** δ 7.70 (d, J = 8.2 Hz, 2H), 7.29 (d, J = 8.1 Hz, 2H), 6.87 (s, 1H), 6.49 (d, J = 9.6 Hz, 1H), 6.38 (dd, J = 9.6, 1.2 Hz, 1H), 3.36 – 3.25 (m, 2H), 2.95 – 2.88 (m, 1H), 2.60 (ddd, J = 12.9, 9.3, 6.1 Hz, 1H), 2.49 – 2.43 (m, 1H), 2.42 (s, 3H), 2.01 – 1.88 (m, 4H), 1.85 – 1.79 (m, 1H), 1.66 – 1.56 (m, 1H). **^{13}C{^{1}H} NMR (126 MHz, $CDCl_3$)** δ 209.2, 205.2, 177.6, 144.3, 135.3, 133.2, 132.3, 129.5 (2C), 128.4 (2C), 128.0, 123.0, 84.9, 58.2, 57.9, 44.2, 40.1, 31.0, 23.4, 21.9, 21.6. **IR (KBr, cm^{-1}):** 2925, 1768, 1630, 1398, 1240, 1157, 1089, 852, 788, 658, 614. **HRMS(ESI-TOF)/m/z:** $[M+Na]^+$ calcd for $C_{22}H_{21}BrN_2O_4SNa$ 511.0298; found 511.0295.

10-methyl-1-tosyl-2,3,4,4a,6,7-hexahydro-7a,12a-methanobenzo²pyrido[3,2-g]azocine-5,13(1H)-dione. (4g)

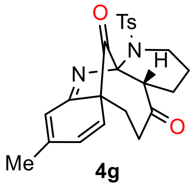

4g

Yield: 74% (50 mg), pink solid, m.p. 107.2 – 108.8 °C. R_f = 0.3 (EtOAc/ petroleum ether = 1:2). **^{1}H NMR (500 MHz, $CDCl_3$)** δ 7.70 (d, *J* = 8.0 Hz, 2H), 7.27 (d, *J* = 5.3 Hz, 2H), 6.52 (d, *J* = 9.3 Hz, 1H), 6.30 (s, 1H), 6.15 (d, *J* = 9.3 Hz, 1H), 3.36 – 3.23 (m, 2H), 2.93 – 2.87 (m, 1H), 2.57 (ddd, *J* = 14.4, 8.9, 6.2 Hz, 1H), 2.49 – 2.43 (m, 1H), 2.41 (s, 3H), 2.10 (s, 3H), 1.97 – 1.87 (m, 4H), 1.75 (ddd, *J* = 14.1, 8.9, 5.9 Hz, 1H), 1.66 – 1.58 (m, 1H). **^{13}C{^{1}H} NMR (126 MHz, $CDCl_3$)** δ 210.7, 205.9, 179.8, 147.0, 144.0, 135.5, 132.1, 129.4 (2C), 128.4 (2C), 126.9, 117.9, 85.0, 58.5, 58.0, 44.2, 40.3, 31.5, 23.5, 22.8, 22.1, 21.6. **IR (KBr, cm^{-1}):** 2954, 2861, 1770, 1585, 1391, 1237, 1089, 968, 813, 735, 659. **HRMS (ESI-TOF) /m/z:** $[M+H]^+$ calcd for $C_{23}H_{25}N_2O_4S$ 425.153; found 425.1528.

10-methoxy-1-tosyl-2,3,4,4a,6,7-hexahydro-7a,12a-methanobenzo²pyrido[3,2-g]azocine-5,13(1H)-dione. (4h)

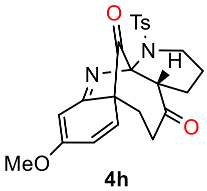

4h

Yield: 81% (46 mg), pink solid, m.p. 197.8 - 198.4 °C. R_f = 0.3 (EtOAc/ petroleum ether = 1:2). **^{1}H NMR (500 MHz, $CDCl_3$)** δ 7.73 (d, *J* = 8.1 Hz, 2H), 7.27 (d, *J* = 8.0 Hz, 2H), 6.61 (d, *J* = 9.7 Hz, 1H), 6.13 (dd, *J* = 9.7, 1.6 Hz, 1H), 5.71 (s, 1H), 3.81 (s, 3H), 3.35 (t, *J* = 11.7 Hz, 1H), 3.26 (d, *J* = 8.6 Hz, 1H), 2.87 (t, *J* = 8.0 Hz, 1H), 2.57 (ddd, *J* = 14.1, 8.6, 5.8 Hz, 2H), 2.50 (dt, *J* = 12.9, 6.4 Hz, 1H), 2.41 (s, 3H), 2.01 – 1.87 (m, 4H), 1.79 (ddd, *J* = 13.9, 8.6, 5.8 Hz, 1H). **^{13}C{^{1}H} NMR (126 MHz, $CDCl_3$)** δ 210.3, 206.2, 179.5, 165.1, 144.0, 135.6, 133.9, 129.4 (2C), 128.4 (2C), 123.2, 92.8, 85.3, 58.6, 57.8, 55.8, 44.1, 40.3, 32.6, 23.6, 22.3, 21.6. **IR (KBr, cm^{-1}):** 2956, 1768, 1448, 1330, 1231, 1089, 866, 726, 657, 586. **HRMS(ESI-TOF)/m/z:** $[M+Na]^+$ calcd for $C_{23}H_{24}N_2O_5SNa$ 463.1298; found 463.1296.

Typical procedure for preparation of 4i (Note: **3i** will deteriorate during separation)

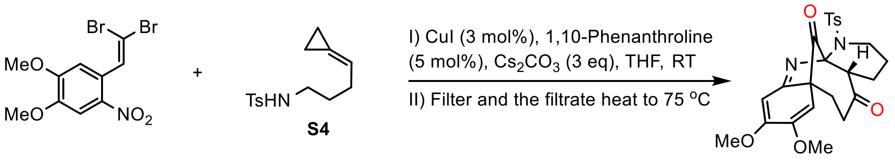

A mixture of 1-(2,2-dibromovinyl)-4,5-dimethoxy-2-nitrobenzene (1.1 eq), **S4** (1 eq), CuI (3 mol%), 1,10-phenantroline (5 mol%), caesium carbonate (3 eq) in THF (0.1 M) were stirred at r.t. for 12 h under N_2 atmosphere in a 25 mL Schlenk tube. After completion (12 h, monitored by TLC), the mixture was filtrated through celite, the resulting filtrate was added to a dried 25 mL Schlenk tube under nitrogen atmosphere and put in a 75 °C oil bath stirring for 12 h. After cooled to room temperature, the mixture was transfered to a 25 mL round bottom flask and evaporated under reduced pressure. The residue was purified by column chromatography (silica gel, petroleum ether/EtOAc = 2:1) to give **4i**.

9,10-dimethoxy-1-tosyl-2,3,4,4a,6,7-hexahydro-7a,12a-methanobenzo²pyrido[3,2-g]azocine-5,13(1H)-dione.(4i)

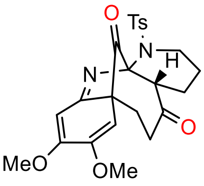

4i

Yield: 68% (40 mg), yellow solid, m.p. 115.2 – 116.0 °C. R_f = 0.3 (EtOAc/ petroleum ether = 1:1). **^{1}H NMR (500 MHz, $CDCl_3$)** δ 7.73 (d, *J* = 8.1 Hz, 2H), 7.27 (d, *J* = 8.2 Hz, 2H), 5.75 (s, 1H), 5.55 (s, 1H), 3.87 (s, 3H), 3.69 (s, 3H), 3.32 (t, *J* = 11.6 Hz, 1H), 3.26 (d, *J* = 8.1 Hz, 1H), 2.87 (t, *J* = 8.1 Hz, 1H), 2.55 (ddd, *J* = 14.0, 8.5, 5.7 Hz, 1H), 2.47 (dt, *J* = 12.9, 6.4 Hz, 1H), 2.41 (s, 3H), 2.00 – 1.86 (m, 4H), 1.81 (ddd, *J* = 13.9, 8.5, 5.8 Hz, 1H), 1.65 – 1.59 (m, 1H). ^{13}C NMR (101 MHz, $CDCl_3$) **^{13}C{^{1}H} NMR (126 MHz, $CDCl_3$)** δ 211.3, 206.4, 178.4, 160.8, 148.6, 144.0, 135.5, 129.4 (2C), 128.5 (2C), 101.5, 94.0, 85.5, 57.9, 57.4, 56.5, 55.7, 44.2, 40.4, 34.1, 23.6, 22.3, 21.6. **IR (KBr, cm^{-1}):** 2955, 1769, 1635, 1452, 1331, 1003, 962, 820, 657. **HRMS(ESI-TOF)/m/z:** [M+H]$^+$ calcd for $C_{24}H_{27}N_2O_6S$ 471.1584; found 471.1584.

1-tosyl-2,3,4,4a,6,7-hexahydro-7a,13a-methano[1,3]dioxolo[4',5':4,5]benzo[1,2-b]pyrido[3,2-g]azocine-5,14(1H)-dione. (4j)

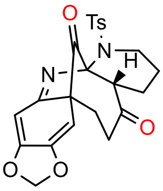

4j

Yield: 76% (53 mg), purple solid, m.p. 197.1 - 198.1 °C. R_f = 0.3 (EtOAc/ petroleum ether = 2:3). **^{1}H NMR (500 MHz, $CDCl_3$)** δ 7.74 (d, *J* = 8.1 Hz, 2H), 7.29 (d, *J* = 8.2 Hz, 2H), 5.93 (s, 1H), 5.81 (s, 1H), 5.79 (s, 1H), 5.77 (s, 1H), 3.34 (t, *J* = 11.6 Hz, 1H), 3.24 (d, *J* = 10.1 Hz, 1H), 2.84 (dd, *J* = 11.1, 5.2 Hz, 1H), 2.58 – 2.46 (m, 2H), 2.42 (s, 3H), 1.98 (dt, *J* = 13.4, 6.6 Hz, 1H), 1.93 – 1.89 (m, 2H), 1.88 – 1.79 (m, 2H), 1.67 – 1.56 (m, 1H).. **^{13}C{^{1}H} NMR (126 MHz, $CDCl_3$)** δ 210.5, 206.4, 178.3, 157.4, 145.7, 144.1, 135.5, 129.4 (2C), 128.4 (2C), 101.5, 99.4, 92.8, 86.1, 58.8, 57.7, 44.1, 40.1, 34.5, 23.7, 22.5, 21.6. **IR (KBr, cm^{-1}):** 3044, 2955, 2924, 1707, 1528, 1382, 1203, 1058, 960, 819, 738, 586. **HRMS(ESI-TOF)/m/z:** [M+Na]$^+$ calcd for $C_{23}H_{22}N_2O_6SNa$ 477.1091; found 477.1086.

9-methoxy-1-tosyl-2,3,4,4a,6,7-hexahydro-7a,12a-methanobenzo²pyrido[3,2-g]azocine-5,13(1H)-dione. (4k)

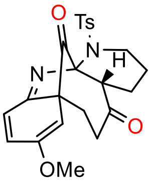

4k

Yield: 63% (42 mg), purple solid, m.p. 109.5 - 110.4 °C. R_f = 0.3 (EtOAc/ petroleum ether = 1:2). **^{1}H NMR (500 MHz, $CDCl_3$)** δ 7.70 (d, *J* = 8.2 Hz, 2H), 7.29 (d, *J* = 8.0 Hz, 2H), 6.59 (dd, *J* = 10.0, 2.4 Hz, 1H), 6.50 (d, *J* = 10.0 Hz, 1H), 5.43 (d, *J* = 2.2 Hz, 1H), 3.63 (s, 3H), 3.32 – 3.24 (m, 2H), 2.97 – 2.90 (m, 1H), 2.58 (ddd, *J* = 12.9, 9.1, 6.1 Hz, 1H), 2.47 – 2.42 (m, 1H), 2.41 (s, 3H), 1.99 – 1.86 (m, 4H), 1.80 (ddd, *J* = 14.2, 9.1, 5.9 Hz, 1H), 1.69 – 1.61 (m, 1H). **^{13}C{^{1}H} NMR** (126 MHz, $CDCl_3$) δ 211.3, 205.9, 179.2, 152.4, 144.1, 136.6, 135.3, 129.4 (2C), 128.5 (2C), 122.1, 99.7, 85.8, 58.1, 58.1, 55.1, 44.3, 40.3, 32.9, 23.5, 22.1, 21.6. **IR (KBr, cm^{-1}):** 2955, 2924, 2855, 1769, 1588, 1408, 1277, 1182, 972, 814, 749, 658. **HRMS (ESI-TOF)/m/z:** [M+Na]$^+$ calcd for $C_{23}H_{24}N_2O_5SNa$ 463.1298; found 463.1295.

9-chloro-1-tosyl-2,3,4,4a,6,7-hexahydro-7a,12a-methanobenzo²pyrido[3,2-g]azocine-5,13(1H)-dione (4l)

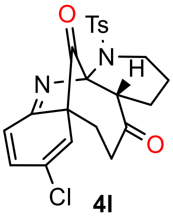

4l

Yield: 63% (40 mg), white solid, m.p. 68.6 – 68.8 °C. R_f = 0.3 (EtOAc/ petroleum ether = 1:2). **^{1}H NMR (500 MHz, $CDCl_3$)** δ 7.72 (d, *J* = 8.1 Hz, 2H), 7.30 (d, *J* = 8.1 Hz, 2H), 6.63 (dd, *J* = 10.0, 1.7 Hz, 1H), 6.55 (d, *J* = 10.0 Hz, 1H), 6.53 (s, 1H), 3.35 (t, *J* = 11.2 Hz, 1H), 3.26 (d, *J* = 10.5 Hz, 1H), 2.97 – 2.90 (m, 1H), 2.61 (ddd, *J* = 12.8, 9.6, 6.3 Hz, 1H), 2.48 – 2.43 (m, 1H), 2.42 (s, 3H), 2.03 – 1.79 (m, 5H), 1.61 – 1.54 (m, 1H). **^{13}C{^{1}H} NMR (126 MHz, $CDCl_3$)** δ 209.1, 205.1, 177.1, 144.3, 138.0, 135.3, 129.5(2C),

128.4(2C), 128.2, 127.9, 122.9, 85.3, 59.8, 58.0, 44.2, 40.1, 30.6, 23.3, 21.9, 21.6. **IR (KBr, cm^{-1}):** 2954, 2867, 1772, 1625, 1402, 1272, 1083, 858, 747, 666, 584. **HRMS(ESI-TOF)/m/z:** $[M+Na]^+$ calcd for $C_{22}H_{21}ClN_2O_4SNa$ 467.0803; found 467.0802.

9-fluoro-1-tosyl-2,3,4,4a,6,7-hexahydro-7a,12a-methanobenzo^{2}pyrido[3,2-g]azocine-5,13(1H)-dione (4m)

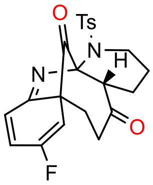

4m

Yield: 80% (44 mg), pink solid, m.p. 91.7 – 92.8 °C. R_f = 0.3 (EtOAc/ petroleum ether = 1:2). **^{1}H NMR (500 MHz, CDCl$_3$)** δ 7.72 (d, *J* = 7.7 Hz, 2H), 7.29 (d, *J* = 7.8 Hz, 2H), 6.68 – 6.54 (m, 2H), 5.99 (d, *J* = 9.0 Hz, 1H), 3.35 (t, *J* = 12.1 Hz, 1H), 3.26 (d, *J* = 11.5 Hz, 1H), 2.97 – 2.89 (m, 1H), 2.65 – 2.54 (m, 1H), 2.49 – 2.43 (m, 1H), 2.42 (s, 3H), 2.03 – 1.77 (m, 5H), 1.62 – 1.55 (m, 1H). **^{13}C{^{1}H} NMR (126 MHz, CDCl$_3$)** δ 209.8, 205.2, 177.6, 155.9 (d, J = 252.5 Hz), 144.3, 135.3, 131.9 (d, J = 37.6 Hz), 129.5 (2C), 128.4 (2C), 123.9 (d, J = 8.6 Hz), 108.4 (d, J = 20.9 Hz), 85.6, 57.98, 57.95, 44.2, 40.1, 31.2 (d, J = 4.5 Hz), 23.3, 21.9, 21.6. **^{19}F NMR (376 MHz, CDCl$_3$)** δ -115.9. **IR (KBr, cm^{-1}):** 2955, 1772, 1640, 1489, 1331, 1154, 1009, 962, 854, 732, 696, 587. **HRMS (ESI-TOF) /m/z:** $[M+Na]^+$ calcd for $C_{22}H_{21}FN_2O_4SNa$ 451.1098; found 451.1098.

methyl-5,13-dioxo-1-tosyl-1,2,3,4,4a,5,6,7-octahydro-7a,12a-methanobenzo[b]pyrido[3,2-g]azocine-9-carboxylate (4n)

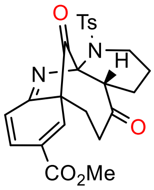

4n

Yield: 71% (67 mg), pink solid, m.p. 121.1 - 121.4 °C. R_f = 0.3 (EtOAc/ petroleum ether = 1:2). **^{1}H NMR (500 MHz, CDCl$_3$)** δ 7.67 (d, J = 8.1 Hz, 2H), 7.49 (s, 1H), 7.29 – 7.25 (m, 3H), 6.59 (d, J = 10.0 Hz, 1H), 3.80 (s, 3H), 3.28 – 3.26 (m, 1H), 2.98 – 2.91 (m, 1H), 2.62 (ddd, J = 12.9, 9.5, 6.2 Hz, 1H), 2.45 (dt, J = 12.6, 5.9 Hz, 1H), 2.40 (s, 3H), 2.02 – 1.87 (m, 4H), 1.75 (dd, J = 22.8, 5.9 Hz, 2H), 1.67 – 1.55 (m, 1H). **^{13}C{^{1}H} NMR (126 MHz, CDCl$_3$)** δ 208.9, 205.0, 177.9, 164.8, 144.3, 139.6, 135.1, 133.9, 129.5 (2C), 128.4 (2C), 127.1, 121.8, 85.1, 59.0, 58.0, 52.4, 44.2, 40.1, 30.2, 23.3, 21.8, 21.6. **IR (KBr, cm^{-1}):** 2955, 2869, 1773, 1631, 1441, 1334, 1272, 1088, 968, 813. **HRMS (ESI-TOF)/m/z:** $[M+H]^+$ calcd for $C_{24}H_{25}N_2O_6S$ 469.1428; found 469.1431.

8-fluoro-1-tosyl-2,3,4,4a,6,7-hexahydro-7a,12a-methanobenzo^{2}pyrido[3,2-g]azocine-5,13(1H)-dione(4o) (3o is heated at 80 °C for 36 h)

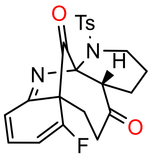

4o

Yield: 62% (41 mg), yellow solid, m.p. 130.3 – 130.8 °C. R_f = 0.3 (EtOAc/ petroleum ether = 1:1). **^{1}H NMR (500 MHz, CDCl$_3$)** δ 7.71 (d, J = 8.1 Hz, 2H), 7.28 (d, J = 8.1 Hz, 2H), 6.74 (dt, J = 9.6, 6.4 Hz, 1H), 6.39 (d, J = 9.7 Hz, 1H), 5.81 (dd, J = 10.1, 6.7 Hz, 1H), 3.33 – 3.22 (m, 2H), 2.95 (dd, J = 9.1, 7.0 Hz, 1H), 2.72 (ddd, J = 12.5, 9.8, 6.5 Hz, 1H), 2.50 (dt, J = 12.5, 5.5 Hz, 1H), 2.41 (s, 3H), 2.35 (dt, J = 11.9, 5.8 Hz, 1H), 1.99 – 1.84 (m, 4H), 1.65 – 1.56 (m, 1H). **^{13}C{^{1}H} NMR (126 MHz, CDCl$_3$)** δ 205.9, 204.7, 177.8, 161.5 (d, J = 286.9 Hz), 144.3, 135.8 (d, J = 7.8 Hz), 135.2, 129.5 (2C), 128.4 (2C), 118.3 (d, J = 6.0 Hz), 101.9 (d, J = 17.6 Hz), 84.5, 58.5 (d, J = 20.2 Hz), 58.3, 44.2, 40.3, 28.9 (d, J = 4.0 Hz), 23.1, 21.6, 21.4. **^{19}F NMR (471 MHz, CDCl$_3$)** δ -108.0. **IR (KBr, cm^{-1}):** 2925, 2862, 1774, 1633, 1450, 1236, 1154, 854, 727, 659, 580. **HRMS(ESI-TOF)/m/z:** $[M+H]^+$ calcd for $C_{22}H_{22}FN_2O_4S$ 429.1279; found 429.1272.

11-methyl-1-tosyl-2,3,4,4a,6,7-hexahydro-7a,12a-methanobenzo[b]pyrido[3,2-g]azocine-5,13(1H)-dione (4p)

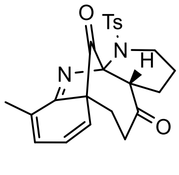

4p

Yield: 60% (30 mg), yellow solid, R_f = 0.4 (EtOAc/ petroleum ether = 3:1). **^{1}H NMR (500 MHz, CDCl$_3$)** δ 7.65 (d, J = 8.2 Hz, 2H), 7.26 (d, J = 8.3 Hz, 2H), 6.45 (d, J = 5.7 Hz, 1H), 6.41 (d, J = 9.2 Hz, 1H), 6.19 (dd, J = 9.2, 5.8 Hz, 1H), 3.37 – 3.31 (m, 1H), 3.28 (t, J = 11.3 Hz, 1H), 2.93 (dd, J = 10.4, 5.8 Hz, 1H), 2.55 (ddd, J = 12.9, 9.1, 6.1 Hz, 1H), 2.41 (s, 3H), 2.40 – 2.35 (m, 1H), 2.07 (s, 3H), 1.98 – 1.85 (m, 4H), 1.71 (ddd, J = 13.8, 9.1, 5.8 Hz, 2H). **^{13}C NMR (126 MHz, CDCl$_3$)** δ 211.4, 206.1, 180.7, 143.9, 135.5, 131.6, 130.3, 130.2, 129.3 (2C), 128.5 (2C), 122.8, 85.3, 59.2, 58.1, 44.3, 40.0, 31.2, 23.5, 22.0, 21.6, 16.0. **IR (KBr, cm^{-1})** 2924, 1708, 1648, 1565, 1547, 1461, 1366, 1334, 1082, 749, 574, 543. **HRMS (ESI-TOF)/m/z:** $[M+H]^+$ calcd for $C_{23}H_{25}N_2O_4S$ 425.1530; found 425.1532.

Typical procedure for preparation of 4q (Note: **4q** will deteriorate during separation)

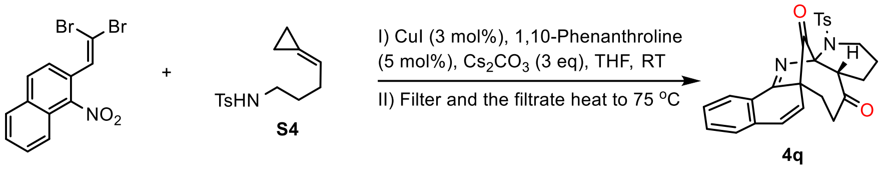

A mixture of 2-(2,2-dibromovinyl)-1-nitronaphthalene (1.1 eq), **S4** (1 eq), CuI (3 mol%), 1,10-phenantroline (5 mol%), caesium carbonate (3 eq) in THF (0.1 M) were stirred at r.t. for 12 h under N_2 atmosphere in a 25 mL Schlenk tube. After completion (12 h, monitored by TLC),. the mixture was filtrated through celite, the resulting filtrate was added to a dried 25 mL schlenk tube under nitrogen atmosphere and put in a 75 °C oil bath stirring for 12 h. After cooled to room temperature, the mixture was transfered to a 25 mL round bottom flask and evaporated under reduced pressure. The residue was purified by column chromatography (silica gel, petroleum ether/EtOAc = 2:1) to give **4q**.

1-tosyl-2,3,4,4a,6,7-hexahydro-7a,14a-methanonaphtho[1,2-b]pyrido[3,2-g]azocine-5,15(1H)-dione. (4q)

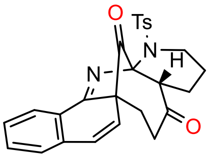

4q

Yield: 62% (36 mg), yellow solid, m.p. 195.4 - 196.8 °C. R_f = 0.3 (EtOAc/ petroleum ether = 1:1). **^{1}H NMR (500 MHz, CDCl$_3$)** δ 7.92 (d, J = 7.6 Hz, 1H), 7.58 (d, J = 7.9 Hz, 2H), 7.53 (t, J = 7.5 Hz, 1H), 7.38 (t, J = 7.5 Hz, 1H), 7.27 (d, J = 8.0 Hz, 1H), 7.12 (d, J = 8.0 Hz, 2H), 6.64 (d, J = 9.4 Hz, 1H), 6.44 (d, J = 9.4 Hz, 1H), 3.38 (d, J = 10.7 Hz, 1H), 3.28 (t, J = 11.4 Hz, 1H), 2.98 (dd, J = 12.3, 3.9 Hz, 1H), 2.63 (ddd, J = 12.2, 10.3, 6.7 Hz, 1H), 2.40 (dt, J = 12.4, 5.5 Hz, 1H), 2.36 (s, 3H), 2.03 – 1.95 (m, 2H), 1.92 – 1.85 (m, 1H), 1.78 – 1.71 (m, 1H), 1.70 – 1.62 (m, 2H). **^{13}C{^{1}H} NMR (126 MHz, CDCl$_3$)** δ 211.1, 205.5, 177.9, 143.9, 135.2, 132.7, 129.2(3C), 128.7(2C), 128.4, 128.2, 127.8, 127.0, 126.4, 125.7, 85.7, 58.3, 57.4, 44.5, 40.4, 30.4, 23.3, 21.5, 21.4. **IR (KBr, cm^{-1}):** 3064, 2940, 2224, 1956, 1659, 1604, 1577, 1415, 1169, 1076, 737.5, 672.4, 571.9, 546. **HRMS(ESI-TOF)/m/z:** $[M+H]^+$ $C_{26}H_{25}N_2O_4S$ calcd for 461.1530; found 461.1531 .

1-tosyl-2,3,4,4a,6,7-hexahydro-7a,12a-methanodipyrido[2,3-b:2',3'-g]azocine-5,13(1H)-dione. (4r)

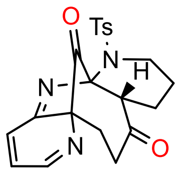

4r

Yield: 63% (34 mg), Yellow liquid, R_f = 0.3 (EtOAc/ petroleum ether = 3:1). **^{1}H NMR (500 MHz, $CDCl_3$)** δ 8.21 (s, 1H), 7.77 (d, *J* = 8.0 Hz, 2H), 7.30 (s, 2H), 6.93 (d, *J* = 9.7 Hz, 1H), 6.52 (dd, *J* = 9.6, 2.5 Hz, 1H), 3.47 (t, *J* = 12.2 Hz, 1H), 3.24 (d, *J* = 11.4 Hz, 1H), 3.03 (dd, *J* = 10.2, 5.9 Hz, 1H), 2.77 – 2.67 (m, 1H), 2.43 (d, *J* = 7.9 Hz, 4H), 2.21 – 2.10 (m, 2H), 2.00 – 1.89 (m, 3H), 1.68 (ddd, *J* = 13.5, 10.5, 6.1 Hz, 1H). **^{13}C{^{1}H} NMR (126 MHz, $CDCl_3$)** δ 208.5, 204.8, 178.2, 155.6, 144.3, 135.6, 129.5 (2C), 128.3 (2C), 128.2, 127.7, 83.9, 74.3, 58.4, 43.9, 39.7, 30.0, 23.2, 21.6, 21.5. **IR (KBr, cm^{-1}):** 2924, 1777, 1640, 1448, 1327, 1236, 1152, 969, 851, 787, 660, 575. **HRMS (ESI-TOF)/m/z:** $[M+H]^+$ calcd for $C_{21}H_{22}N_3O_4S$ 412.1326; found 412.1321.

4a-ethyl-1-tosyl-2,3,4,4a,6,7-hexahydro-7a,12a-methanobenzo^{2}pyrido[3,2-g]azocine-5,13(1H)-dione. (4s)

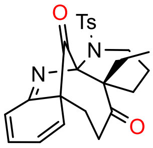

4s

Yield: 36% (21 mg), white solid, m.p. 143.1 – 145.9 °C. R_f = 0.3 (EtOAc/ petroleum ether = 1:3). **^{1}H NMR (500 MHz, $CDCl_3$)** δ 7.74 (d, J = 8.3 Hz, 2H), 7.28 (d, J = 8.1 Hz, 2H), 6.63 (ddd, J = 9.7, 5.6, 1.1 Hz, 1H), 6.45 (d, J = 9.2 Hz, 1H), 6.40 (d, J = 9.7 Hz, 1H), 6.21 (dd, J = 9.2, 5.6 Hz, 1H), 3.51 – 3.44 (m, 1H), 3.26 (dd, J = 11.5, 4.7 Hz, 1H), 3.04 – 2.94 (m, 1H), 2.42 (s, 3H), 2.28 – 2.17 (m, 2H), 2.09 – 2.00 (m, 2H), 1.88 (dq, J = 13.4, 4.5, 3.4 Hz, 3H), 1.81 – 1.73 (m, 1H), 1.66 (dq, J = 13.8, 5.0 Hz, 1H), 0.61 (t, J = 7.6 Hz, 3H). **^{13}C{^{1}H} NMR (126 MHz, $CDCl_3$)** δ 208.7, 208.3, 178.5, 143.8, 136.5, 135.0, 132.9, 129.4 (2C), 128.1 (2C), 122.4, 121.4, 86.6, 58.6, 58.4, 44.5, 35.8, 29.4, 21.6, 20.9, 20.7, 19.1, 7.1. **IR (KBr, cm^{-1}):** 3060, 2922, 1763, 1632, 1456, 1200, 968, 789. **HRMS(ESI-TOF)/m/z:** $[M+H]^+$ calcd for $C_{24}H_{27}N_2O_4S$ 439.1686; found 439.1683.

(4aR)-4a-phenyl-1-tosyl-2,3,4,4a,6,7-hexahydro-7a,12a-methanobenzo^{2}pyrido[3,2-g]azocine-5,13(1H)-dione (4t)

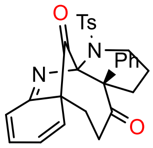

4t

Yield: 21% (12 mg), yellow solid, m.p. 158.5 – 159.1 °C. R_f = 0.3 (EtOAc/ petroleum ether = 2:1). **^{1}H NMR (500 MHz, $CDCl_3$)** δ 7.77 (d, *J* = 8.3 Hz, 2H), 7.42 (brs, 2H), 7.36 (d, *J* = 8.0 Hz, 2H), 7.25 – 7.17 (m, 3H), 6.67 (ddd, *J* = 9.7, 5.6, 1.1 Hz, 1H), 6.45 (d, *J* = 9.8 Hz, 1H), 6.40 (d, *J* = 9.2 Hz, 1H), 6.22 (dd, *J* = 9.2, 5.4 Hz, 1H), 3.51 (td, *J* = 13.3, 3.9 Hz, 1H), 3.38 (dd, *J* = 11.6, 3.9 Hz, 1H), 2.71 (td, *J* = 12.7, 7.7 Hz, 1H), 2.58 (td, *J* = 13.6, 3.3 Hz, 1H), 2.47 (s, 3H), 2.19 (ddd, *J* = 12.2, 6.1, 1.9 Hz, 1H), 1.92 (td, *J* = 13.1, 6.2 Hz, 1H), 1.77 – 1.61 (m, 3H), 1.44 – 1.34 (m, 1H). **^{13}C{^{1}H} NMR (126 MHz, $CDCl_3$)** δ 207.6, 206.0, 179.0, 144.1, 137.7, 136.1, 135.3, 133.2, 129.4 (2C), 129.3, 128.3 (2C), 127.9, 127.1, 122.3, 121.4, 85.7, 64.6, 58.7, 44.8, 37.8, 30.4, 29.8, 21.6, 19.3. **IR (KBr, cm^{-1}):** 2923, 1770, 1742, 1646, 1547, 1531, 1516, 1365, 1266, 1160, 746, 704, 660. **HRMS(ESI-TOF)/m/z:** $[M+H]^+$ calcd for $C_{28}H_{27}N_2O_4S$ 487.1686; found 487.1683.

Typical procedure for preparation of 4u' and 4v'

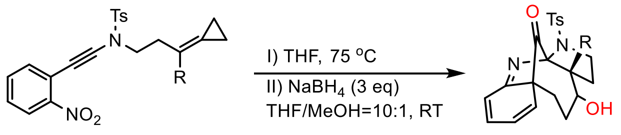

3 **4'**

A dried 25 mL Schlenk tube was flushed with N_2 three times. The solution of **3** (1 eq) in the THF (0.03

M) was added to the tube under nitrogen atmosphere. The resuting mixture was put in a 75 °C oil bath and stirred for 12 h. When the rearrangement reaction mixture was cooled to room temperature, MeOH (0.5 mL) and $NaBH_4$ (3 eq) were added, then stirred for another 1 h. The mixture was quenched with water (3 mL), extracted with EtOAc (3*3 mL), dried over Na_2SO_4 and concentrated under reduced pressure. The residue was purified by column chromatography (silica gel, petroleum ether/EtOAc = 1:3) gave **4u'** and **4v'**.

3'-acryloyl-6-(dimethylamino)-1'-tosylspiro[indoline-2,2'-piperidin]-3-one. (4u')

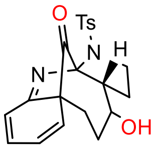

4u'

Yield: 58% (34 mg), yellow solid, m.p. 171.1 – 171.8 °C. R_f = 0.3 (EtOAc/ petroleum ether = 3:1). **^{1}H NMR (500 MHz, $CDCl_3$)** δ 7.73 (d, J = 8.1 Hz, 2H), 7.28 (d, J = 8.6 Hz, 2H), 6.76 (dd, J = 9.5, 5.7 Hz, 1H), 6.50 (dd, J = 15.2, 9.5 Hz, 2H), 6.31 (dd, J = 9.2, 5.7 Hz, 1H), 4.02 (s, 1H), 3.67 (t, J = 9.2 Hz, 1H), 3.49 (q, J = 9.3 Hz, 1H), 2.64 – 2.52 (m, 1H), 2.46 (ddd, J = 13.5, 5.8, 2.9 Hz, 1H), 2.41 (s, 3H), 2.23 – 2.15 (m, 1H), 2.10 (dt, J = 12.3, 6.4 Hz, 1H), 2.01 (td, J = 13.7, 5.4 Hz, 1H), 1.85 (dd, J = 13.6, 3.9 Hz, 2H), 1.68 (td, J = 14.3, 6.1 Hz, 1H). **^{13}C{^{1}H} NMR (126 MHz, $CDCl_3$)** δ 198.6, 192.8, 160.3, 157.1, 143.4, 136.3, 136.1, 129.3 (2C), 129.1, 128.6 (2C), 126.0, 110.8, 107.0, 92.4, 78.3, 51.8, 44.5, 40.3, 23.7, 23.4, 21.6. **IR (KBr, cm^{-1}):** 2924, 1617, 1396, 1238, 1117, 989, 862, 790, 662, 564. **HRMS (ESI-TOF)/m/z:** $[M+Na]^+$ calcd for $C_{21}H_{22}N_2O_4SNa$ 421.1192; found 421.1191.

4-hydroxy-3a-methyl-1-tosyl-2,3,3a,4,5,6-hexahydro-1H-6a,11a-methanobenzo^{2}pyrrolo[3,2-g]azocin-12-one. (4v')

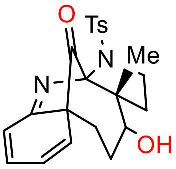

4v

Yield: 45% (25 mg), pink solid, m.p. 223.1 - 223.7 °C, R_f = 0.3 (EtOAc/ petroleum ether = 3:1). **^{1}H NMR (500 MHz, $CDCl_3$)** δ 7.73 (d, *J* = 8.1 Hz, 2H), 7.27 (d, *J* = 8.2 Hz, 2H), 6.73 (dd, *J* = 9.6, 5.6 Hz, 1H), 6.50 (d, *J* = 9.3 Hz, 1H), 6.45 (d, *J* = 9.7 Hz, 1H), 6.29 (dd, *J* = 9.3, 5.7 Hz, 1H), 3.75 (t, *J* = 9.7 Hz, 1H), 3.71 (d, *J* = 4.1 Hz, 1H), 3.40 (q, *J* = 9.4 Hz, 1H), 2.93 (q, *J* = 10.6 Hz, 1H), 2.41 (s, 3H), 2.12 (brs, 1H), 2.06 – 1.93 (m, 3H), 1.90 – 1.84 (m, 1H), 1.71 (dd, *J* = 12.1, 7.2 Hz, 1H), 1.12 (s, 3H). **^{13}C{^{1}H} NMR** (126 MHz, $CDCl_3$) δ 207.4, 181.0, 143.6, 136.2, 136.0, 133.7, 129.3 (2C), 128.3 (2C), 123.0, 120.5, 95.8, 73.0, 58.1, 55.5, 45.3, 32.0, 30.8, 27.8, 21.6, 21.0. **IR (KBr, cm^{-1}):** 3469, 2925, 1771, 1636, 1399, 1260, 905, 730, 666. **HRMS (ESI-TOF)/m/z:** $[M+H]^+$ calcd for $C_{22}H_{25}N_2O_4S$ 413.1530; found 413.1527.

3'-acryloyl-6-(dimethylamino)-1'-tosylspiro[indoline-2,2'-piperidin]-3-one. (4w')

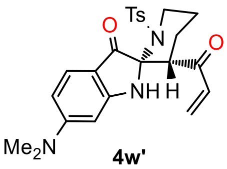

4w'

Yield: 58% (34 mg), red liquid, R_f = 0.3 (EtOAc/ petroleum ether = 2:1). **^{1}H NMR (500 MHz, $CDCl_3$)** δ 7.80 (d, *J* = 8.1 Hz, 2H), 7.40 (d, *J* = 8.8 Hz, 1H), 7.24 (d, *J* = 8.1 Hz, 2H), 6.24 (d, *J* = 8.8 Hz, 1H), 6.19 (dd, *J* = 17.4, 10.3 Hz, 1H), 6.10 (d, *J* = 17.3 Hz, 1H), 5.85 (s, 1H), 5.62 (d, *J* = 10.3 Hz, 1H), 5.44 (s, 1H), 3.54 – 3.44 (m, 2H), 3.07 (t, *J* = 13.0 Hz, 1H), 3.01 (s, 6H), 2.40 (s, 3H), 1.85 – 1.73 (m, 3H), 1.65 – 1.57 (m, 1H) **^{13}C NMR (126 MHz, $CDCl_3$)** δ 198.6, 192.8, 160.3, 157.1, 143.4, 136.3, 136.1, 129.3, 129.1 (2C), 128.6 (2C), 126.0, 110.8, 107.0, 92.4, 78.3, 51.8, 44.5, 40.3 (2C), 23.7, 23.4, 21.6. **IR (KBr, cm^{-1}):** 2924, 1617, 1396, 1238, 1117, 989, 862, 790, 662, 564. **HRMS(ESI-TOF)/m/z:** $[M+Na]^+$ calcd for $C_{24}H_{27}N_3O_4SNa$ 476.1614; found 476.1611

1-(methylsulfonyl)-2,3,4,4a,6,7-hexahydro-7a,12a-methanobenzopyrido[3,2-g]azocine-5,13(1H)-dione. (4x)

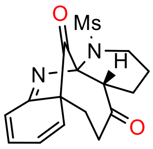

4x

Yield: 78% (44 mg), White solid, m.p. 85.8 – 87.1 °C, R_f = 0.3 (EtOAc/ petroleum ether = 1:2). **^{1}H NMR (500 MHz, CDCl$_3$)** δ 6.68 (dd, *J* = 9.8, 5.6 Hz, 1H), 6.45 (t, *J* = 8.3 Hz, 2H), 6.25 (dd, *J* = 9.3, 5.6 Hz, 1H), 3.61 (dt, *J* = 11.5, 3.6 Hz, 1H), 3.48 (td, *J* = 12.2, 2.9 Hz, 1H), 2.96 – 2.90 (m, 1H), 2.88 (s, 3H), 2.57 (ddd, *J* = 12.9, 9.3, 6.1 Hz, 1H), 2.43 (dt, *J* = 12.6, 6.1 Hz, 1H), 2.11 – 2.03 (m, 1H), 2.00 – 1.87 (m, 3H), 1.78 (dddt, *J* = 22.7, 14.2, 9.3, 5.3 Hz, 2H) **^{13}C{^{1}H} NMR (126 MHz, CDCl$_3$)** δ 210.3, 205.6, 179.7, 136.0, 132.7, 122.7, 121.2, 85.0, 59.2, 57.9, 44.6, 41.3, 40.1, 30.8, 23.8, 22.2. **IR (KBr, cm^{-1}):** 3041, 2938, 1770, 1578, 1329, 1199, 1055, 970, 745, 665, 521. **HRMS(ESI-TOF)/m/z:** [M+H]$^+$ calcd for $C_{16}H_{19}N_2O_4S$ 335.1060; found 335.1059.

(4aS)-1-((4-nitrophenyl)sulfonyl)-2,3,4,4a,6,7-hexahydro-7a,12a-methanobenzo[b]pyrido [3,2-g]azocine-5,13(1H)-dione (4y)

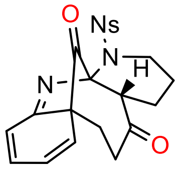

4y

Yield: 65% (62 mg), pink solid, m.p. 160.4 – 162.1 °C, R_f = 0.3 (EtOAc/ petroleum ether = 1:2). **^{1}H NMR (500 MHz, CDCl$_3$)** δ 8.08 – 8.02 (m, 1H), 7.73 – 7.68 (m, 2H), 7.66 – 7.60 (m, 1H), 6.71 (dd, *J* = 9.3, 5.3 Hz, 1H), 6.51 (d, *J* = 9.7 Hz, 1H), 6.44 (d, *J* = 9.2 Hz, 1H), 6.28 (dd, *J* = 9.2, 5.6 Hz, 1H), 3.71 – 3.58 (m, 2H), 2.90 (dd, *J* = 12.0, 4.5 Hz, 1H), 2.56 – 2.43 (m, 2H), 2.06 – 1.97 (m, 2H), 1.97 – 1.93 (m, 1H), 1.93 – 1.87 (m, 1H), 1.81 – 1.72 (m, 2H). **^{13}C{^{1}H} NMR (126 MHz, CDCl$_3$)** δ 210.5, 205.7, 179.4, 136.0, 133.71, 133.68, 132.1, 131.8, 130.6, 123.9, 123.2, 121.5, 85.9, 59.6, 58.1, 44.9, 39.9, 31.8, 23.7, 22.7. **IR (KBr, cm^{-1}):** 2926, 1642, 1579, 1452, 1264, 1099, 875, 745, 561. **HRMS (ESI – TOF)/m/z:** [M+H]$^+$ calcd for $C_{21}H_{20}N_3O_6S^+$ 442.1067; found 442.1063.

Tert-butyl(4aS)-5,13-dioxo-3,4,4a,5,6,7-hexahydro-7a,12a-methanobenzo[b]pyrido[3,2-g]azocine-1(2H)-carboxylate (4z)

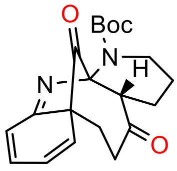

4z

Yield: 62% (33 mg), yellow oil, R_f = 0.4 (EtOAc/ petroleum ether = 1:2). **^{1}H NMR (500 MHz, CDCl$_3$)** δ 6.56 (dd, *J* = 9.5, 5.6 Hz, 1H), 6.45 (d, *J* = 9.2 Hz, 1H), 6.32 (d, *J* = 9.4 Hz, 1H), 6.16 (dd, *J* = 9.3, 5.6 Hz, 1H), 3.74 (dd, *J* = 12.5, 3.5 Hz, 1H), 3.19 (t, *J* = 9.8 Hz, 1H), 2.80 (dd, *J* = 12.2, 3.2 Hz, 1H), 2.53 (s, 1H), 2.35 (dt, *J* = 12.2, 5.7 Hz, 1H), 1.92 – 1.85 (m, 2H), 1.85 – 1.78 (m, 2H), 1.67 (ddd, *J* = 13.7, 9.4, 6.1 Hz, 2H), 1.26 (s, 9H). **^{13}C{^{1}H} NMR (126 MHz, CDCl$_3$)** δ 208.6, 206.4, 179.3, 155.8, 135.3, 133.3, 122.3, 121.1, 82.9, 81.7, 59.0, 56.3, 42.8, 40.5, 30.9, 28.1 (3C), 23.0, 21.8. **IR (KBr, cm^{-1}):** 2929, 2867, 1765, 1684, 1540, 1364, 1270, 1073, 893, 782, 738. **HRMS (ESI – TOF)/m/z:** [M+H]$^+$ calcd for $C_{20}H_{25}N_2O_4$ 357.1809; found 357.1805.

1'-tosyl-2',3',4',4a'-tetrahydro-1'H,12'H-spiro[cyclobutane-1,5'-pyrido[2',3':3,4]isoxazolo[2,3-a]indol]-12'-one. (4aa')

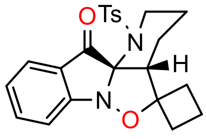

4aa'

Yield: 90% (44 mg), brown oil, R_f = 0.3 (EtOAc/ petroleum ether = 1:1). **^{1}H NMR (500 MHz, CDCl$_3$)** δ 7.72 (d, *J* = 8.1 Hz, 2H), 7.69 (d, *J* = 7.7 Hz, 1H), 7.64 (t, *J* = 7.6 Hz, 1H), 7.48 (d, *J* = 8.1 Hz, 1H), 7.28 (d, *J* = 7.4 Hz, 1H), 7.25 (d, *J* = 8.1 Hz, 2H), 3.63 (dt, *J* = 10.6, 4.8 Hz, 1H), 3.12 (td, *J* = 10.6, 4.5 Hz, 1H), 2.75 (dd, *J* = 8.8, 7.1 Hz, 1H), 2.40 (s, 3H), 2.17 (t, *J* = 7.9 Hz, 2H), 2.04 – 1.94 (m, 1H), 1.82 – 1.70 (m, 2H), 1.67 – 1.44 (m, 3H), 1.42 – 1.33 (m, 2H). **^{13}C{^{1}H} NMR (126 MHz, CDCl$_3$)** δ 198.1,

160.5, 143.5, 136.4, 136.3, 129.3 (2C), 128.2 (2C), 126.1, 126.1, 123.7, 119.7, 90.4, 86.9, 51.9, 43.7, 38.8, 29.9, 22.8, 21.6, 20.8, 14.0. **IR (KBr, cm^{-1}):** 3058, 1732, 1597, 1474, 1338, 1162, 1023, 808, 666, 598. **HRMS(ESI-TOF)/m/z:** $[M+H]^+$ calcd for $C_{23}H_{25}N_2O_4S$ 425.1530; found 425.1527.

Procedure for preparation of 4ab'.

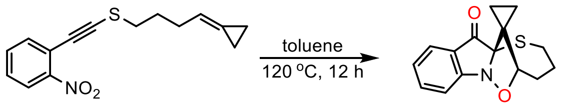

3ab **4ab'**, 67%

To a 25 mL Schlenk tube with a magnetic bar were added a solution of **3ab** (1.0 eq, 0.15 mmol) in PhMe (0.03 M) at r.t. under N_2 atmosphere. The mixture was stirred at a 120 °C oil bath for 12 h. After the reaction was completed (12 h, monitored by TLC), the solvent was concentrated in vacuo. The residue was purified by column chromatography (silica gel, petroleum ether/EtOAc = 3:1) to afford **4ab'** (67%).

(5'R,12a'S)-2',3',4',5'-tetrahydro-12'H-spiro[cyclopropane-1,13'-[5,12a]methano[1,4,2] oxathiazocino[2,3-a]indol]-12'-one (4ab')

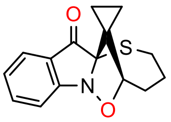

4ab'

Yield: 67% (25 mg), yellow oil, R_f = 0.3 (EtOAc/ petroleum ether = 1:4). **^{1}H NMR (500 MHz, CDCl$_3$)** δ 7.68 (t, *J* = 7.7 Hz, 1H), 7.64 (d, *J* = 7.7 Hz, 1H), 7.42 (d, *J* = 8.1 Hz, 1H), 7.23 (t, *J* = 7.5 Hz, 1H), 4.35 (s, 1H), 3.09 (dd, *J* = 13.5, 10.7 Hz, 1H), 3.01 (dd, *J* = 13.9, 8.4 Hz, 1H), 2.43 – 2.34 (m, 1H), 2.33 – 2.23 (m, 1H), 2.09 – 2.02 (m, 1H), 1.56 – 1.51 (m, 1H), 1.15 (dt, *J* = 10.0, 6.6 Hz, 1H), 1.01 (dt, *J* = 11.4, 6.0 Hz, 1H), 0.84 (dt, *J* = 10.6, 5.6 Hz, 1H), 0.63 (dt, *J* = 12.1, 6.9 Hz, 1H). **^{13}C{^{1}H} NMR (126 MHz, CDCl$_3$)** δ 193.2, 163.1, 137.5, 125.5, 124.4, 122.7, 118.3, 89.7, 86.8, 33.8, 33.5, 29.7, 26.5, 16.1, 6.6. **IR (KBr, cm^{-1}):** 3133, 1636, 1617, 1401, 1322, 1192, 1093, 993, 765, 613. **HRMS (ESI – TOF)/m/z:** $[M+H]^+$ calcd for $C_{15}H_{16}NO_2S$ 274.0896; found 274.0892.

Procedure for preparation of 4ac'.

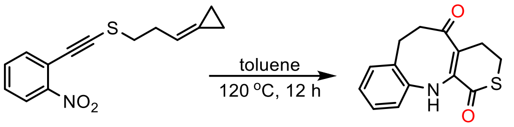

3ac **4ac'**, 25%

To a 25 mL Schlenk tube with a magnetic bar were added a solution of **3ac** (1.0 eq, 0.15 mmol) in PhMe (0.03 M) at r.t. under N_2 atmosphere. The mixture was stirred at a 120 °C oil bath for 12 h. After the reaction was completed (monitored by TLC), the solvent was concentrated in vacuo. The residue was purified by column chromatography (silica gel, petroleum ether/EtOAc = 3:1) to afford **4ac'** (25%).

4,6,7,12-tetrahydro-1H-benzo[b]thiopyrano[4,3-g]azocine-1,5(3H)-dione (4ac')

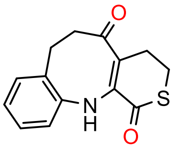

4ac'

Yield: 25% (10 mg), yellow oil, R_f = 0.3 (EtOAc/ petroleum ether = 1:30). **^{1}H NMR (500 MHz, CDCl$_3$)** δ 7.07 – 7.01 (m, 3H), 6.91 – 6.85 (m, 1H), 6.74 (brs, 1H), 3.10 – 3.04 (m, 4H), 3.00 (t, *J* = 6.3 Hz, 2H), 2.74 (t, *J* = 6.5 Hz, 2H). **^{13}C{^{1}H} NMR (126 MHz, CDCl$_3$)** δ 206.1, 191.1, 140.6, 140.0, 135.0, 130.6, 127.4, 126.4, 125.7, 122.1, 41.6, 27.8, 27.7, 26.2. **IR (KBr, cm^{-1}):** 3155, 2920, 1716, 1630, 1561, 1312, 1219, 1066, 841, 763, 560. **HRMS (ESI – TOF)/m/z:** $[M+H]^+$ calcd for $C_{14}H_{14}NO_2S$ 260.0740; found 260.0736.

Procedure for preparation of 4ad' and 4ad''.

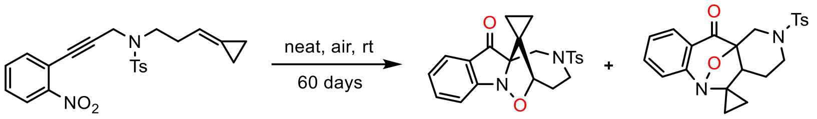

3ad **4ad'**, 25% **4ad''**, 32%

A round-bottom flask was charged with substrates **3ad** (1.0 eq, 0.15 mmol) and exposure to air at room temperature for two months. The crude product was purified by column chromatography (silica gel, petroleum ether/EtOAc = 4:1) to afford **4ad'** (25%) and **4ad''** (32%).

(2'R,6a'S)-5'-tosyl-3',4',5',6'-tetrahydro-2'H,7'H-spiro[cyclopropane-1,13'-[2,6a]methano [1,2,5]oxadiazocino[2,3-a]indol]-7'-one (4ad')

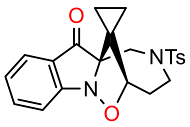

4ad'

Yield: 25% (15 mg), white solid, m.p. 140.8 – 141.9 °C , R_f = 0.3 (EtOAc/ petroleum ether = 1:5). **^{1}H NMR (500 MHz, CDCl$_3$)** δ 7.69 (d, *J* = 8.0 Hz, 2H), 7.66 (d, *J* = 7.7 Hz, 1H), 7.62 (d, *J* = 7.7 Hz, 1H), 7.39 (d, *J* = 8.1 Hz, 1H), 7.29 (d, *J* = 8.0 Hz, 2H), 7.24 (d, *J* = 7.5 Hz, 1H), 4.34 (d, *J* = 12.9 Hz, 1H), 4.25 (d, *J* = 3.5 Hz, 1H), 3.94 (d, *J* = 14.5 Hz, 1H), 3.26 (t, *J* = 13.2 Hz, 1H), 2.83 (d, *J* = 12.9 Hz, 1H), 2.41 (s, 3H), 1.94 (td, *J* = 13.2, 12.1, 4.1 Hz, 1H), 1.88 (d, *J* = 11.6 Hz, 1H), 1.12 (dt, *J* = 9.5, 6.8 Hz, 1H), 0.99 (dt, *J* = 9.6, 6.4 Hz, 1H), 0.58 (dt, *J* = 10.8, 5.7 Hz, 1H), 0.50 (dt, *J* = 9.7, 6.2 Hz, 1H). **^{13}C{^{1}H} NMR (126 MHz, CDCl$_3$)**) δ 194.8, 163.9, 143.3, 137.4, 136.2, 129.8 (2C), 126.9 (2C), 125.7, 124.0, 123.1, 118.9, 88.5, 79.1, 52.4, 43.3, 34.8, 30.5, 21.5, 12.9, 3.5. **IR (KBr, cm^{-1}):** 3130, 1712, 1618, 1401, 1293, 1156, 970, 767, 616. **IR (KBr, cm^{-1}):** 3130, 1712, 1618, 1401, 1293, 1156, 970, 767, 616. **HRMS (ESI – TOF)/m/z:** [M+H]$^+$ calcd for $C_{22}H_{23}N_2O_4S$ 411.1373; found 411.1374.

2'-tosyl-2',3',4',4a'-tetrahydro-1'H,11'H-spiro[cyclopropane-1,5'-[6,11a]epoxybenzo[b] pyrido[3,4-e]azepin]-11'-one (4ad'')

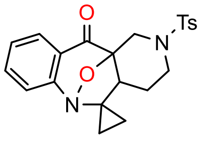

4ad''

Yield: 32% (20 mg), white solid, m.p. 183.4 – 185.1 °C, R_f = 0.3 (EtOAc/ petroleum ether = 1:8). **^{1}H NMR (500 MHz, CDCl$_3$)** δ 7.90 (d, *J* = 7.6 Hz, 1H), 7.66 (d, *J* = 8.1 Hz, 2H), 7.47 (t, *J* = 7.1 Hz, 1H), 7.29 (t, *J* = 7.6 Hz, 1H), 7.25 (d, *J* = 8.0 Hz, 2H), 6.91 (d, *J* = 7.8 Hz, 1H), 3.87 (d, *J* = 14.2 Hz, 1H), 3.77 (d, *J* = 14.2 Hz, 1H), 3.48 (ddd, *J* = 11.7, 8.7, 3.4 Hz, 1H), 3.09 (ddd, *J* = 11.4, 7.8, 3.4 Hz, 1H), 2.39 (t, *J* = 6.1 Hz, 1H), 2.36 (s, 3H), 1.79 – 1.69 (m, 1H), 1.59 – 1.55 (m, 1H), 1.15 (ddd, *J* = 11.1, 7.5, 5.6 Hz, 1H), 0.82 (dt, *J* = 10.7, 6.4 Hz, 1H), 0.75 (dt, *J* = 12.0, 5.9 Hz, 1H), 0.56 (dt, *J* = 9.8, 7.1 Hz, 1H). **^{13}C{^{1}H} NMR (126 MHz, CDCl$_3$)**) δ 192.5, 154.0, 143.5, 134.8, 134.6, 129.7 (2C), 127.7 (2C), 127.6, 127.4, 123.4, 122.9, 87.9, 54.3, 42.0, 40.6, 40.3, 24.1, 21.6, 12.4, 8.0. **HRMS (ESI – TOF)/m/z:** [M+H]$^+$ calcd for $C_{22}H_{23}N_2O_4S$ 411.1373; found 411.1371.

Procedure for preparation of 4ae'

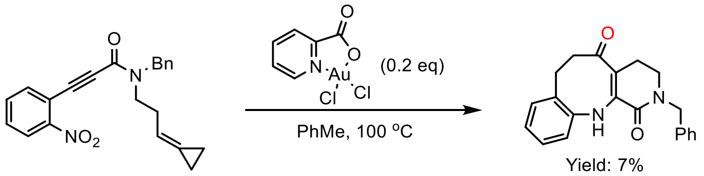

Yield: 7%

A round-bottom flask was charged with substrate **3ae** (1 eq, 0.35 mmol) in toluene (0.03 M). To this solution was added dichloro(2-pyridinecarboxylate)gold (0.2 eq, 0.07 mmol). The resulting mixture was

heated to 100 °C for 12 h. Subsequently, the reaction was filtered through a silica plug. After concentration in vacuo, the residue was purified via silica gel column chromatography (EtOAc/petroleum ether = 1:3).

2-benzyl-2,3,4,6,7,12-hexahydrobenzo[b]pyrido[4,3-g]azocine-1,5-dione (4ae')

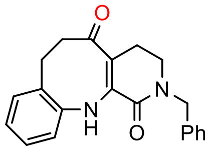

4ae'

Yield: 7% (8 mg), white solid, m.p. 101.6 – 102.8 °C, R_f = 0.3 (EtOAc/ petroleum ether = 1:3). **^{1}H NMR (500 MHz, $CDCl_3$)** δ 7.63 (s, 1H), 7.39 – 7.34 (m, 2H), 7.34 – 7.29 (m, 3H), 7.16 – 7.08 (m, 3H), 7.01 (d, *J* = 6.3 Hz, 1H), 4.72 (s, 2H), 3.35 (t, *J* = 7.0 Hz, 2H), 3.15 (t, *J* = 6.9 Hz, 2H), 2.91 (t, *J* = 6.9 Hz, 2H), 2.47 (t, *J* = 7.0 Hz, 2H). **^{13}C{^{1}H} NMR (126 MHz, $CDCl_3$)** δ 207.1, 163.0, 139.4, 136.3, 136.1, 135.2, 130.0, 128.8 (2C), 128.0 (2C), 127.9, 127.1, 126.0, 124.2, 111.6, 51.6, 44.8, 44.3, 26.8, 26.0. **IR (KBr, cm^{-1}):** 3129, 1638, 1619, 1400, 1294, 1092, 855, 766, 613. **HRMS (ESI – TOF)/m/z:** $[M+H]^+$ calcd for $C_{21}H_{21}N_2O_2$ 333.1598; found 333.1601.

Procedure for preparation of 5

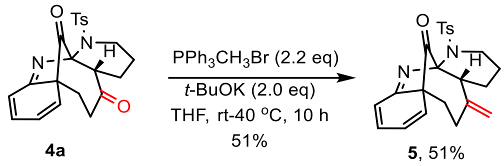

A suspension of Wittig reagent (2.2 eq, 0.22 mmol) in dry THF (2 ml) was prepared under N_2. Potassium tert-butoxide (2.0 eq, 1.0 M in THF, 0.2 mL) solution was added slowly to the suspension using a syringe pump over 5 minutes at 0 °C. Then the suspension was warmed to r.t. After 1 h, **4a** (1 eq, 0.1 mmol) in THF solution (1 mL) was added slowly to the suspension using a syringe pump over 5 minutes. After reacting at room temperature for 1h, the reaction was removed to 40 °C oil bath. The reaction was monitored by TLC until completion (10 h). The mixture was filtrated through a pad of silica gel. The organic phases were concentrated in vacuo and purified over silica gel (petroleum ether/EtOAc = 4:1, 51% yield) to afford **5** (21 mg, 51% yield), white solid, m.p. 116.4 – 117.5 °C. R_f = 0.3 (EtOAc/ petroleum ether = 1:4). **^{1}H NMR (500 MHz, $CDCl_3$)** δ 7.70 (d, *J* = 8.1 Hz, 2H), 7.25 (d, *J* = 7.7 Hz, 2H), 6.66 (dd, *J* = 9.6, 5.5 Hz, 1H), 6.47 (t, *J* = 9.8 Hz, 2H), 6.21 (dd, *J* = 9.2, 5.6 Hz, 1H), 4.94 (s, 1H), 4.82 (s, 1H), 3.35 (d, *J* = 11.3 Hz, 1H), 3.26 (t, *J* = 10.7 Hz, 1H), 2.59 (dd, *J* = 10.5, 5.4 Hz, 1H), 2.40 (s, 3H), 2.38 – 2.32 (m, 1H), 2.14 – 2.04 (m, 1H), 1.94 (dt, *J* = 12.9, 3.1 Hz, 1H), 1.88 – 1.79 (m, 2H), 1.77 – 1.70 (m, 2H), 1.64 – 1.56 (m, 1H). **^{13}C{^{1}H} NMR (126 MHz, $CDCl_3$)** δ 210.8, 179.3, 144.6, 143.7, 135.7, 135.0, 133.8, 129.3 (2C), 128.6 (2C), 121.8, 121.4, 116.6, 87.1, 58.8, 51.0, 44.5, 34.1, 33.3, 25.0, 24.1, 21.6. **IR (KBr, cm^{-1}):** 3694, 2956, 1849, 1733, 1588, 1490, 1335, 1161, 968, 811, 661. **HRMS(ESI-TOF)/m/z:** $[M+H]^+$ calcd for $C_{23}H_{25}N_2O_3S$ 409.1580; found 409.1577.

Procedure for preparation of 6

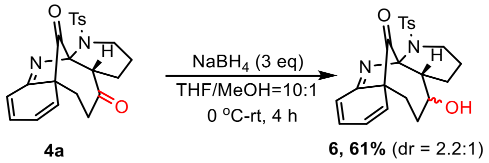

To a 25 mL Schlenk tube with a magnetic bar were added **4a** (1.0 eq, 0.1 mmol), the mixed solvent (THF/ MeOH = 10:1, 1 mL) and $NaBH_4$ (3 eq, 0.3 mmol) at 0 °C. Then the reaction was warmed to r.t. The reaction was monitored by TLC until completion (4h). The mixture was washed with water (3 mL),

extracted with EtOAc (3*5 mL), dried over Na_2SO_4 and concentrated in vacuo. The crude product was purified by column chromatography (silica gel, EtOAc/petroleum ether = 2:1) to afford **6** as white solid (25 mg, 61% yield, two isomers, ratio ≈ 2.2 :1), m.p. 180.5 – 182.8 °C. **^{1}H NMR (500 MHz, $CDCl_3$)** δ 7.88 (d, J = 8.2 Hz, 2H, minor), 7.68 (d, J = 8.1 Hz, 2H(*2.2), major), 7.28 (d, J = 8.5 Hz, 2H, minor), 7.26 (d, J = 7.9 Hz, 2H(*2.2), major), 6.74 (dd, J = 9.3, 5.3 Hz, 1H(*2.2), major), 6.54 (d, J = 9.2 Hz, 1H(*2.2), major), 6.51 (d, J = 9.7 Hz, 1H(*2.2), major), 6.25 (dd, J = 9.2, 5.6 Hz, 1H(*2.2), major), 6.07 (dd, J = 9.7, 5.4 Hz, 1H, minor), 6.00 (dd, J = 9.3, 5.5 Hz, 1H, minor), 5.85 (d, J = 9.3 Hz, 1H, minor), 5.64 (d, J = 9.7 Hz, 1H, minor), 4.01 (d, J = 9.3 Hz, 1H, minor), 3.89 (d, J = 4.7 Hz, 1H(*2.2), major), 3.34 (dd, J = 8.3, 3.6 Hz, 1H, minor), 3.28 (d, J = 9.7 Hz, 1H(*2.2), major), 3.22 (t, J = 11.4 Hz, 1H(*2.2), major), 3.12 (td, J = 12.5, 3.2 Hz, 1H, minor), 2.41 (s, 3H, minor), 2.40 (s, 3H(*2.2), major), 2.19 – 1.19 (m, 10H, minor and 10H(*2.2), major). **^{13}C{^{1}H} NMR (126 MHz, $CDCl_3$)** δ 209.2, 209.1, 182.7, 143.9, 143.7, 136.9, 136.0, 135.5, 134.5, 129.3, 129.3 (2C), 128.5 (2C), 128.3, 127.2, 124.7, 123.7, 121.6, 121.4, 121.2, 88.6, 88.0, 74.0, 73.5, 72.7, 58.7, 52.9, 51.5, 44.3, 44.2, 43.8, 31.8, 29.7, 28.4, 27.3, 24.2, 24.13, 24.06, 24.0, 22.5, 21.6. **IR (KBr, cm^{-1}):** 2929, 1768, 1585, 1490, 1444, 1330, 1156, 1087, 904, 744, 692, 582, 540. **HRMS (ESI-TOF) /m/z:** $[M+H]^+$ calcd for $C_{22}H_{25}N_2O_4S$ 413.1530; found 413.1529.

Procedure for preparation of 7

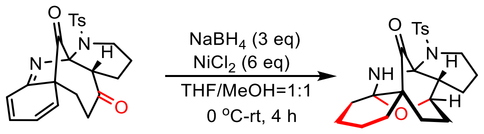

4a **7**, 68%

To a 25 mL Schlenk tube with a magnetic bar were added **4a** (1.0 eq, 0.11 mmol), the mixed solvent (THF/ MeOH = 1:1, 1.1 mL), $NaBH_4$ (3 eq, 0.33 mmol) and $NiCl_2$ (6 eq) at 0 °C. Then the suspension was warmed to r.t. The reaction was monitored by TLC until completion (4h). The reaction mixture was washed with water (3 mL), extracted with EtOAc (3*3 mL), dried over Na_2SO_4 and concentrated in vacuo. The crude product was purified by column chromatography (silica gel, petroleum ether/EtOAc = 2:1) to afford **7** as white solid (31.4 mg, 68% yield), white solid, m.p. 155.5 – 156.1 °C. R_f = 0.3 (EtOAc/ petroleum ether = 1:2). **^{1}H NMR (500 MHz, $CDCl_3$)** δ 7.94 (d, *J* = 8.2 Hz, 2H), 7.28 (d, *J* = 8.2 Hz, 2H), 3.92 (d, *J* = 9.3 Hz, 1H), 3.27 (d, *J* = 11.9 Hz, 1H), 2.89 (td, *J* = 12.5, 2.9 Hz, 1H), 2.41 (s, 3H), 2.18 (dddd, *J* = 13.4, 9.5, 6.5, 3.3 Hz, 1H), 2.09 (td, *J* = 13.9, 3.8 Hz, 1H), 1.93 (d, *J* = 13.8 Hz, 1H), 1.87 – 1.68 (m, 5H), 1.67 – 1.60 (m, 5H), 1.59 – 1.51 (m, 1H), 1.50 – 1.40 (m, 2H), 1.34 – 1.26 (m, 1H), 1.11 (dd, *J* = 13.2, 3.8 Hz, 1H). **^{13}C{^{1}H} NMR (126 MHz, $CDCl_3$)** δ 210.8, 143.5, 136.7, 129.2 (2C), 128.6 (2C), 89.3, 77.1, 73.3, 48.6, 44.2, 42.9, 33.1, 30.4, 28.7, 24.0, 23.9, 22.9, 21.8, 21.8, 21.6. **IR (KBr, cm^{-1}):** 3770, 3077, 1849, 1718, 1540, 1476, 1269, 1159, 961, 755, 661. **HRMS(ESI-TOF)/m/z:** $[M+H]^+$ calcd for $C_{22}H_{29}N_2O_4S$ 417.1843; found 417.1839.

Procedure for preparation of 8

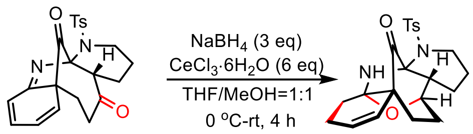

4a **8**, 95%

To a 25 mL Schlenk tube with a magnetic bar were added **4a** (1.0 eq, 0.1 mmol), the mixed solvent (THF/ MeOH = 1:1, 1 mL), $NaBH_4$ (3 eq, 0.3 eq) and $CeCl_3•6H_2O$ (6 eq) at 0 °C. Then the suspension

was warmed to r.t. The reaction was monitored by TLC until completion (4h). After the reaction was finished, the mixture was washed with water (3 mL), extracted with EtOAc (3 * 3 mL), dried over Na_2SO_4, and concentrated in vacuo. The crude product was purified by column chromatography (silica gel, petroleum ether/EtOAc = 2:1) to afford **8** as white solid (40 mg, 95% yield), m.p. 200.3 – 202.7 °C. R_f = 0.3 (EtOAc/ petroleum ether = 1:2). **^{1}H NMR (500 MHz, $CDCl_3$)** δ 7.80 (d, *J* = 8.2 Hz, 2H), 7.20 (d, *J* = 8.1 Hz, 2H), 5.82 (dd, *J* = 9.5, 4.9 Hz, 1H), 5.55 (dd, *J* = 9.7, 2.2 Hz, 1H), 3.92 (d, *J* = 9.3 Hz, 1H), 3.25 (dt, *J* = 11.8, 3.3 Hz, 1H), 2.85 (td, *J* = 12.4, 3.2 Hz, 1H), 2.34 (s, 3H), 2.28 (dddt, *J* = 17.9, 10.3, 5.4, 2.5 Hz, 1H), 2.13 (dt, *J* = 13.6, 9.0 Hz, 1H), 2.05 (dt, *J* = 17.5, 5.6 Hz, 1H), 1.88 (dd, *J* = 12.7, 8.4 Hz, 1H), 1.81 (dt, *J* = 13.1, 3.3 Hz, 2H), 1.78 – 1.69 (m, 2H), 1.68 – 1.59 (m, 2H), 1.45 (tt, *J* = 12.9, 4.0 Hz, 1H), 1.40 – 1.34 (m, 1H), 1.33 – 1.25 (m, 1H), 1.13 (dd, *J* = 13.2, 4.0 Hz, 1H). **^{13}C{^{1}H} NMR (126 MHz, $CDCl_3$)** δ 207.0, 142.5, 135.6, 128.1(2C), 127.5(2C), 127.1, 124.2, 88.2, 75.6, 72.7, 50.8, 43.2, 42.4, 27.6, 27.1, 23.9, 23.0, 22.9, 21.2, 20.5. **IR (KBr, cm^{-1}):** 3305, 3031, 2935, 1766, 1588, 1454, 1265, 1091, 962, 879, 746, 622. **HRMS(ESI-TOF)/m/z:** $[M+H]^+$ calcd for $C_{22}H_{27}N_2O_4S$ 415.1686; found 415.1683.

Procedure for preparation of 9

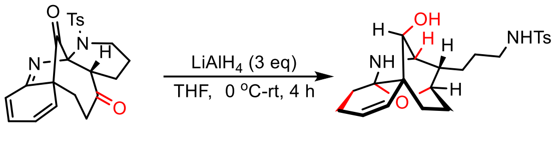

To a 25 mL Schlenk tube with a magnetic bar were added **4a** (1.0 eq, 0.1 mmol) and $LiAlH_4$ (3.0 eq, 0.3 mmol) in THF (0.1 M) at 0 °C. Then the suspension was warmed to r.t. After the reaction was finished, the mixture was washed with water at 0 °C (2 mL, must be carefully), extracted with EtOAc (3 * 3 mL), dried over Na_2SO_4 and concentrated in vacuo. The crude product was purified by column chromatography (silica gel, petroleum ether/THF = 1:1) to afford **9** as white solid (33 mg, 80% yield), m.p. 178.8 –180.1 °C. **^{1}H NMR (500 MHz, THF-d^8)** δ 7.70 (d, J = 7.9 Hz, 2H), 7.31 (d, J = 7.8 Hz, 2H), 6.44 (t, J = 5.4 Hz, 1H), 5.56 – 5.51 (m, 1H), 5.45 (dd, J = 9.3, 5.8 Hz, 1H), 4.51 (d, J = 3.9 Hz, 1H), 3.90 (t, J = 4.2 Hz, 1H), 3.71 (d, J = 9.0 Hz, 1H), 2.96 (d, J = 4.5 Hz, 1H), 2.86 (q, J = 5.7, 5.0 Hz, 2H), 2.39 (s, 3H), 2.08 (t, J = 15.4 Hz, 2H), 1.93 – 1.85 (m, 2H), 1.80 – 1.76 (m, 2H), 1.67 – 1.62 (m, 1H), 1.58 – 1.53 (m, 2H), 1.49 – 1.41 (m, 3H), 1.18 (dt, J = 12.6, 9.5 Hz, 1H), 0.89 (t, J = 6.4 Hz, 1H). **^{13}C{^{1}H} NMR (126 MHz, THF)** δ 142.2, 139.0, 134.9, 129.1 (2C), 126.8 (2C), 122.7, 91.0, 79.8, 72.2, 58.1, 44.8, 43.1, 38.1 30.6, 28.4, 28.4, 28.33, 23.5, 22.0, 20.4. **IR (KBr, cm^{-1}):** 2924, 1742, 1707, 1547, 1531, 1516, 1484, 1315, 1154, 1093, 808, 730, 666, 553, 515, 447. **HRMS (ESI-TOF)/m/z:** $[M+H]^+$ calcd for $C_{22}H_{31}N_2O_4S$ 419.1999; found 419.1998.

Procedure for preparation of 10

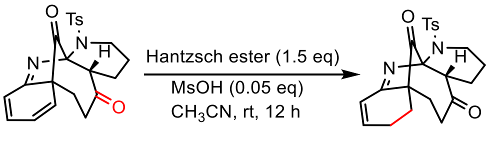

To a 25 mL Schlenk tube with a magnetic bar were added **4a** (1.0 eq, 0.1 mmol), Hantzsch ester (1.5 eq, 0.15 mmol) and MsOH (0.05 eq, 0.005 mmol) in CH_3CN (0.1M) at r.t. After the reaction was finished, the reaction mixture was concentrated in vacuo. The crude product was purified by column chromatography (silica gel, petroleum ether/EtOAc = 2:1) to afford **10** as yellow oil (37 mg, 91% yield), R_f

= 0.3 (EtOAc/ petroleum ether = 1:5). **^{1}H NMR (500 MHz, $CDCl_3$)** δ 7.74 (d, J = 8.1 Hz, 2H), 7.22 (d, J = 8.2 Hz, 2H), 5.84 – 5.74 (m, 2H), 3.30 (t, J = 11.4 Hz, 1H), 3.16 (d, J = 11.5 Hz, 1H), 2.76 (dd, J = 12.4, 3.7 Hz, 1H), 2.63 – 2.53 (m, 3H), 2.51 – 2.38 (m, 2H), 2.37 – 2.30 (m, 4H), 1.95 (ddd, J = 13.2, 7.2, 2.7 Hz, 1H), 1.86 – 1.76 (m, 2H), 1.75 – 1.65 (m, 2H), 1.48 – 1.36 (m, 1H). **^{13}C{^{1}H} NMR (126 MHz, $CDCl_3$)** δ 211.2, 205.3, 182.7, 144.0, 135.8, 129.4 (2C), 128.7, 128.3 (2C), 125.5, 85.3, 58.5, 56.6, 44.3, 40.7, 31.2, 27.2, 26.3, 23.0, 21.6, 20.3. **IR (KBr, cm^{-1}):** 3669, 2953, 1769, 1636, 1540, 1332, 1201, 1014, 904, 814, 661. **HRMS(ESI-TOF)/m/z:** $[M+Na]^+$ calcd for $C_{22}H_{24}N_2O_4SNa$ 435.1349; found 435.1341.

Procedure for preparation of 11

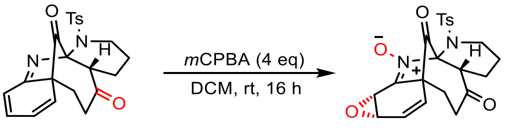

4a **11**, 61%

To a 25 mL Schlenk tube with a magnetic bar were added **4a** (1.0 eq, 0.1 mmol) and *m*-CPBA (4 eq, 0.4 mmol) in DCM (0.1 M) at r.t. After the reaction was finished, the reaction mixture was washed with water (3 mL), extracted with DCM (3*3 mL), dried over Na_2SO_4 and concentrated in vacuo. The crude product was purified by column chromatography (silica gel, petroleum ether/EtOAc = 1:1) to afford **11** as white solid (28 mg, 61% yield), m.p. 160.6 – 161.4 °C, R_f = 0.3 (EtOAc/ petroleum ether = 1:1). **^{1}H NMR (500 MHz, $CDCl_3$)** δ 7.81 (d, *J* = 8.2 Hz, 2H), 7.33 (d, *J* = 8.0 Hz, 2H), 6.30 (d, *J* = 9.6 Hz, 1H), 6.16 (dd, *J* = 9.6, 3.8 Hz, 1H), 4.70 (d, *J* = 3.9 Hz, 1H), 4.03 (t, *J* = 11.9 Hz, 1H), 3.73 (s, 1H), 3.26 (d, *J* = 11.1 Hz, 1H), 2.88 (dd, *J* = 12.9, 3.6 Hz, 1H), 2.70 – 2.58 (m, 2H), 2.44 (s, 3H), 2.37 – 2.26 (m, 2H), 2.14 – 2.08 (m, 1H), 1.98 (d, *J* = 13.5 Hz, 1H), 1.82 – 1.77 (m, 1H), 1.51 – 1.40 (m, 1H). **^{13}C{^{1}H} NMR (101 MHz, $CDCl_3$)** δ 203.7, 201.5, 144.8, 140.4, 134.7, 131.2, 129.6 (2C), 128.4 (2C), 124.6, 84.6, 57.6, 56.2, 51.5, 49.6, 44.6, 40.9, 34.9, 22.3, 21.6, 19.5. **IR (KBr, cm^{-1}):** 3049, 2962, 1811, 1718, 1589, 1489, 1195, 1088, 955, 862, 755, 663, 584. $[M+H]^+$ calcd for $C_{22}H_{23}N_2O_6S^+$ 443.1271; found 443.1265.

Procedure for preparation of 12

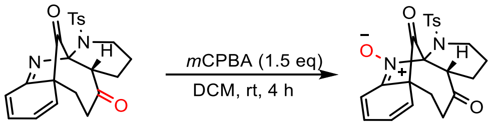

4a **12**, 71%

To a 25 mL Schlenk tube with a magnetic bar were added **4a** (1.0 eq, 0.1 mmol) and *m*-CPBA (1.5 eq, 0.15 mmol) in DCM (0.1 M) at r.t. The reaction mixture was stirred at room temperature and monitored by TLC. After the reaction was finished, the reaction mixture was washed with water (3 mL), extracted with DCM (3 * 3 mL), dried over Na_2SO_4 and concentrated in vacuo. The crude product was purified by column chromatography (silica gel, EtOAc/petroleum ether =1:1) to afford **12** as green solid (30 mg, 71% yield), m.p. 162.6 – 163.6 °C, R_f = 0.3 (EtOAc/ petroleum ether = 1:1). **^{1}H NMR (500 MHz, $CDCl_3$)** δ 7.69 (d, J = 8.1 Hz, 2H), 7.28 (d, J = 8.1 Hz, 2H), 6.77 (d, J = 9.7 Hz, 1H), 6.49 – 6.40 (m, 2H), 6.24 (dd, J = 9.2, 5.6 Hz, 1H), 3.82 (t, J = 10.3 Hz, 1H), 3.33 (d, J = 7.9 Hz, 1H), 2.95 (dd, J = 12.9, 3.7 Hz, 1H), 2.68 (ddd, J = 12.4, 10.0, 7.4 Hz, 1H), 2.55 (dt, J = 12.5, 5.6 Hz, 1H), 2.4 (s, 3H), 2.34 (qd, J = 13.3, 3.3 Hz, 1H), 2.09 – 1.99 (m, 2H), 1.99 – 1.93 (m, 1H), 1.80 (dd, J = 10.3, 3.5 Hz, 1H), 1.54 (qt, J = 13.0, 4.3 Hz, 1H). **^{13}C{^{1}H} NMR (126 MHz, $CDCl_3$)** δ 204.0, 202.4, 144.7, 144.0, 134.0, 131.1, 129.5 (2C), 128.9, 128.5 (2C), 123.4, 113.7, 81.9, 57.0, 55.6, 45.2, 40.6, 33.5, 22.6, 21.6, 20.0. **IR (KBr, cm^{-1}):** 3695, 2957, 1718, 1542, 1339, 1200, 1088, 912, 755, 660, 552. H**RMS(ESI-TOF)/m/z:** $[M+Na]^+$ calcd for $C_{22}H_{22}N_2NaO_5S$ 449.1142; found 449.1139.

Procedure for preparation of 13

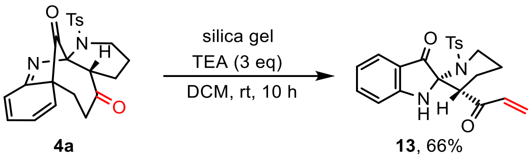

To a 25 mL Schlenk tube with a magnetic bar were added **4a** (1.0 eq, 0.14 mmol), the corresponding solvent DCM (0.1 M), silica gel (50 mg) and TEA (3 eq, 0.42 mmol) at r.t. The reaction mixture was stirred at room temperature. When the reaction was finished (10 h), the reaction mixture was filtrated through silica gel, washed with water (3 mL), extracted with DCM (3 * 3 mL), dried over Na_2SO_4 and concentrated in vacuo. The crude product was purified by column chromatography (silica gel, EtOAc/petroleum ether = 1:2) to afford **13** as pink solid (37 mg, 66% yield), m.p. 146.2 – 148.6 °C, R_f = 0.3 (EtOAc/ petroleum ether = 1:2). **^{1}H NMR (500 MHz, CDCl$_3$)** δ 7.73 (d, *J* = 8.1 Hz, 2H), 7.59 (d, *J* = 7.7 Hz, 1H), 7.44 (t, *J* = 7.6 Hz, 1H), 7.27 (d, *J* = 7.5 Hz, 2H), 6.88 (t, *J* = 7.4 Hz, 1H), 6.81 (d, *J* = 8.2 Hz, 1H), 6.12 (dd, *J* = 17.4, 9.8 Hz, 1H), 6.05 (d, *J* = 17.1 Hz, 1H), 5.64 (d, *J* = 9.9 Hz, 1H), 5.49 (s, 1H), 3.53 – 3.47 (m, 2H), 3.08 (td, *J* = 12.3, 2.5 Hz, 1H), 2.42 (s, 3H), 1.90 – 1.82 (m, 1H), 1.84 – 1.77 (m, 2H), 1.72 – 1.67 (m, 1H). **^{13}C{^{1}H} NMR (126 MHz, CDCl$_3$)** δ 198.3, 196.6, 158.2, 143.8, 137.5, 135.8, 135.7, 130.2, 129.3 (2C), 128.5 (2C), 124.8, 121.9, 120.8, 113.4, 77.52, 51.9, 44.4, 23.8, 23.3, 21.6. **IR (KBr, cm^{-1}):** 2924, 1714, 1617, 1416, 1315, 1197, 988, 863, 727, 665, 570. **HRMS (ESI-TOF)/m/z:** $[M+Na]^+$ calcd for $C_{22}H_{22}N_2O_4SNa$ 433.1192; found 433.1191.

Procedure for preparation of 14

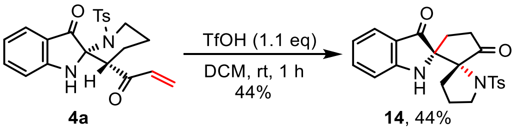

Method a: To a 25 mL Schlenk tube with a magnetic bar were added **4a** (1.0 eq, 0.1 mmol), the corresponding solvent DCM (0.1 M) and TfOH (1.1 eq, 0.11 mmol) at r.t. The reaction mixture was stirred at room temperature. After the reaction was finished (1h), the reaction mixture was concentrated in vacuo. The crude product was purified by column chromatography (silica gel, EtOAc/petroleum ether = 1:5) to afford **14** as green solid (18 mg, 44% yield). m.p. 159.6 – 162.0 °C. **^{1}H NMR (400 MHz, CDCl$_3$)** δ 7.77 (d, J = 8.3 Hz, 2H), 7.61 (d, J = 7.8 Hz, 1H), 7.53 (ddd, J = 8.3, 7.2, 1.3 Hz, 1H), 7.33 (s, 2H), 7.01 (d, J = 8.3 Hz, 1H), 6.85 (t, J = 7.4 Hz, 1H), 6.43 (s, 1H), 3.11 (td, J = 8.3, 2.0 Hz, 1H), 2.97 – 2.88 (m, 1H), 2.84 (d, J = 18.5 Hz, 1H), 2.87 – 2.76 (m, 1H), 2.71 (ddd, J = 18.6, 6.8, 5.4 Hz, 1H), 2.44 (s, 3H), 2.33 (dd, J = 10.0, 2.4 Hz, 1H), 2.32 (d, J = 9.8 Hz, 1H), 1.86 – 1.75 (m, 2H), 1.61 – 1.56 (m, 1H).. **^{13}C{^{1}H} NMR (126 MHz, CDCl$_3$)** δ 213.7, 201.3, 160.8, 144.1, 137.9, 134.7, 129.5 (2C), 128.5 (2C), 124.2, 119.9, 118.9, 112.6, 79.2, 74.6, 48.8, 35.4, 34.6, 29.7, 24.0, 21.6. **IR (KBr, cm^{-1}):** 2921, 1752, 1690, 1483, 1326, 1268, 1152, 754. **HRMS (ESI-TOF) /m/z:** $[M+Na]^+$ calcd for $C_{22}H_{22}N_2NaO_4S$ 433.1192; found 433.1186.

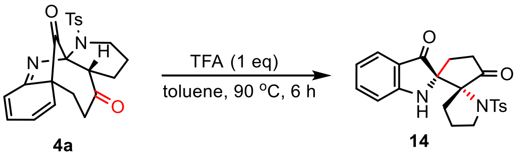

Method b: To a 25 mL Schlenk tube with a magnetic bar were added the solution of **4a** (1.0 eq, 0.1 mmol) in toluene (0.1 M) and TFA (1 eq, 0.1 mmol) at r.t. The reaction mixture was stirred at 90 °C for 6 h. After the reaction was finished (6 h), the reaction mixture was concentrated in vacuo. The crude product

was purified by column chromatography (silica gel, EtOAc/petroleum ether = 1:5) to afford **14** as green solid (31.5 mg, 77% yield).

Procedure for preparation of 15

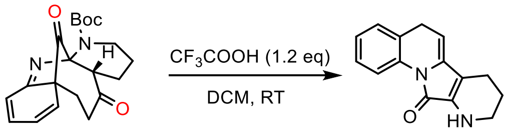

4aa **15** quant.

To a round bottow flask were added **4aa** (1.0 eq, 0.1 mmol) in 2 mL DCM, and CF_3COOH (1.2 eq, 0.12 mmol) under nitrogen atmosphere. The resulting mixture was stirred at rt for 5 h. After completion, the solvent was concentrated under reduced pressure to give a residue which is in good purity for NMR characterization. (Note: This product will gradually decompose at ambient temperature in air). R_f = 0.3 (EtOAc/ petroleum ether = 1:1). **^{1}H NMR (500 MHz, $CDCl_3$)** δ 8.73 (d, *J* = 8.3 Hz, 1H), 7.23 (t, *J* = 7.8 Hz, 1H), 7.15 (d, *J* = 7.4 Hz, 1H), 7.04 (t, *J* = 7.4 Hz, 1H), 5.39 (t, *J* = 4.3 Hz, 1H), 4.39 (brs, 1H), 3.64 (d, *J* = 4.2 Hz, 2H), 3.33 (t, *J* = 5.4 Hz, 2H), 2.45 (t, *J* = 6.2 Hz, 2H), 1.96 (p, *J* = 6.1 Hz, 2H). **^{13}C{^{1}H} NMR (126 MHz, $CDCl_3$)** δ 163.1, 137.2, 136.2, 134.9, 128.9, 127.3, 123.4, 122.1, 116.8, 108.7, 101.5, 41.6, 27.7, 21.6, 18.1. **^{19}F NMR (471 MHz, $CDCl_3$)** δ -75.9. **IR (KBr, cm^{-1}):** 2959, 2852, 1639, 1560, 1401, 1295, 1086, 763, 672, 649. **HRMS (ESI – TOF)/m/z:** $[M+Na]^+$ calcd for $C_{15}H_{14}N_2ONa$ 261.0998; found 261.0993.

Procedure for preparation of 16

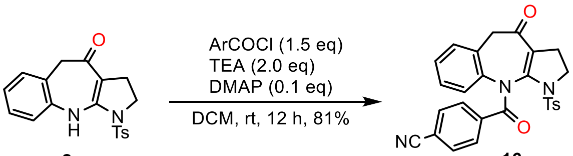

2a **16**

To a solution of amine **2a** (35 mg, 0.1 mmol), TEA (20 mg, 0.2 mmol) in DCM (1 mL) was added ArCOCl (1.5 eq, 0.15 mmol) and DMAP (0.1 eq, 0.01 mmol) at room temperature. The resulting mixture was stirred at room temperature overnight. The mixture was then transferred to flask and evaporated under reduced pressure. The residue was purified by silica gel column chromatography (EtOAc/petroleum ether = 1:5). Yield: 81% (28 mg), yellow oil, R_f = 0.4 (EtOAc/ petroleum ether = 1:5). **^{1}H NMR (500 MHz, $CDCl_3$)** δ 8.12 (d, *J* = 8.2 Hz, 2H), 8.02 (d, *J* = 8.1 Hz, 2H), 7.74 (d, *J* = 8.2 Hz, 2H), 7.30 (d, *J* = 8.0 Hz, 2H), 7.17 (t, *J* = 7.6 Hz, 1H), 7.09 (d, *J* = 7.4 Hz, 1H), 7.06 (d, *J* = 8.2 Hz, 1H), 6.95 (d, *J* = 7.4 Hz, 1H), 3.95 (t, *J* = 7.2 Hz, 2H), 3.44 (s, 2H), 2.57 (t, *J* = 7.0 Hz, 2H), 2.39 (s, 3H). **^{13}C{^{1}H} NMR (126 MHz, $CDCl_3$)** δ 161.9, 151.9, 148.7, 145.0, 144.6, 136.2, 132.6 (2C), 132.1, 130.7 (2C), 129.2 (2C), 129.1, 128.8 (2C), 127.7, 127.0, 126.5, 125.6, 118.5, 117.6, 117.6, 46.3, 37.0, 24.4, 21.7. **IR (KBr, cm^{-1}):** 3018, 2924, 1741, 1640, 1400, 1256, 1069, 1017, 860, 759, 673. **HRMS (ESI – TOF)/m/z:** $[M+H]^+$ calcd for $C_{27}H_{22}N_3O_4S$ 484.1326; found 484.1326.

Typical procedure for preparation of 17

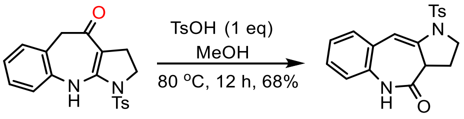

2a **17**

To a 25 mL Schlenk tube with a magnetic bar were added **2a** (1.0 eq, 0.1 mmol) and TsOH (1 eq, 0.1

mmol) in MeOH (0.1 M) at r.t. The mixture was stirred at 80 °C for 12 h. After the reaction was concentrated in vacuo, the crude product was purified by column chromatography (silica gel, petroleum ether/EtOAc = 1:1) to afford **17** as pink solid. Yield: 68% (24 mg), pink solid, m.p. 134.2 – 135.4 °C, R_f = 0.3 (EtOAc/ petroleum ether = 1:2). **^{1}H NMR (500 MHz, $CDCl_3$)** δ 8.03 (s, 1H), 7.69 (d, *J* = 8.1 Hz, 2H), 7.38 (d, *J* = 7.5 Hz, 1H), 7.24 – 7.16 (m, 4H), 6.94 (d, *J* = 8.0 Hz, 1H), 6.92 (s, 1H), 4.01 (t, *J* = 9.1 Hz, 1H), 3.68 (q, *J* = 9.6 Hz, 1H), 2.94 (d, *J* = 8.7 Hz, 1H), 2.66 (dd, *J* = 12.6, 7.5 Hz, 1H), 2.33 (s, 3H), 2.05 – 1.95 (m, 1H). **^{13}C{^{1}H} NMR (126 MHz, $CDCl_3$)** δ 169.4, 144.5, 137.8, 133.4, 133.3, 130.2, 129.9, 129.6 (2C), 127.4 (2C), 127.0, 124.6, 121.8, 105.2, 51.5, 46.0, 21.6, 21.1. **IR (KBr, cm^{-1}):** 3157, 1672, 1641, 1480, 1271, 1213, 1091, 977, 845, 762, 591. **HRMS (ESI – TOF)/m/z:** $[M+H]^+$ calcd for $C_{19}H_{19}N_2O_3S$ 355.1111; found 355.1107.

II. Supplementary Discussion

Control experiment

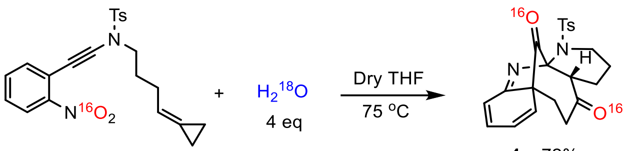

3a **4a**, 72%

Take the dried schlenk tube and perform five times of nitrogen exchange operations. Under a nitrogen atmosphere, **3a** (41 mg, 0.1 mmol) was dissolved in dry THF and added to the tube. $H_2{}^{18}O$ (4 eq, 0.4 mmol) was added to the solution. The solution was stirred under N_2 atmosphere at 75 °C for 12 h. After cooling to room temperature, the solvent was evaporated under reduced pressure and the resulting residue was purified by silica gel column chromatography (petroleum ether/EtOAc = 2:1) to give the cyclization product, which mixed the normal obtainted **4** (about 1 to 1) and then subject to ^{13}C-NMR analysis. The resulting spectrum is identical to that of **4**, indicating no ^{18}O is incorporated. In addition, the **HRMS** analysis of the cyclization product was also conducted, and no mass peak of the product labeled with ^{18}O is observed.

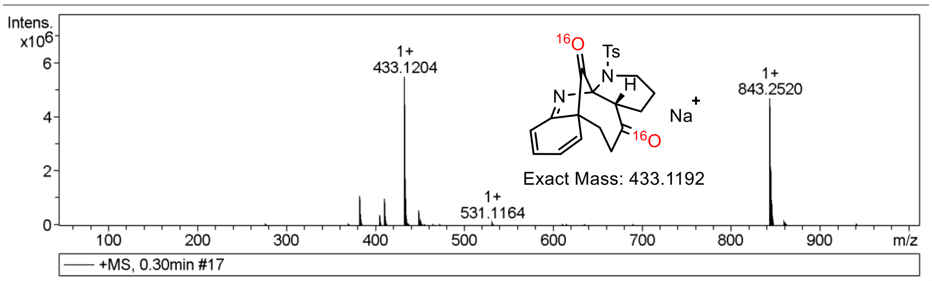

#	m/z	I	I %
1	383.1429	1105156	20.2
2	384.1452	242580	4.4
3	385.1417	63036	1.2
4	405.1242	390662	7.1
5	406.1268	93010	1.7
6	411.1378	988774	18.1
7	412.1399	232661	4.2
8	413.1369	61848	1.1
9	433.1204	5476135	100.0
10	434.1231	1206306	22.0
11	435.1200	298389	5.4
12	436.1202	55362	1.0
13	437.1924	55406	1.0
14	449.0934	572174	10.4
15	450.0958	143174	2.6
16	451.0929	75714	1.4

Supplementary Figure 1. HRMS analysis of the product

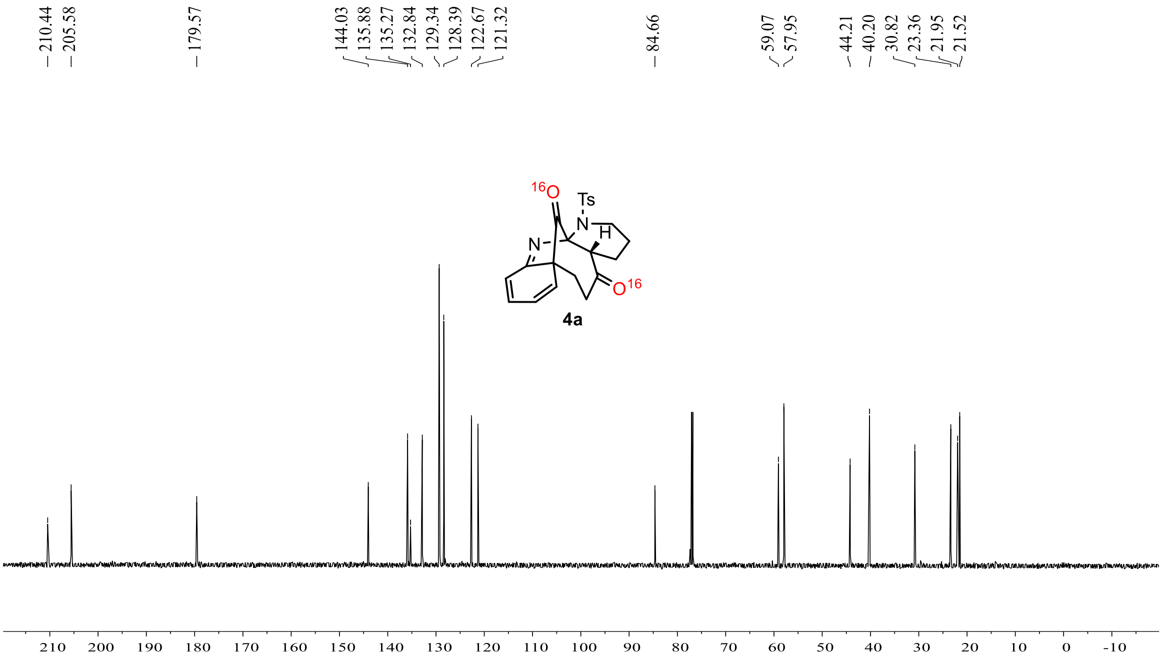

Supplementary Figure 2. ^{13}C-NMR spectroscopy of the product

In situ IR spectroscopy

A 25 ml double-necked round-bottomed flask was equipped with a magnetic stir bar. The two-neck bottle is filled with nitrogen by schlenk operation through a nitrogen balloon. The ReactIR DiComp probe was inserted and fitted to the side neck by a Teflon adapter. The starting material is dissolved in toluene and added to the flask via a syringe. ReactIR data collection was started (with an interval of 1 min). Put the flask into the oil bath and start to heat to the reaction temperature (it takes about 15 minutes). **1a** was heated at 90 ºC for 5 hours, and **3a** was heated at 75 ºC for 6.5 hours. Through the spectrum, we can clearly see the disappearance of the peak of triple bond of ynamide and the increase of intensity of the carbonyl group stretching vibration peaks.

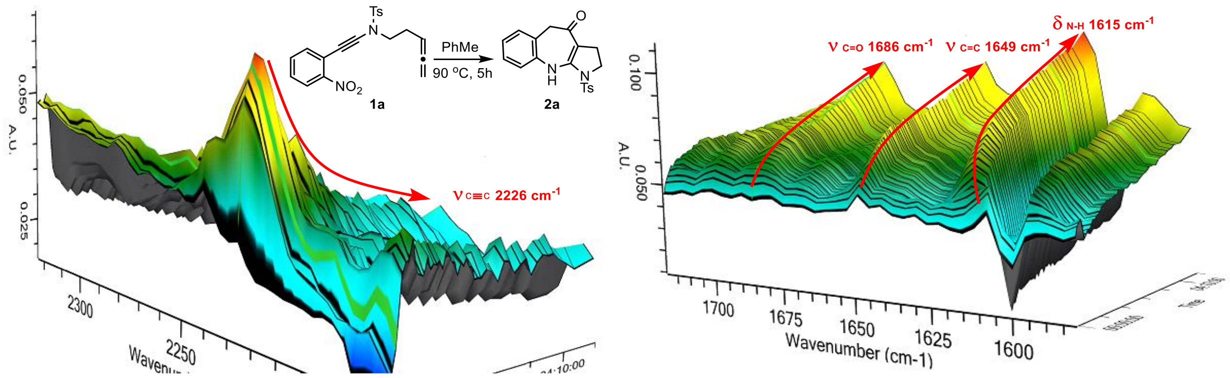

Supplementary Figure 3. In situ IR spectroscopy investigation reaction profiles of 1a

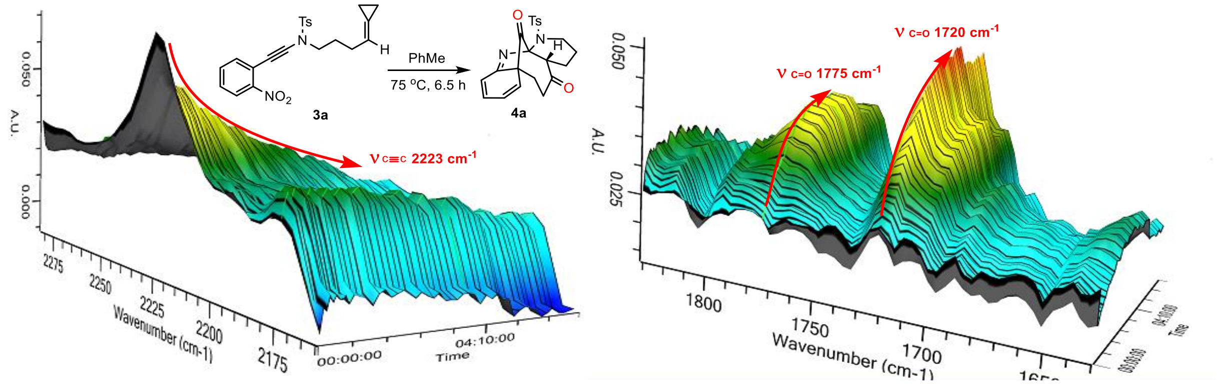

Supplementary Figure 4. In situ IR spectroscopy investigation reaction profiles of 3a

CO detection

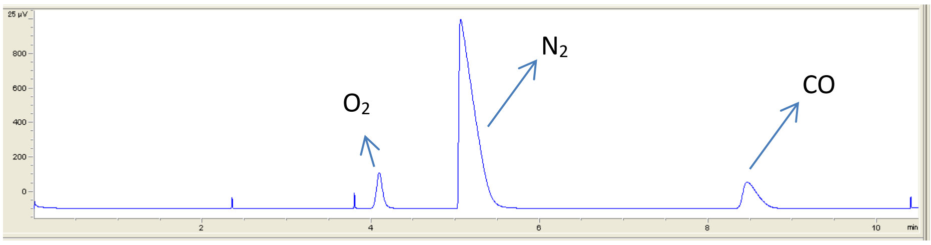

Supplementary Figure 5. GC detect the reaction generated CO

The reaction is carried out under a nitrogen atmosphere. After the reaction finished, the gas in the reaction system is sucked by a syringe and injected into the GC detector. GC type and method**: GC (Agilent 7890A), 99.999% Ar, Agilent G3591-80035 column, TCD Detector, 50 ℃.**

Biological activity tests

Anti-virus infectivity analysis

HEK293T (ATCC CRL-3216), SupT1 (ATCC CRL-1942) cells were maintained in Dulbecco's modified Eagle's medium (DMEM; Gibco) supplemented with 10% (v/v) fetal bovine serum (FBS; Gibco), at 37°C in a 5% CO_2 incubator. HEK293T-Gluc constitutively expresses the negative-strand RNA of the Gluc gene that is converted into positive-strand RNA upon IAV infection and expresses Gluc. Influenza A/WSN/1933 (H1N1) was generated using the pHW2000 eight-plasmid system. Single-round infectious IAV was produced with A/WSN/1933 by displacing the HA coding sequence with the Gaussia luciferase (Gluc) sequence. 293T-GLUC cells were inoculated in 96-well culture plates at 5% CO_2 and incubated at 37C for 24 hours. Pre-incubate with drug for 2 hours, then inoculate the virus at MOI=0.15 by diluting the original virus. After 24 hours of incubation, the luciferase activity in the infected cells was measured and the inhibition rate of each sample was calculated.

The vesicular stomatitis virus glycoprotein (VSV-G) expression vector pHIT/G, HIV-1 proviral indicator construct pNL-Luc-E- was provided by Dr. Johnny He. Confluent 293T cells (40–50%) in 3.5-cm dishes were cotransfected with 200 ng pNL-Luc-E-, 140 ng pSVCMV-VSV-G. After 20 h transfection, the supernatants were collected by filtration through a 0.45-mm filter for later experiments. SupT1 cells were inoculated in 96-well culture plates, incubated with drugs for 0.5 h in advance, and then inoculated with virus at MOI=0.01 and incubated in 5% CO2 for 48 h at 37℃. After 48 hours of incubation, the luciferase activity in the infected cells was measured using a firefly Luciferase Assay System (Promega).

Cytotoxicity analysis

HEK293T cells or SupT1 cells were cultured at 37°C in 96-well plates for 24 h and then incubated with the test compounds at the indicated concentrations. Mock-treated cells served as control. A cell viability assay was performed after 48 h using Cell Counting kit-8 (CCK-8, Beyotime, China).

Statistical analysis.

Data are presented as mean ± standard deviation (SD) from at least three independent experiments and evaluated via Prism (version 5.01, GraphPad Software, San Diego, CA, USA).

Supplementary Table 3. Effects of chemotype **2** compounds on HIV-1 inhibition

Compounds	Concentration	Cell viability %*	Inhibition %
**2a**	10μM	86.31±21.05	0.00
**2b**	10μM	72.52±8.8	21.58±11
**2c**	10μM	57.76±1.43	0.43±7
**2g**	10μM	70.08±8.43	2.72±1.8
**2m**	10μM	79.8±8.4	3.74±4.2
**2i**	10μM	80.31±1.24	0.00
**2j**	10μM	80.55±0.10	0.00
**2k**	10μM	79.74±0.36	0.00
**2l**	10μM	94.10±18.84	4±7.05
**2h**	10μM	98.00±0.01	0.00
**2n**	10μM	77.08±7.36	10.26±5.89
**2o**	10μM	91.74±2.03	33.60±17
**2p**	10μM	87.6±0.85	20.40±19
**2q**	10μM	69.56±3.17	0.00
**2r**	10μM	65.99±0.78	18.94±9.87
**2s**	10μM	92.85±7.41	21.53±5.8
**2u**	10μM	85.82±5.31	3.78±1.3
**4ac'**	10μM	87.01±4.93	0.00
**2t'**	10μM	100.90±19.04	4.15±6.53
**17**	10μM	100.64±2.92	0.00
**Nevirapine**	10μM	NT**	98.49±1.16

* In SupT1 cells; **NT means it was not tested

Supplementary Table 4. Effects of compounds on HIV-1 inhibition

Compounds	Concentration	Cell viability %*	Inhibition %
**4a**	10μM	107.93±2.85	-24.05±17.32
**4b**	10μM	87.28±20.49	-21.36±26.15
**4c**	10μM	58.63±2.23	31.33±0.26
**4e**	10μM	89.23±6.46	-48.03±17.05
**4h**	10μM	90.49±11.49	-39.26±15.89
**4i**	10μM	85.89±12.19	-24.21±6.07
**4j**	10μM	87.54±1.42	-42.16±11.8
**4l**	10μM	76.94±0.26	44.25±1.65
**4m**	10μM	95.11±4.91	5.37±3.15
**4n**	10μM	78.01±6.24	-69.08±15.15
**4p**	10μM	95.25±17.51	-83.75±1.84
**7**	10μM	94.95±9.50	-62.70±11.73
**8**	10μM	107.20±1.08	-28.92±18.52
**12**	10μM	92.36±5.73	-40.61±41.4
**13**	10μM	52.14±3.31	97.78±1.87
**Efavirenz**	50nM	NT**	97.57±0.76

* In SupT1 cells; **NT means it was not tested

Supplementary Table 5. Effects of compounds on IAV inhibition

Compounds	Concentration	Cell viability %*	Inhibition %
**4a**	10µM	97.79±0.74	92.09±2.60
**4b**	10µM	87.24±0.23	12.06±6.05
**4c**	10µM	88.19±0.77	-28.47±3.00
**4e**	10µM	71.46±0.39	-5.21±3.65
**4h**	10µM	84.96±1.11	-3.13±0.24
**4i**	10µM	82.36±1.40	8.63±7.84
**4j**	10µM	92.51±3.74	12.78±13.46
**4l**	10µM	88.50±0.77	3.01±16.5
**4m**	10µM	100.95±1.03	64.36±9.63
**4n**	10µM	96.24±8.17	-38.30±3.59
**4p**	10µM	86.75±1.66	-5.76±4.70
**7**	10µM	81.76±3.52	9.63±4.93
**8**	10µM	85.05±4.92	9.32±6.11
**12**	10µM	104.14±0.97	36.74±10.50
**13**	10µM	96.29±1.25	30.86±17.82
**Ribavirin**	50µM	NT**	98.65±1.15
**T-705**	10µM	NT**	92.29±6.28
**Oseltamivir**	20µM	NT**	90.90±1.72

* In 293T-GLUC cells; **NT means it was not tested

X-Ray diffraction analysis

Supplementary Table 6. Crystal data and structure refinement for 2k

The single crystal of **2k** was grown from DCM/PE by slow evaporation. A suitable crystal was selected and measured on an Agilent SuperNova, Dual, Cu at zero, AtlasS2 diffractometer. The crystal was kept at 100 K during data collection. The X-ray crystal structure of **2k** with thermal ellipsoids at the 50% probability level is draw as below.

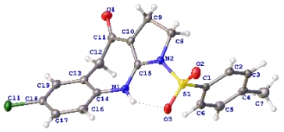

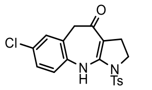

2k

Supplementary Figure 6: Ellipsoid plot of the crystal structure of **2k** (Prob = 50, Temp = 100 K)

CCDC	2092437
Empirical formula	$C_{19}H_{17}ClN_2O_3S$
Formula weight	388.85
Temperature/K	150.00(10)
Crystal system	Triclinic
Space group	P-1
a/Å	9.3976(5)
b/Å	11.0644(6)
c/Å	17.8600(11)
α/°	106.081(5)
β/°	96.616(5)
γ/°	97.925(5)
Volume/Å^3	1744.24(18)
Z	4
ρ_{calc}g/cm^3	1.481
μ/mm^{-1}	0.361
F(000)	808.0
Crystal size/mm^3	0.13 × 0.11 × 0.09
Radiation	Mo Kα (λ = 0.71073)
2Θ range for data collection/°	3.894 to 49.996
Index ranges	-11 ≤ h ≤ 9, -13 ≤ k ≤ 12, -20 ≤ l ≤ 21
Reflections collected	11843
Independent reflections	6138 [R_{int} = 0.0341, R_{sigma} = 0.0605]
Data/restraints/parameters	6138/0/479
Goodness-of-fit on F^2	1.041
Final R indexes [I>=2σ (I)]	R_1 = 0.0470, wR_2 = 0.1070
Final R indexes [all data]	R_1 = 0.0640, wR_2 = 0.1168
Largest diff. peak/hole / e Å^{-3}	0.36/-0.37

Supplementary Table 7. Crystal data and structure refinement for 2u

The single crystal of **2u** was grown from DCM/PE by slow evaporation. A suitable crystal was selected and measured on an Agilent SuperNova, Dual, Cu at zero, AtlasS2 diffractometer. The crystal was kept at 100 K during data collection.

The X-ray crystal structure of **2u** with thermal ellipsoids at the 50% probability level is draw as below.

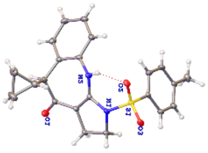

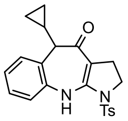

2u

Supplementary Figure 7: Ellipsoid plot of the crystal structure of **2u** (Prob = 50, Temp = 100 K)**.**

CCDC	2092438
Identification code	59-257
Empirical formula	$C_{22}H_{22}N_2O_3S$
Formula weight	394.47
Temperature/K	150.00(10)
Crystal system	monoclinic
Space group	$P2_1/c$
a/Å	21.0513(13)
b/Å	5.1267(2)
c/Å	19.2240(12)
α/°	90
β/°	112.817(7)
γ/°	90
Volume/Å^3	1912.4(2)
Z	4
ρ_{calc}g/cm^3	1.370
μ/mm^{-1}	1.719
F(000)	832.0
Crystal size/mm^3	0.14 × 0.1 × 0.08
Radiation	Cu Kα (λ = 1.54184)
2Θ range for data collection/°	4.554 to 147.582
Index ranges	-21 ≤ h ≤ 25, -4 ≤ k ≤ 6, -20 ≤ l ≤ 23
Reflections collected	6215
Independent reflections	3743 [R_{int} = 0.0378, R_{sigma} = 0.0500]
Data/restraints/parameters	3743/0/258
Goodness-of-fit on F^2	1.054
Final R indexes [I>=2σ (I)]	R_1 = 0.0605, wR_2 = 0.1615
Final R indexes [all data]	R_1 = 0.0688, wR_2 = 0.1715
Largest diff. peak/hole / e Å^{-3}	0.75/-0.63

Supplementary Table 8. Crystal data and structure refinement for 4a.

The single crystal of **4a** was grown from DCM/PE by slow evaporation. A suitable crystal was selected and measured on an Agilent SuperNova, Dual, Cu at zero, AtlasS2 diffractometer. The crystal was kept at 100 K during data collection.

The X-ray crystal structure of **4a** with thermal ellipsoids at the 50% probability level is draw as below.

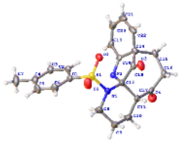

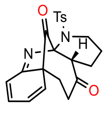

4a

Supplementary Figure 8: Ellipsoid plot of the crystal structure of **4a** (Prob = 50, Temp = 100 K)

CCDC	2092439
Empirical formula	$C_{22}H_{22}N_2O_4S$
Formula weight	410.47
Temperature/K	150.00(10)
Crystal system	monoclinic
Space group	$P2_1/n$
a/Å	8.2388(7)
b/Å	22.3529(17)
c/Å	10.9955(8)
α/°	90
β/°	104.887(9)
γ/°	90
Volume/Å^3	1957.0(3)
Z	4
ρ_{calc}g/cm^3	1.393
μ/mm^{-1}	0.198
F(000)	864.0
Crystal size/mm^3	0.13 × 0.12 × 0.1
Radiation	Mo Kα (λ = 0.71073)
2Θ range for data collection/°	4.244 to 49.992
Index ranges	-7 ≤ h ≤ 9, -26 ≤ k ≤ 26, -12 ≤ l ≤ 13
Reflections collected	7836
Independent reflections	3442 [R_{int} = 0.0329, R_{sigma} = 0.0501]
Data/restraints/parameters	3442/0/263
Goodness-of-fit on F^2	1.100
Final R indexes [I>=2σ (I)]	R_1 = 0.0517, wR_2 = 0.1047
Final R indexes [all data]	R_1 = 0.0698, wR_2 = 0.1131
Largest diff. peak/hole / e Å^{-3}	0.37/-0.40

Supplementary Table 9. Crystal data and structure refinement for 4u'.

The single crystal of **4u'** was grown from DCM/PE by slow evaporation. A suitable crystal was selected and measured on an Agilent SuperNova, Dual, Cu at zero, AtlasS2 diffractometer. The crystal was kept at 100 K during data collection.

The X-ray crystal structure of **4u'** with thermal ellipsoids at the 50% probability level is draw as below.

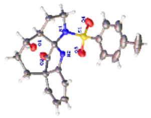

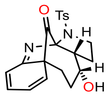

4u'

Supplementary Figure 9: Ellipsoid plot of the crystal structure of **4u'** (Prob = 50, Temp = 100 K)

CCDC	2092440
Empirical formula	$C_{21}H_{22}N_2O_4S$
Formula weight	398.46
Temperature/K	149.99(10)
Crystal system	tetragonal
Space group	P-42$_1$c
a/Å	17.1880(6)
b/Å	17.1880(6)
c/Å	13.0162(7)
α/°	90
β/°	90
γ/°	90
Volume/Å^3	3845.3(3)
Z	8
ρ_{calc}g/cm^3	1.377
μ/mm^{-1}	0.199
F(000)	1680.0
Crystal size/mm^3	0.12 × 0.11 × 0.09
Radiation	Mo Kα (λ = 0.71073)
2Θ range for data collection/°	4.74 to 49.994
Index ranges	-20 ≤ h ≤ 12, -13 ≤ k ≤ 20, -15 ≤ l ≤ 10
Reflections collected	9538
Independent reflections	3359 [R_{int} = 0.0343, R_{sigma} = 0.0406]
Data/restraints/parameters	3359/7/255
Goodness-of-fit on F^2	1.077
Final R indexes [I>=2σ (I)]	R_1 = 0.0514, wR_2 = 0.1328
Final R indexes [all data]	R_1 = 0.0595, wR_2 = 0.1389
Largest diff. peak/hole / e Å^{-3}	0.79/-0.26
Flack parameter	-0.04(5)

Supplementary Table 10. Crystal data and structure refinement for 4ad''.

The single crystal of **4ad''** was grown from DCM/PE by slow evaporation. A suitable crystal was selected and measured on an Agilent SuperNova, Dual, Cu at zero, AtlasS2 diffractometer. The crystal was kept at 100 K during data collection.

The X-ray crystal structure of **4ad''** with thermal ellipsoids at the 50% probability level is draw as below.

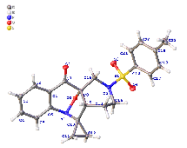

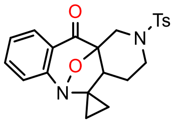

4ad''

Supplementary Figure 10: Ellipsoid plot of the crystal structure of **4ad''** (Prob = 50, Temp = 100 K)

CCDC	2131908
Empirical formula	$C_{22}H_{22}N_2O_4S$
Formula weight	410.47
Temperature/K	150.00(10)
Crystal system	monoclinic
Space group	$P2_1/c$
a/Å	20.2516(14)
b/Å	6.4641(7)
c/Å	14.7499(11)
α/°	90
β/°	93.171(7)
γ/°	90
Volume/Å^3	1927.9(3)
Z	4
ρ_{calc}g/cm^3	1.414
μ/mm^{-1}	0.201
F(000)	864.0
Crystal size/mm^3	0.15 × 0.13 × 0.1
Radiation	Mo Kα (λ = 0.71073)
2Θ range for data collection/°	4.028 to 50
Index ranges	-24 ≤ h ≤ 17, -5 ≤ k ≤ 7, -17 ≤ l ≤ 17
Reflections collected	8315
Independent reflections	3399 [R_{int} = 0.0415, R_{sigma} = 0.0614]
Data/restraints/parameters	3399/0/263
Goodness-of-fit on F^2	1.052
Final R indexes [I>=2σ (I)]	R_1 = 0.0476, wR_2 = 0.0966
Final R indexes [all data]	R_1 = 0.0682, wR_2 = 0.1082
Largest diff. peak/hole / e Å^{-3}	0.22/-0.35

Supplementary Table 11. Crystal data and structure refinement for 7.

The single crystal of **7** was grown from DCM/PE by slow evaporation. A suitable crystal was selected and measured on an Agilent SuperNova, Dual, Cu at zero, AtlasS2 diffractometer. The crystal was kept at 100 K during data collection.

The X-ray crystal structure of **7** with thermal ellipsoids at the 50% probability level is draw as below

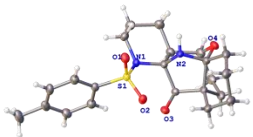

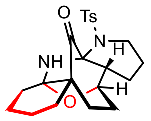

7

Supplementary Figure11: Ellipsoid plot of the crystal structure of **7** (Prob = 50, Temp = 100 K)

CCDC	2092441
Identification code	216-4
Empirical formula	$C_{22}H_{28}N_2O_4S$
Formula weight	416.52
Temperature/K	149.99(10)
Crystal system	orthorhombic
Space group	Pbcn
a/Å	10.1784(4)
b/Å	12.6371(4)
c/Å	31.5900(10)
α/°	90
β/°	90
γ/°	90
Volume/Å^3	4063.3(2)
Z	8
ρ_{calc}g/cm^3	1.362
μ/mm^{-1}	1.679
F(000)	1776.0
Crystal size/mm^3	0.14 × 0.12 × 0.08
Radiation	Cu Kα (λ = 1.54184)
2Θ range for data collection/°	5.596 to 148.172
Index ranges	-8 ≤ h ≤ 12, -14 ≤ k ≤ 15, -33 ≤ l ≤ 39
Reflections collected	15874
Independent reflections	4071 [R_{int} = 0.0536, R_{sigma} = 0.0392]
Data/restraints/parameters	4071/0/267
Goodness-of-fit on F^2	1.065
Final R indexes [I>=2σ (I)]	R_1 = 0.0457, wR_2 = 0.1202
Final R indexes [all data]	R_1 = 0.0536, wR_2 = 0.1283
Largest diff. peak/hole / e Å^{-3}	0.39/-0.54

Supplementary Table 12. Crystal data and structure refinement for 8

The single crystal of **8** was grown from DCM/PE by slow evaporation. A suitable crystal was selected and measured on an Agilent SuperNova, Dual, Cu at zero, AtlasS2 diffractometer. The crystal was kept at 100 K during data collection.

The X-ray crystal structure of **8** with thermal ellipsoids at the 50% probability level is draw as below

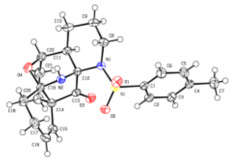

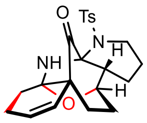

8

Supplementary Figure 12: Ellipsoid plot of the crystal structure of **8** (Prob = 50, Temp = 100 K)

CCDC	2092442
Identification code	224-3
Empirical formula	$C_{23}H_{28}Cl_2N_2O_4S$
Formula weight	499.43
Temperature/K	150.00(10)
Crystal system	monoclinic
Space group	$P2_1/n$
a/Å	12.8220(7)
b/Å	11.1087(6)
c/Å	16.5291(8)
α/°	90
β/°	96.509(5)
γ/°	90
Volume/Å^3	2339.2(2)
Z	4
ρ_{calc}g/cm^3	1.418
μ/mm^{-1}	0.400
F(000)	1048.0
Crystal size/mm^3	0.14 × 0.13 × 0.12
Radiation	Mo Kα (λ = 0.71073)
2Θ range for data collection/°	3.818 to 49.986
Index ranges	-15 ≤ h ≤ 14, -13 ≤ k ≤ 13, -16 ≤ l ≤ 19
Reflections collected	11150
Independent reflections	4125 [R_{int} = 0.0230, R_{sigma} = 0.0291]
Data/restraints/parameters	4125/0/294
Goodness-of-fit on F^2	1.042
Final R indexes [I>=2σ (I)]	R_1 = 0.0381, wR_2 = 0.0894
Final R indexes [all data]	R_1 = 0.0474, wR_2 = 0.0956
Largest diff. peak/hole / e Å^{-3}	0.54/-0.43

Supplementary Table 13. Crystal data and structure refinement for 9

The single crystal of **9** was grown from MeOH/THF//PE by slow evaporation. A suitable crystal was selected and measured on an Agilent SuperNova, Dual, Cu at zero, AtlasS2 diffractometer. The crystal was kept at 100 K during data collection.

The X-ray crystal structure of **9** with thermal ellipsoids at the 50% probability level is draw as below

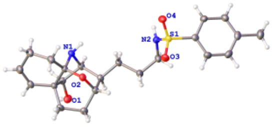

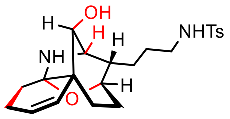

9

Supplementary Table 13: Ellipsoid plot of the crystal structure of **9** (Prob = 50, Temp = 100 K)

CCDC	2092443
Identification code	204
Empirical formula	$C_{22}H_{30}N_2O_4S$
Formula weight	418.54
Temperature/K	149.99(10)
Crystal system	monoclinic
Space group	$P2_1/c$
a/Å	11.6009(5)
b/Å	6.7441(3)
c/Å	27.2491(12)
α/°	90
β/°	92.931(4)
γ/°	90
Volume/Å^3	2129.12(16)
Z	4
ρ_{calc}g/cm^3	1.306
μ/mm^{-1}	1.602
F(000)	896.0
Crystal size/mm^3	0.13 × 0.12 × 0.09
Radiation	Cu Kα (λ = 1.54184)
2Θ range for data collection/°	6.496 to 147.908
Index ranges	-14 ≤ h ≤ 14, -8 ≤ k ≤ 8, -1 ≤ l ≤ 33
Reflections collected	4177
Independent reflections	4177 [R_{int} = 0.0467, R_{sigma} = 0.0607]
Data/restraints/parameters	4177/0/273
Goodness-of-fit on F^2	1.040
Final R indexes [I>=2σ (I)]	R_1 = 0.0717, wR_2 = 0.1684
Final R indexes [all data]	R_1 = 0.0813, wR_2 = 0.1733
Largest diff. peak/hole / e Å^{-3}	0.32/-0.46

Supplementary Table 14. Crystal data and structure refinement for 11

The single crystal of **11** was grown from DCM/PE by slow evaporation. A suitable crystal was selected and measured on an Agilent SuperNova, Dual, Cu at zero, AtlasS2 diffractometer. The crystal was kept at 100 K during data collection.

The X-ray crystal structure of **11** with thermal ellipsoids at the 50% probability level is draw as below

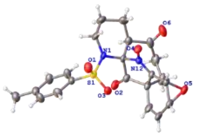

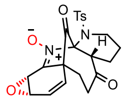

11

Supplementary Figure 14: Ellipsoid plot of the crystal structure of **11** (Prob = 50, Temp = 100 K)

CCDC	2092444
Empirical formula	$C_{22}H_{22}N_2O_6S$
Formula weight	442.47
Temperature/K	149.99(10)
Crystal system	monoclinic
Space group	Cc
a/Å	7.8695(5)
b/Å	32.157(2)
c/Å	8.0698(5)
α/°	90
β/°	103.156(7)
γ/°	90
Volume/Å^3	1988.6(2)
Z	4
ρ_{calc}g/cm^3	1.478
μ/mm^{-1}	1.837
F(000)	928.0
Crystal size/mm^3	0.14 × 0.12 × 0.11
Radiation	Cu Kα (λ = 1.54184)
2Θ range for data collection/°	5.496 to 146.69
Index ranges	-9 ≤ h ≤ 9, -37 ≤ k ≤ 39, -6 ≤ l ≤ 9
Reflections collected	3667
Independent reflections	2369 [R_{int} = 0.0368, R_{sigma} = 0.0503]
Data/restraints/parameters	2369/2/281
Goodness-of-fit on F^2	1.053
Final R indexes [I>=2σ (I)]	R_1 = 0.0438, wR_2 = 0.1179
Final R indexes [all data]	R_1 = 0.0477, wR_2 = 0.1215
Largest diff. peak/hole / e Å^{-3}	0.40/-0.32
Flack parameter	0.5

Supplementary Table 15. Crystal data and structure refinement for 14

The single crystal of **14** was grown from DCM/PE by slow evaporation. A suitable crystal was selected and measured on an Agilent SuperNova, Dual, Cu at zero, AtlasS2 diffractometer. The crystal was kept at 100 K during data collection.

The X-ray crystal structure of **14** with thermal ellipsoids at the 50% probability level is draw as below

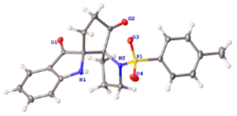

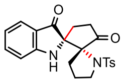

14

Supplementary Figure 15: Ellipsoid plot of the crystal structure of **14** (Prob = 50, Temp = 100 K)

CCDC	2092445
Identification code	222-2
Empirical formula	$C_{22}H_{22}N_2O_4S$
Formula weight	410.47
Temperature/K	150.00(10)
Crystal system	monoclinic
Space group	C2/c
a/Å	19.6571(19)
b/Å	13.9479(15)
c/Å	14.2549(14)
α/°	90
β/°	93.948(9)
γ/°	90
Volume/Å^3	3899.1(7)
Z	8
ρ_{calc}g/cm^3	1.399
μ/mm^{-1}	0.199
F(000)	1728.0
Crystal size/mm^3	0.13 × 0.11 × 0.09
Radiation	Mo Kα (λ = 0.71073)
2Θ range for data collection/°	4.154 to 49.992
Index ranges	-23 ≤ h ≤ 22, -13 ≤ k ≤ 16, -16 ≤ l ≤ 16
Reflections collected	8323
Independent reflections	3441 [R_{int} = 0.0540, R_{sigma} = 0.0693]
Data/restraints/parameters	3441/135/332
Goodness-of-fit on F^2	1.046
Final R indexes [I>=2σ (I)]	R_1 = 0.0586, wR_2 = 0.1417
Final R indexes [all data]	R_1 = 0.0760, wR_2 = 0.1558
Largest diff. peak/hole / e Å^{-3}	0.45/-0.46

Supplementary Table 16. Crystal data and structure refinement for 15

The single crystal of **15** was grown from DCM/PE by slow evaporation. A suitable crystal was selected and measured on an Agilent SuperNova, Dual, Cu at zero, AtlasS2 diffractometer. The crystal was kept at 100 K during data collection.

The X-ray crystal structure of **15** with thermal ellipsoids at the 50% probability level is draw as below

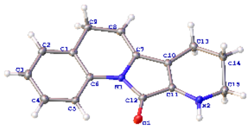

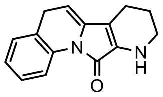

15

Supplementary Figure 16: Ellipsoid plot of the crystal structure of **15** (Prob = 50, Temp = 100 K)

CCDC	2131952
Empirical formula	$C_{15}H_{14}N_2O$
Formula weight	238.28
Temperature/K	150.00(10)
Crystal system	monoclinic
Space group	C2/c
a/Å	10.2386(8)
b/Å	19.8233(13)
c/Å	11.9562(9)
α/°	90
β/°	104.097(8)
γ/°	90
Volume/Å^3	2353.6(3)
Z	8
ρ_{calc}g/cm^3	1.345
μ/mm^{-1}	0.086
F(000)	1008.0
Crystal size/mm^3	0.14 × 0.13 × 0.12
Radiation	Mo Kα (λ = 0.71073)
2Θ range for data collection/°	4.588 to 59.258
Index ranges	-13 ≤ h ≤ 13, -26 ≤ k ≤ 26, -9 ≤ l ≤ 15
Reflections collected	5582
Independent reflections	2786 [R_{int} = 0.0247, R_{sigma} = 0.0371]
Data/restraints/parameters	2786/0/175
Goodness-of-fit on F^2	1.030
Final R indexes [I>=2σ (I)]	R_1 = 0.0495, wR_2 = 0.1141
Final R indexes [all data]	R_1 = 0.0727, wR_2 = 0.1311
Largest diff. peak/hole / e Å^{-3}	0.32/-0.25

III. Supplementary NMR Spectra

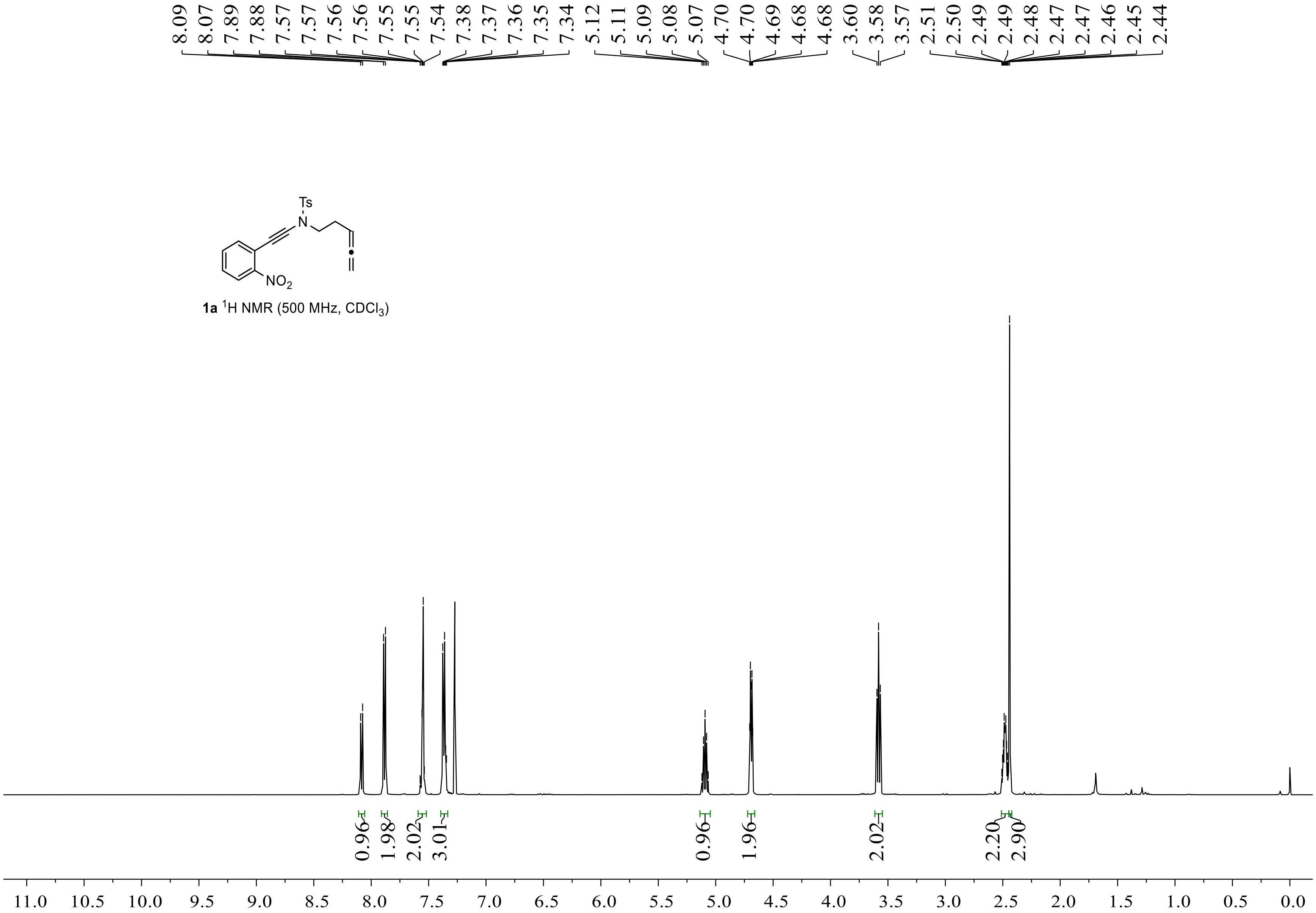

Supplementary Figure 17. ^{1}H NMR ($CDCl_3$, 500 MHz, 298 K) spectrum for **1a**

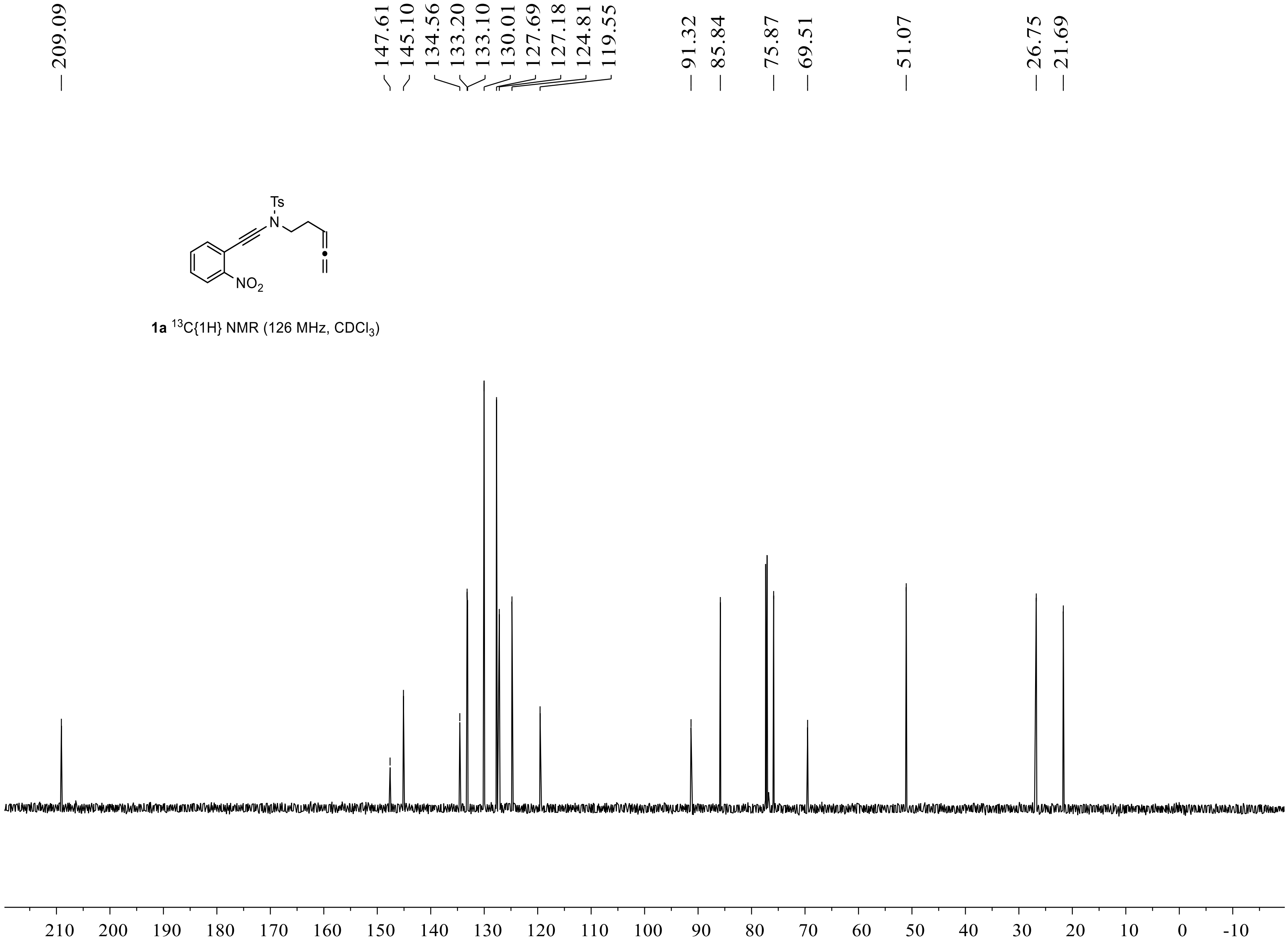

Supplementary Figure 18. ^{13}C NMR ($CDCl_3$, 126 MHz, 298 K) spectrum for **1a**

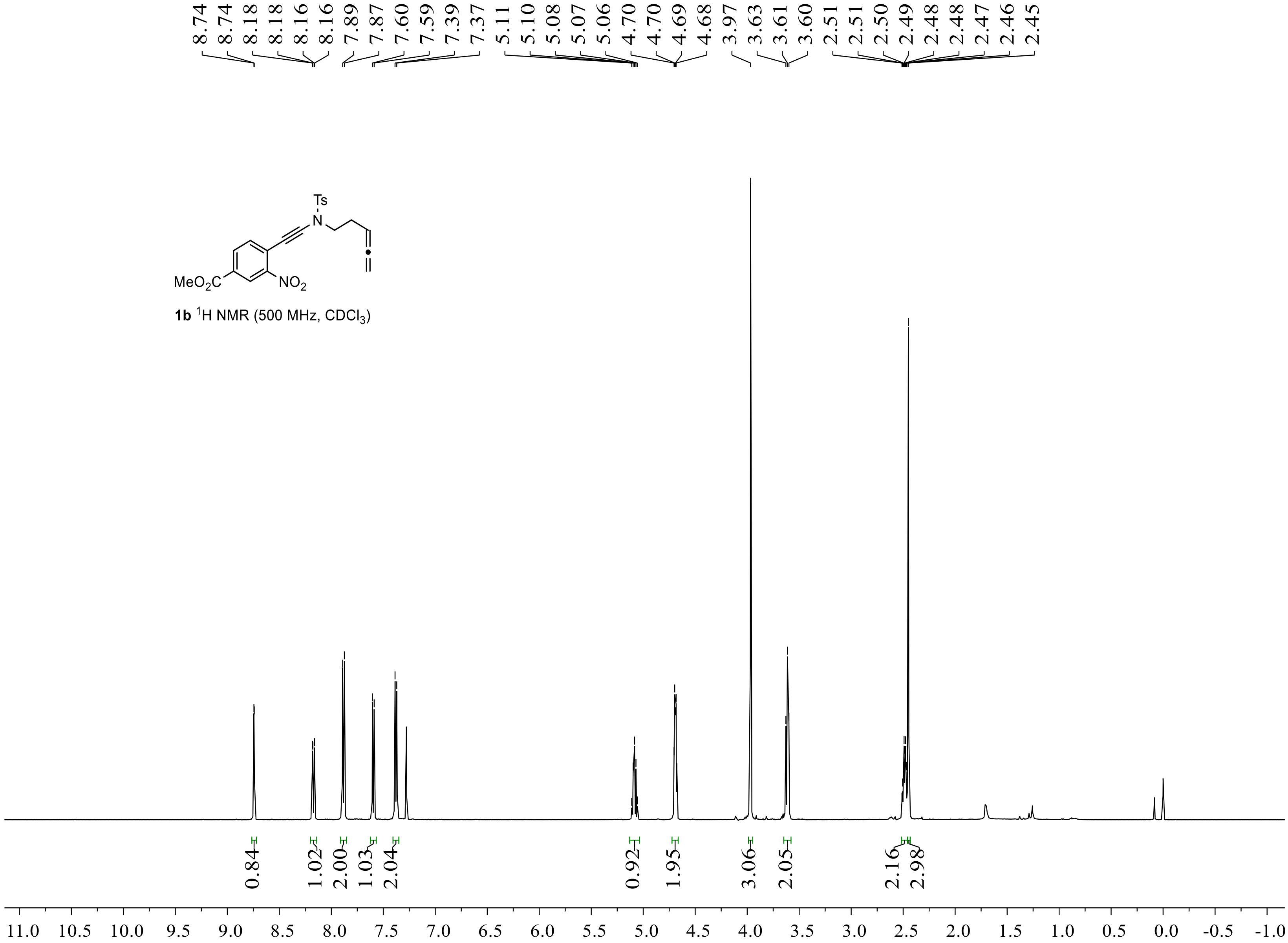

Supplementary Figure 19. ^{1}H NMR ($CDCl_3$, 500 MHz, 298 K) spectrum for **1b**

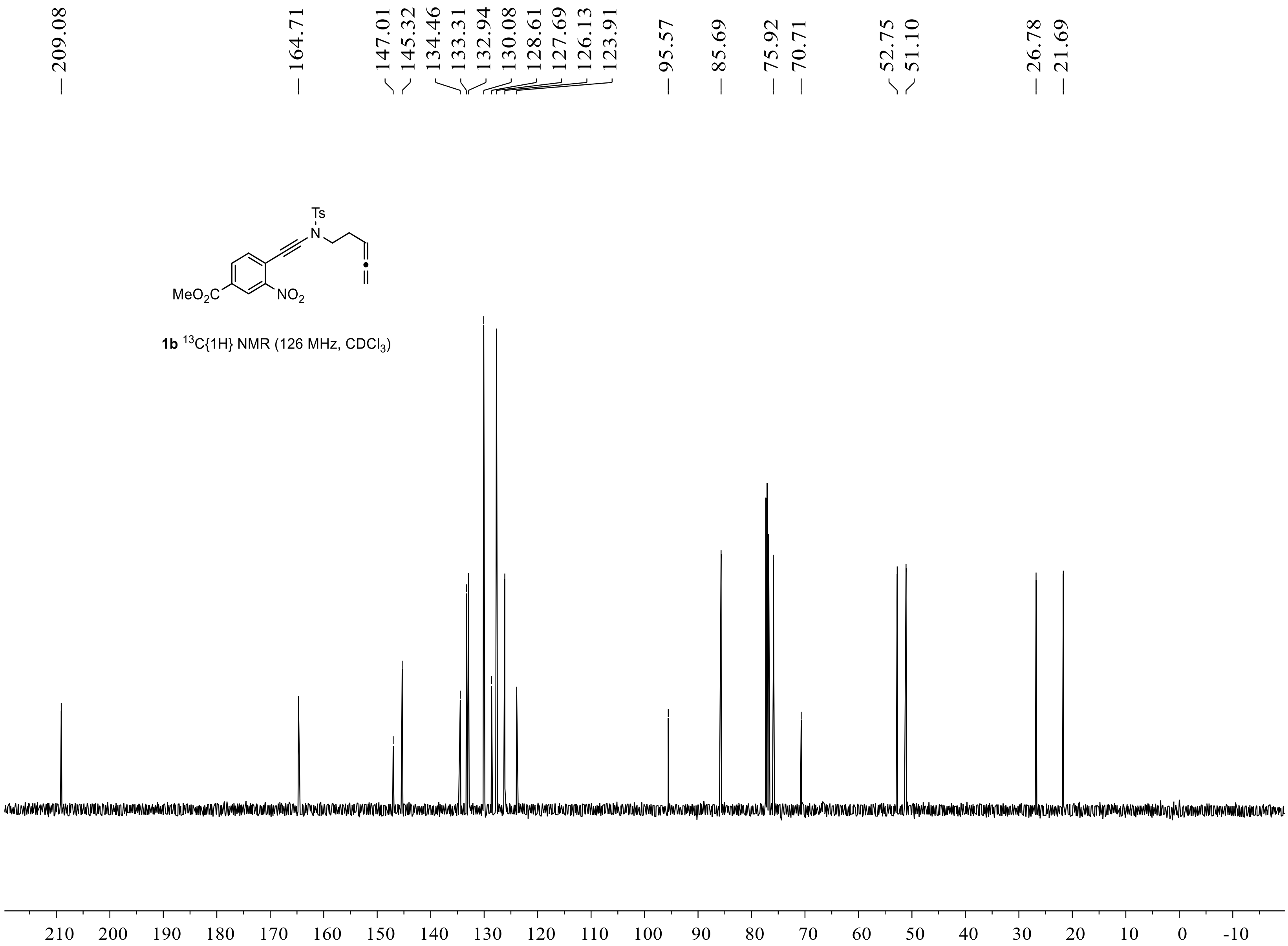

Supplementary Figure 20. ^{13}C NMR ($CDCl_3$, 126 MHz, 298 K) spectrum for **1b**

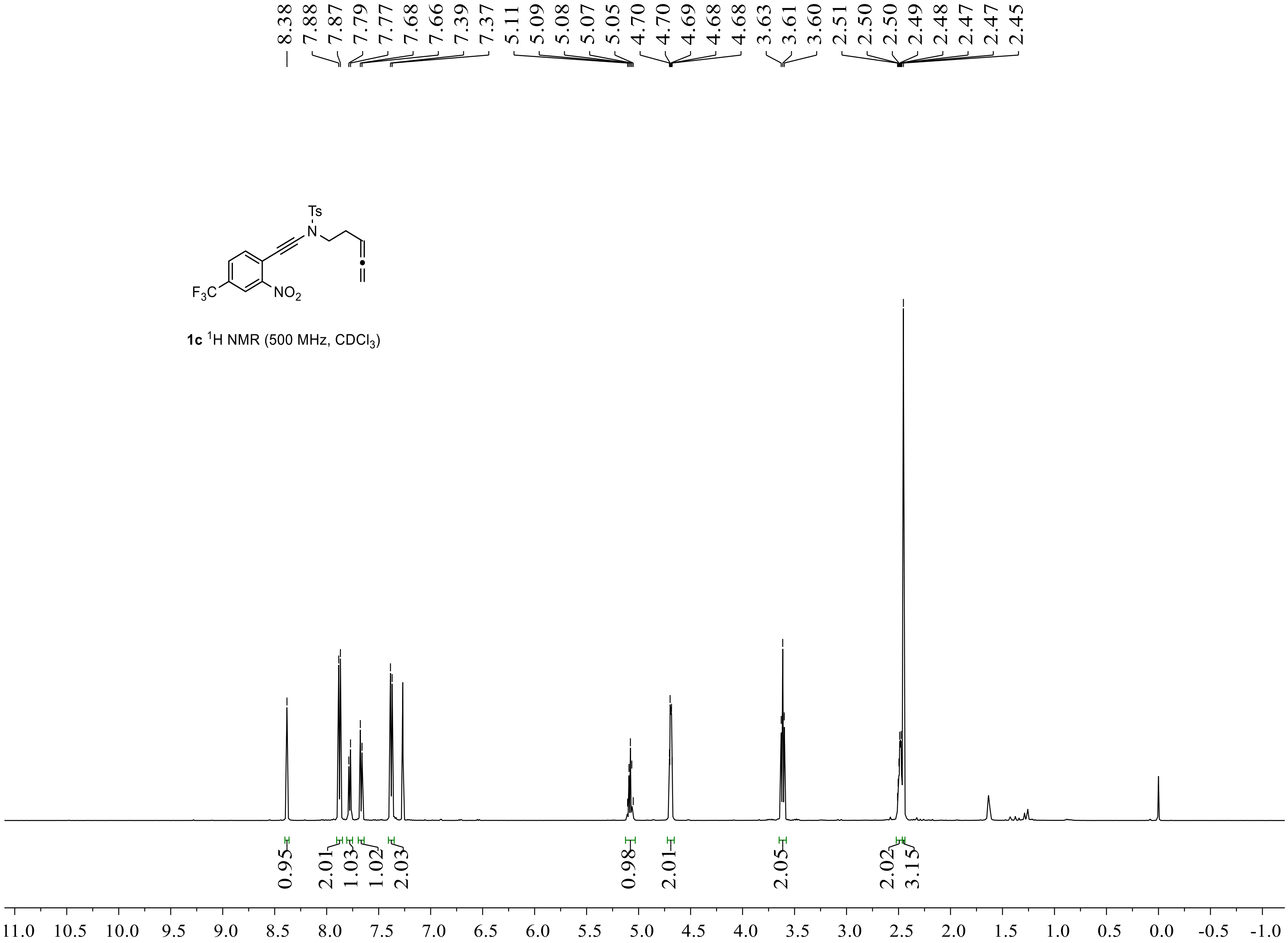

Supplementary Figure 21. ^{1}H NMR ($CDCl_3$, 500 MHz, 298 K) spectrum for **1c**

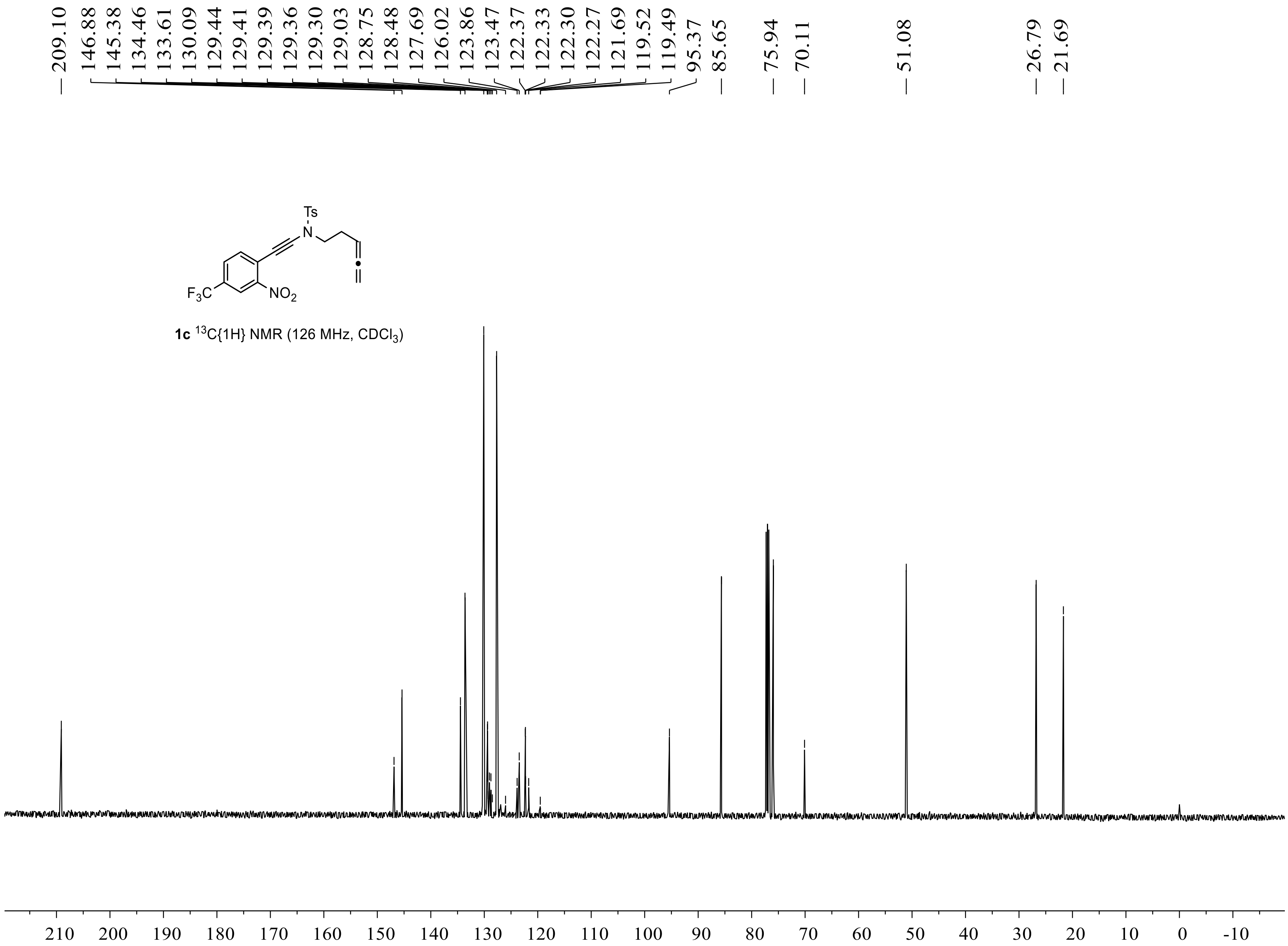

Supplementary Figure 22. ^{13}C NMR ($CDCl_3$, 126 MHz, 298 K) spectrum for **1c**

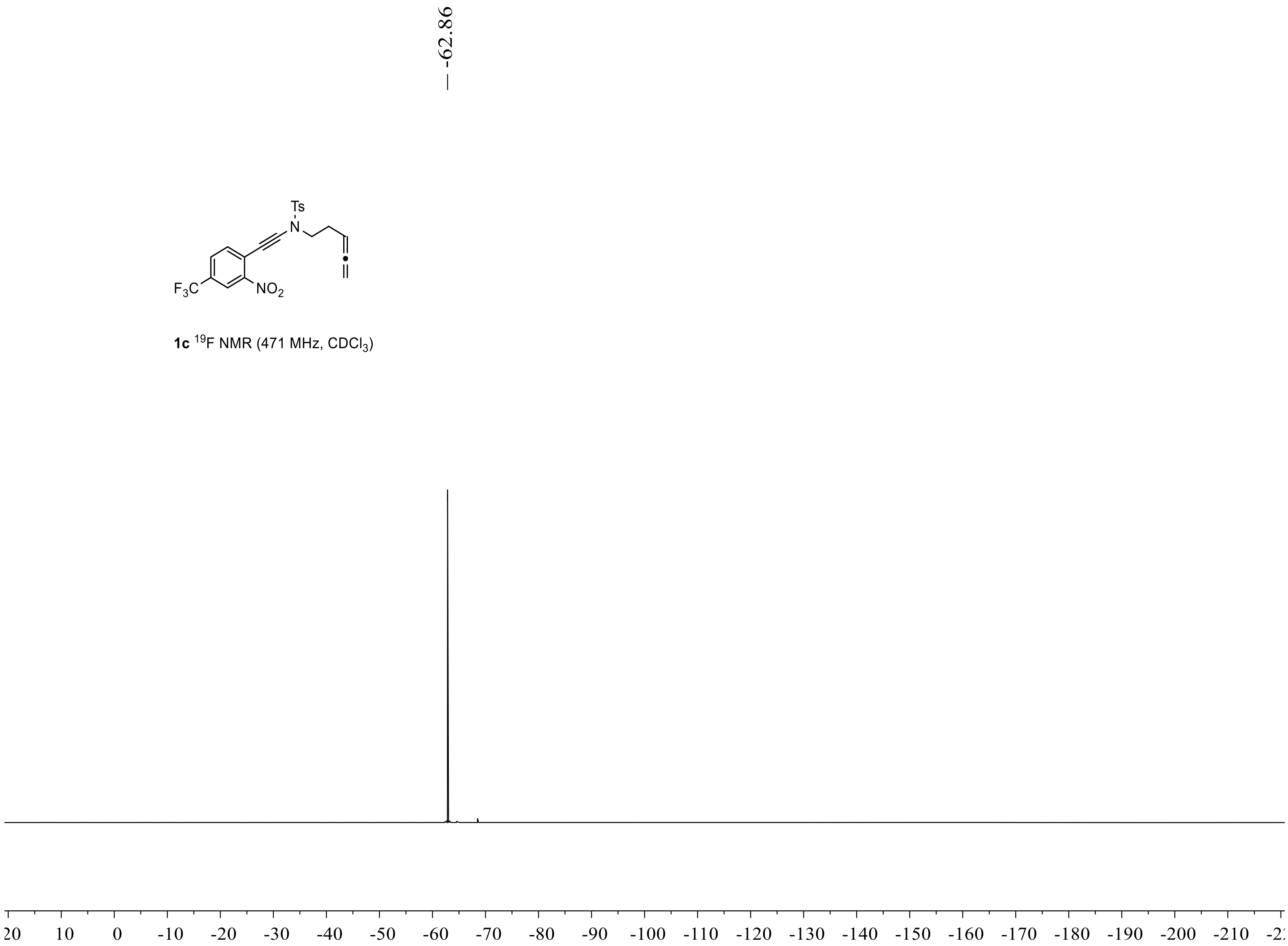

Supplementary Figure 23. ^{19}F NMR ($CDCl_3$, 471 MHz, 298 K) spectrum for **1c**

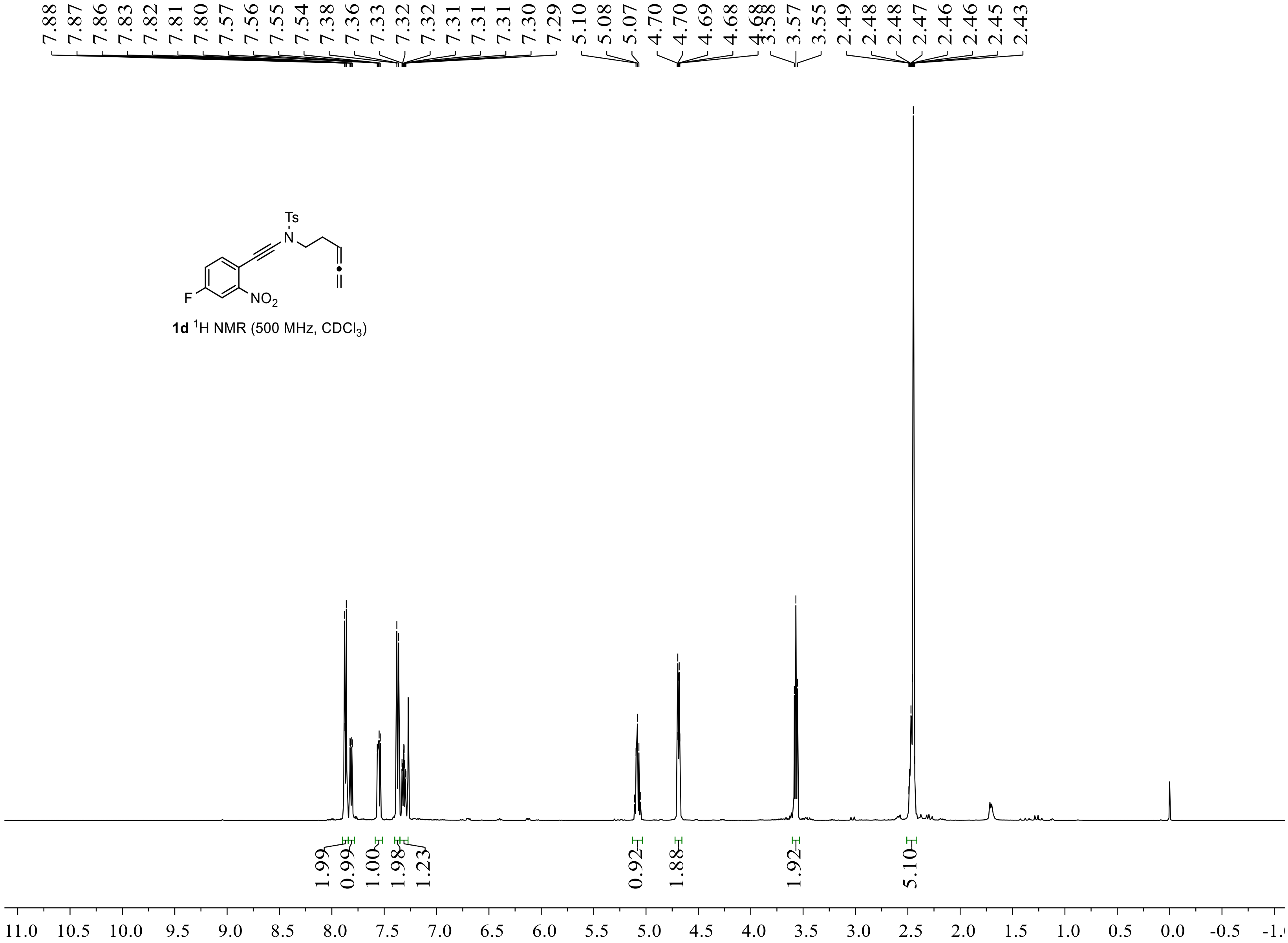

Supplementary Figure 24. ^{1}H NMR ($CDCl_3$, 500 MHz, 298 K) spectrum for **1d**

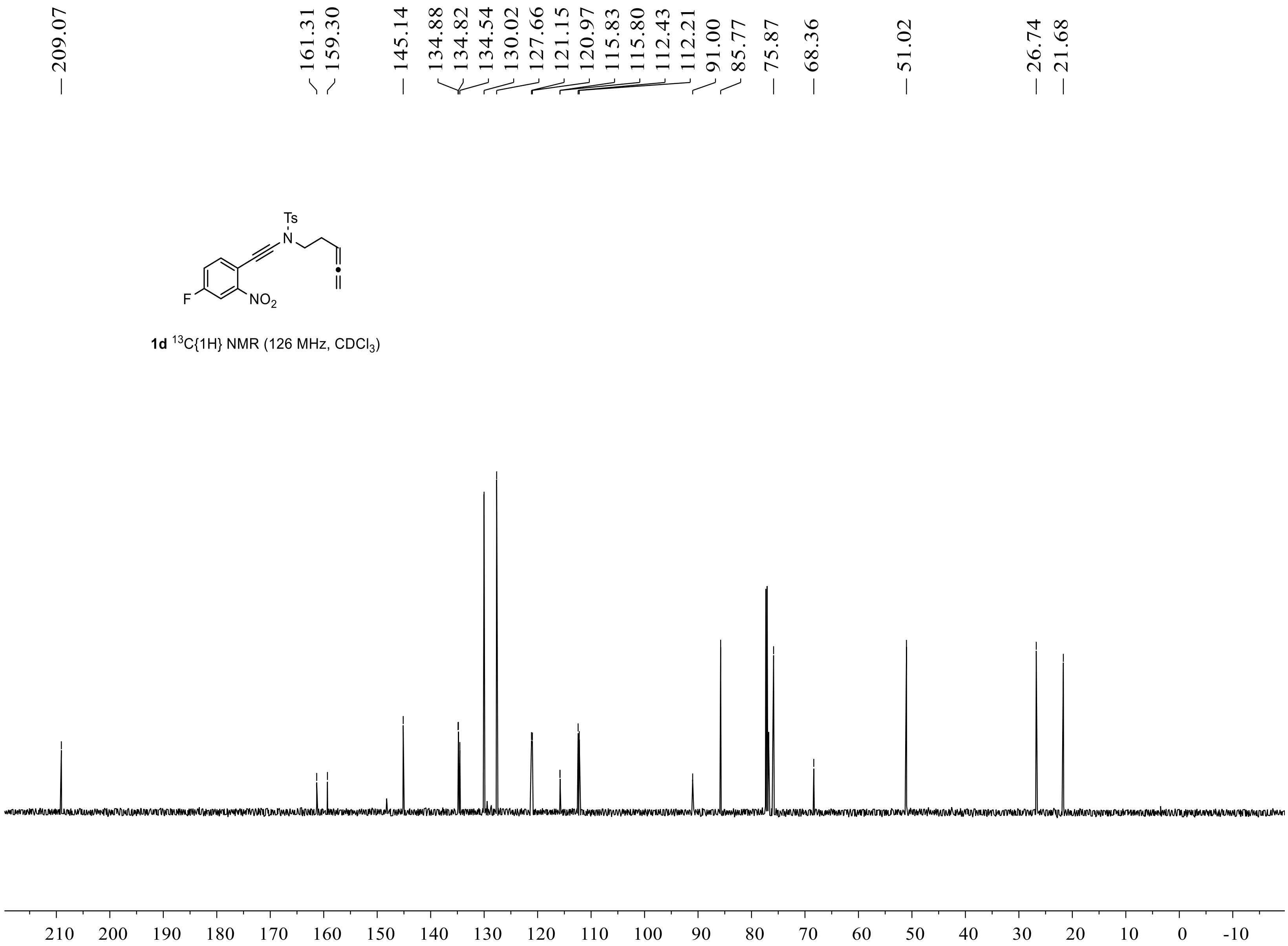

Supplementary Figure 25. ^{13}C NMR ($CDCl_3$, 126 MHz, 298 K) spectrum for **1d**

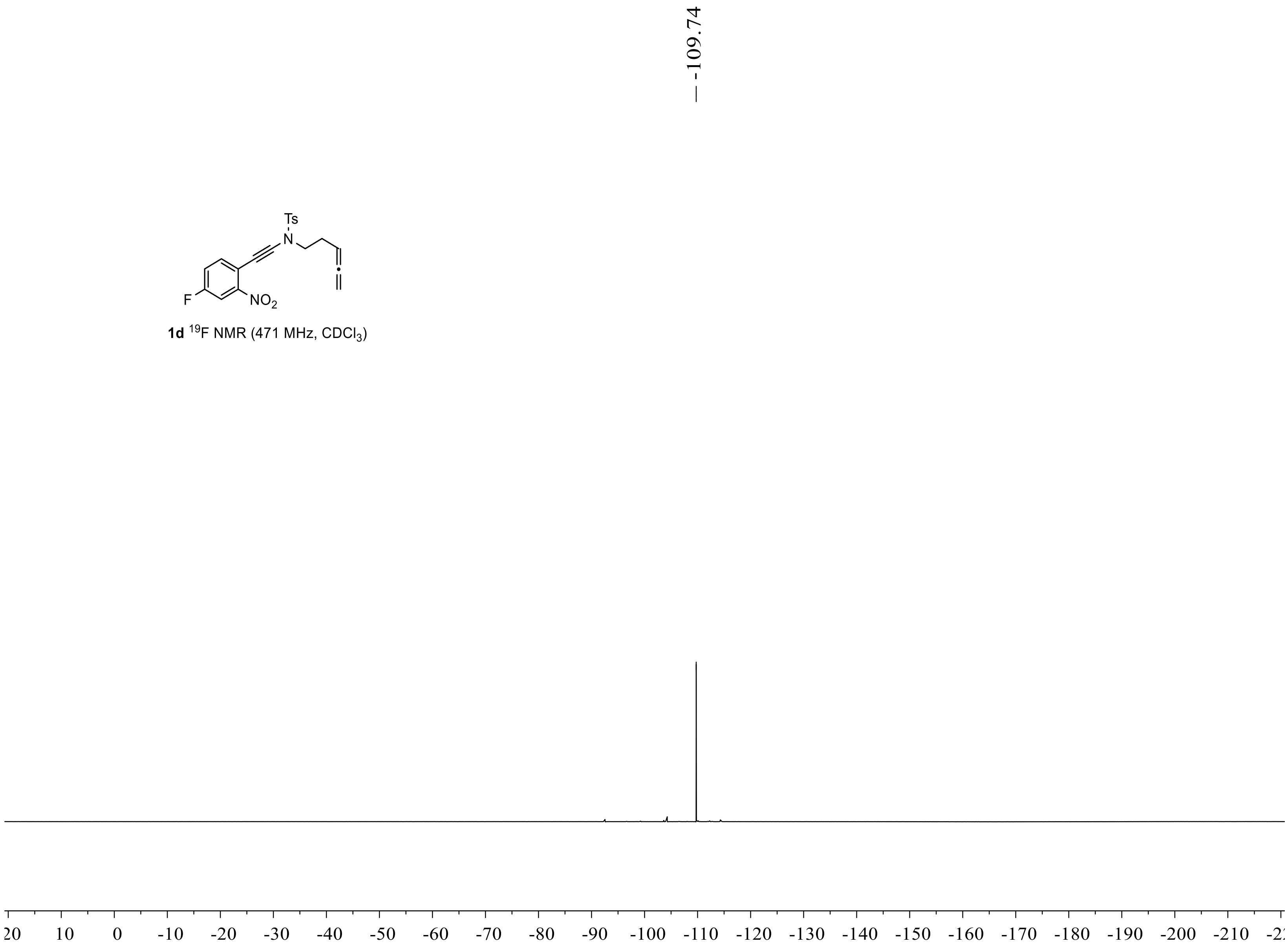

Supplementary Figure 26. ^{19}F NMR ($CDCl_3$, 471 MHz, 298 K) spectrum for **1d**

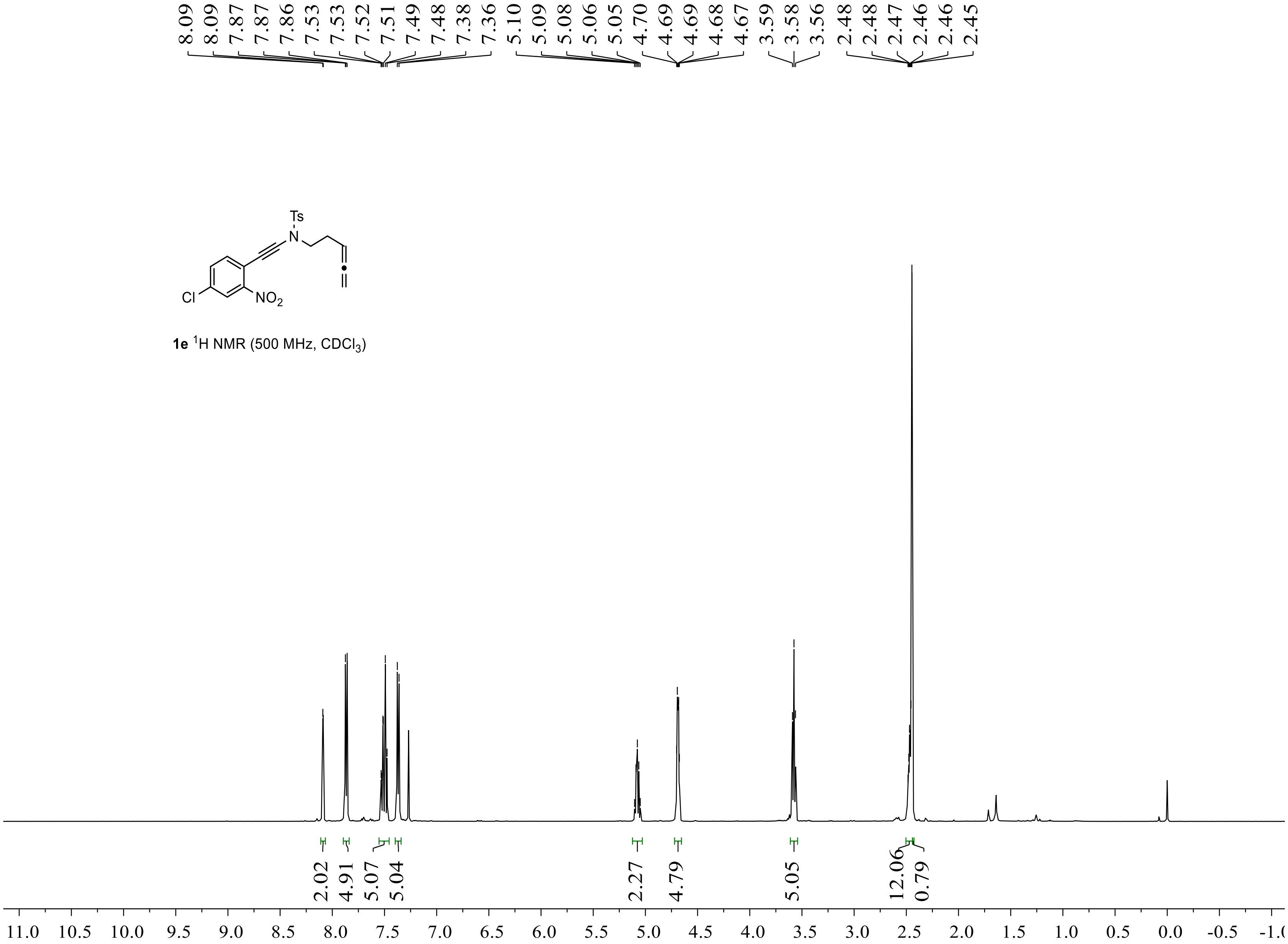

Supplementary Figure 27. ^{1}H NMR ($CDCl_3$, 500 MHz, 298 K) spectrum for **1e**

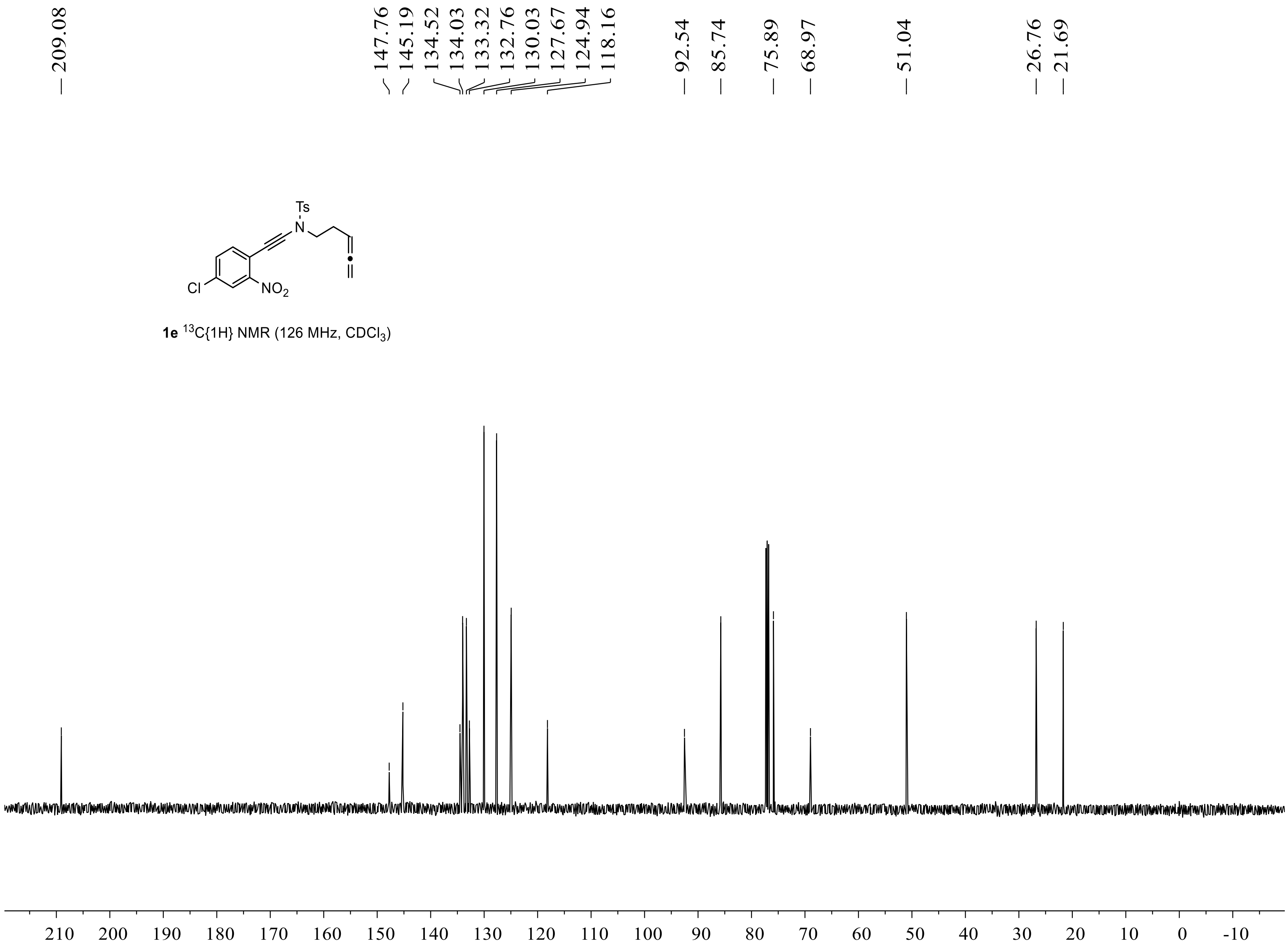

Supplementary Figure 28. ^{13}C NMR ($CDCl_3$, 126 MHz, 298 K) spectrum for **1e**

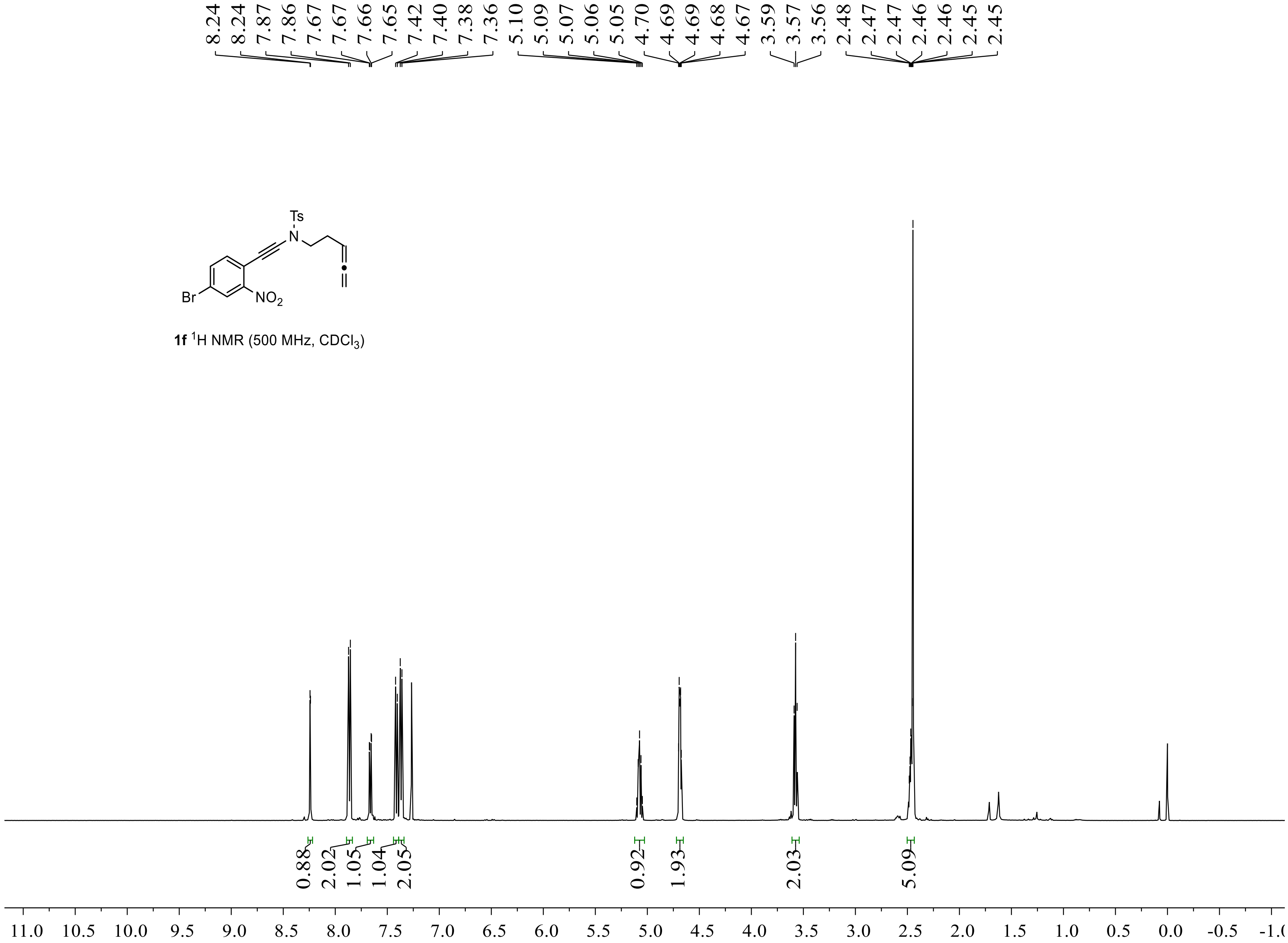

Supplementary Figure 29. ^{1}H NMR ($CDCl_3$, 500 MHz, 298 K) spectrum for **1f**

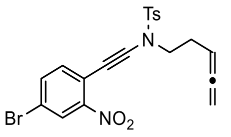

1f $^{13}C\{1H\}$ NMR (126 MHz, $CDCl_3$)

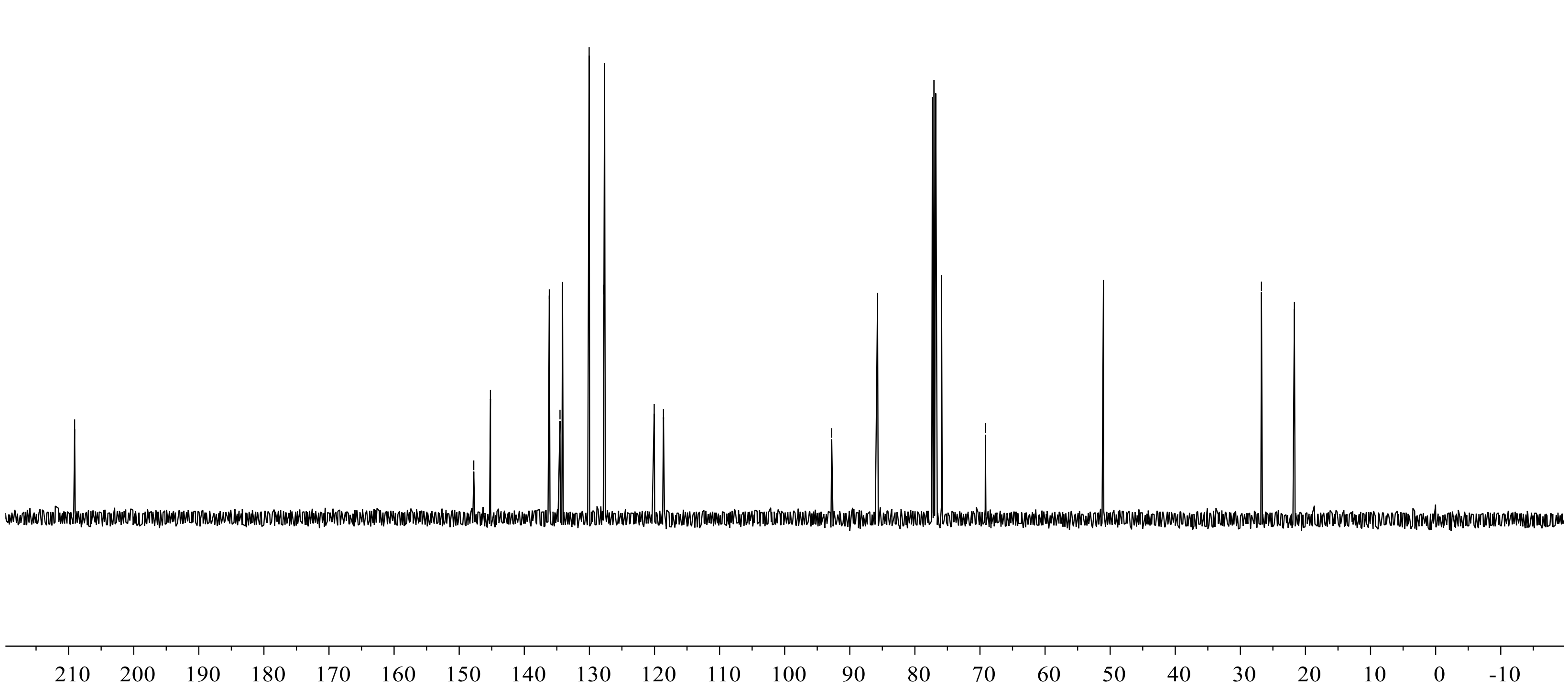

Supplementary Figure 30. ^{13}C NMR ($CDCl_3$, 126 MHz, 298 K) spectrum for **1f**

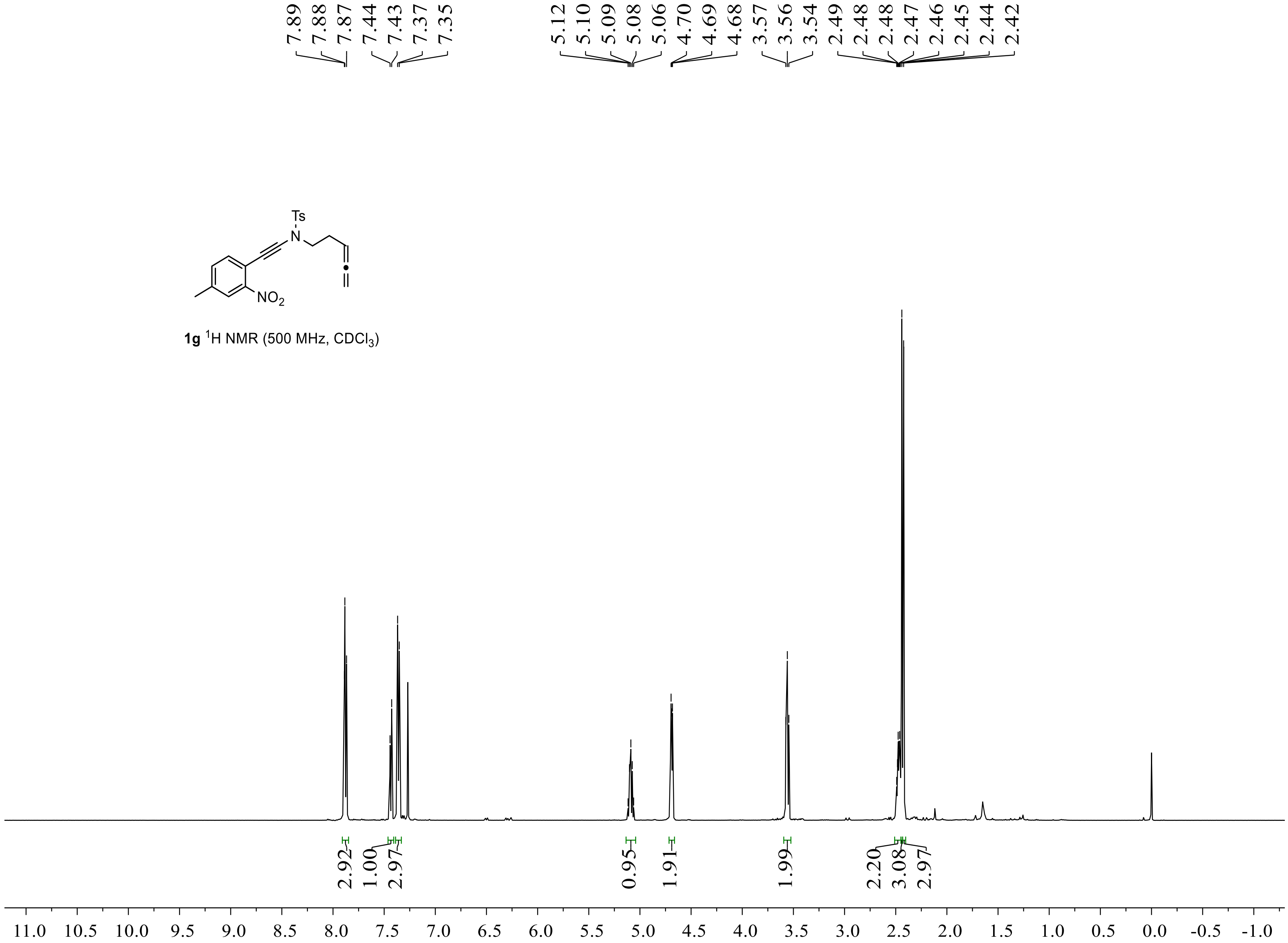

Supplementary Figure 31. ^{1}H NMR ($CDCl_3$, 500 MHz, 298 K) spectrum for **1g**

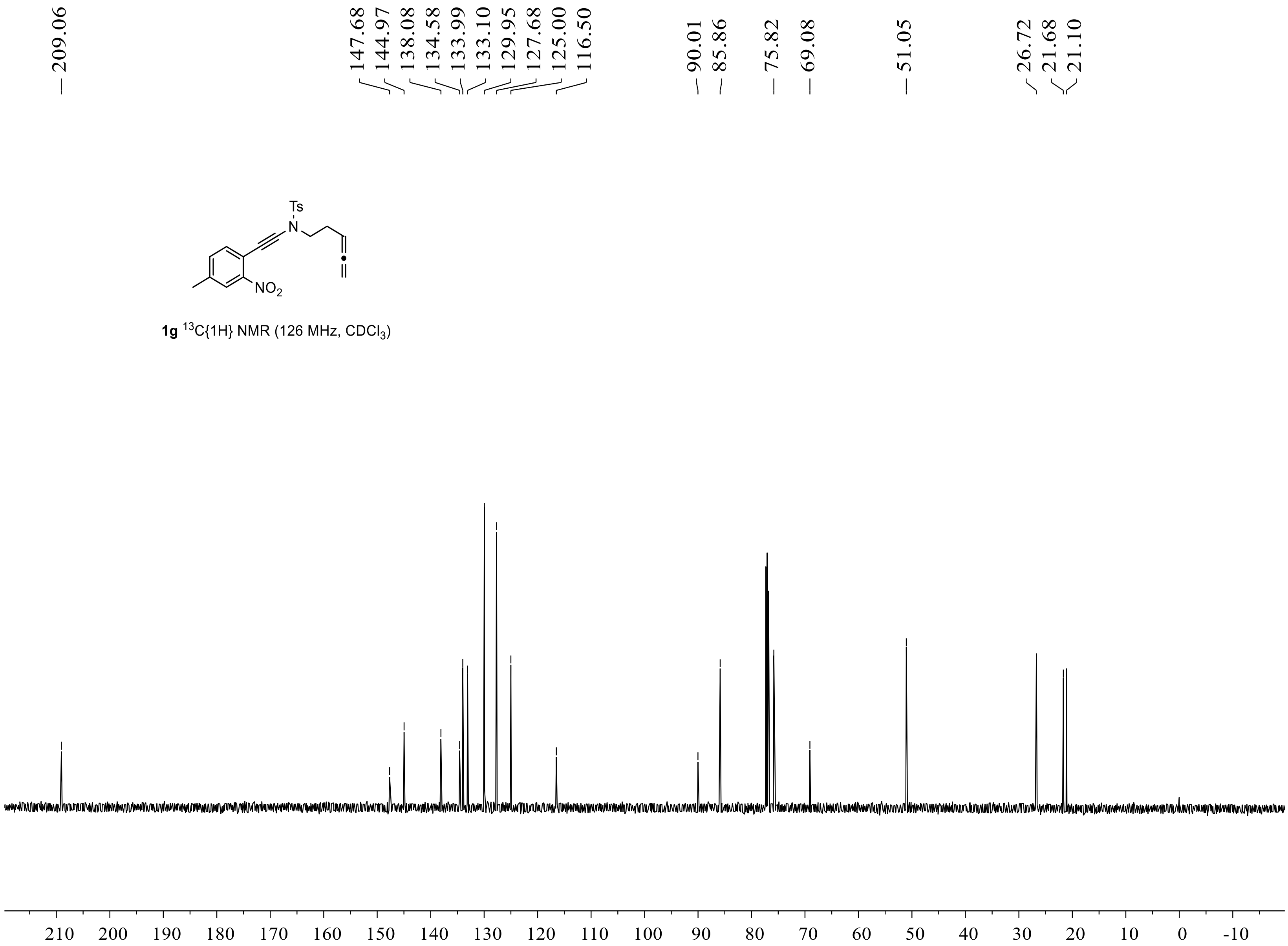

Supplementary Figure 32. ^{13}C NMR ($CDCl_3$, 126 MHz, 298 K) spectrum for **1g**

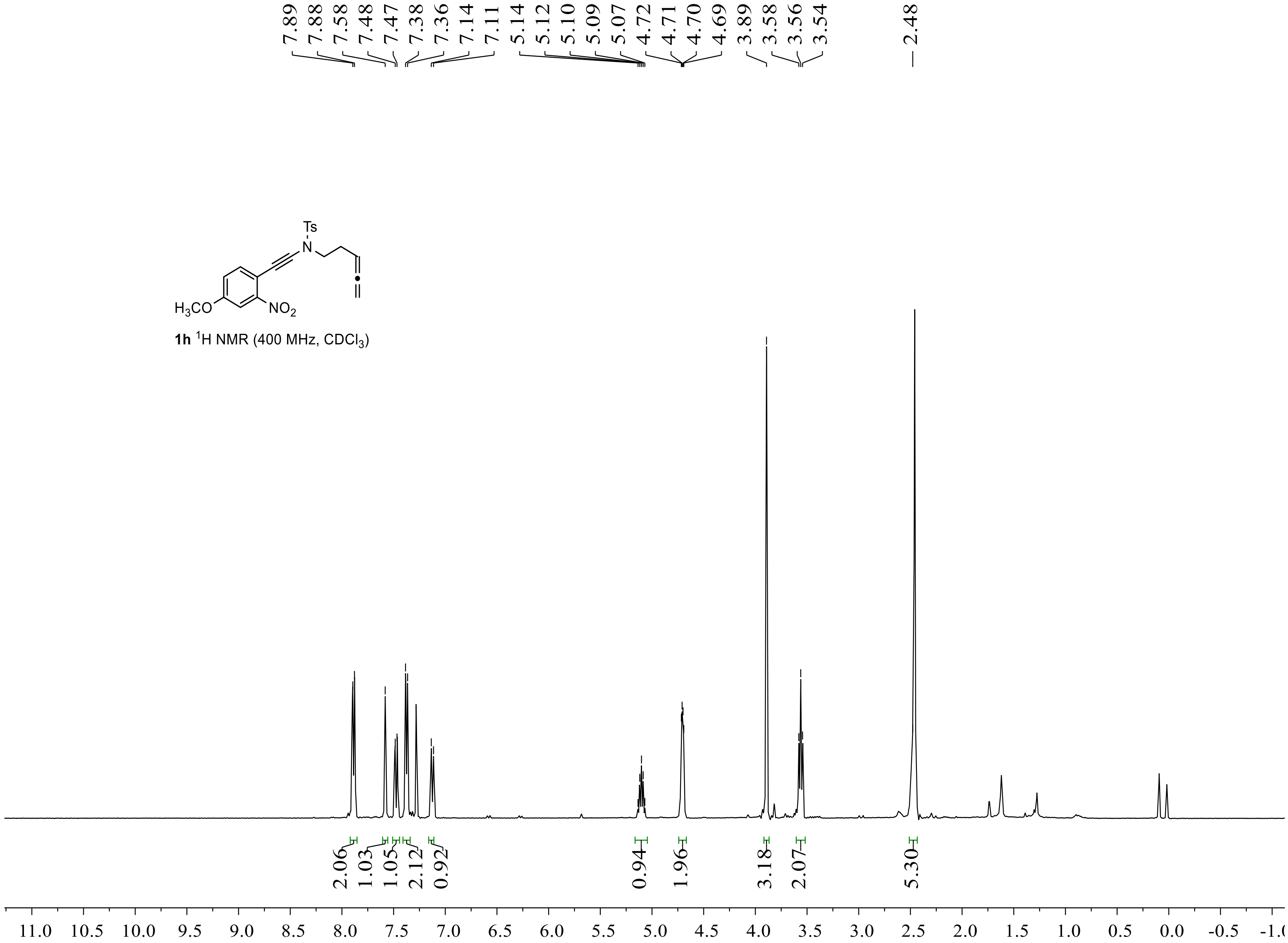

Supplementary Figure 33. ^{1}H NMR ($CDCl_3$, 400 MHz, 298 K) spectrum for **1h**

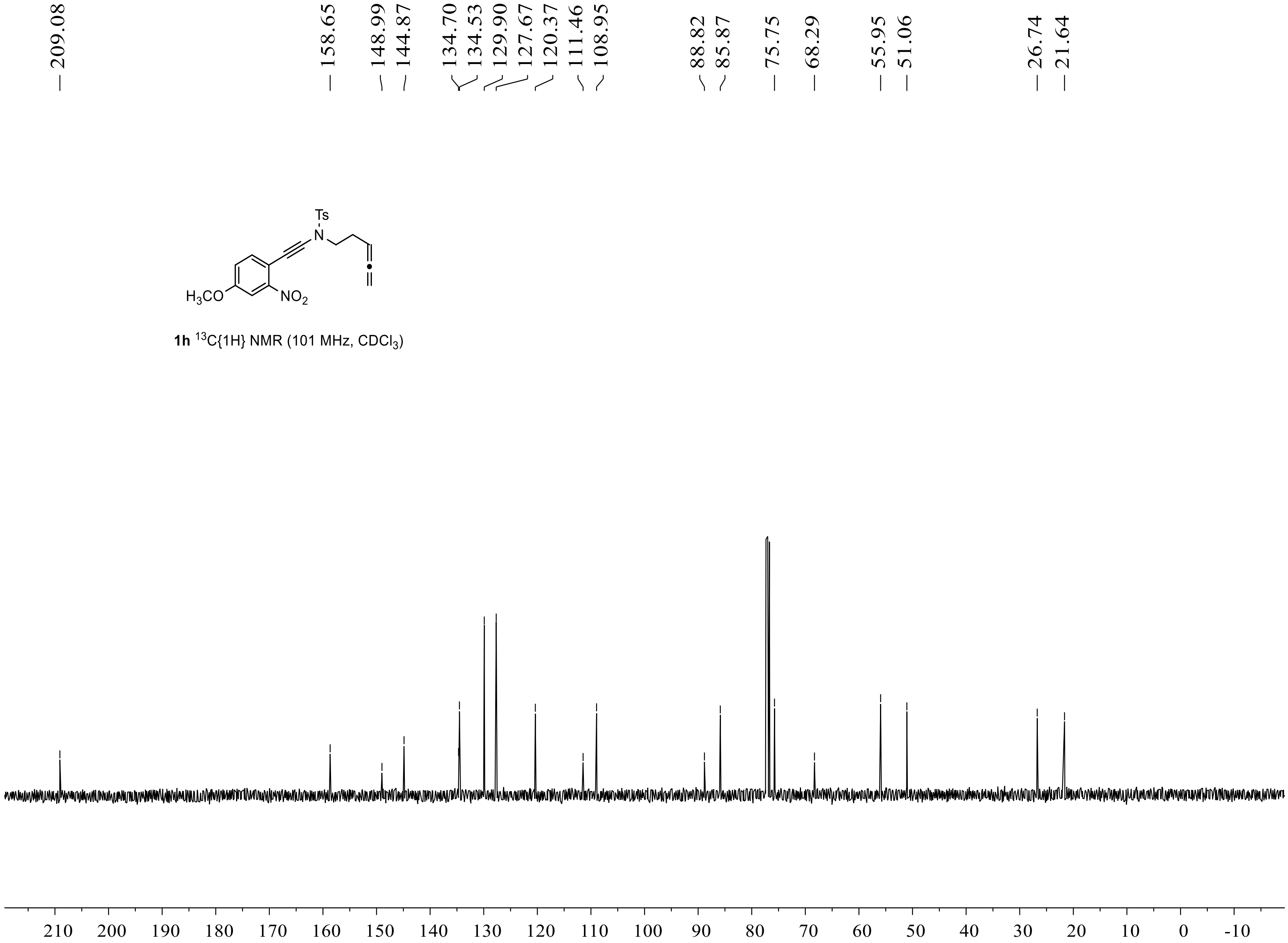

Supplementary Figure 34. ^{13}C NMR ($CDCl_3$, 101 MHz, 298 K) spectrum for **1h**

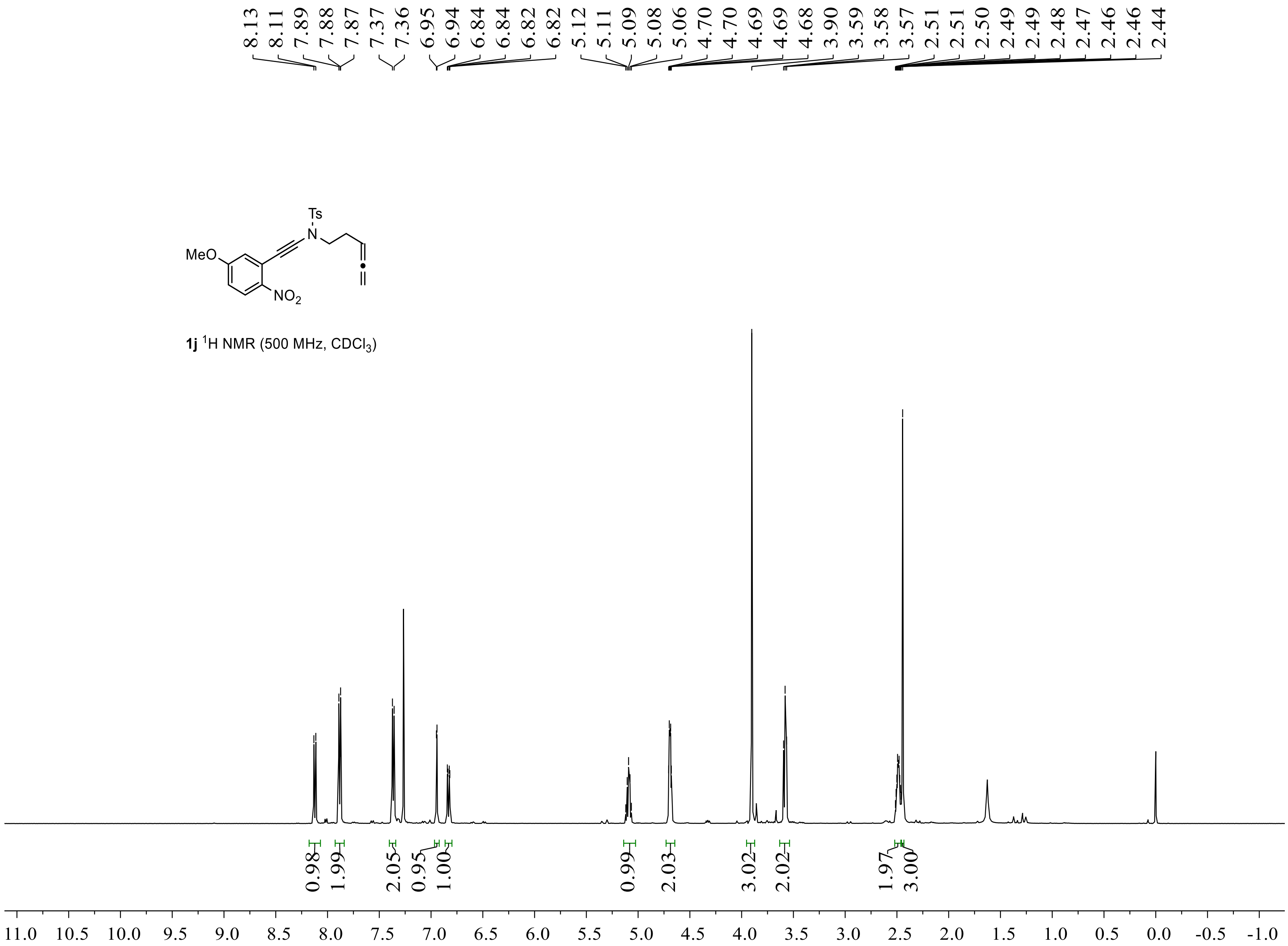

Supplementary Figure 35. ^{1}H NMR ($CDCl_3$, 500 MHz, 298 K) spectrum for **1j**

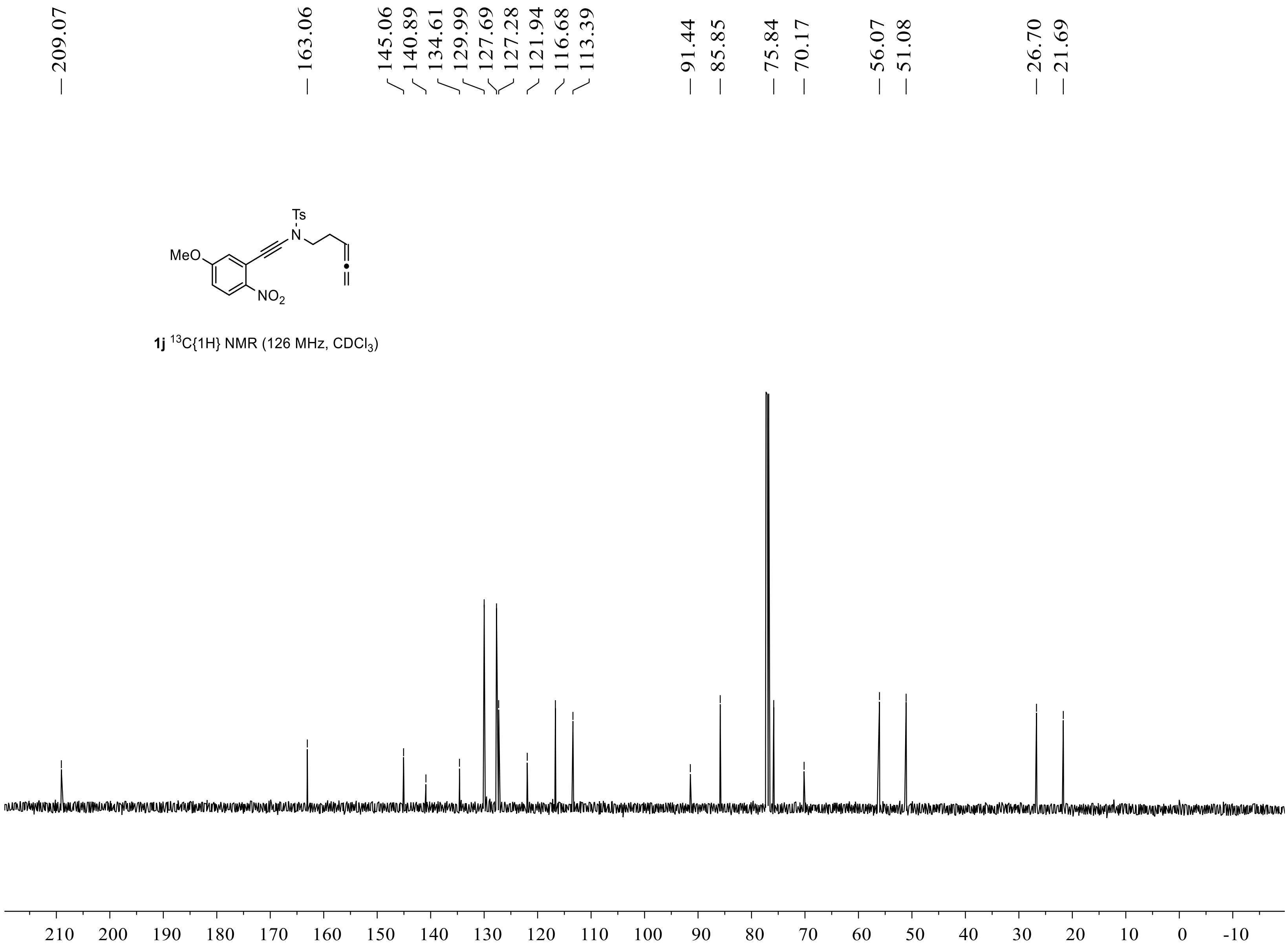

Supplementary Figure 36. ^{13}C NMR ($CDCl_3$, 126 MHz, 298 K) spectrum for **1j**

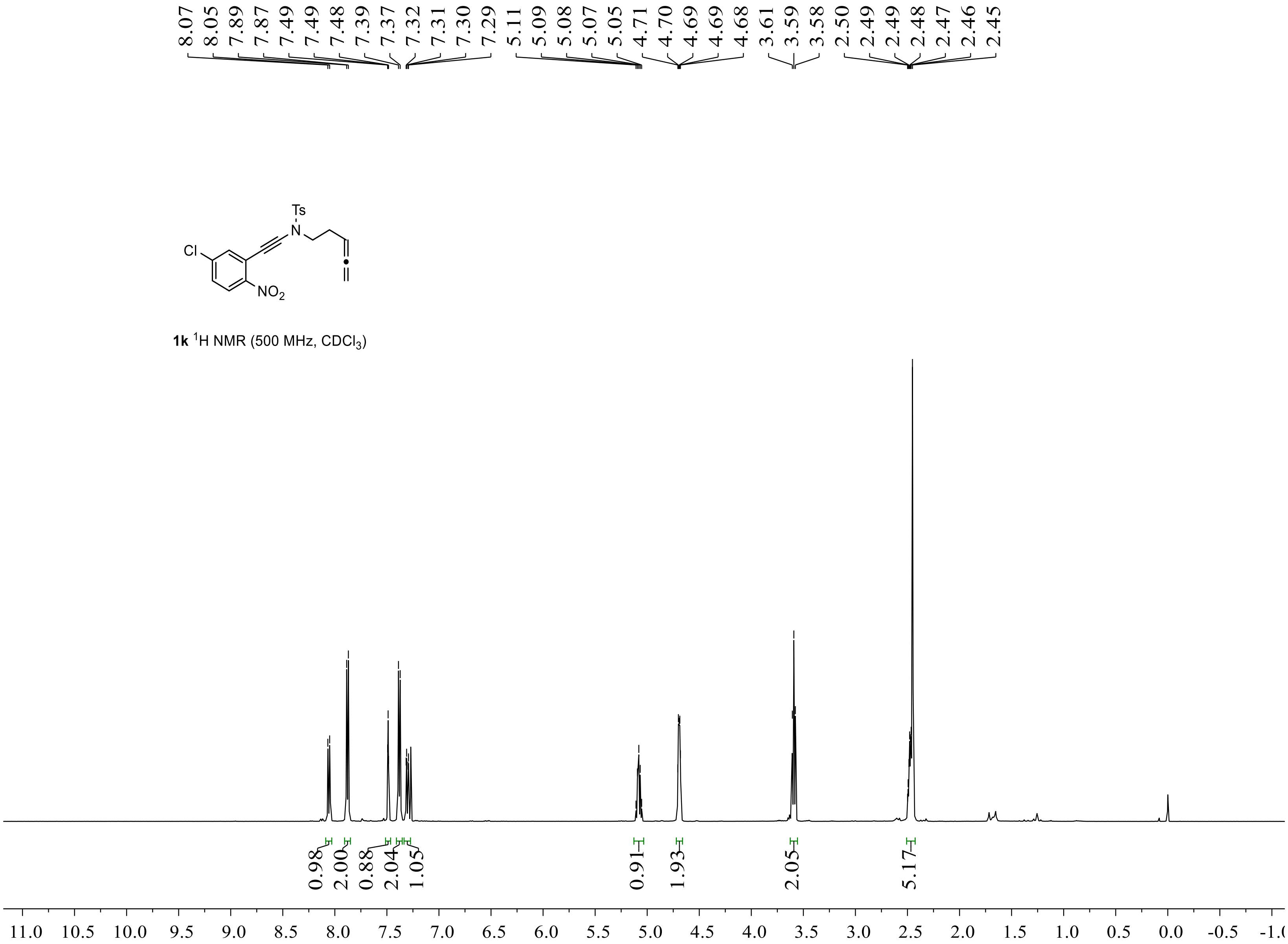

Supplementary Figure 37. ^{1}H NMR ($CDCl_3$, 500 MHz, 298 K) spectrum for **1k**

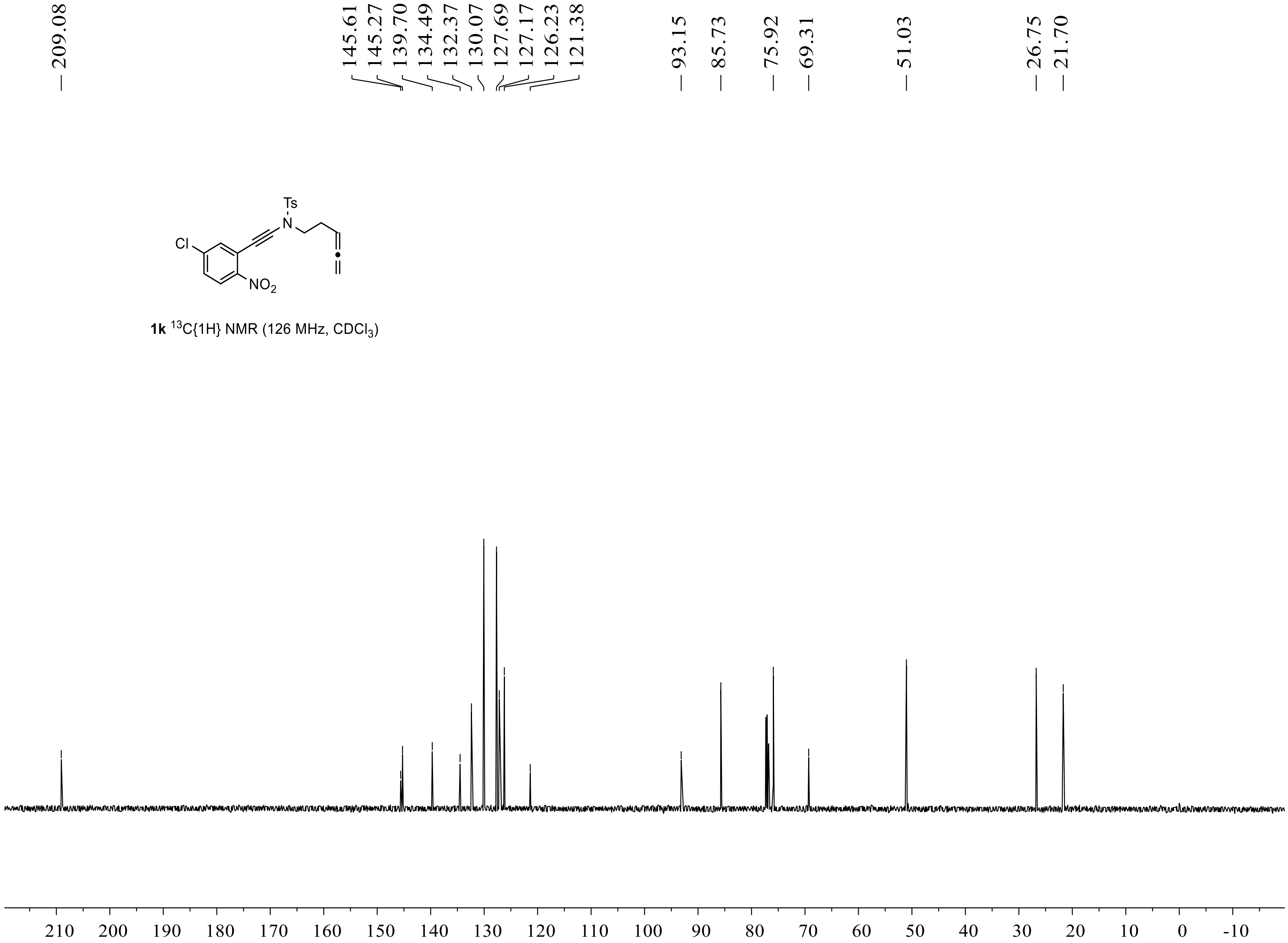

Supplementary Figure 38. ^{13}C NMR ($CDCl_3$, 126 MHz, 298 K) spectrum for **1k**

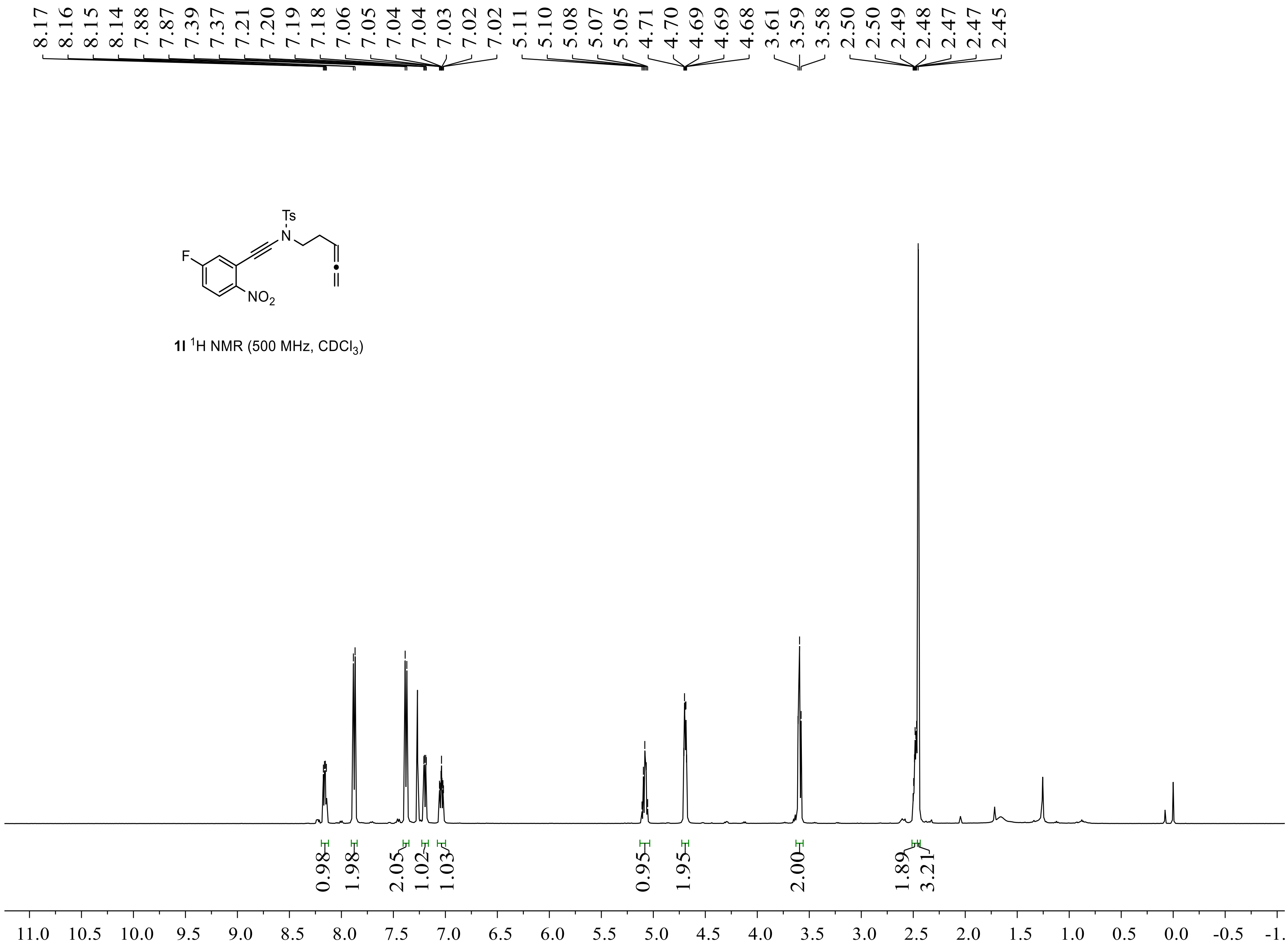

Supplementary Figure 39. ^{1}H NMR ($CDCl_3$, 500 MHz, 298 K) spectrum for **1l**

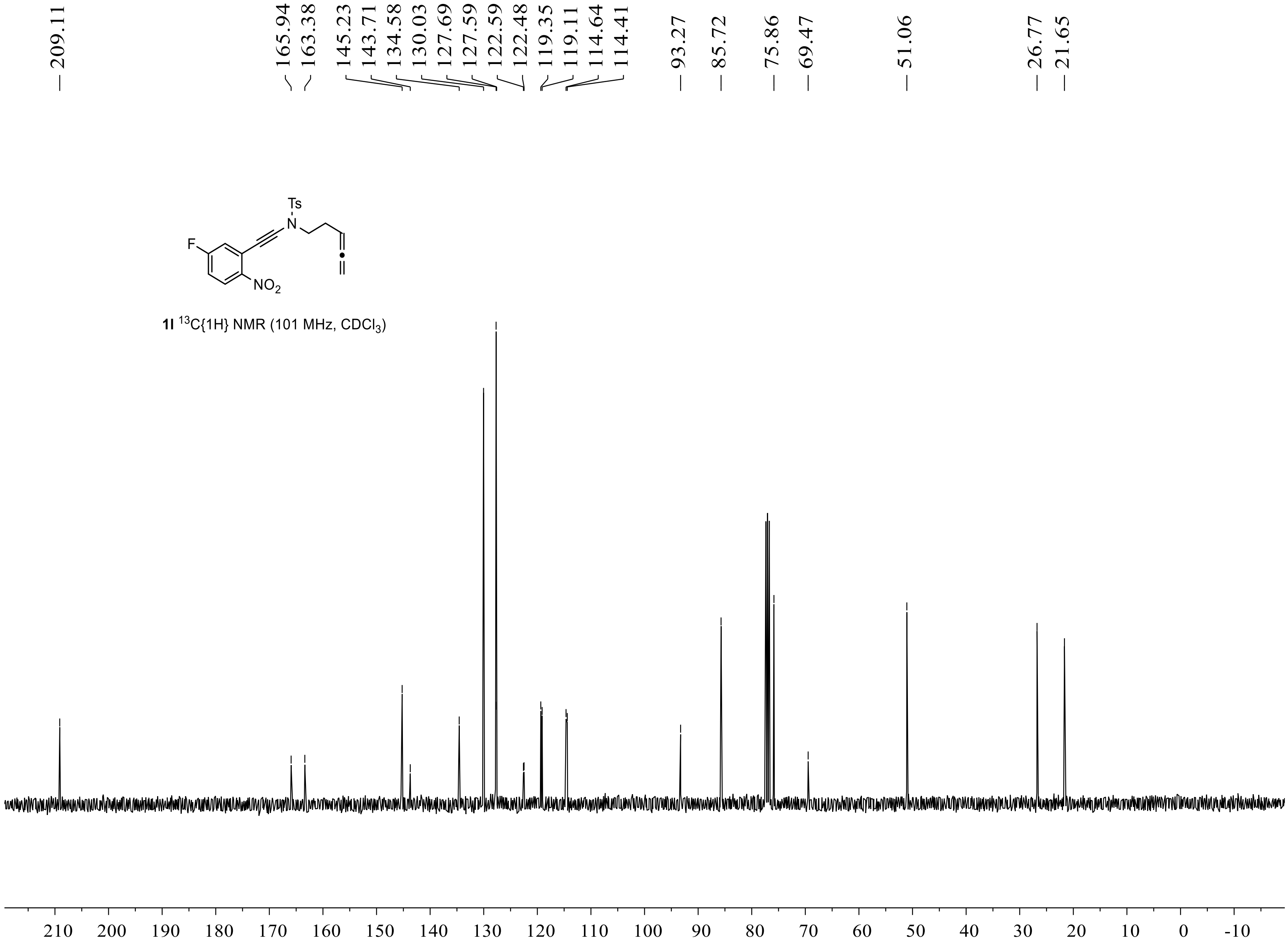

Supplementary Figure 40. ^{13}C NMR ($CDCl_3$, 126 MHz, 298 K) spectrum for **1l**

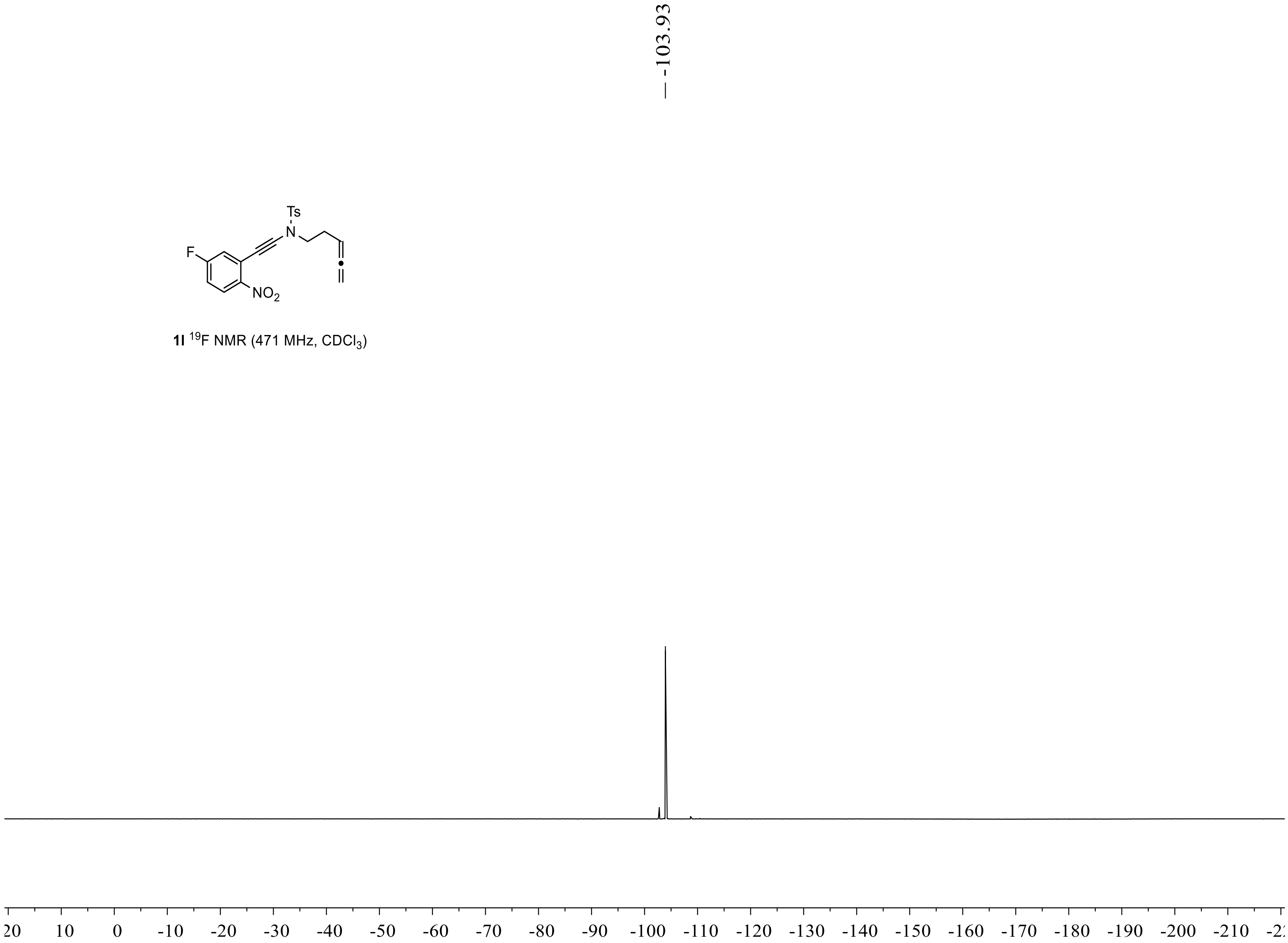

Supplementary Figure 41. ^{19}F NMR ($CDCl_3$, 471 MHz, 298 K) spectrum for **1l**

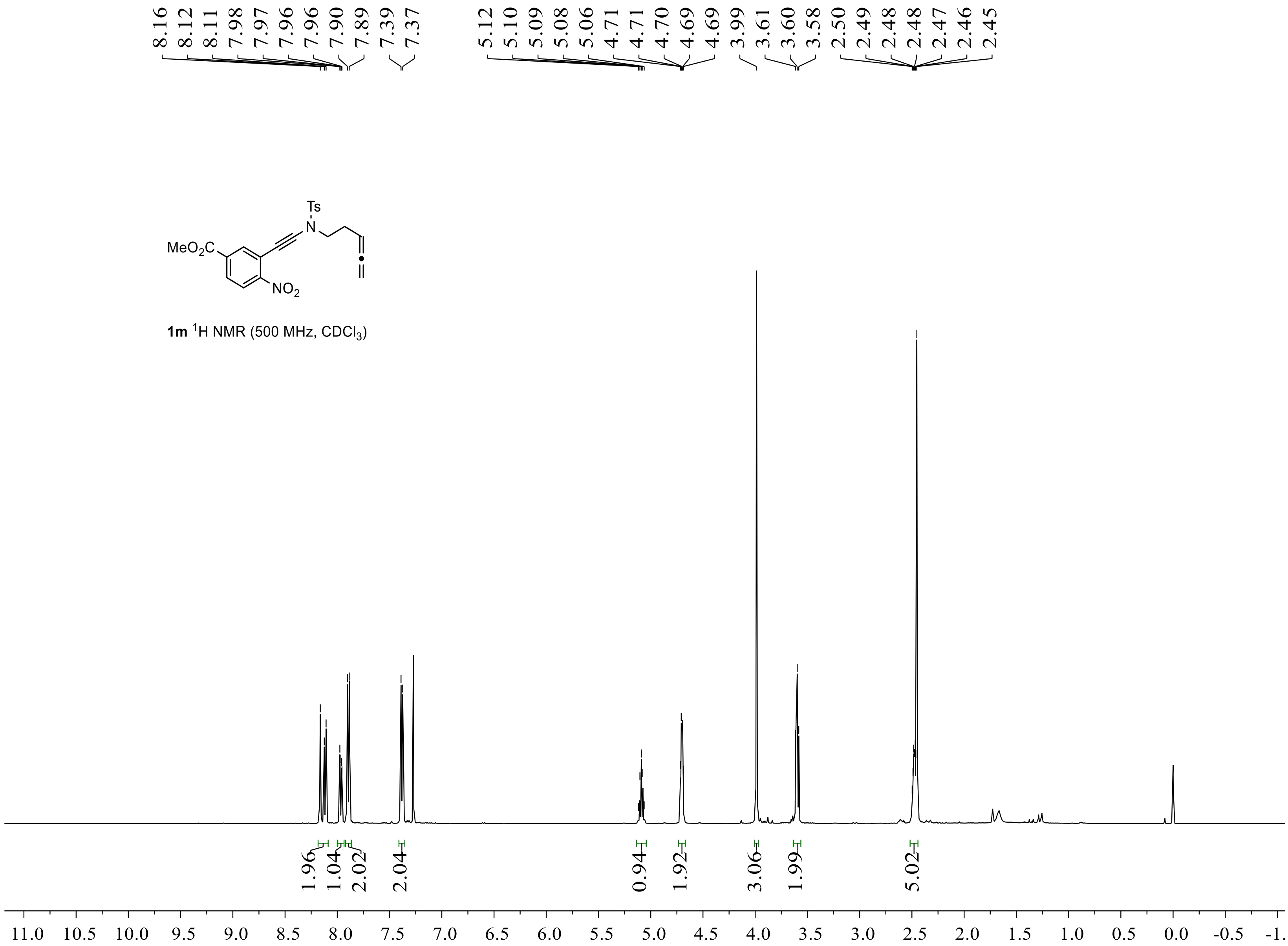

Supplementary Figure 42. ^{1}H NMR ($CDCl_3$, 500 MHz, 298 K) spectrum for **1m**

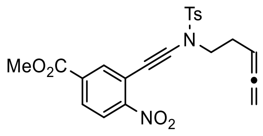

1m $^{13}C\{1H\}$ NMR (126 MHz, $CDCl_3$)

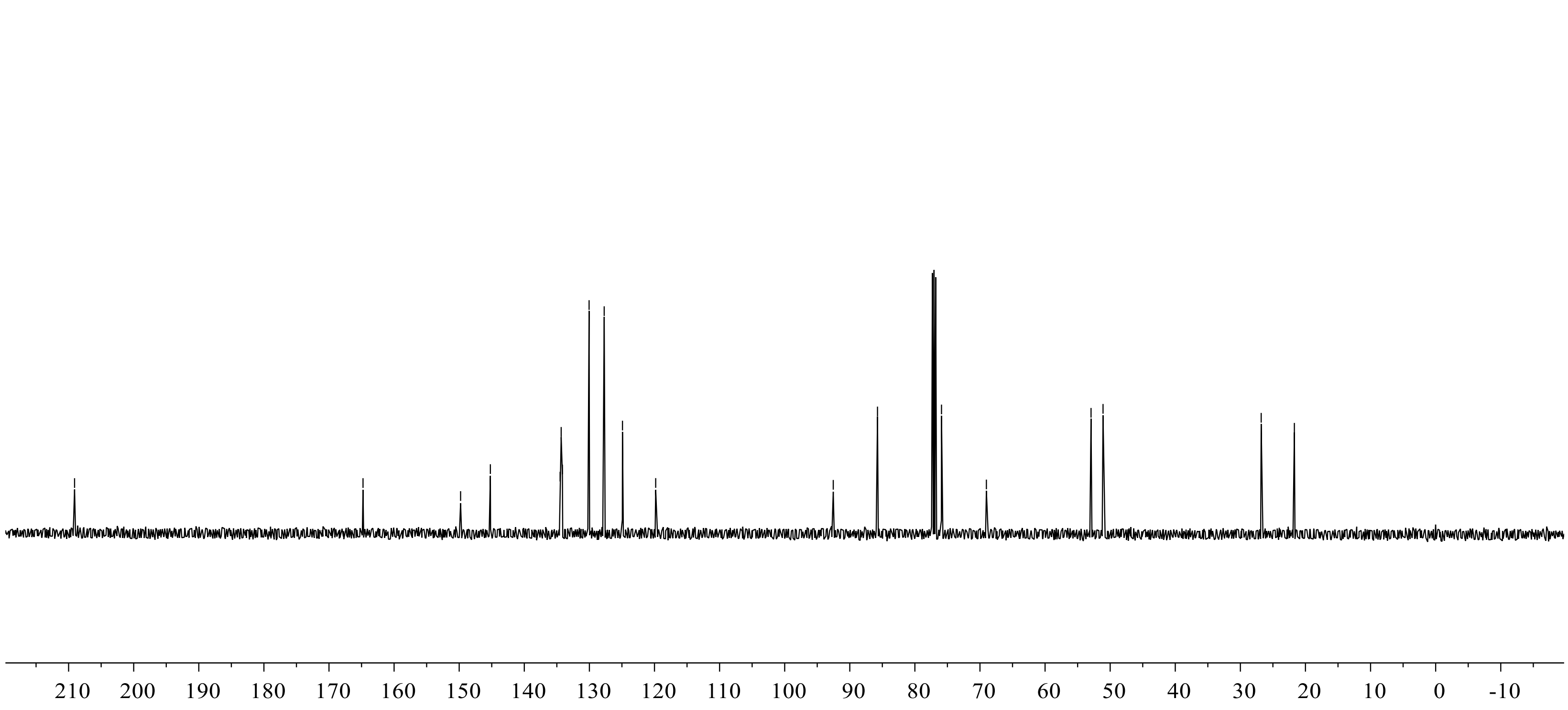

Supplementary Figure 43. ^{13}C NMR ($CDCl_3$, 126 MHz, 298 K) spectrum for **1m**

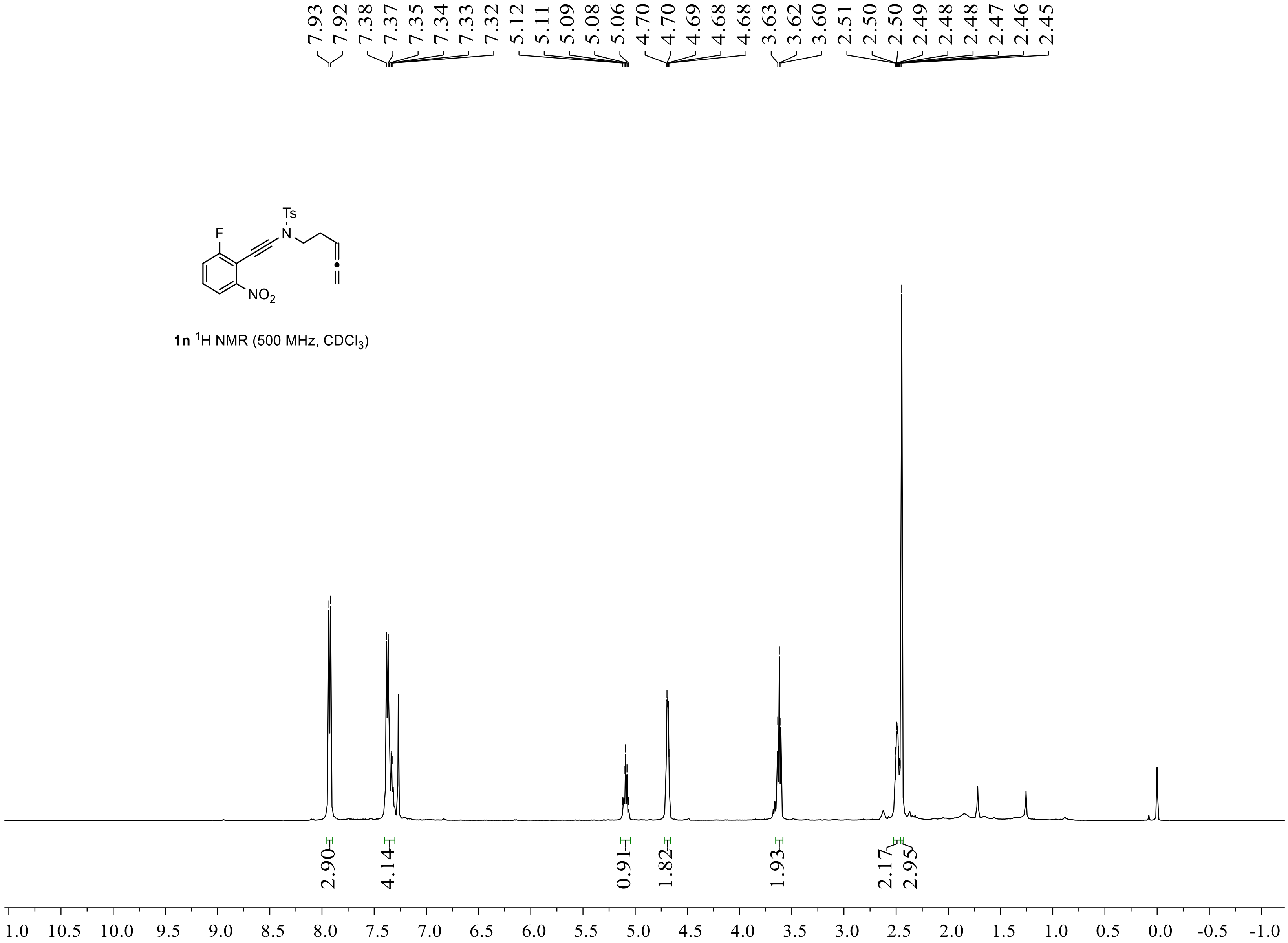

Supplementary Figure 44. ^{1}H NMR ($CDCl_3$, 500 MHz, 298 K) spectrum for **1n**

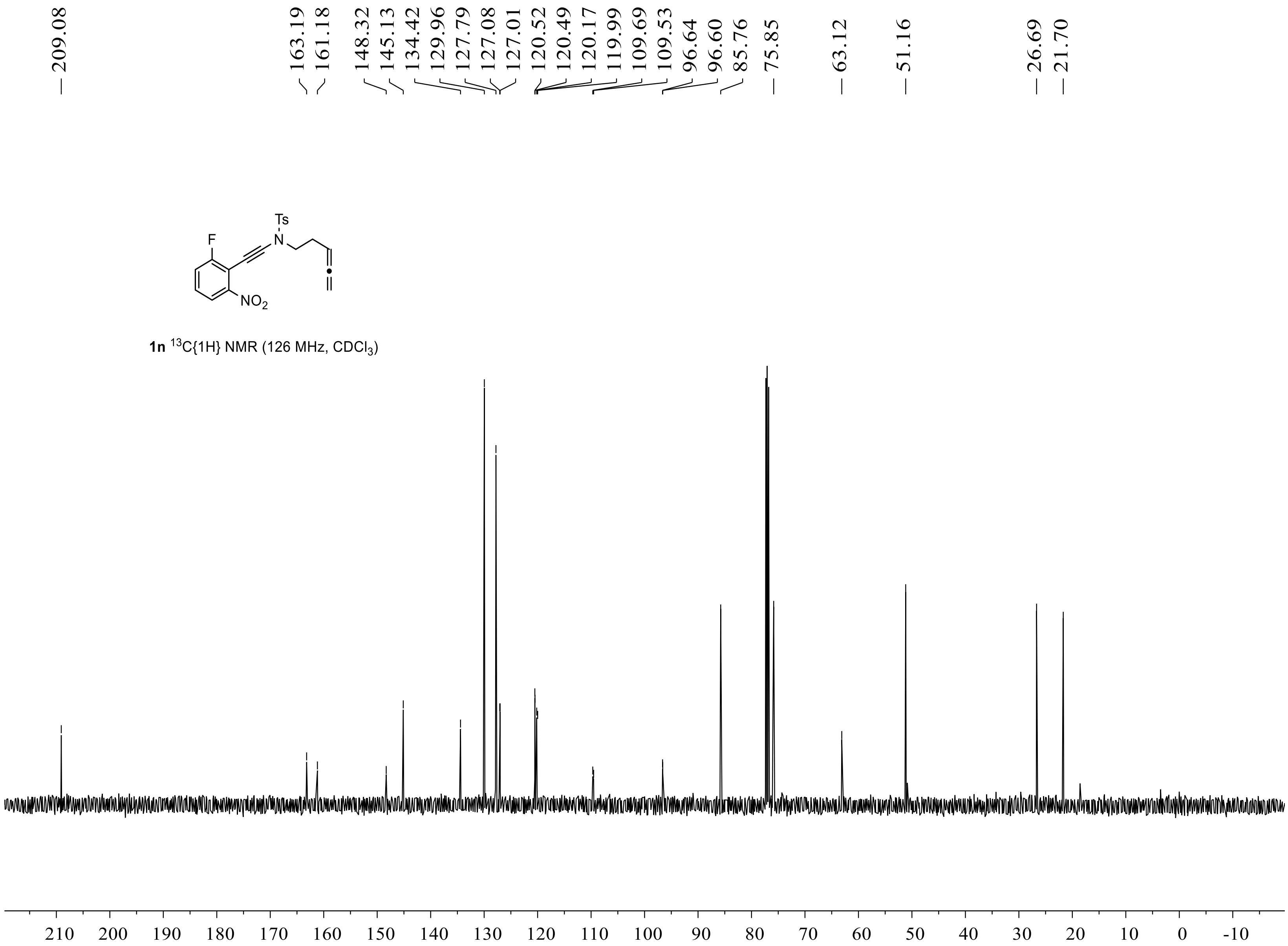

Supplementary Figure 45. ^{13}C NMR ($CDCl_3$, 126 MHz, 298 K) spectrum for **1n**

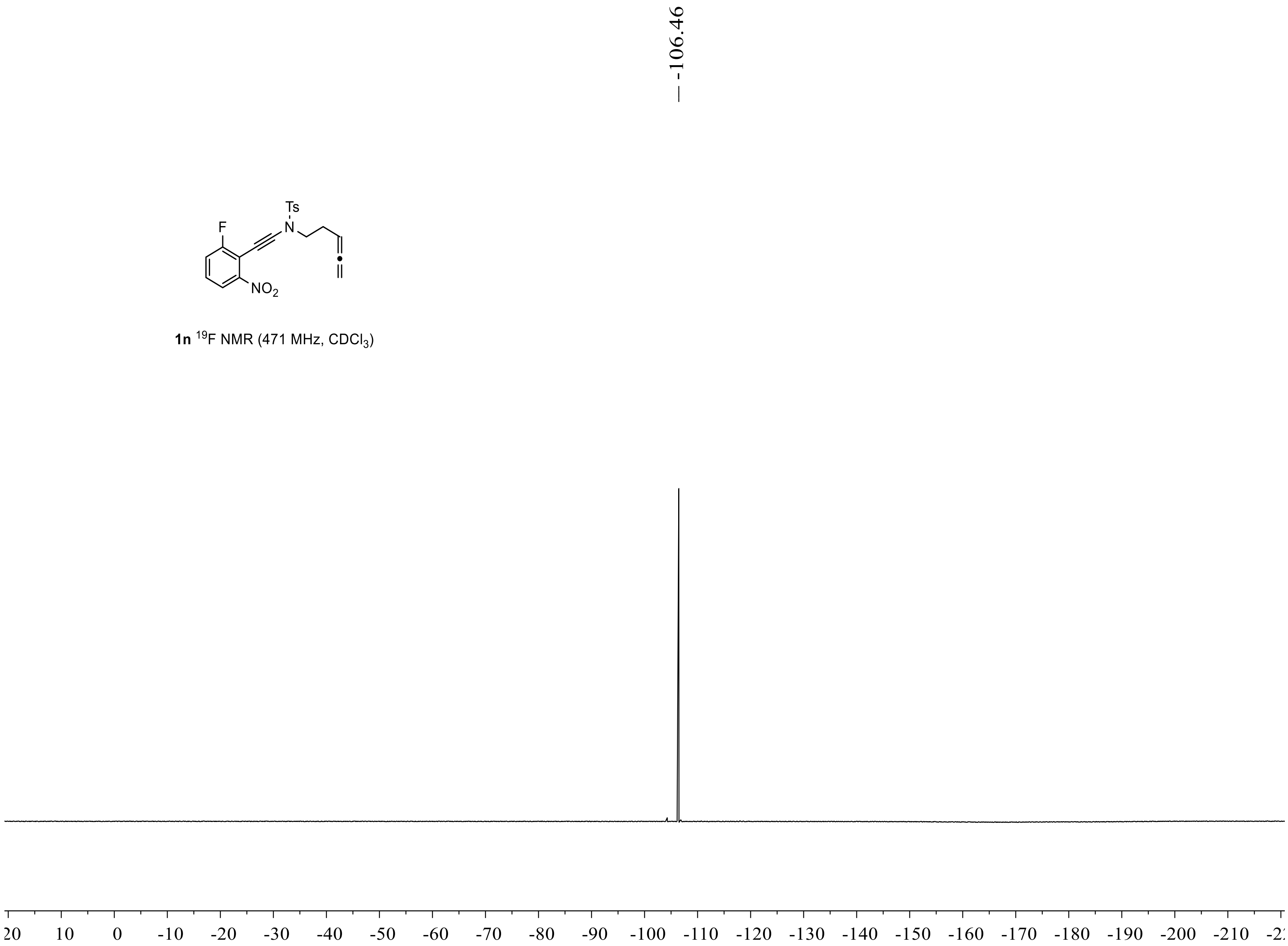

Supplementary Figure 46. ^{19}F NMR ($CDCl_3$, 471 MHz, 298 K) spectrum for **1n**

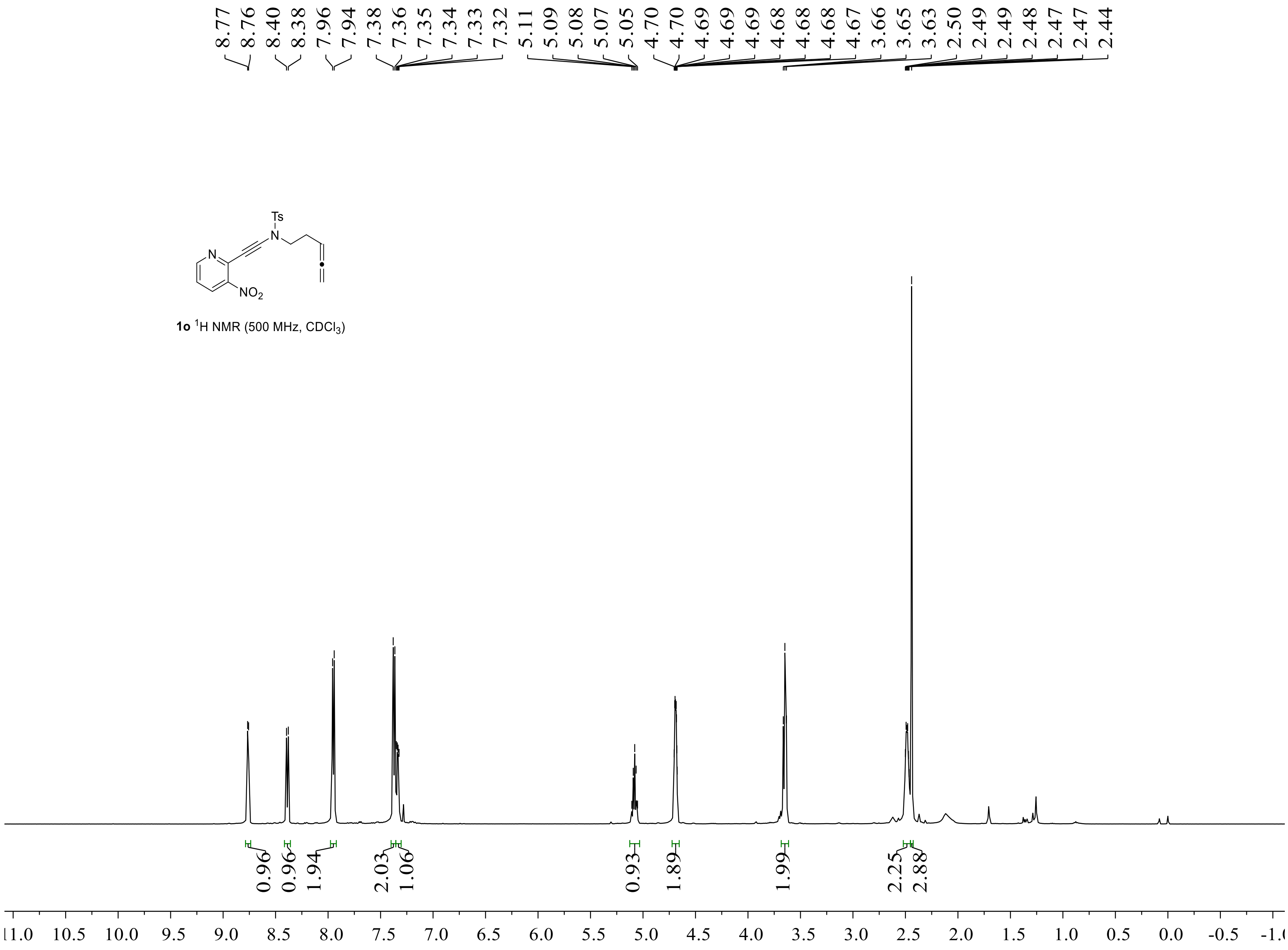

Supplementary Figure 47. ^{1}H NMR ($CDCl_3$, 500 MHz, 298 K) spectrum for **1o**

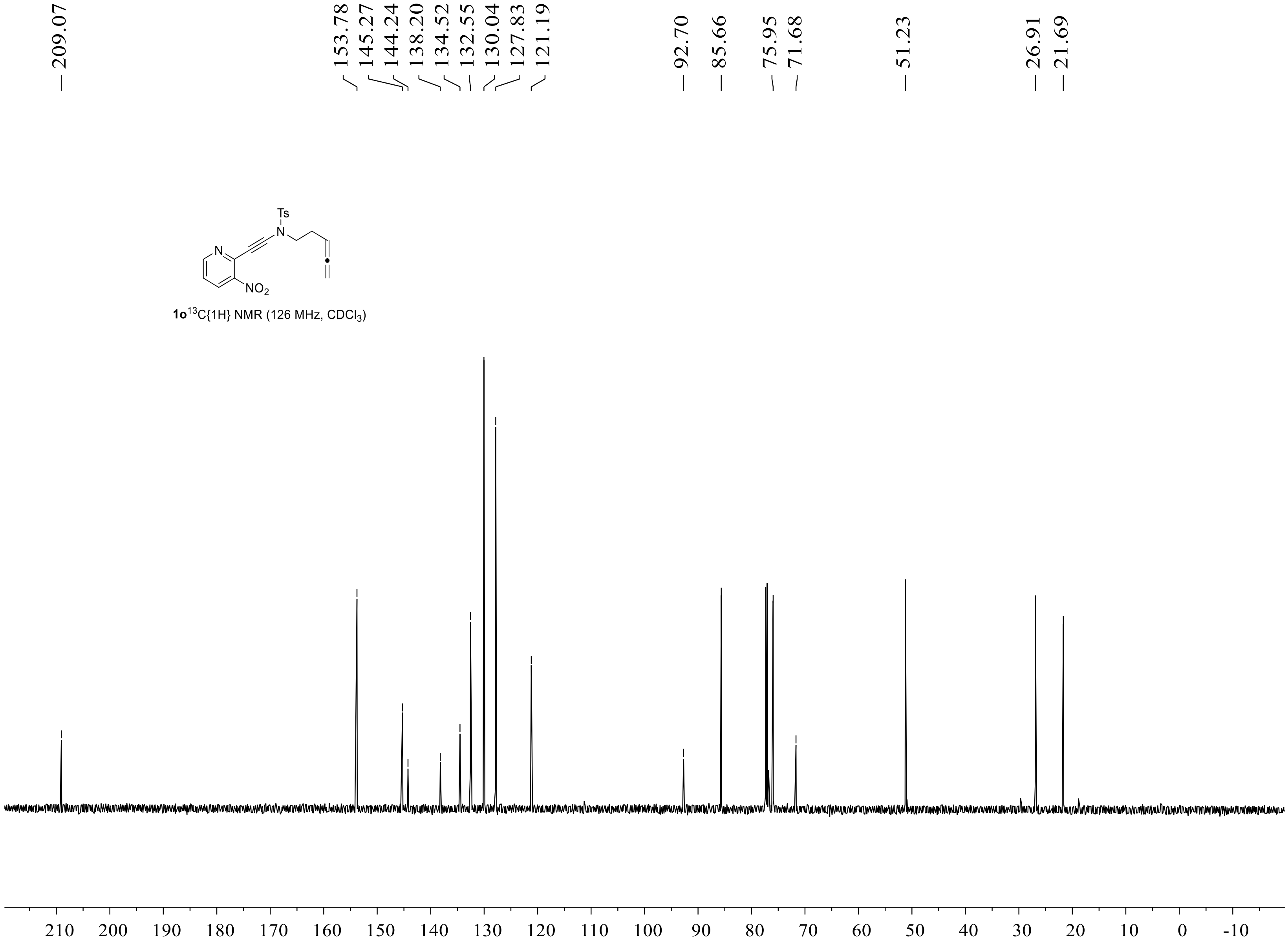

Supplementary Figure 48. ^{13}C NMR ($CDCl_3$, 126 MHz, 298 K) spectrum for **1o**

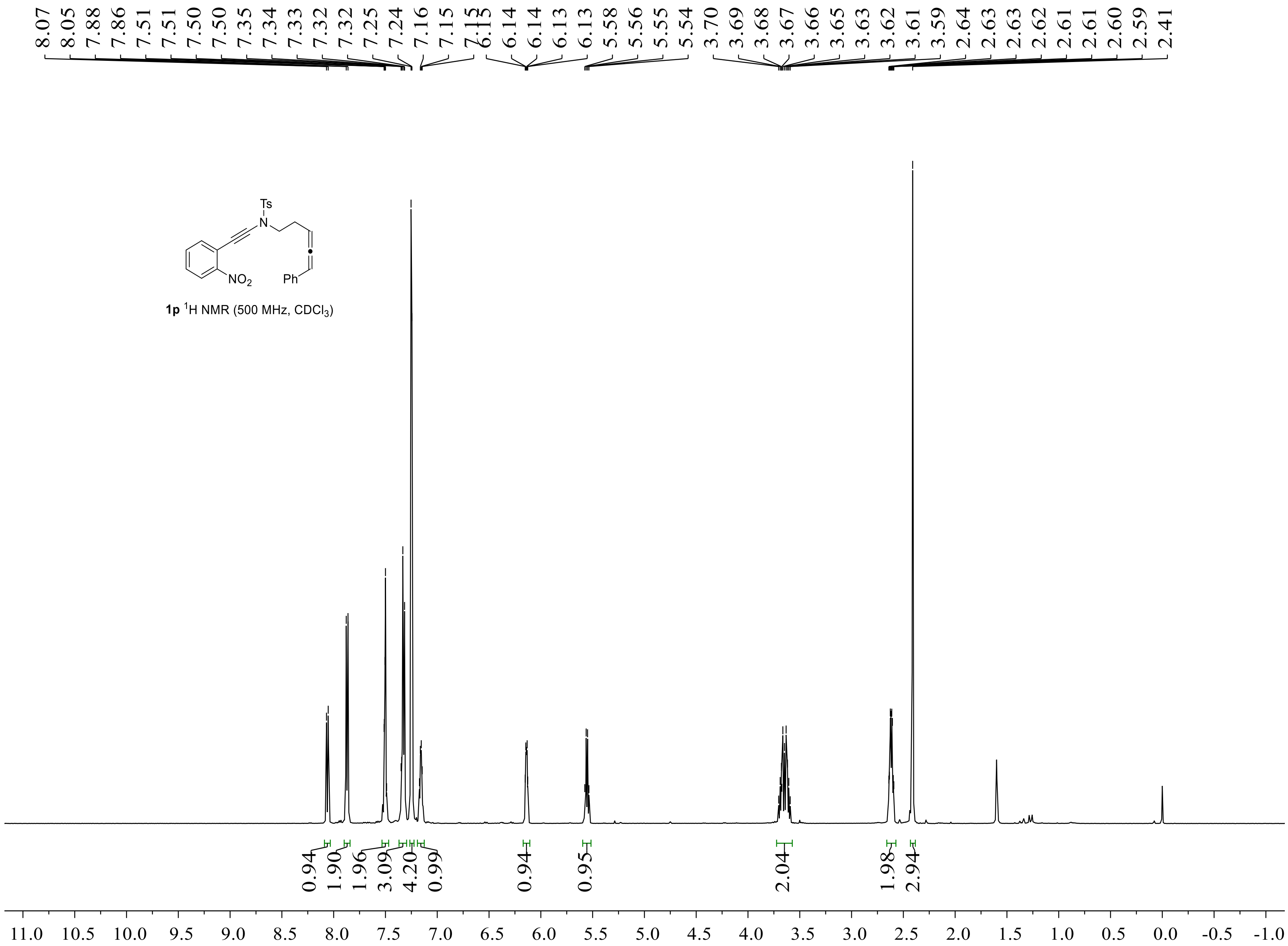

Supplementary Figure 49. ^{1}H NMR ($CDCl_3$, 500 MHz, 298 K) spectrum for **1p**

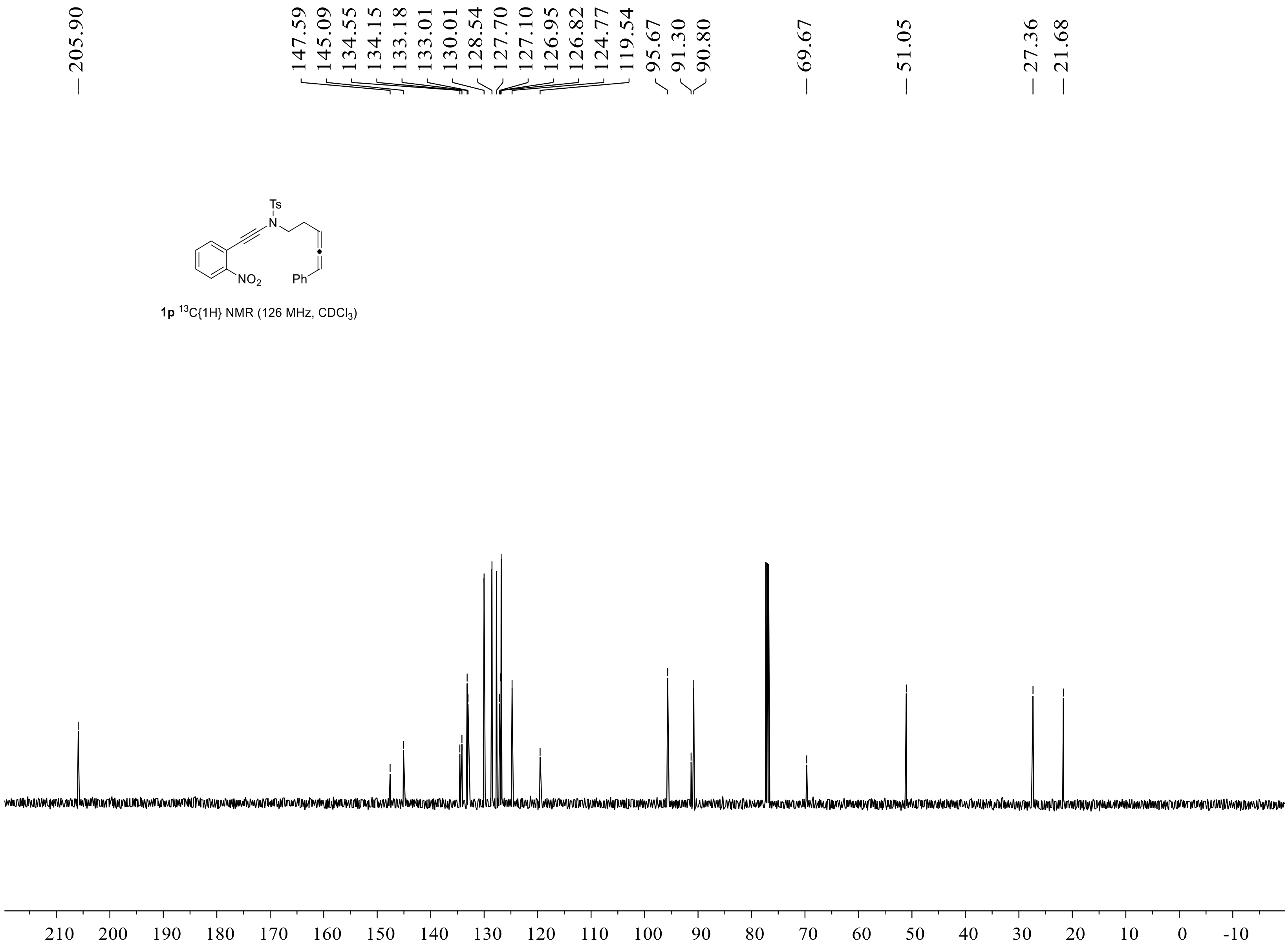

Supplementary Figure 50. ^{13}C NMR ($CDCl_3$, 126 MHz, 298 K) spectrum for **1p**

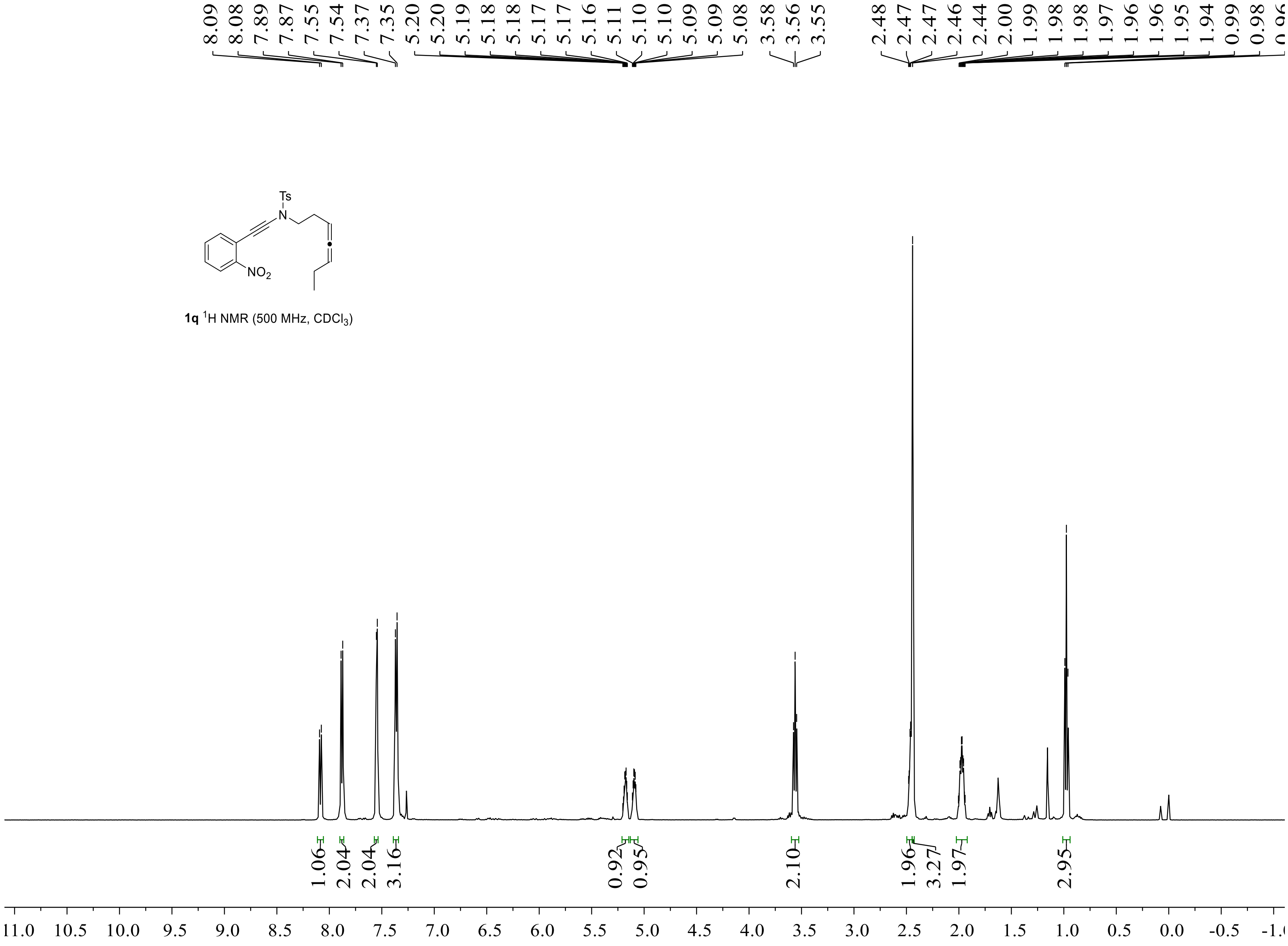

Supplementary Figure 51. ^{1}H NMR ($CDCl_3$, 500 MHz, 298 K) spectrum for **1q**

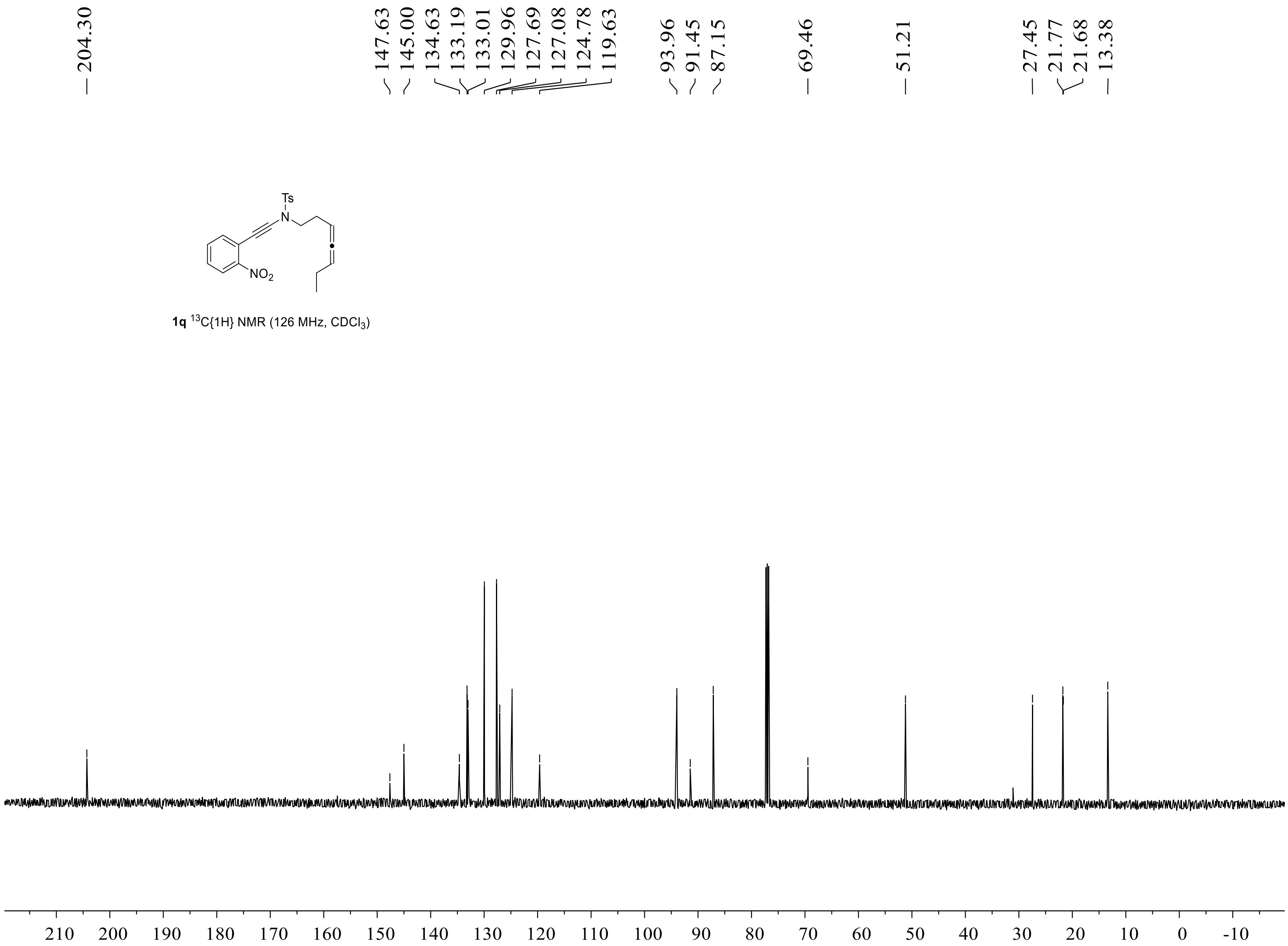

Supplementary Figure 52. ^{13}C NMR ($CDCl_3$, 126 MHz, 298 K) spectrum for **1q**

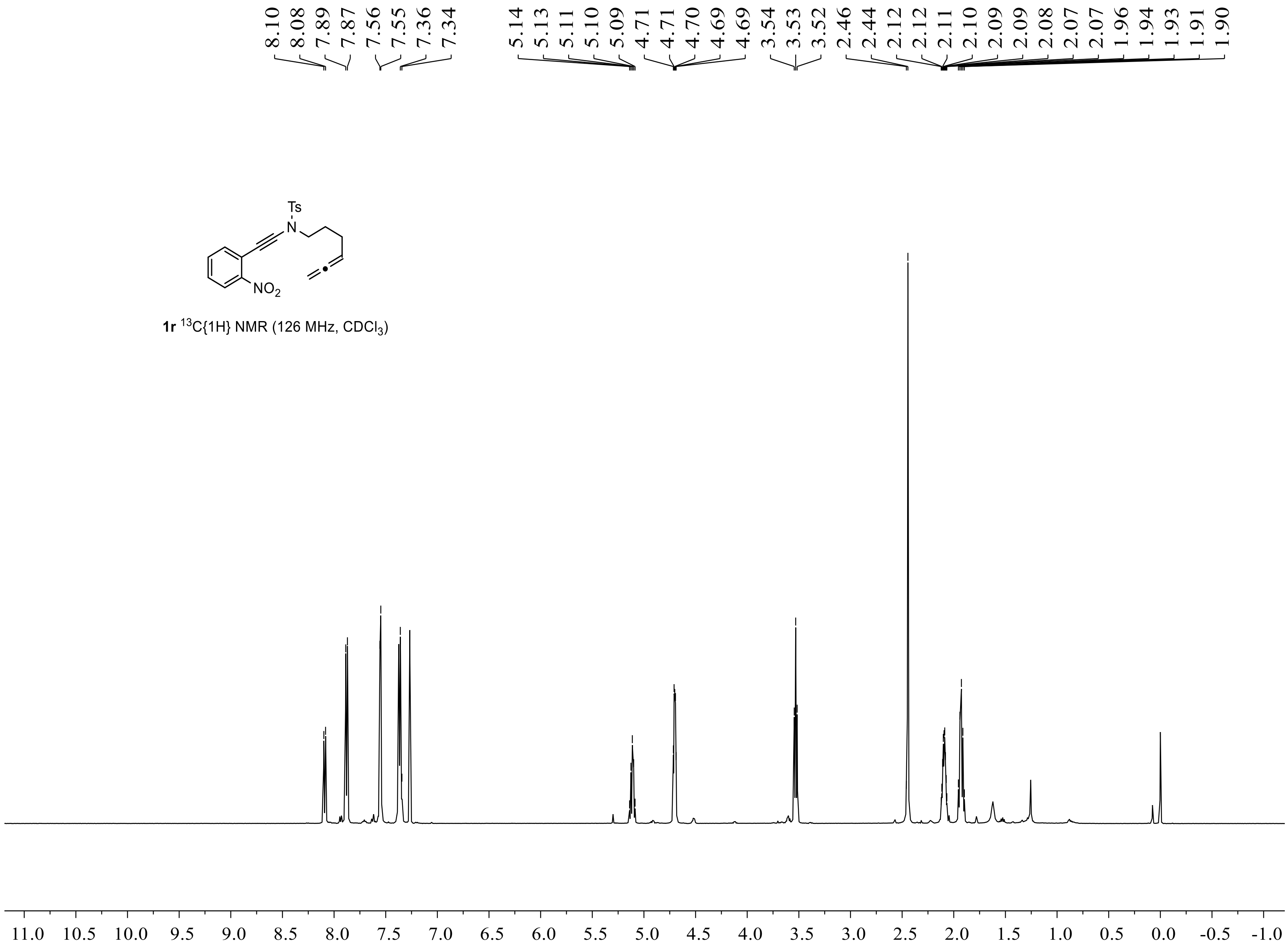

Supplementary Figure 53. ^{1}H NMR ($CDCl_3$, 500 MHz, 298 K) spectrum for **1r**

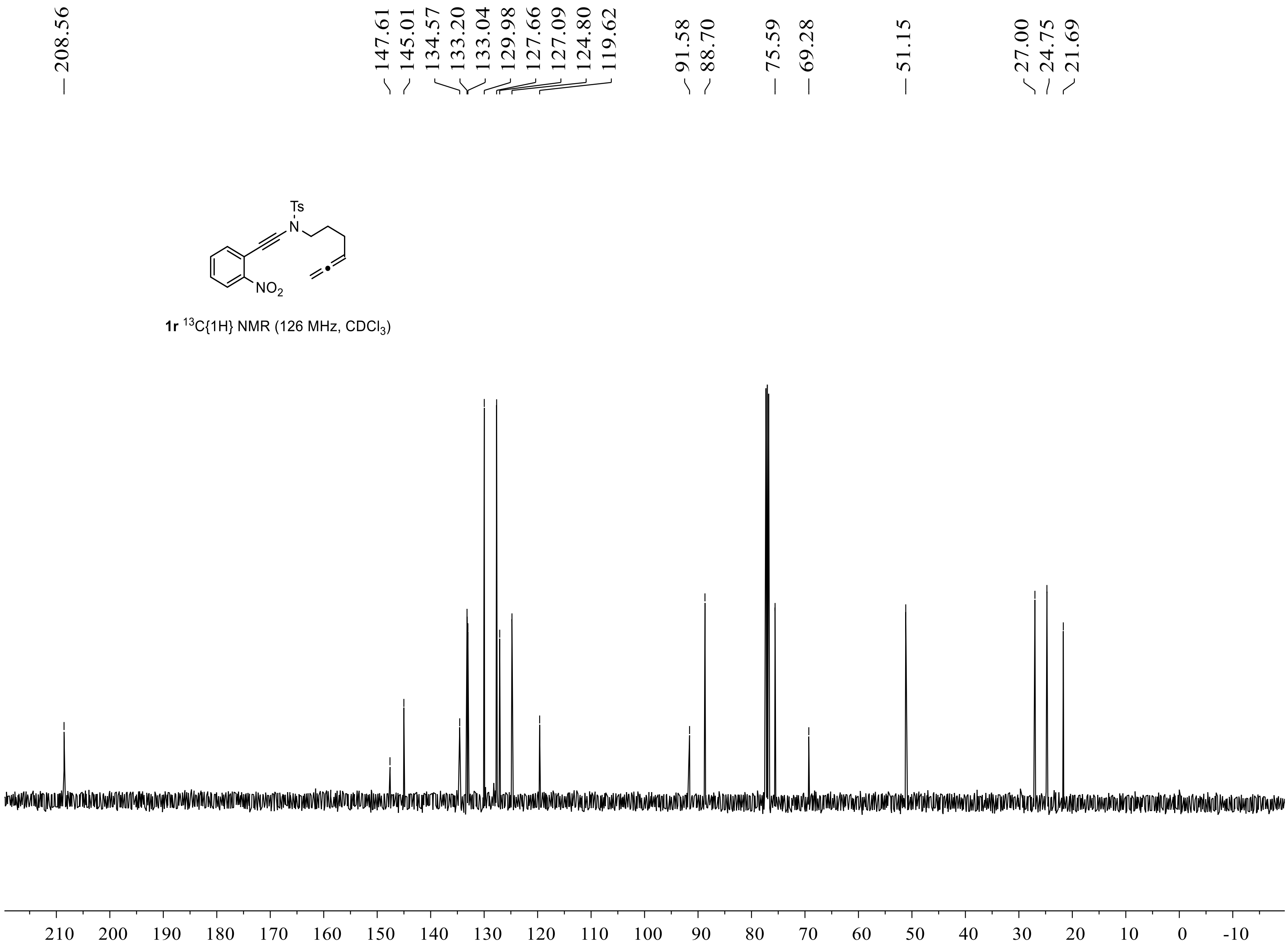

Supplementary Figure 54. ^{13}C NMR ($CDCl_3$, 126 MHz, 298 K) spectrum for **1r**

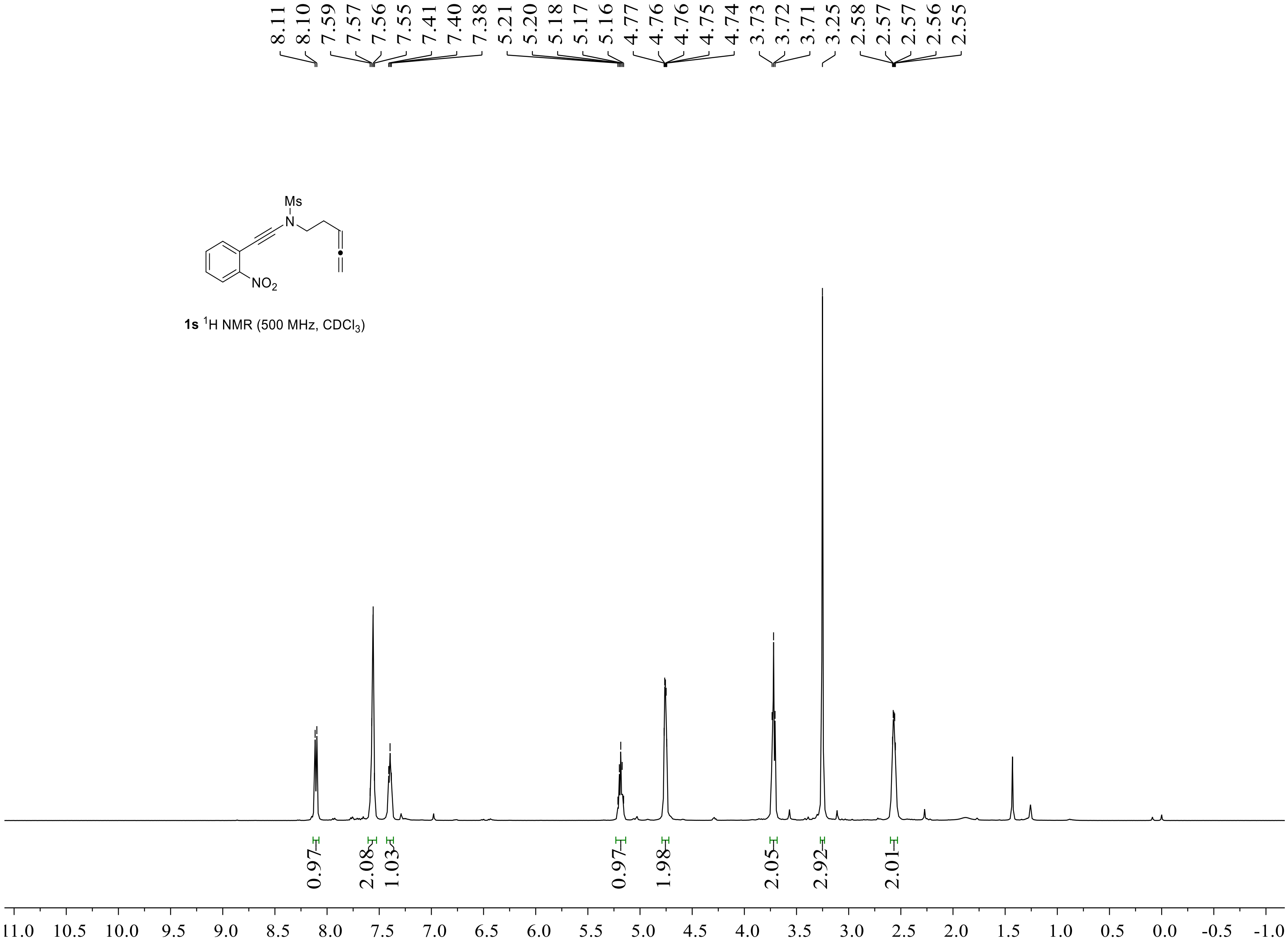

Supplementary Figure 55. ^{1}H NMR ($CDCl_3$, 500 MHz, 298 K) spectrum for **1s**

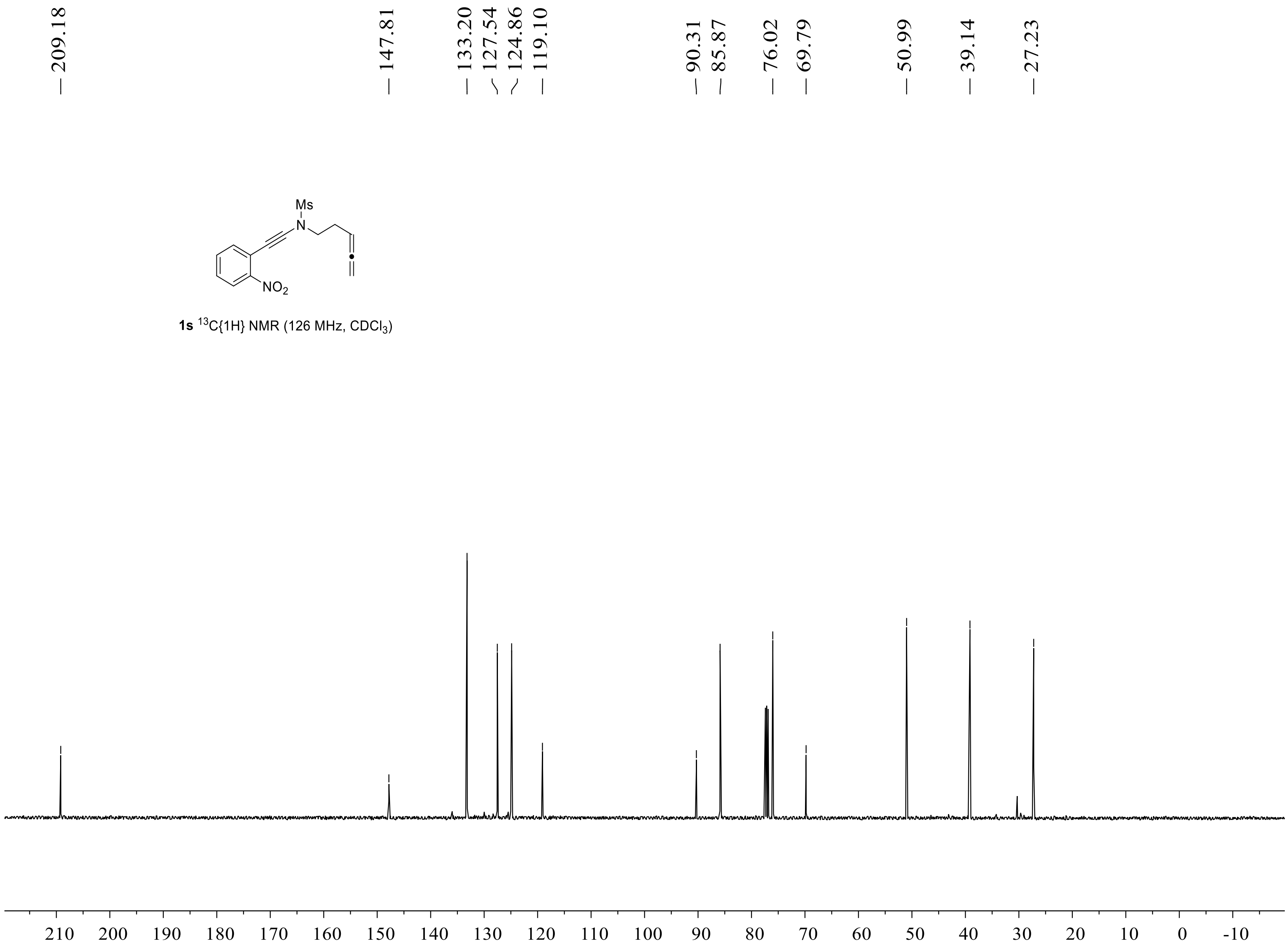

Supplementary Figure 56. ^{13}C NMR ($CDCl_3$, 126 MHz, 298 K) spectrum for **1s**

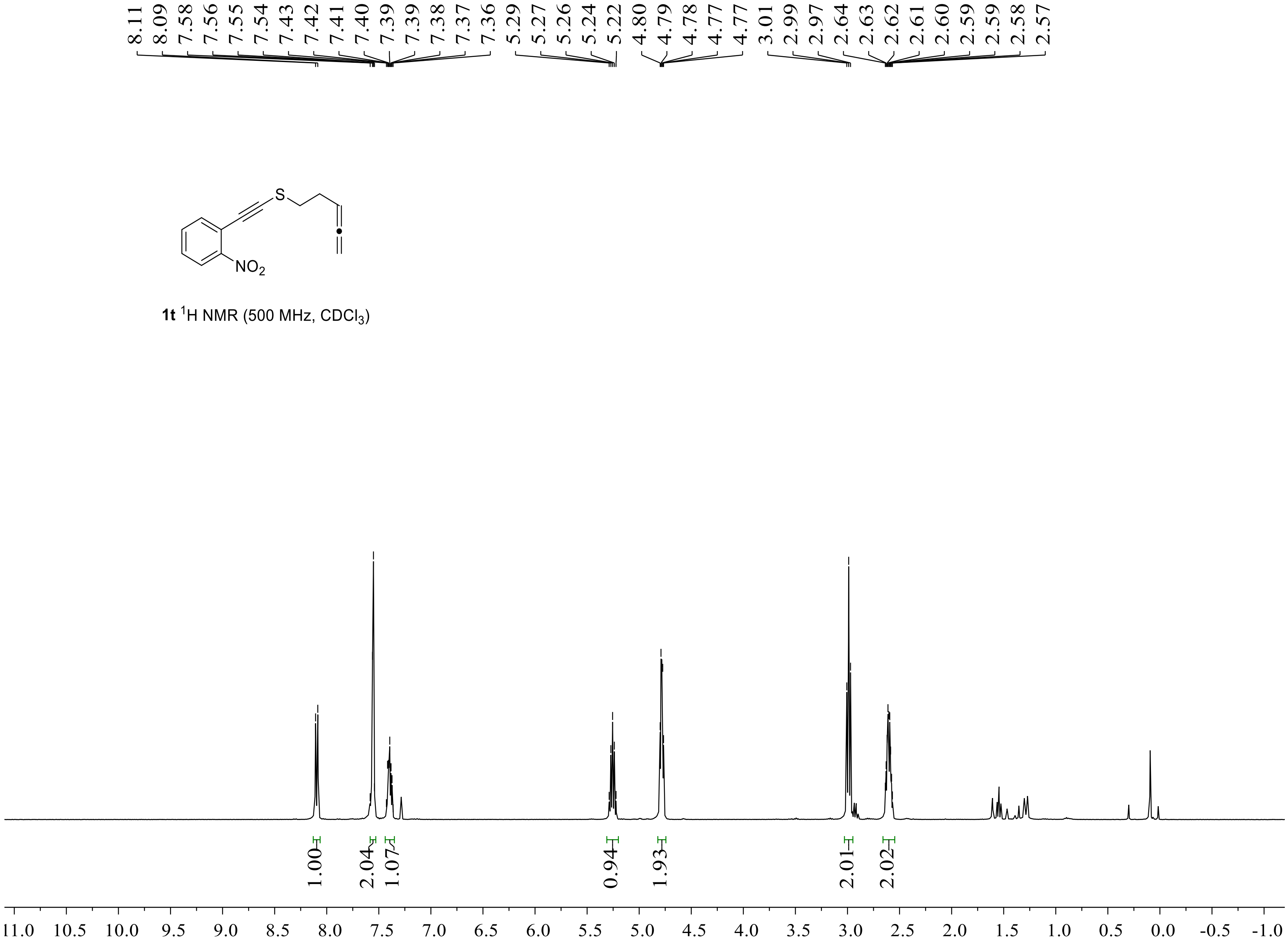

Supplementary Figure 57. ^{1}H NMR ($CDCl_3$, 500 MHz, 298 K) spectrum for **1t**

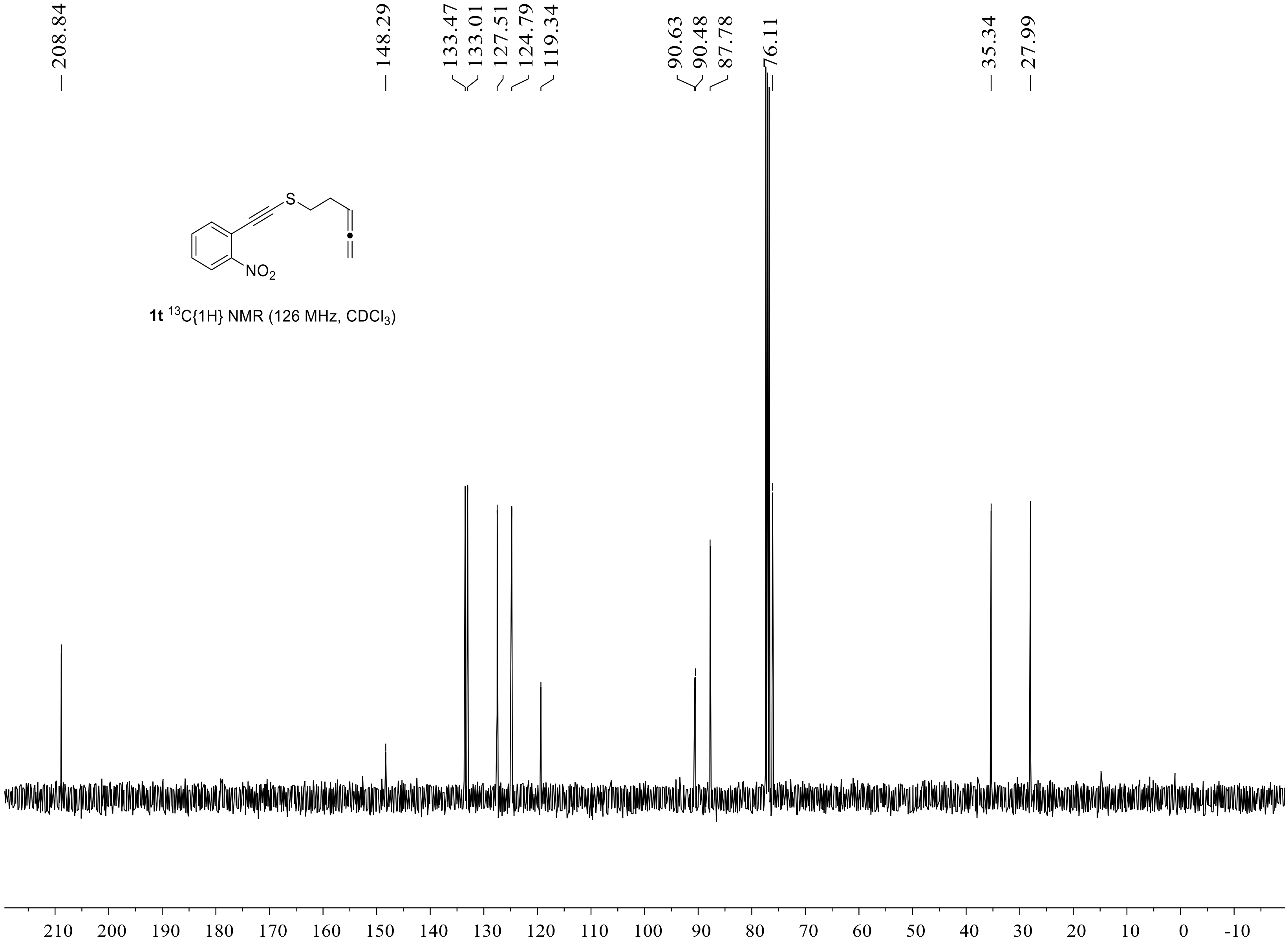

Supplementary Figure 58. ^{13}C NMR ($CDCl_3$, 126 MHz, 298 K) spectrum for **1t**

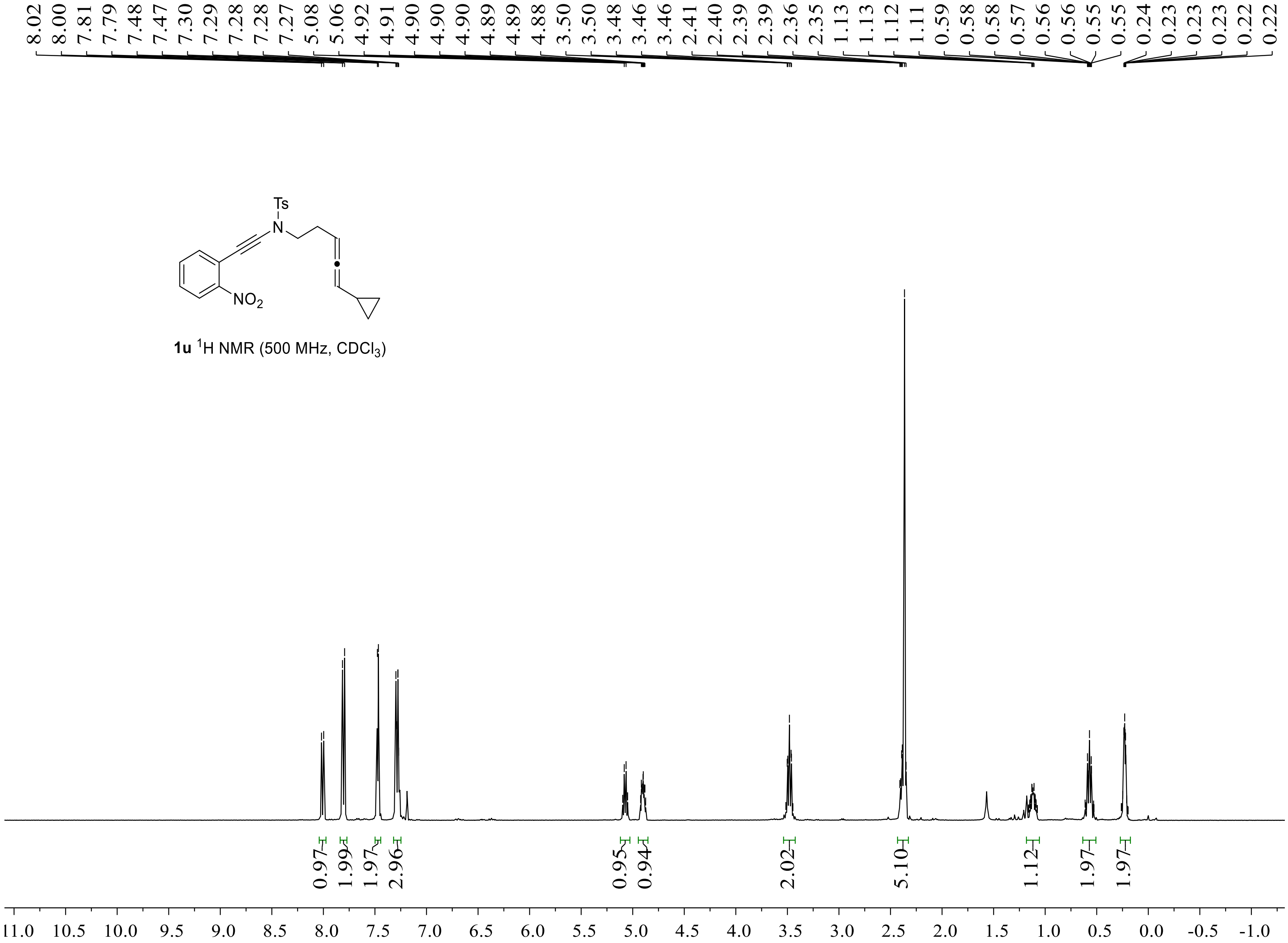

Supplementary Figure 59. ^{1}H NMR ($CDCl_3$, 500 MHz, 298 K) spectrum for **1u**

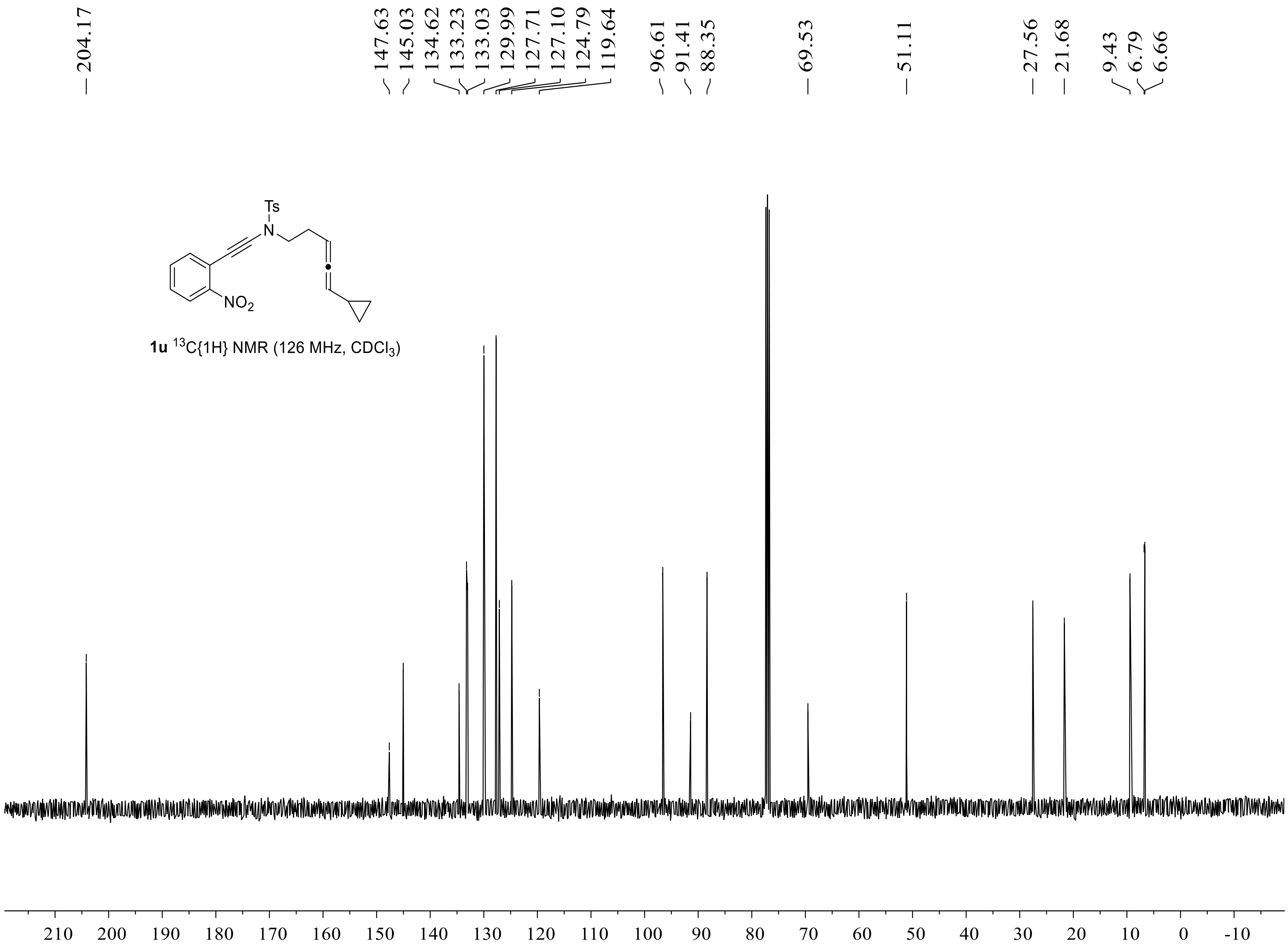

Supplementary Figure 60. ^{13}C NMR ($CDCl_3$, 126 MHz, 298 K) spectrum for **1u**

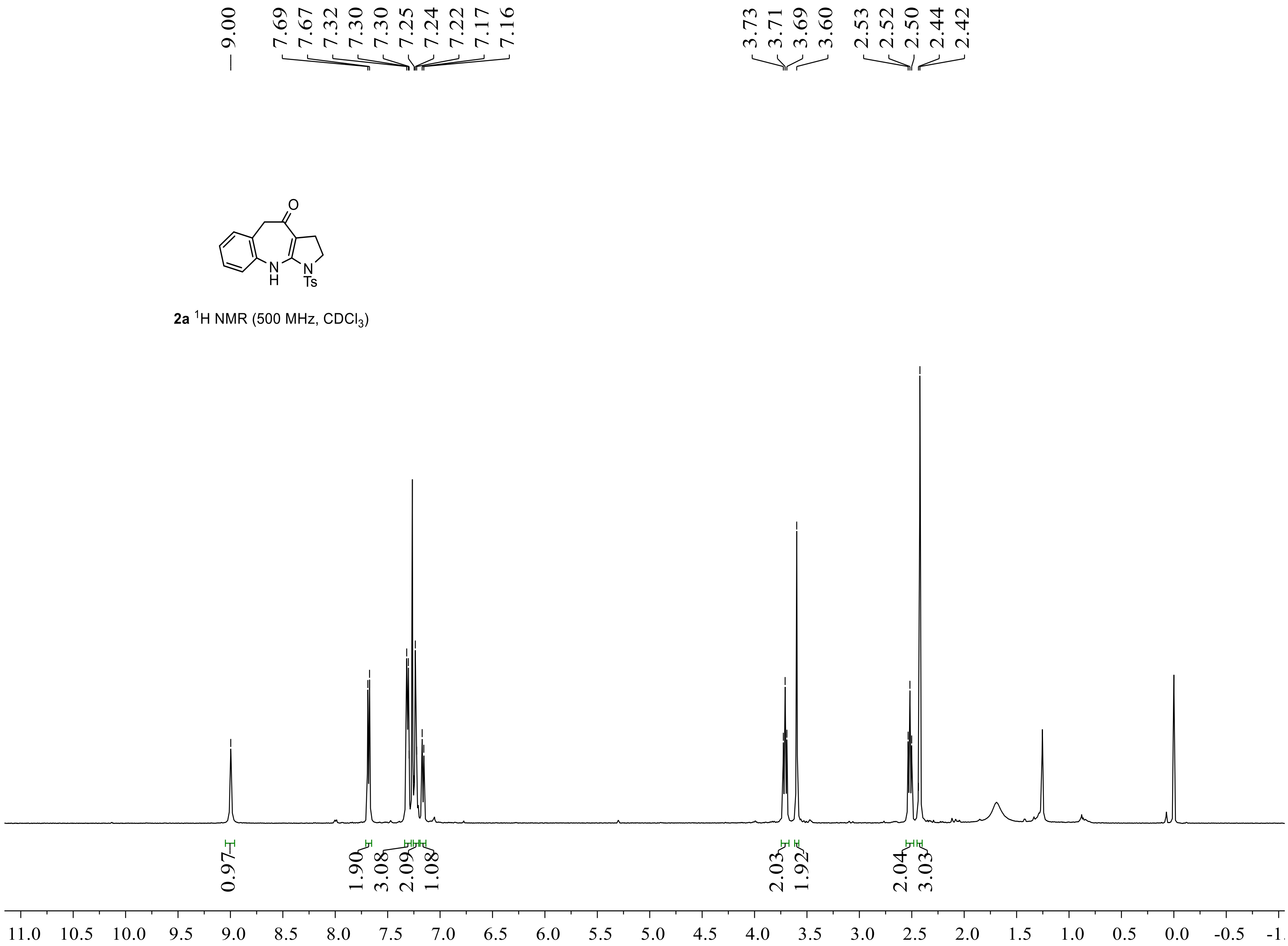

Supplementary Figure 61. ^{1}H NMR ($CDCl_3$, 500 MHz, 298 K) spectrum for **2a**

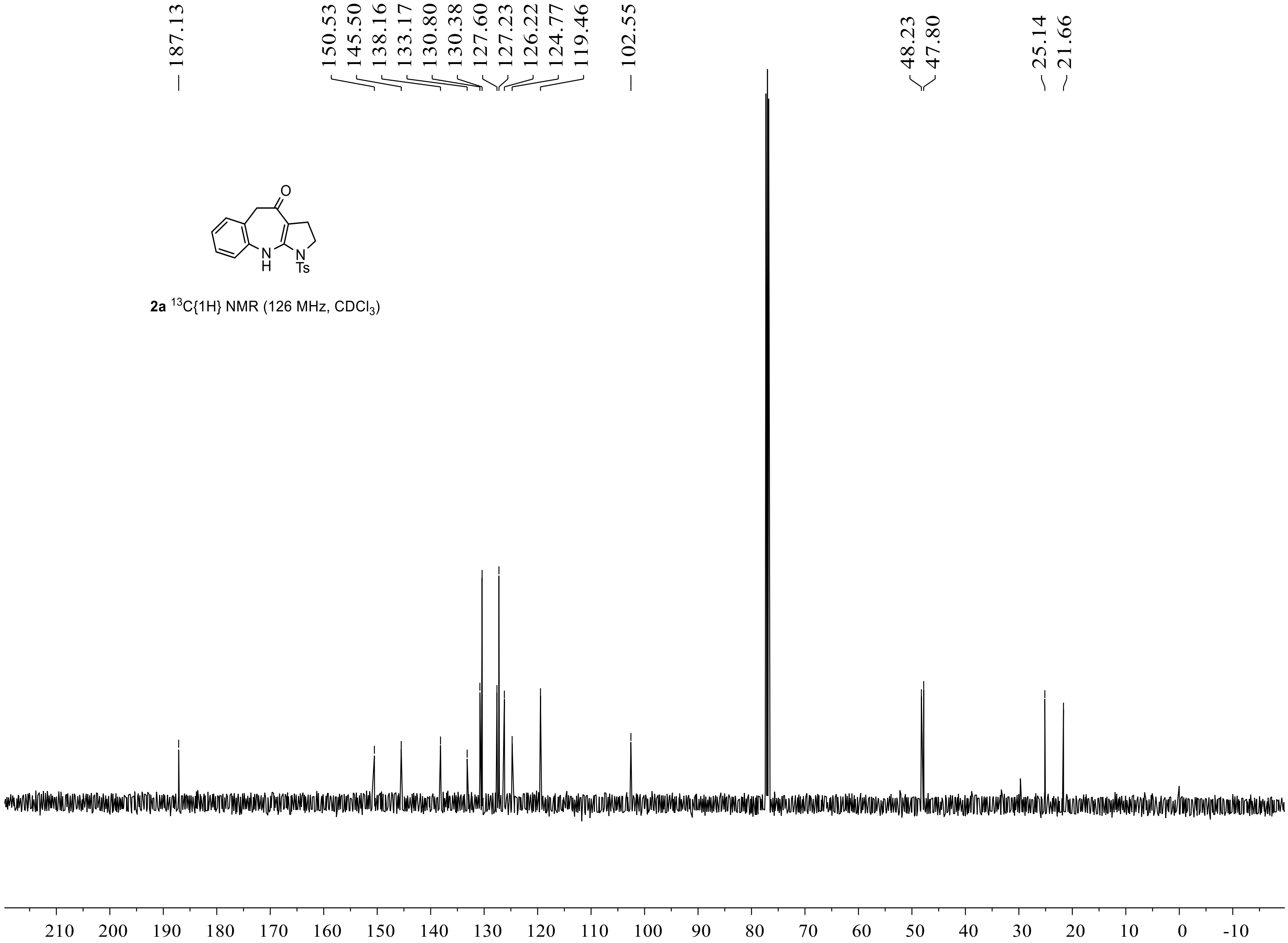

Supplementary Figure 62. ^{13}C NMR ($CDCl_3$, 126 MHz, 298 K) spectrum for **2a**

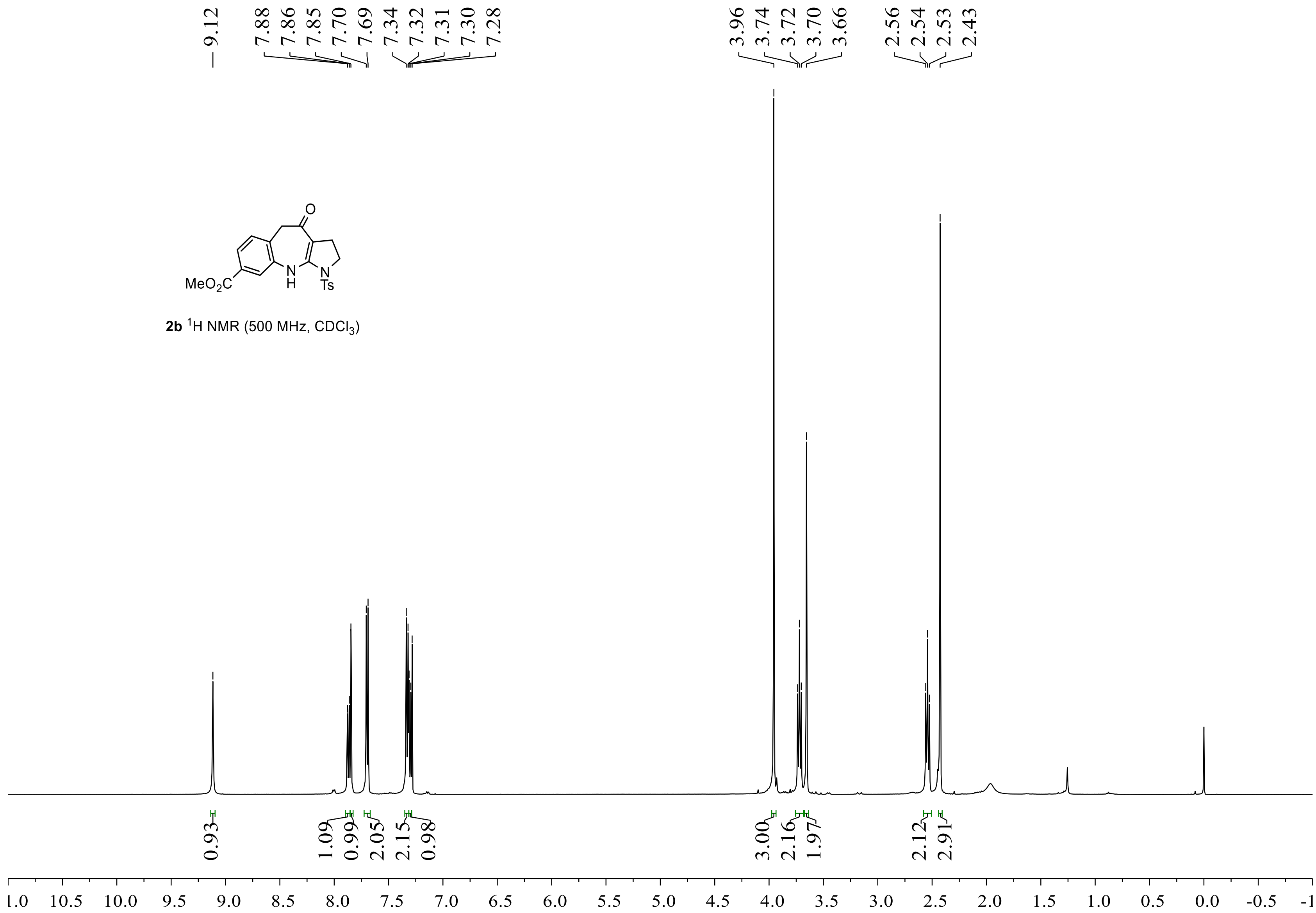

Supplementary Figure 63. ^{1}H NMR ($CDCl_3$, 500 MHz, 298 K) spectrum for **2b**

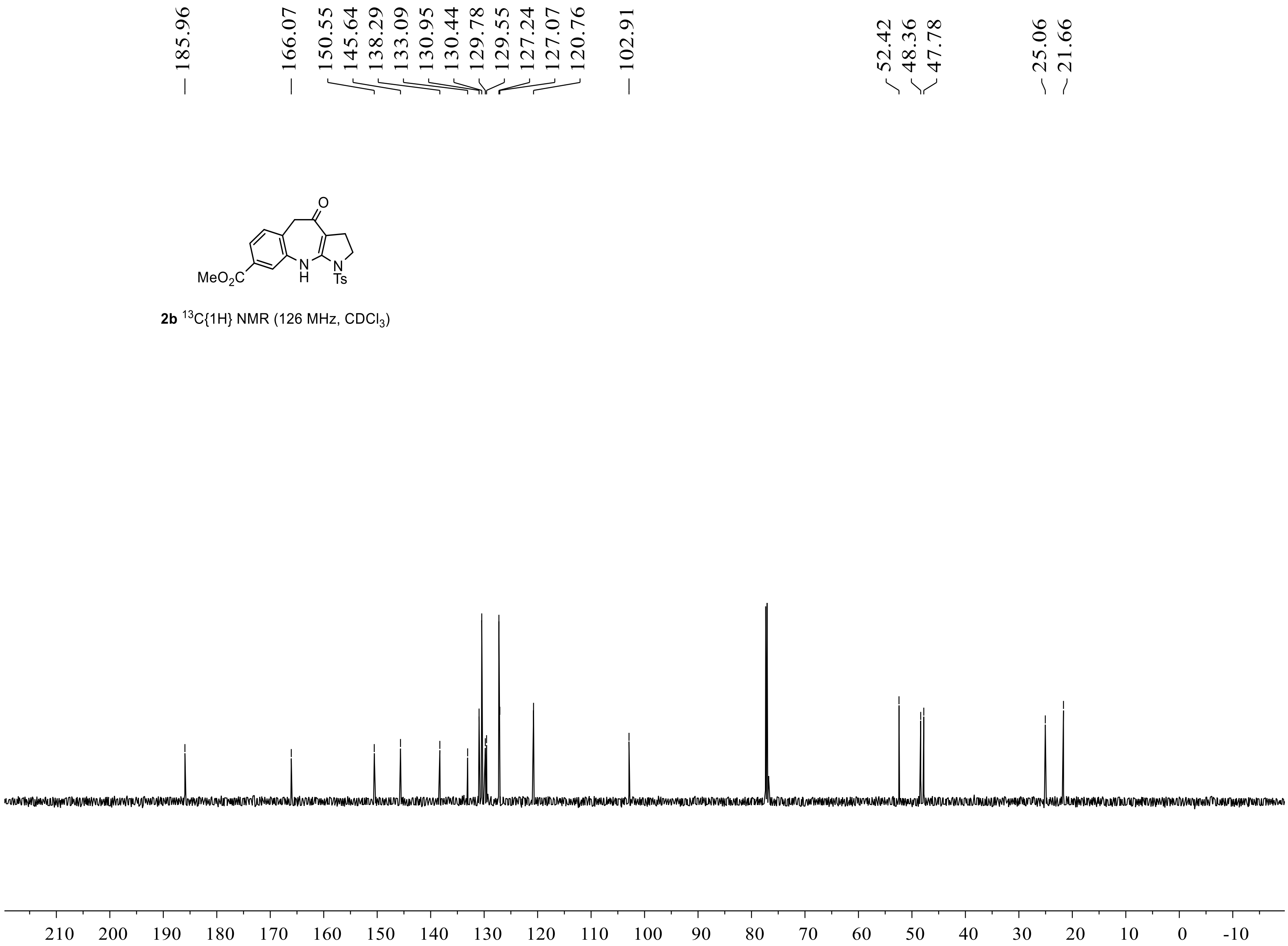

Supplementary Figure 64. ^{13}C NMR ($CDCl_3$, 126 MHz, 298 K) spectrum for **2b**

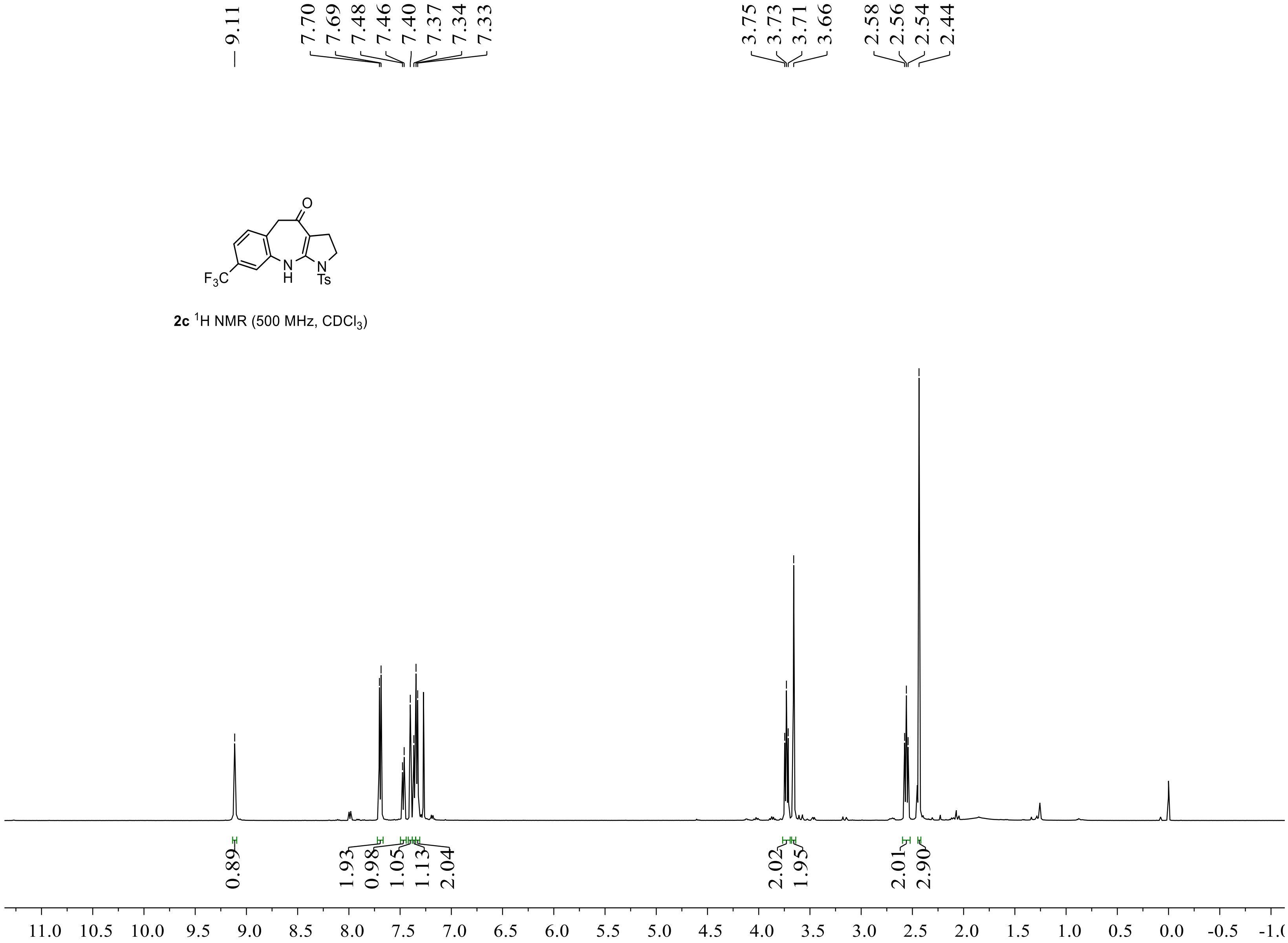

Supplementary Figure 65. ^{1}H NMR ($CDCl_3$, 500 MHz, 298 K) spectrum for **2c**

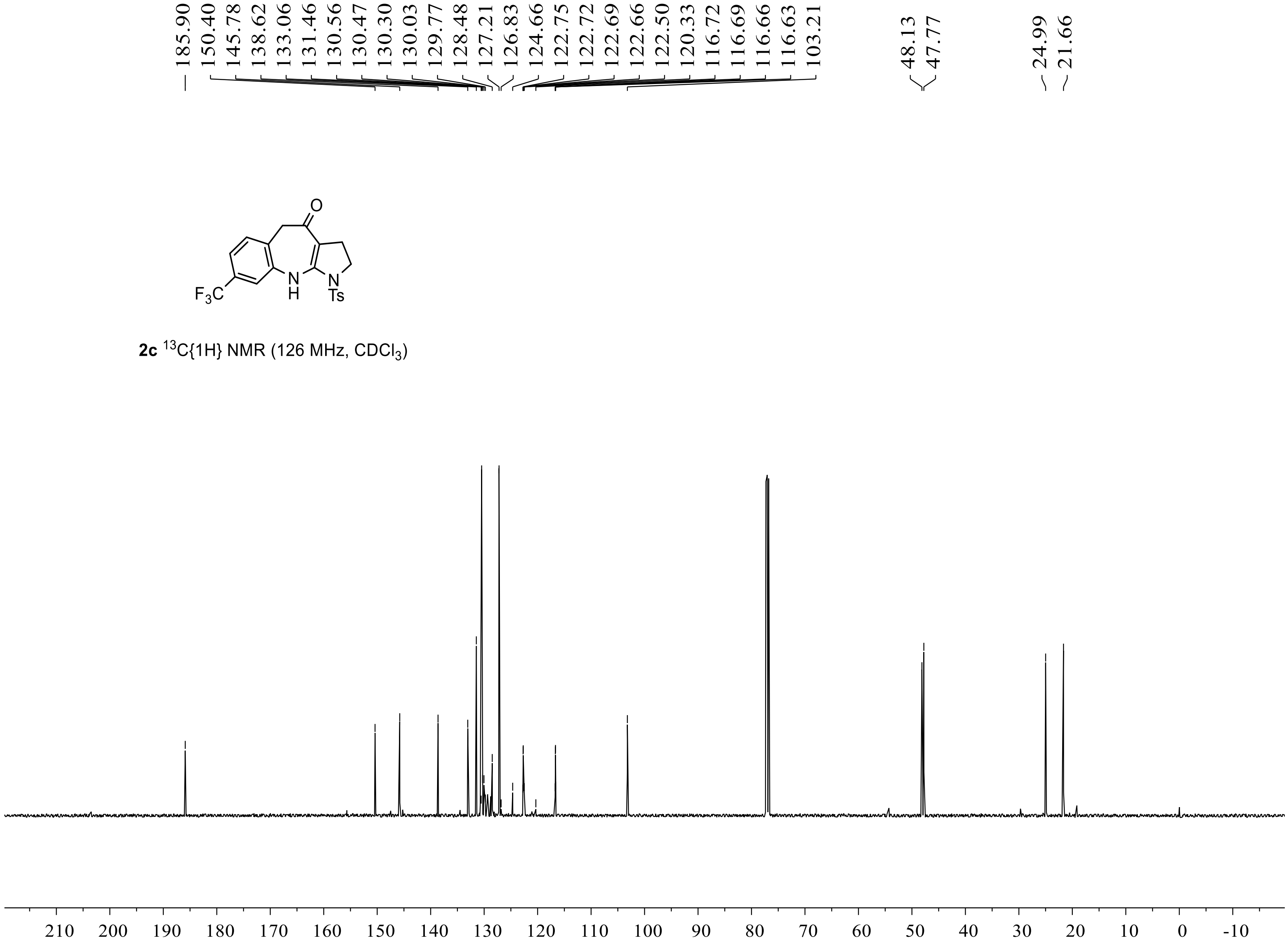

Supplementary Figure 66. ^{13}C NMR ($CDCl_3$, 126 MHz, 298 K) spectrum for **2c**

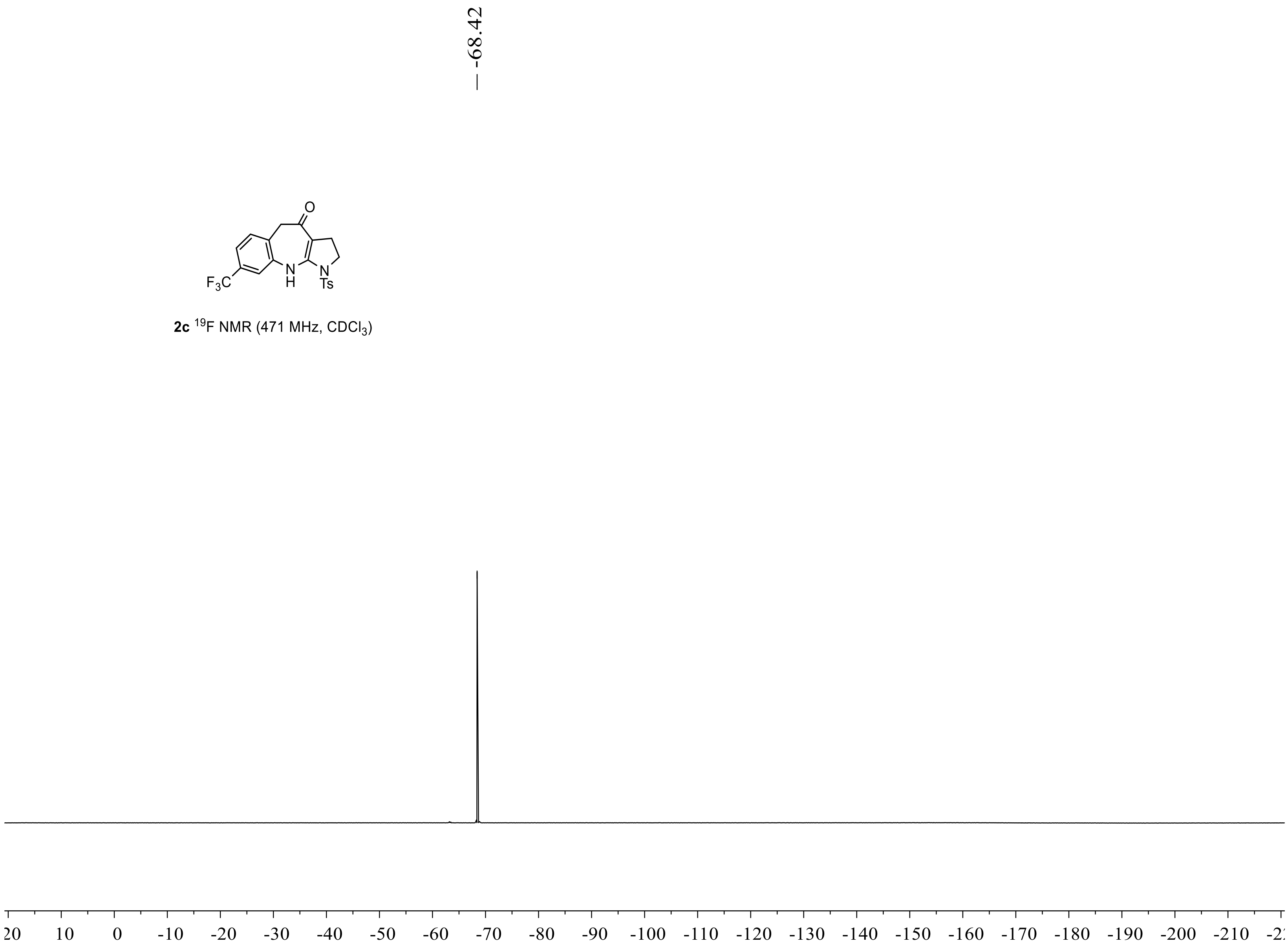

Supplementary Figure 67. **19**F NMR ($CDCl_3$, 471 MHz, 298 K) spectrum for **2c**

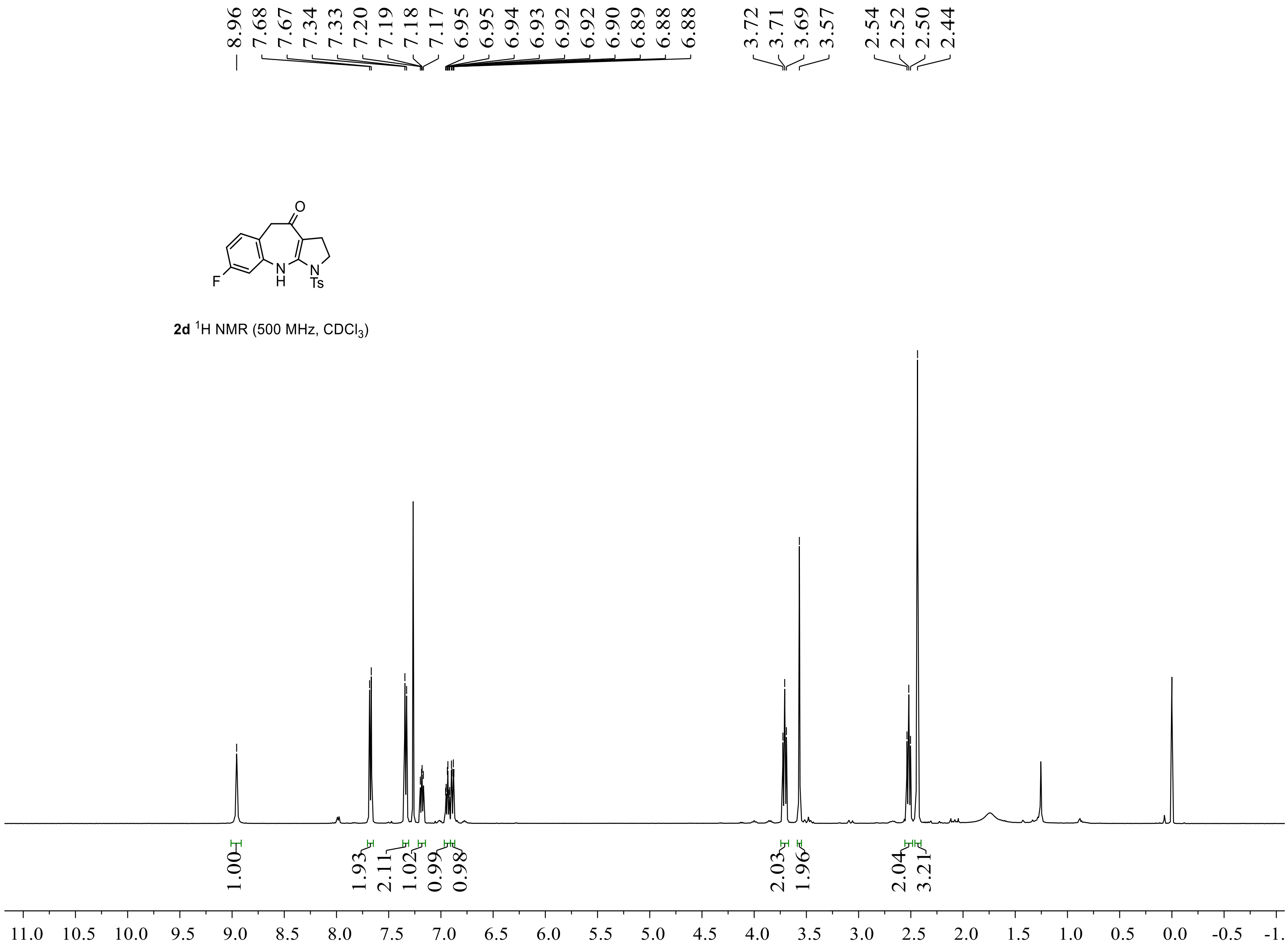

Supplementary Figure 68. ^{1}H NMR ($CDCl_3$, 500 MHz, 298 K) spectrum for **2d**

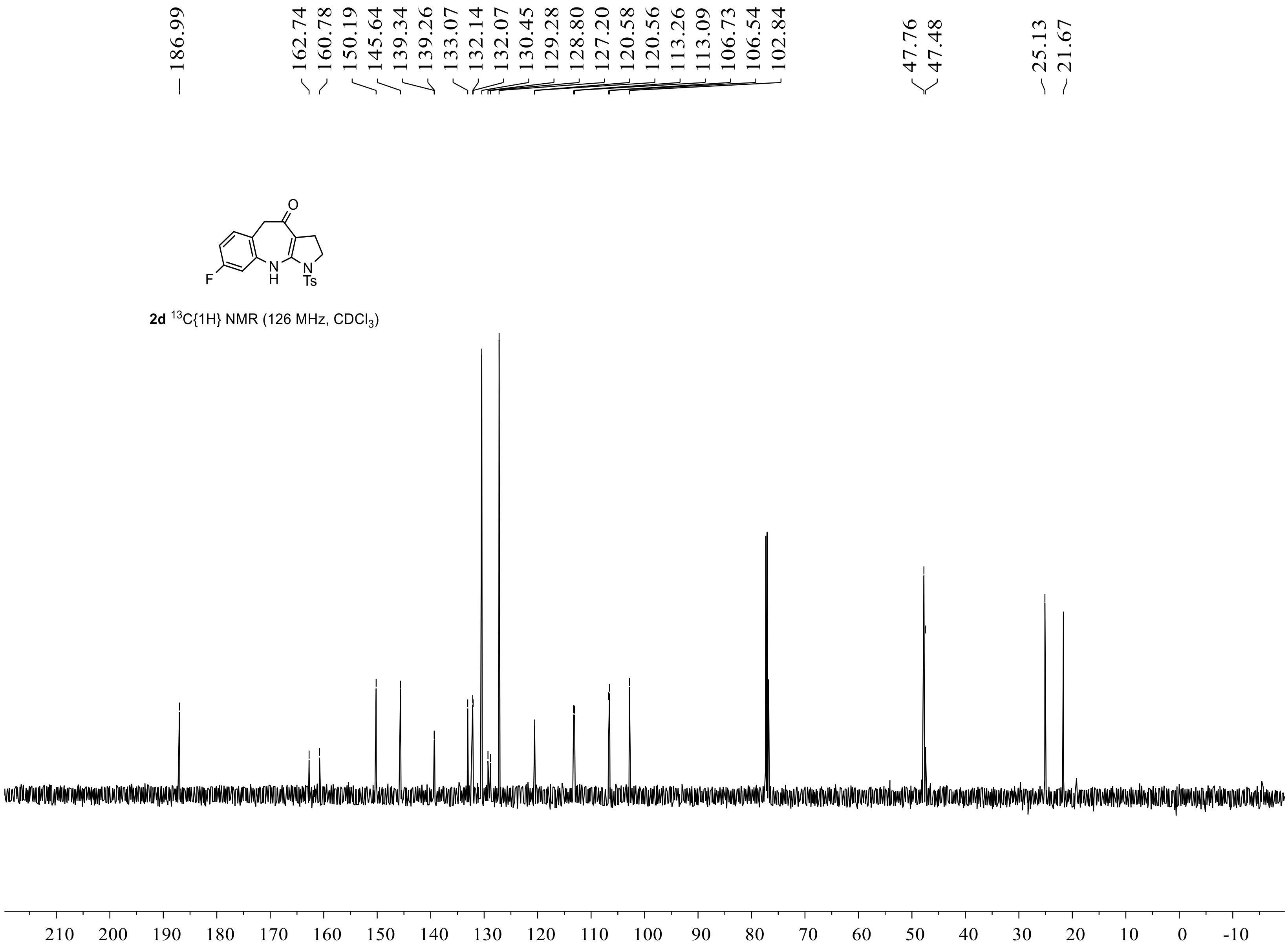

Supplementary Figure 69. ^{13}C NMR ($CDCl_3$, 126 MHz, 298 K) spectrum for **2d**

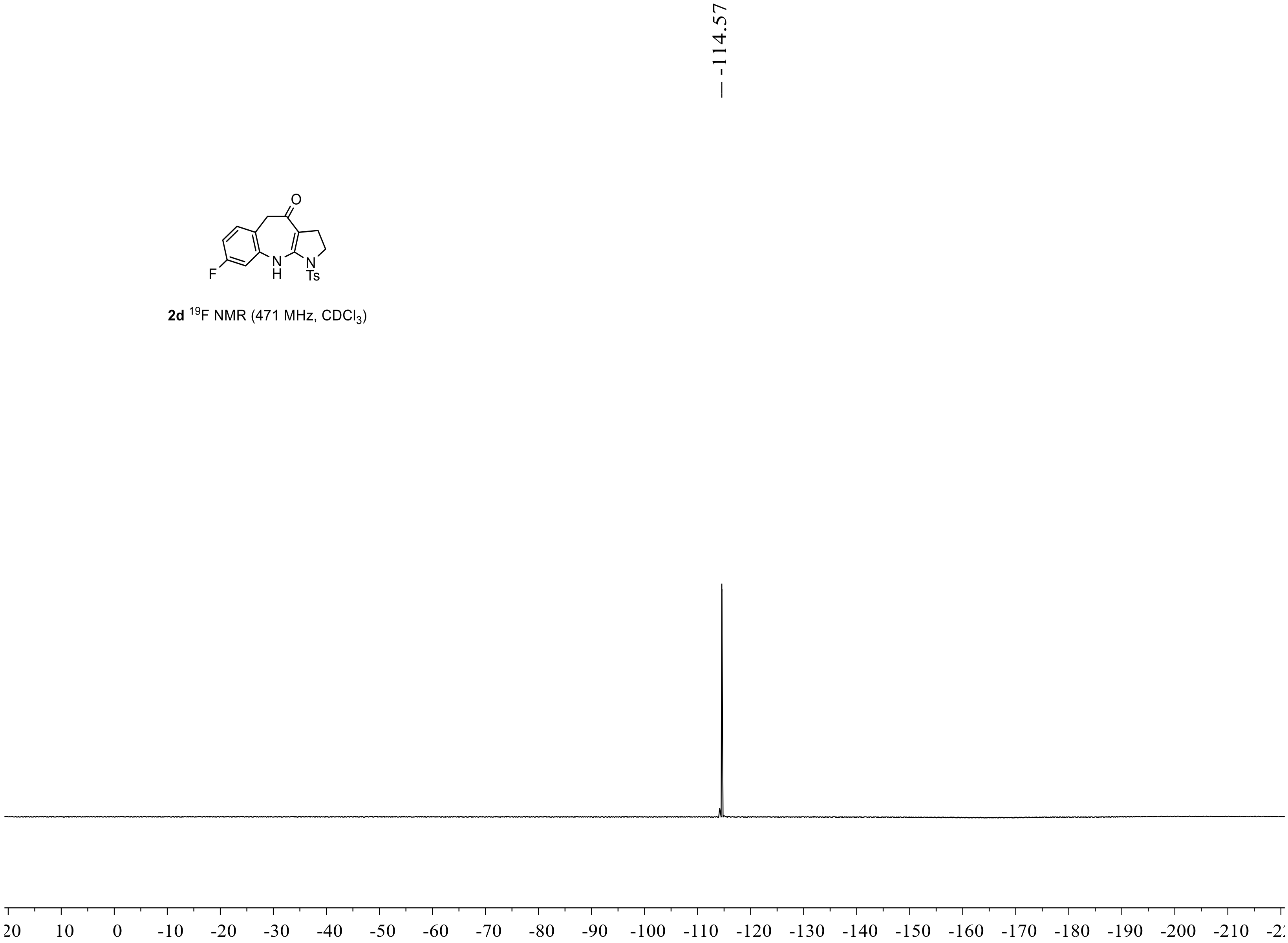

Supplementary Figure 70. ^{19}F NMR ($CDCl_3$, 471 MHz, 298 K) spectrum for **2d**

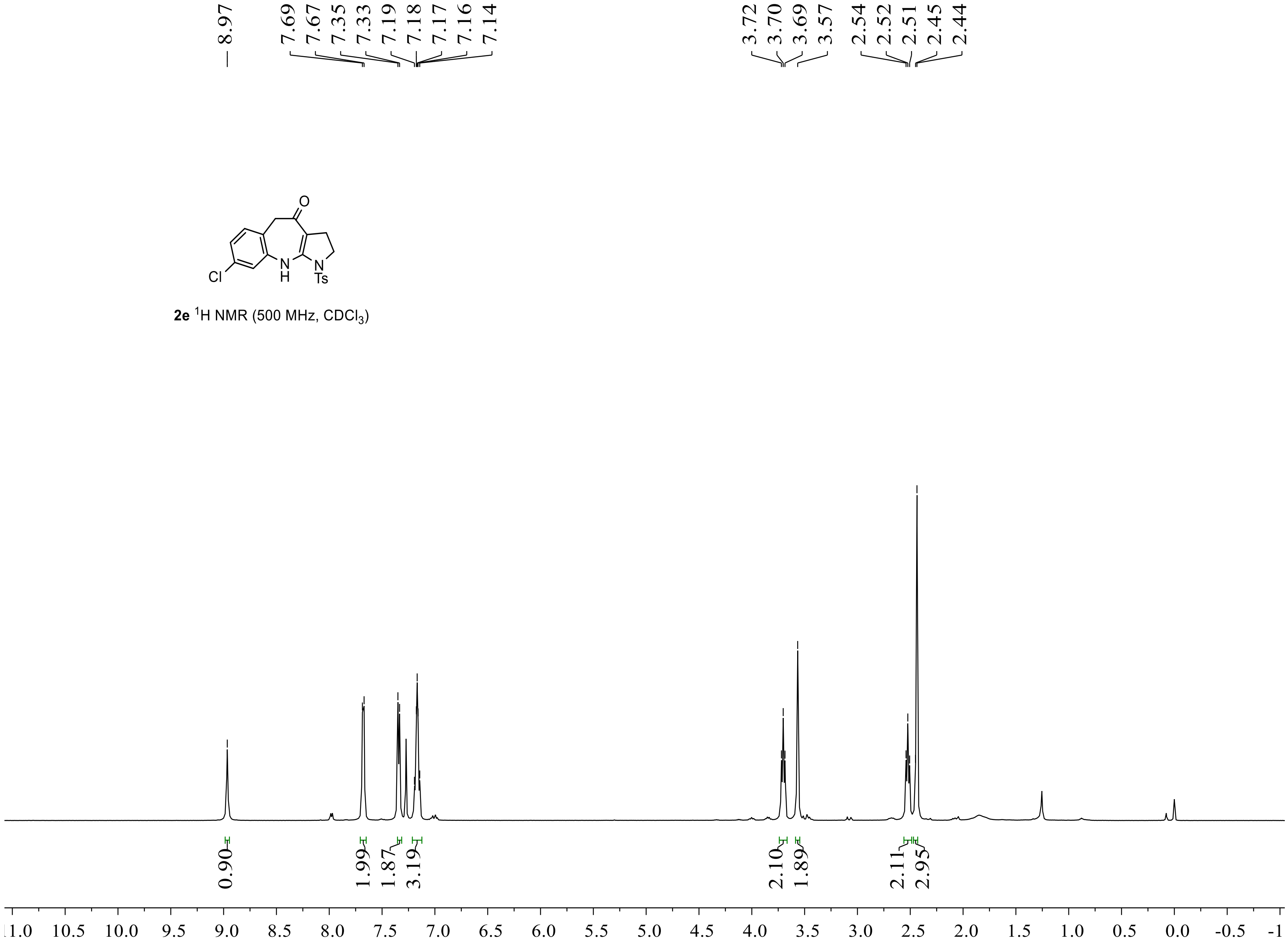

Supplementary Figure 71. ^{1}H NMR ($CDCl_3$, 500 MHz, 298 K) spectrum for **2e**

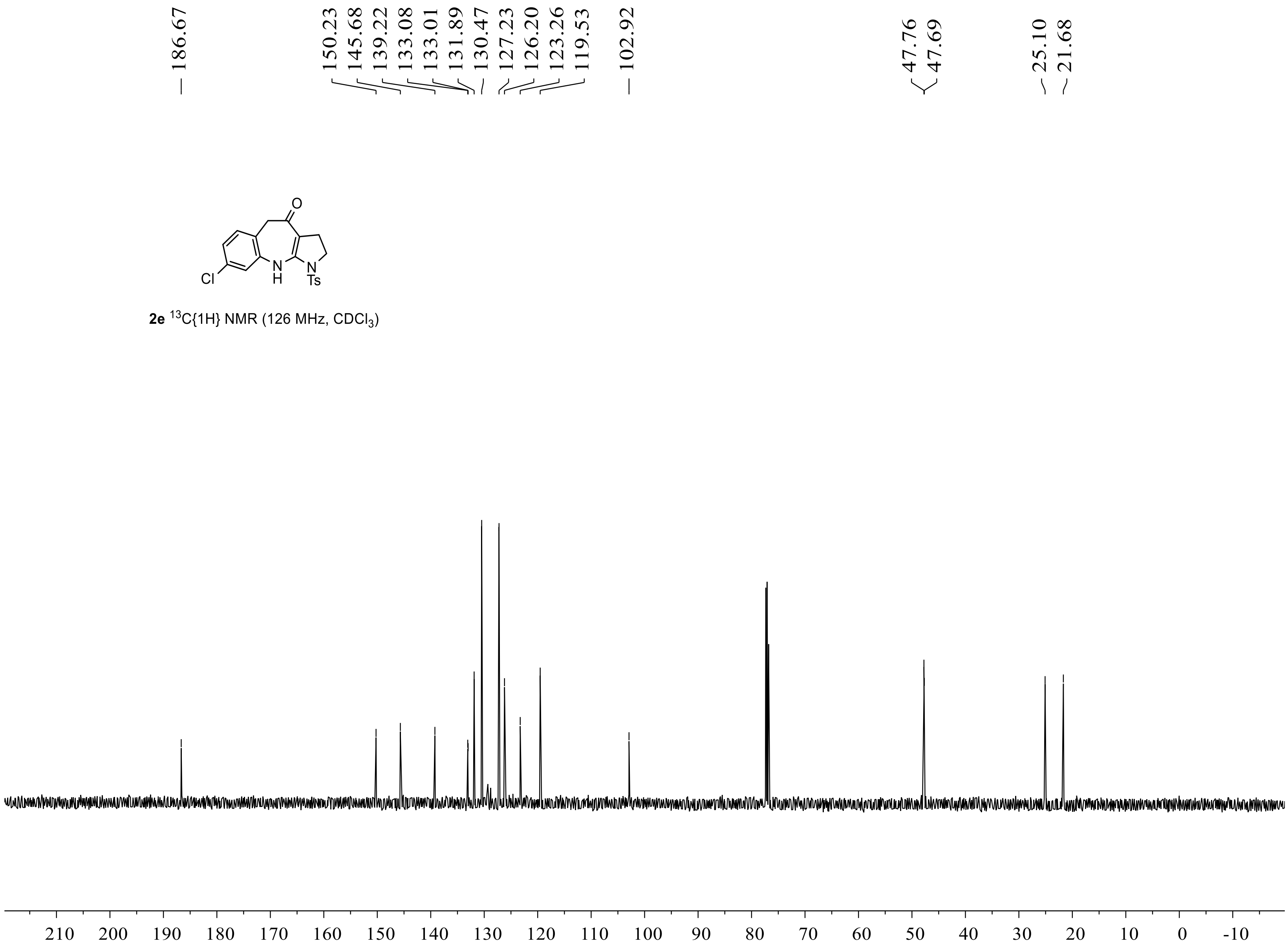

Supplementary Figure 72. ^{13}C NMR ($CDCl_3$, 126 MHz, 298 K) spectrum for **2e**

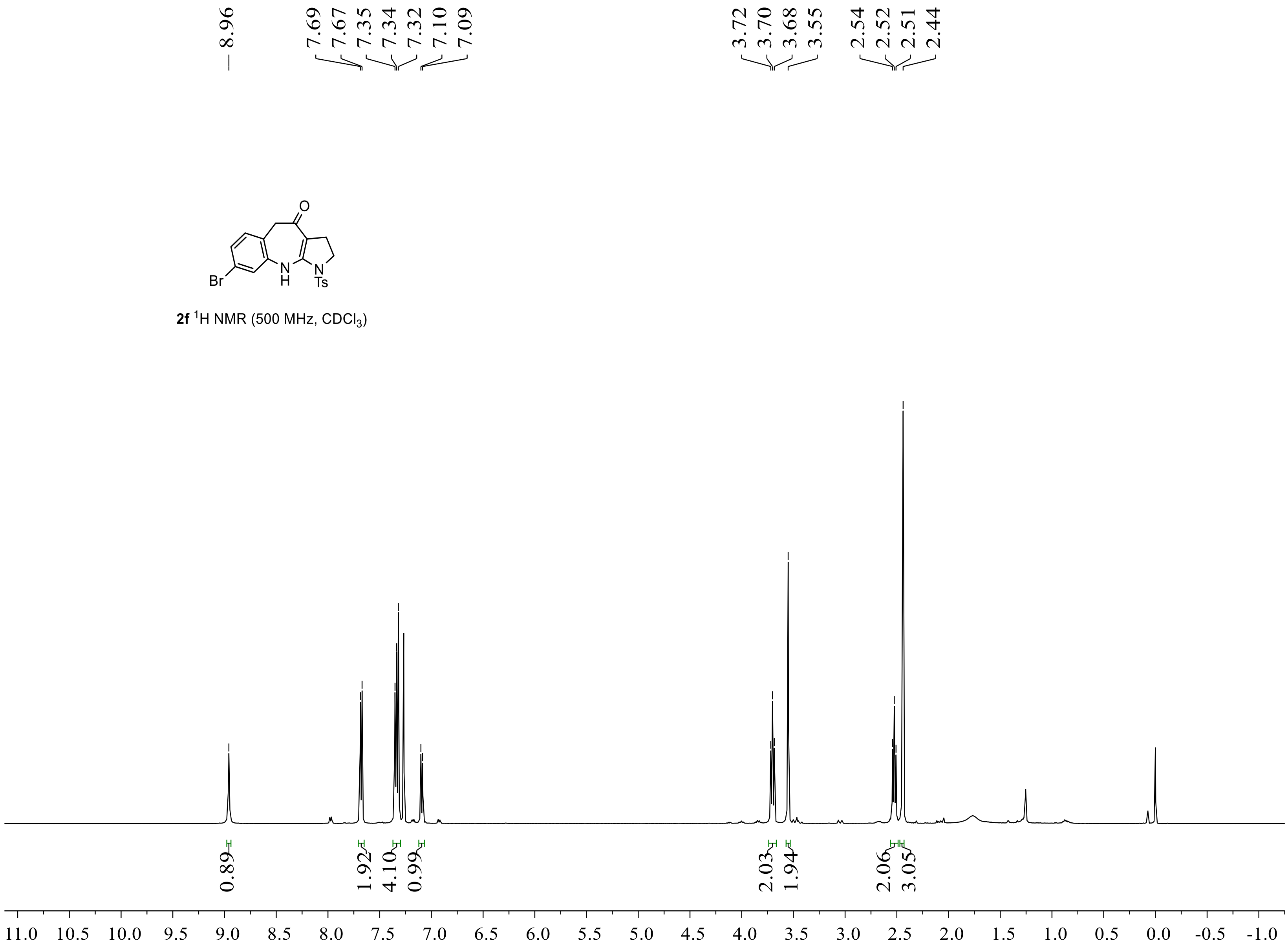

Supplementary Figure 73. ^{1}H NMR ($CDCl_3$, 500 MHz, 298 K) spectrum for **2f**

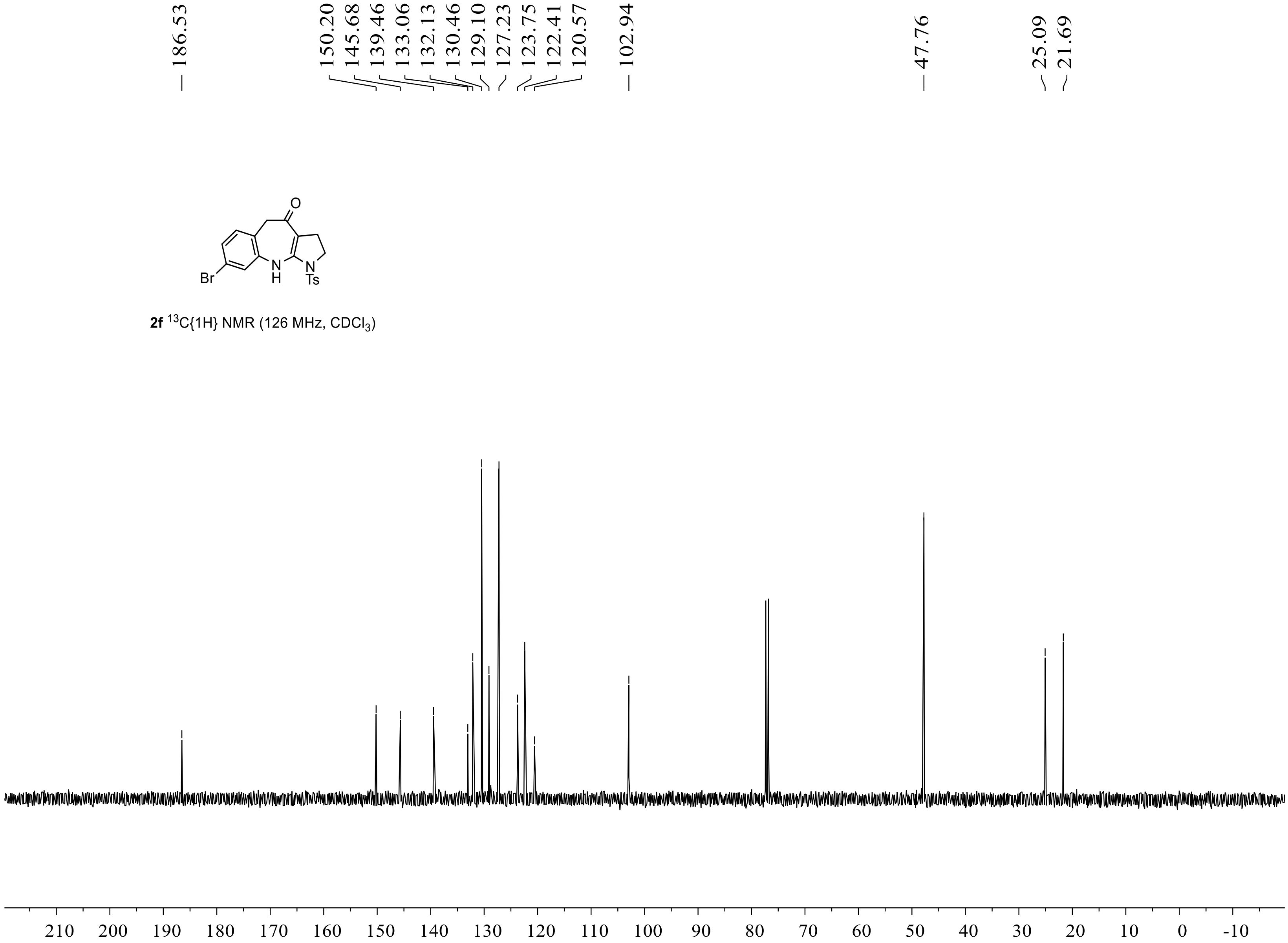

Supplementary Figure 74. ^{13}C NMR ($CDCl_3$, 126 MHz, 298 K) spectrum for **2f**

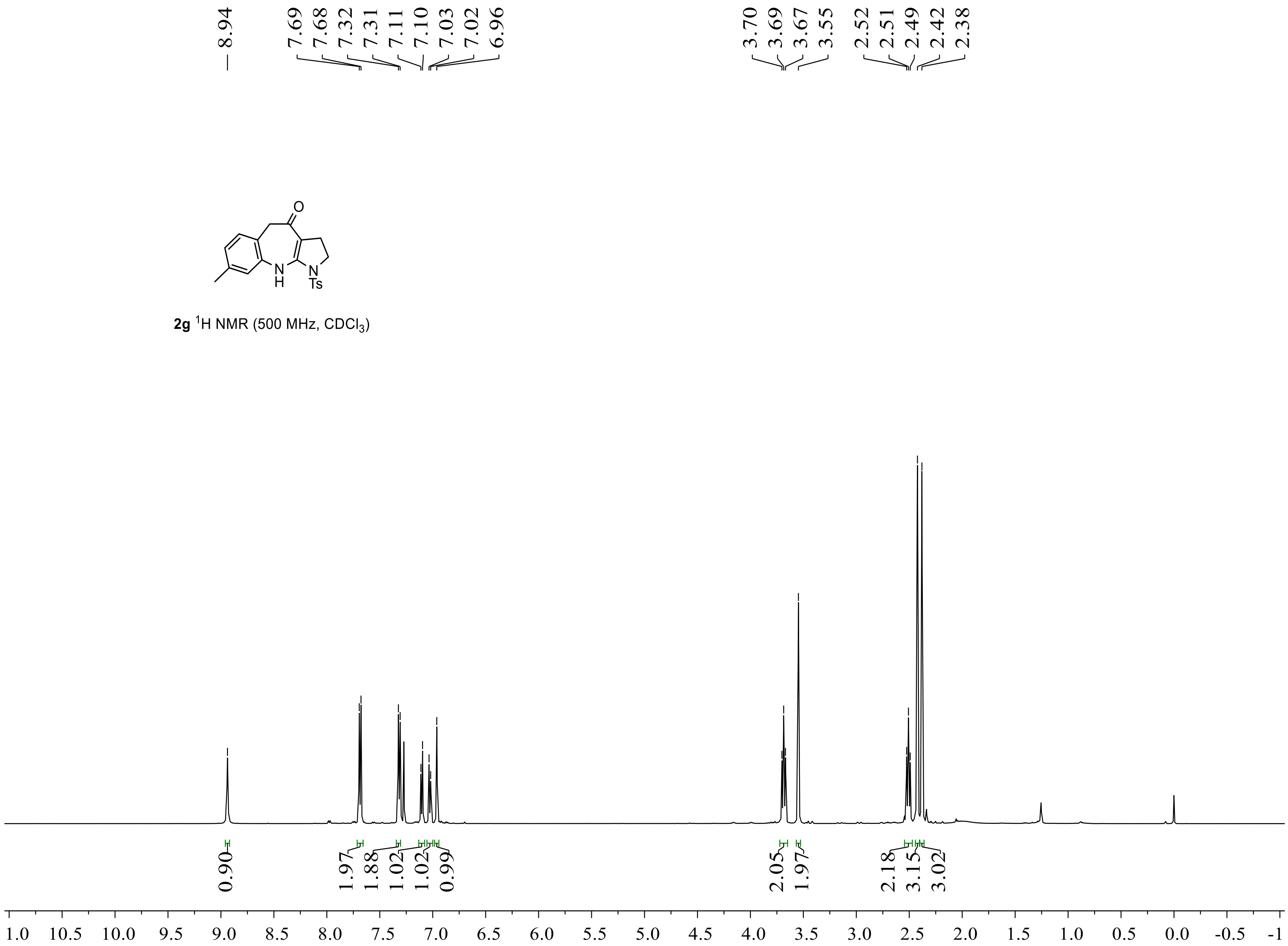

Supplementary Figure 75. ^{1}H NMR ($CDCl_3$, 500 MHz, 298 K) spectrum for **2g**

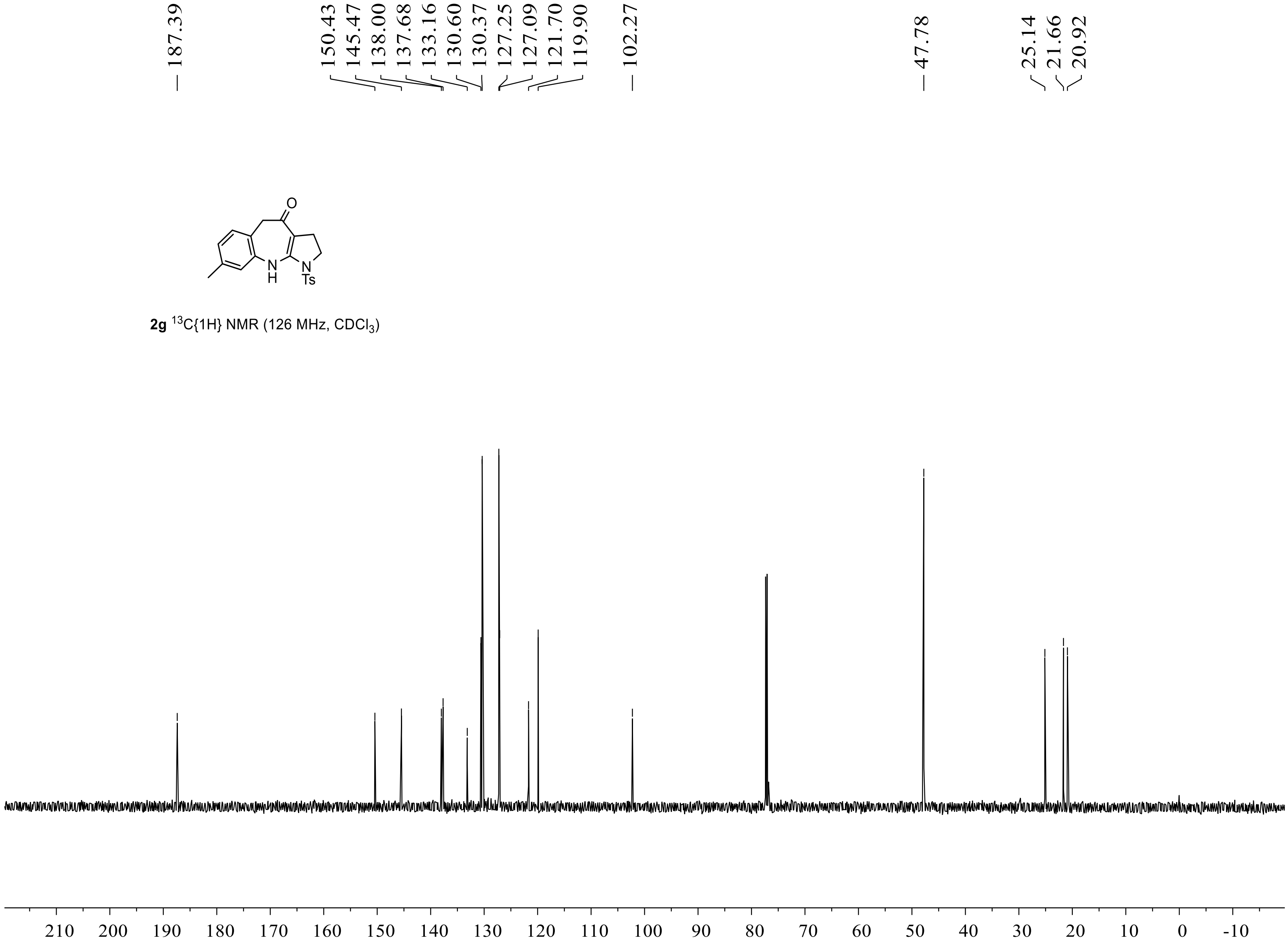

Supplementary Figure 76. ^{13}C NMR ($CDCl_3$, 126 MHz, 298 K) spectrum for **2g**

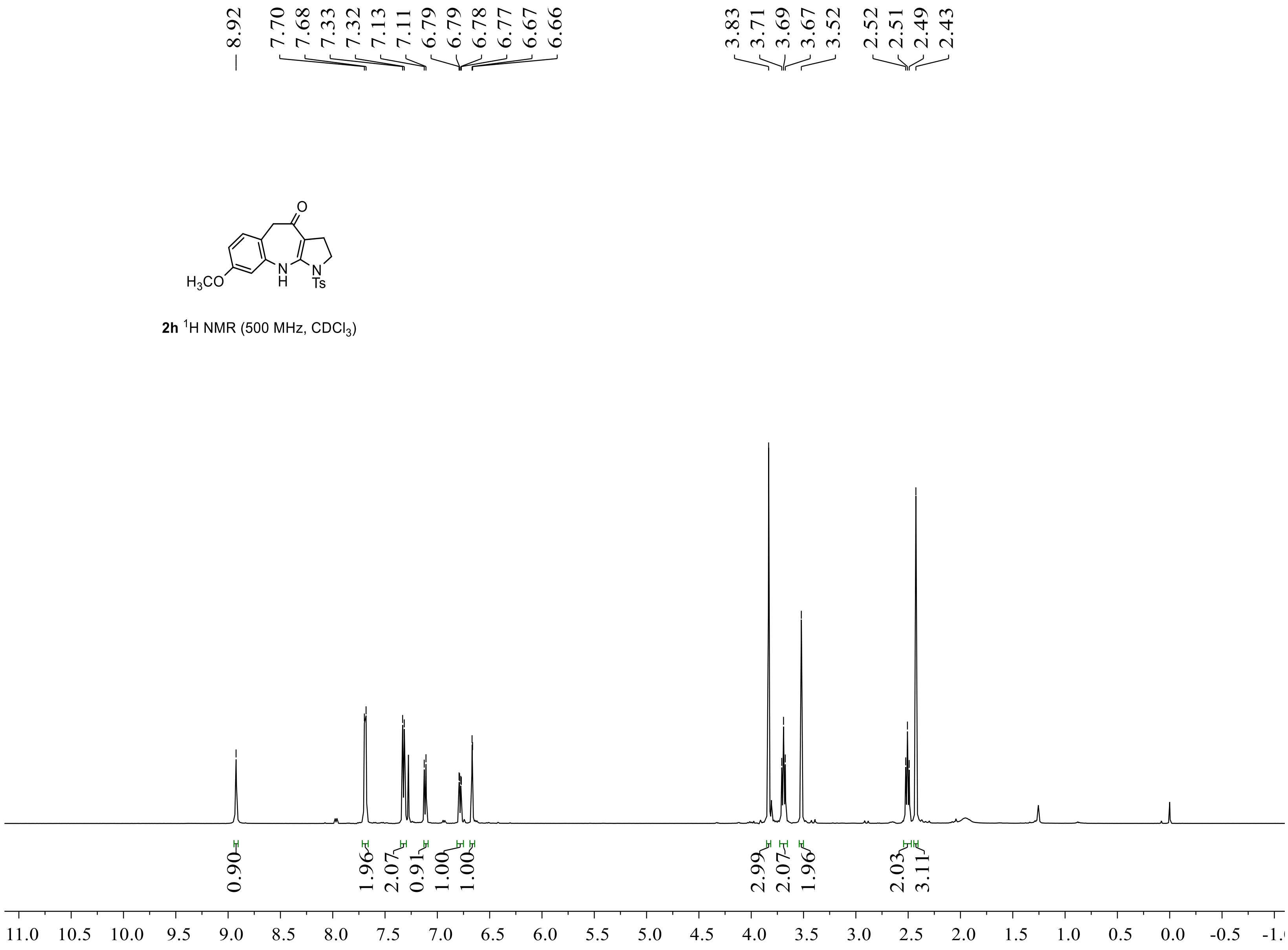

Supplementary Figure 77. ^{1}H NMR ($CDCl_3$, 500 MHz, 298 K) spectrum for **2h**

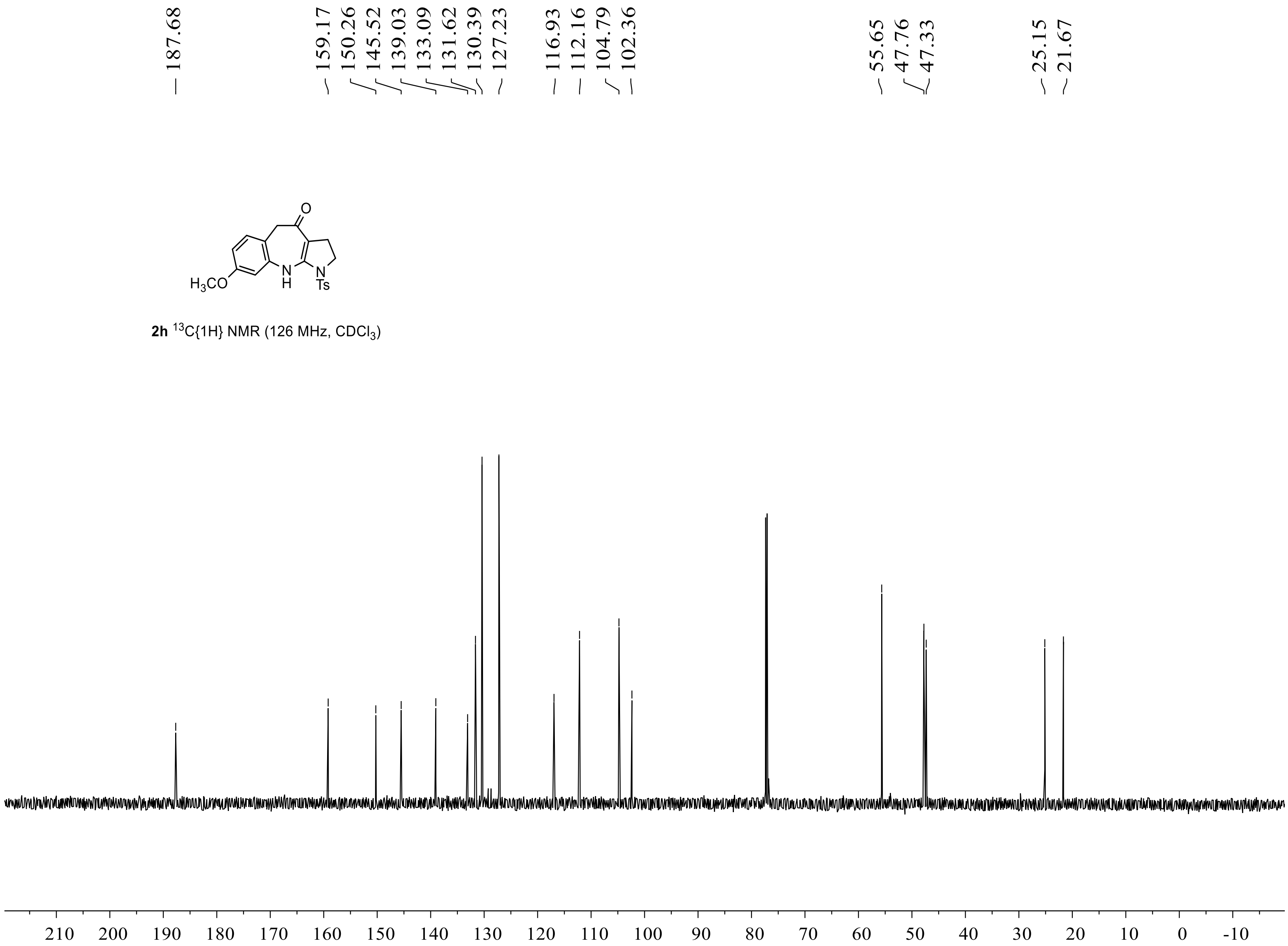

Supplementary Figure 78. ^{13}C NMR ($CDCl_3$, 126 MHz, 298 K) spectrum for **2h**

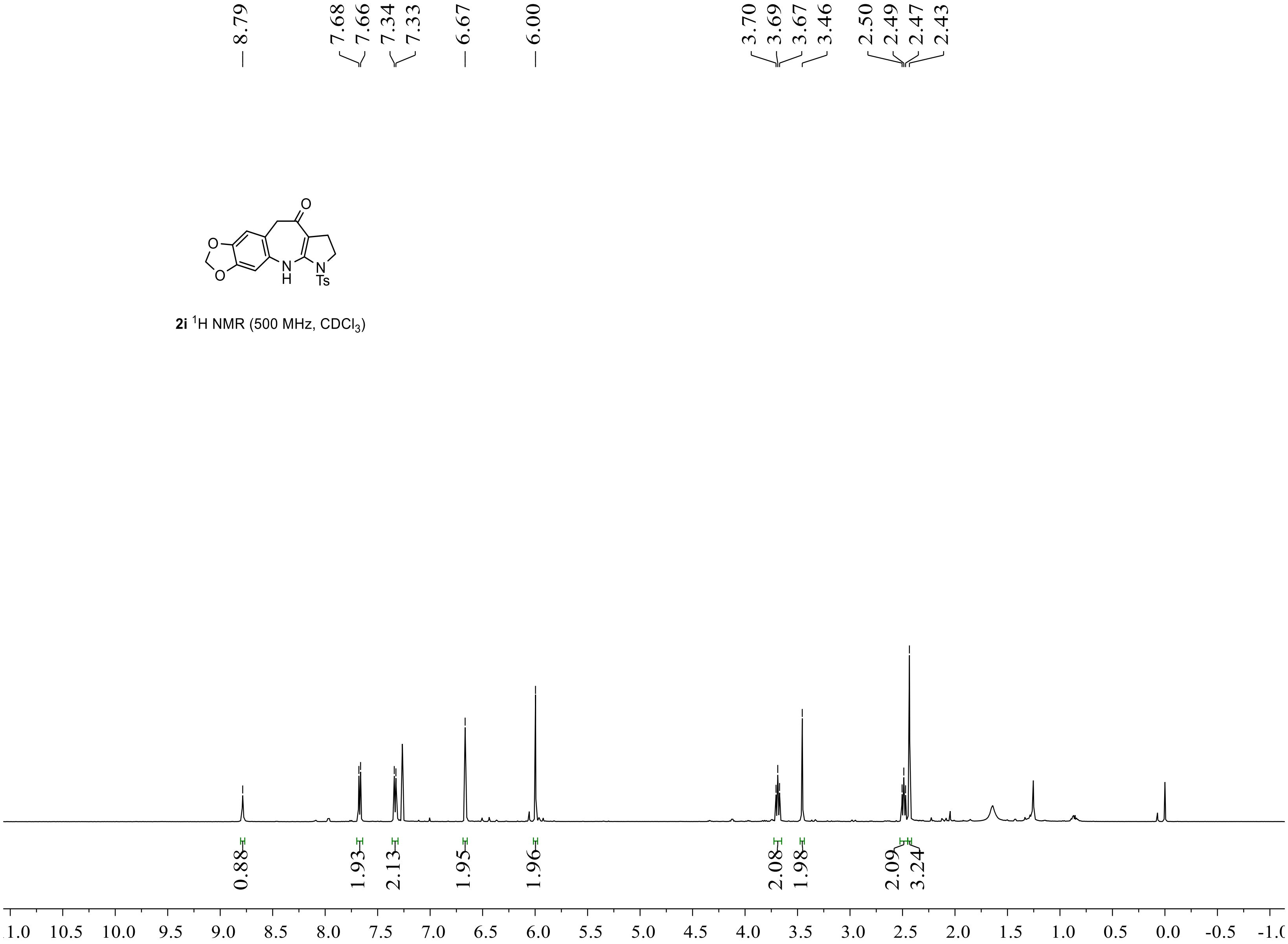

Supplementary Figure 79. ^{1}H NMR ($CDCl_3$, 500 MHz, 298 K) spectrum for **2i**

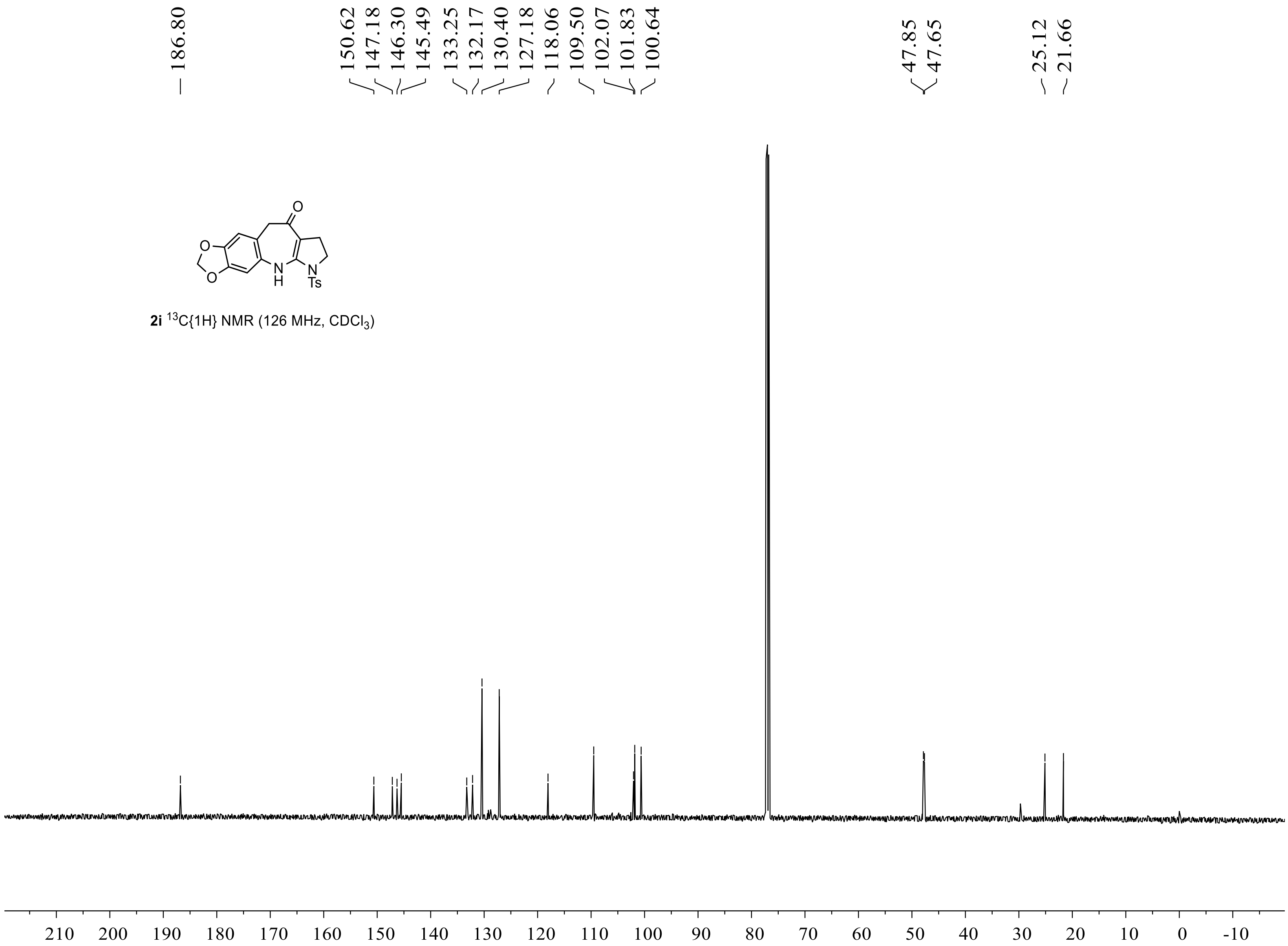

Supplementary Figure 80. ^{13}C NMR ($CDCl_3$, 126 MHz, 298 K) spectrum for **2i**

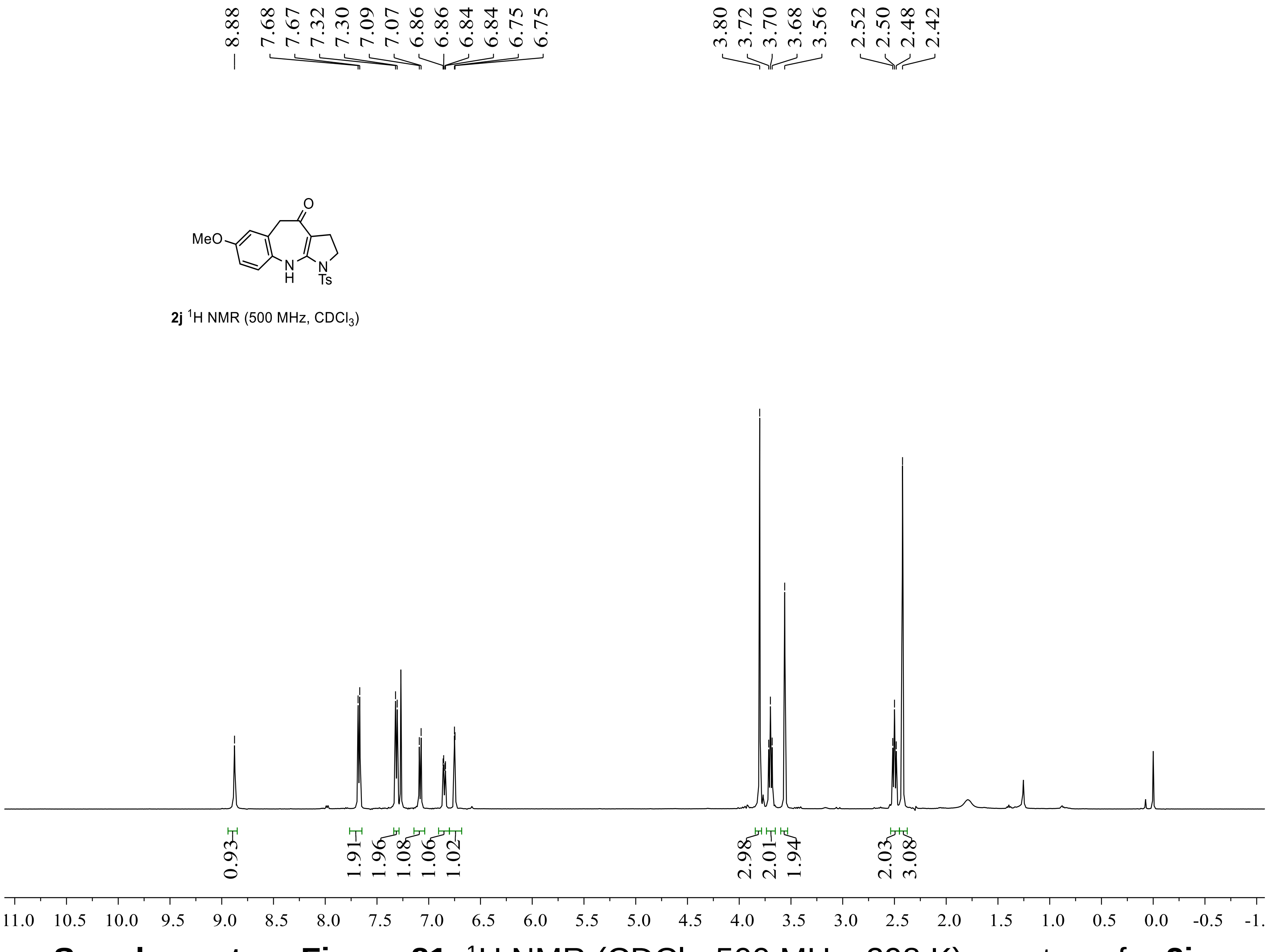

Supplementary Figure 81. ^{1}H NMR ($CDCl_3$, 500 MHz, 298 K) spectrum for **2i**

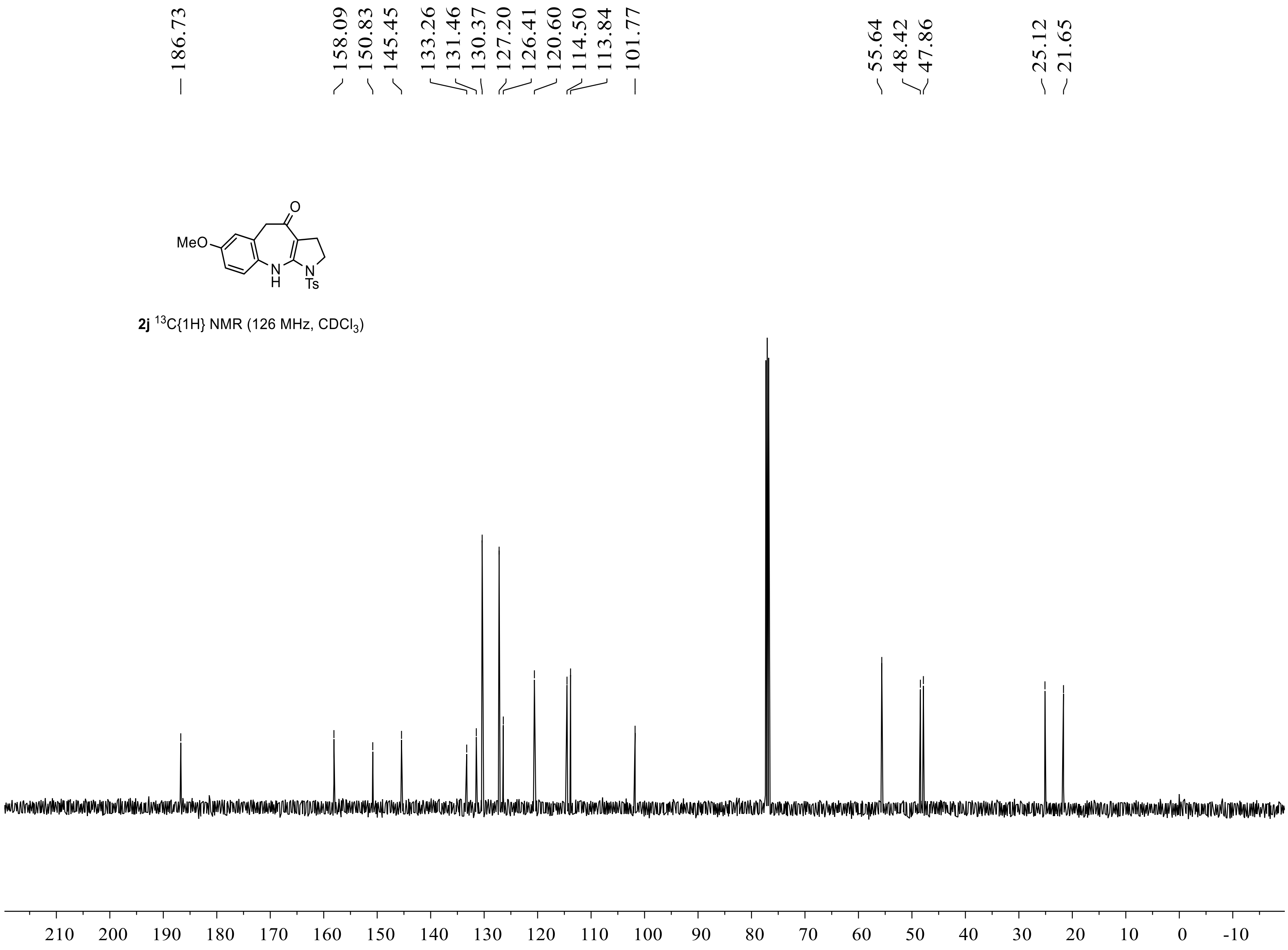

Supplementary Figure 82. ^{13}C NMR ($CDCl_3$, 126 MHz, 298 K) spectrum for **2j**

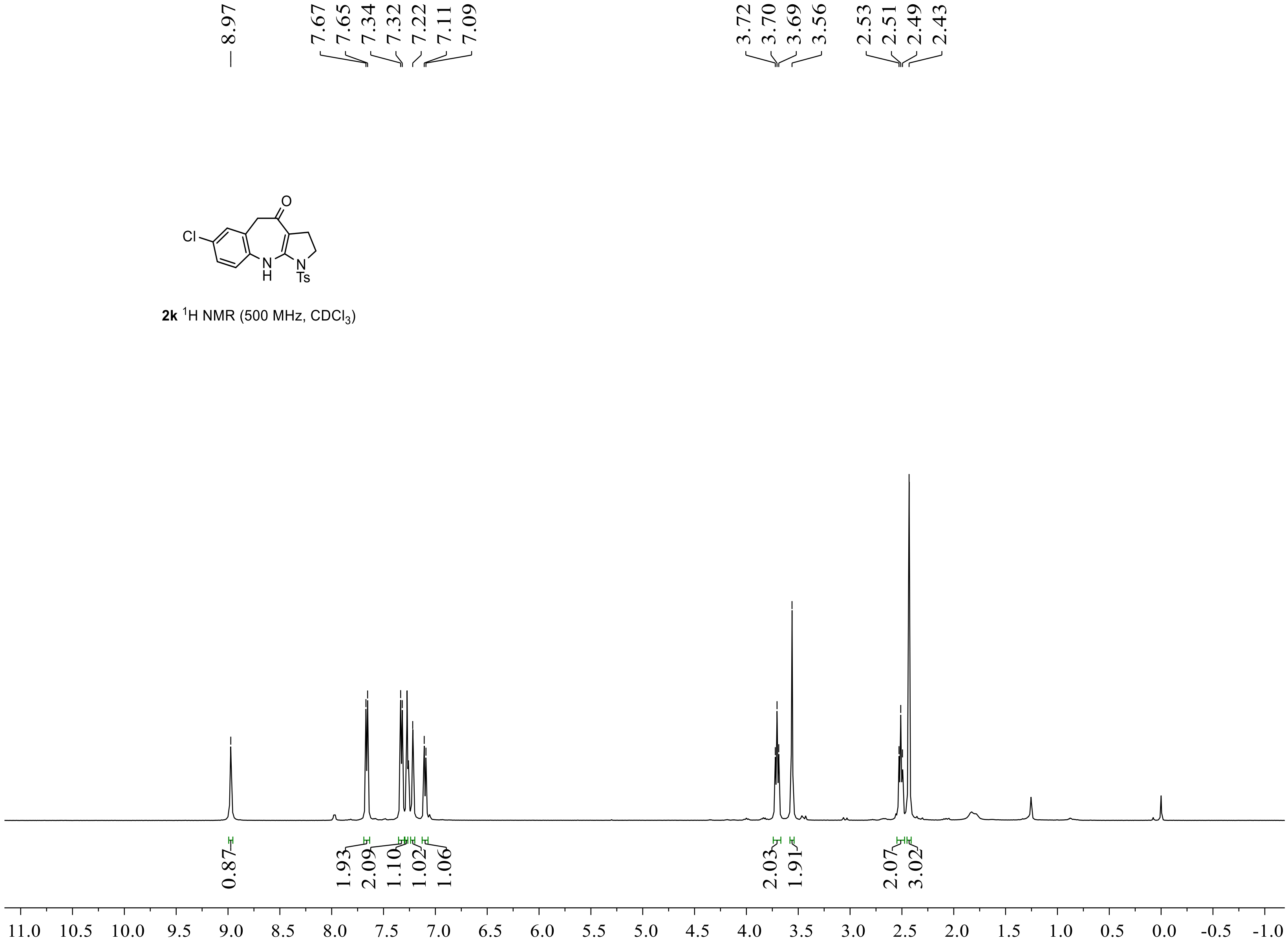

Supplementary Figure 83. ^{1}H NMR ($CDCl_3$, 500 MHz, 298 K) spectrum for **2k**

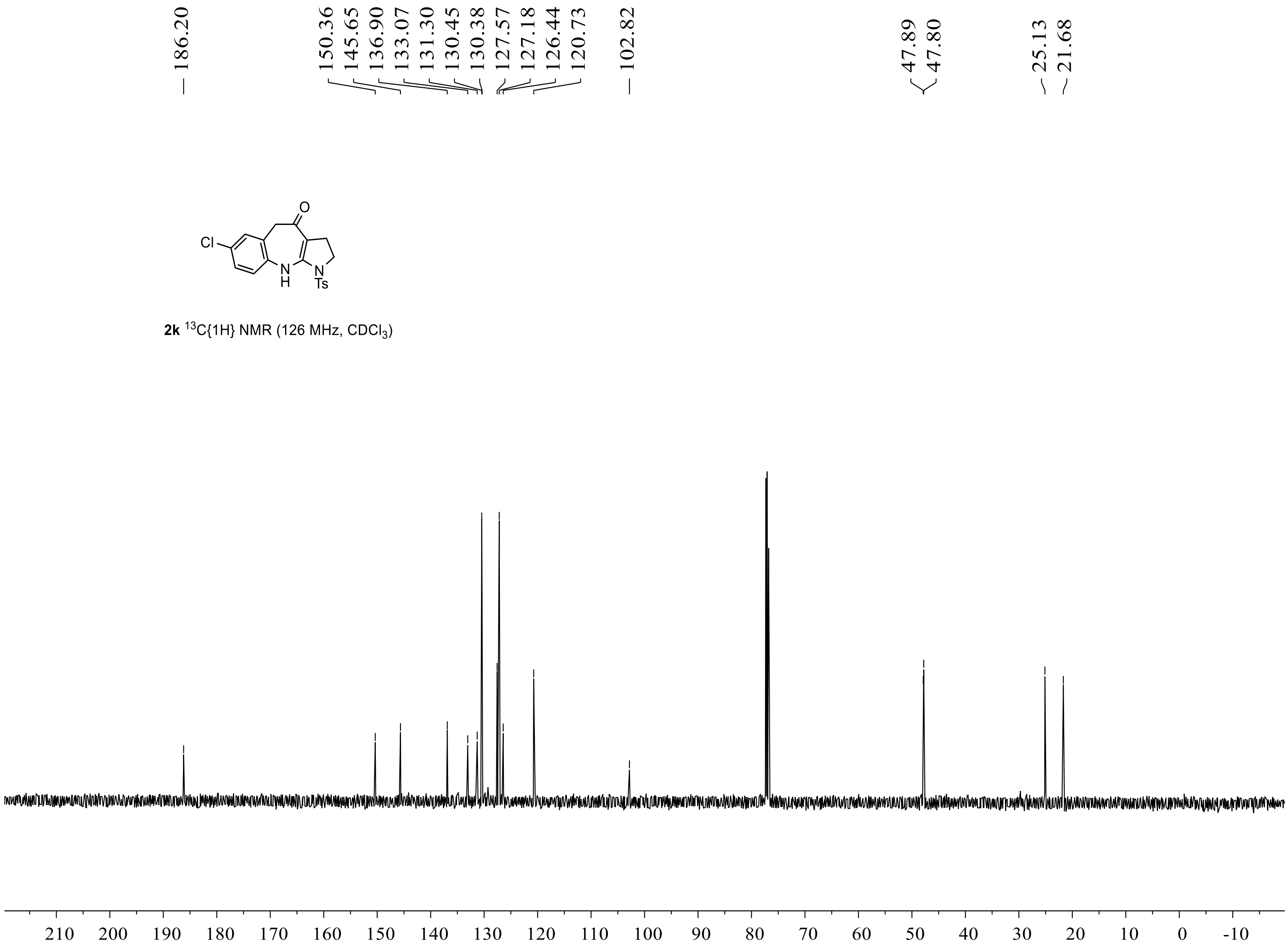

Supplementary Figure 84. ^{13}C NMR ($CDCl_3$, 126 MHz, 298 K) spectrum for **2k**

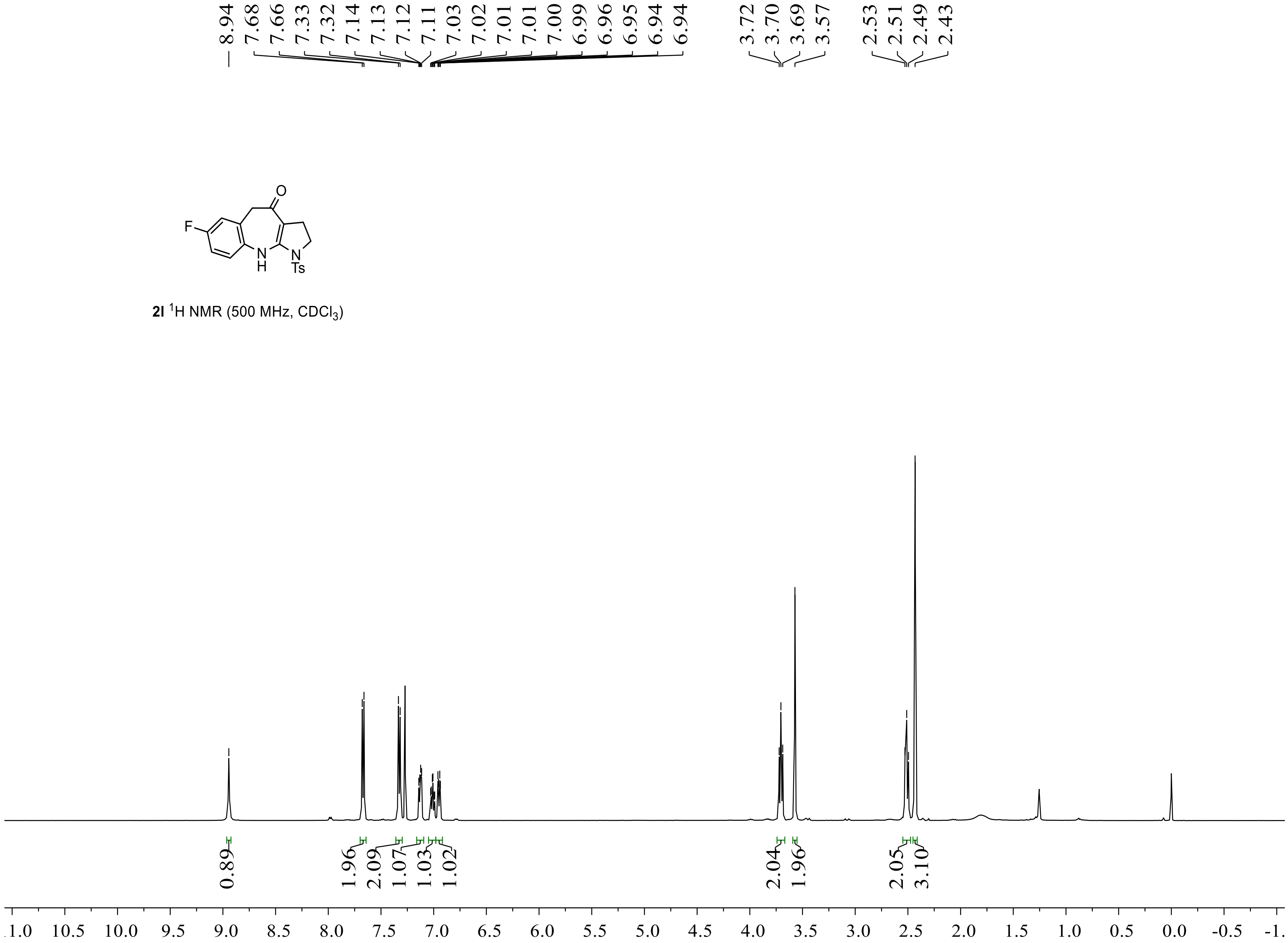

Supplementary Figure 85. ^{1}H NMR ($CDCl_3$, 500 MHz, 298 K) spectrum for **2l**

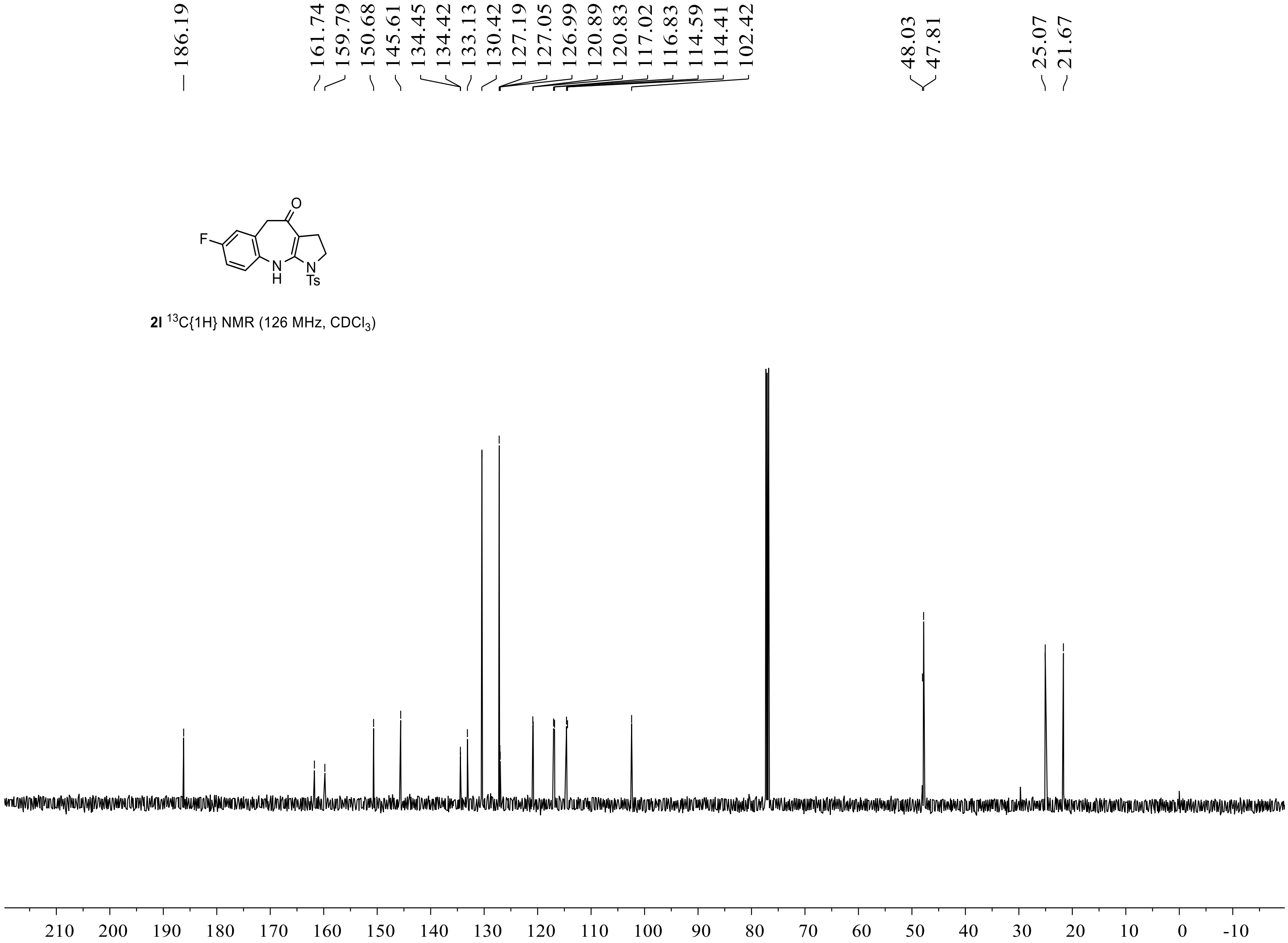

Supplementary Figure 86. ^{13}C NMR ($CDCl_3$, 126 MHz, 298 K) spectrum for **2l**

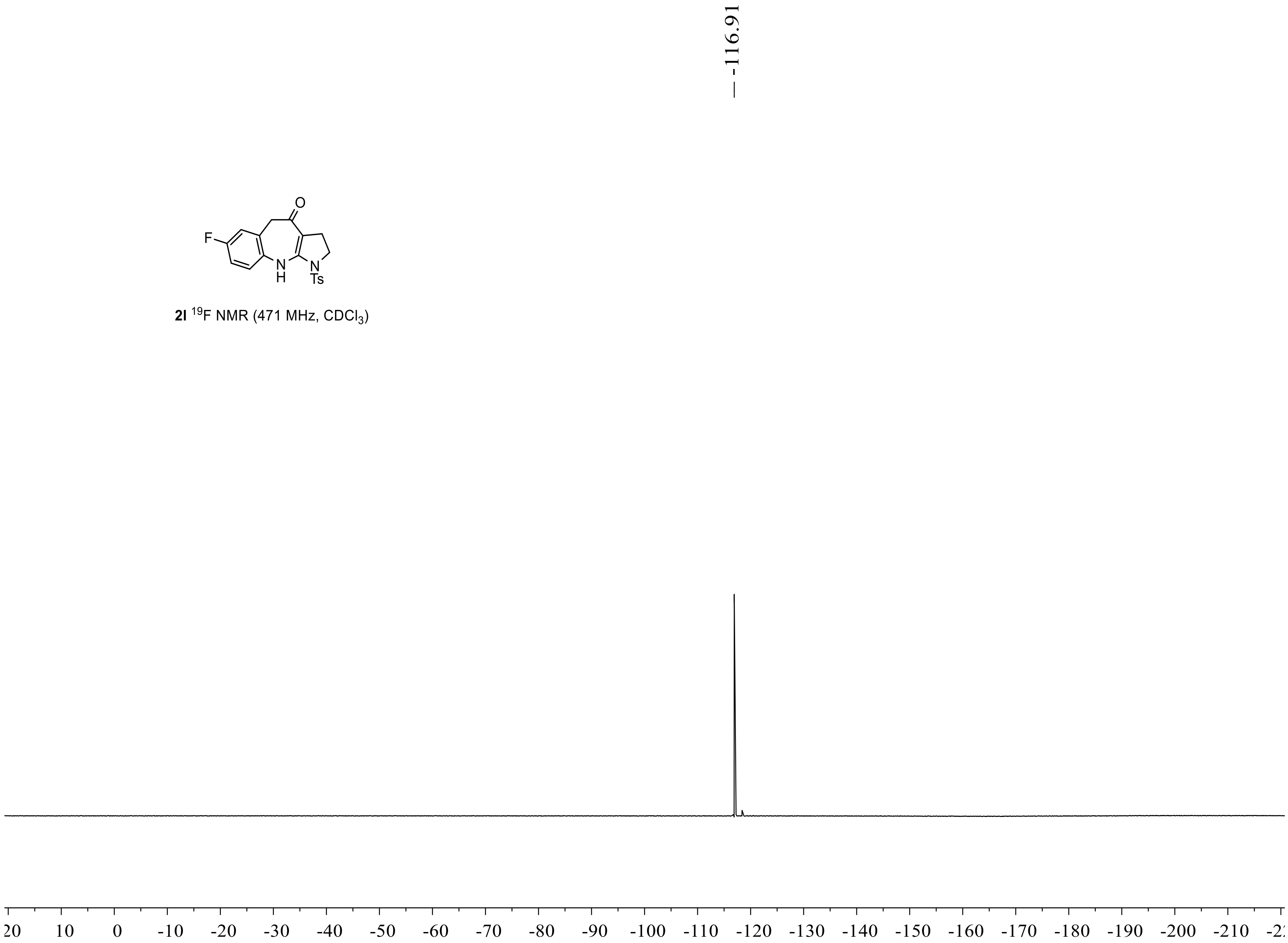

Supplementary Figure 87. ^{19}F NMR ($CDCl_3$, 471 MHz, 298 K) spectrum for **2l**

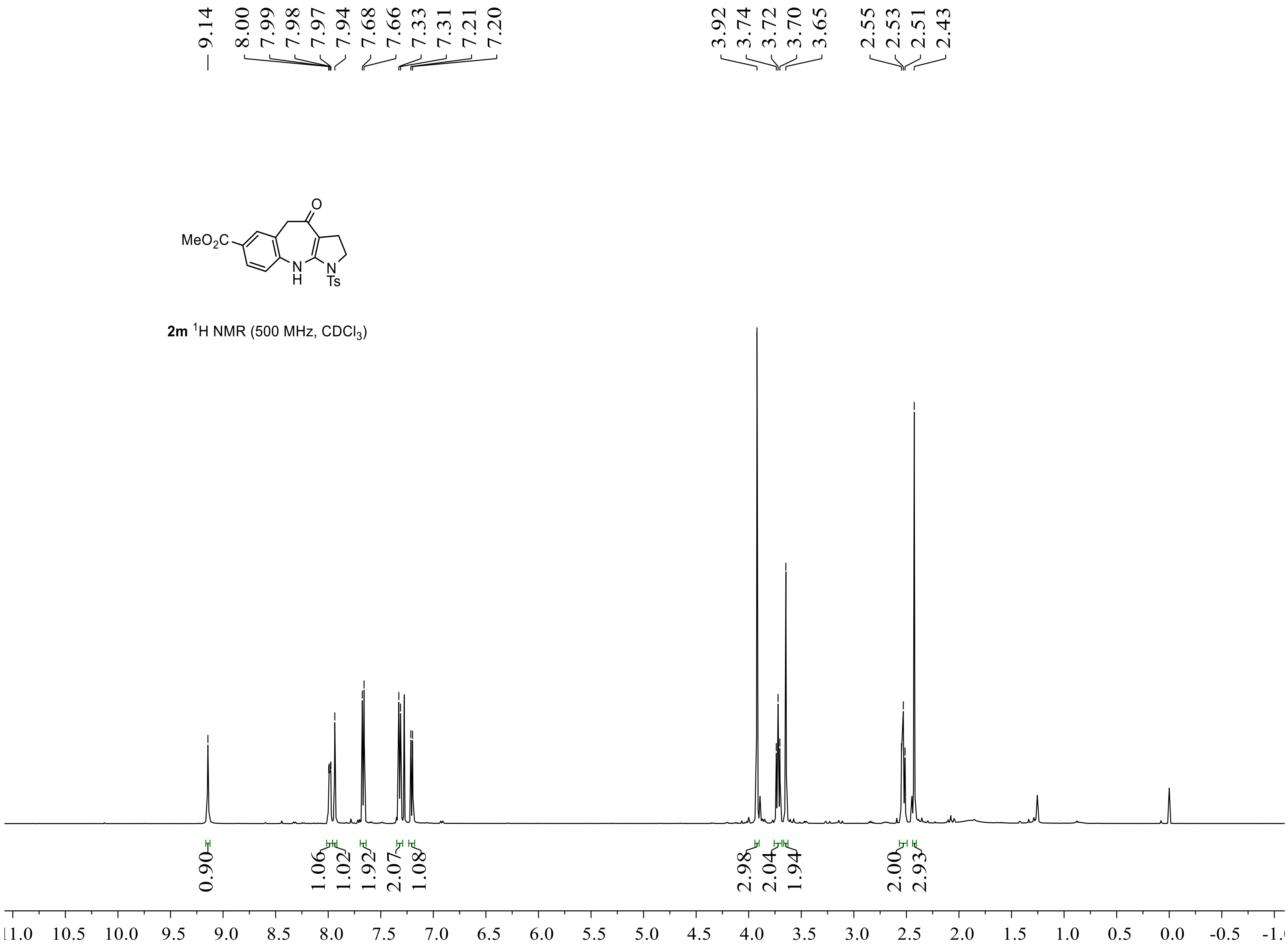

Supplementary Figure 88. ^{1}H NMR ($CDCl_3$, 500 MHz, 298 K) spectrum for **2m**

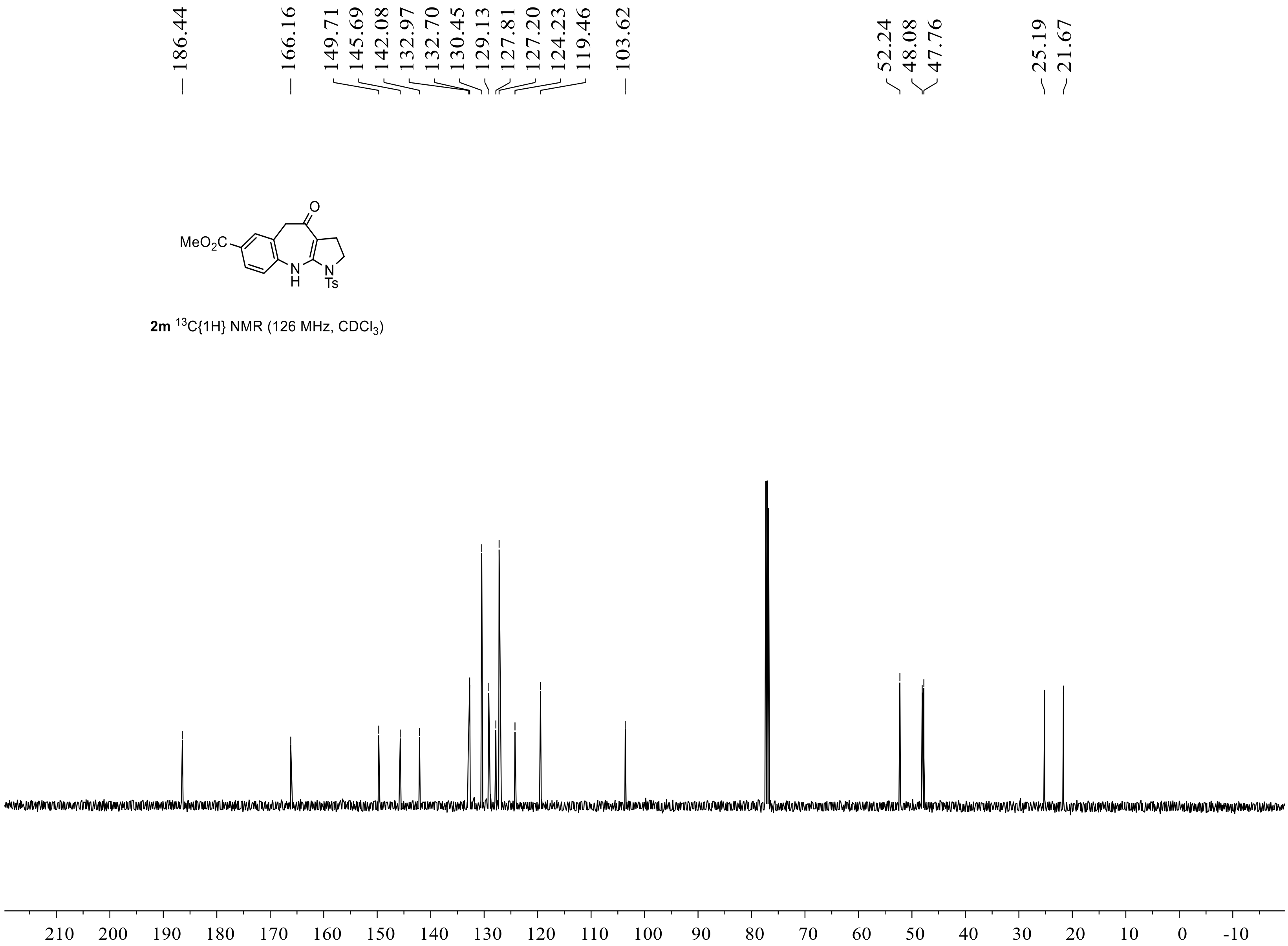

Supplementary Figure 89. ^{13}C NMR ($CDCl_3$, 126 MHz, 298 K) spectrum for **2m**

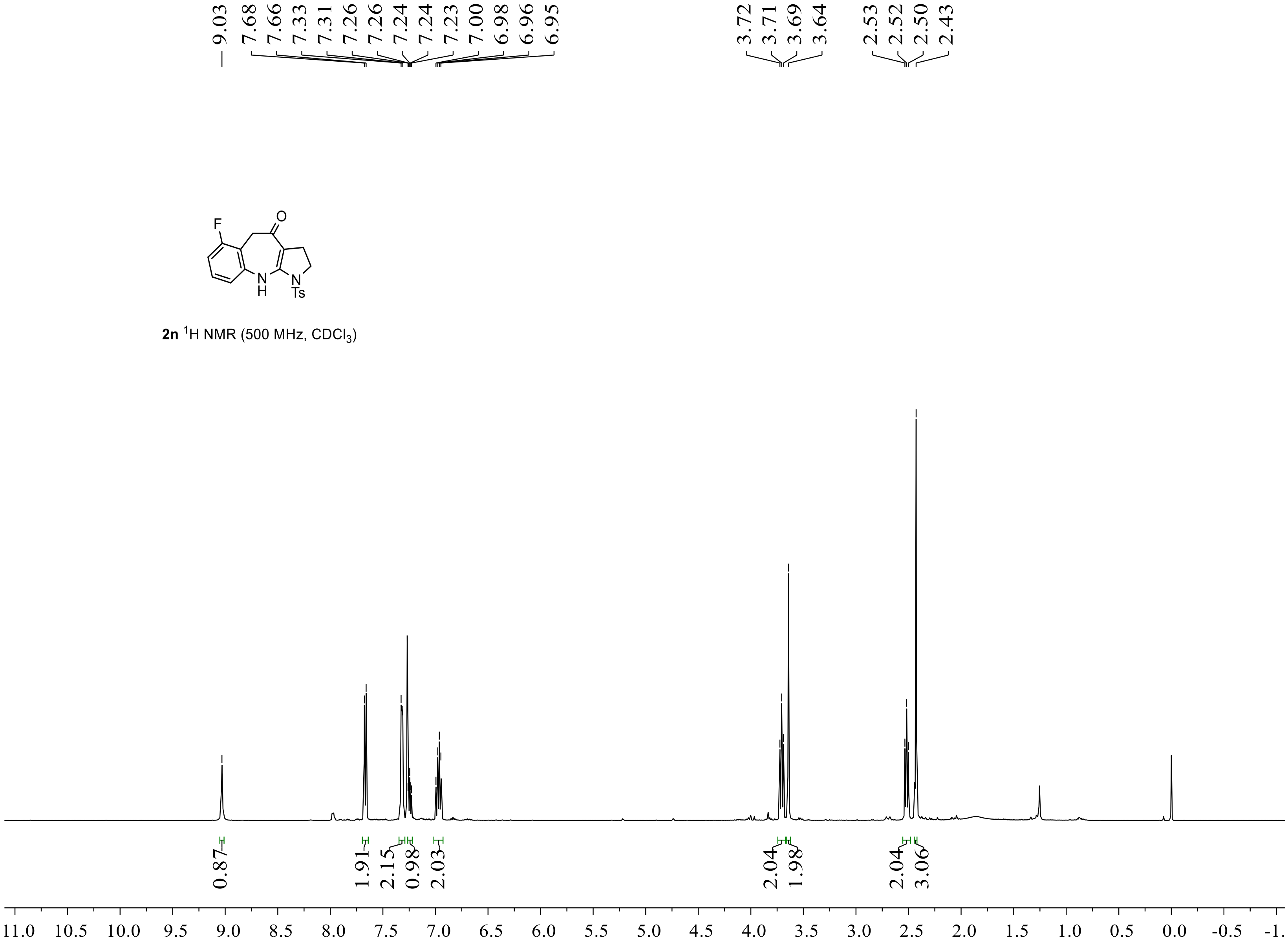

Supplementary Figure 90. ^{1}H NMR ($CDCl_3$, 500 MHz, 298 K) spectrum for **2n**

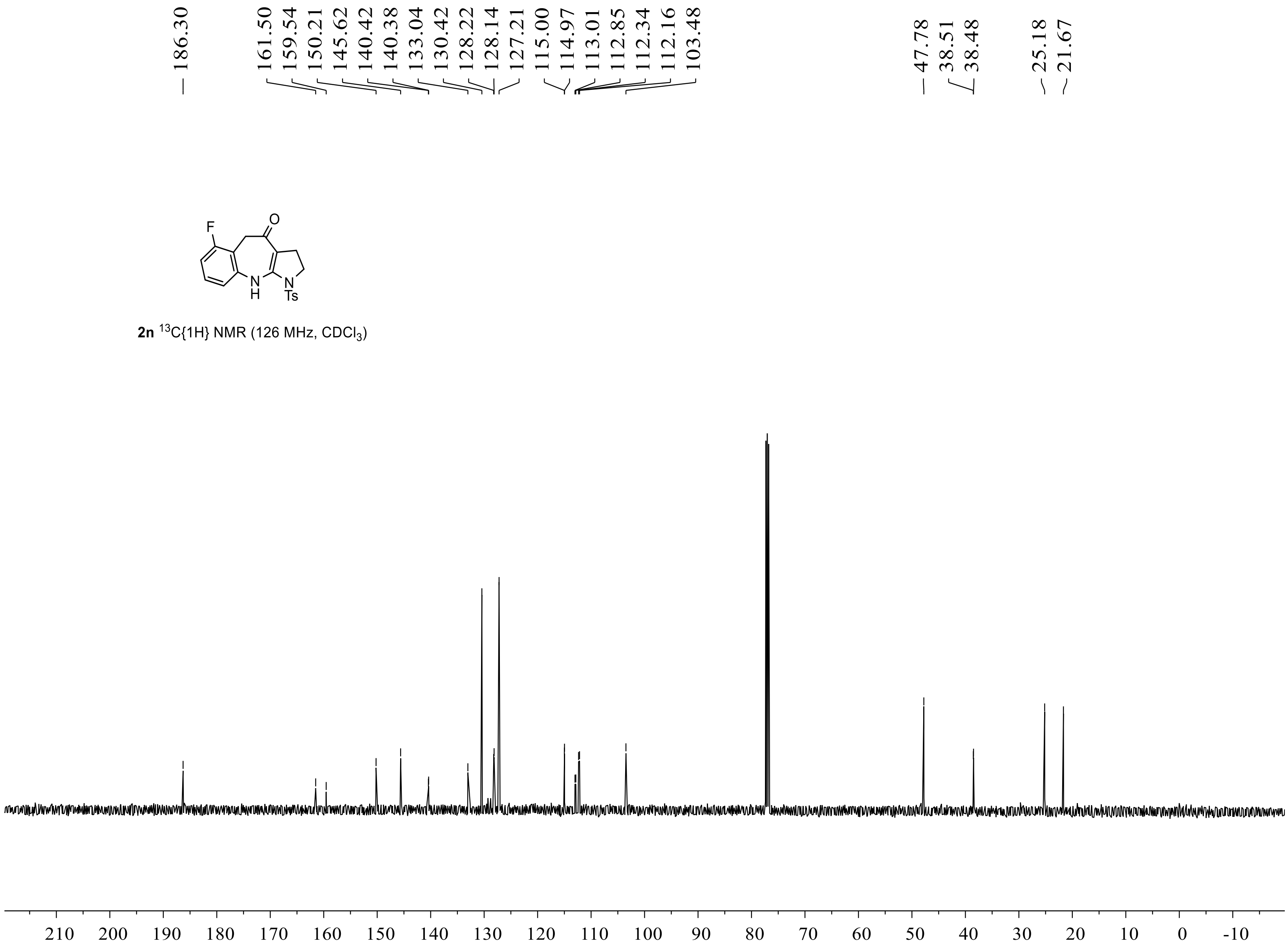

Supplementary Figure 91. ^{13}C NMR ($CDCl_3$, 126 MHz, 298 K) spectrum for **2n**

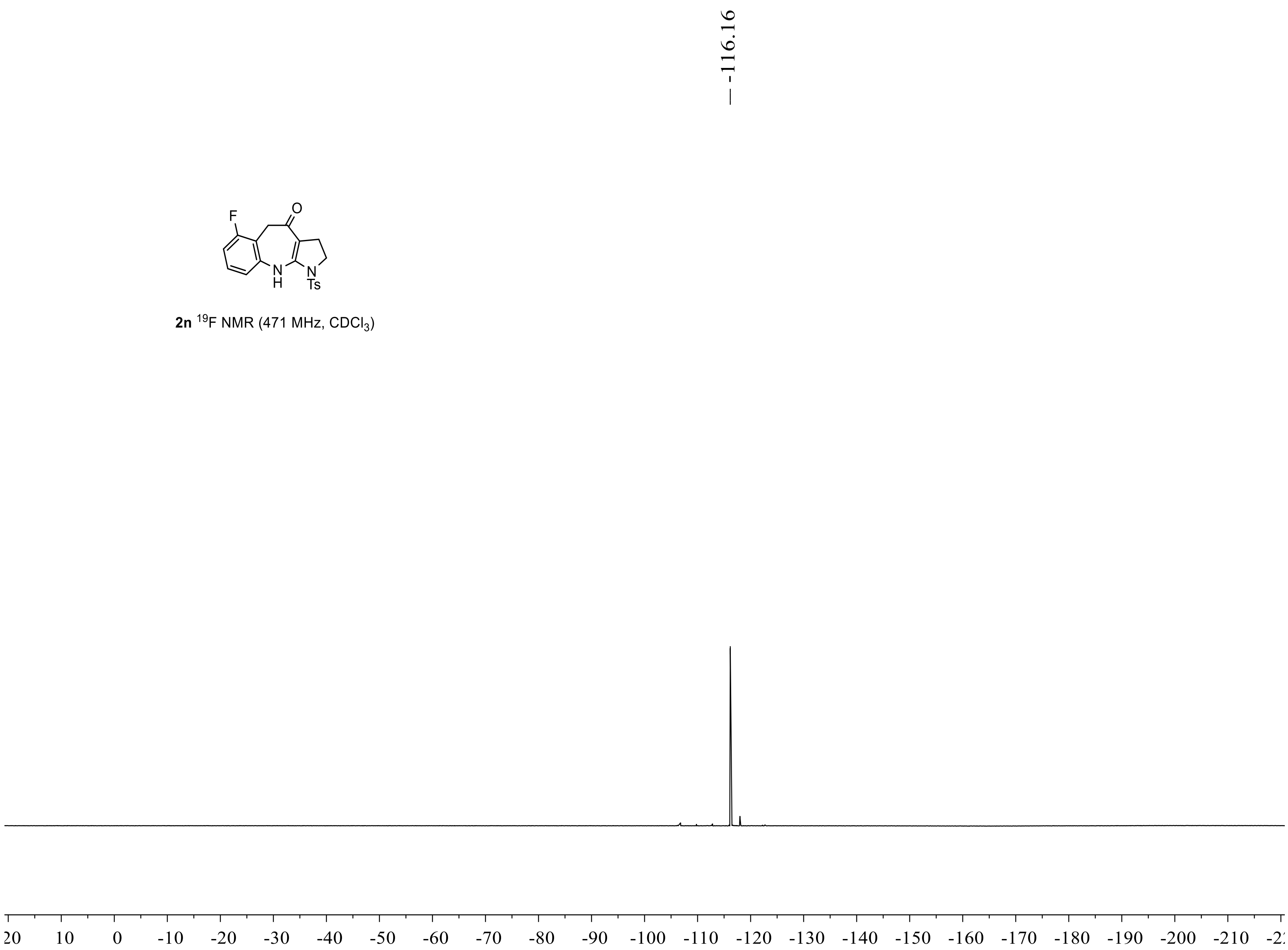

Supplementary Figure 92. ^{19}F NMR ($CDCl_3$, 471 MHz, 298 K) spectrum for **2n**

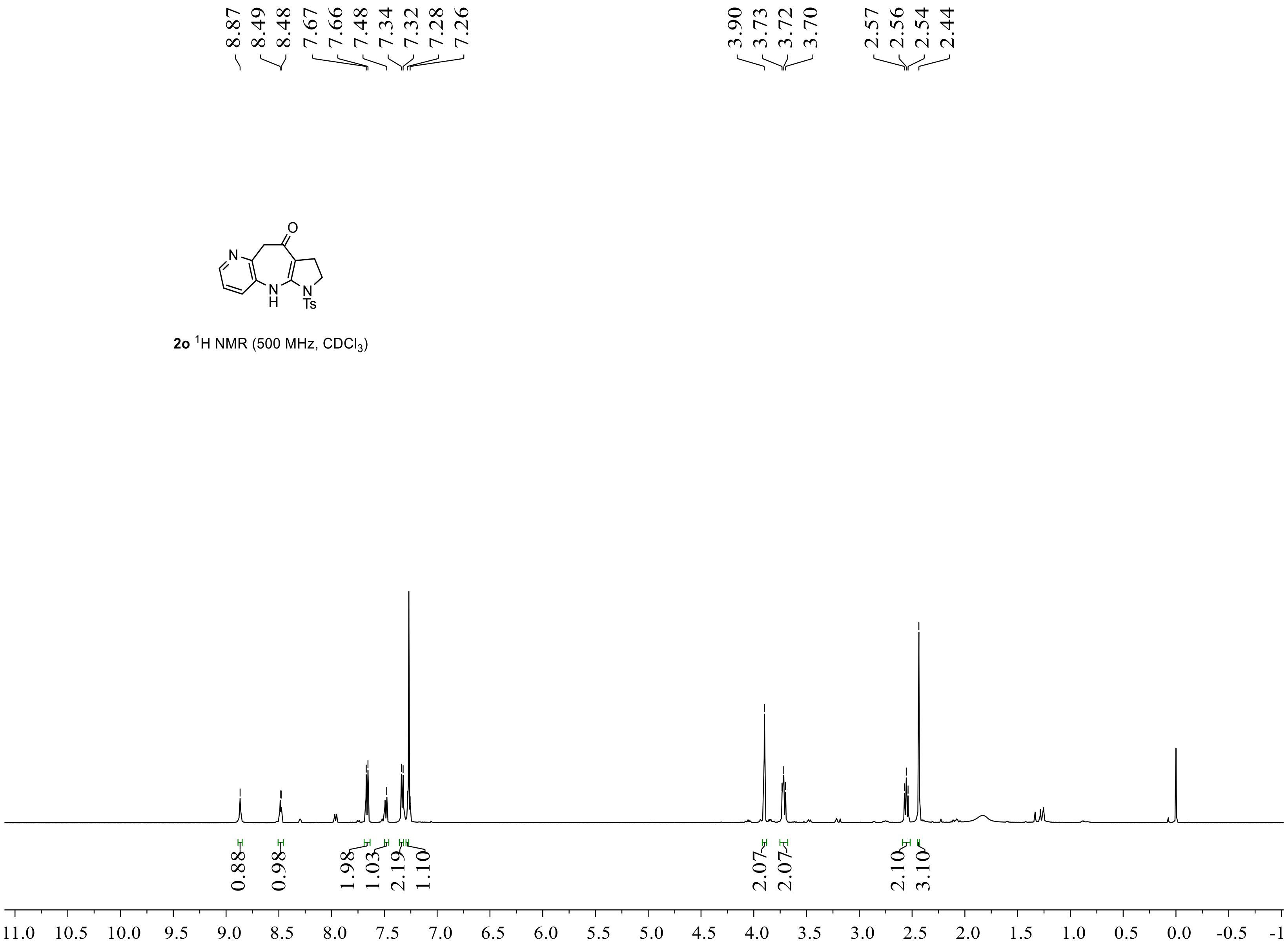

Supplementary Figure 93. ^{1}H NMR ($CDCl_3$, 500 MHz, 298 K) spectrum for **2o**

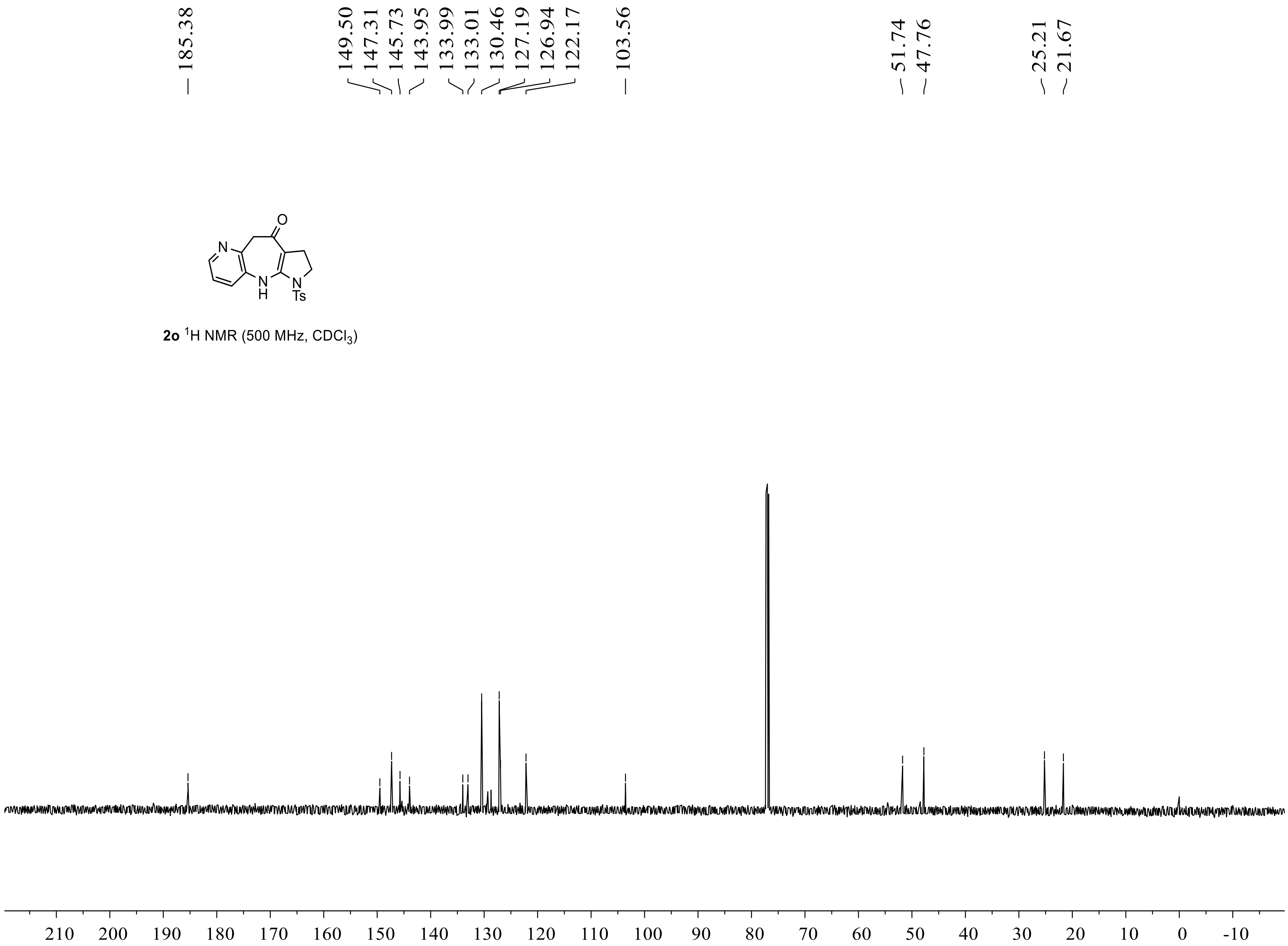

Supplementary Figure 94. ^{13}C NMR ($CDCl_3$, 126 MHz, 298 K) spectrum for **2o**

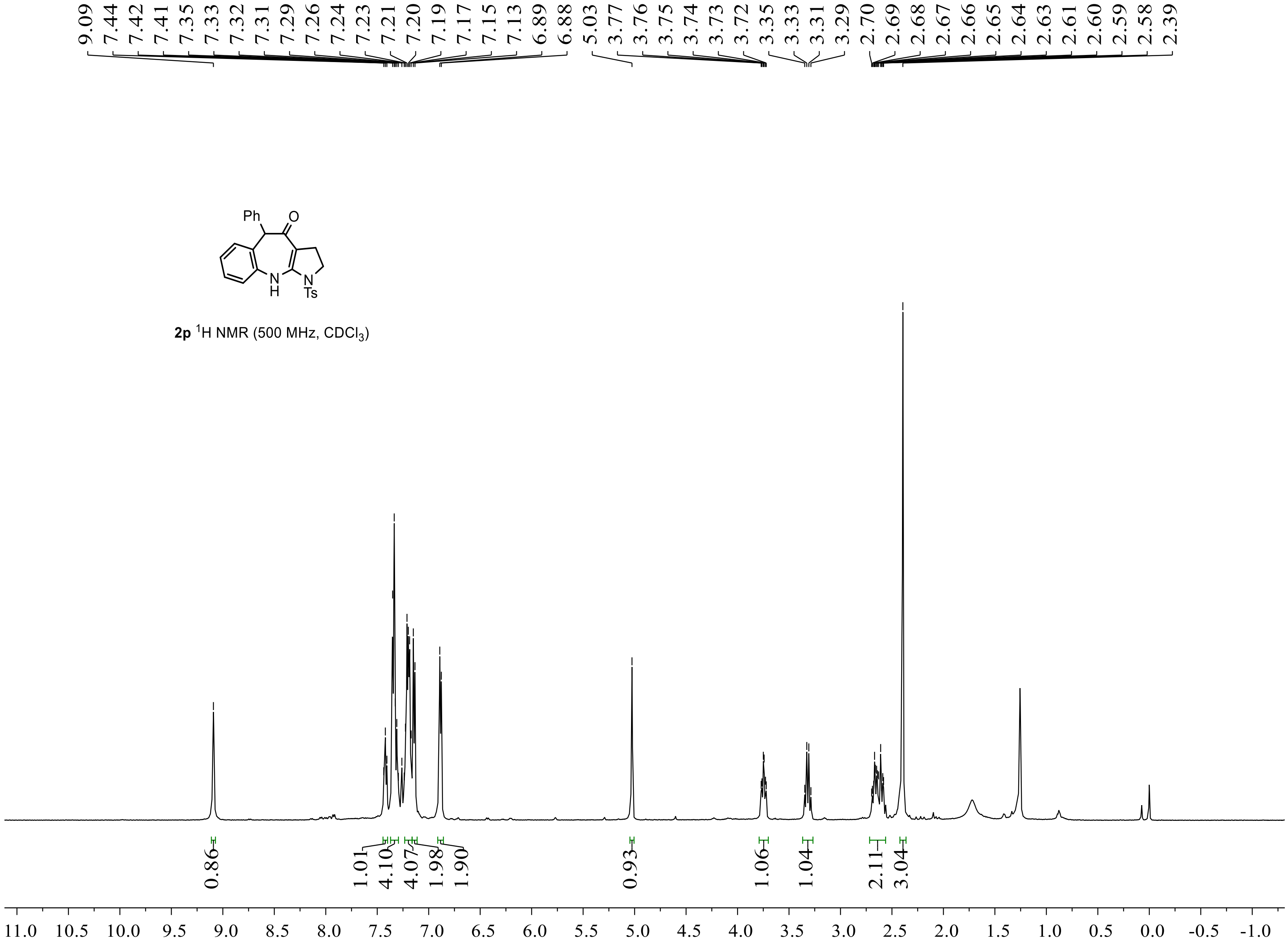

Supplementary Figure 95. ^{1}H NMR ($CDCl_3$, 500 MHz, 298 K) spectrum for **2p**

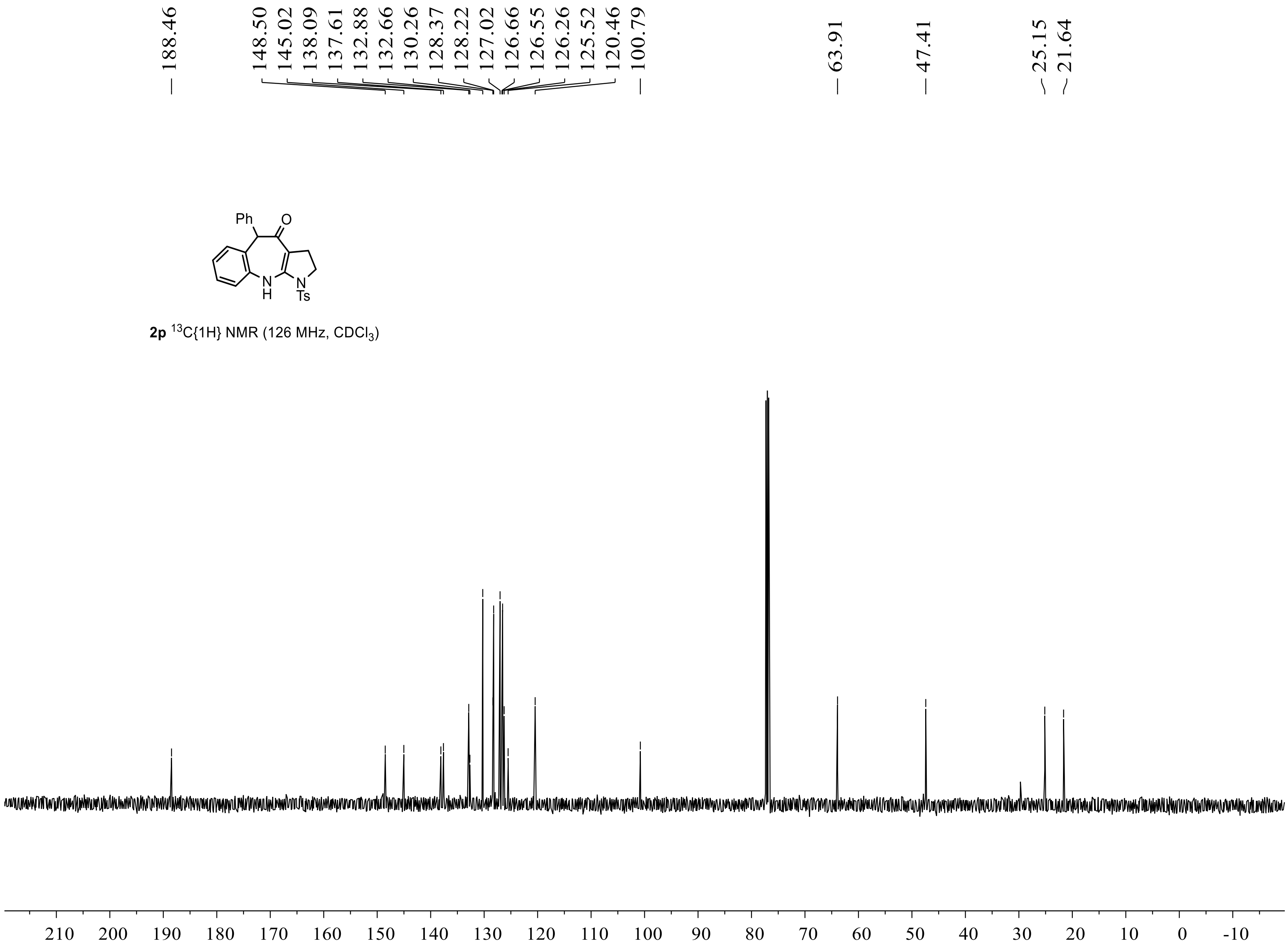

Supplementary Figure 96. ^{13}C NMR ($CDCl_3$, 126 MHz, 298 K) spectrum for **2p**

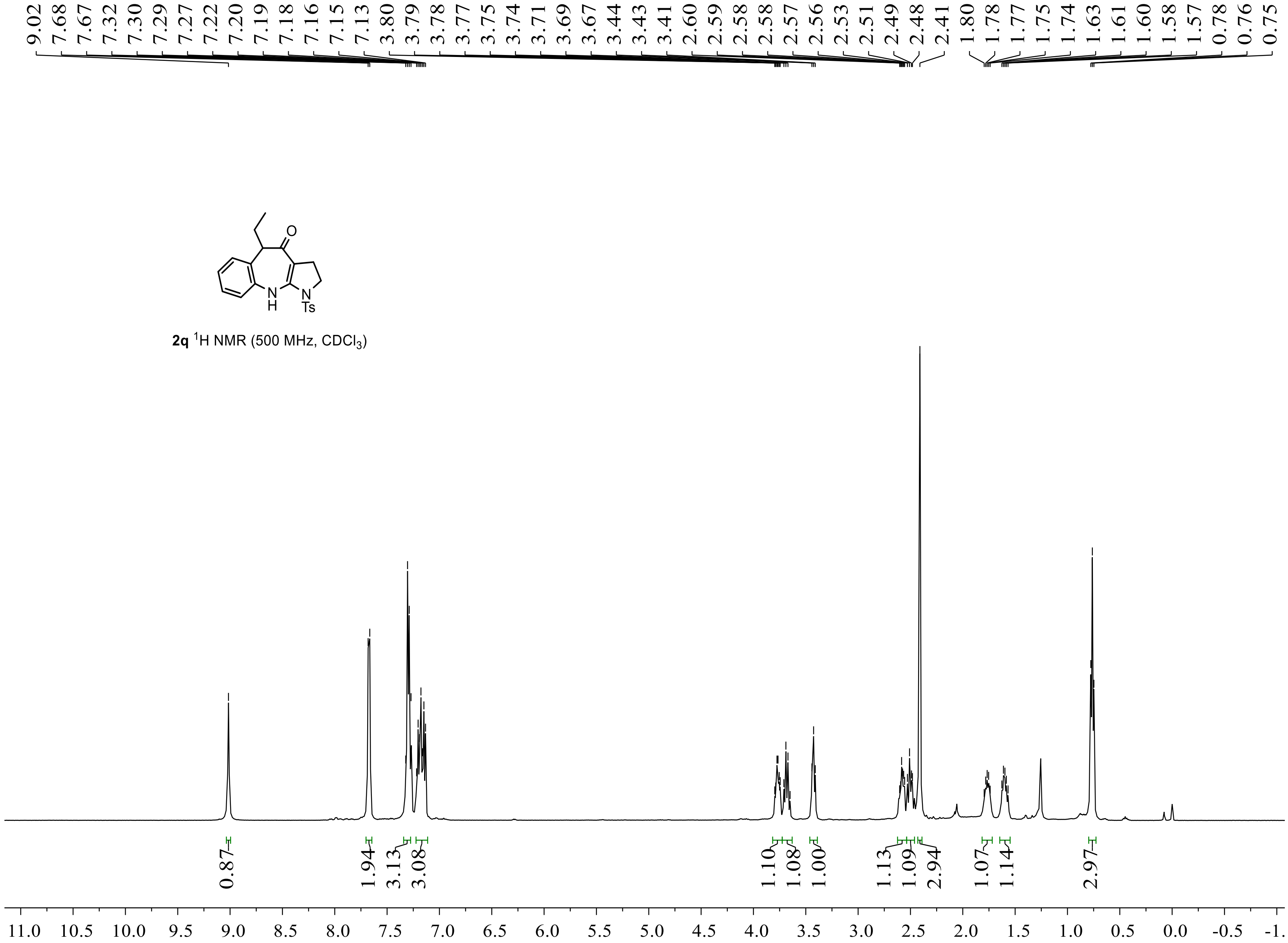

Supplementary Figure 97. ^{1}H NMR ($CDCl_3$, 500 MHz, 298 K) spectrum for **2q**

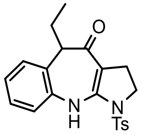

2q $^{13}C\{1H\}$ NMR (126 MHz, $CDCl_3$)

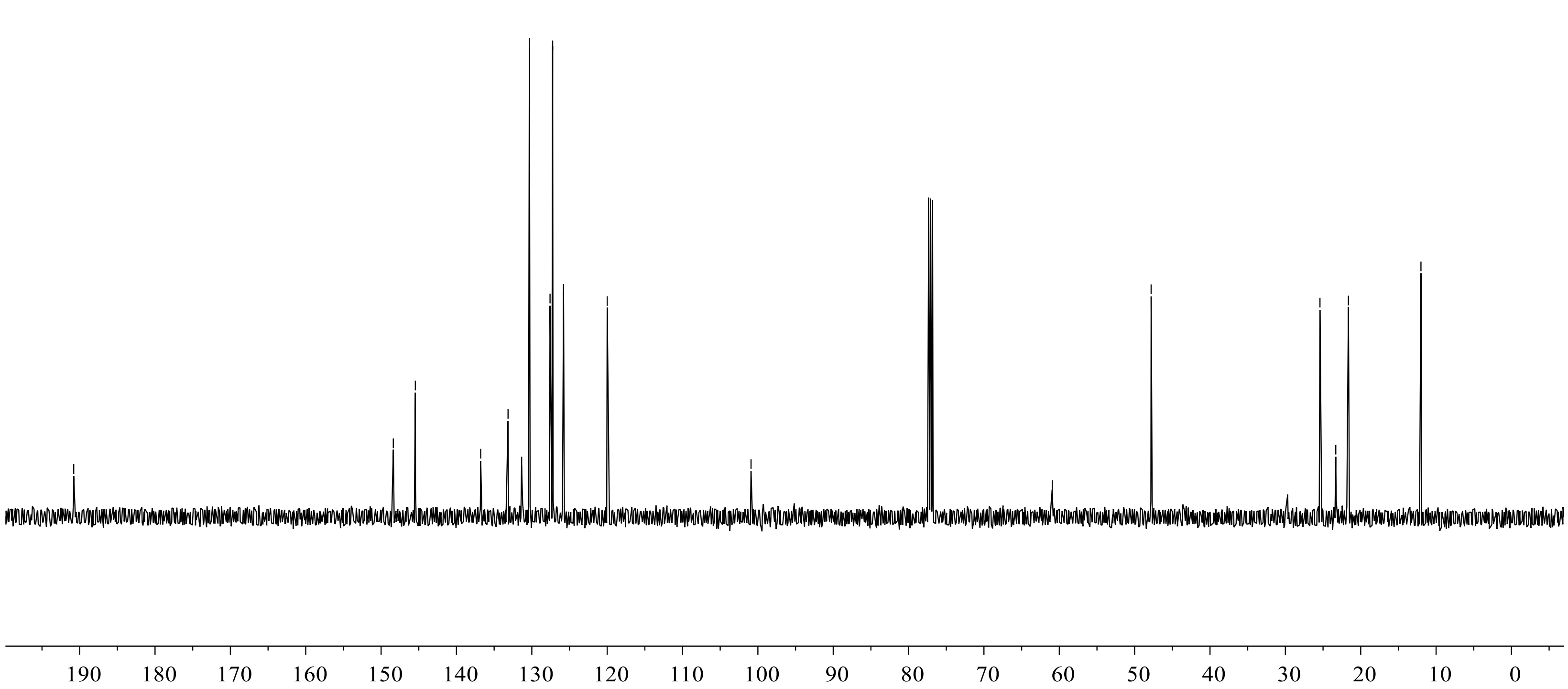

Supplementary Figure 98. ^{13}C NMR ($CDCl_3$, 126 MHz, 298 K) spectrum for **2q**

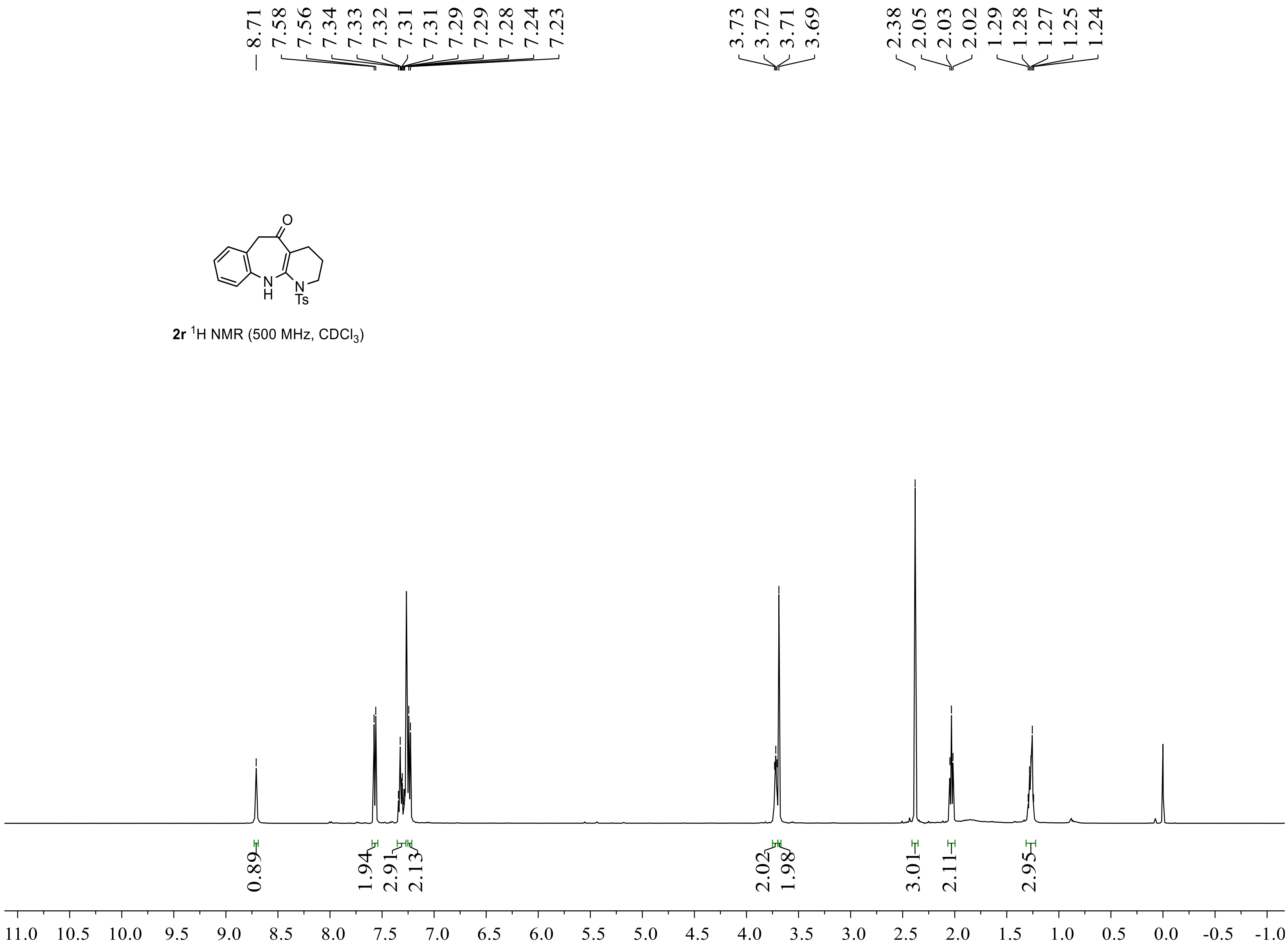

Supplementary Figure 99. ^{1}H NMR ($CDCl_3$, 500 MHz, 298 K) spectrum for **2r**

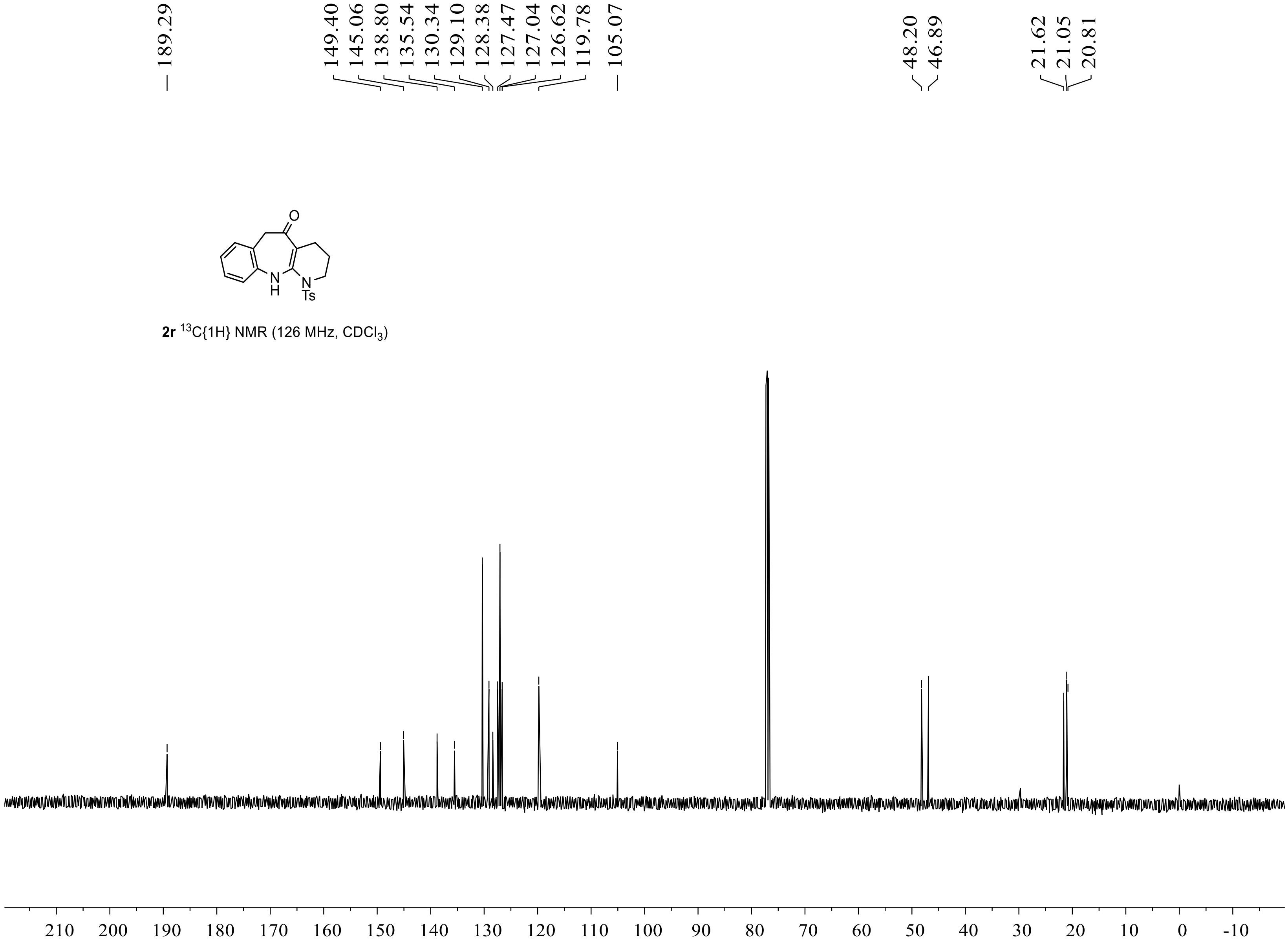

Supplementary Figure 100. ^{13}C NMR ($CDCl_3$, 126 MHz, 298 K) spectrum for **2r**

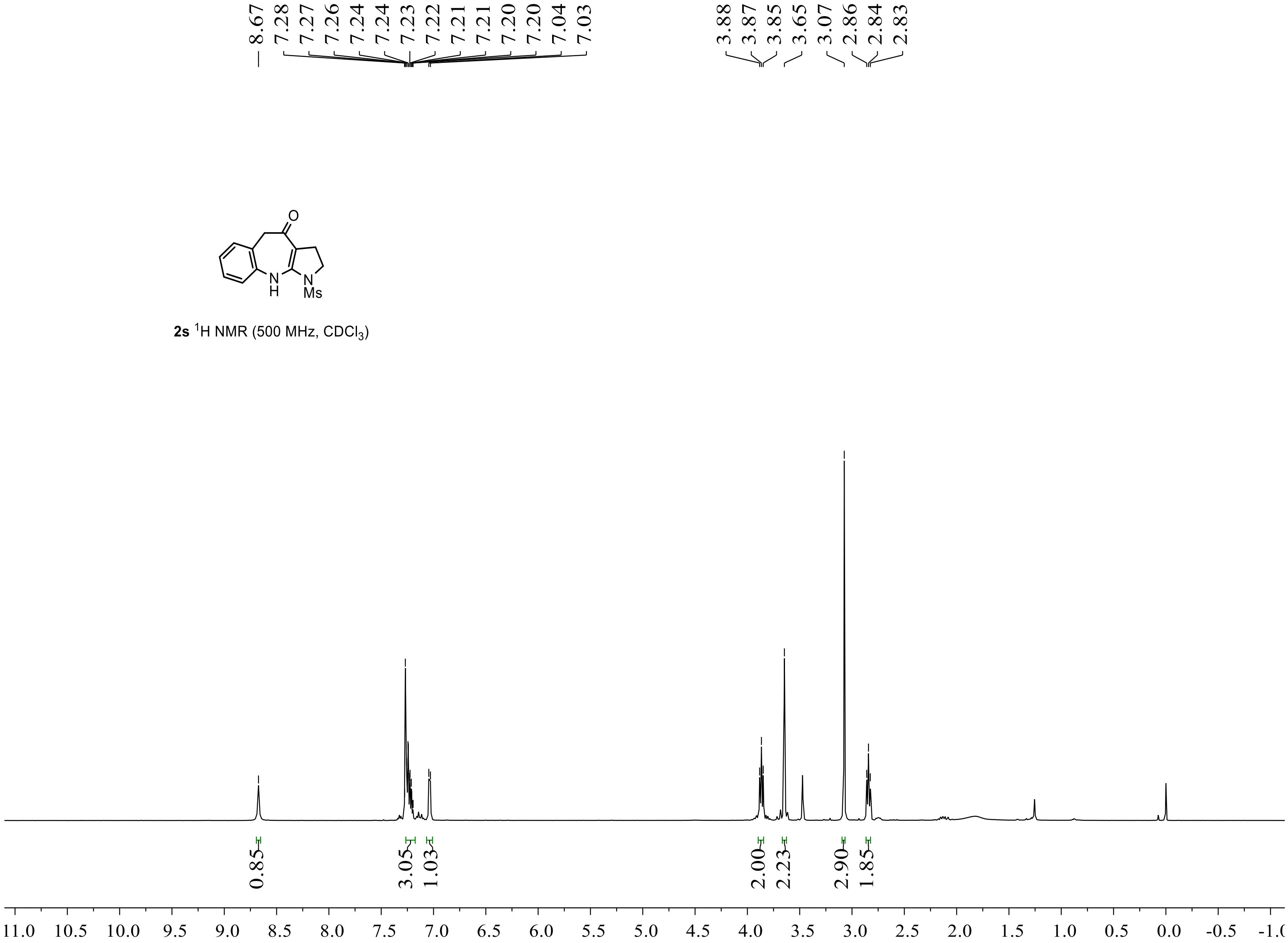

Supplementary Figure 101. ^{1}H NMR ($CDCl_3$, 500 MHz, 298 K) spectrum for **2s**

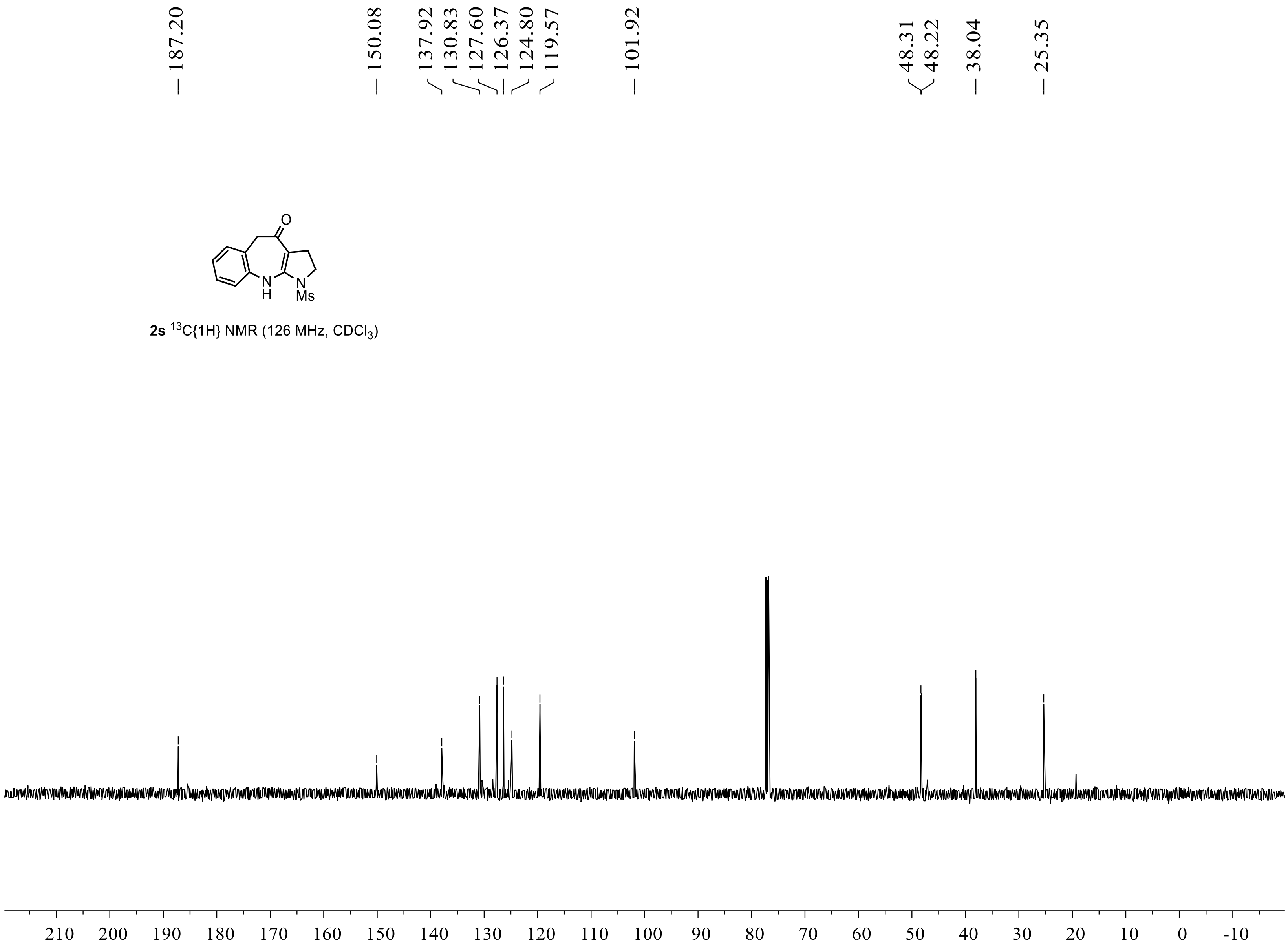

Supplementary Figure 102. ^{13}C NMR ($CDCl_3$, 126 MHz, 298 K) spectrum for **2s**

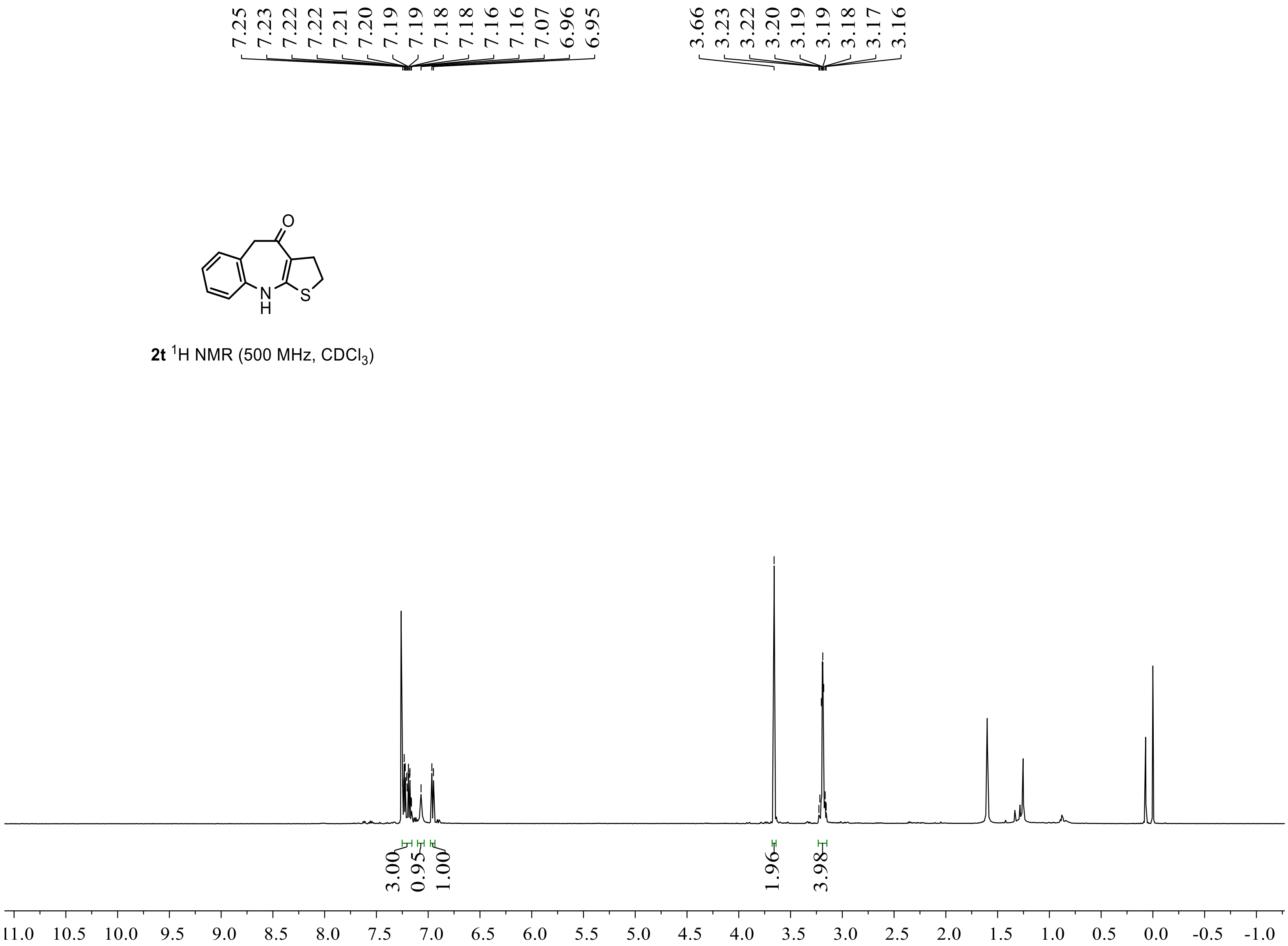

Supplementary Figure 103. ^{1}H NMR ($CDCl_3$, 500 MHz, 298 K) spectrum for **2t**

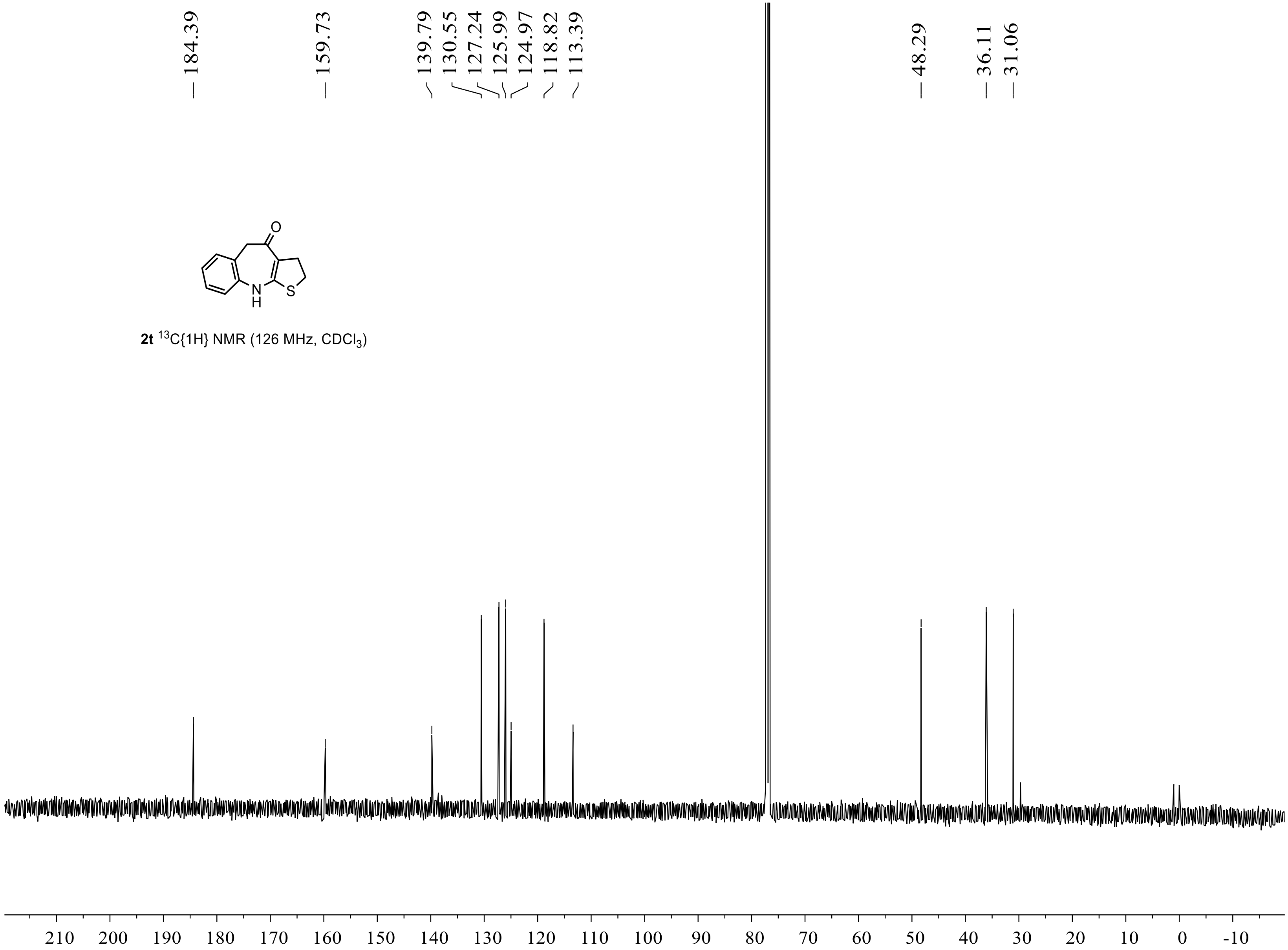

Supplementary Figure 104. ^{13}C NMR ($CDCl_3$, 126 MHz, 298 K) spectrum for **2t**

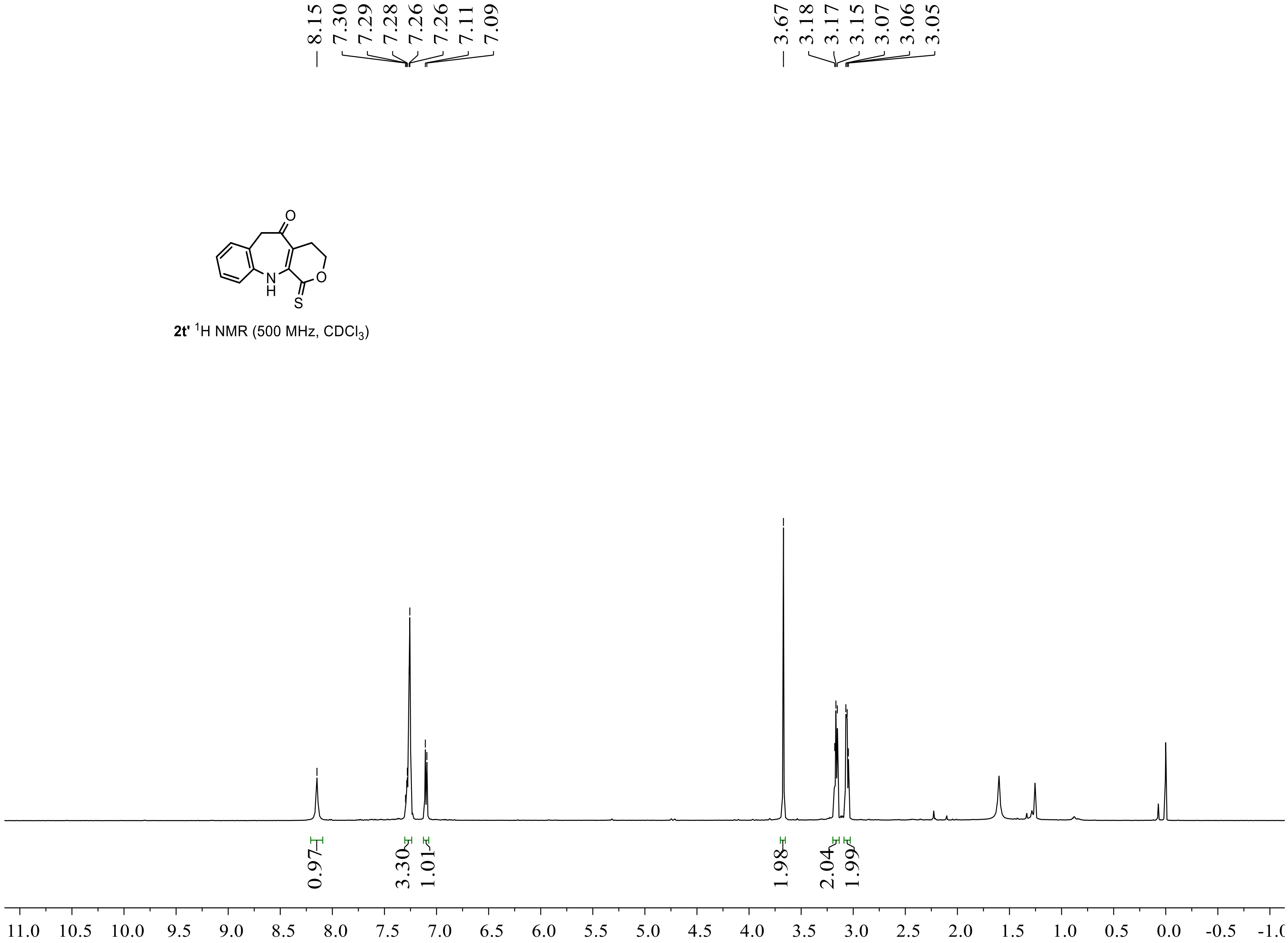

Supplementary Figure 105. ^{1}H NMR ($CDCl_3$, 500 MHz, 298 K) spectrum for **2t'**

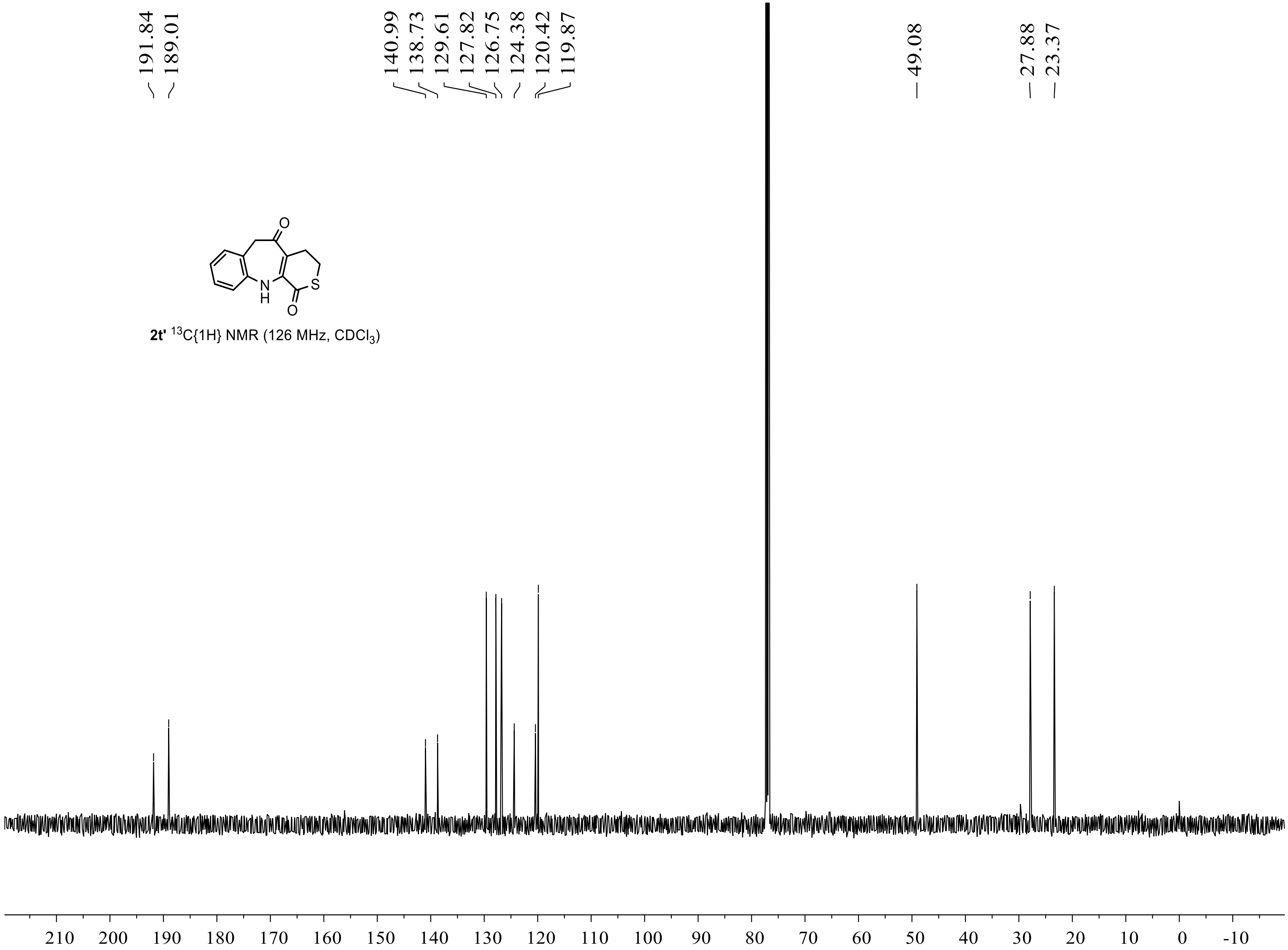

Supplementary Figure 106. ^{13}C NMR ($CDCl_3$, 126 MHz, 298 K) spectrum for **2t'**

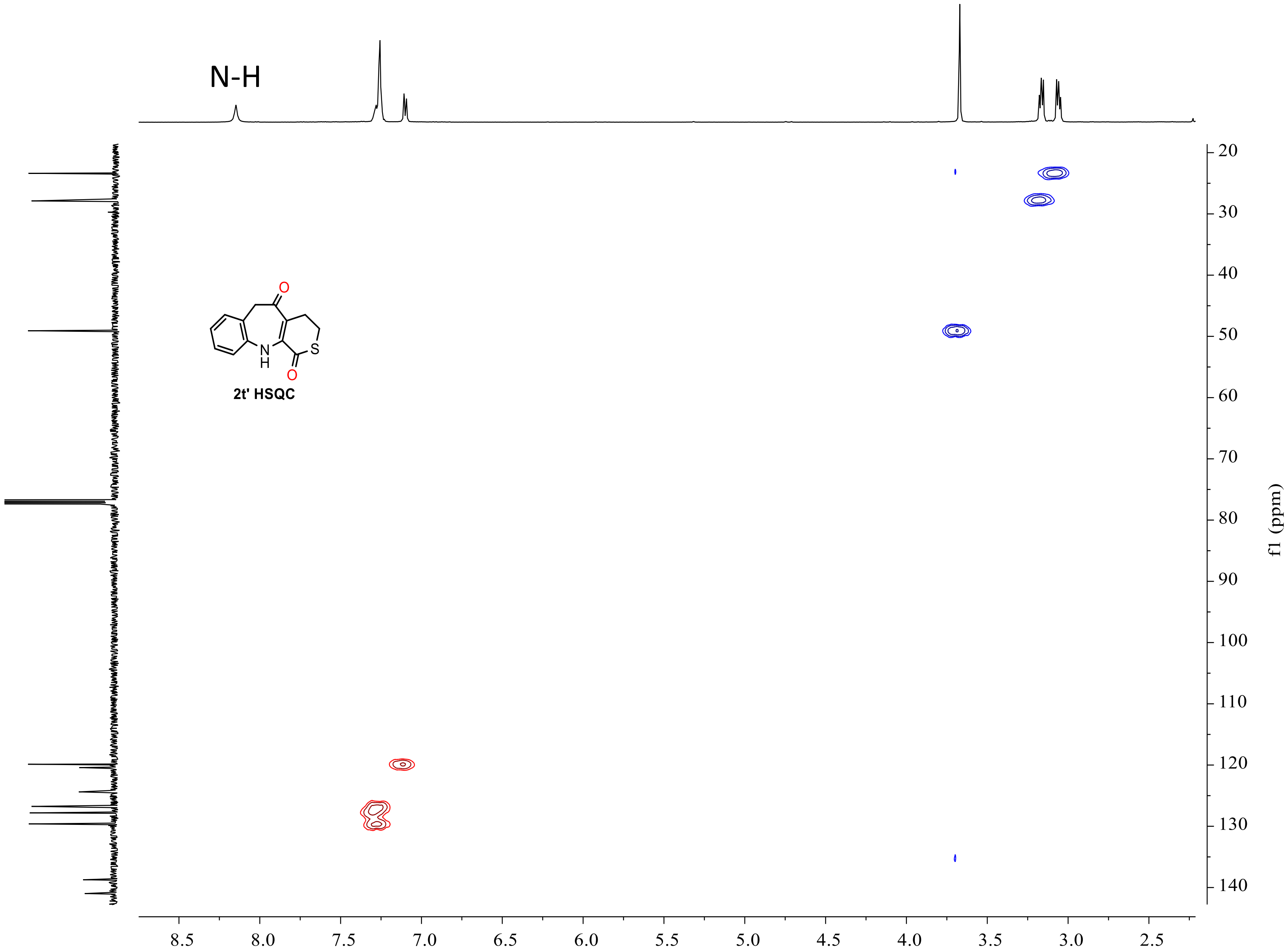

Supplementary Figure 107. HSQC ($CDCl_3$, 298 K) spectrum for **2t’**

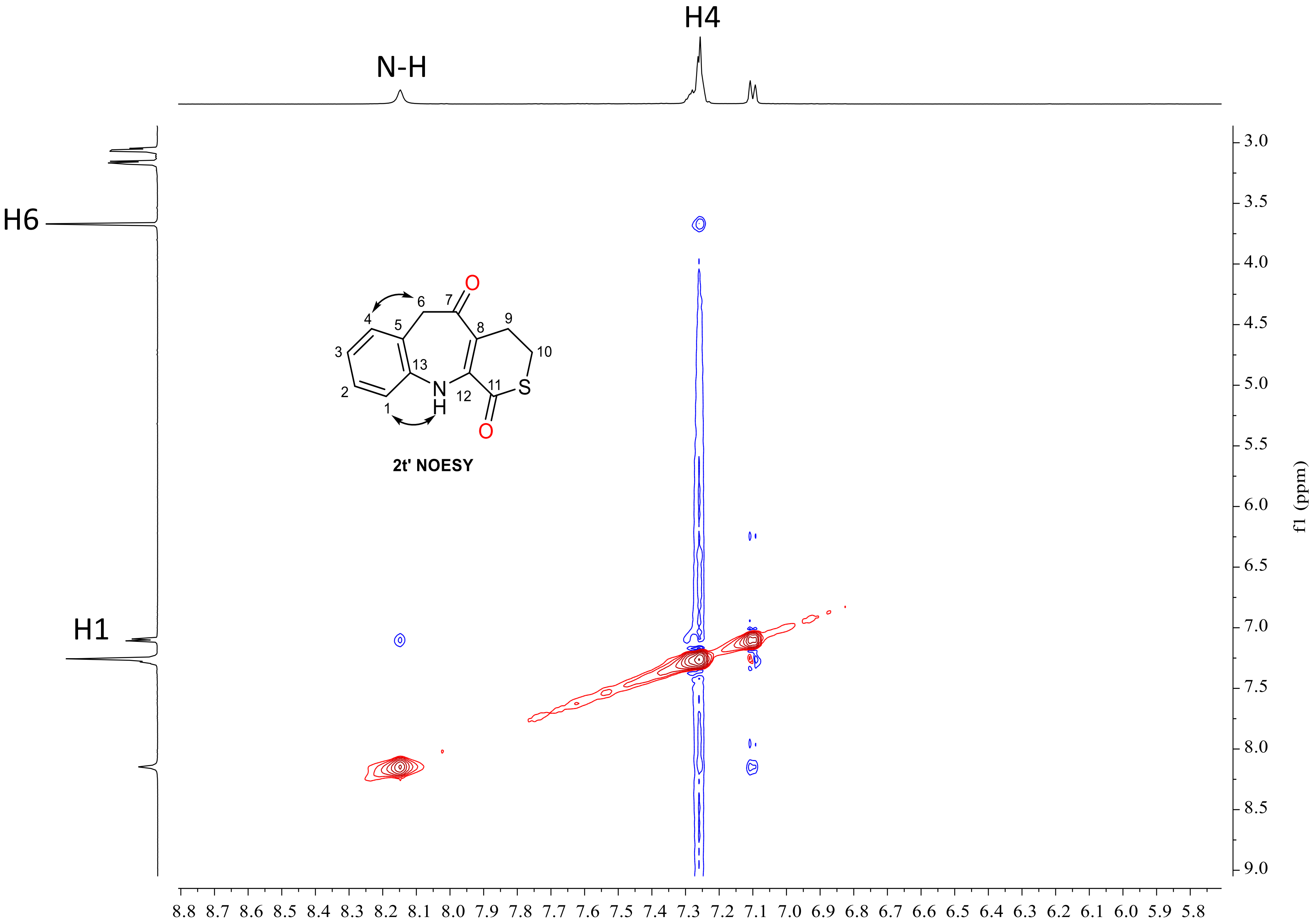

Supplementary Figure 108. NOESY ($CDCl_3$, 298 K) spectrum for **2t'**

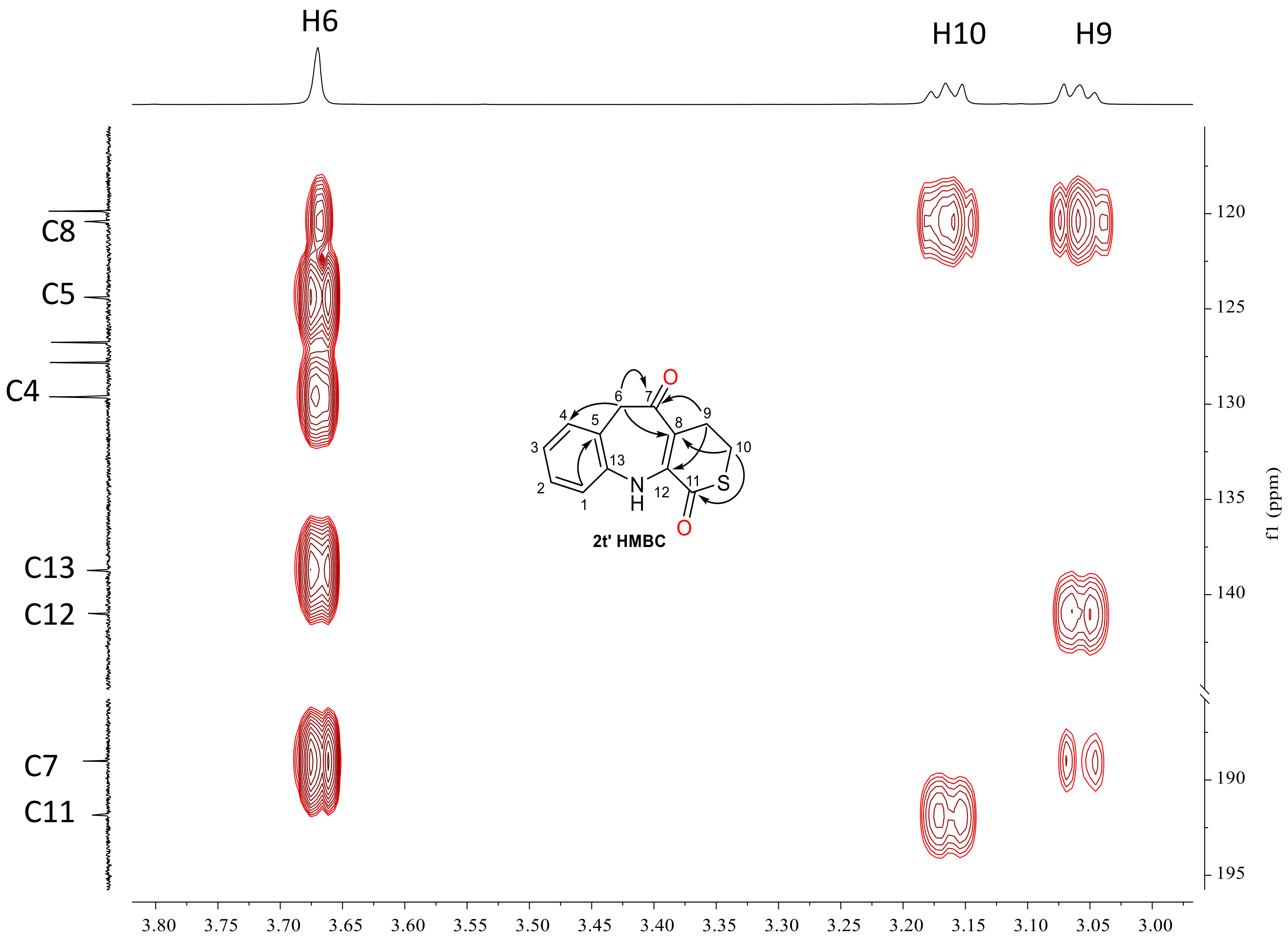

Supplementary Figure 109. HMBC ($CDCl_3$, 298 K) spectrum for **2t'**

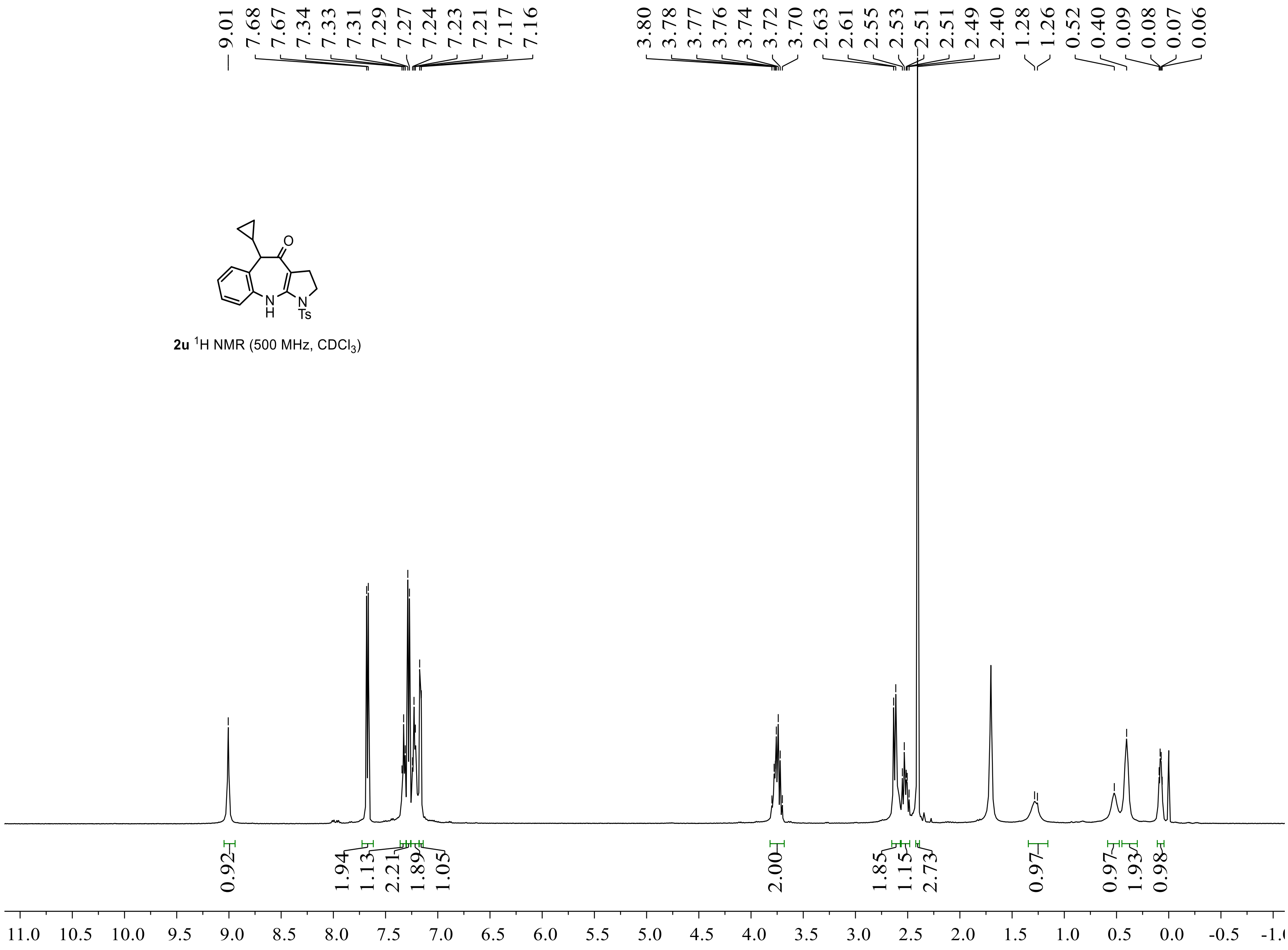

Supplementary Figure 110. ^{1}H NMR ($CDCl_3$, 500 MHz, 298 K) spectrum for **2u**

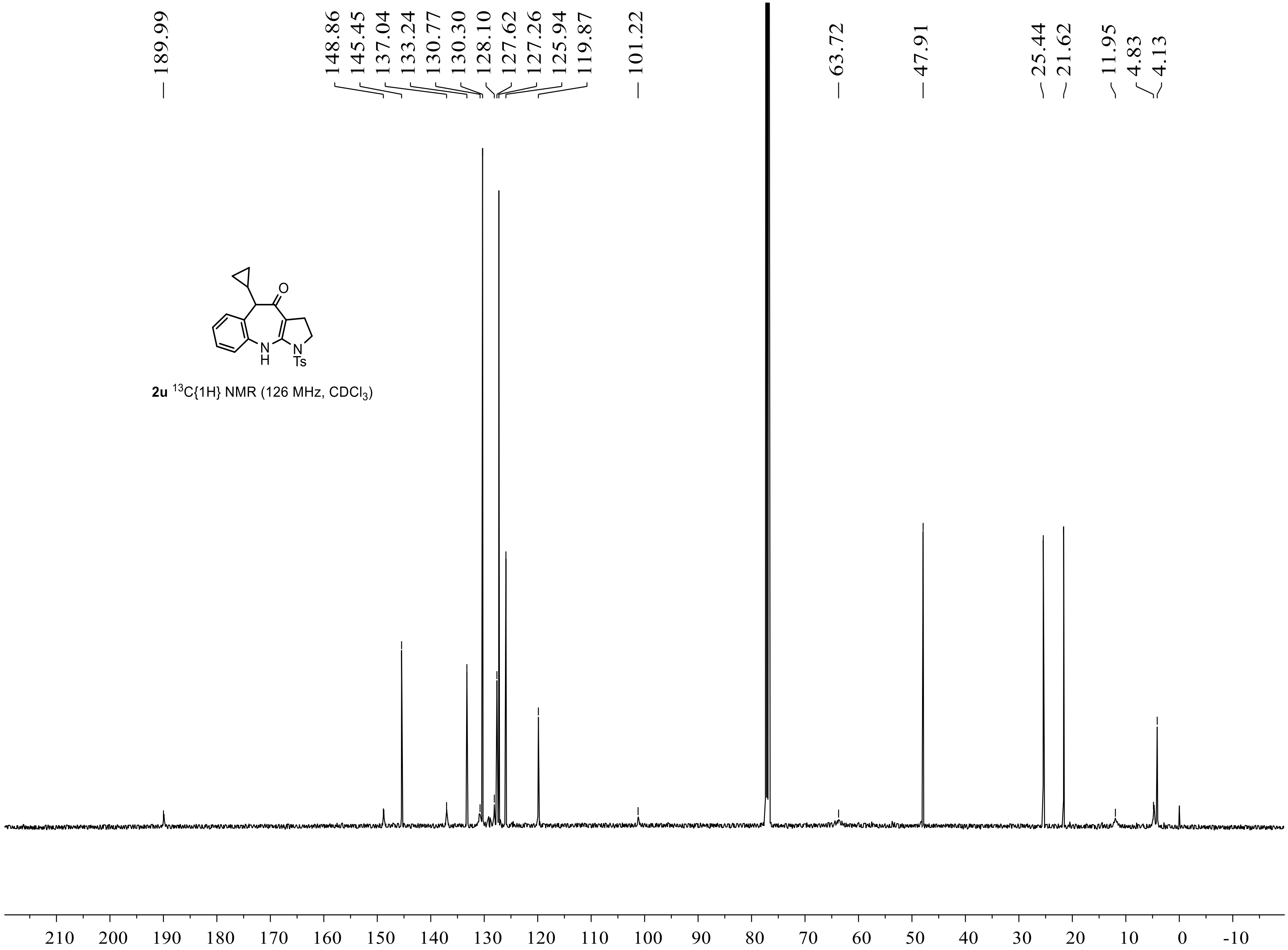

Supplementary Figure 111. ^{13}C NMR ($CDCl_3$, 126 MHz, 298 K) spectrum for **2u**

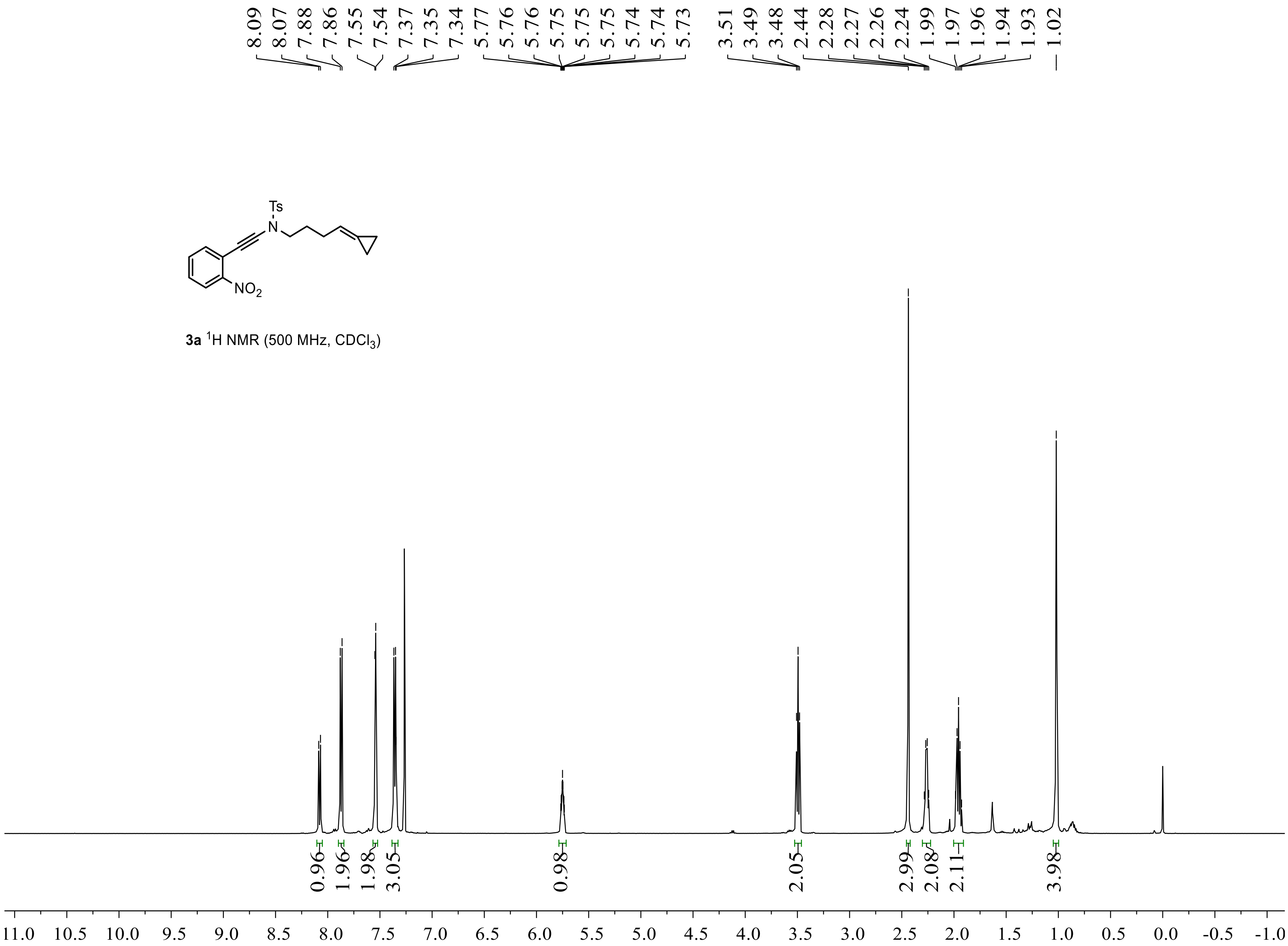

Supplementary Figure 112. ^{1}H NMR ($CDCl_3$, 500 MHz, 298 K) spectrum for **3a**

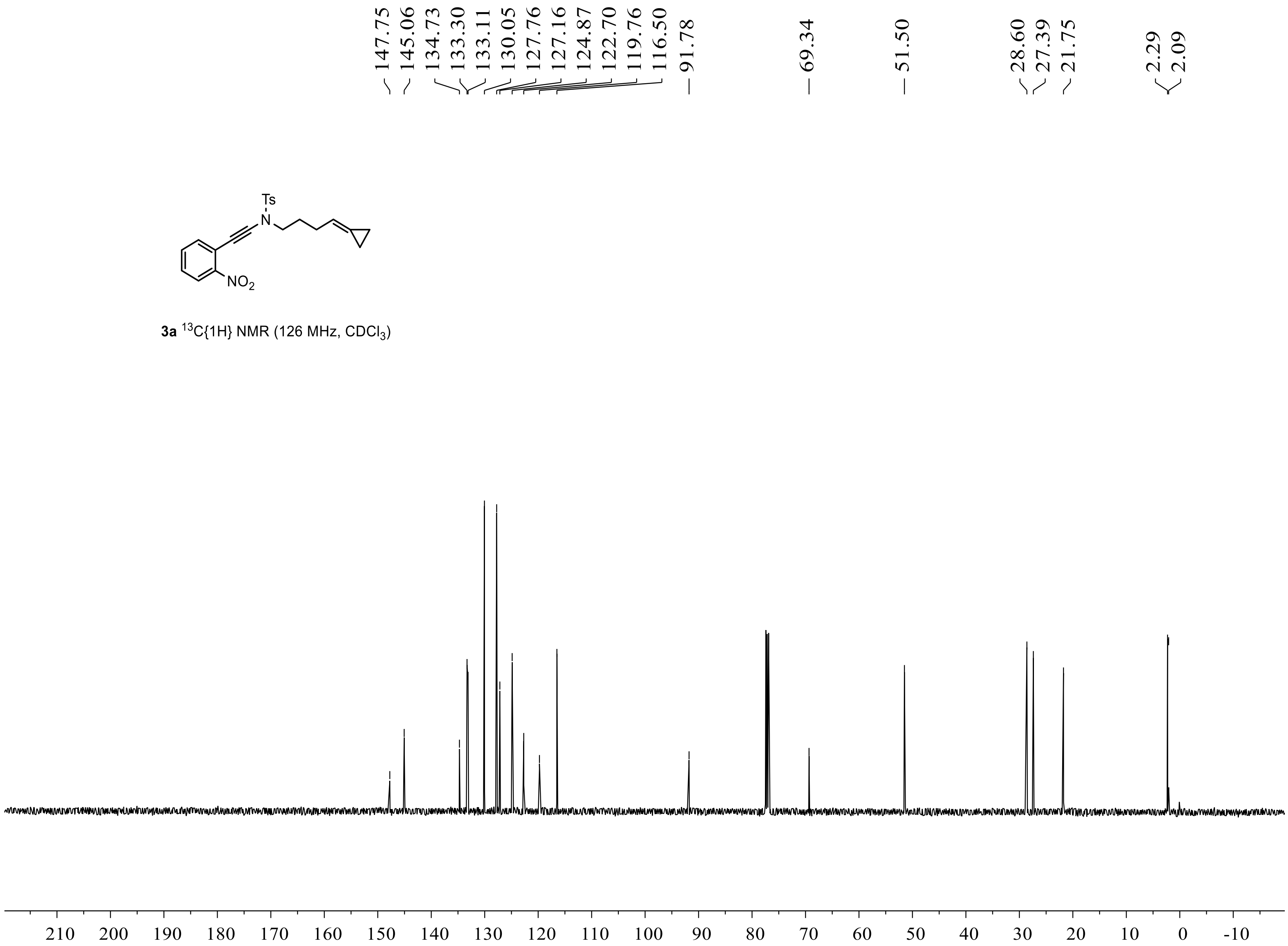

Supplementary Figure 113. ^{13}C NMR ($CDCl_3$, 126 MHz, 298 K) spectrum for **3a**

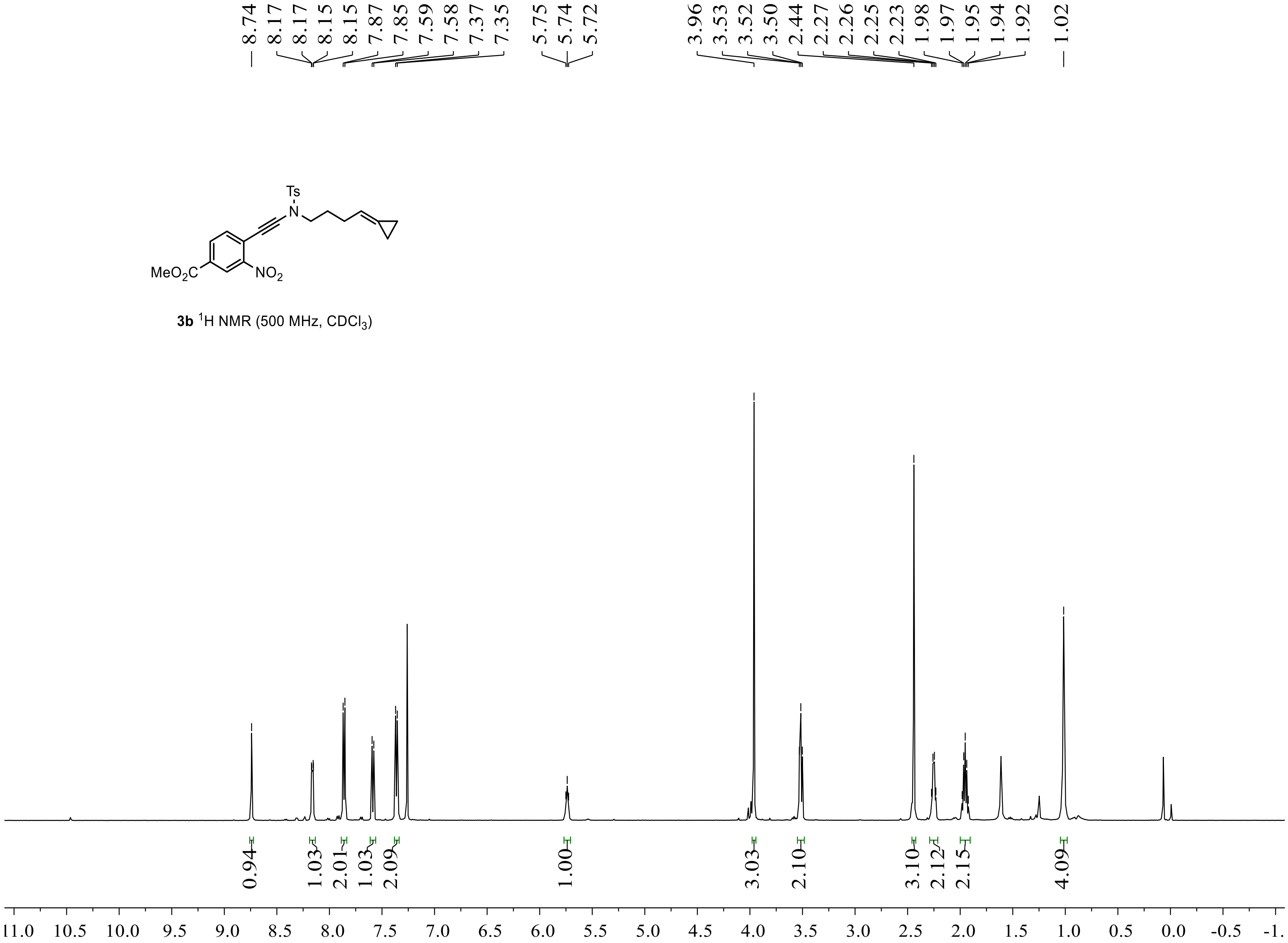

Supplementary Figure 114. ^{1}H NMR ($CDCl_3$, 500 MHz, 298 K) spectrum for **3b**

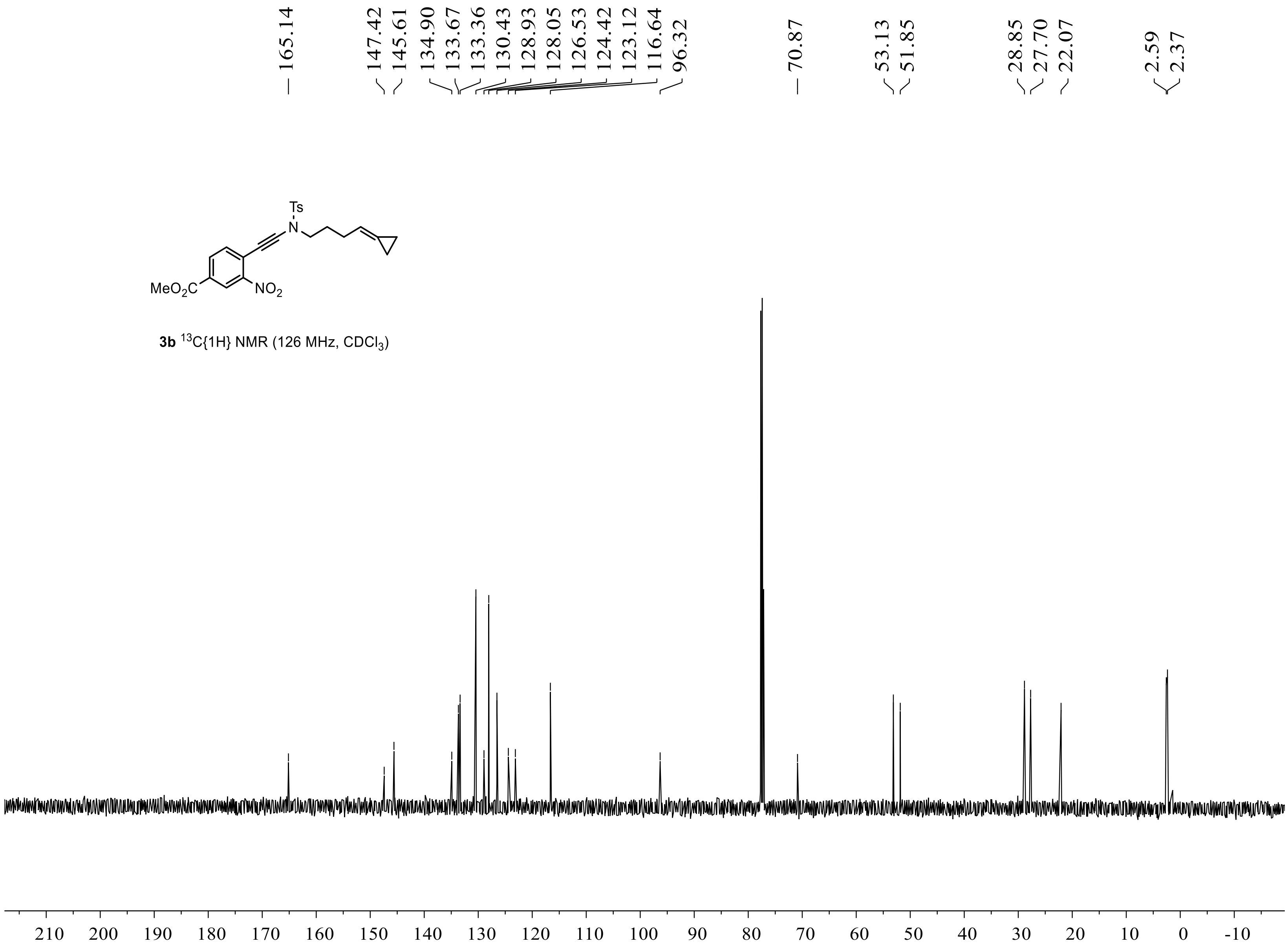

Supplementary Figure 115. ^{13}C NMR ($CDCl_3$, 126 MHz, 298 K) spectrum for **3b**

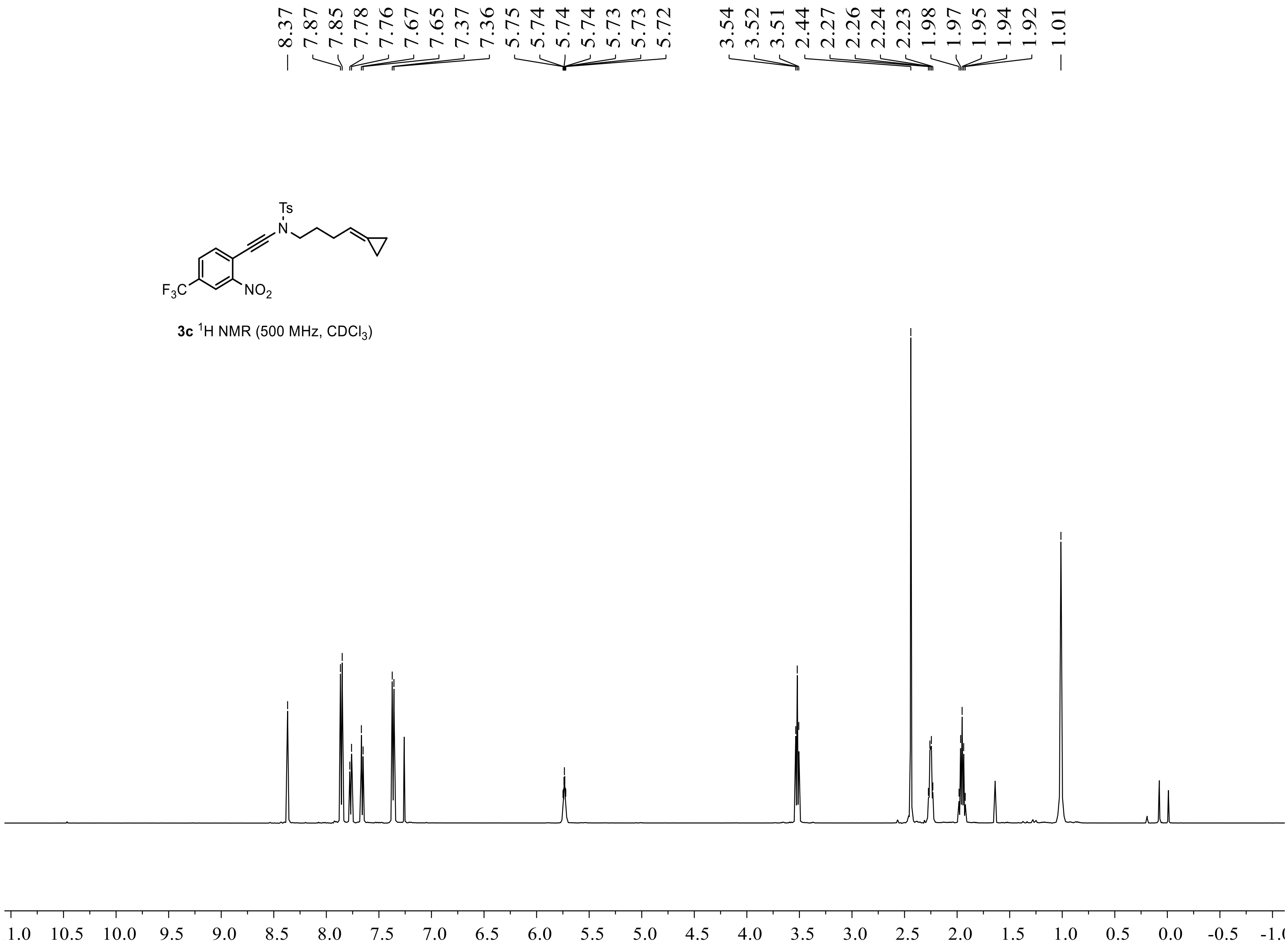

Supplementary Figure 116. ^{1}H NMR ($CDCl_3$, 500 MHz, 298 K) spectrum for **3c**

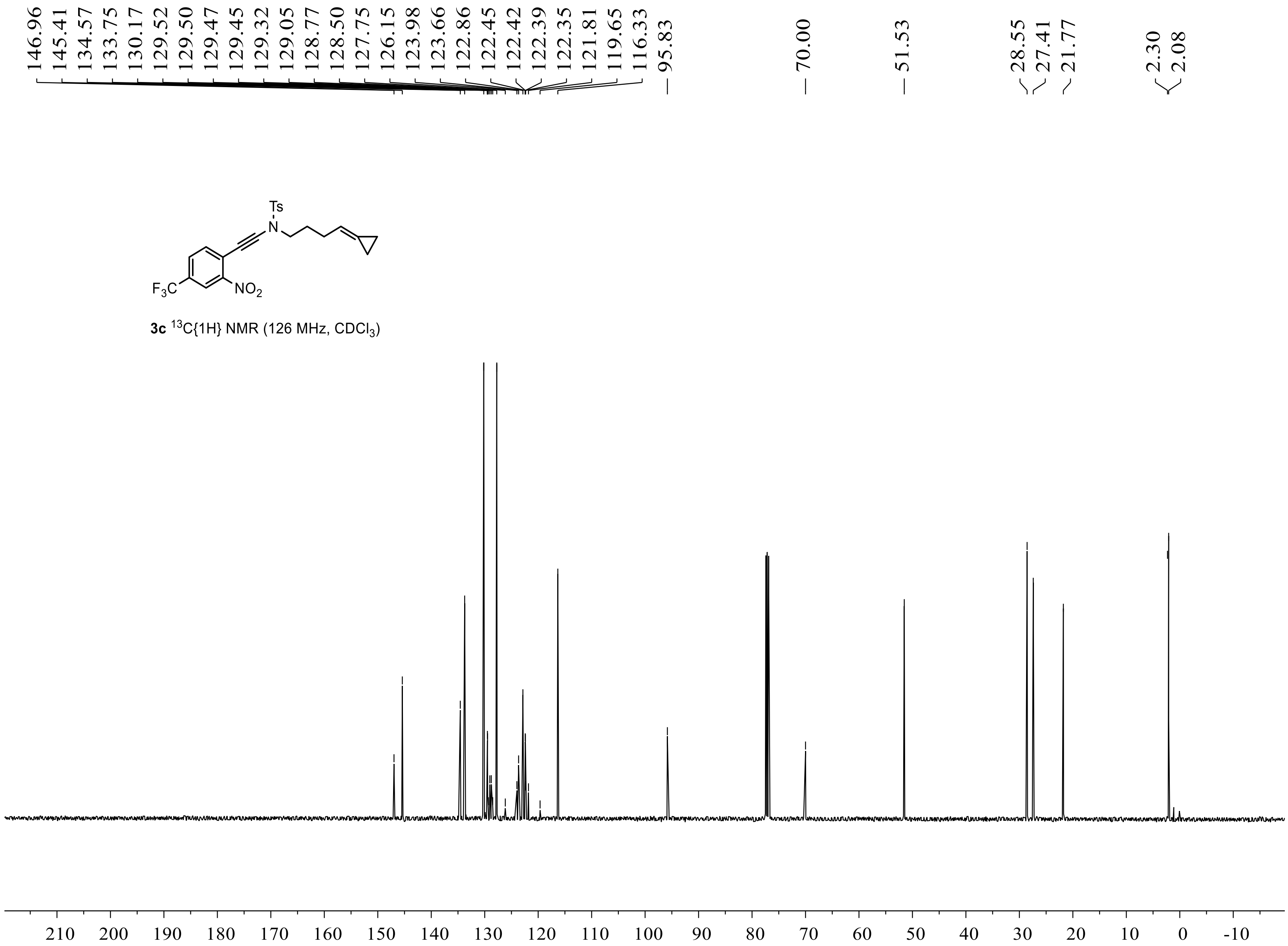

Supplementary Figure 117. ^{13}C NMR ($CDCl_3$, 126 MHz, 298 K) spectrum for **3c**

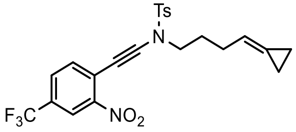

3c 119F NMR (471 MHz, CDCl3)

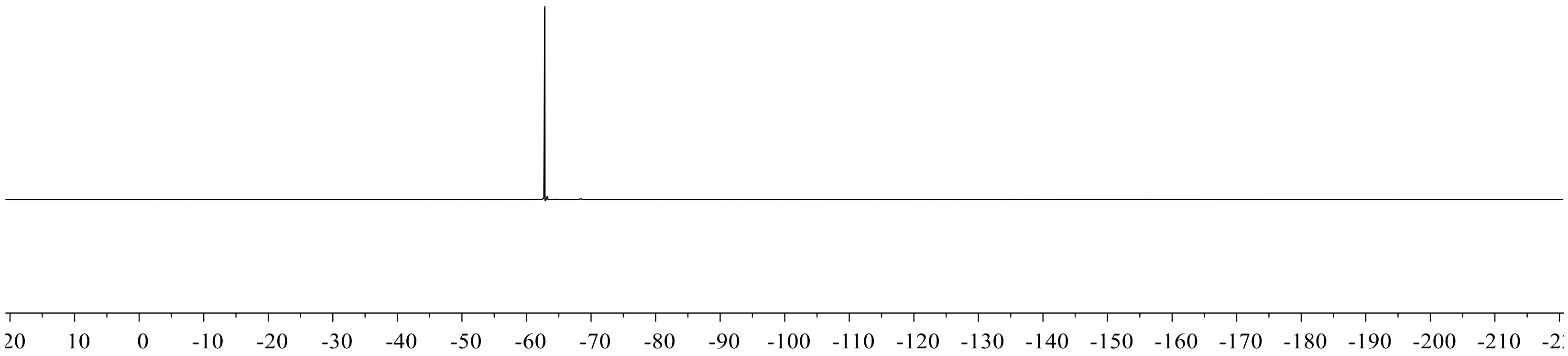

Supplementary Figure 118. ^{19}F NMR ($CDCl_3$, 471 MHz, 298 K) spectrum for **3c**

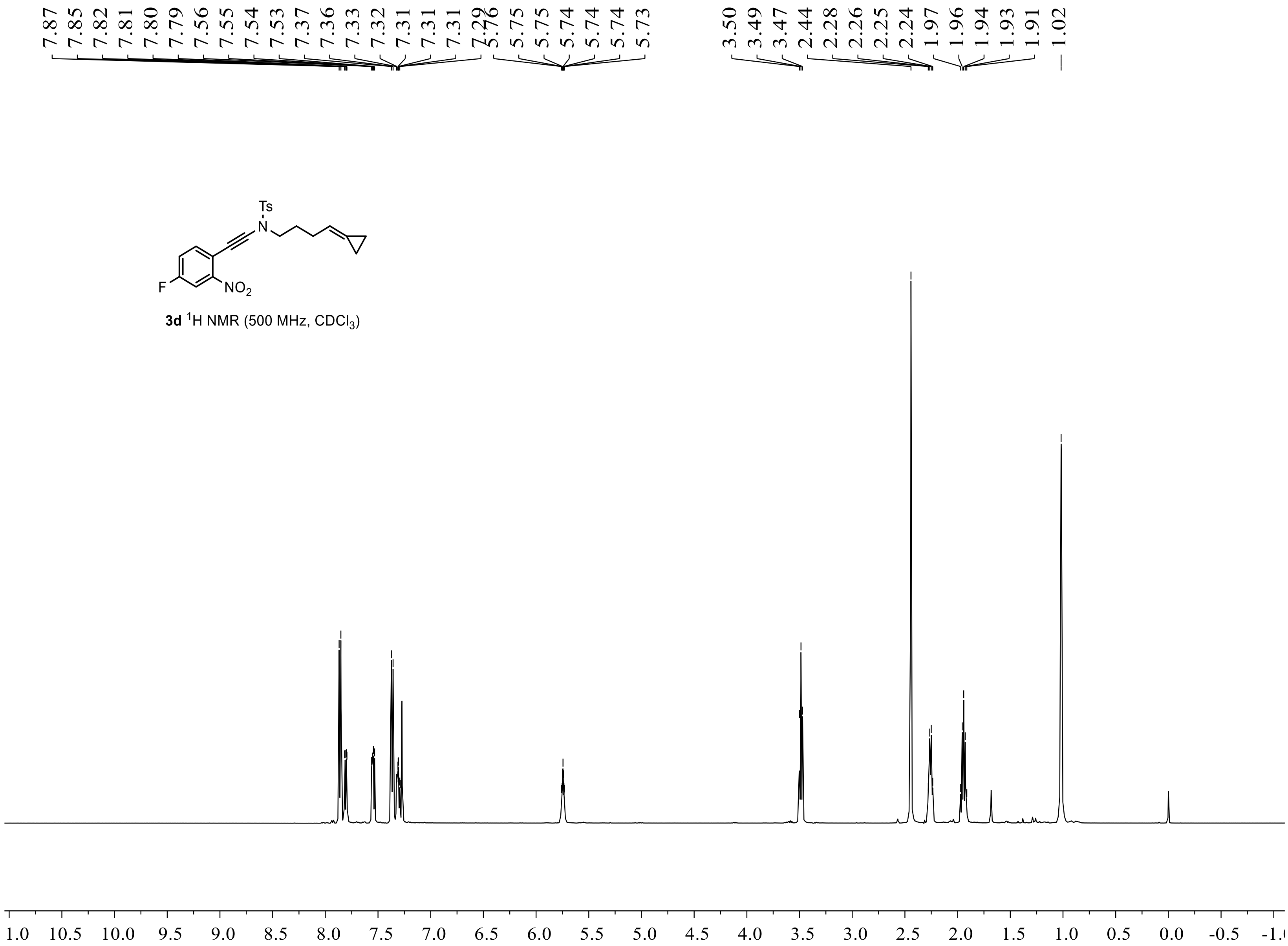

Supplementary Figure 119. ^{1}H NMR ($CDCl_3$, 500 MHz, 298 K) spectrum for **3d**

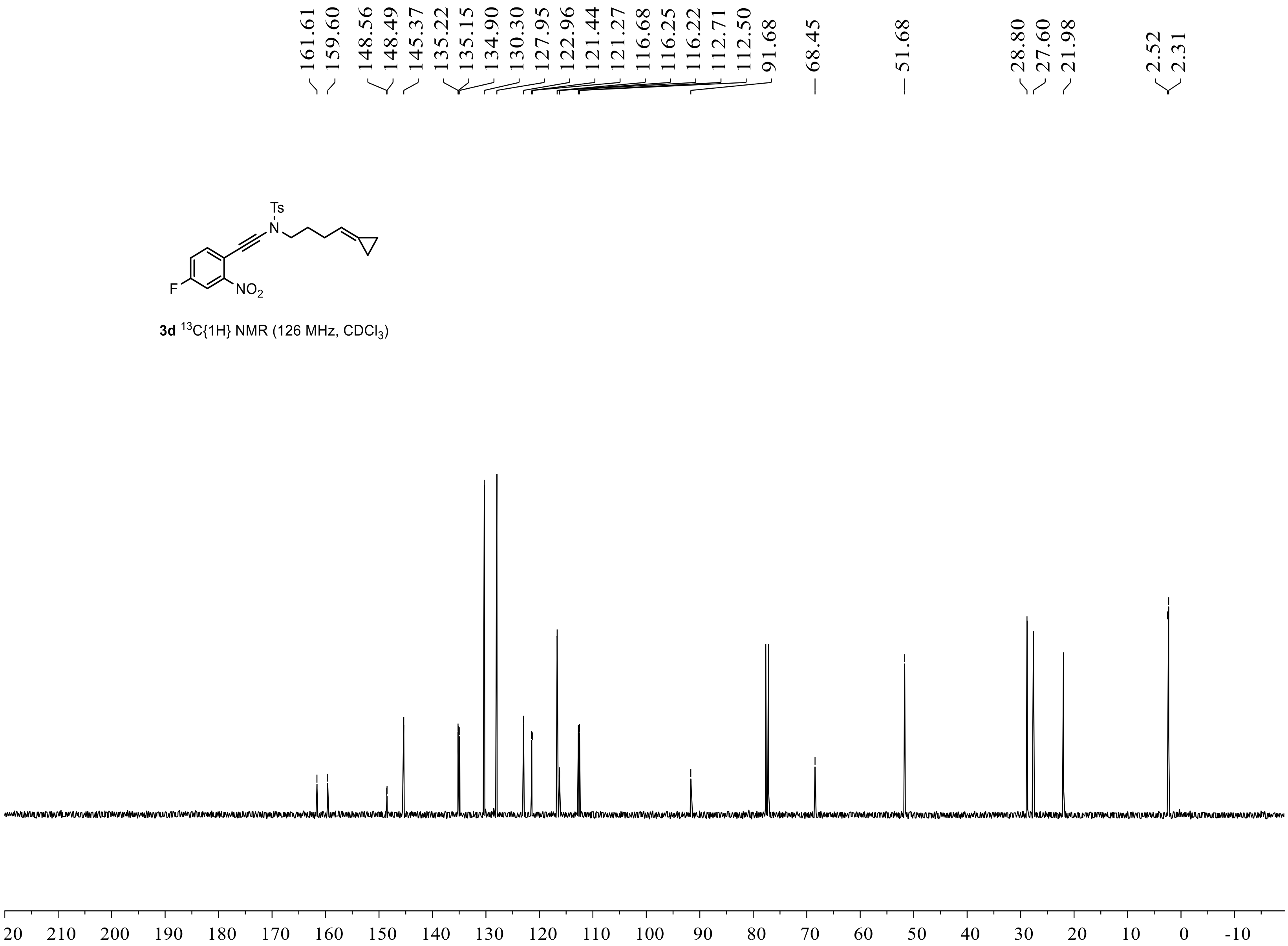

Supplementary Figure 120. ^{13}C NMR ($CDCl_3$, 126 MHz, 298 K) spectrum for **3d**

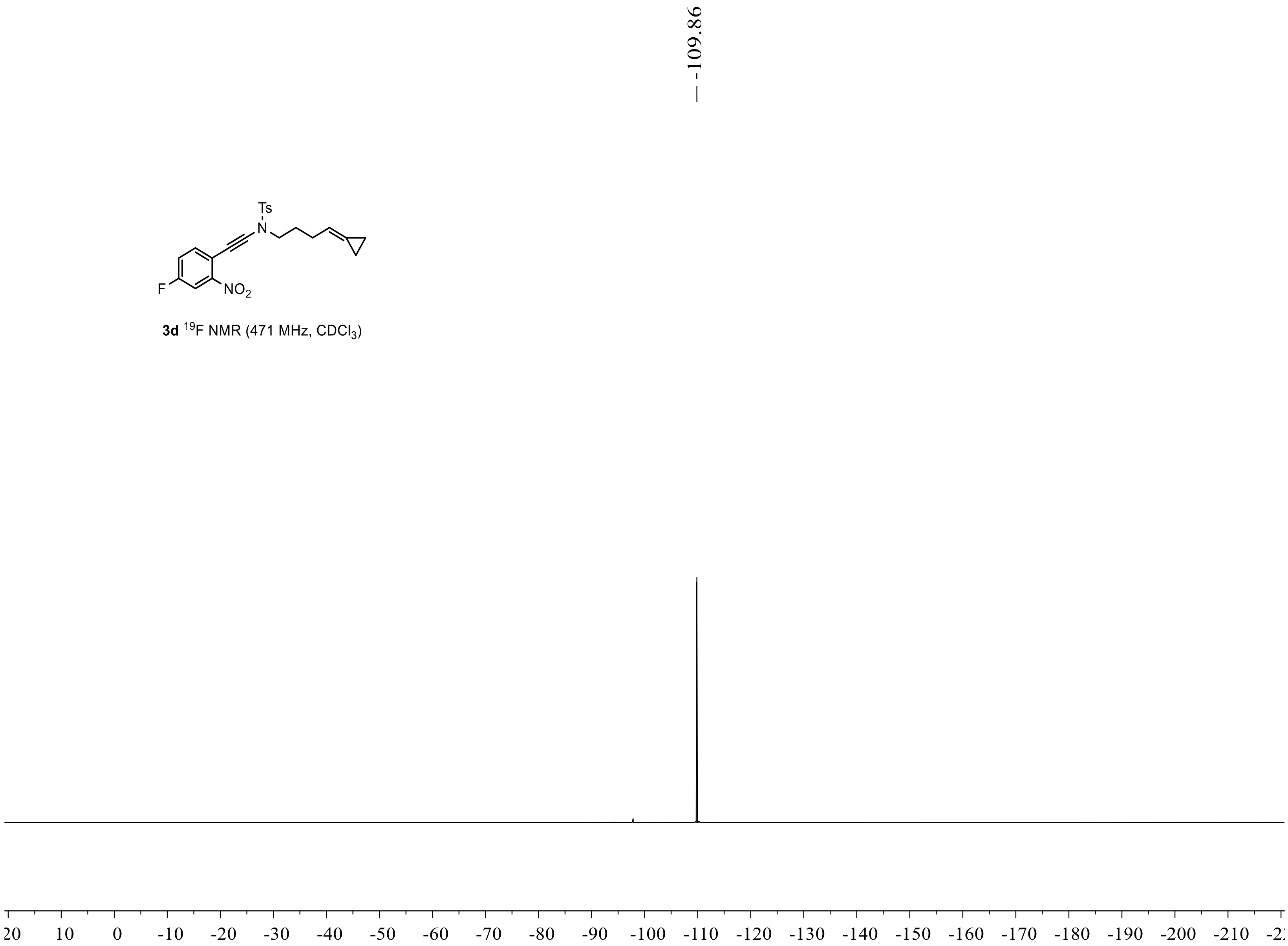

Supplementary Figure 121. ^{19}F NMR ($CDCl_3$, 471 MHz, 298 K) spectrum for **3d**

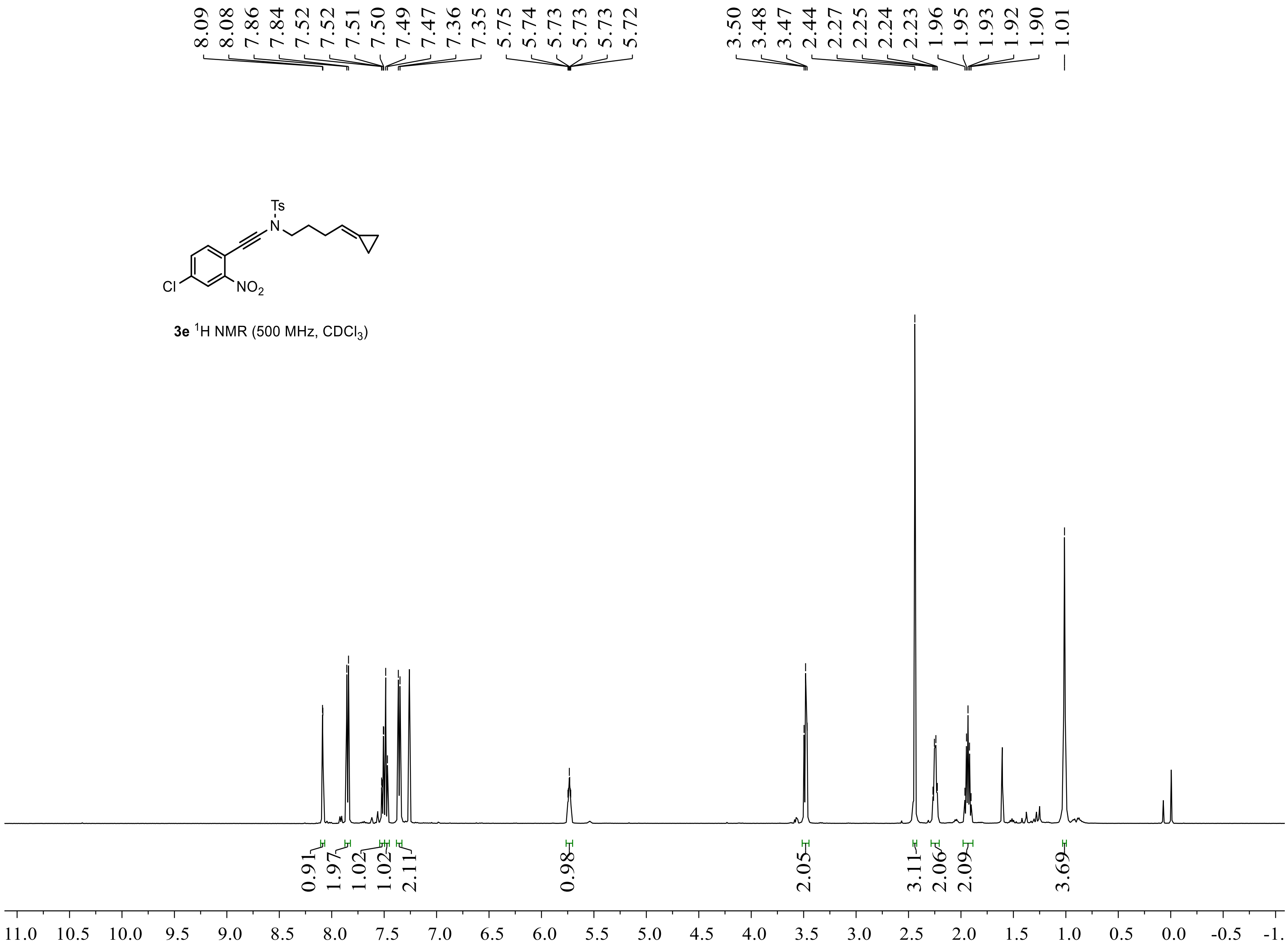

Supplementary Figure 122. ^{1}H NMR ($CDCl_3$, 500 MHz, 298 K) spectrum for **3e**

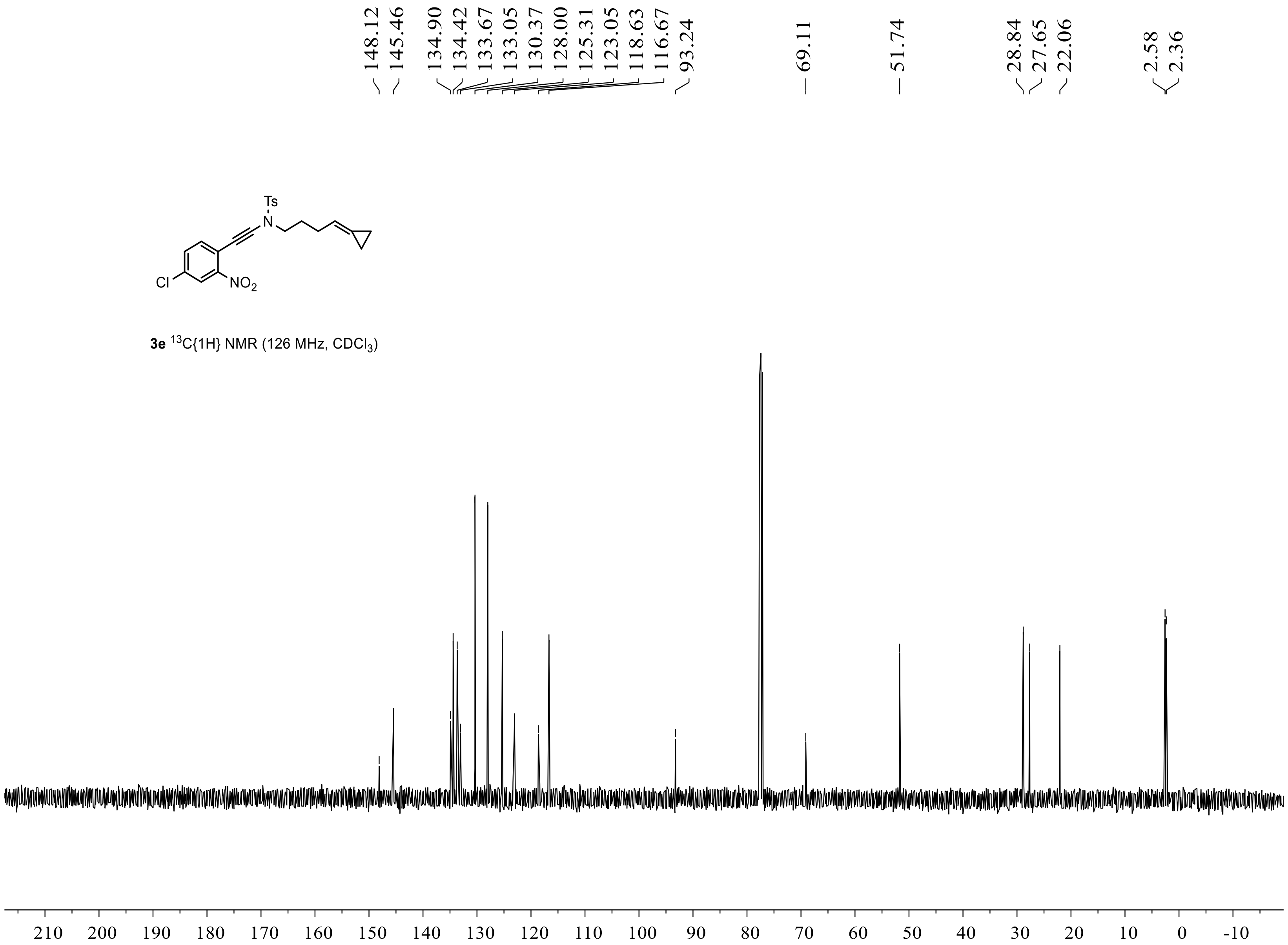

Supplementary Figure 123. ^{13}C NMR ($CDCl_3$, 126 MHz, 298 K) spectrum for **3e**

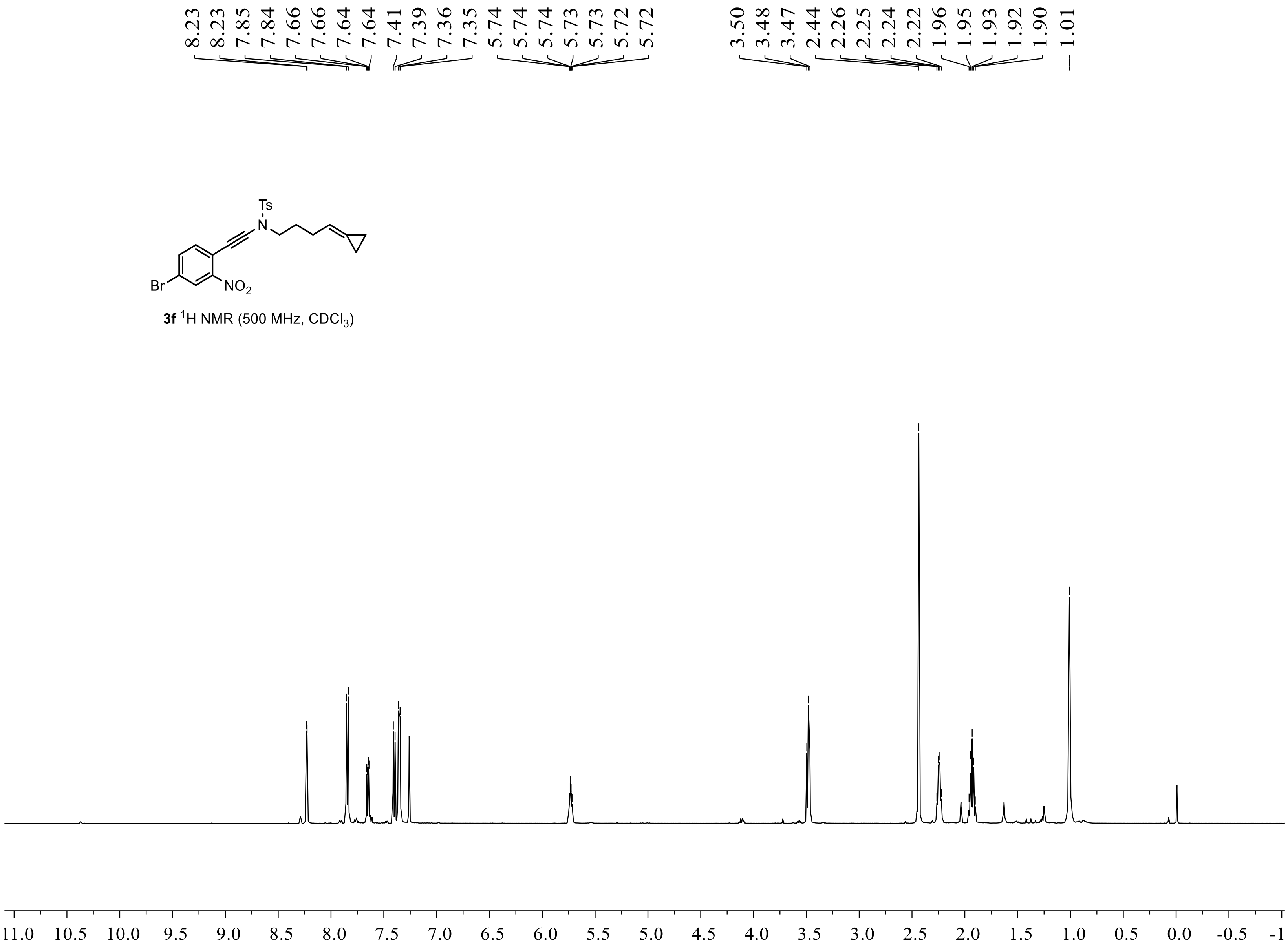

Supplementary Figure 124. ^{1}H NMR ($CDCl_3$, 500 MHz, 298 K) spectrum for **3f**

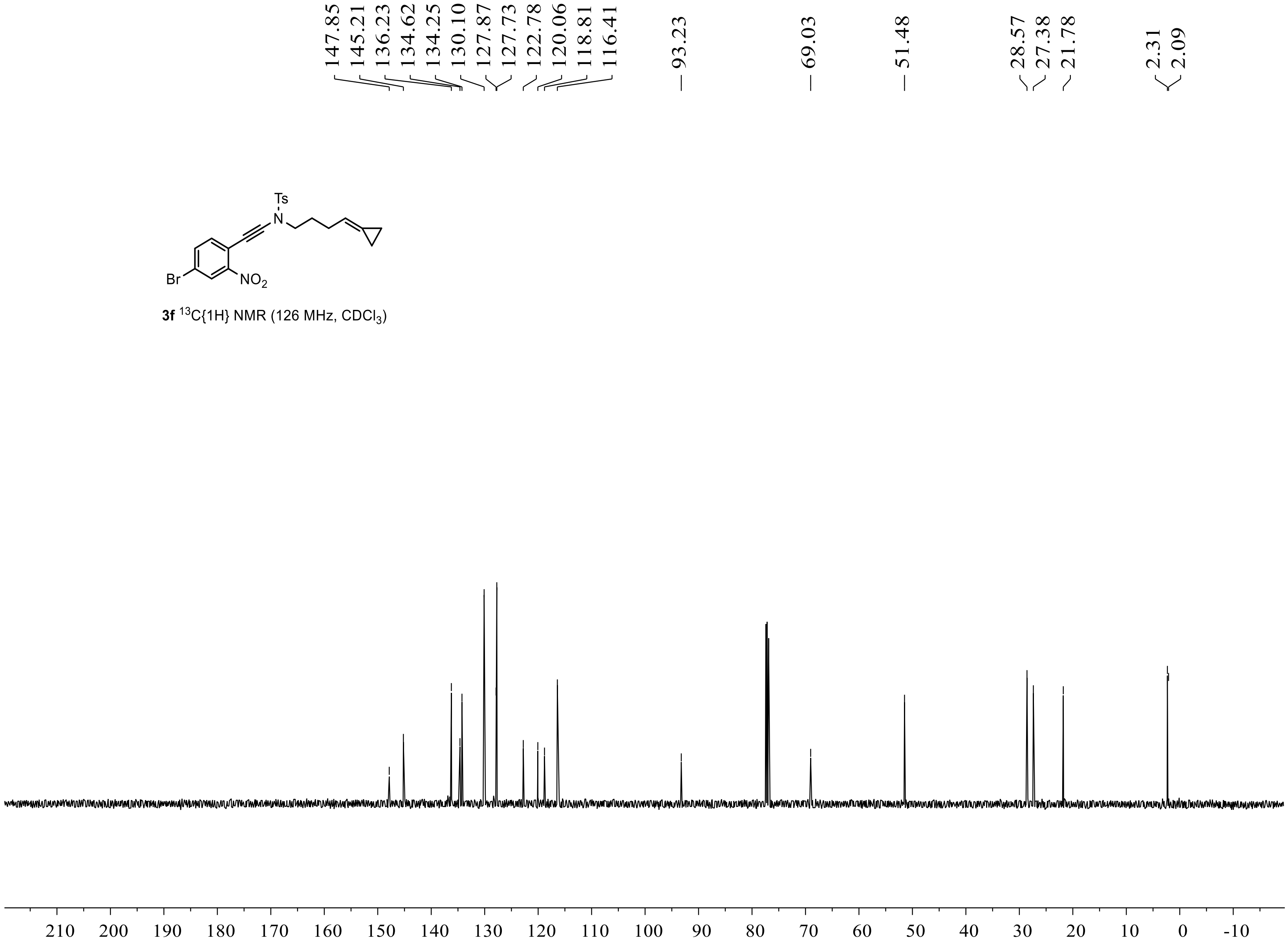

Supplementary Figure 125. ^{13}C NMR ($CDCl_3$, 126 MHz, 298 K) spectrum for **3f**

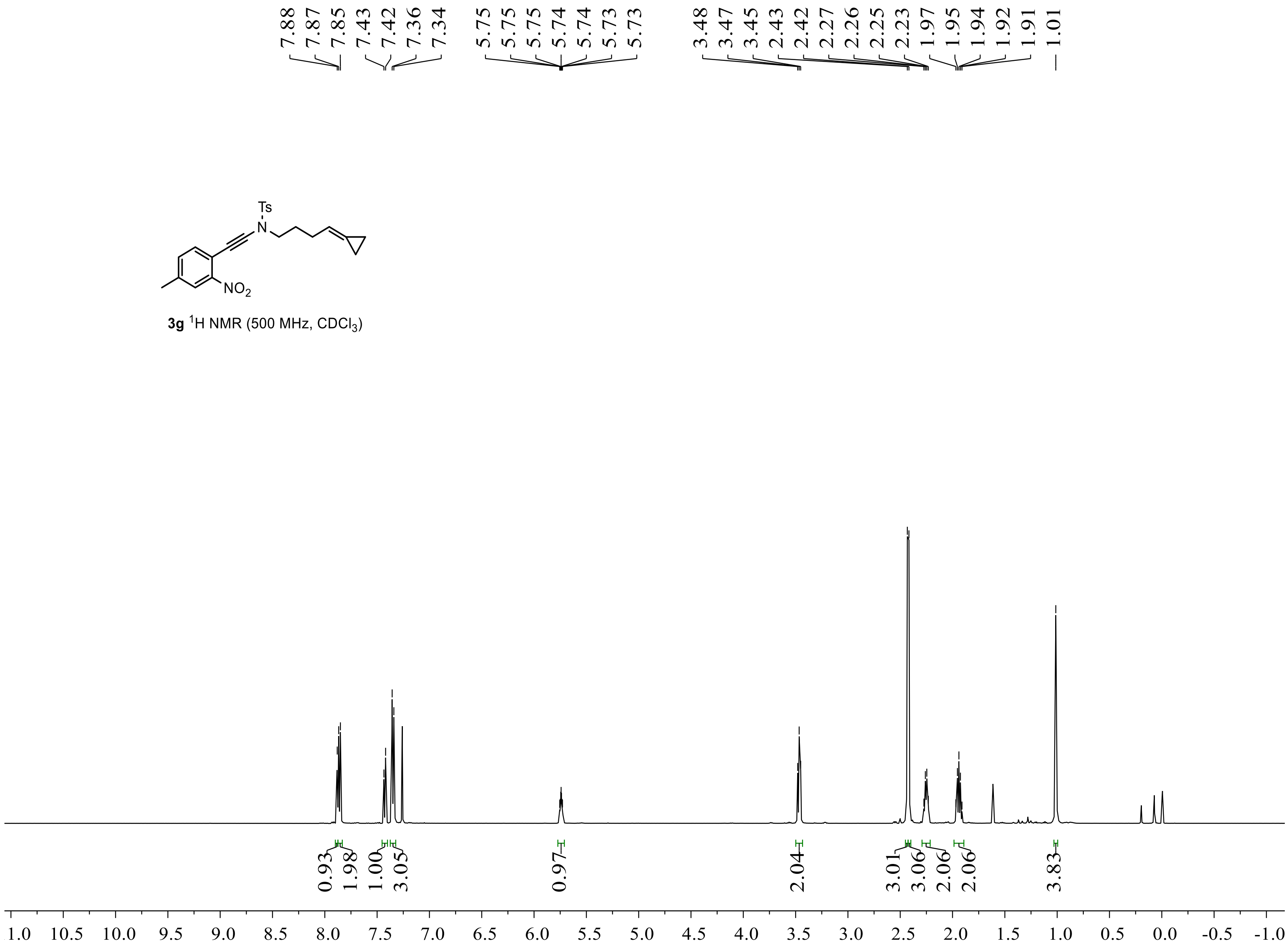

Supplementary Figure 126. ^1H NMR ($CDCl_3$, 500 MHz, 298 K) spectrum for **3g**

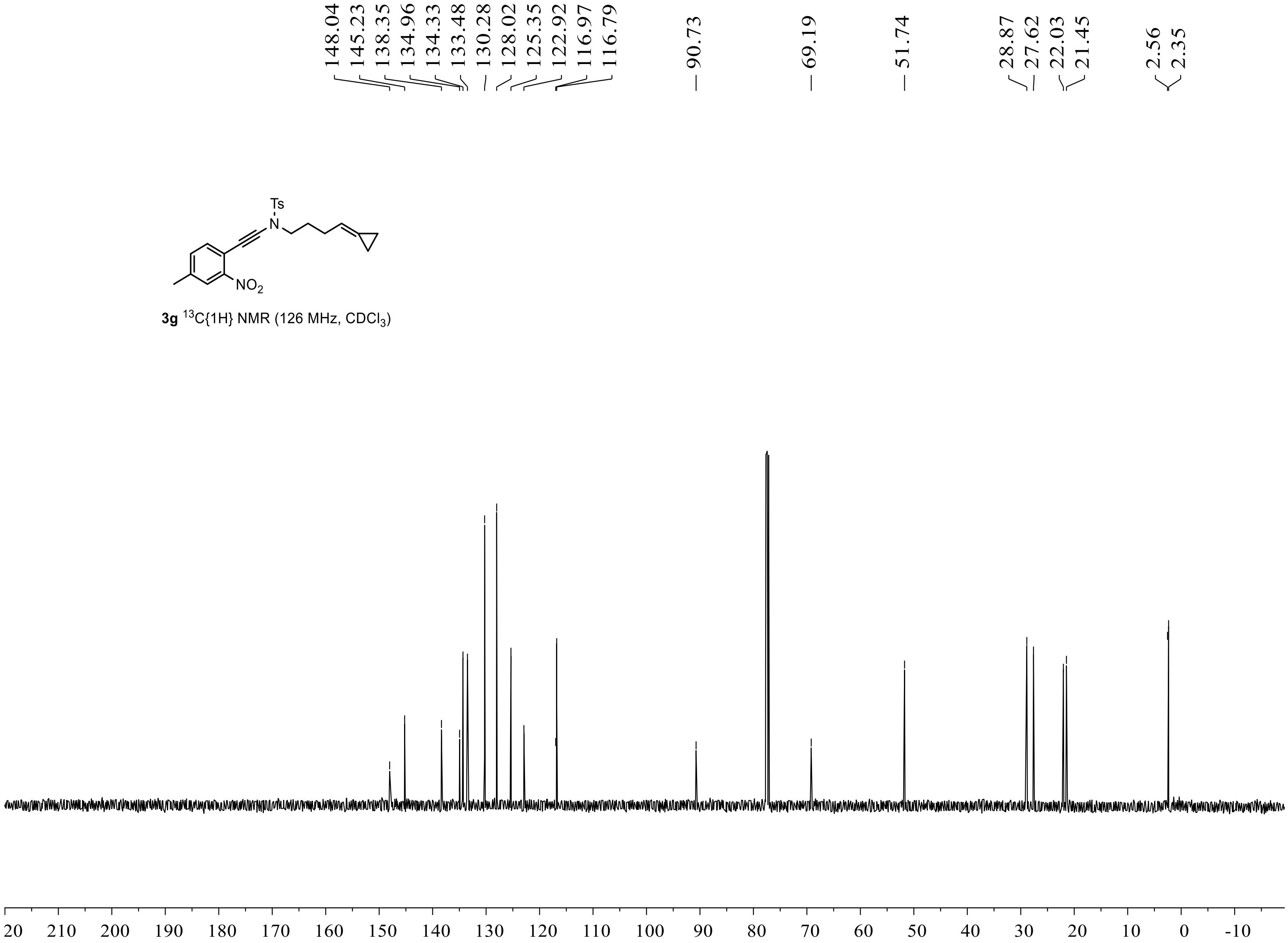

Supplementary Figure 127. ^{13}C NMR ($CDCl_3$, 126 MHz, 298 K) spectrum for **3g**

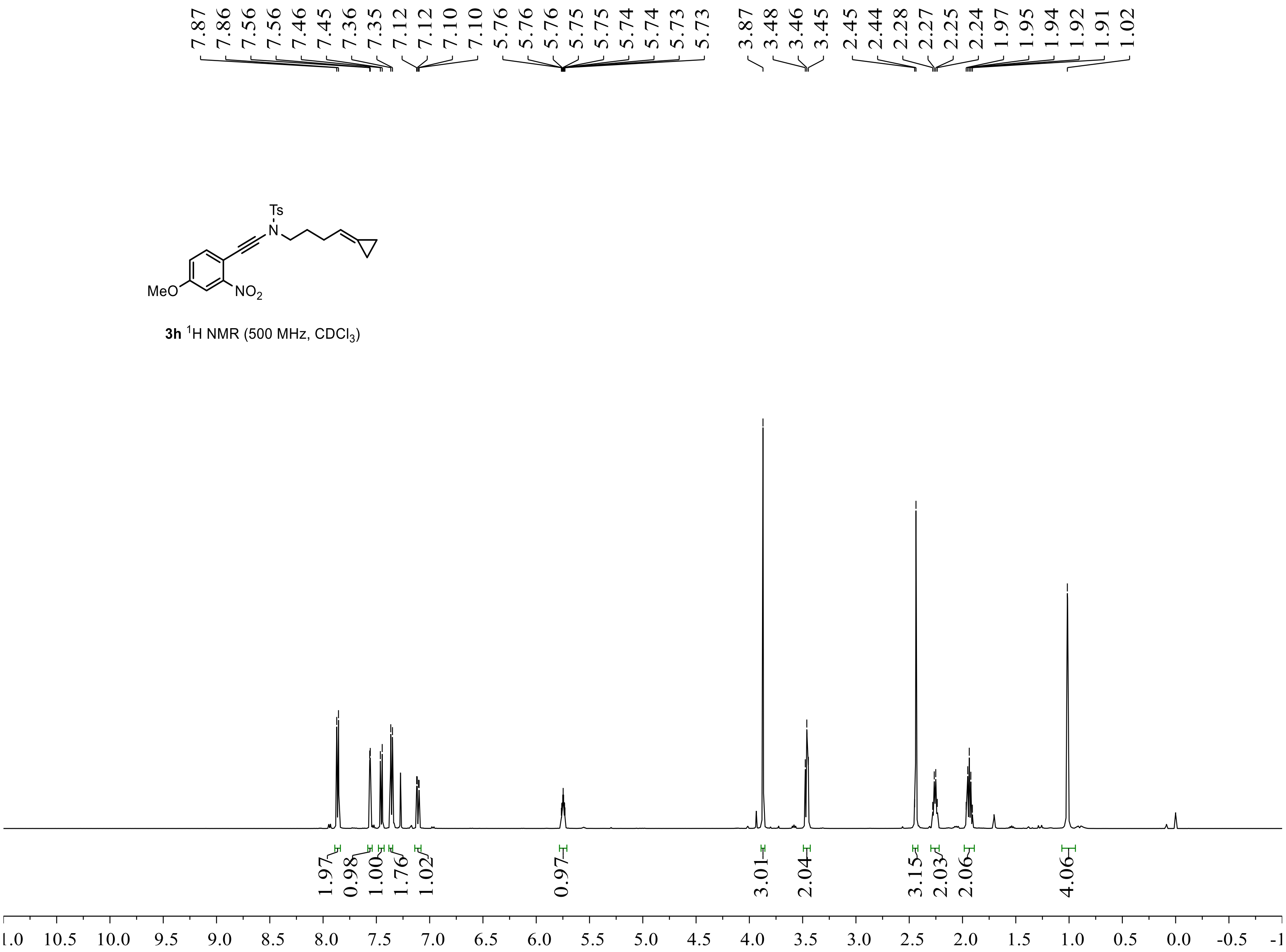

Supplementary Figure 128. ^{1}H NMR ($CDCl_3$, 500 MHz, 298 K) spectrum for **3h**

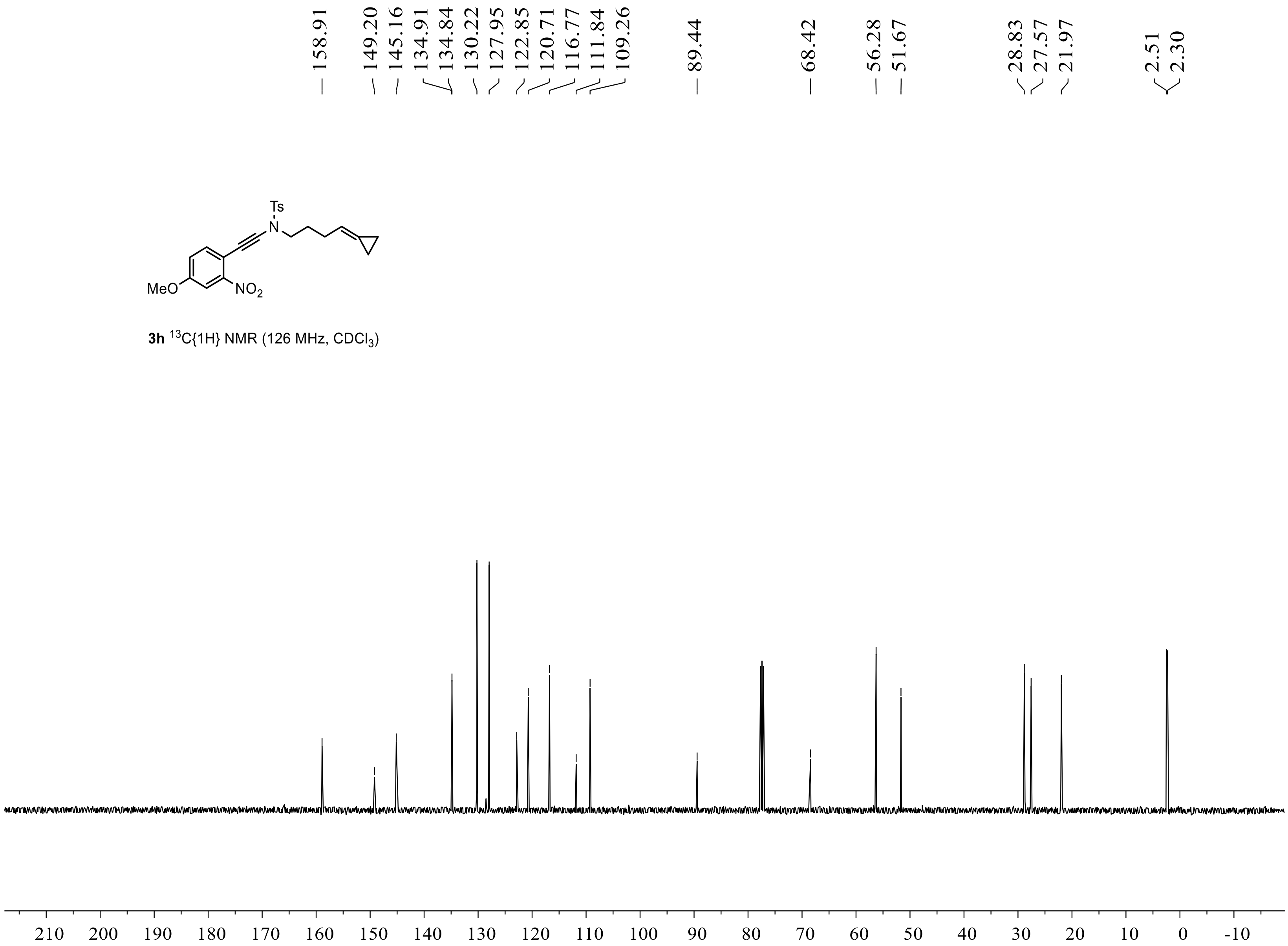

Supplementary Figure 129. ^{13}C NMR ($CDCl_3$, 126 MHz, 298 K) spectrum for **3h**

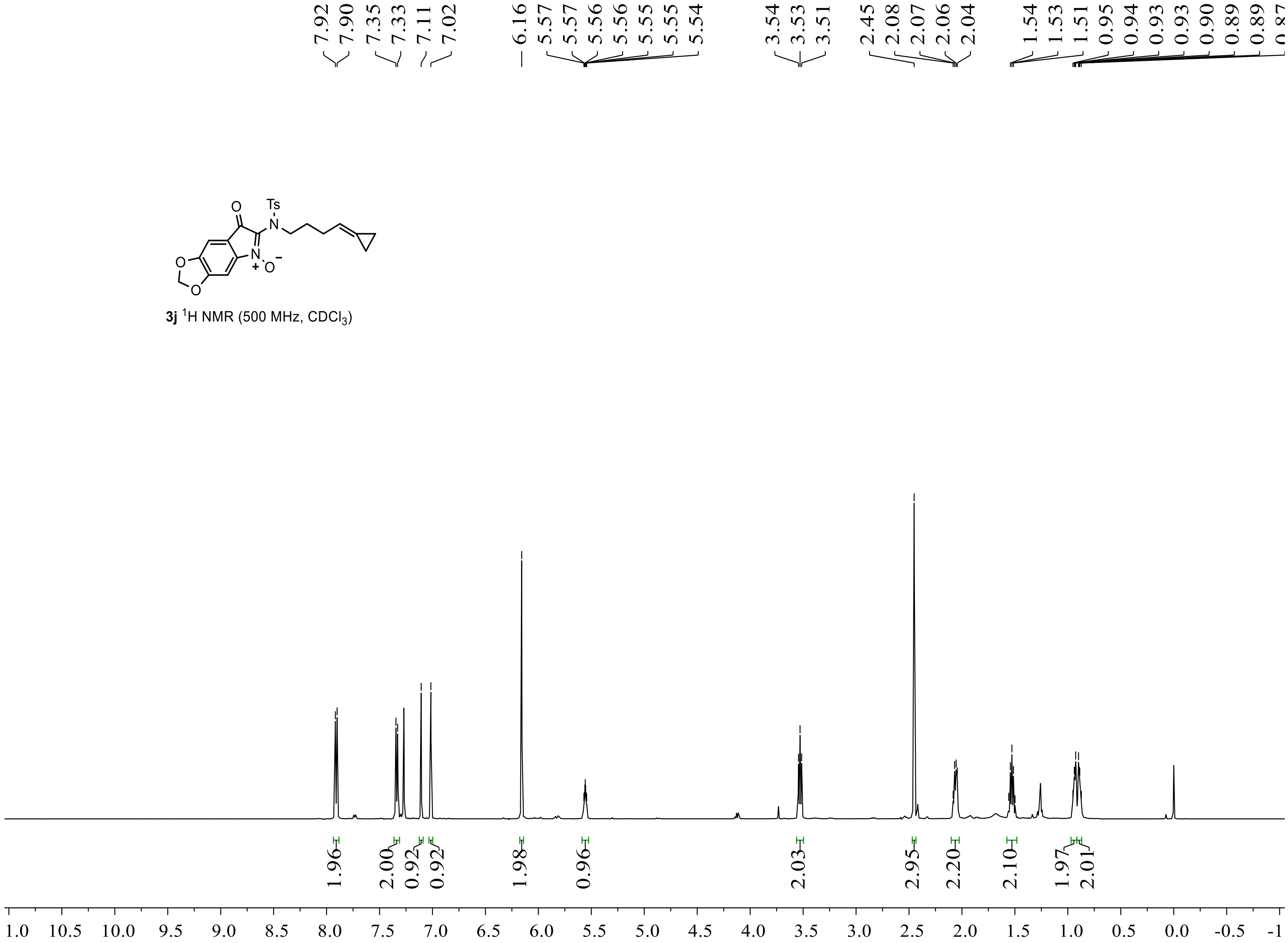

Supplementary Figure 130. ^{1}H NMR ($CDCl_3$, 500 MHz, 298 K) spectrum for **3j**

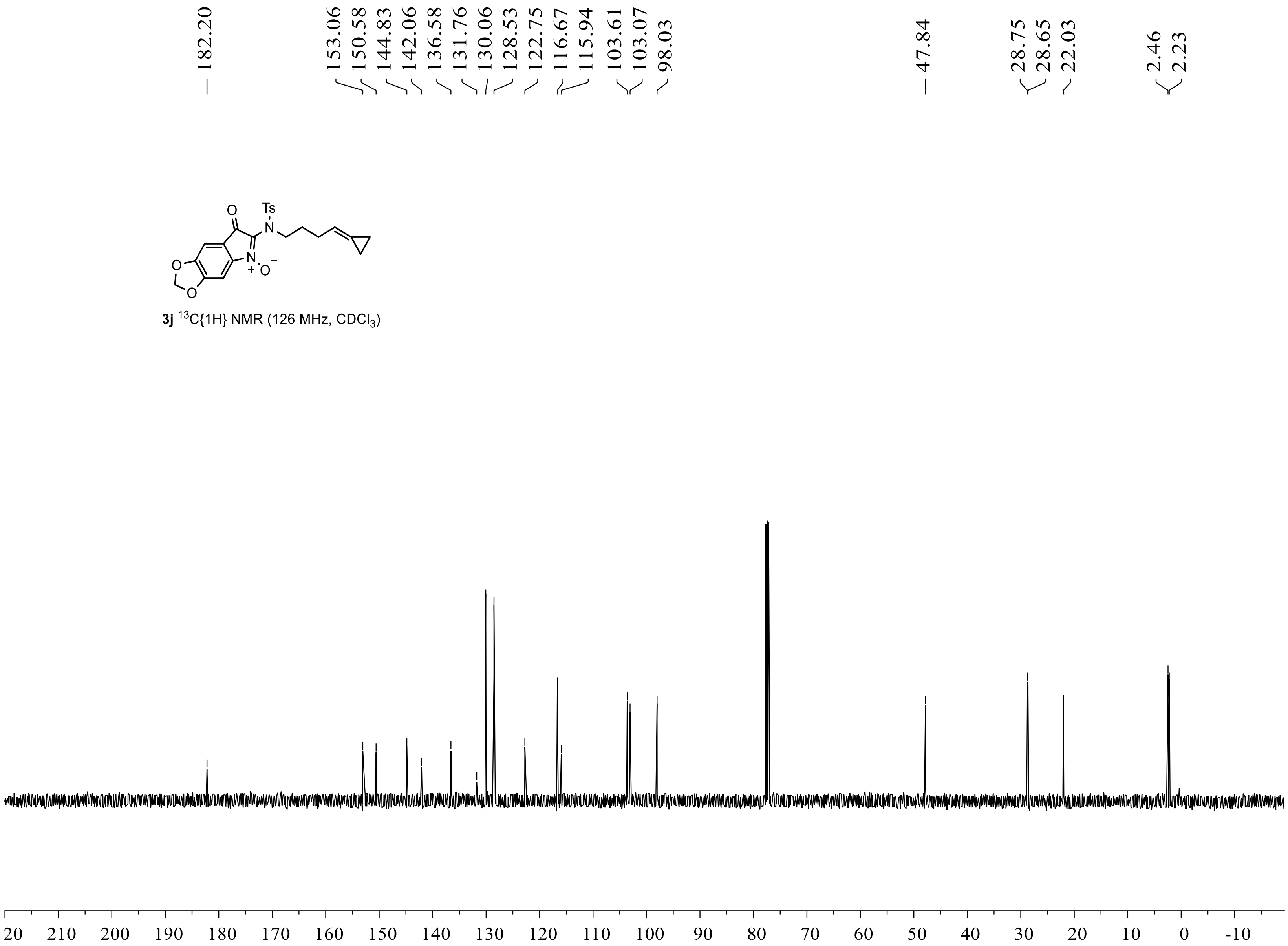

Supplementary Figure 131. ^{13}C NMR ($CDCl_3$, 126 MHz, 298 K) spectrum for **3j**

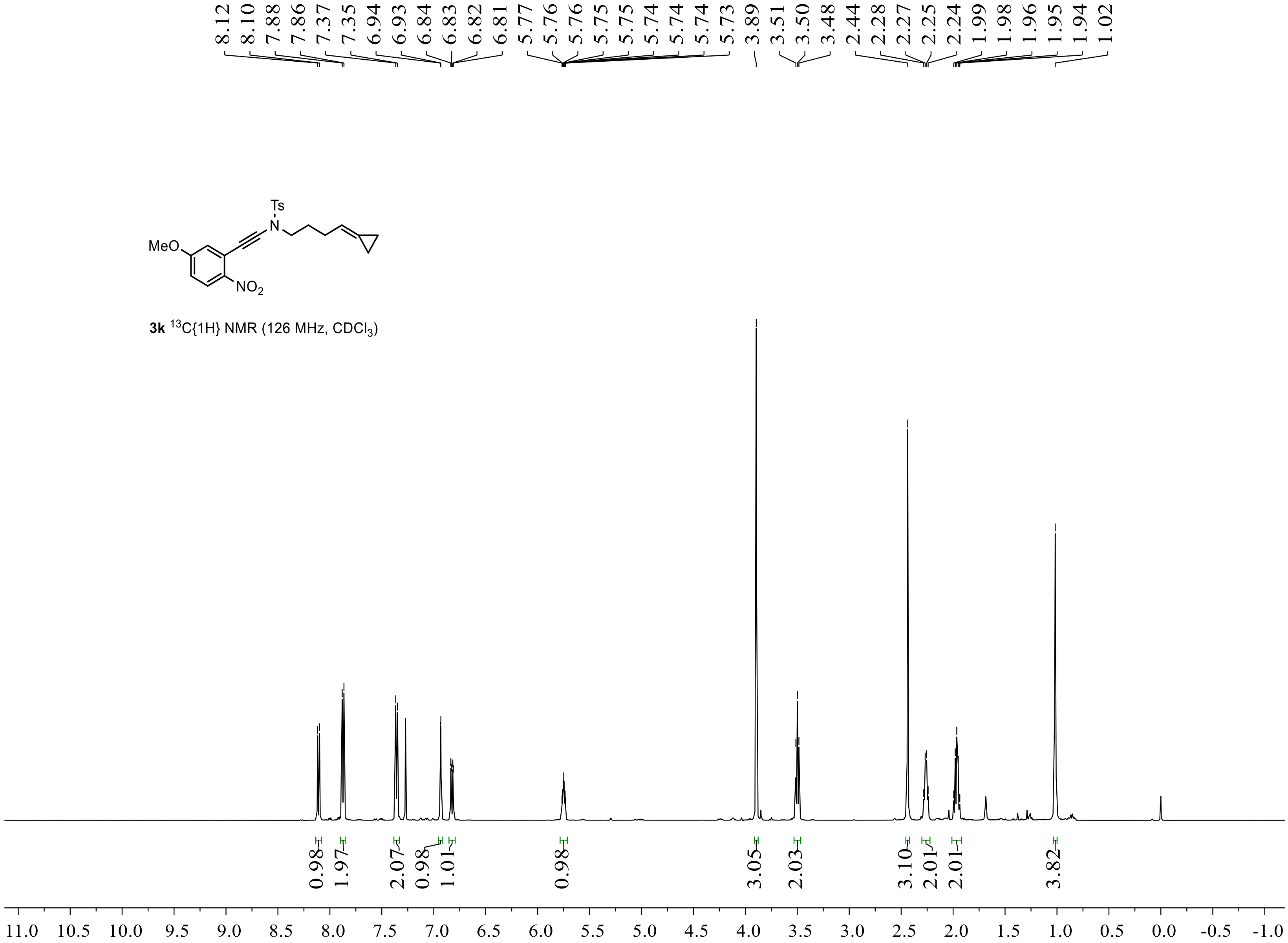

Supplementary Figure 132. ^{1}H NMR ($CDCl_3$, 500 MHz, 298 K) spectrum for **3k**

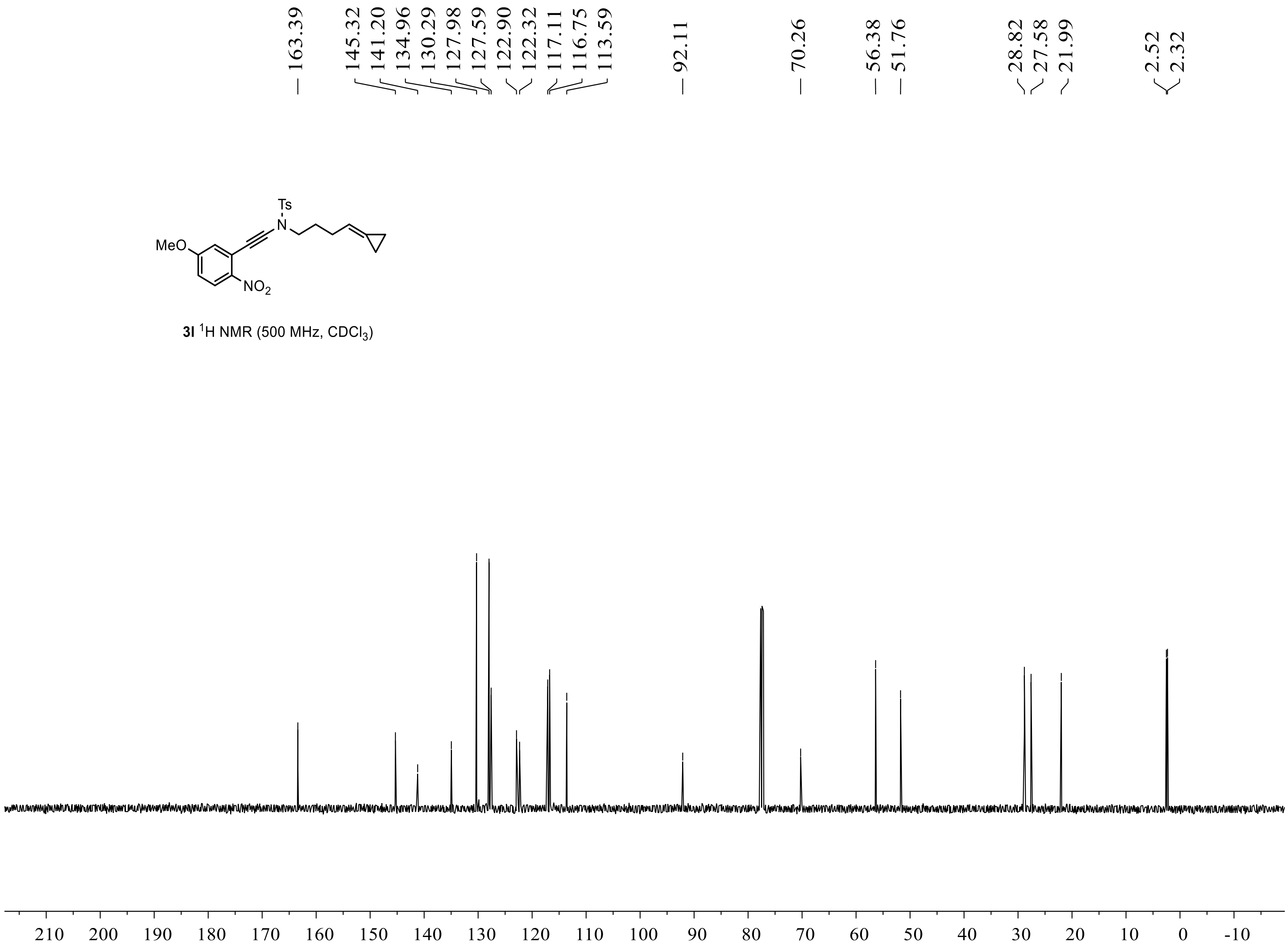

Supplementary Figure 133. ^{13}C NMR ($CDCl_3$, 126 MHz, 298 K) spectrum for **3k**

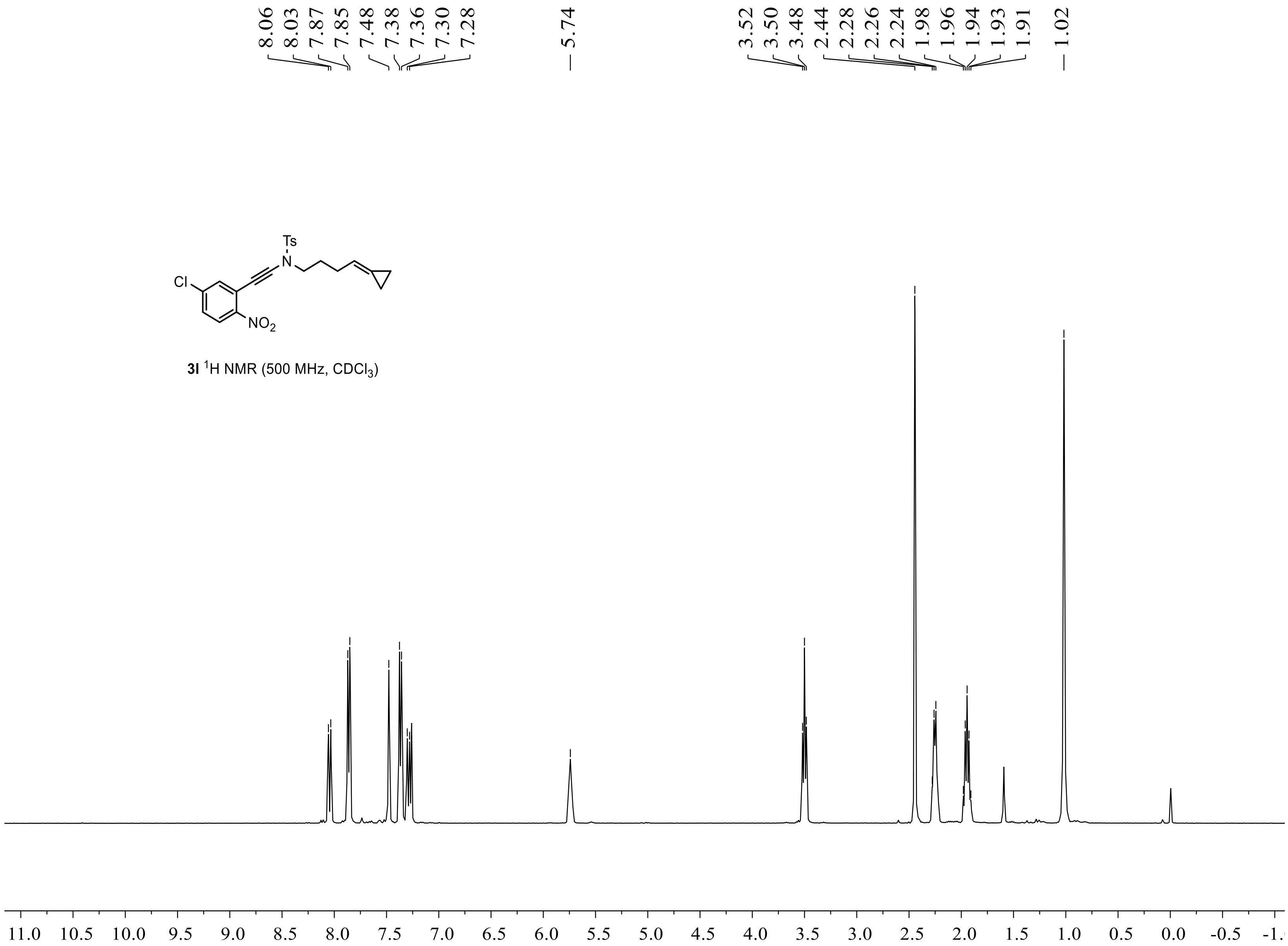

Supplementary Figure 134. ^{1}H NMR ($CDCl_3$, 500 MHz, 298 K) spectrum for **3l**

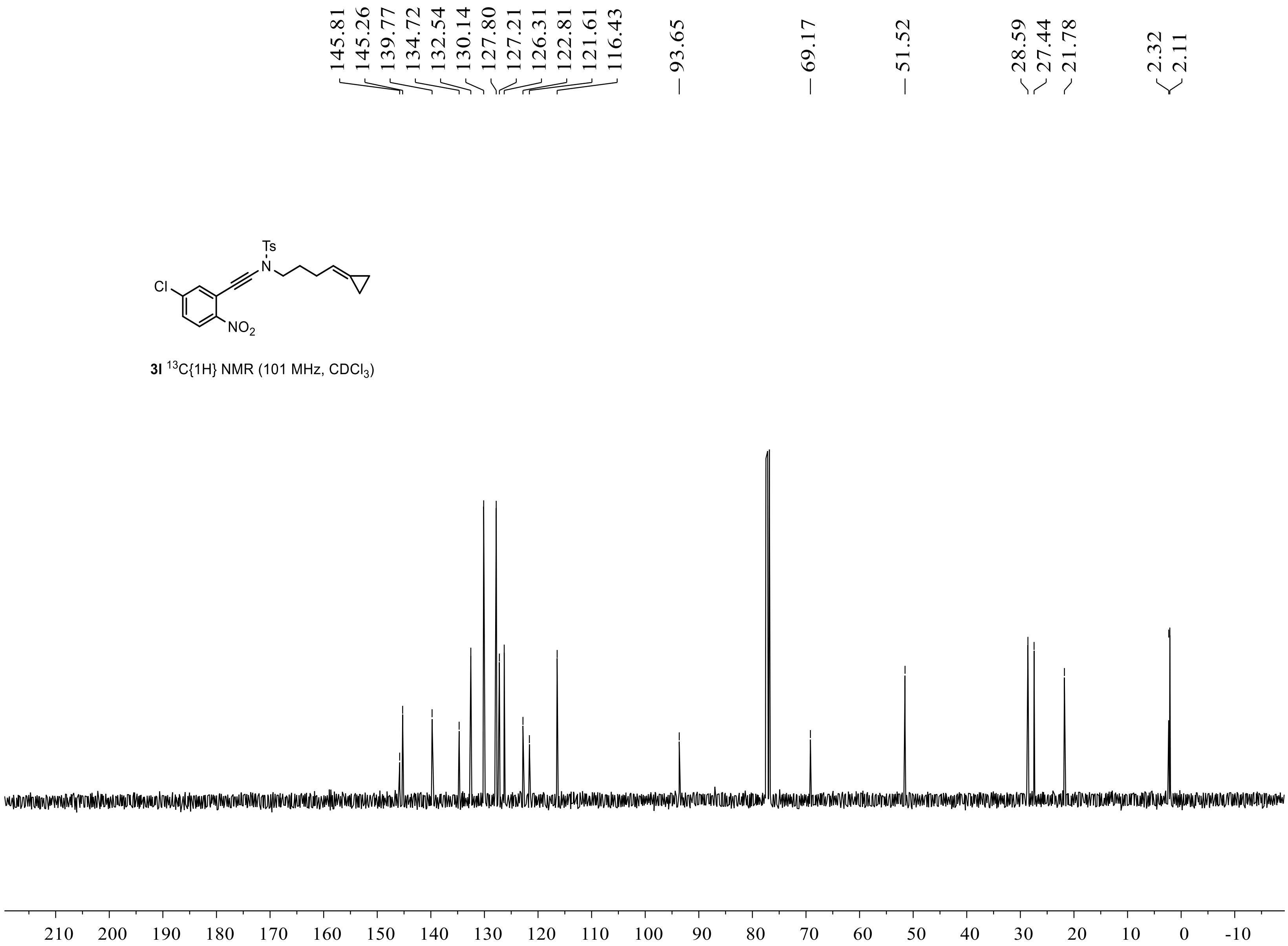

Supplementary Figure 135. ^{13}C NMR ($CDCl_3$, 126 MHz, 298 K) spectrum for **3l**

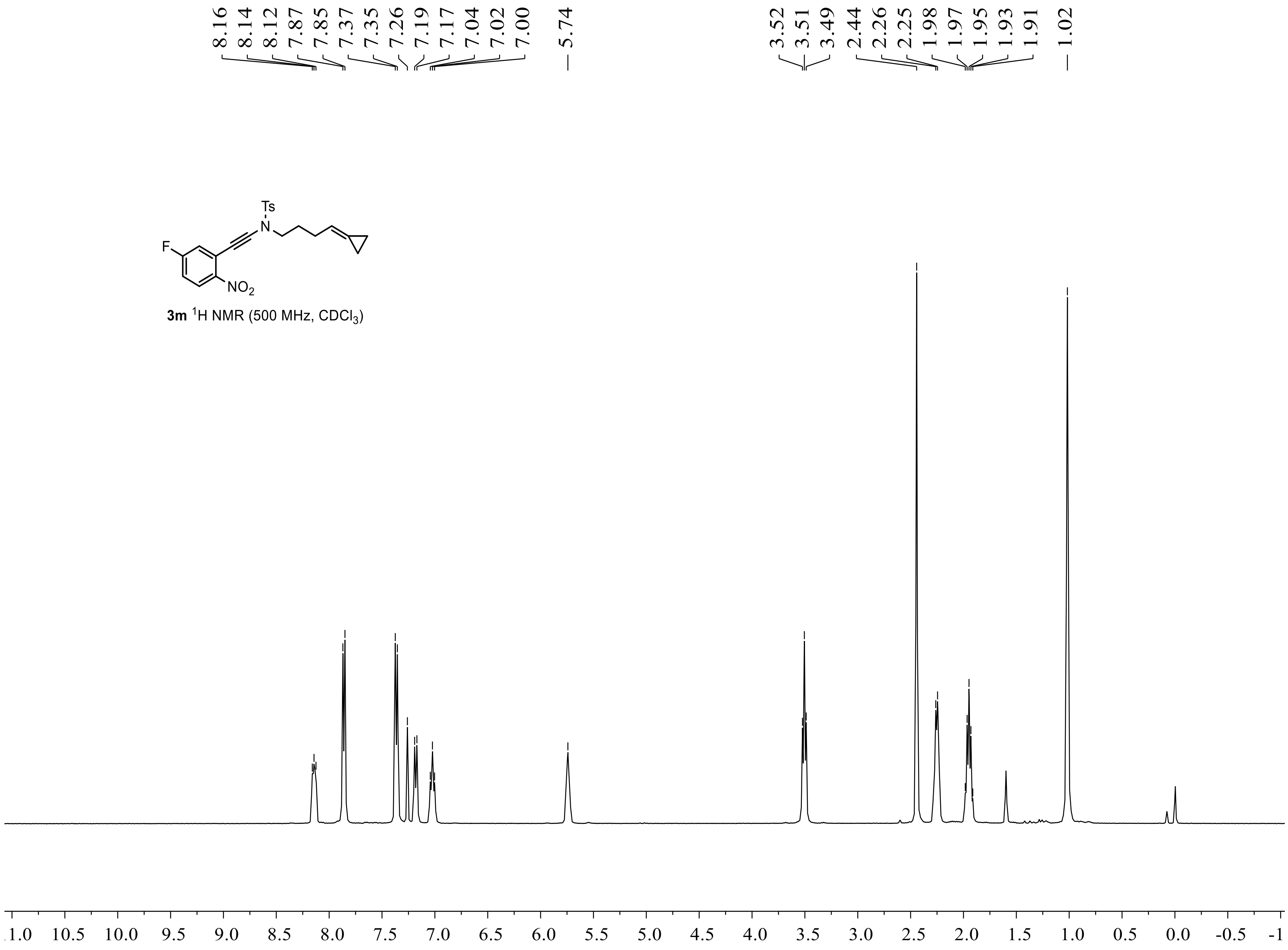

Supplementary Figure 136. ^{1}H NMR ($CDCl_3$, 500 MHz, 298 K) spectrum for **3m**

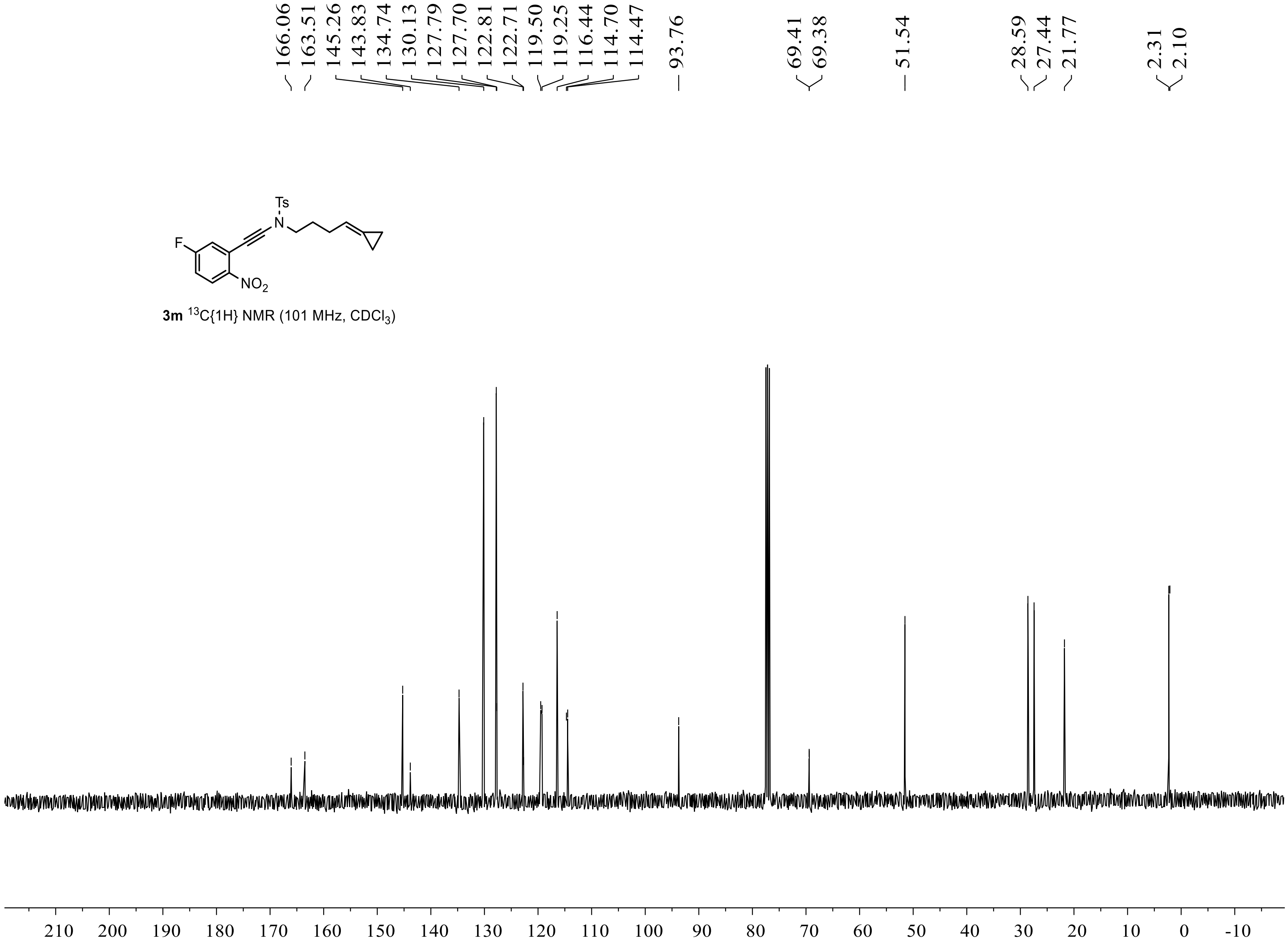

Supplementary Figure 137. ^{13}C NMR ($CDCl_3$, 126 MHz, 298 K) spectrum for **3m**

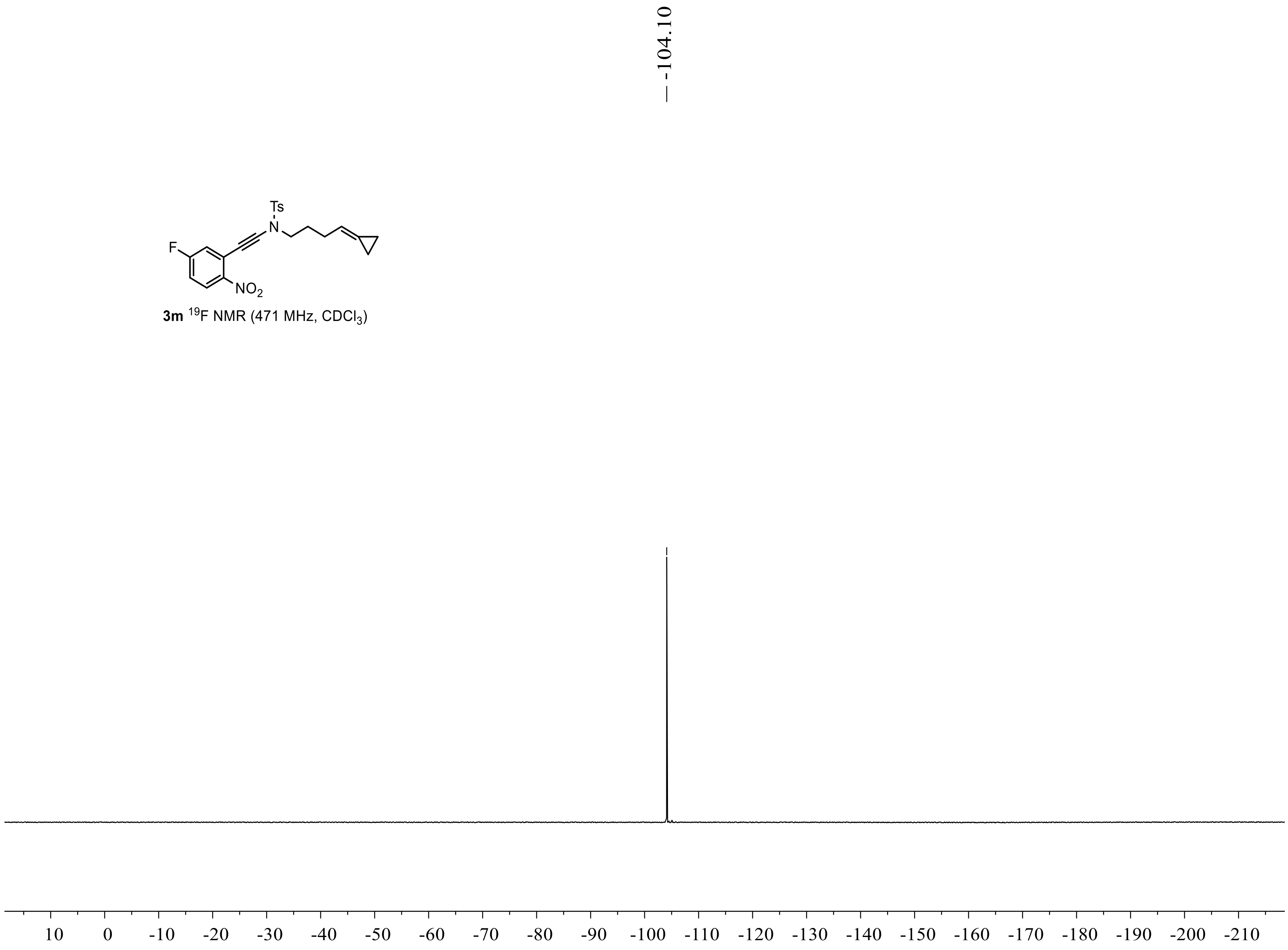

Supplementary Figure 138. ^{19}F NMR ($CDCl_3$, 471 MHz, 298 K) spectrum for **3m**

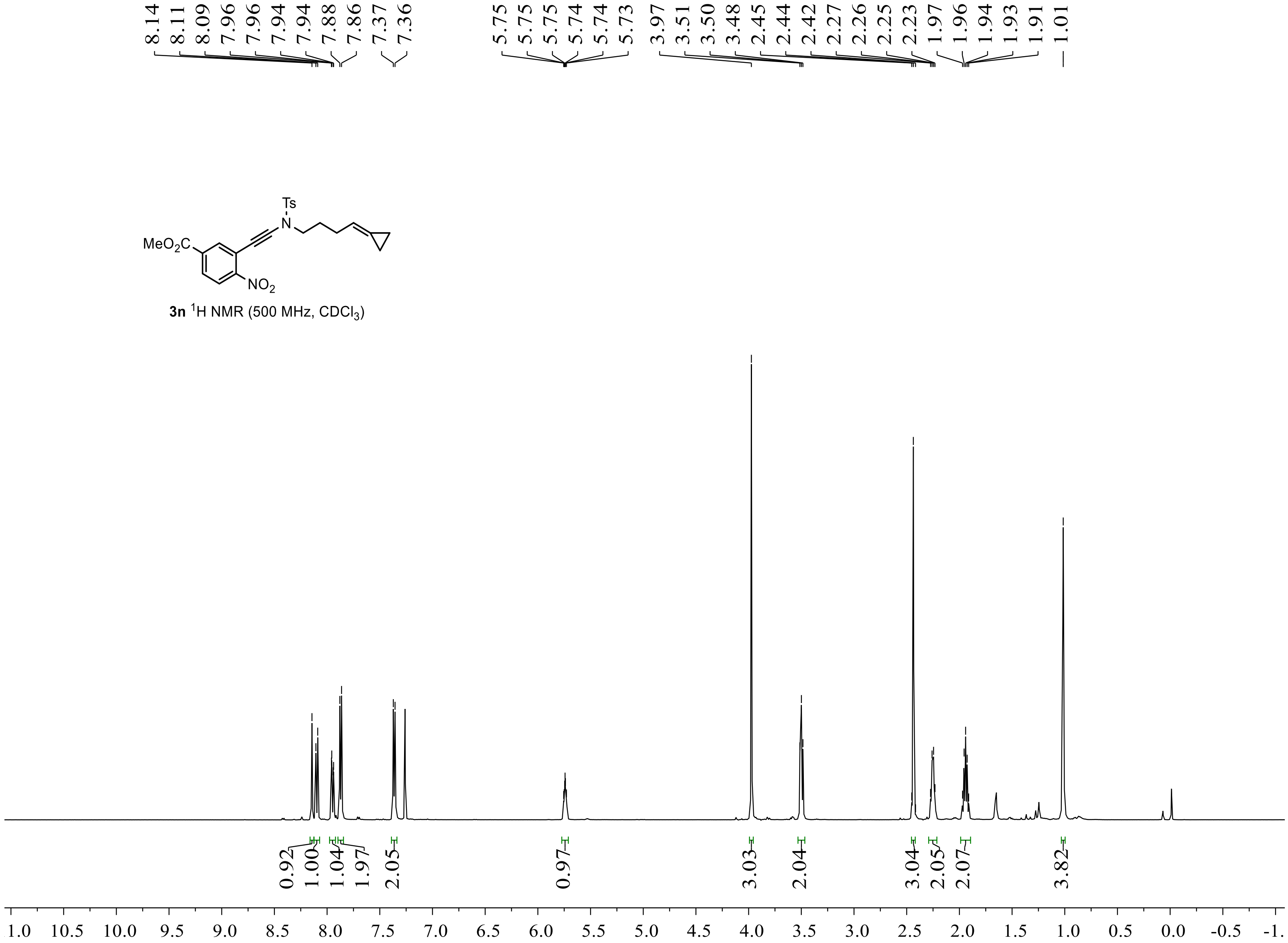

Supplementary Figure 139. ^{1}H NMR ($CDCl_3$, 500 MHz, 298 K) spectrum for **3n**

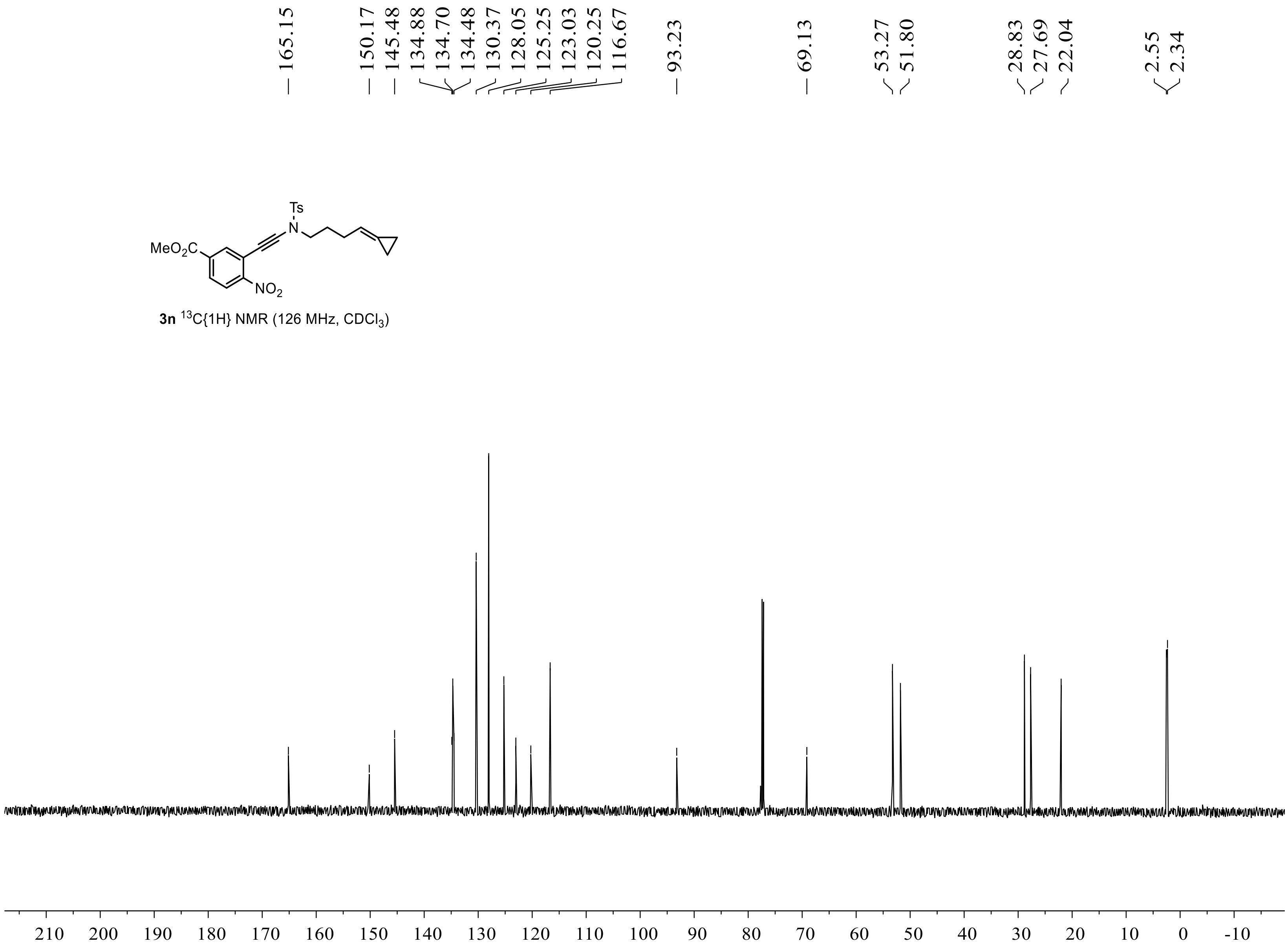

Supplementary Figure 140. ^{13}C NMR ($CDCl_3$, 126 MHz, 298 K) spectrum for **3n**

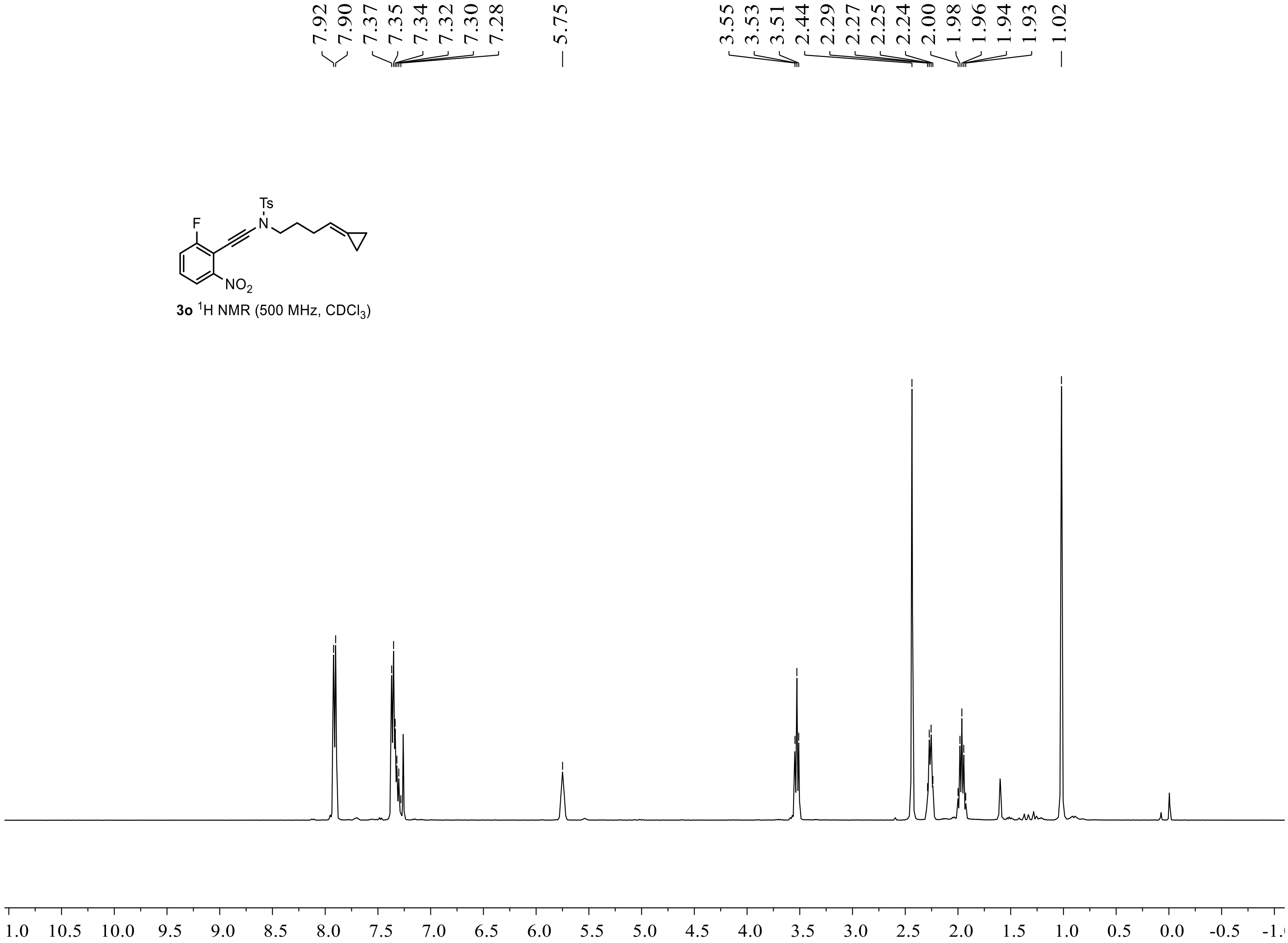

Supplementary Figure 141. ^{1}H NMR ($CDCl_3$, 500 MHz, 298 K) spectrum for **3o**

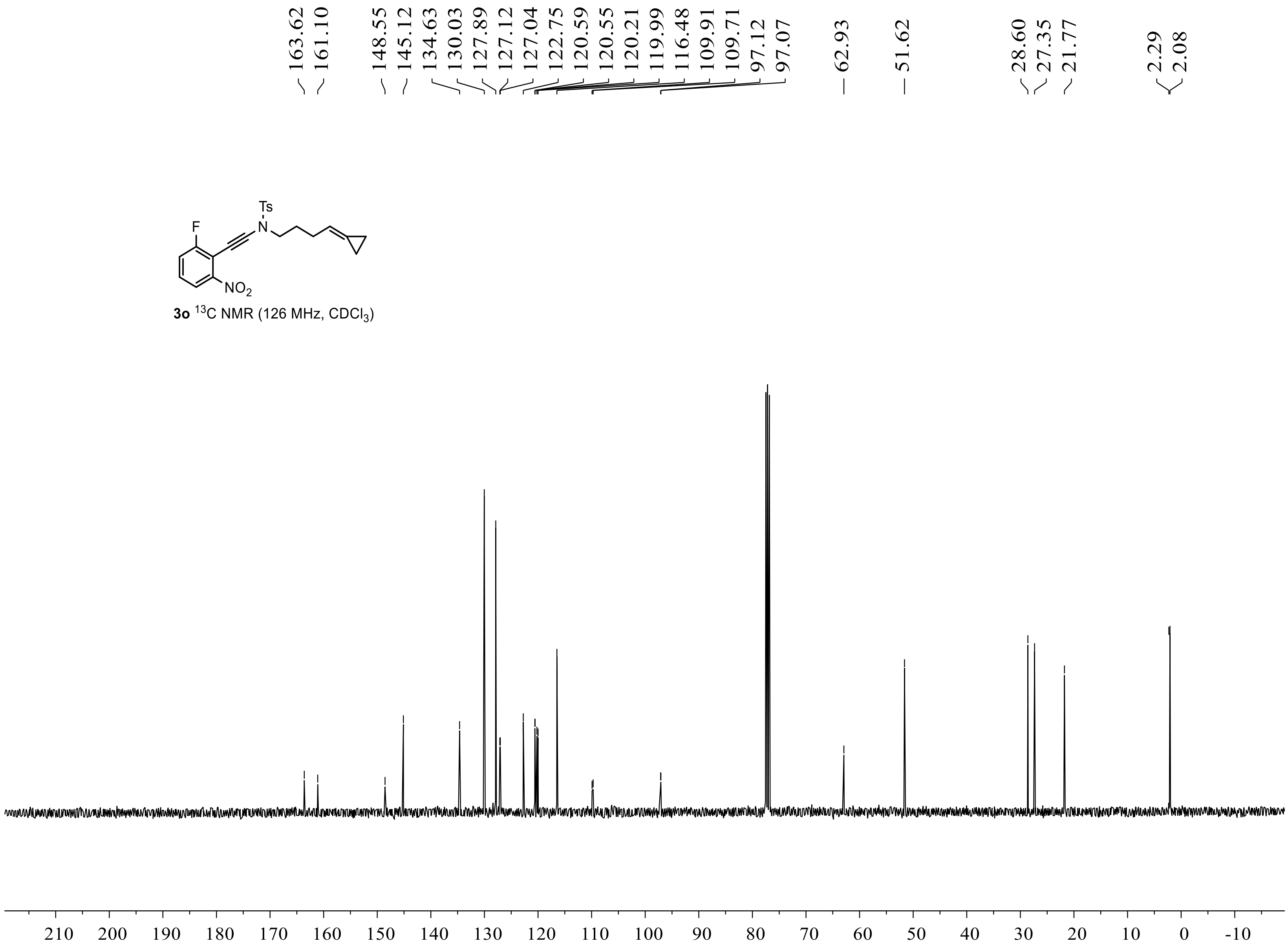

Supplementary Figure 142. ^{13}C NMR ($CDCl_3$, 126 MHz, 298 K) spectrum for **3o**

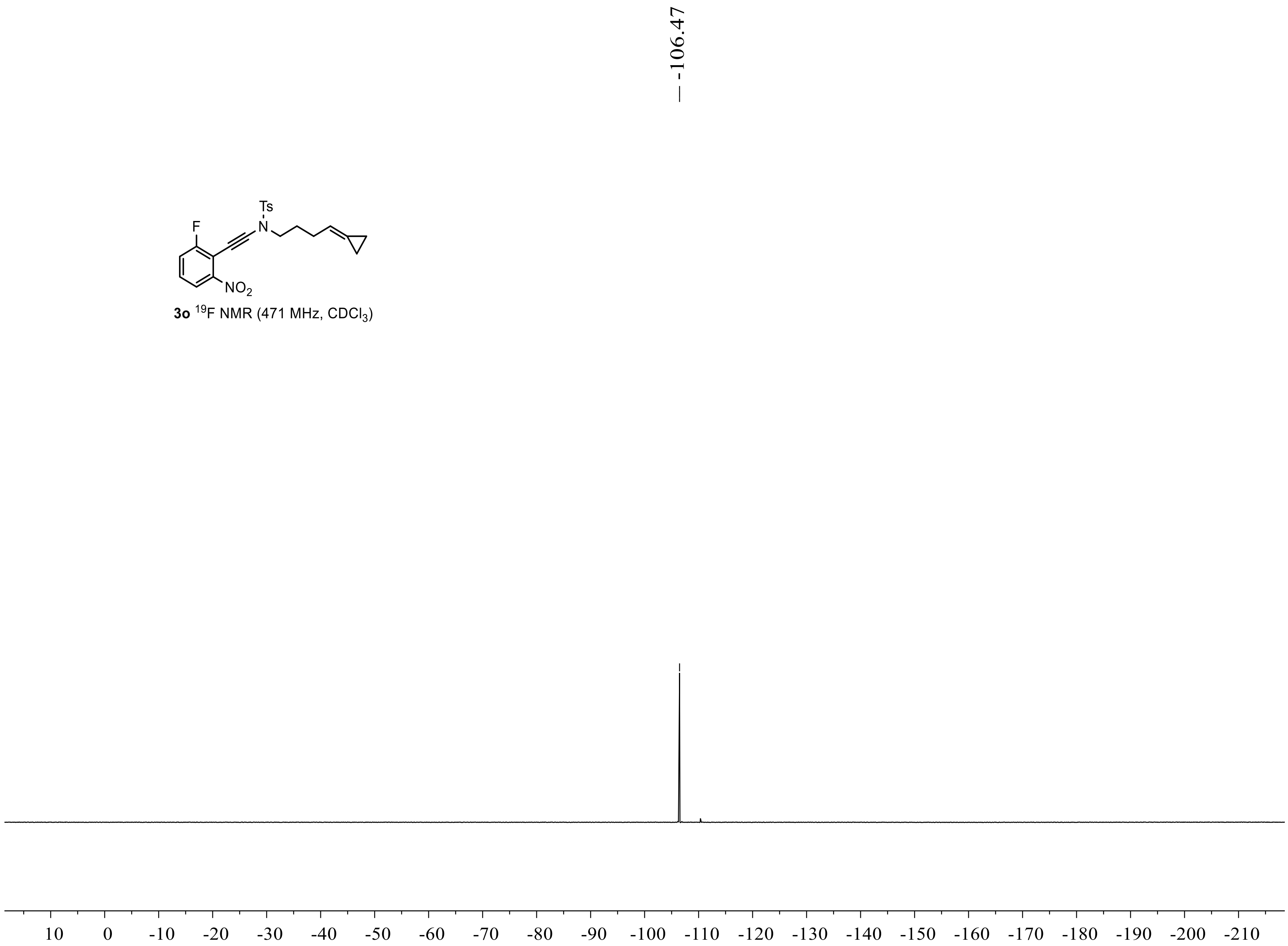

Supplementary Figure 143. ^{19}F NMR ($CDCl_3$, 471 MHz, 298 K) spectrum for **3o**

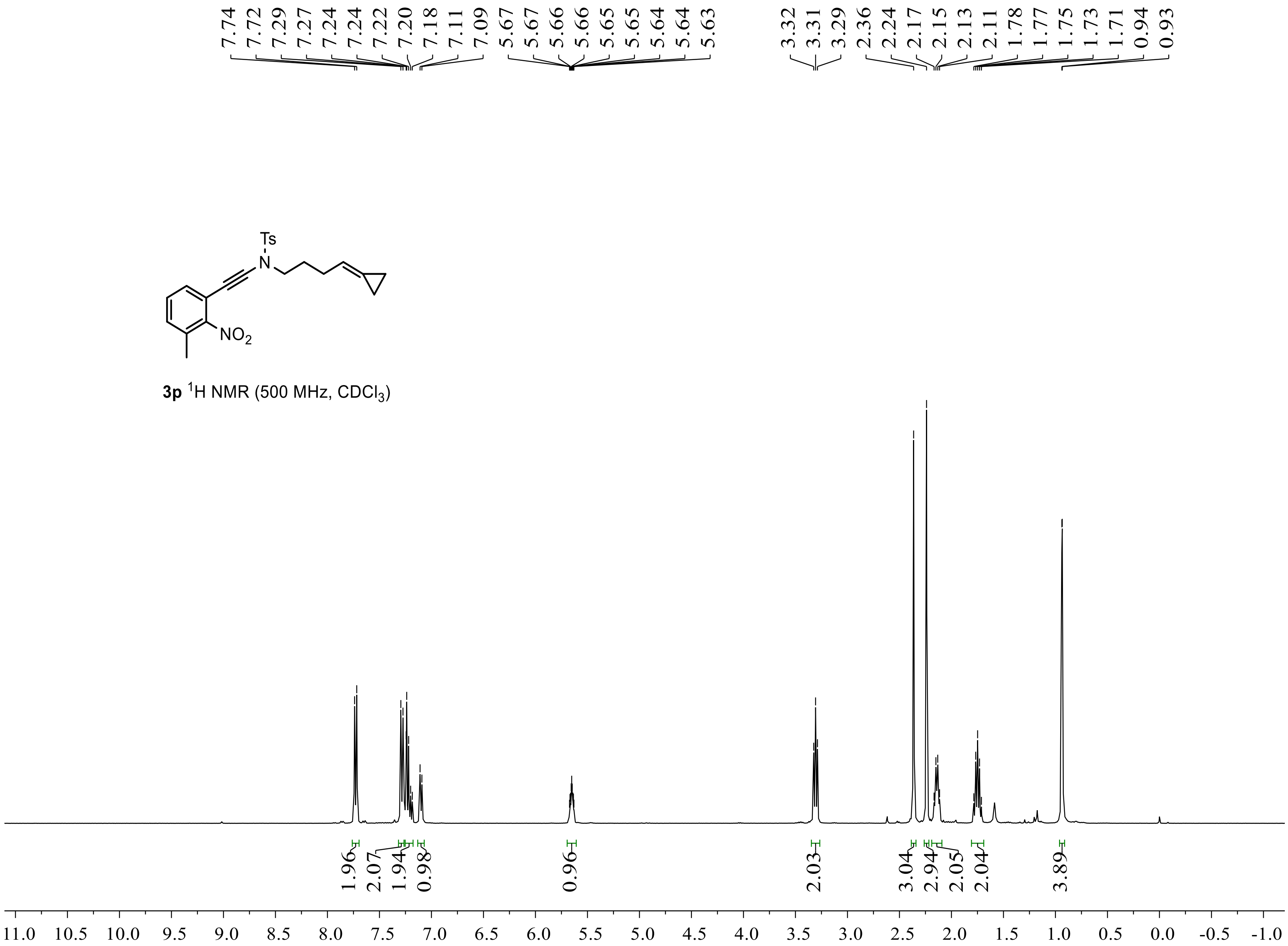

Supplementary Figure 144. ^{1}H NMR ($CDCl_3$, 500 MHz, 298 K) spectrum for **3p**

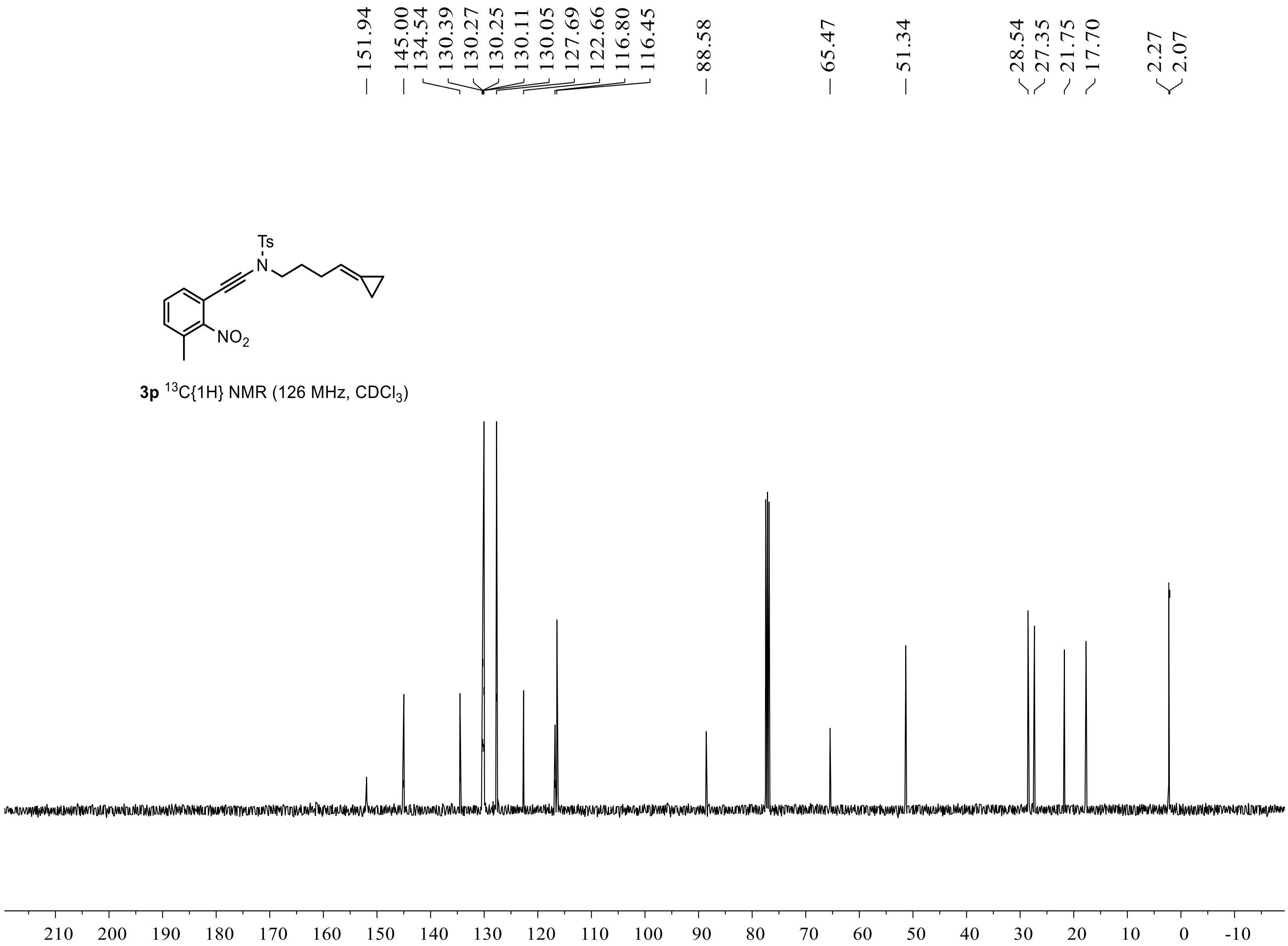

Supplementary Figure 145. ^{13}C NMR ($CDCl_3$, 126 MHz, 298 K) spectrum for **3p**

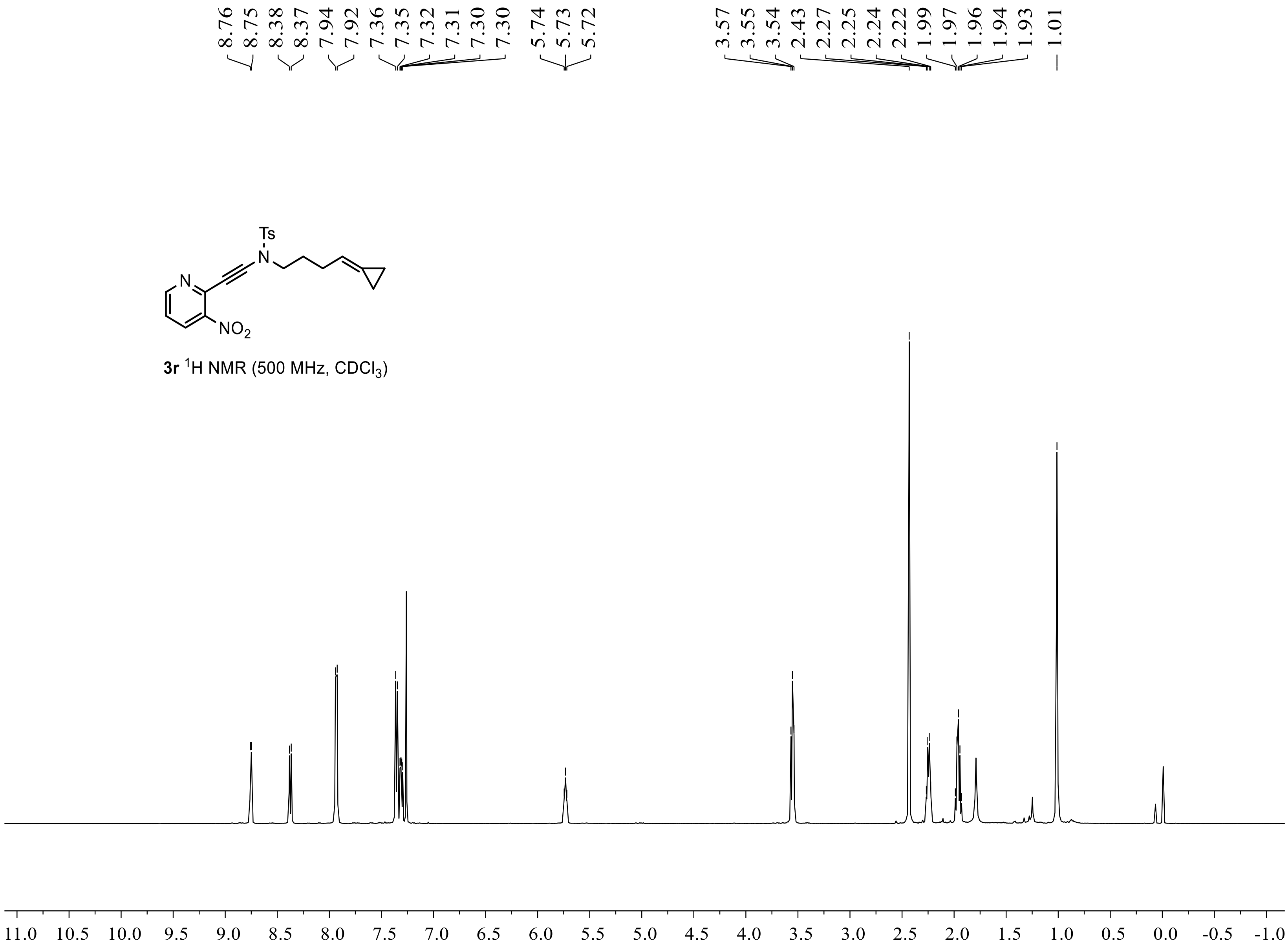

Supplementary Figure 146. ^{1}H NMR ($CDCl_3$, 500 MHz, 298 K) spectrum for **3r**

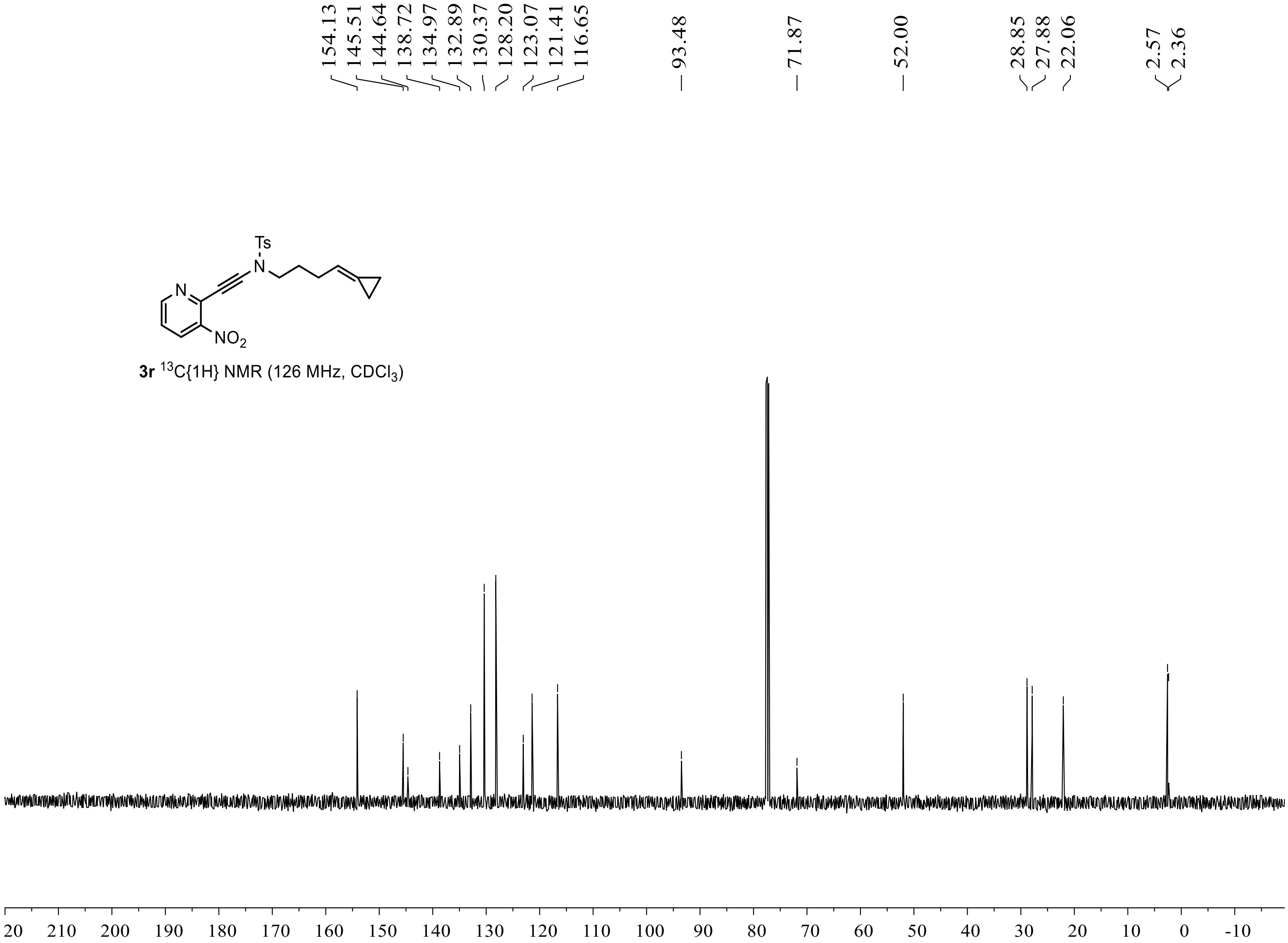

Supplementary Figure 147. ^{13}C NMR ($CDCl_3$, 126 MHz, 298 K) spectrum for **3r**

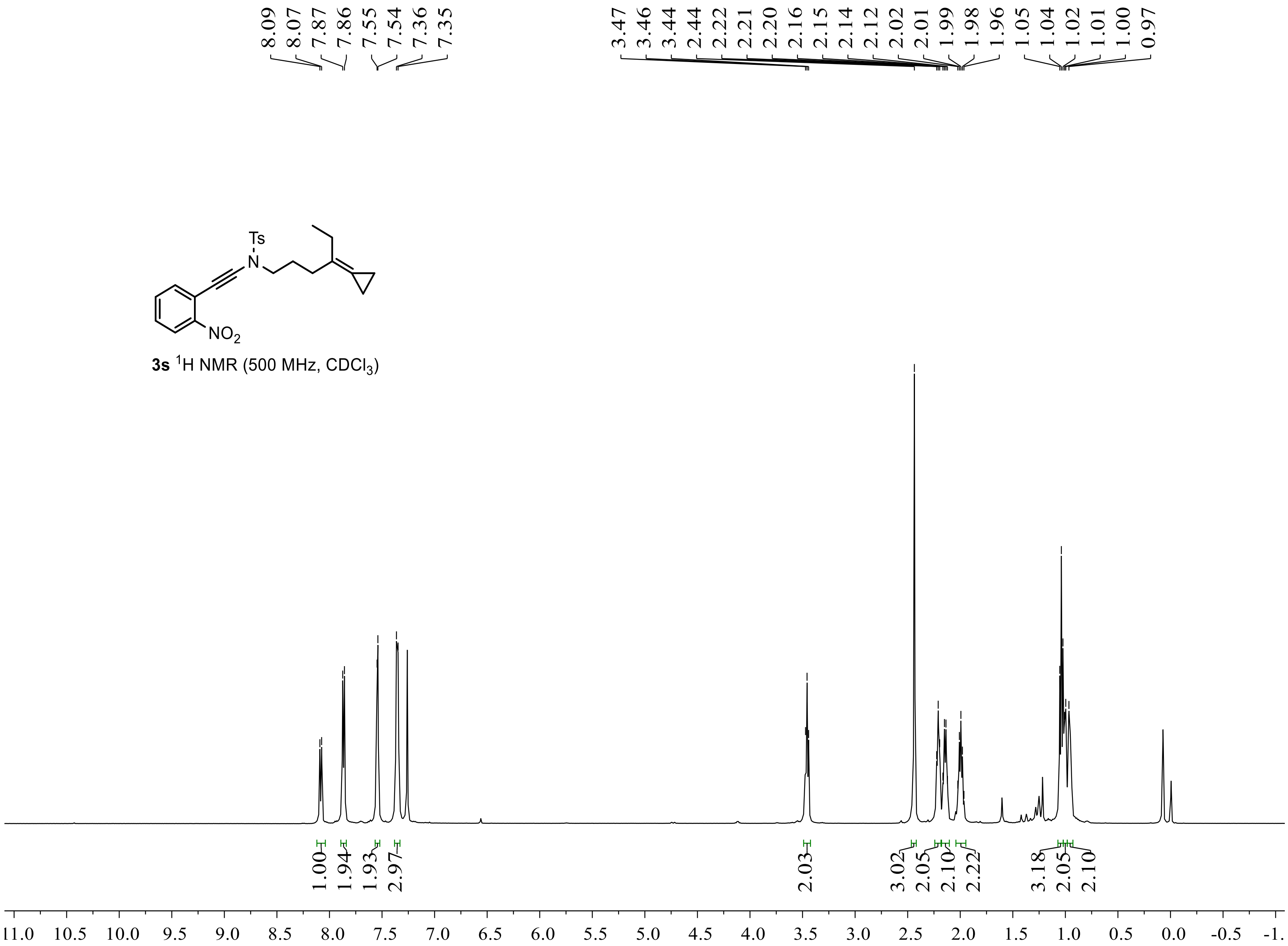

Supplementary Figure 148. ^{1}H NMR ($CDCl_3$, 500 MHz, 298 K) spectrum for **3s**

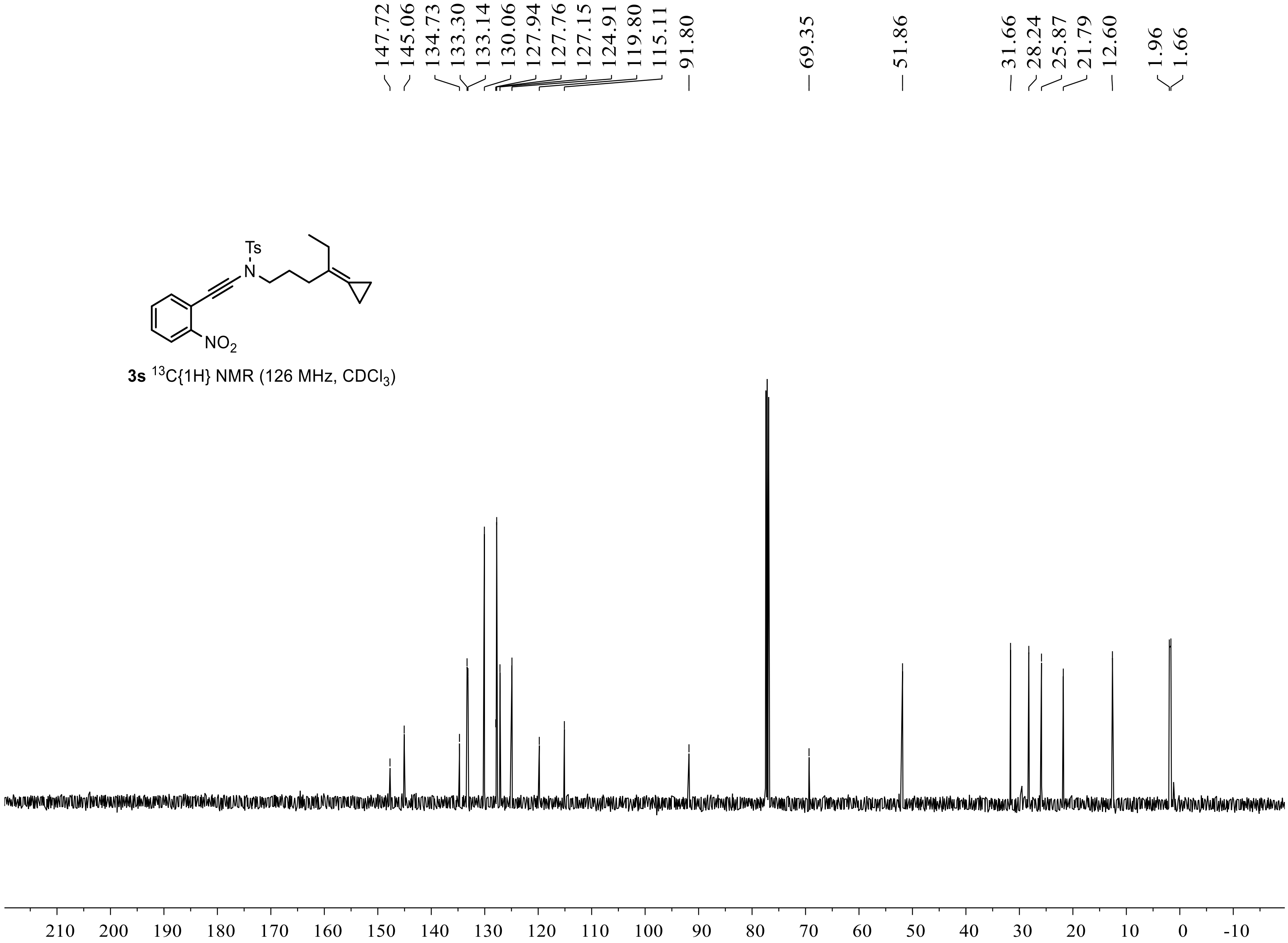

Supplementary Figure 149. ^{13}C NMR ($CDCl_3$, 126 MHz, 298 K) spectrum for **3s**

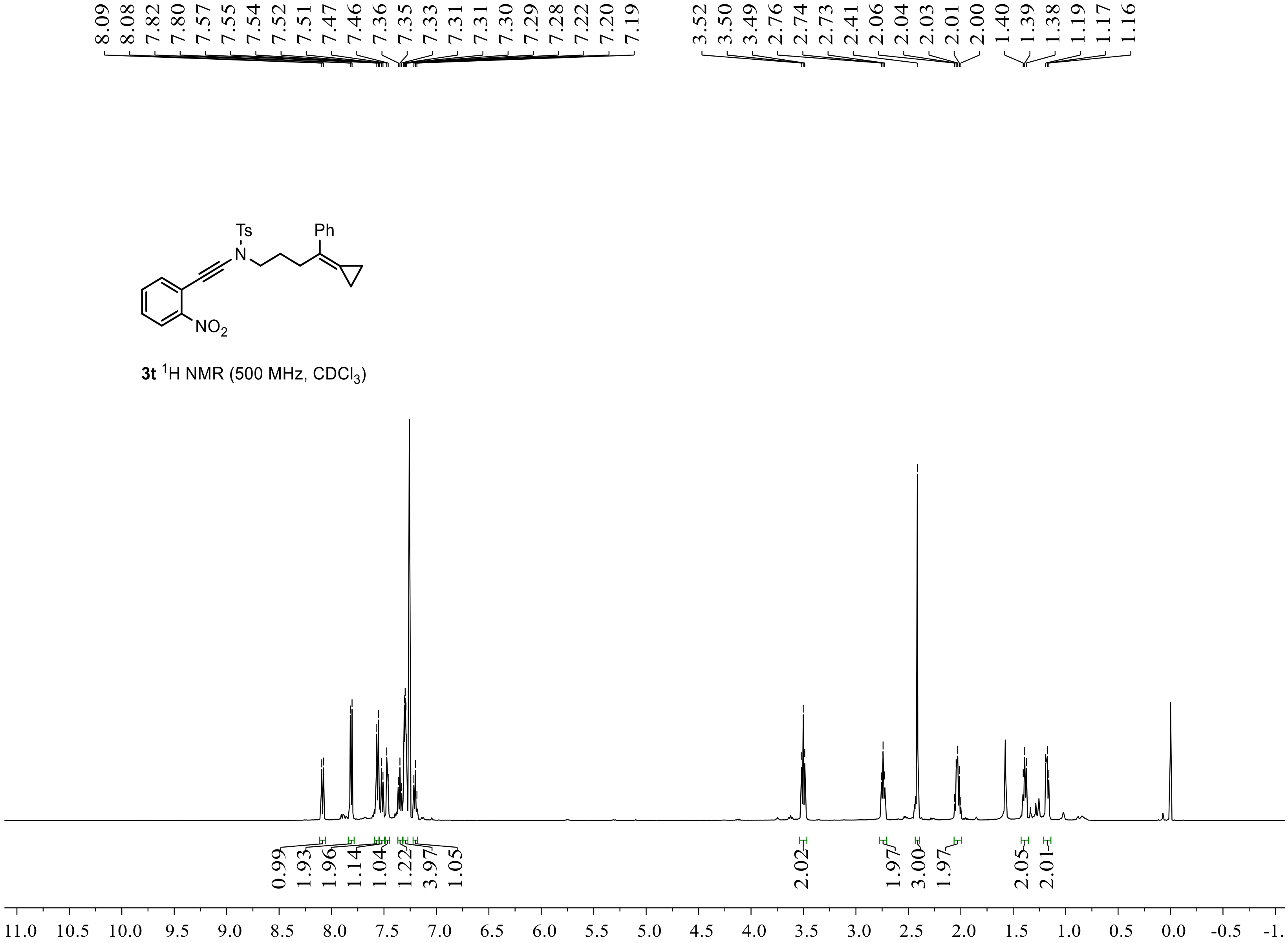

Supplementary Figure 150. ^{1}H NMR ($CDCl_3$, 500 MHz, 298 K) spectrum for **3t**

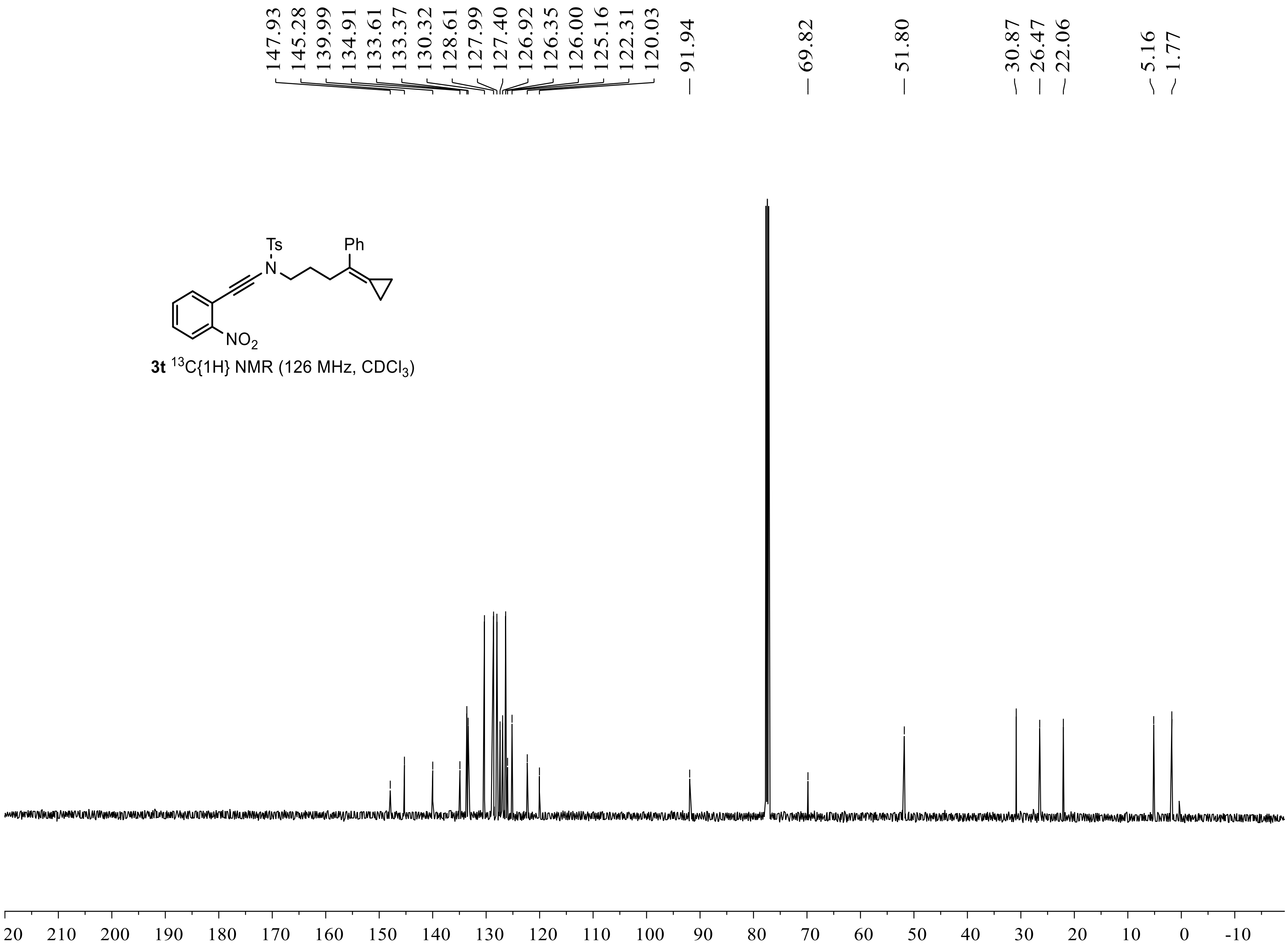

Supplementary Figure 151. ^{13}C NMR ($CDCl_3$, 126 MHz, 298 K) spectrum for **3t**

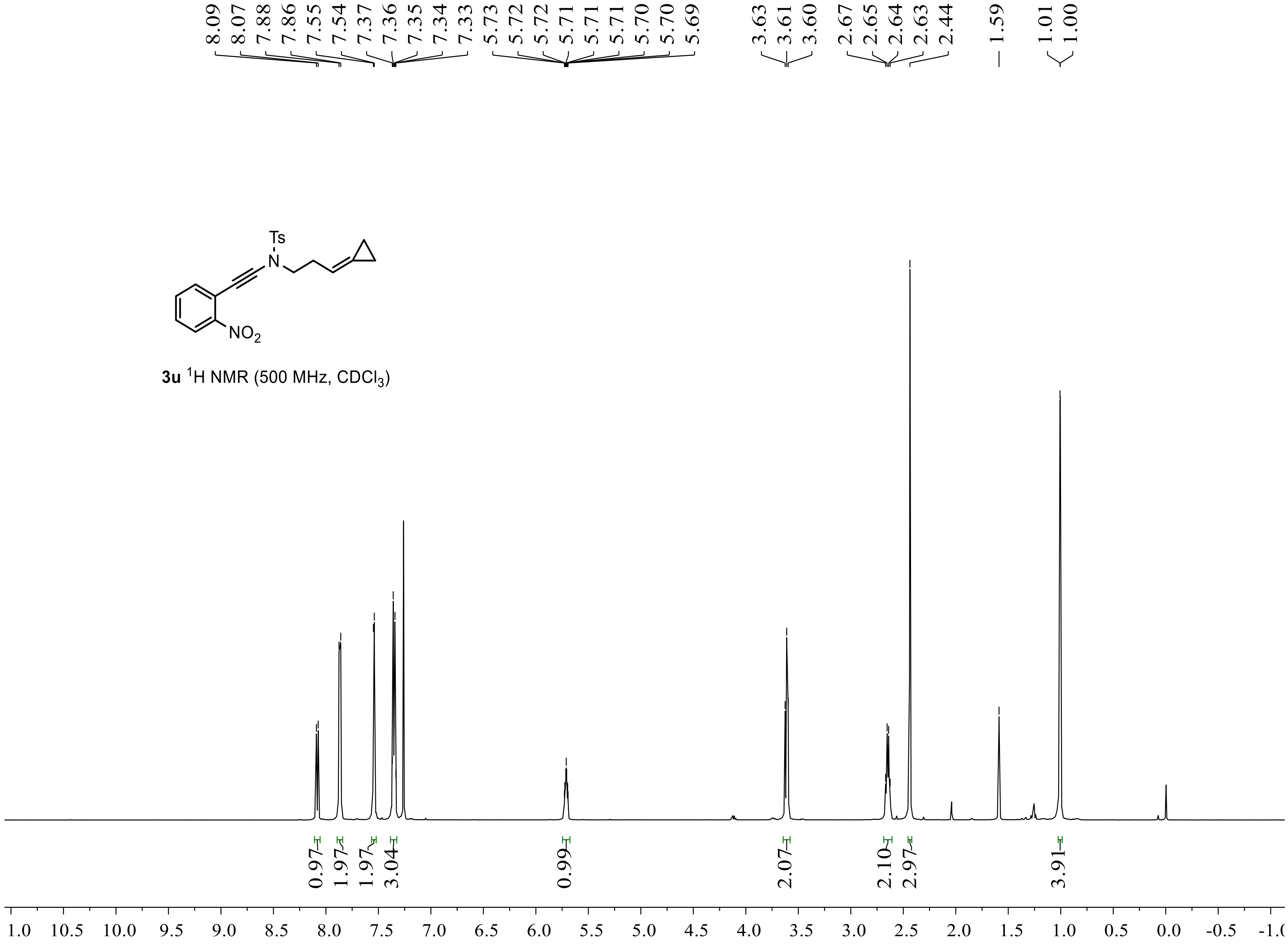

Supplementary Figure 152. ^{1}H NMR ($CDCl_3$, 500 MHz, 298 K) spectrum for **3u**

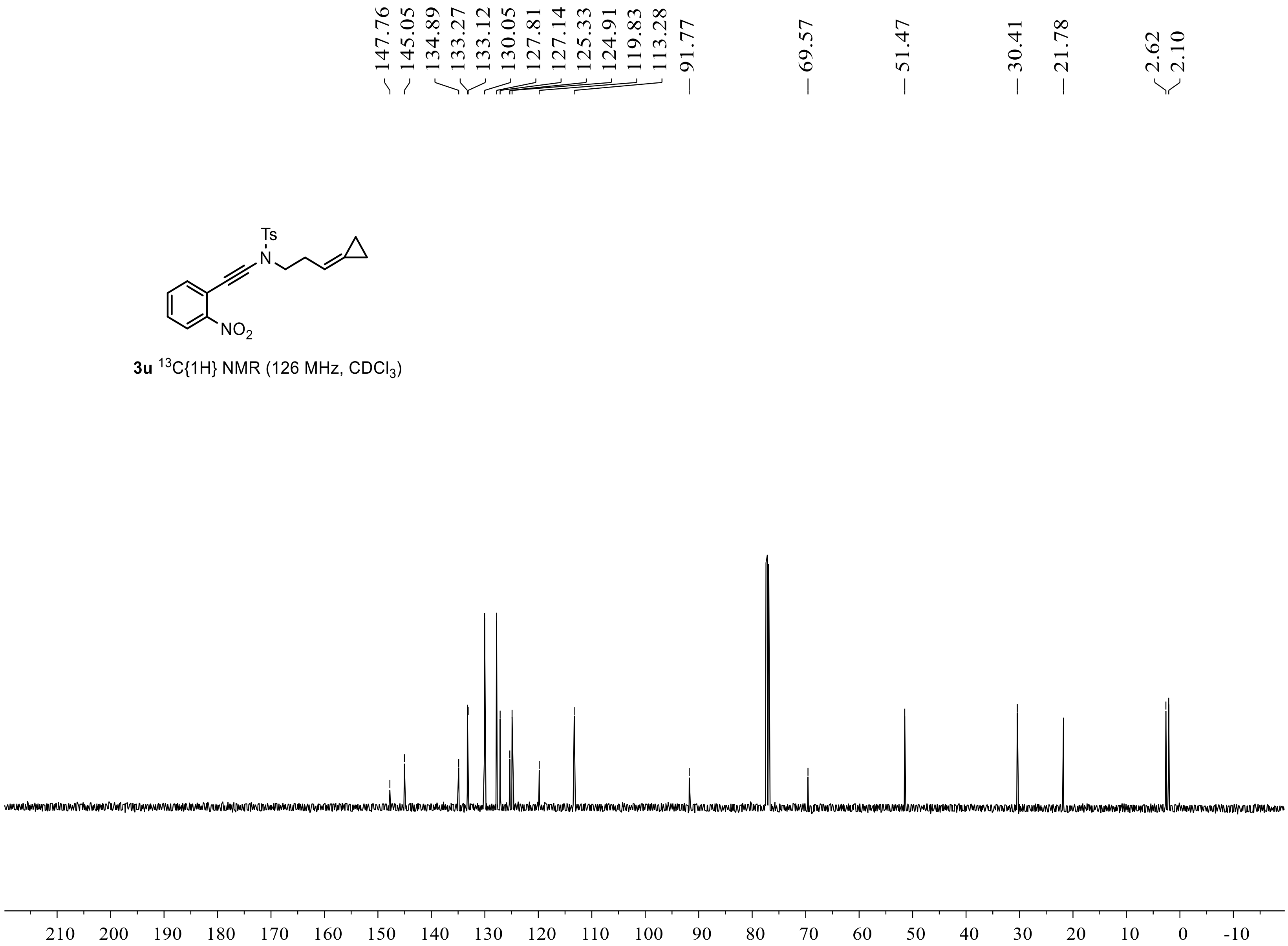

Supplementary Figure 153. ^{13}C NMR ($CDCl_3$, 126 MHz, 298 K) spectrum for **3u**

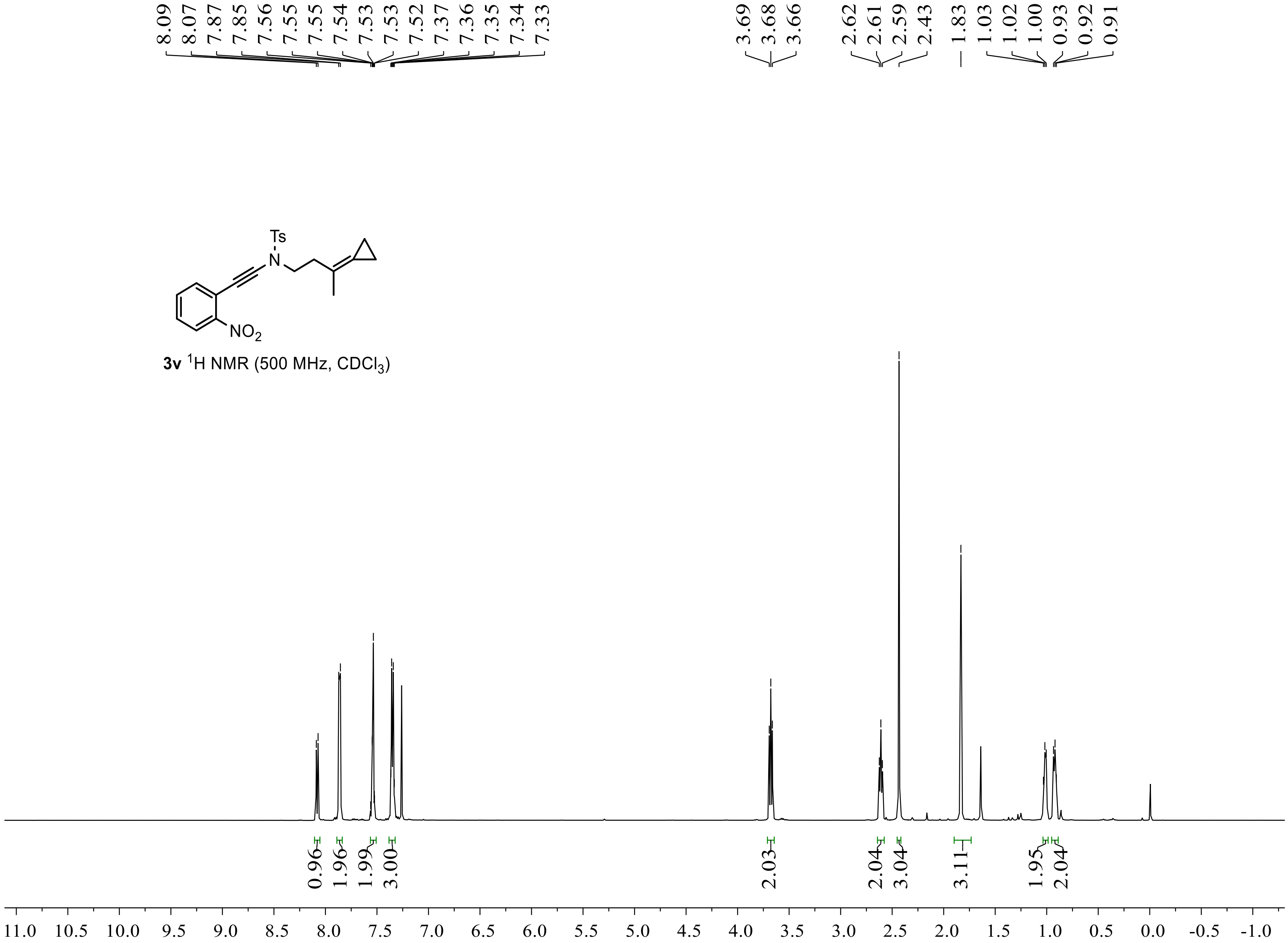

Supplementary Figure 154. ^{1}H NMR ($CDCl_3$, 500 MHz, 298 K) spectrum for **3v**

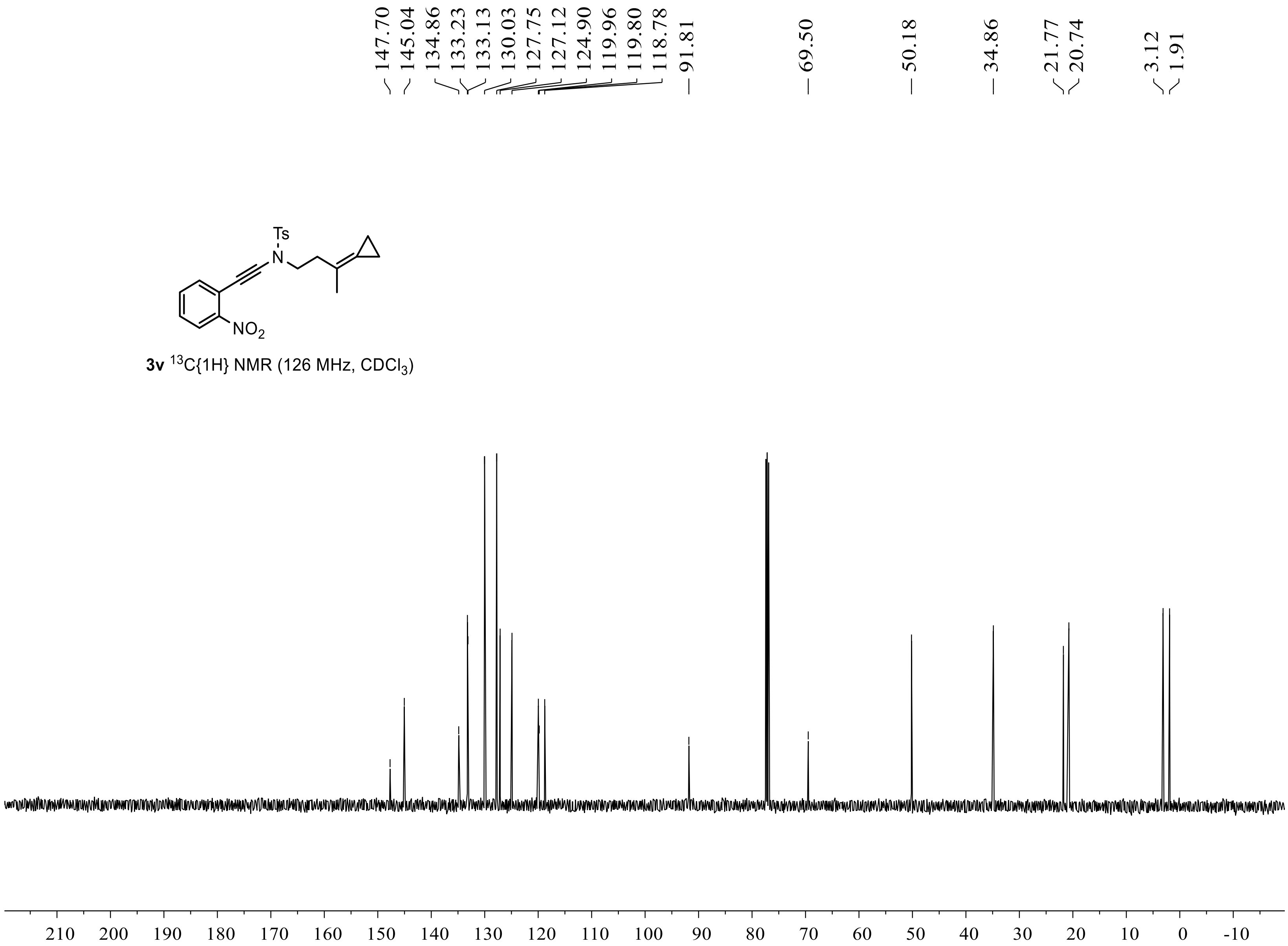

Supplementary Figure 155. ^{13}C NMR ($CDCl_3$, 126 MHz, 298 K) spectrum for **3v**

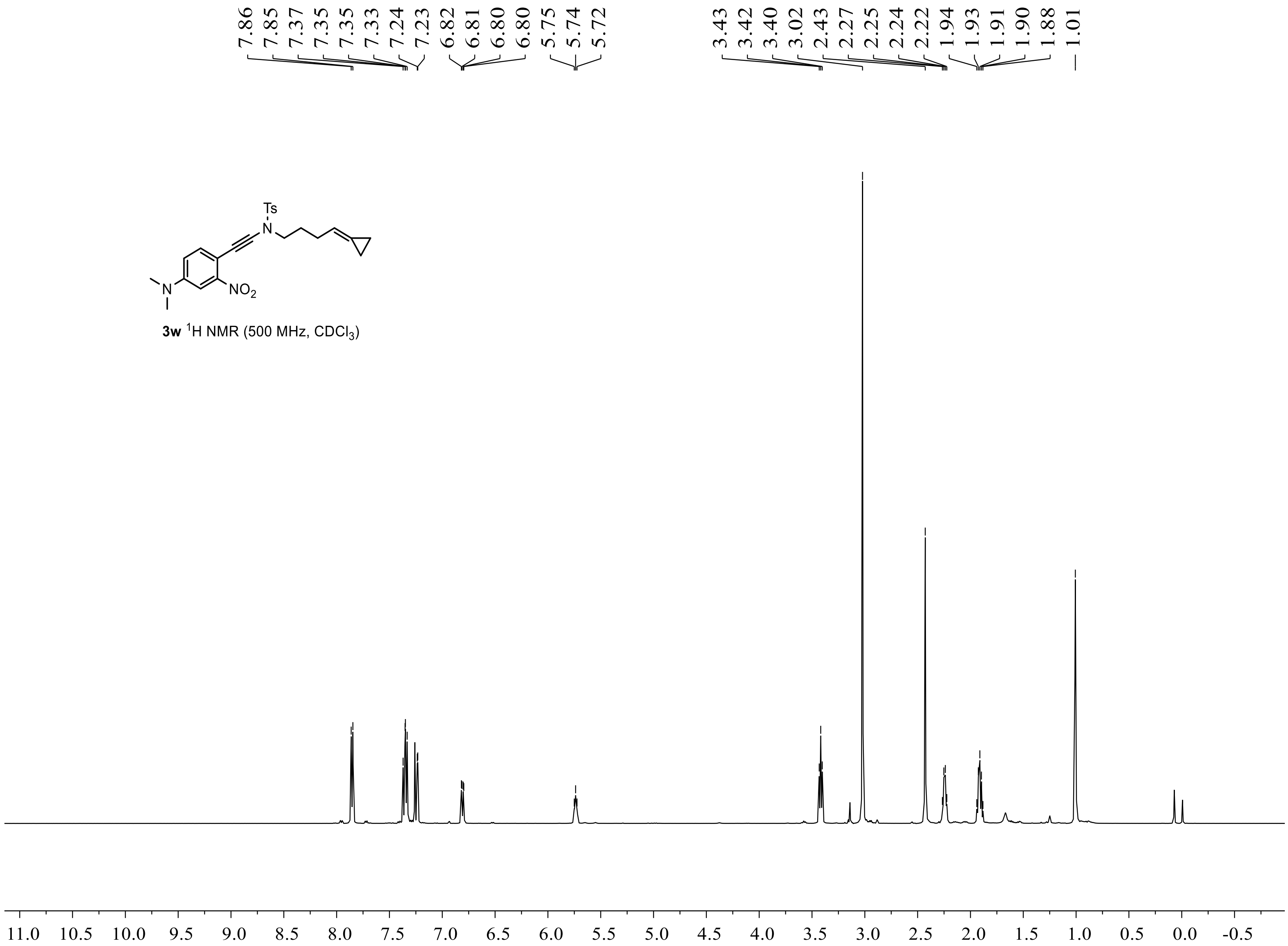

Supplementary Figure 156. ^{1}H NMR ($CDCl_3$, 500 MHz, 298 K) spectrum for **3w**

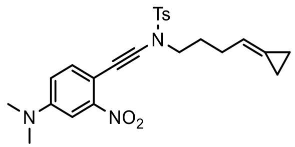

3w $^{13}C\{1H\}$ NMR (126 MHz, $CDCl_3$)

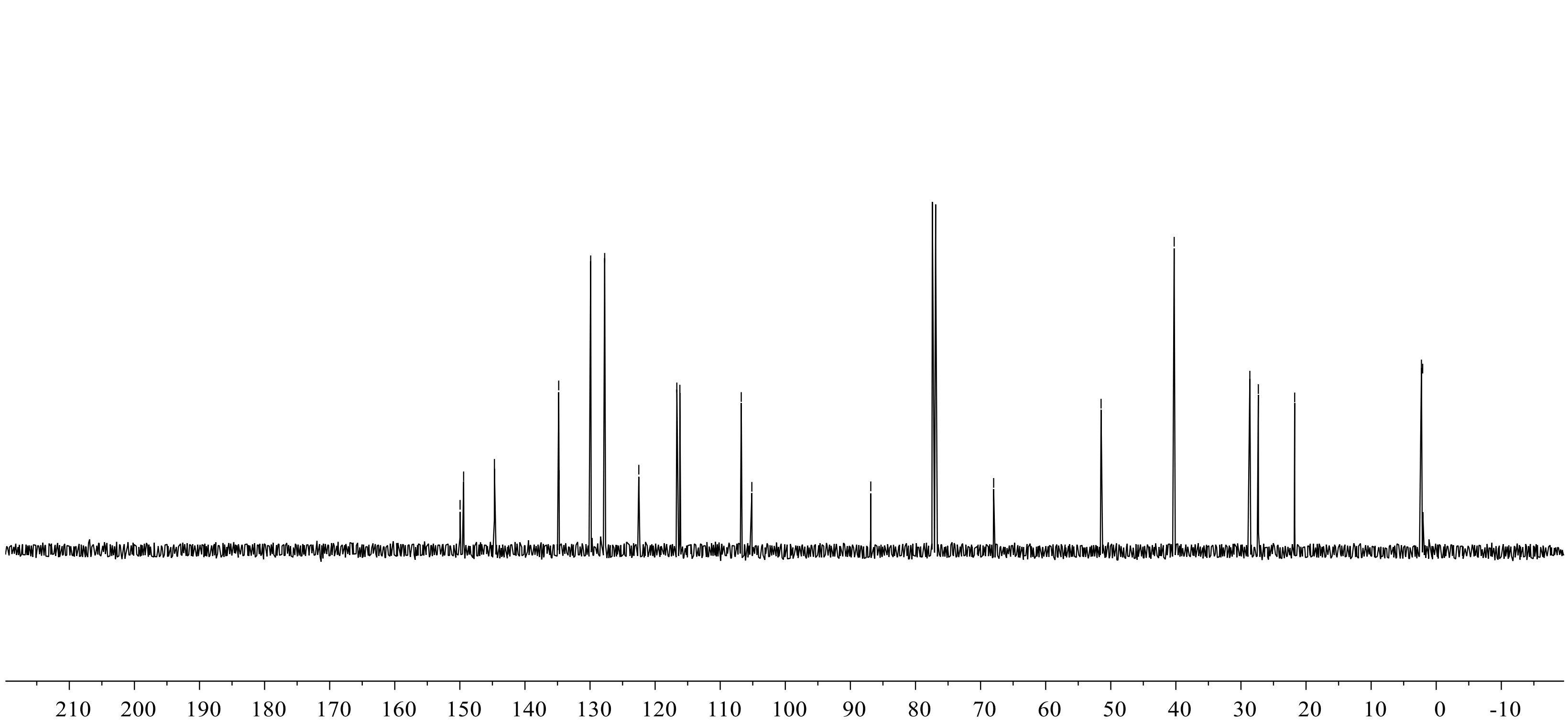

Supplementary Figure 157. ^{13}C NMR ($CDCl_3$, 126 MHz, 298 K) spectrum for **3w**

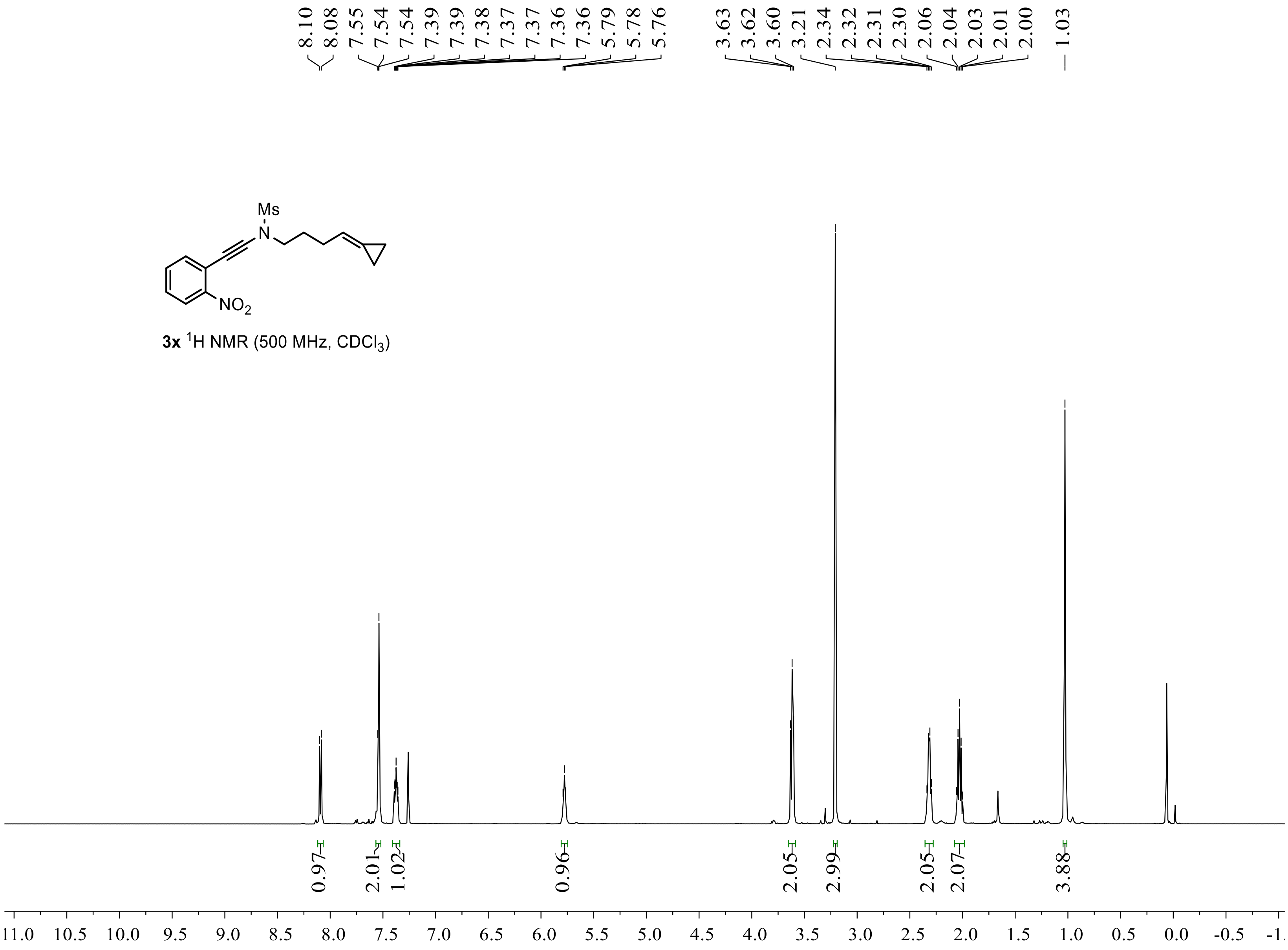

Supplementary Figure 158. ^{1}H NMR ($CDCl_3$, 500 MHz, 298 K) spectrum for **3x**

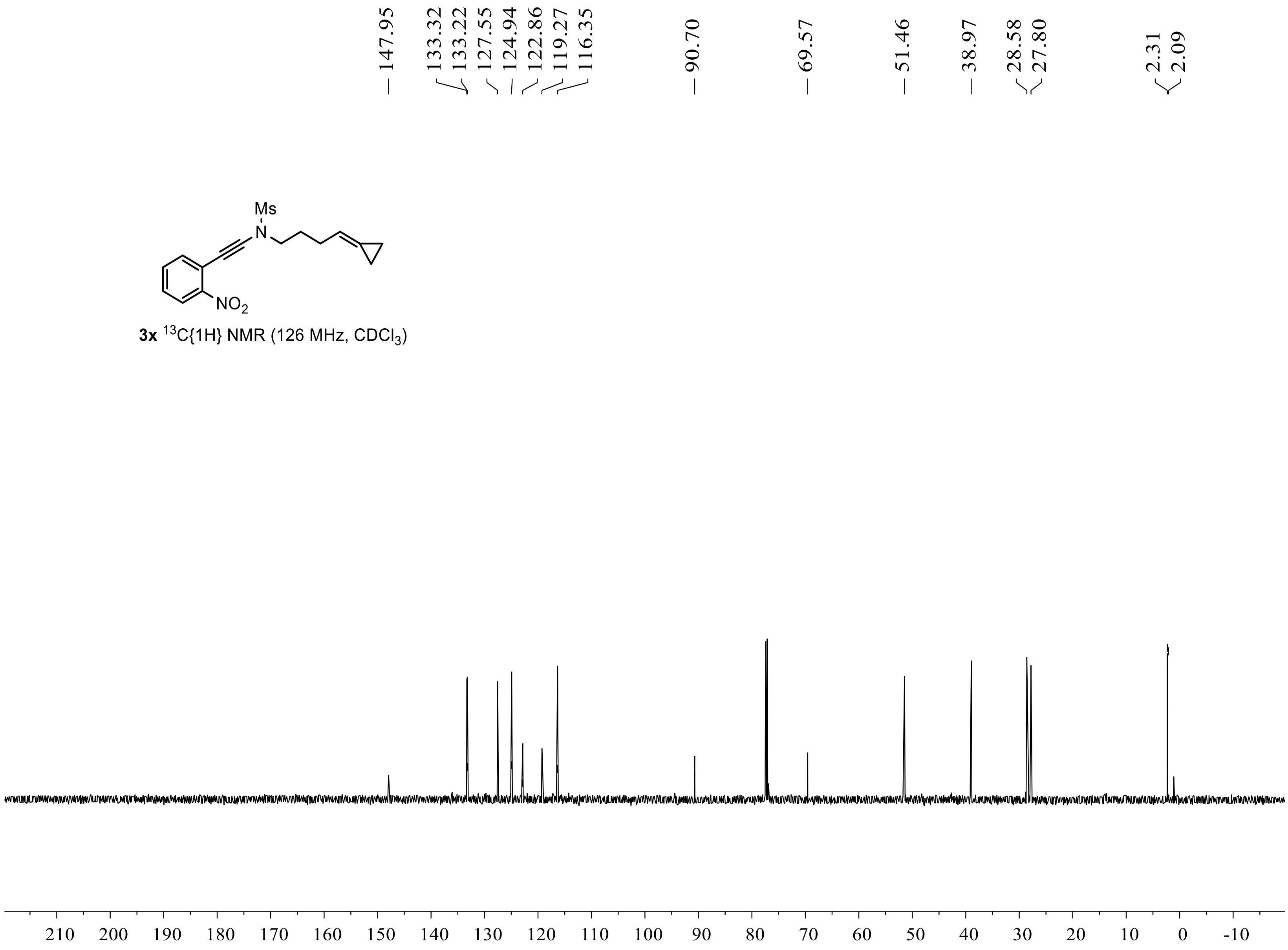

Supplementary Figure 159. ^{13}C NMR ($CDCl_3$, 126 MHz, 298 K) spectrum for **3x**

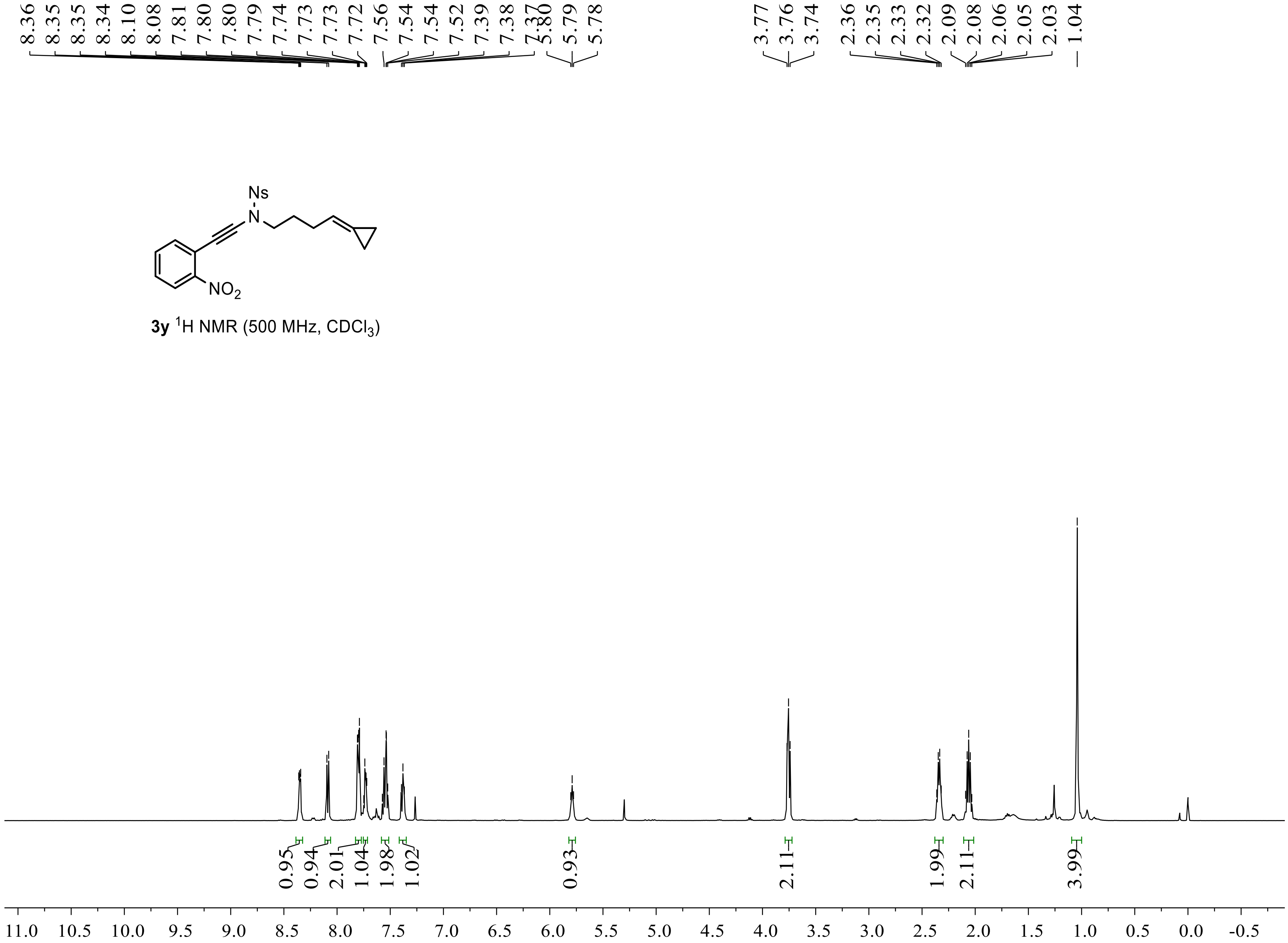

Supplementary Figure 160. ¹H NMR ($CDCl_3$, 500 MHz, 298 K) spectrum for **3y**

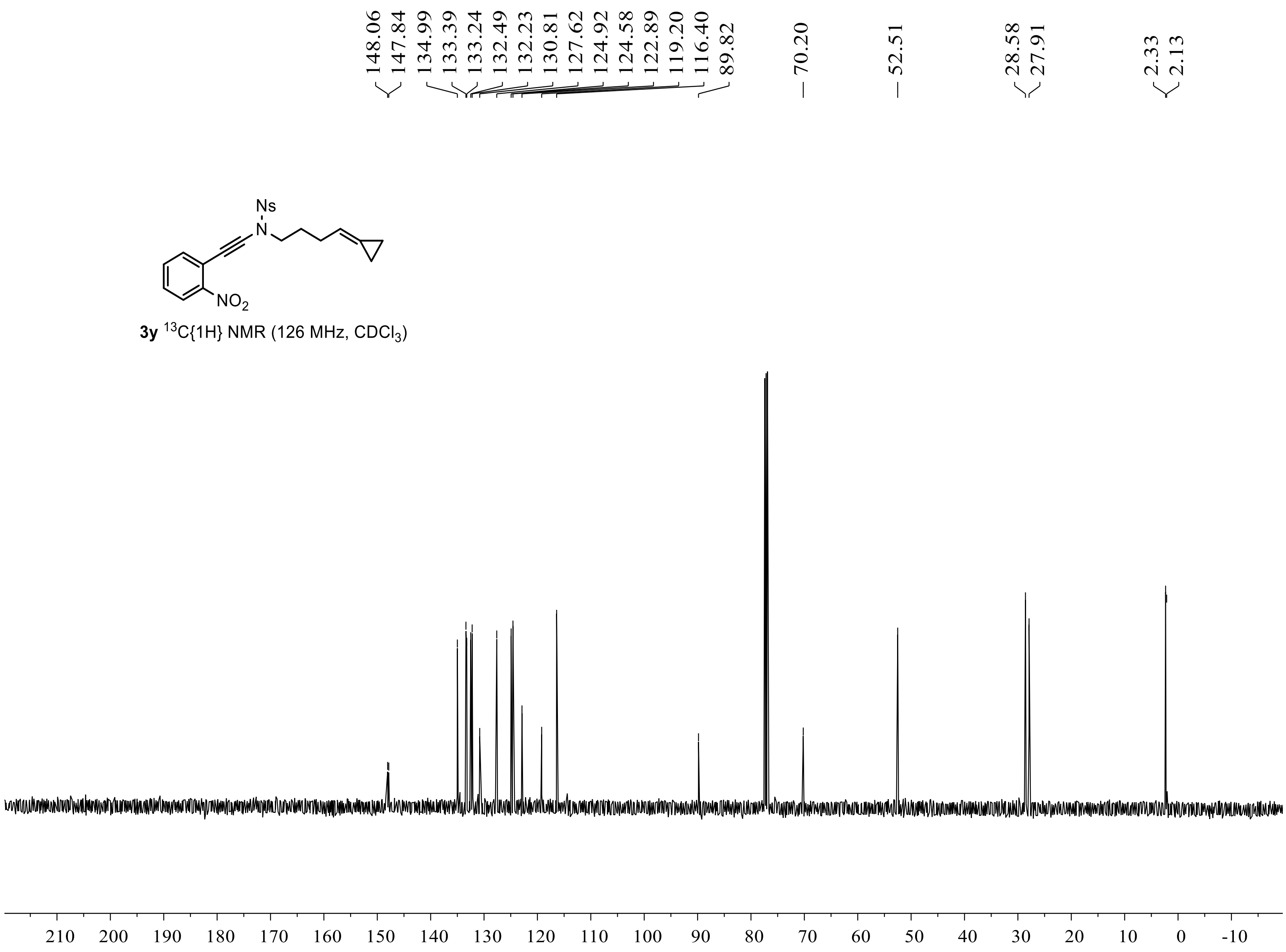

Supplementary Figure 161. ^{13}C NMR ($CDCl_3$, 126 MHz, 298 K) spectrum for **3y**

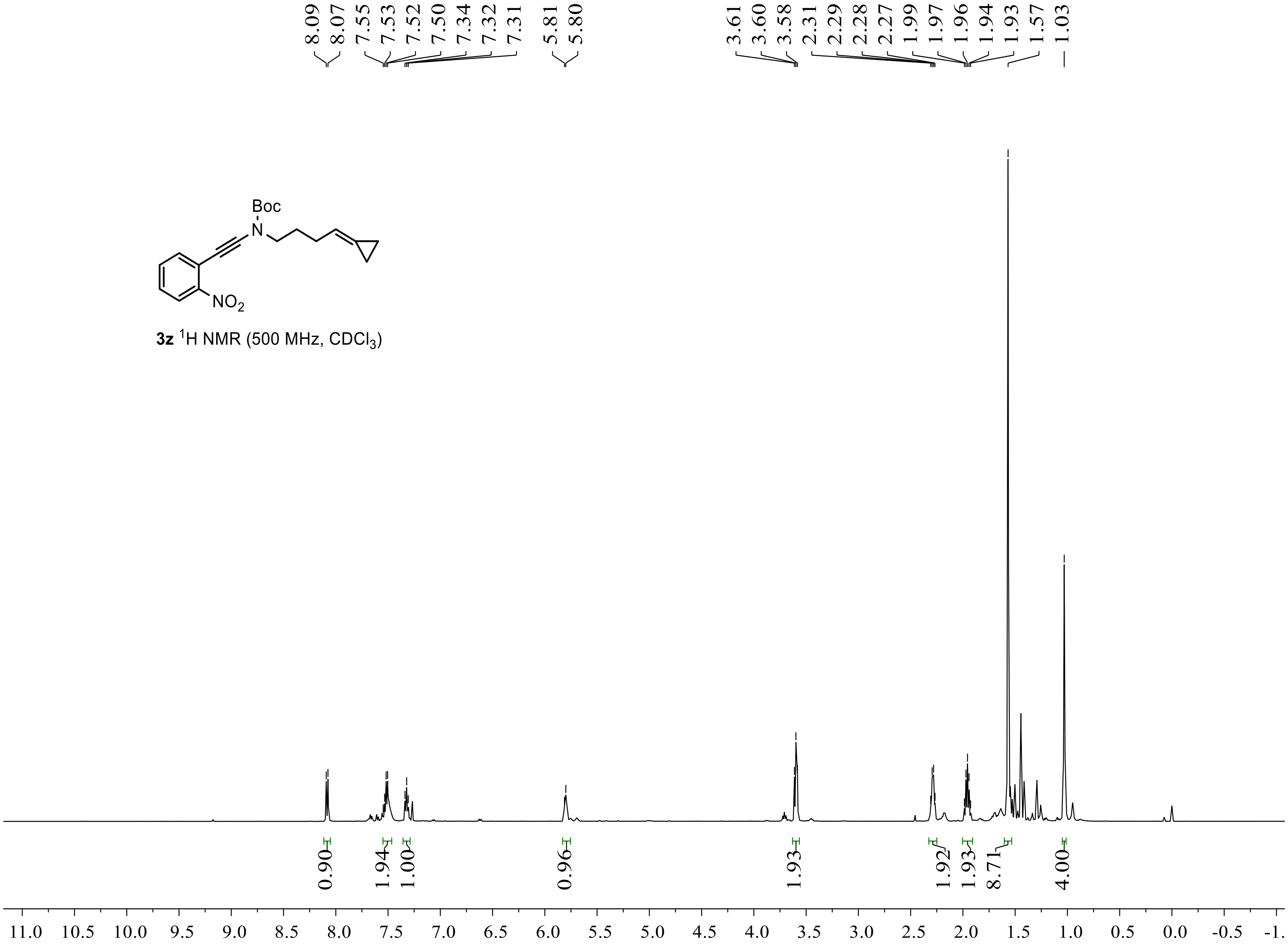

Supplementary Figure 162. ^{1}H NMR ($CDCl_3$, 500 MHz, 298 K) spectrum for **3z**

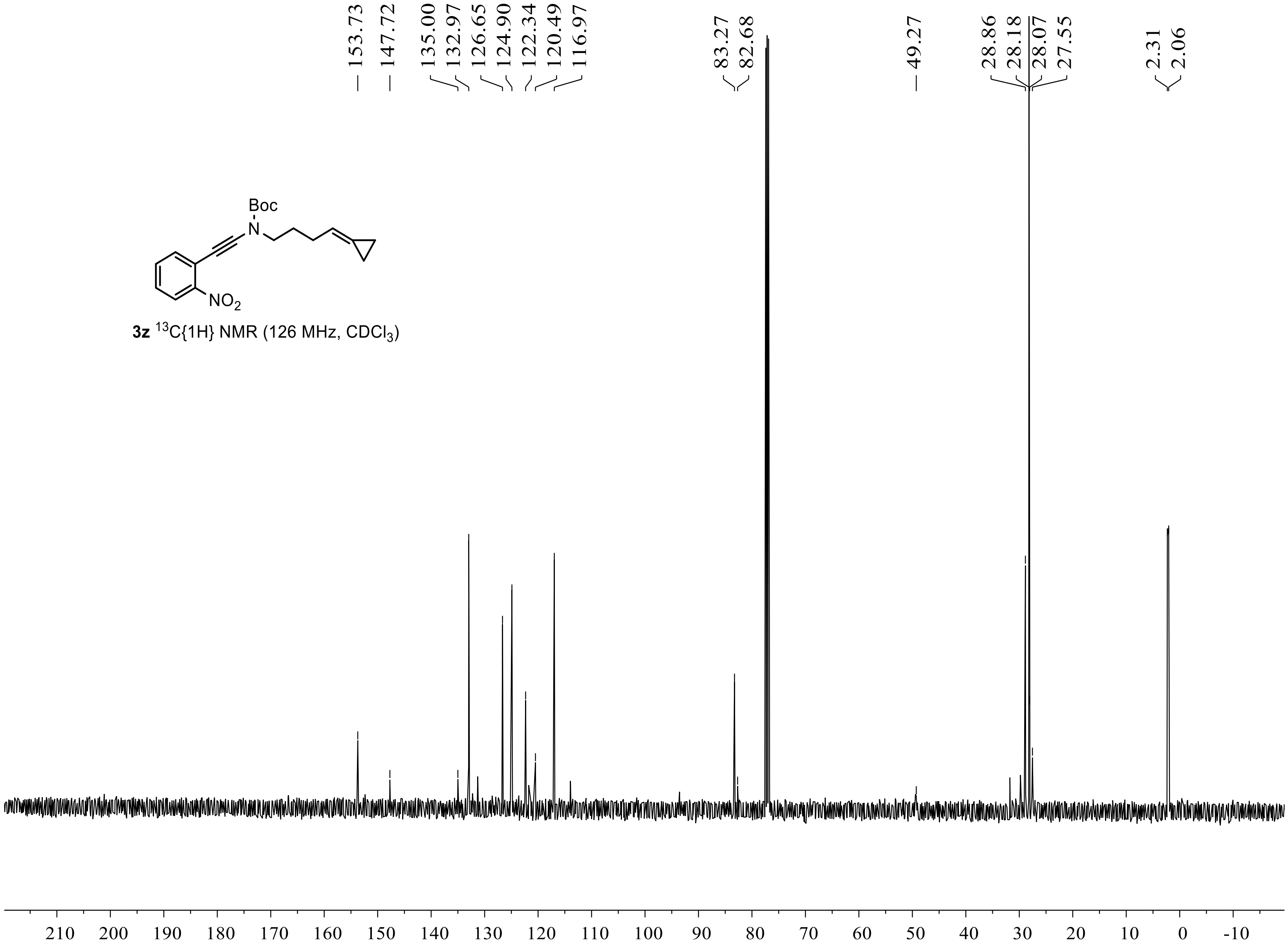

Supplementary Figure 163. ^{13}C NMR ($CDCl_3$, 126 MHz, 298 K) spectrum for **3z**

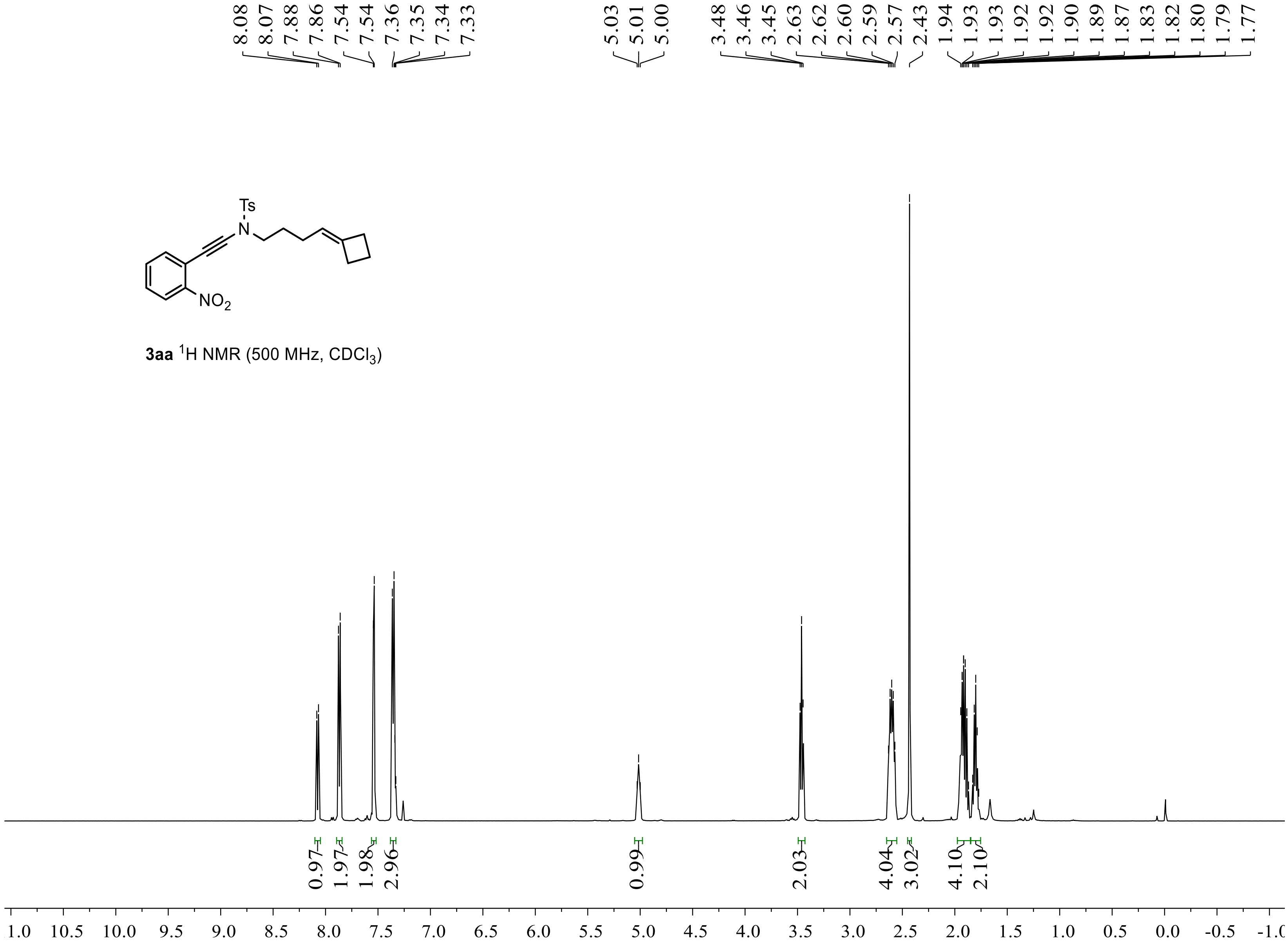

Supplementary Figure 164. ^{1}H NMR ($CDCl_3$, 500 MHz, 298 K) spectrum for **3aa**

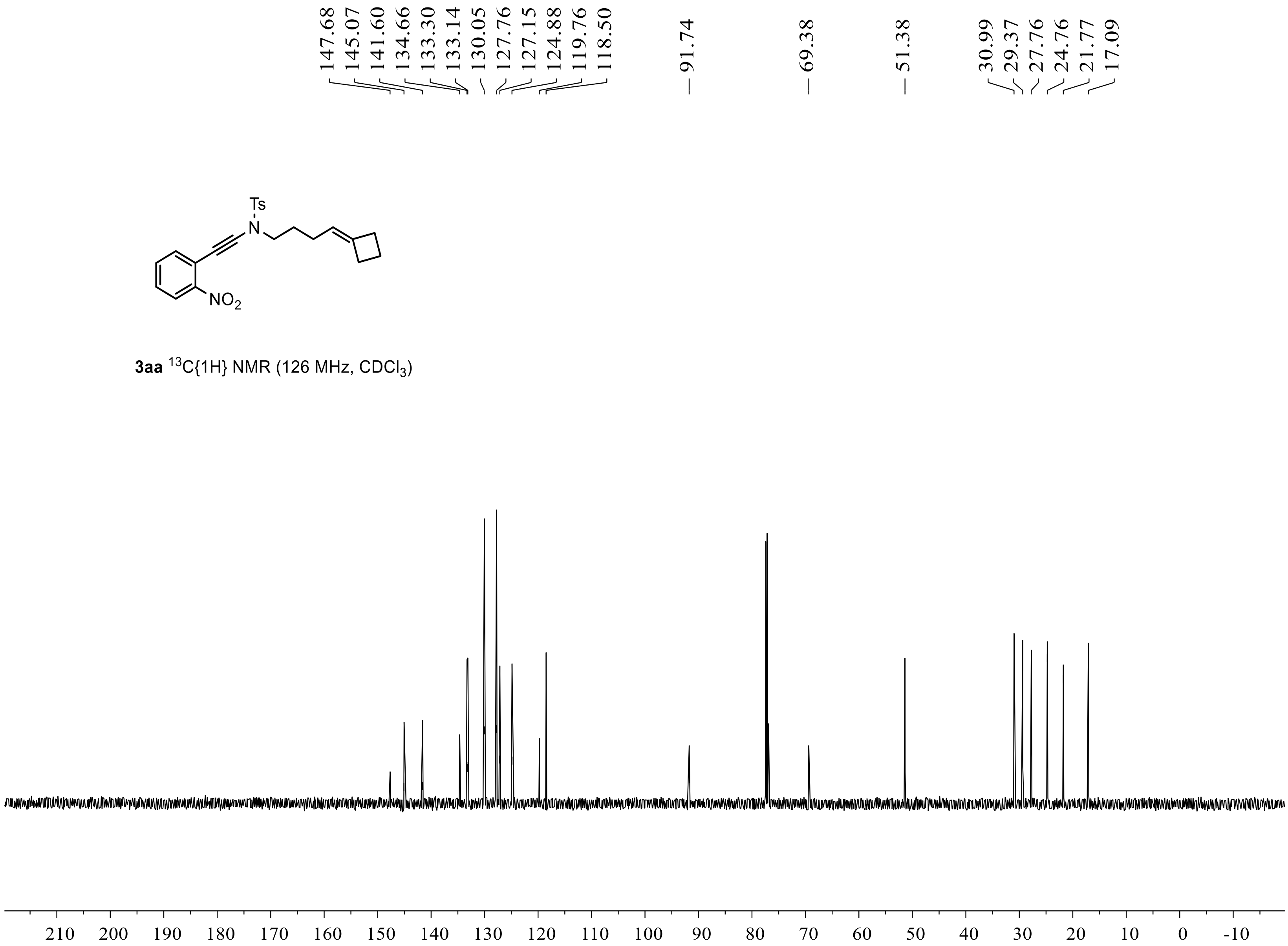

Supplementary Figure 165. ^{13}C NMR ($CDCl_3$, 126 MHz, 298 K) spectrum for **3aa**

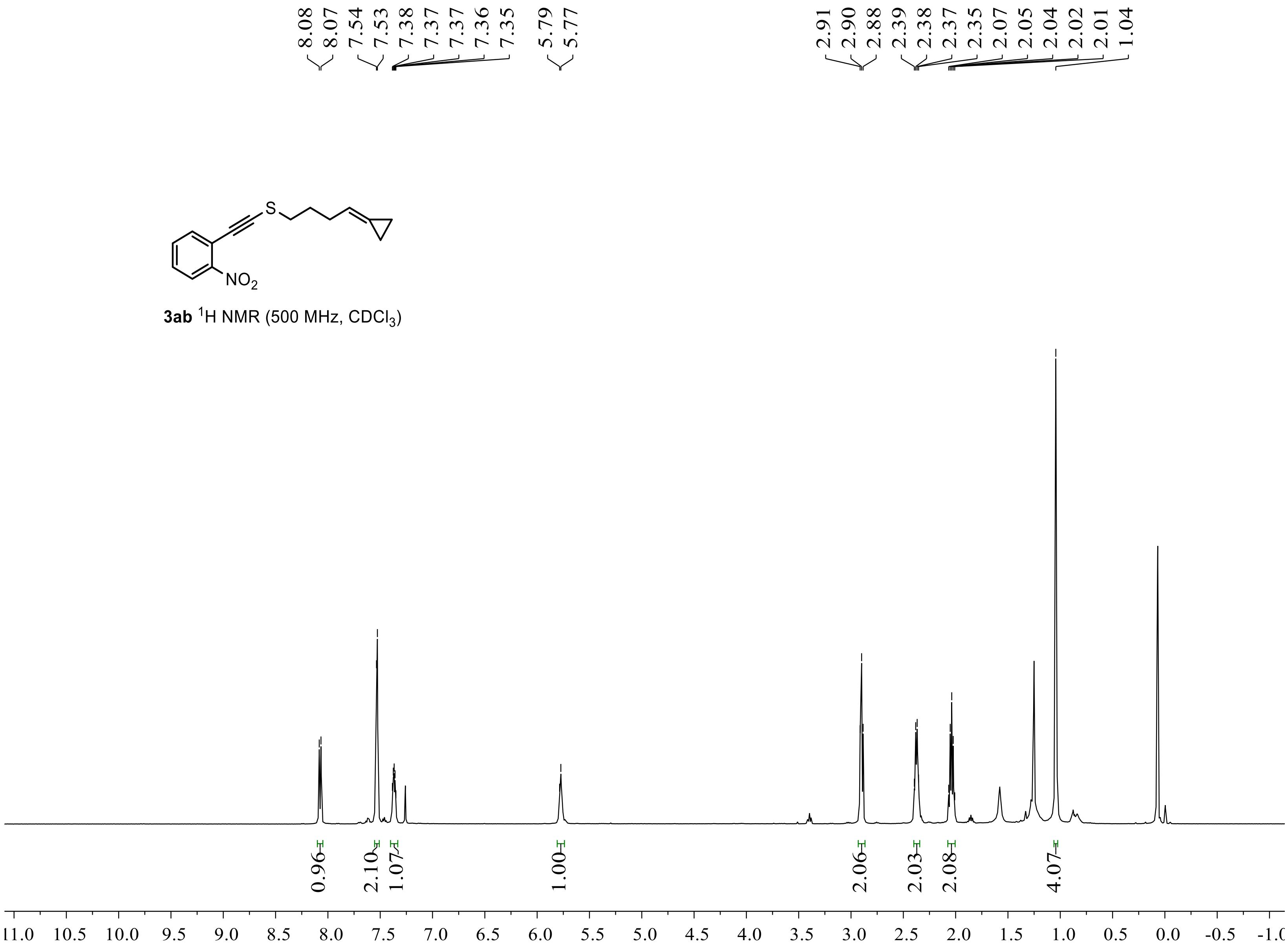

Supplementary Figure 166. ^1H NMR ($CDCl_3$, 500 MHz, 298 K) spectrum for **3ab**

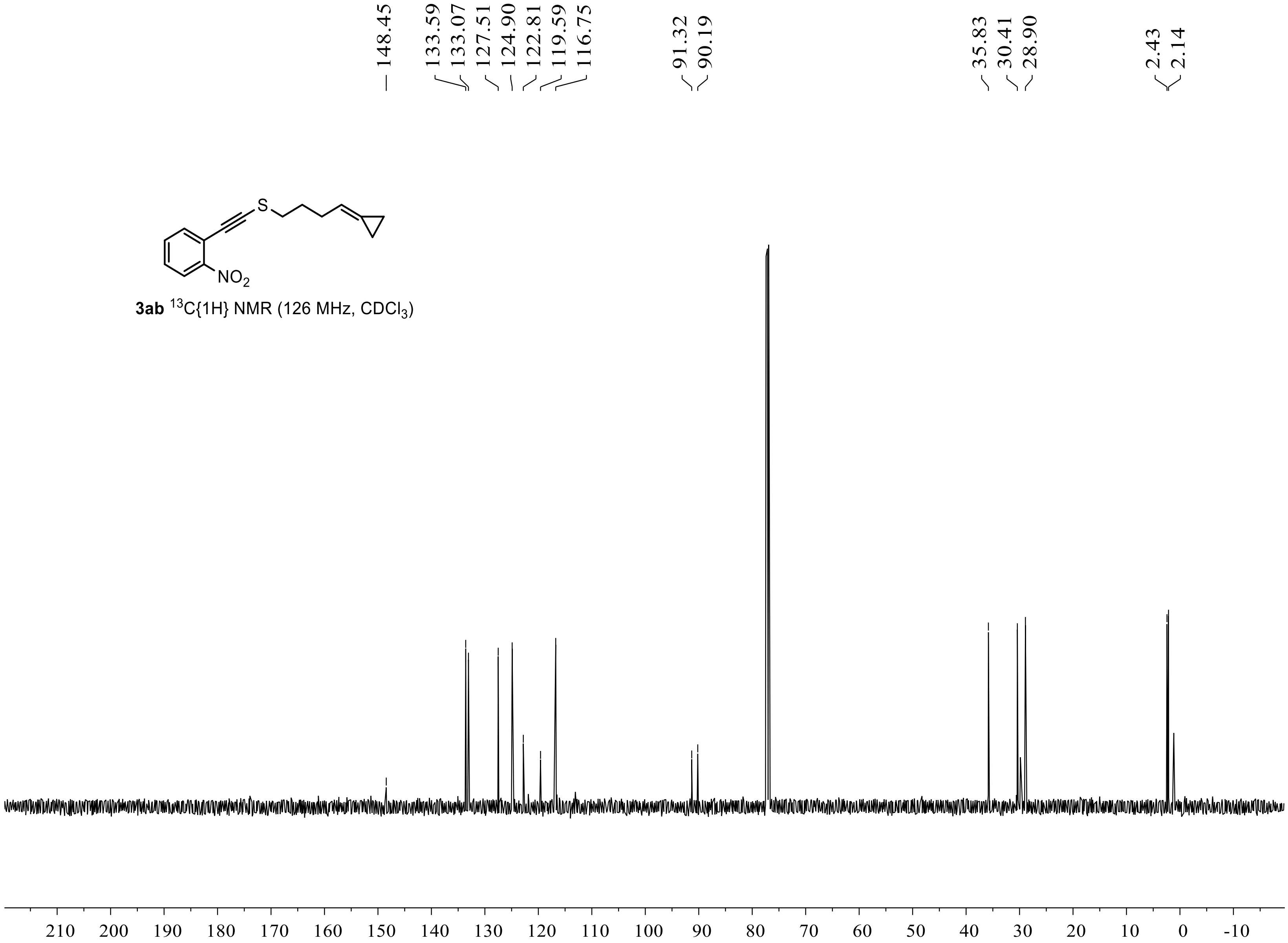

Supplementary Figure 167. ^{13}C NMR ($CDCl_3$, 126 MHz, 298 K) spectrum for **3ab**

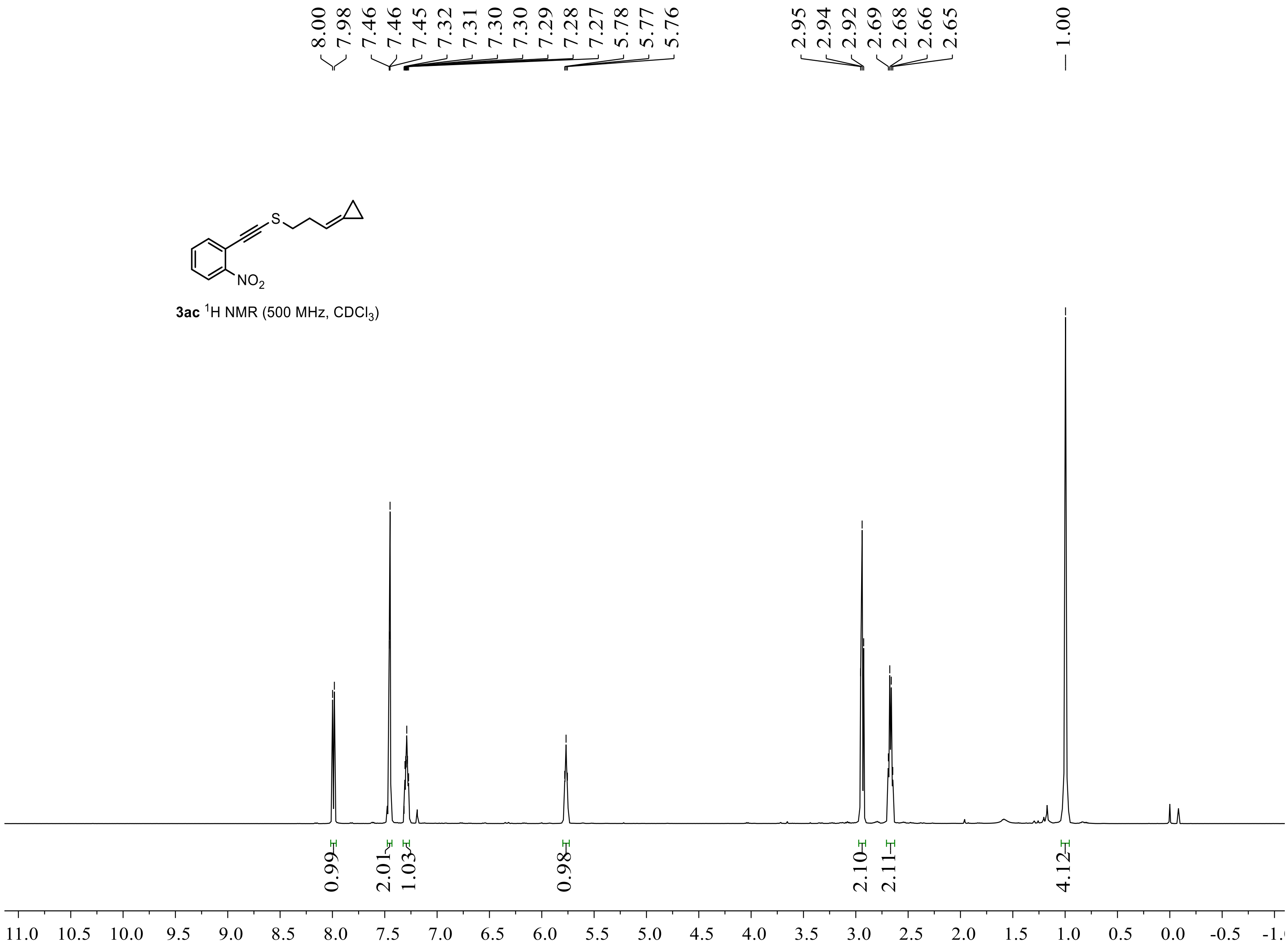

Supplementary Figure 168. ^{1}H NMR ($CDCl_3$, 500 MHz, 298 K) spectrum for **3ac**

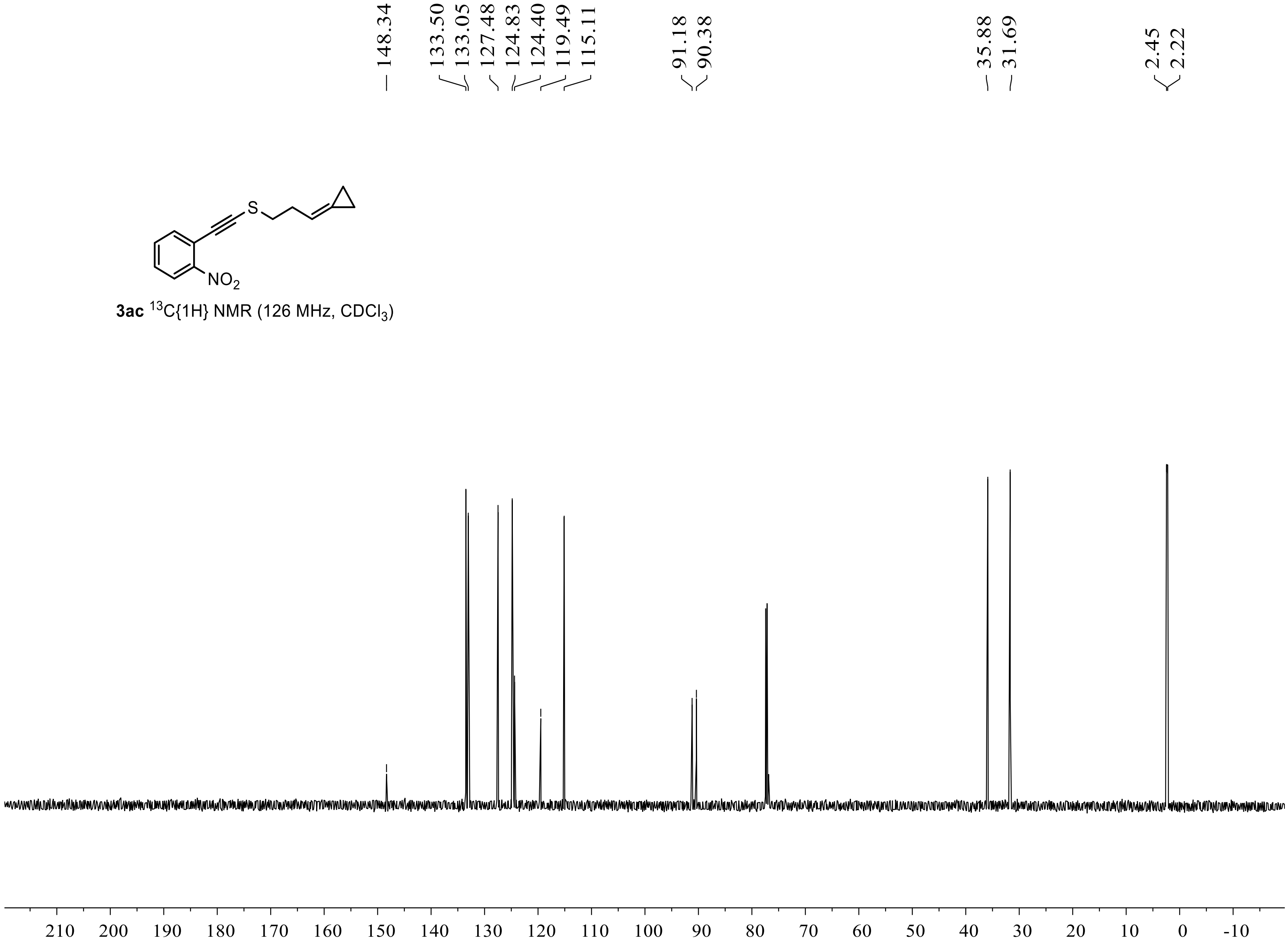

Supplementary Figure 169. ^{13}C NMR ($CDCl_3$, 126 MHz, 298 K) spectrum for **3ac**

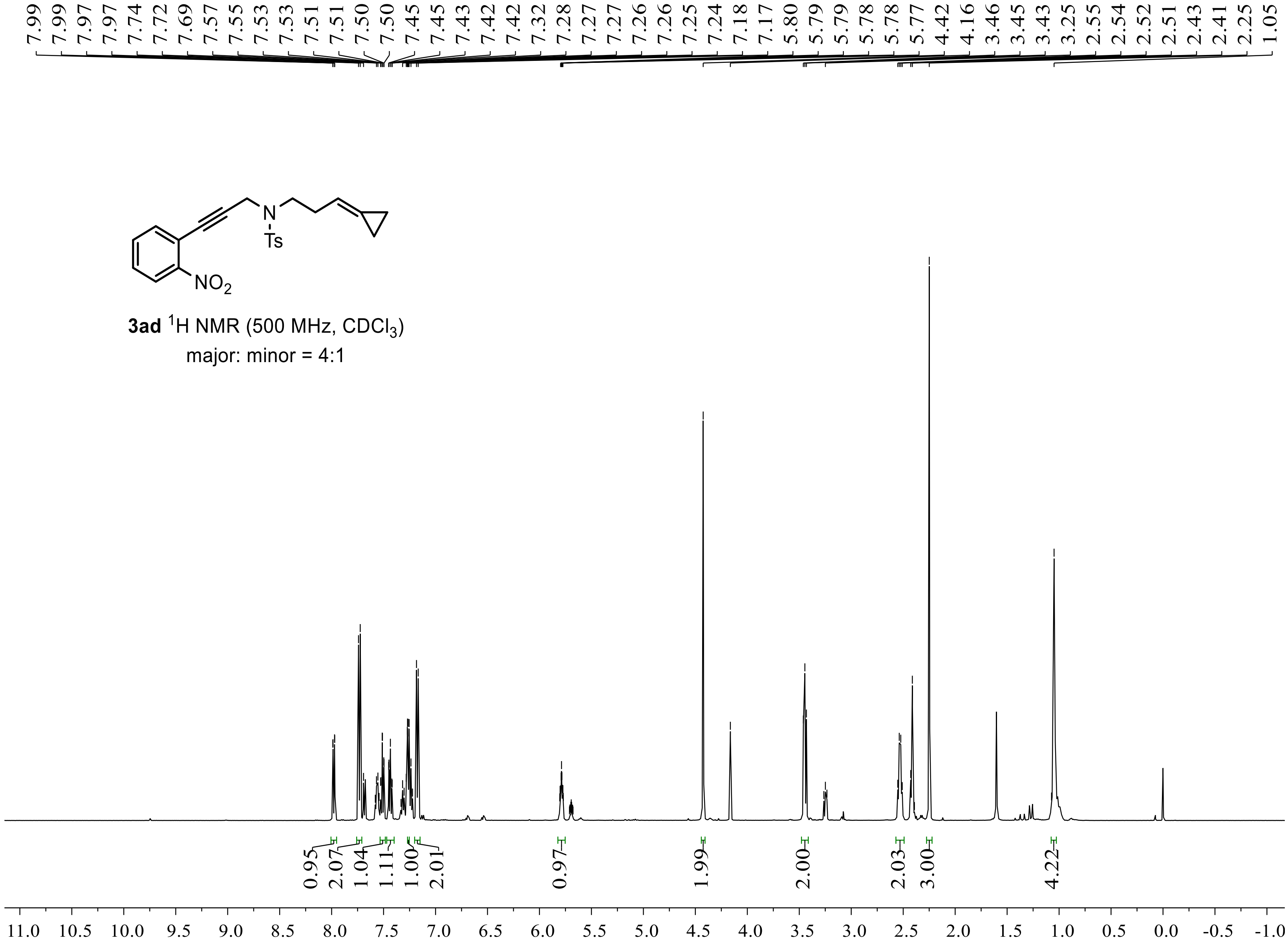

Supplementary Figure 170. ^{1}H NMR ($CDCl_3$, 500 MHz, 298 K) spectrum for **3ad**

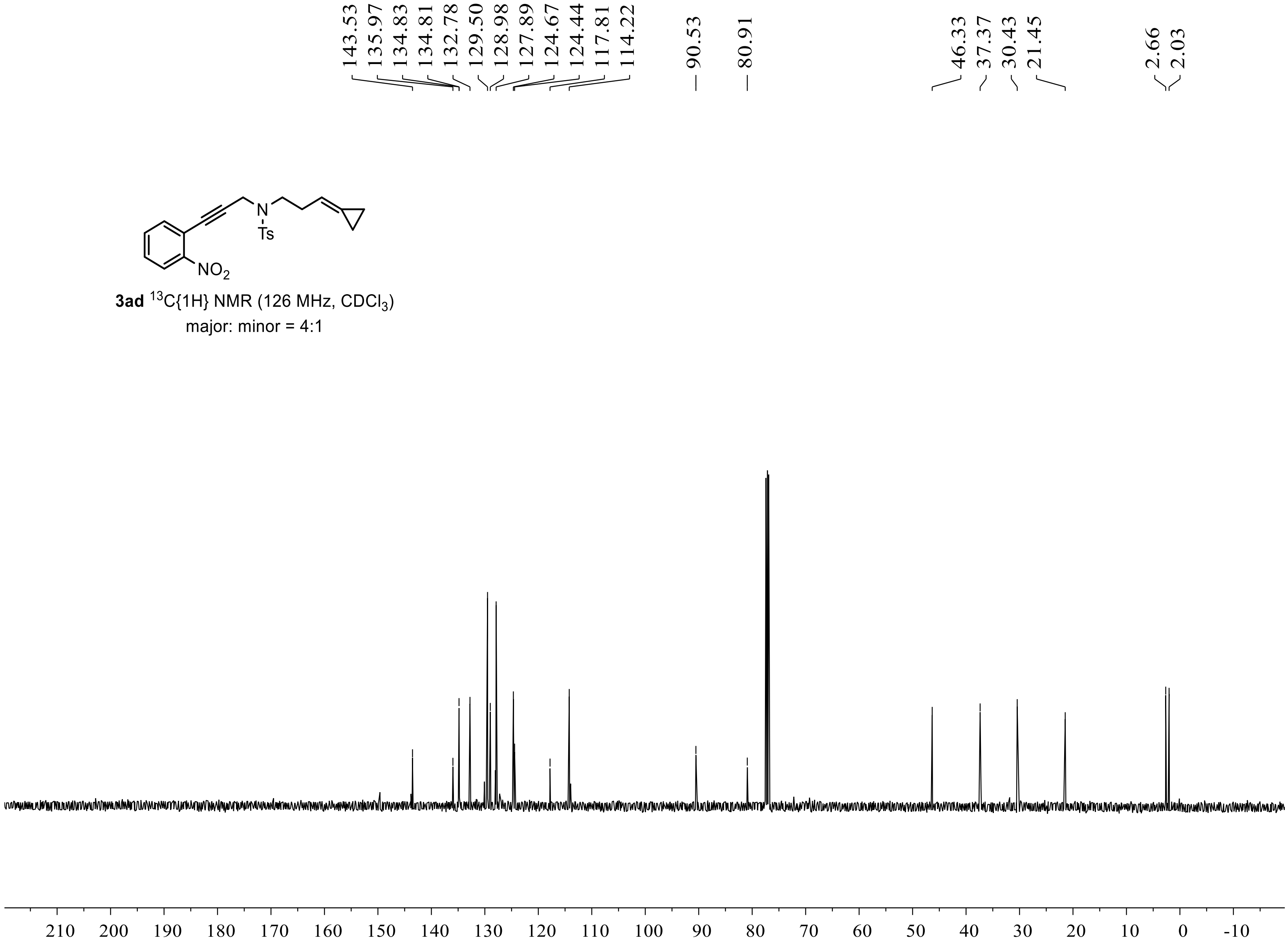

Supplementary Figure 171. ^{13}C NMR ($CDCl_3$, 126 MHz, 298 K) spectrum for **3ad**

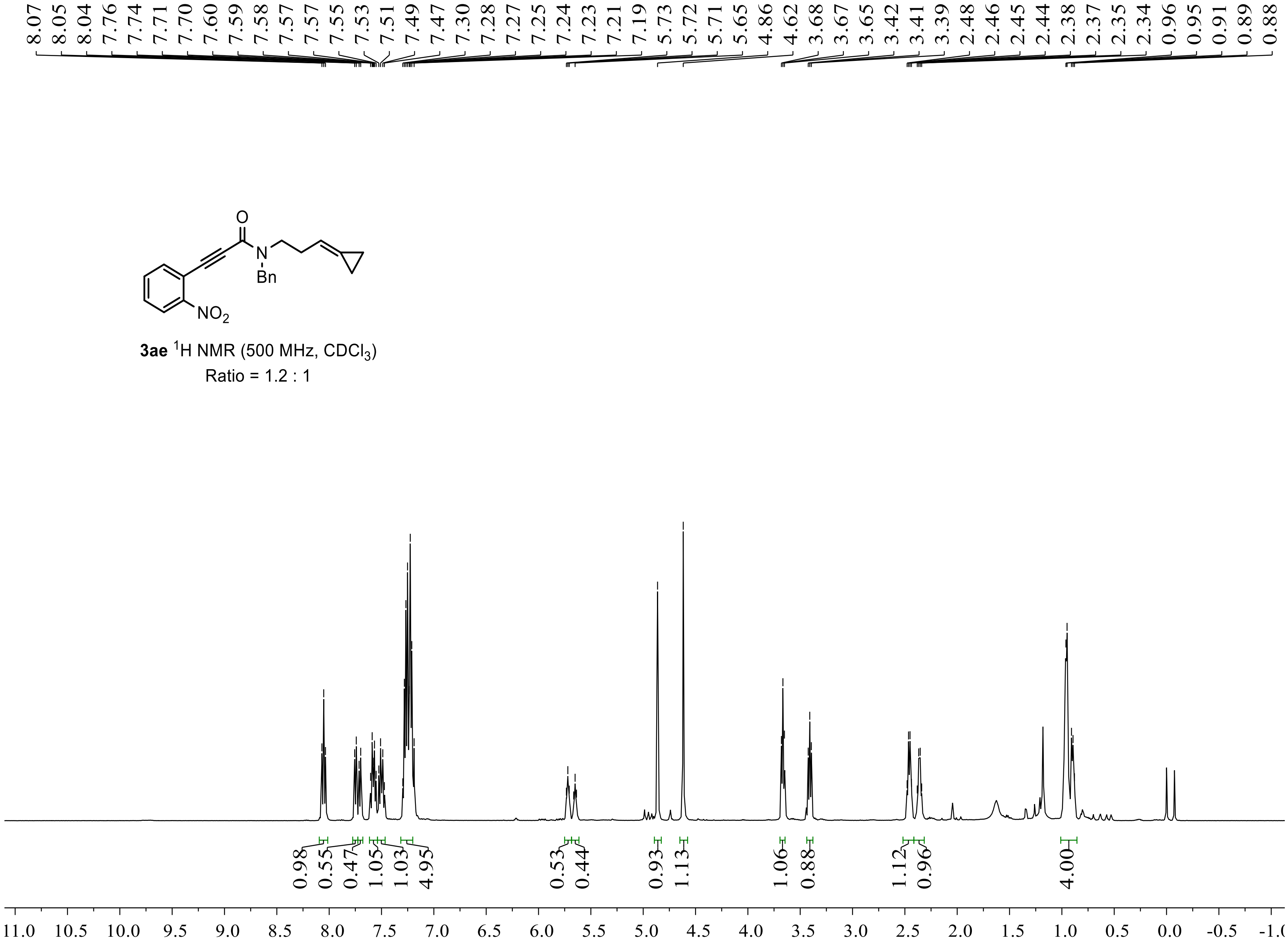

Supplementary Figure 172. ^{1}H NMR ($CDCl_3$, 500 MHz, 298 K) spectrum for **3ae**

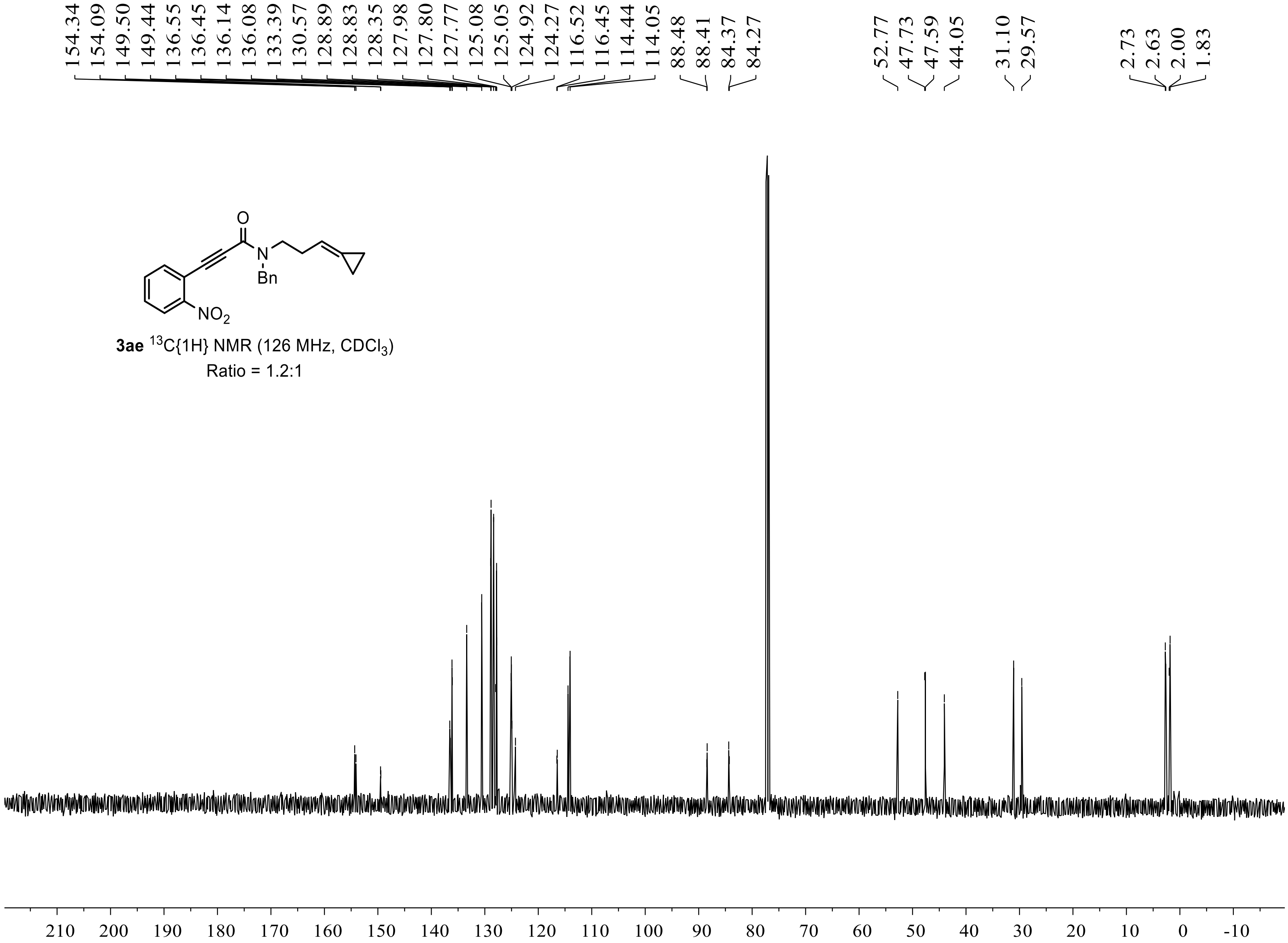

Supplementary Figure 173. ^{13}C NMR ($CDCl_3$, 126 MHz, 298 K) spectrum for **3ae**

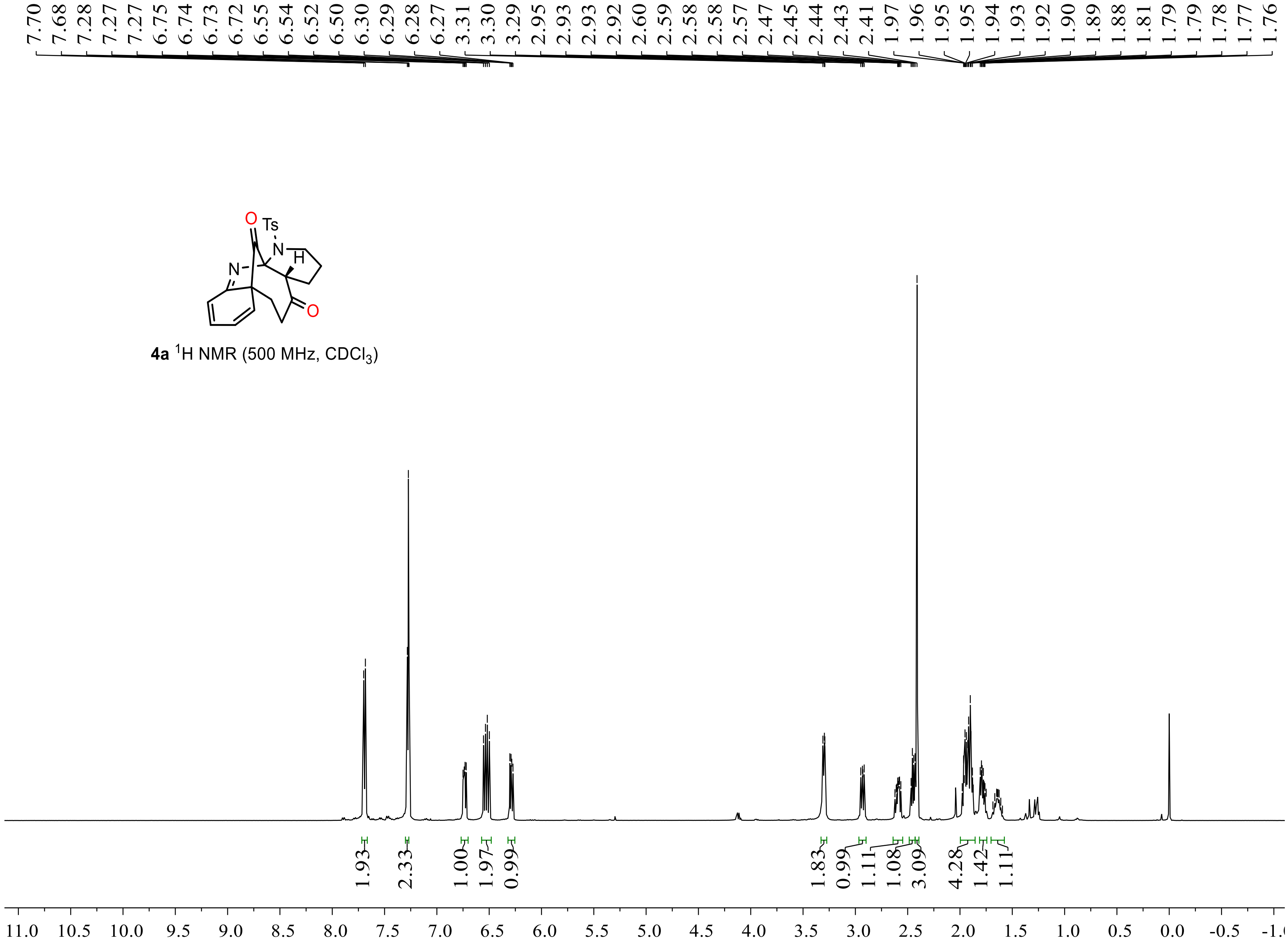

Supplementary Figure 174. ^{1}H NMR ($CDCl_3$, 500 MHz, 298 K) spectrum for **4a**

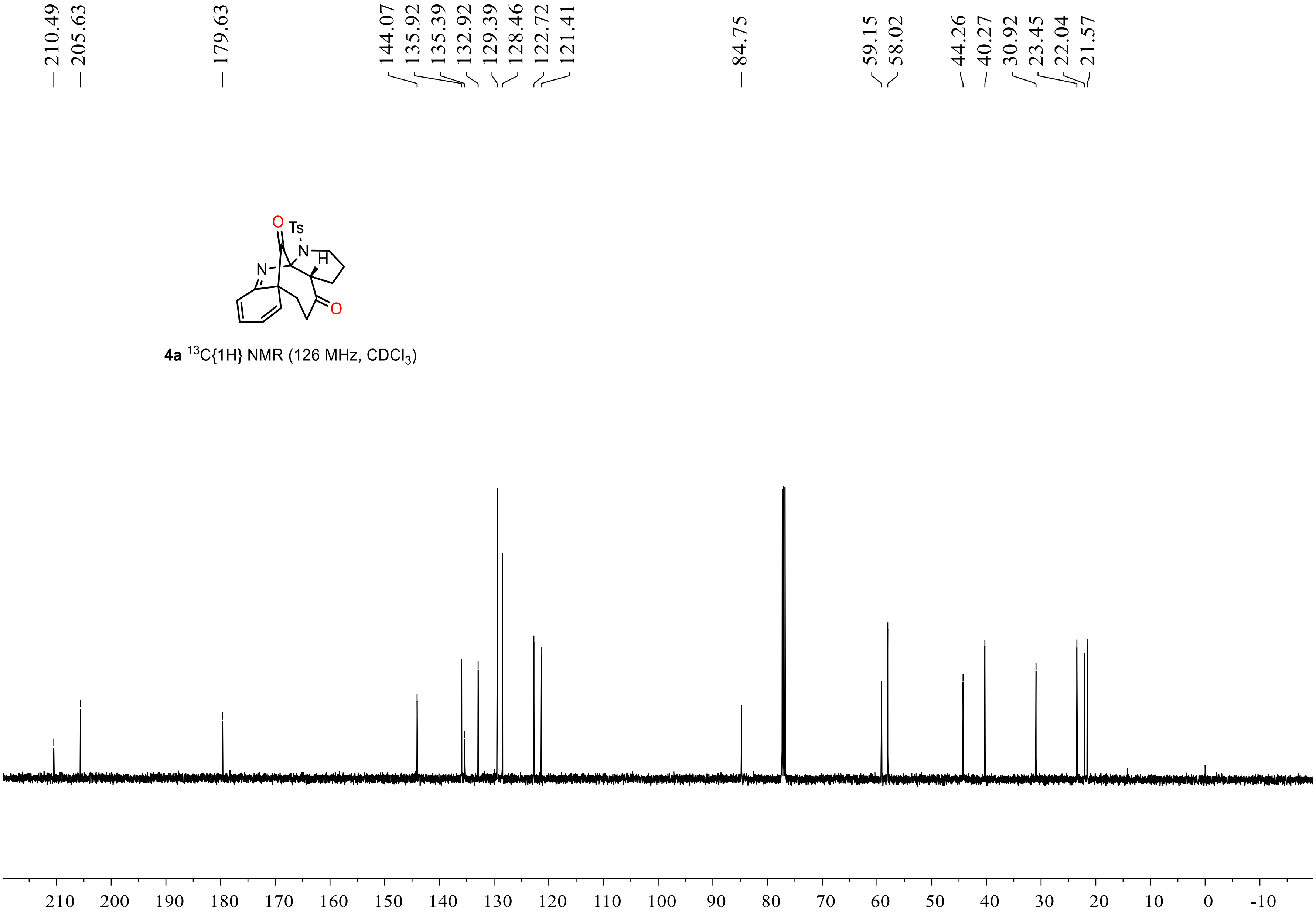

Supplementary Figure 175. ^{13}C NMR ($CDCl_3$, 126 MHz, 298 K) spectrum for **4a**

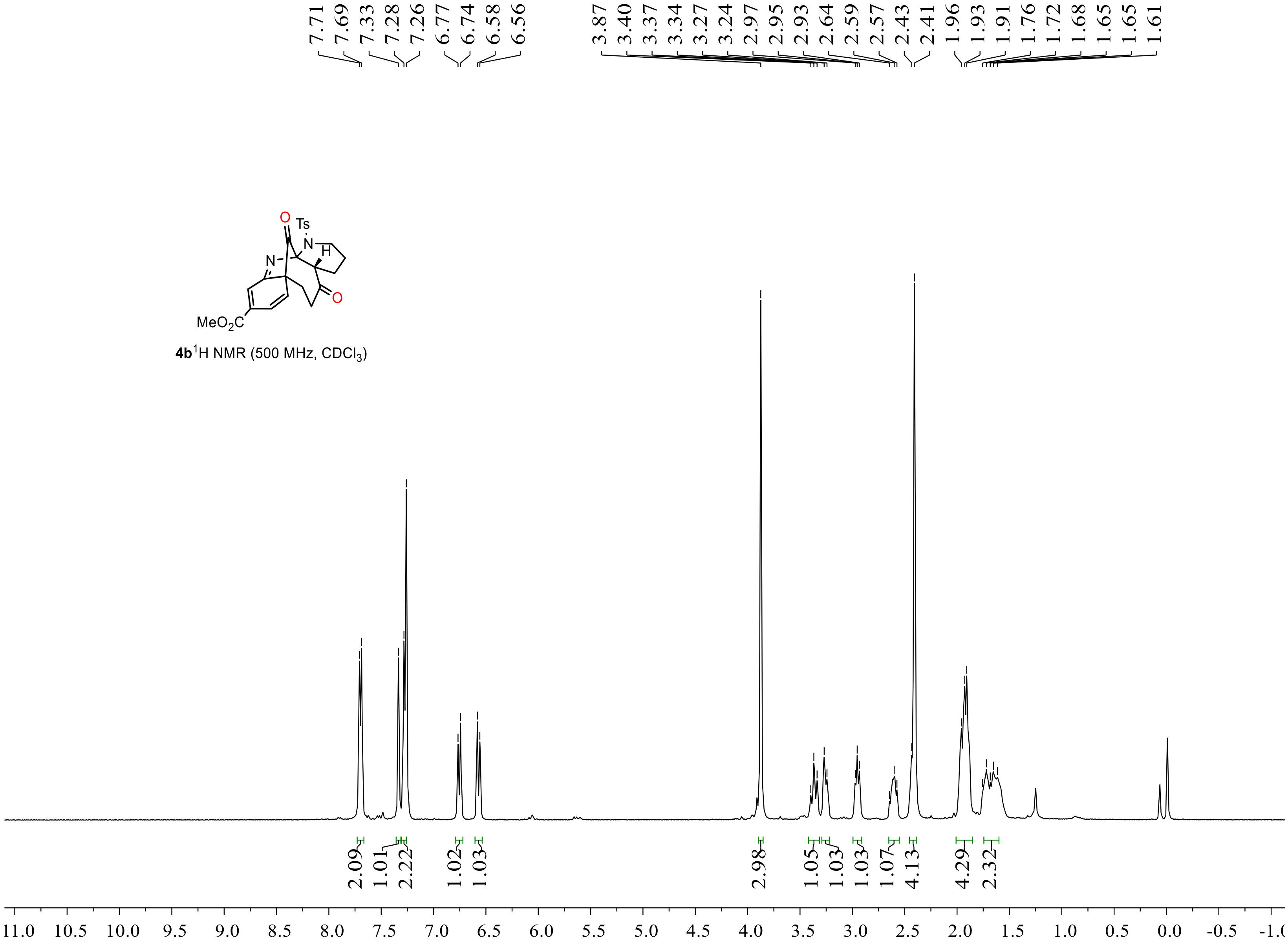

Supplementary Figure 176. ^{1}H NMR ($CDCl_3$, 500 MHz, 298 K) spectrum for **4b**

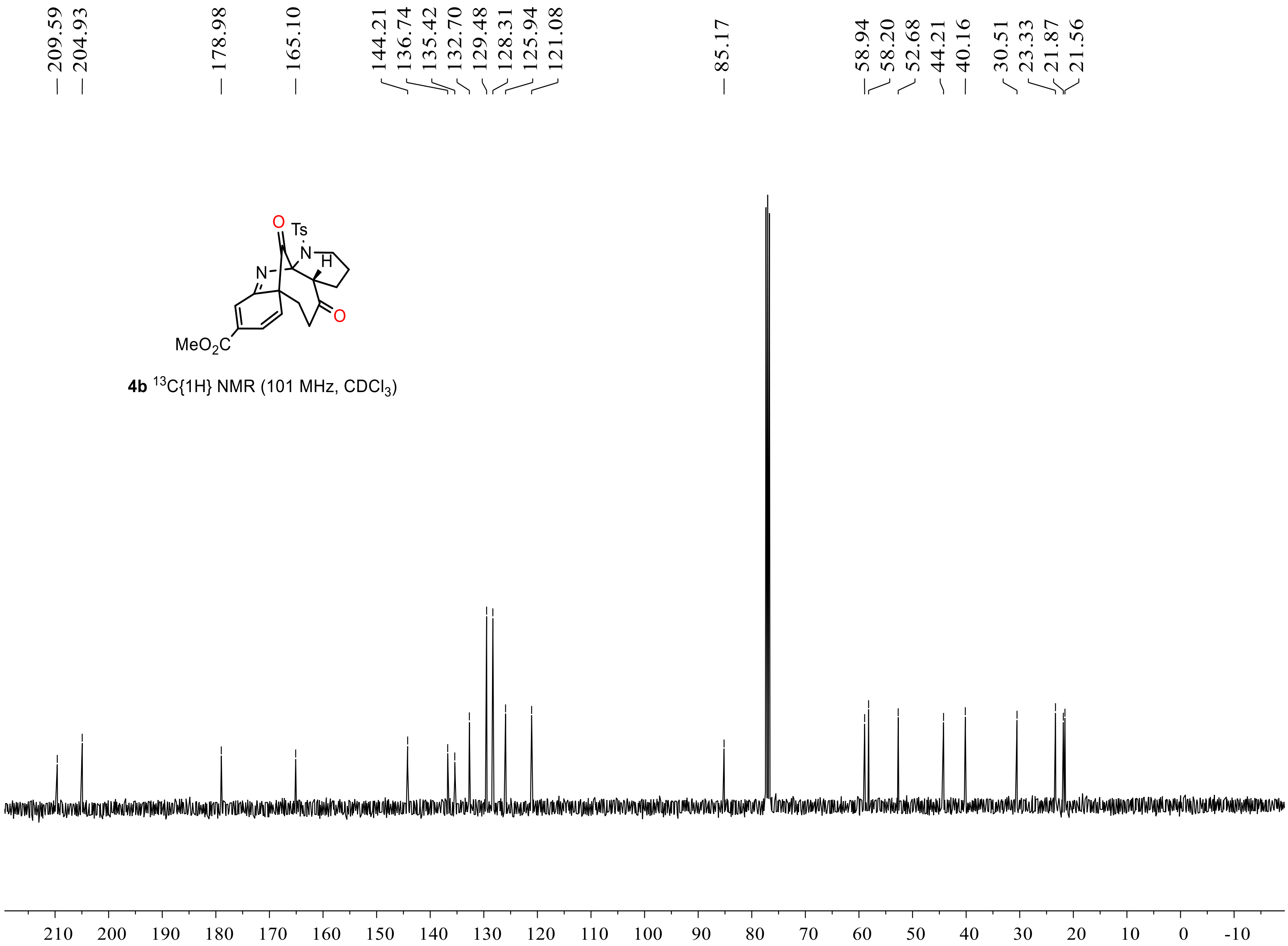

Supplementary Figure 177. ^{13}C NMR ($CDCl_3$, 126 MHz, 298 K) spectrum for **4b**

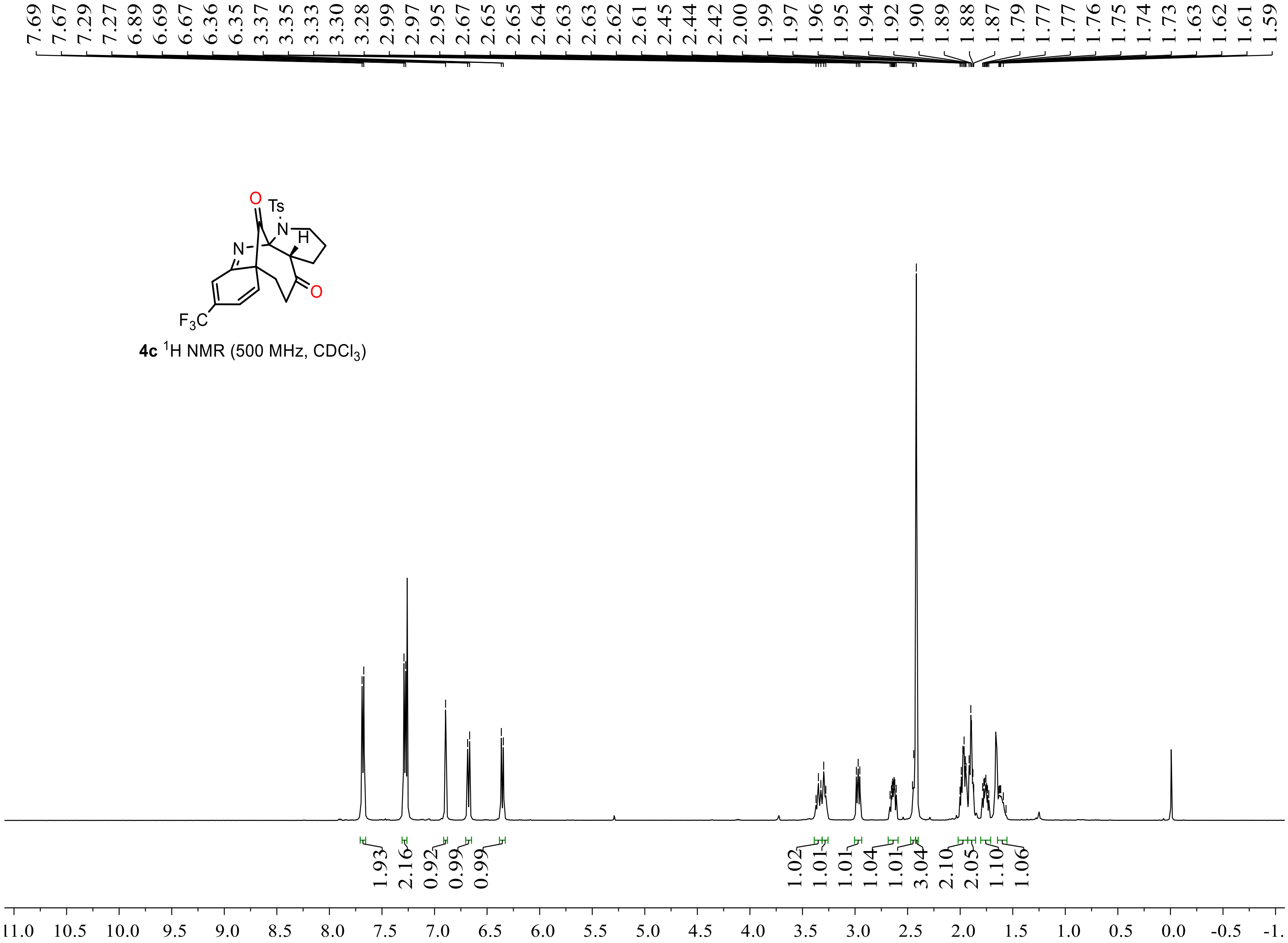

Supplementary Figure 178. ^{1}H NMR ($CDCl_3$, 500 MHz, 298 K) spectrum for **4c**

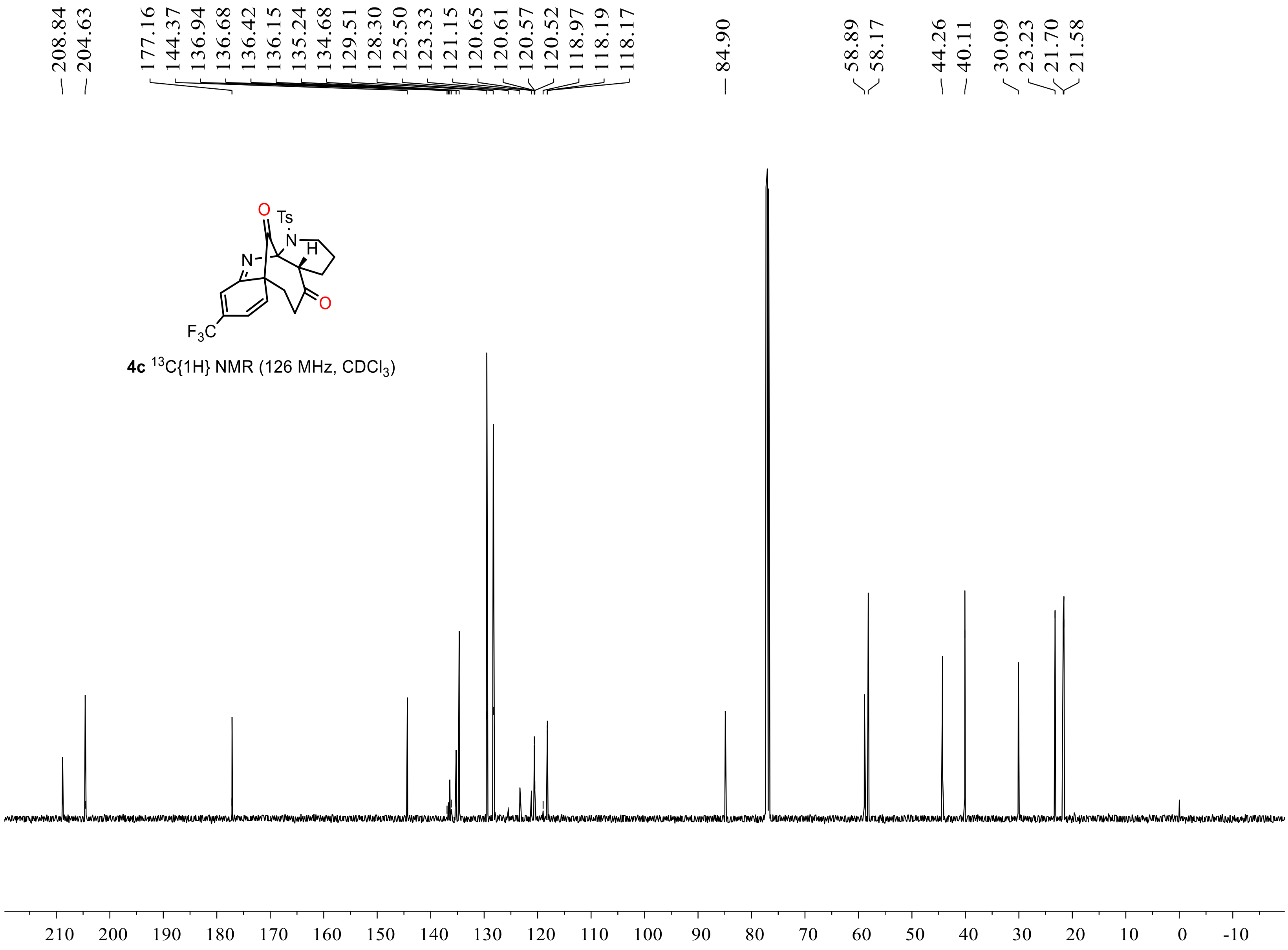

Supplementary Figure 179. ^{13}C NMR ($CDCl_3$, 126 MHz, 298 K) spectrum for **4c**

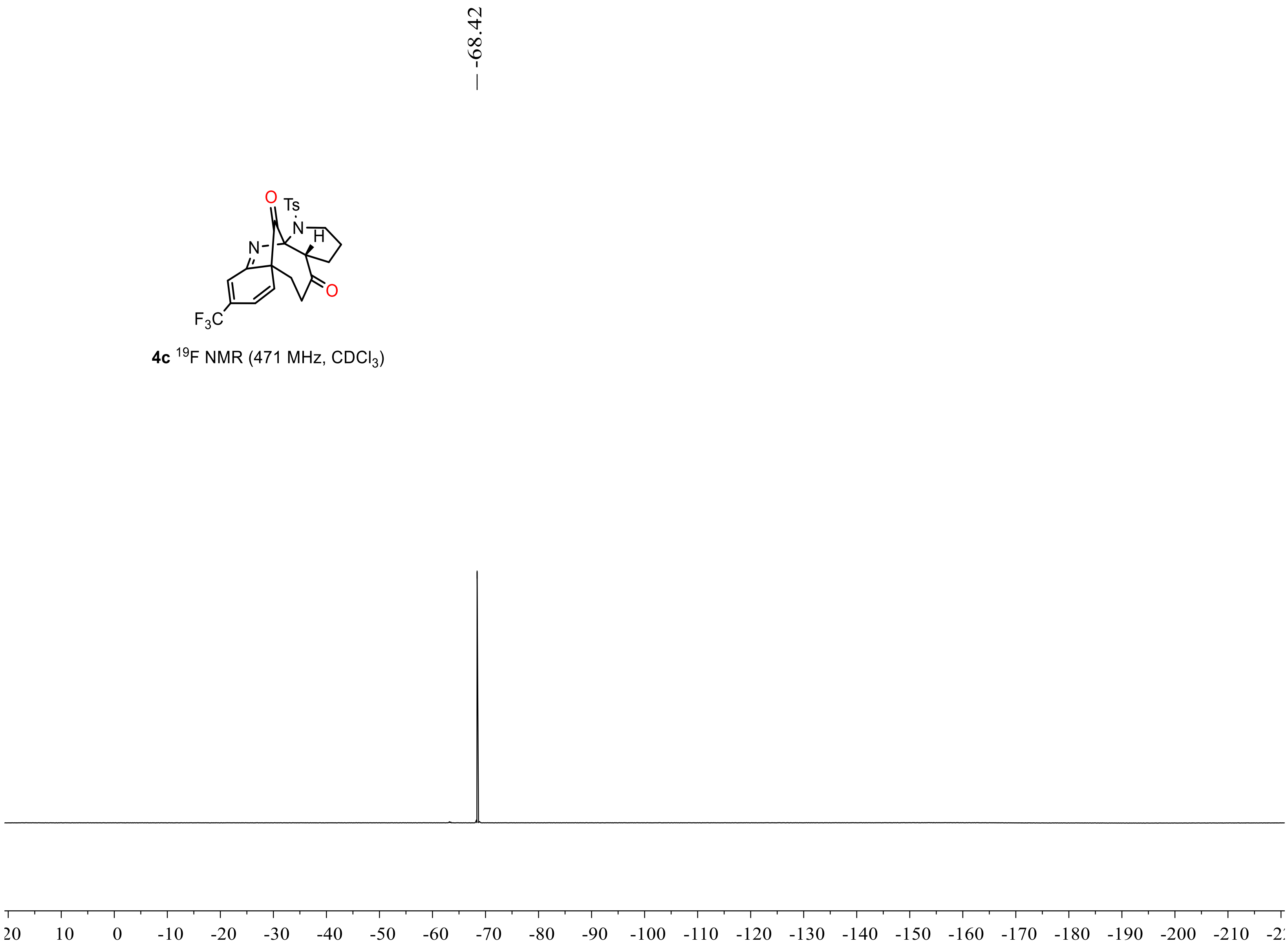

Supplementary Figure 180. ^{19}F NMR ($CDCl_3$, 471 MHz, 298 K) spectrum for **4c**

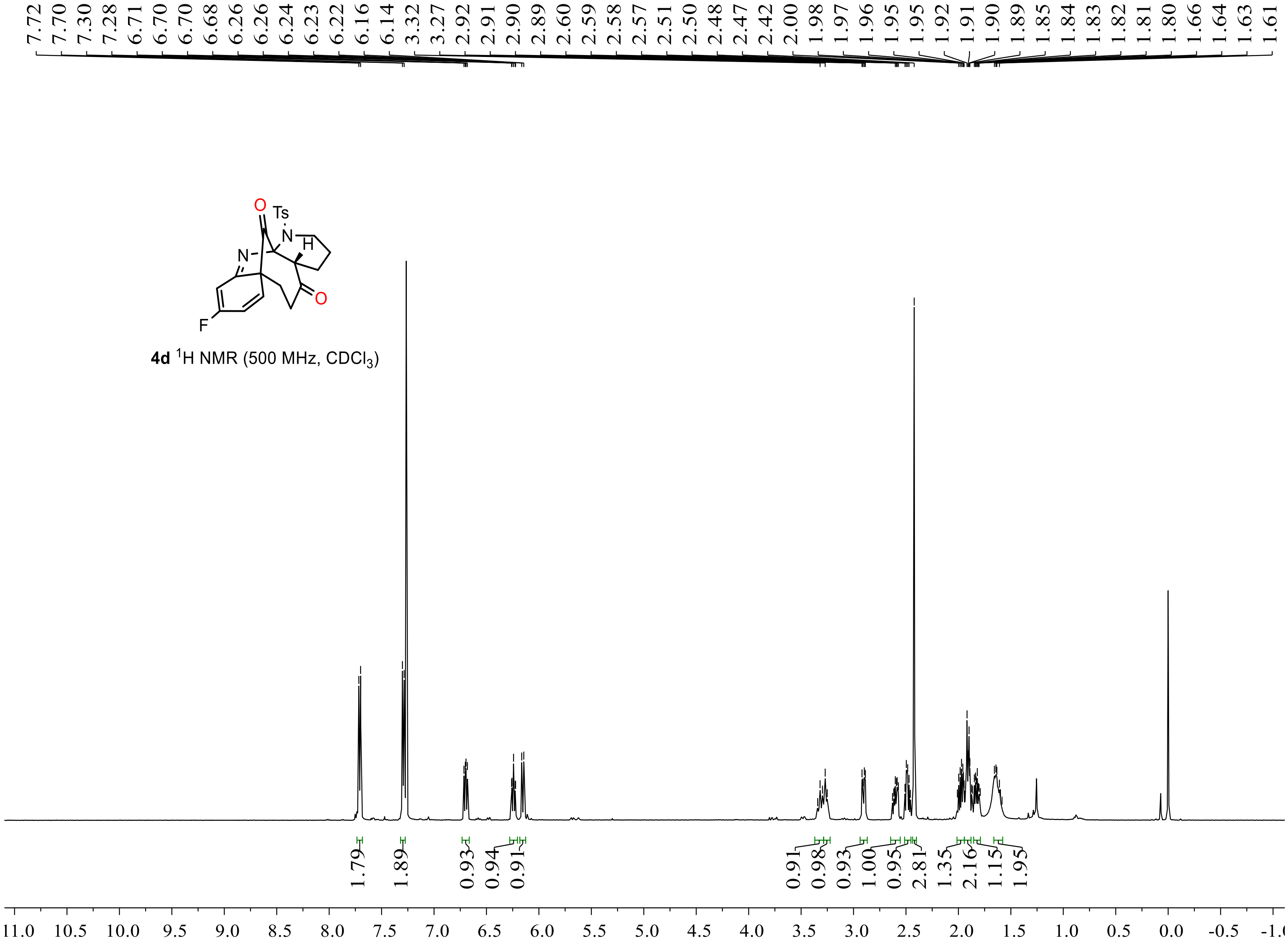

Supplementary Figure 181. ^{1}H NMR (CDCl$_3$, 500 MHz, 298 K) spectrum for **4d**

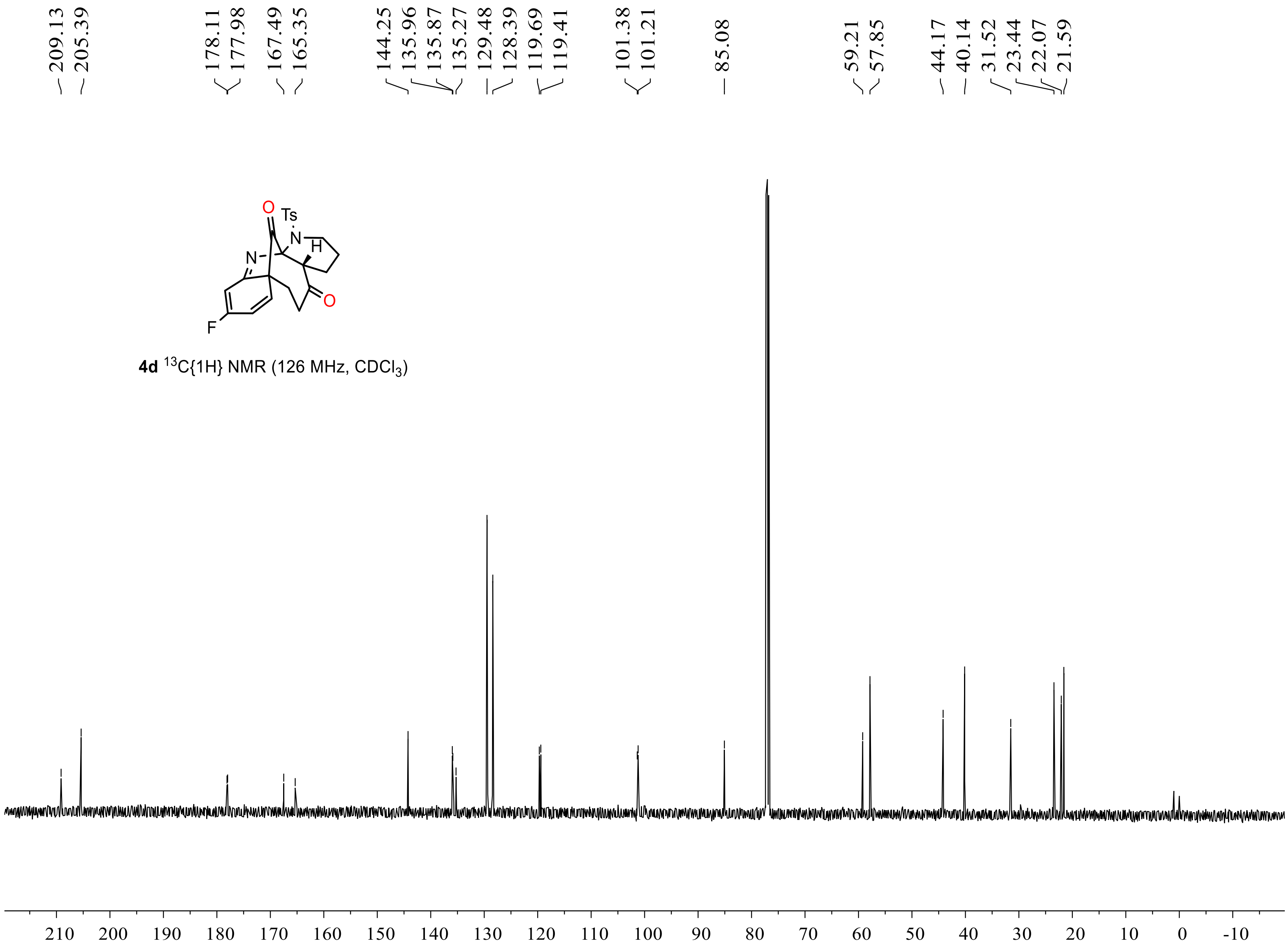

Supplementary Figure 182. ^{13}C NMR ($CDCl_3$, 126 MHz, 298 K) spectrum for **4d**

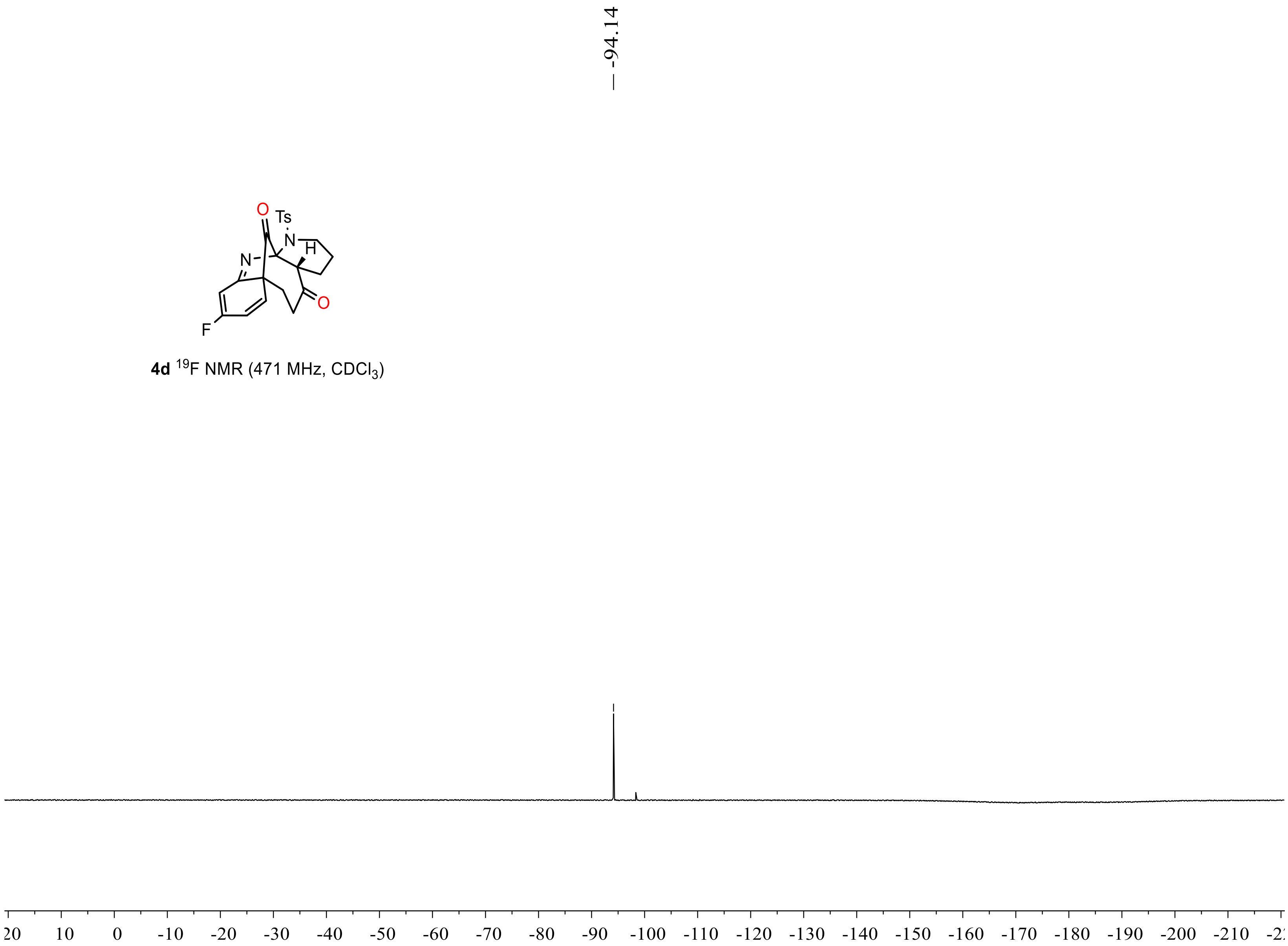

Supplementary Figure 183. ^{19}F NMR ($CDCl_3$, 471 MHz, 298 K) spectrum for **4d**

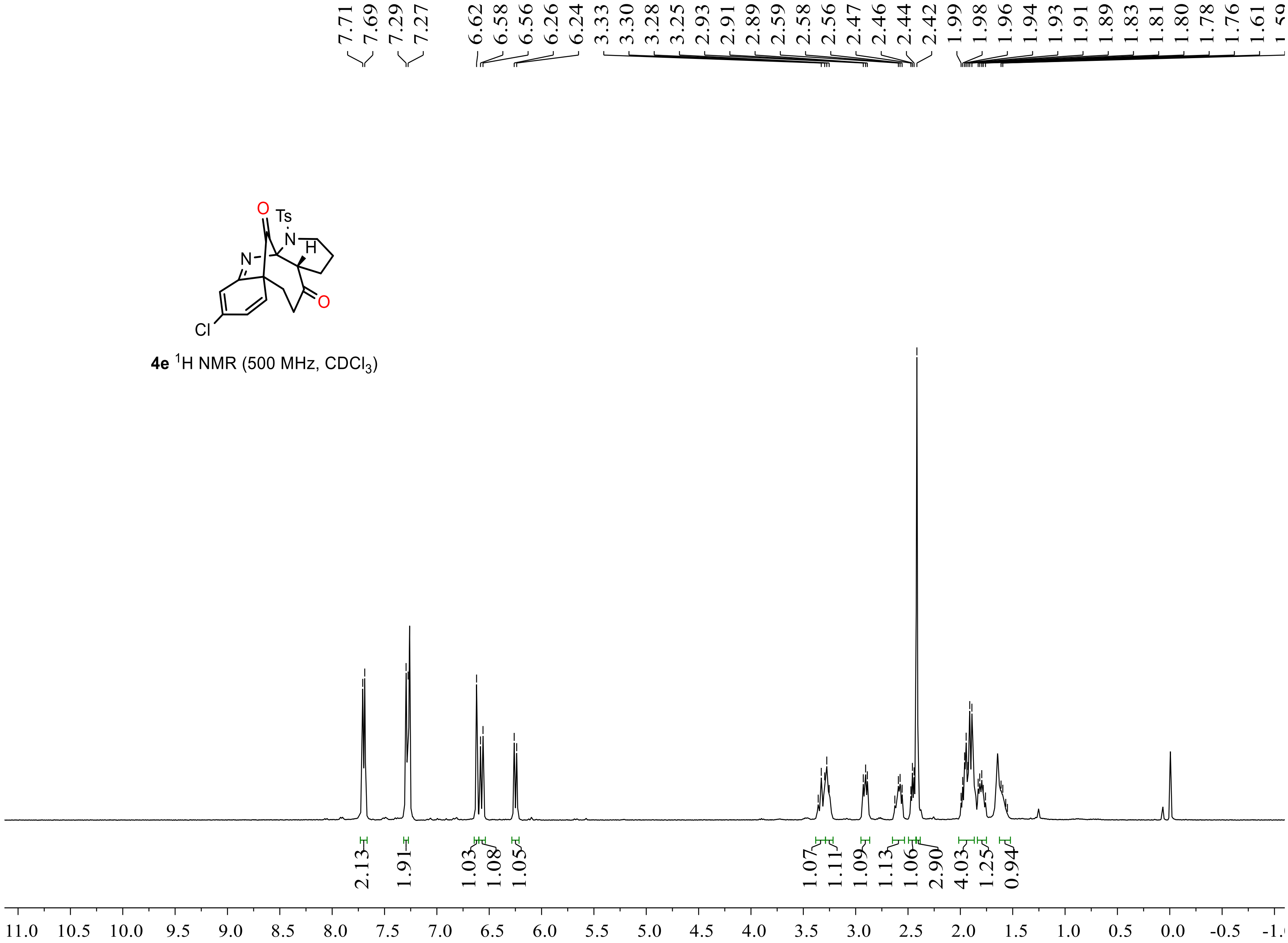

Supplementary Figure 184. ^{1}H NMR ($CDCl_3$, 500 MHz, 298 K) spectrum for **4e**

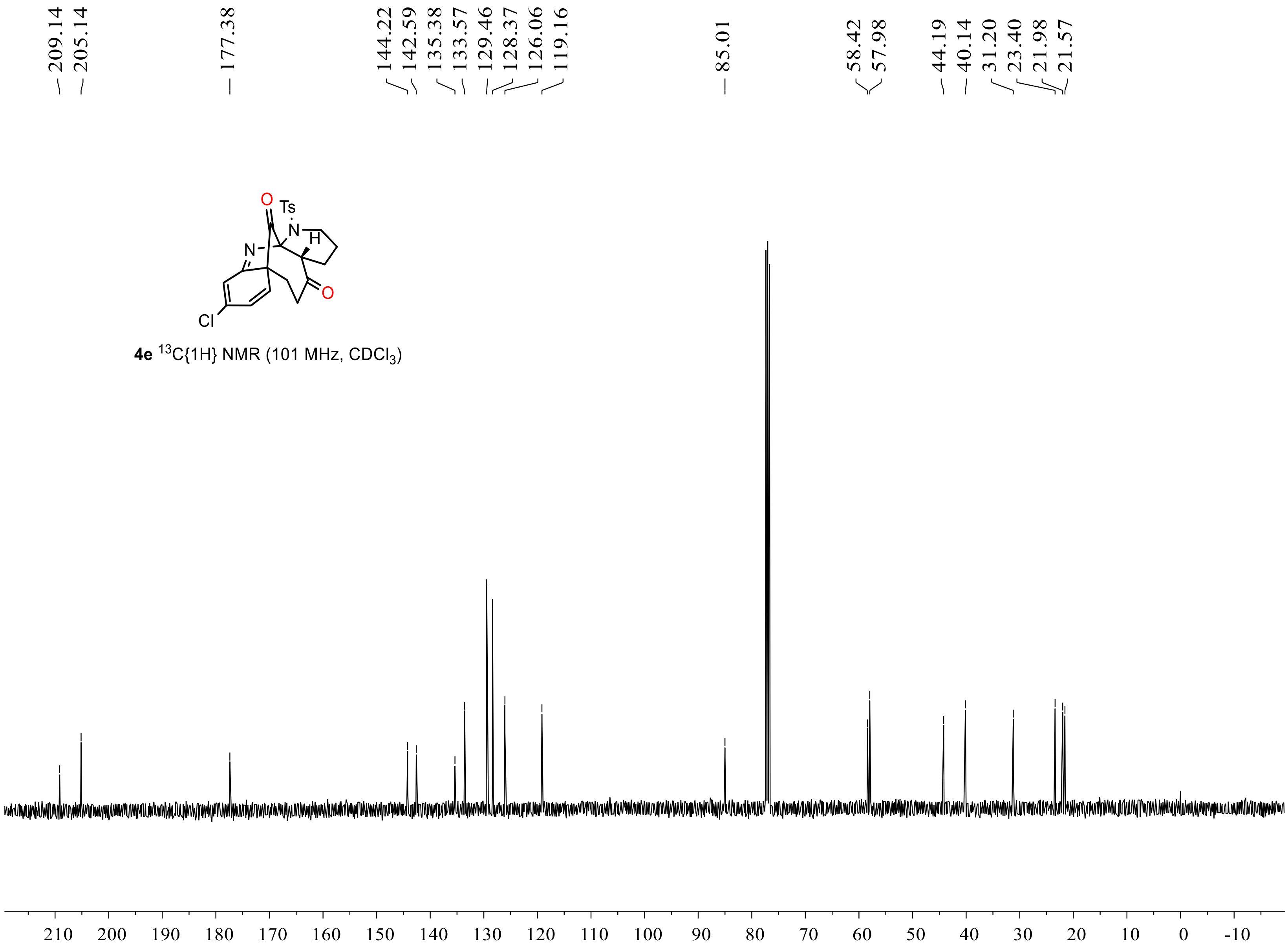

Supplementary Figure 185. ^{13}C NMR ($CDCl_3$, 126 MHz, 298 K) spectrum for **4e**

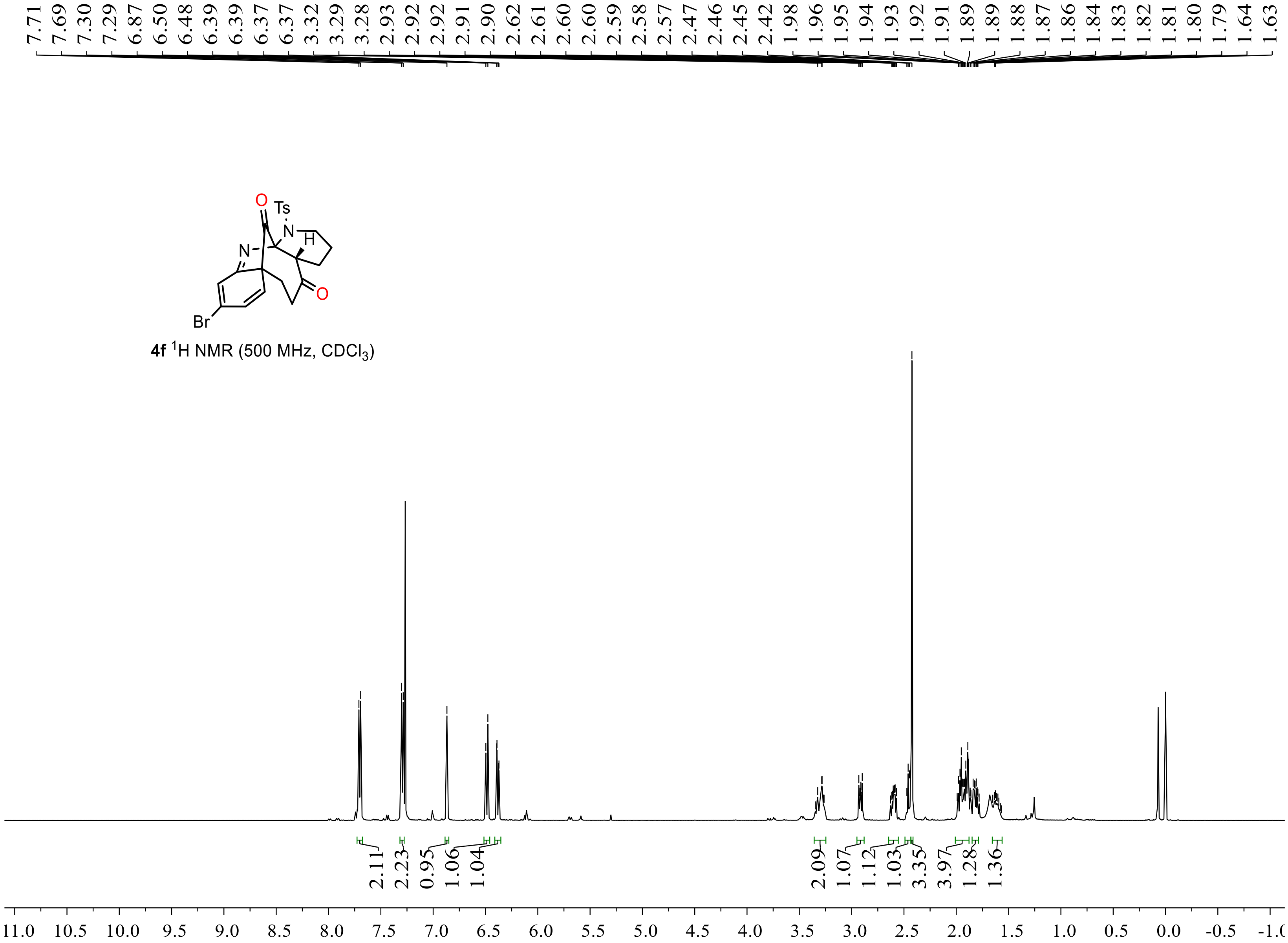

Supplementary Figure 186. ^{1}H NMR ($CDCl_3$, 500 MHz, 298 K) spectrum for **4f**

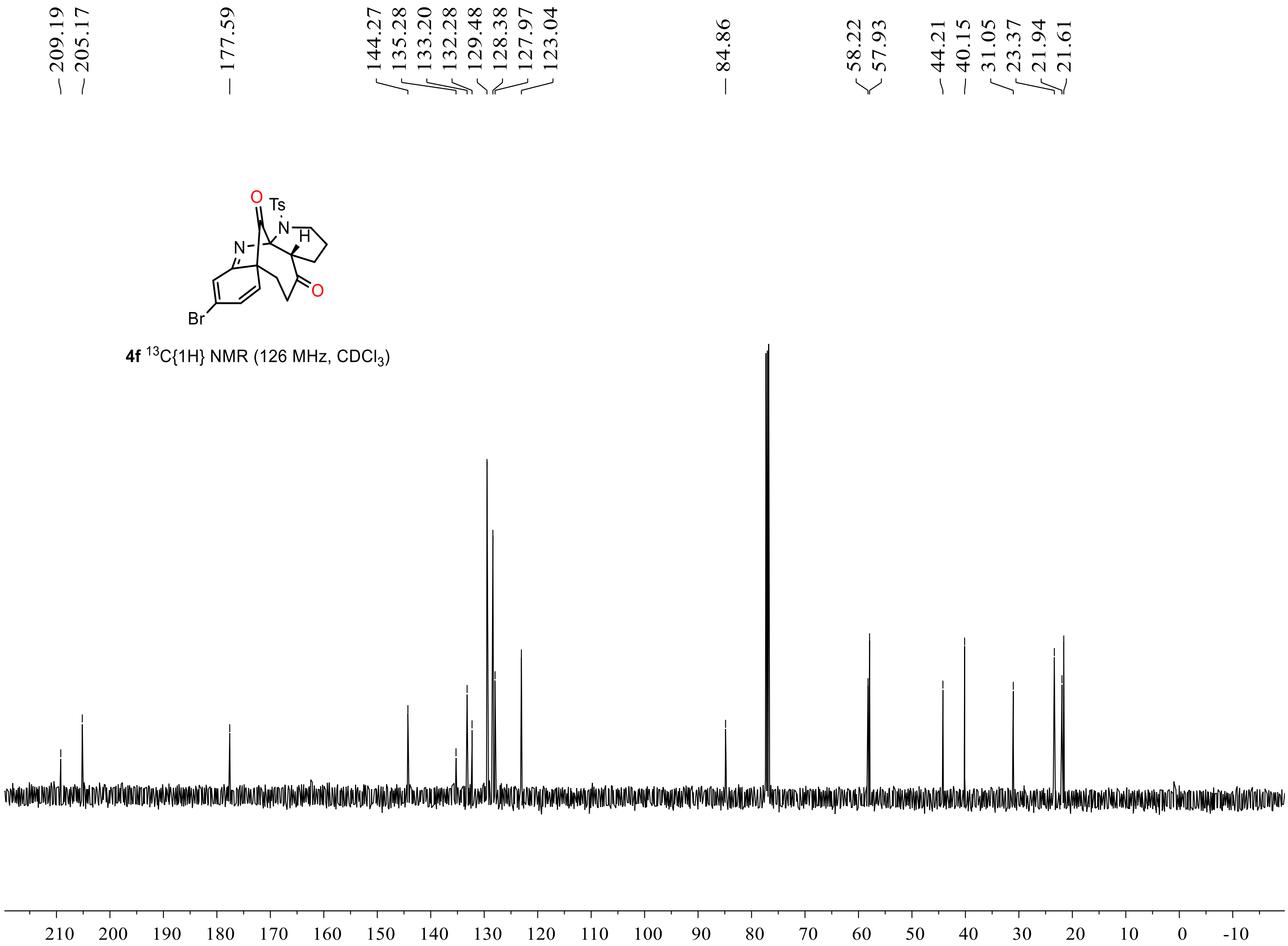

Supplementary Figure 187. ^{13}C NMR ($CDCl_3$, 126 MHz, 298 K) spectrum for **4f**

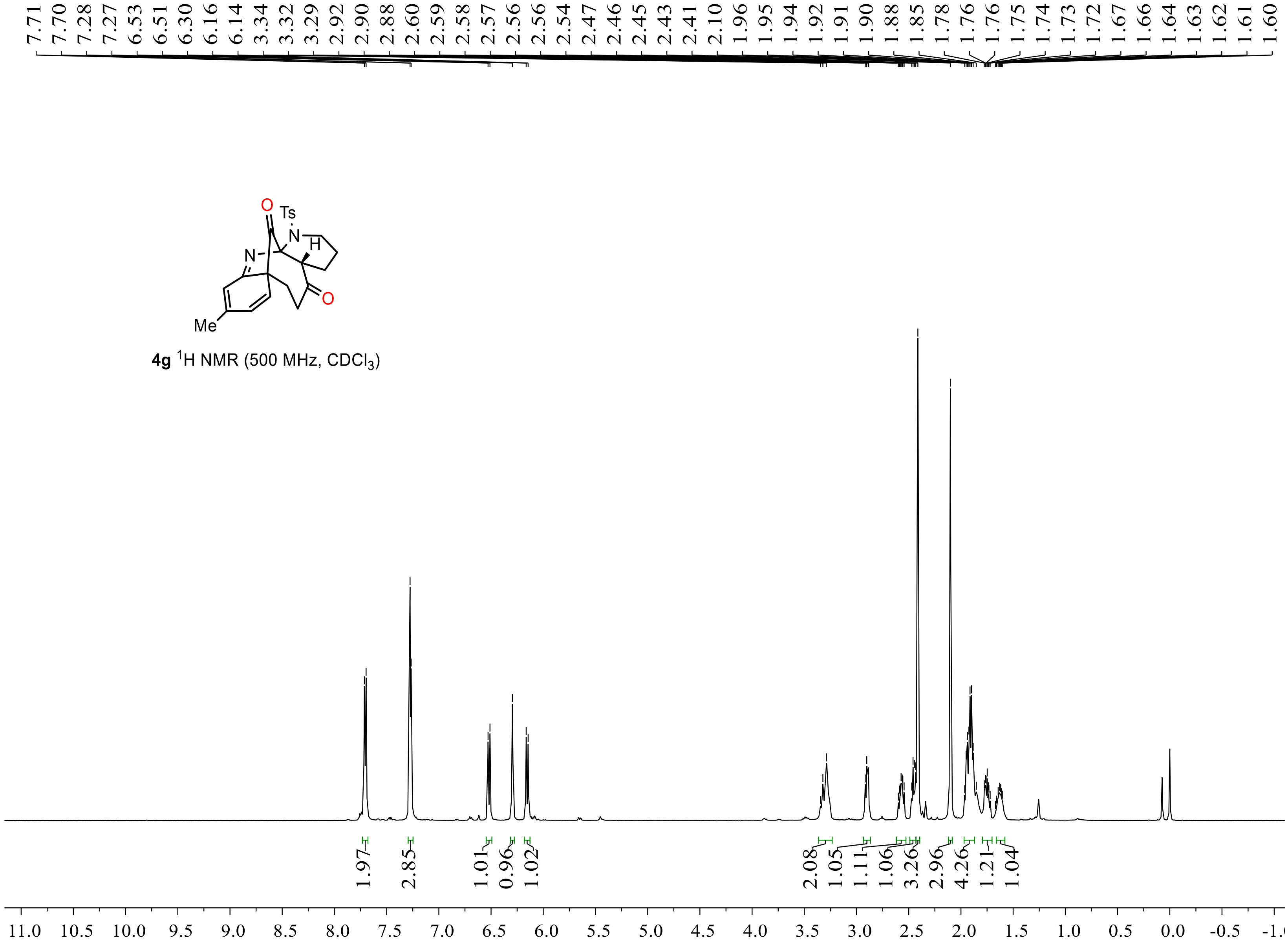

Supplementary Figure 188. ^{1}H NMR ($CDCl_3$, 500 MHz, 298 K) spectrum for **4g**

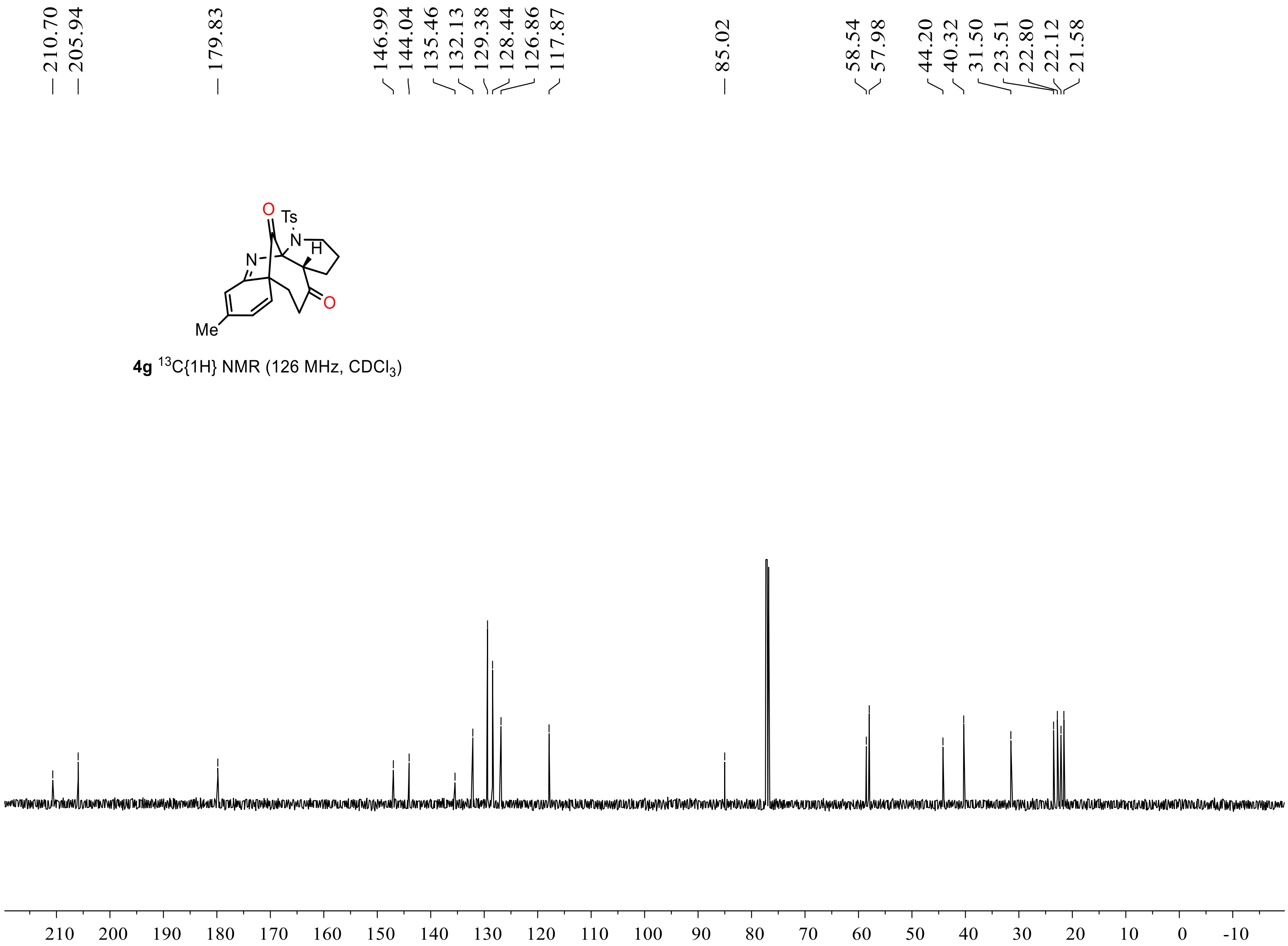

Supplementary Figure 189. ^{13}C NMR ($CDCl_3$, 126 MHz, 298 K) spectrum for **4g**

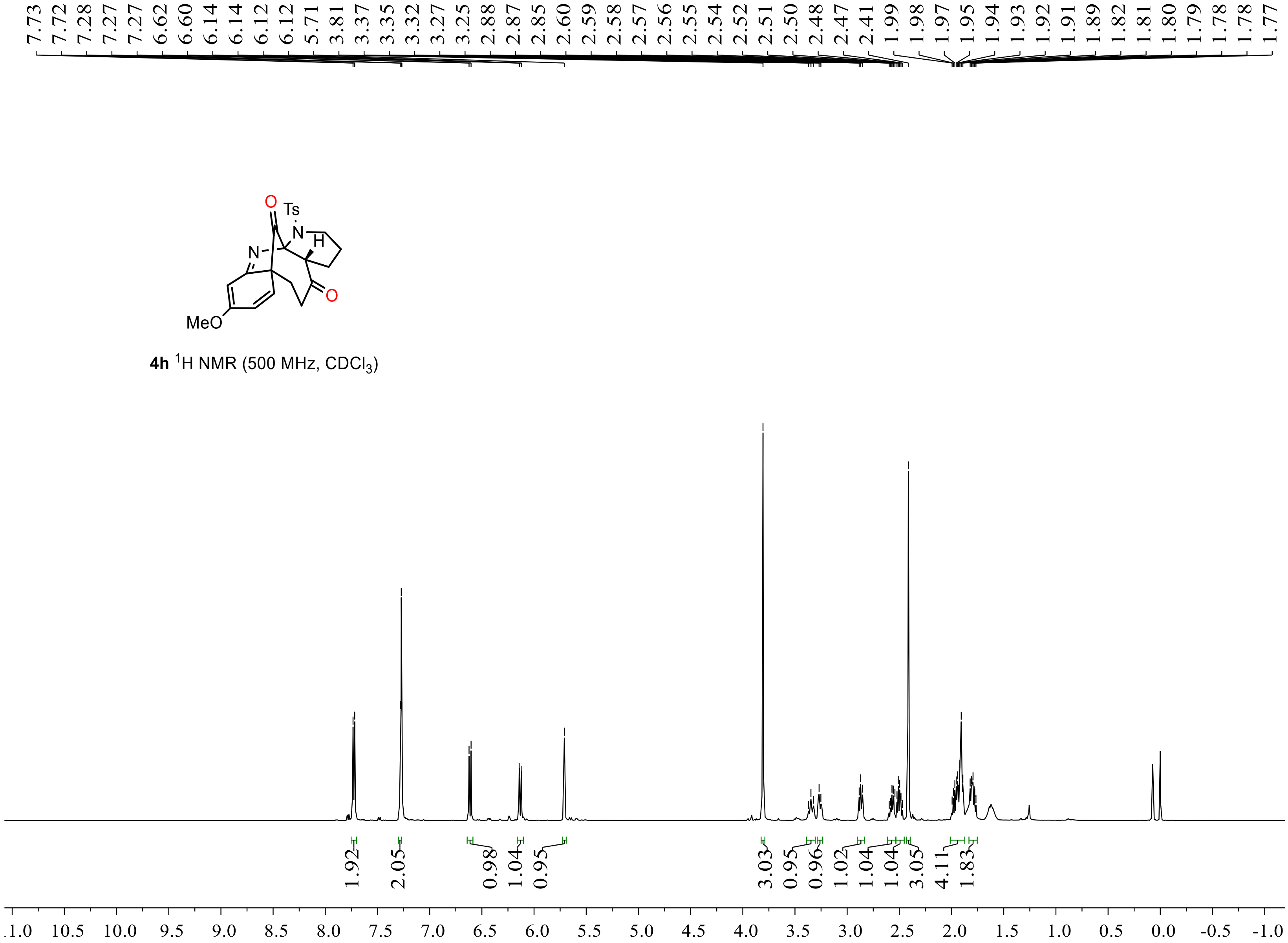

Supplementary Figure 190. ^{1}H NMR ($CDCl_3$, 500 MHz, 298 K) spectrum for **4h**

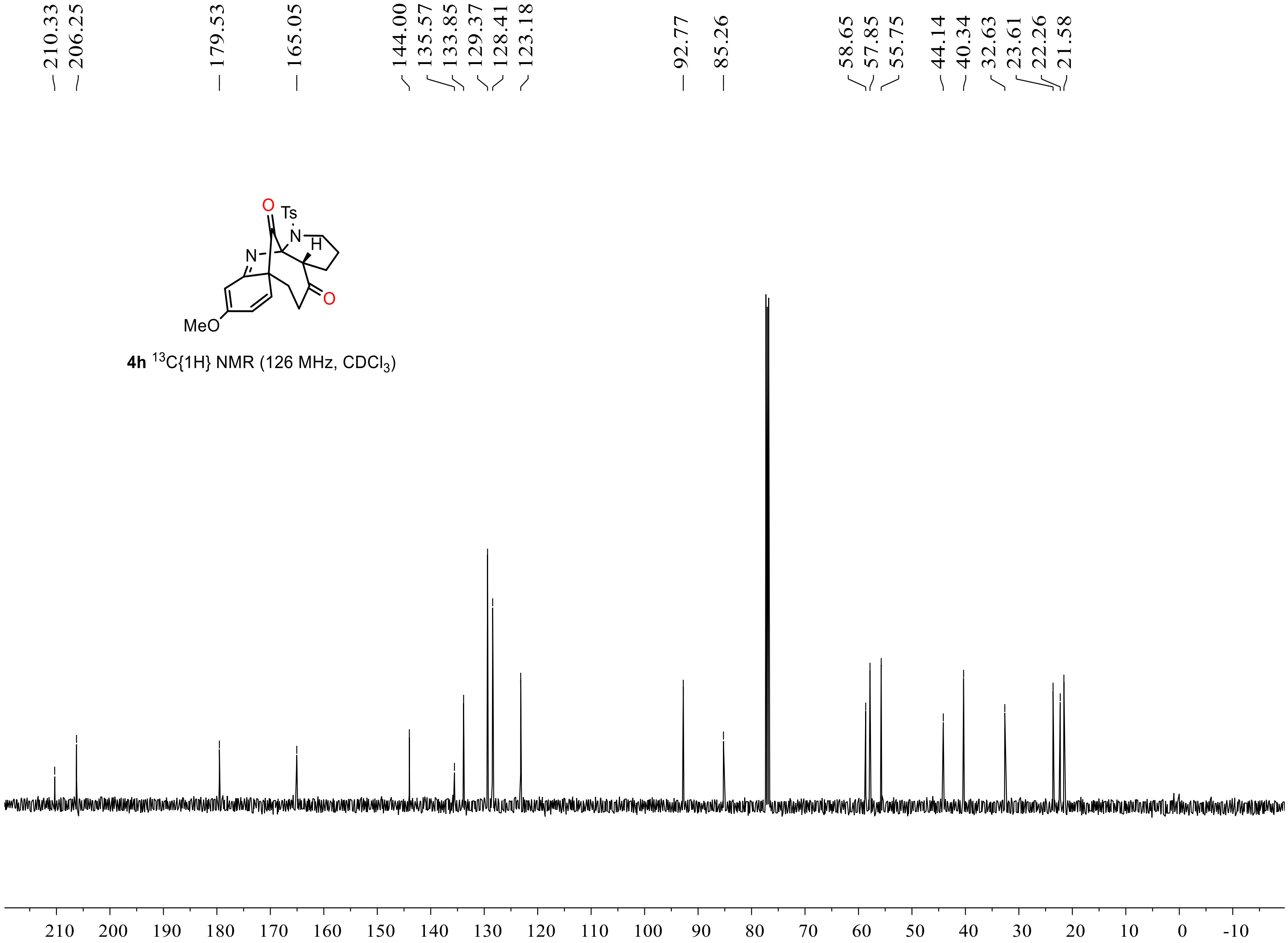

Supplementary Figure 191. ^{13}C NMR ($CDCl_3$, 126 MHz, 298 K) spectrum for **4h**

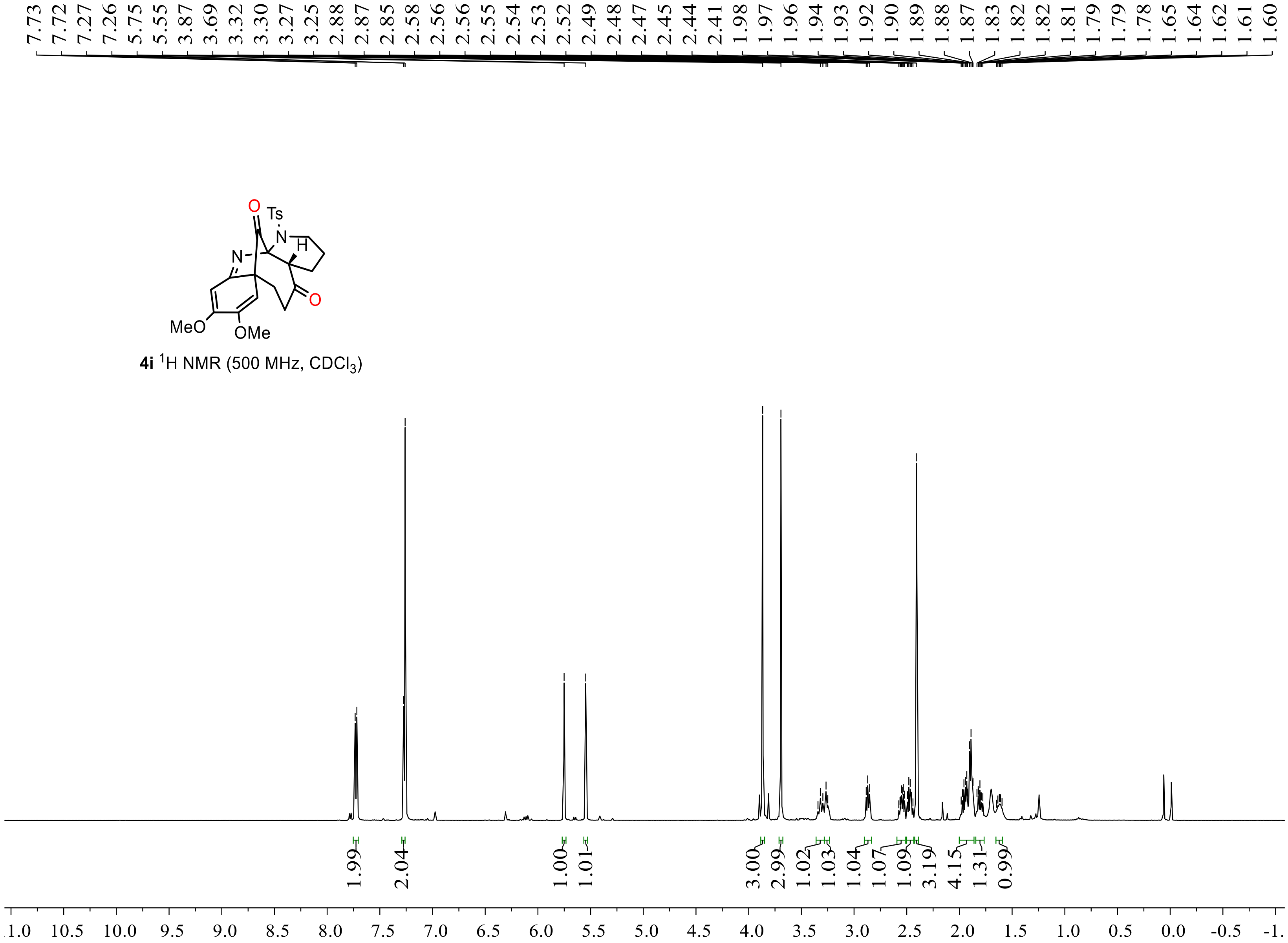

Supplementary Figure 192. ^{1}H NMR ($CDCl_3$, 500 MHz, 298 K) spectrum for **4i**

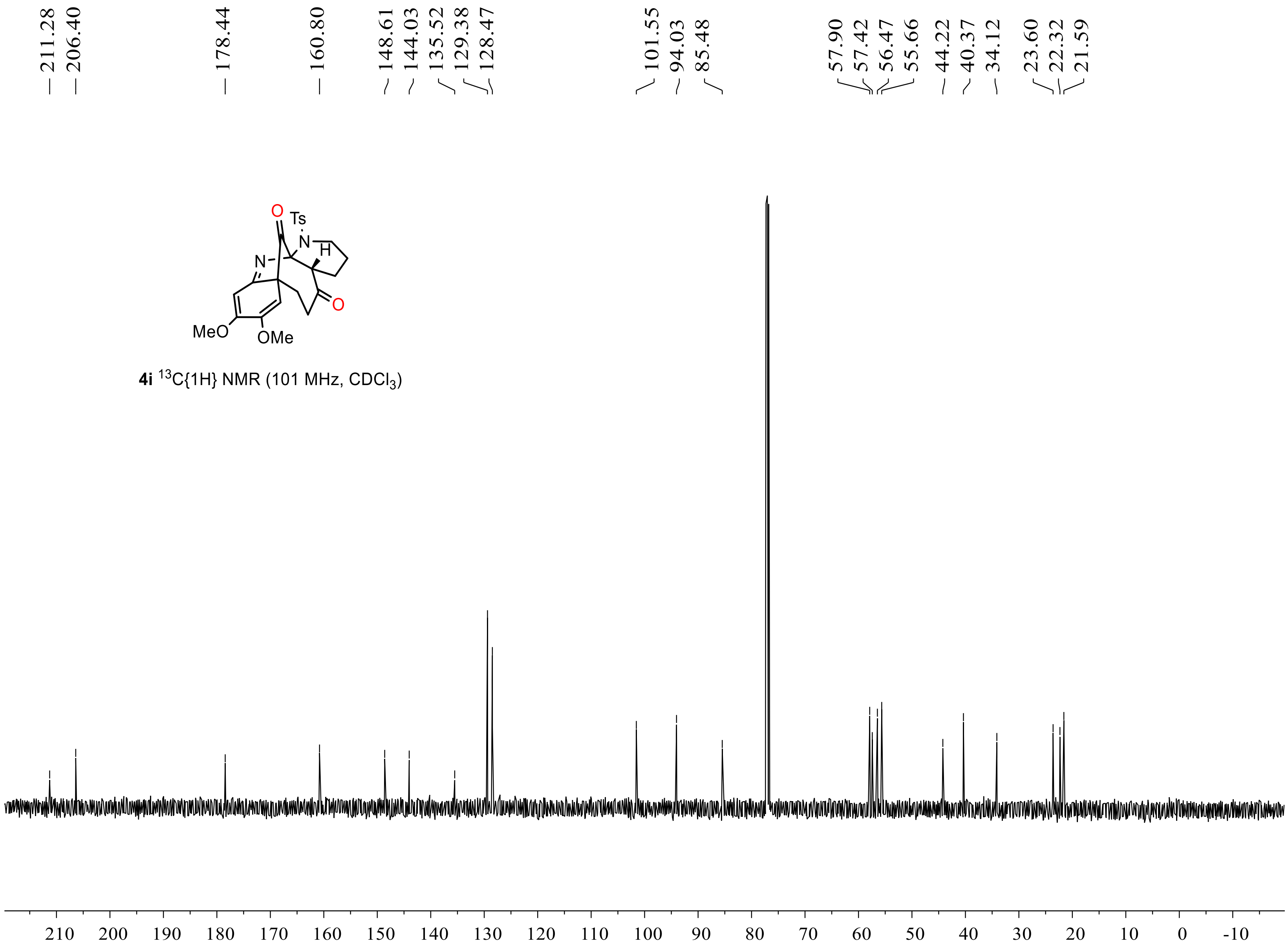

Supplementary Figure 193. ^{13}C NMR ($CDCl_3$, 126 MHz, 298 K) spectrum for **4i**

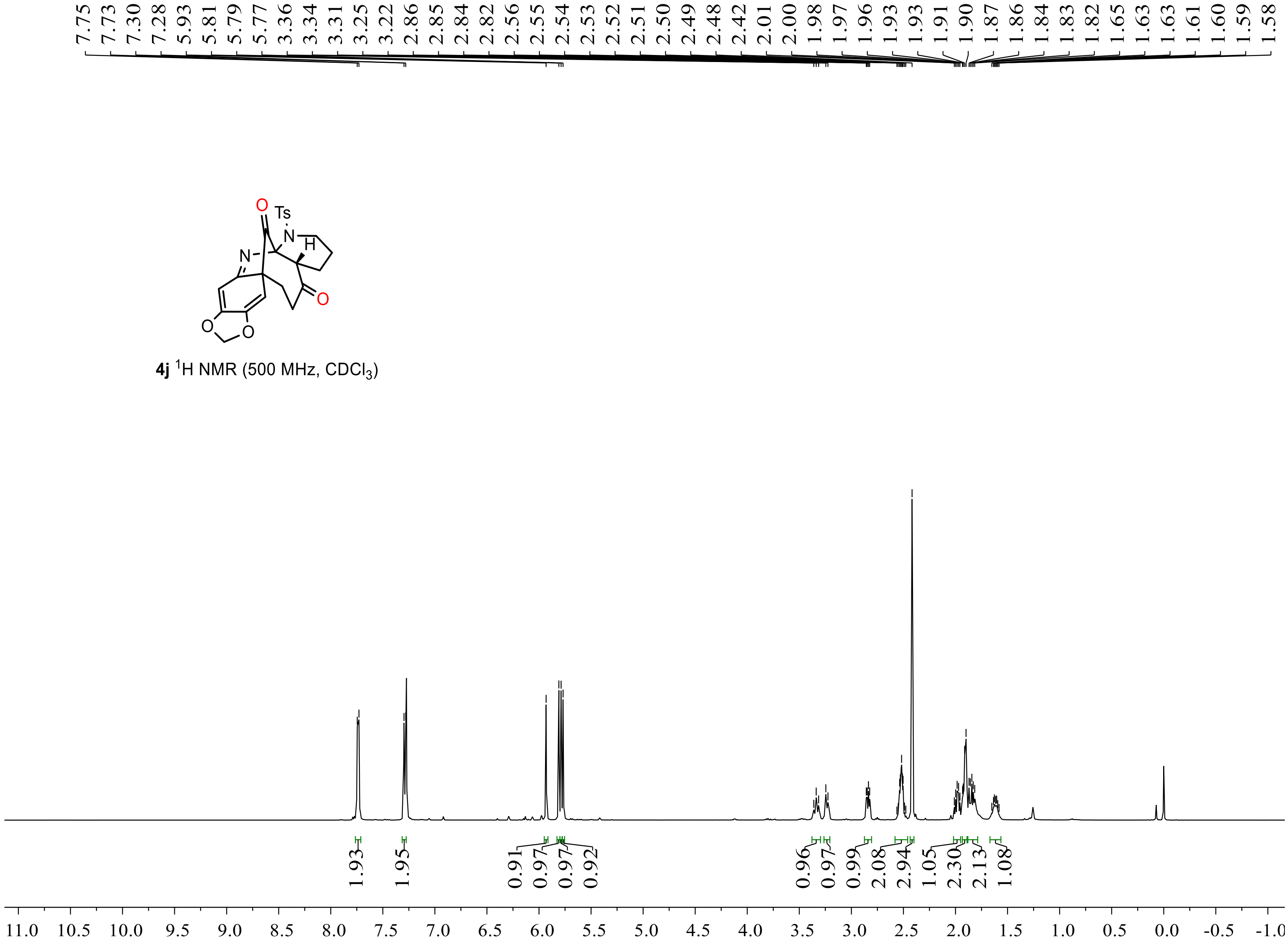

Supplementary Figure 194. ^{1}H NMR ($CDCl_3$, 500 MHz, 298 K) spectrum for **4j**

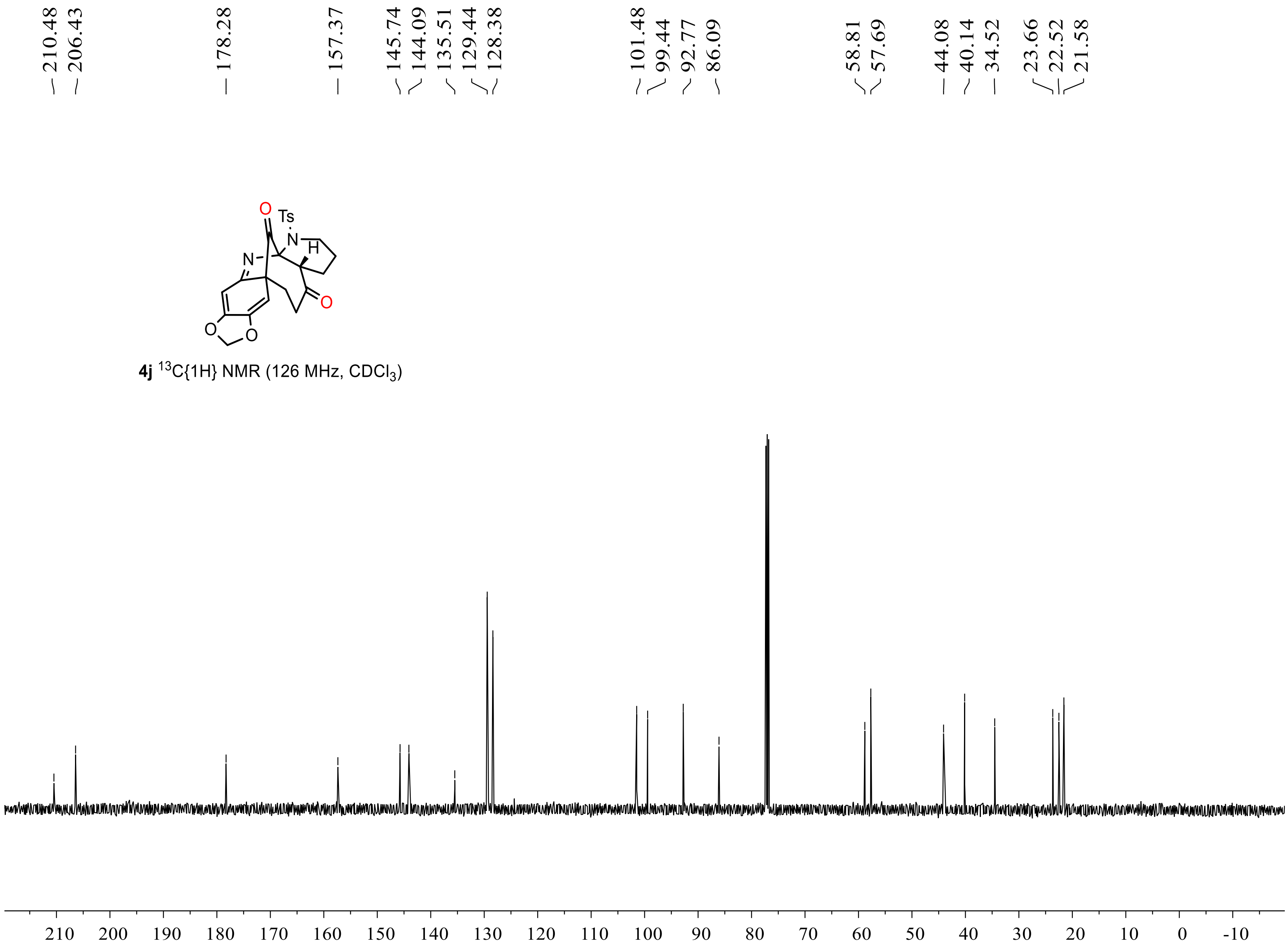

Supplementary Figure 195. ^{13}C NMR ($CDCl_3$, 126 MHz, 298 K) spectrum for **4j**

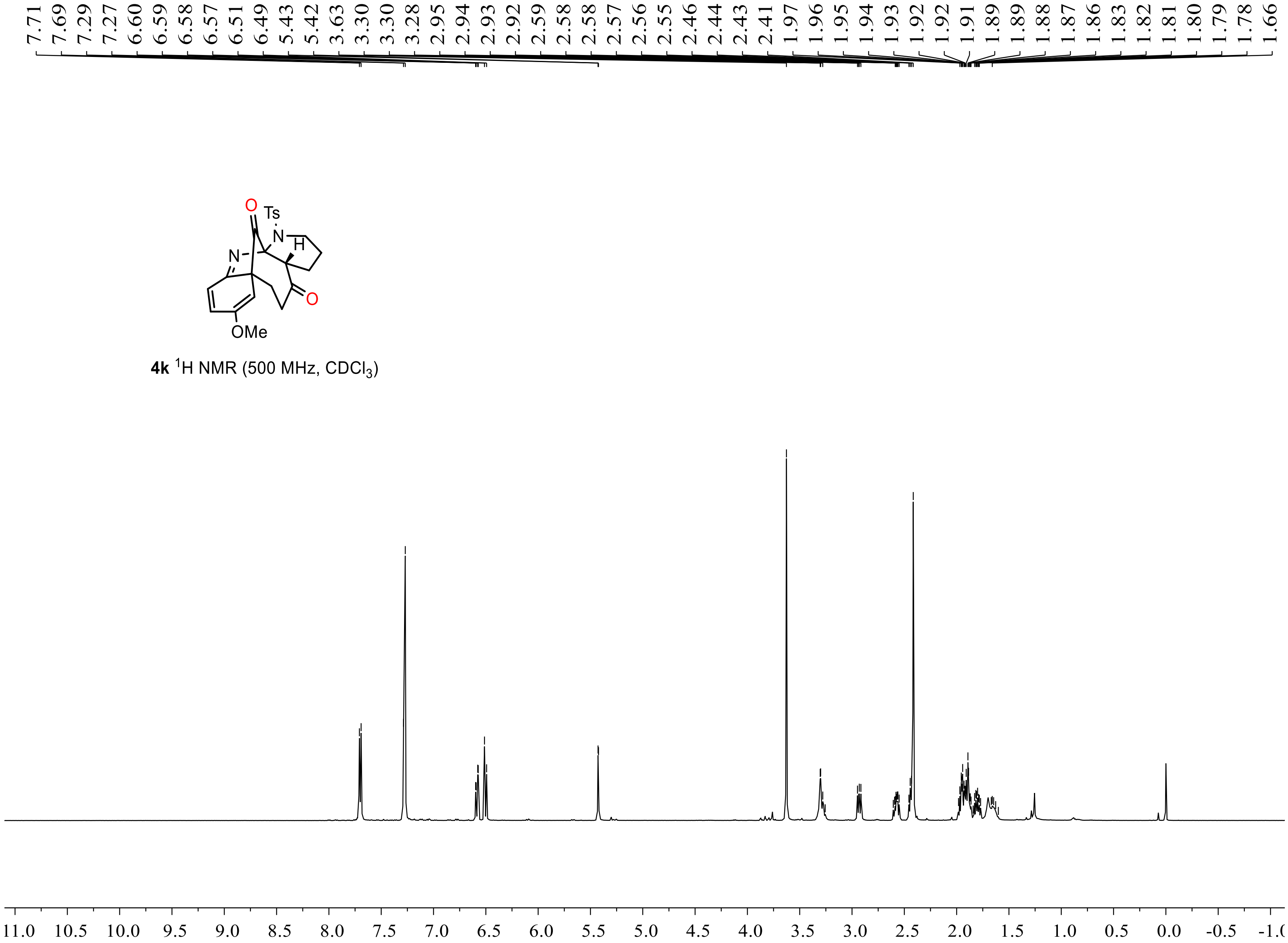

Supplementary Figure 196. ^{1}H NMR ($CDCl_3$, 500 MHz, 298 K) spectrum for **4k**

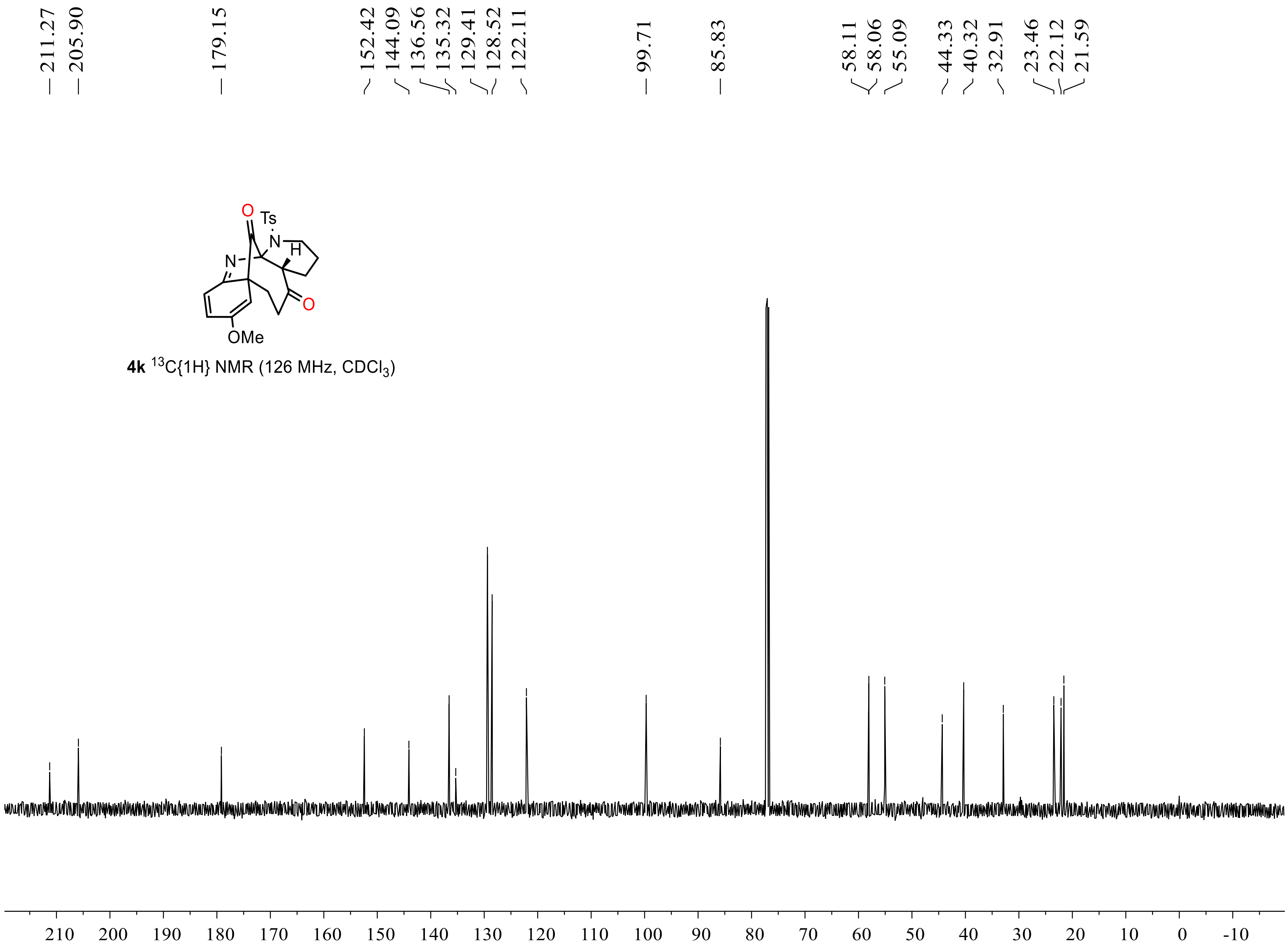

Supplementary Figure 197. ^{13}C NMR ($CDCl_3$, 126 MHz, 298 K) spectrum for **4k**

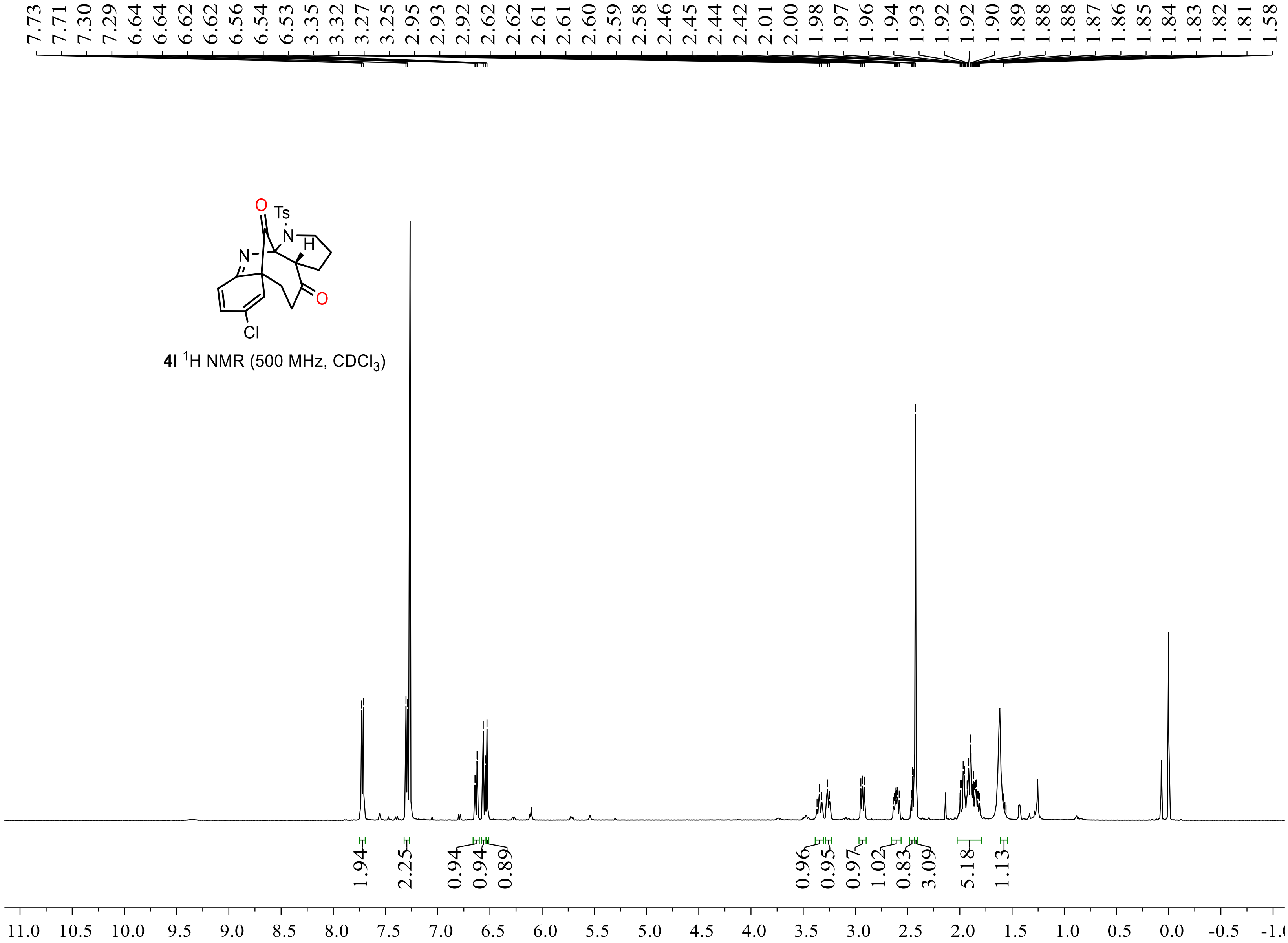

Supplementary Figure 198. ^{1}H NMR ($CDCl_3$, 500 MHz, 298 K) spectrum for **4l**

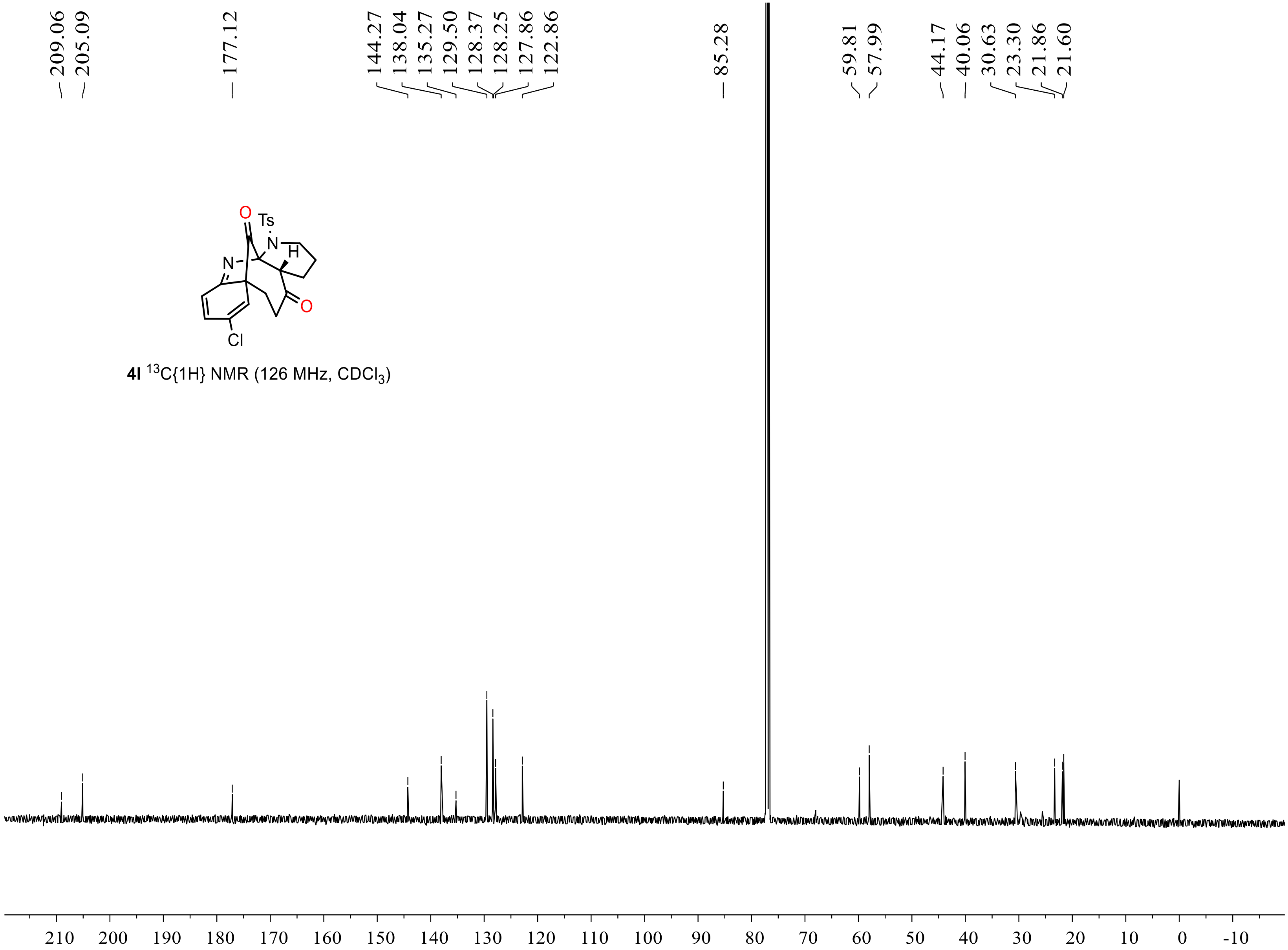

Supplementary Figure 199. ^{13}C NMR ($CDCl_3$, 126 MHz, 298 K) spectrum for **4l**

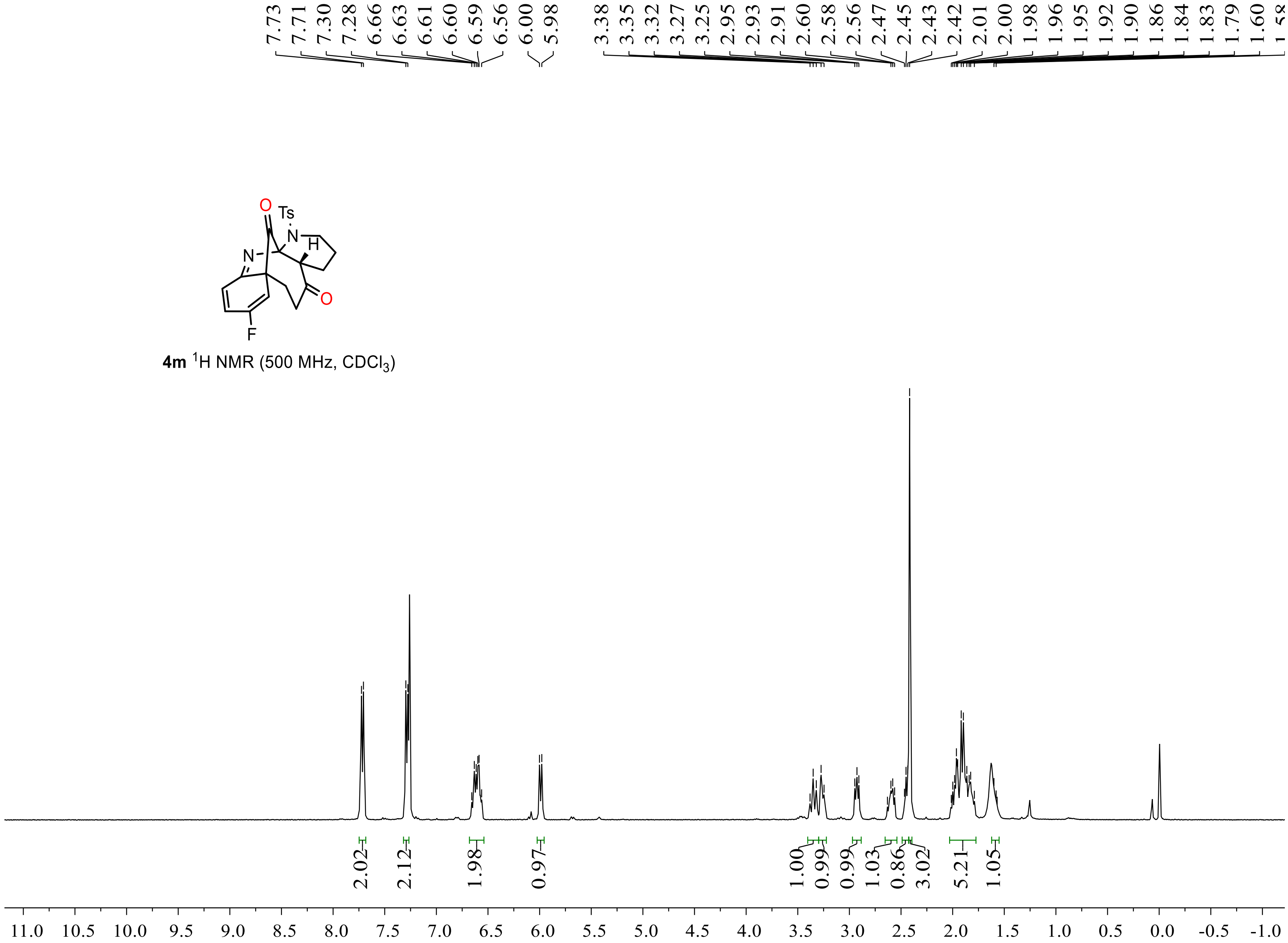

Supplementary Figure 200. ^{1}H NMR ($CDCl_3$, 500 MHz, 298 K) spectrum for **4m**

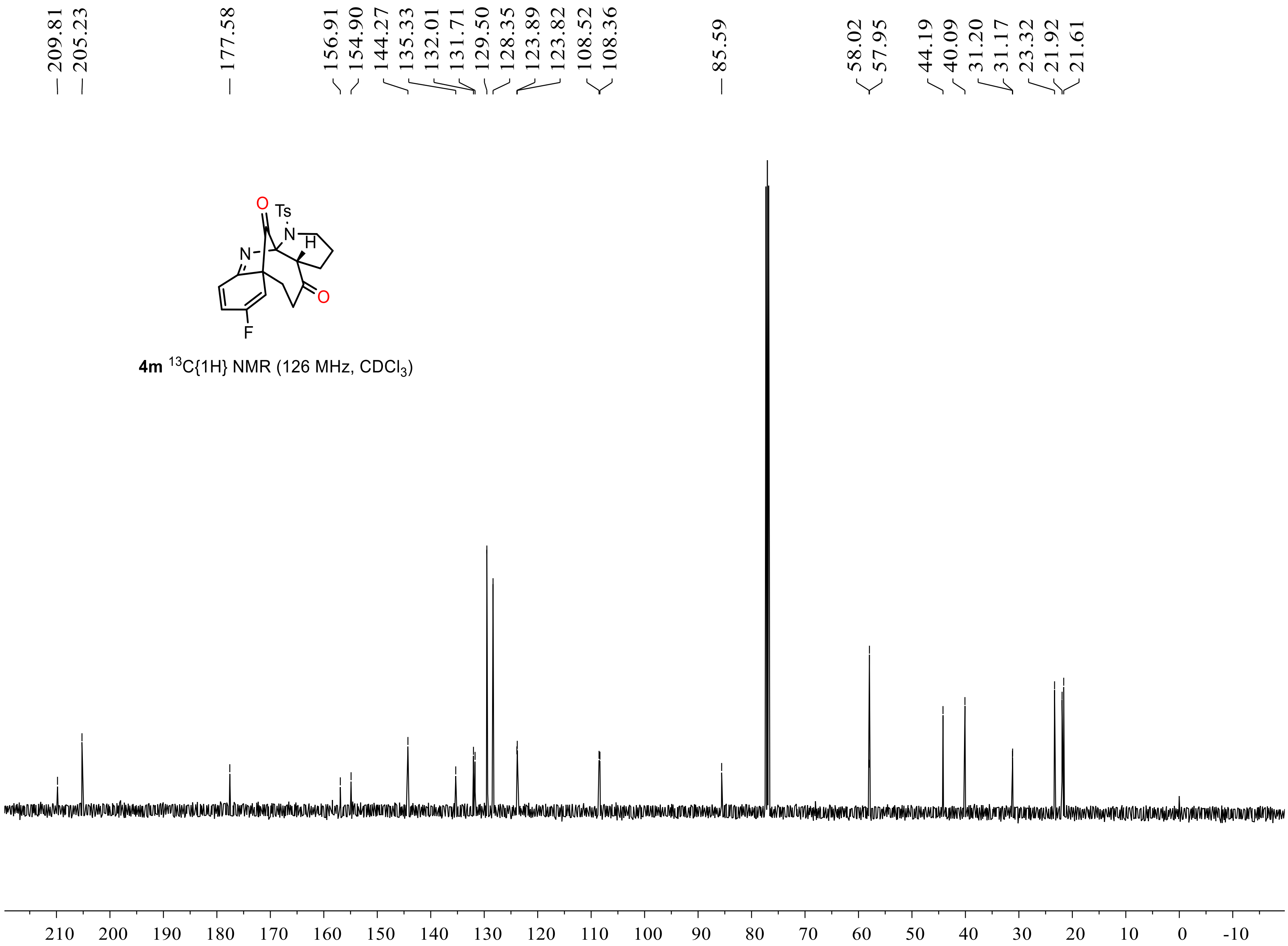

Supplementary Figure 201. ^{13}C NMR ($CDCl_3$, 126 MHz, 298 K) spectrum for **4m**

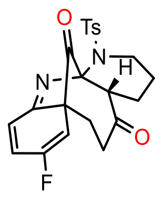

4m ^{19}F NMR (471 MHz, $CDCl_3$)

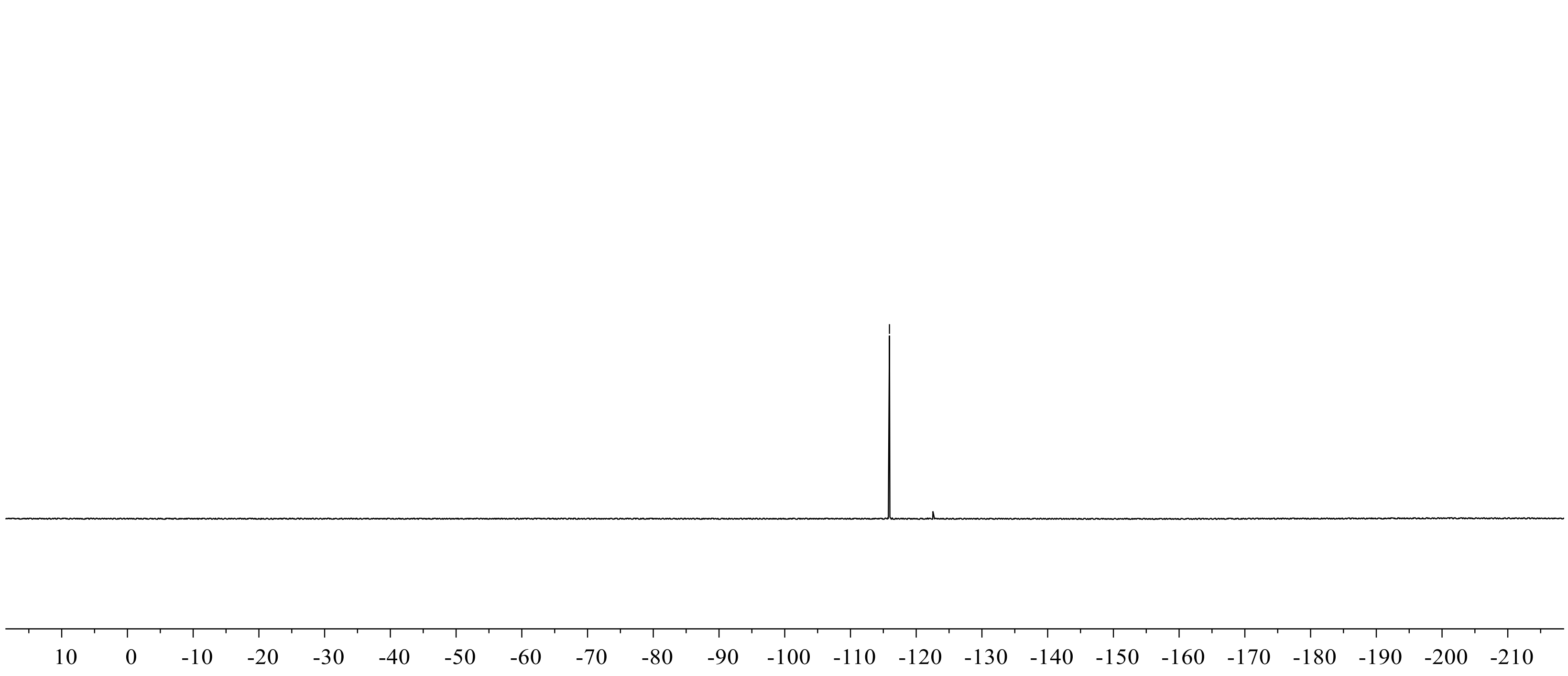

Supplementary Figure 202. ^{19}F NMR ($CDCl_3$, 471 MHz, 298 K) spectrum for **4m**

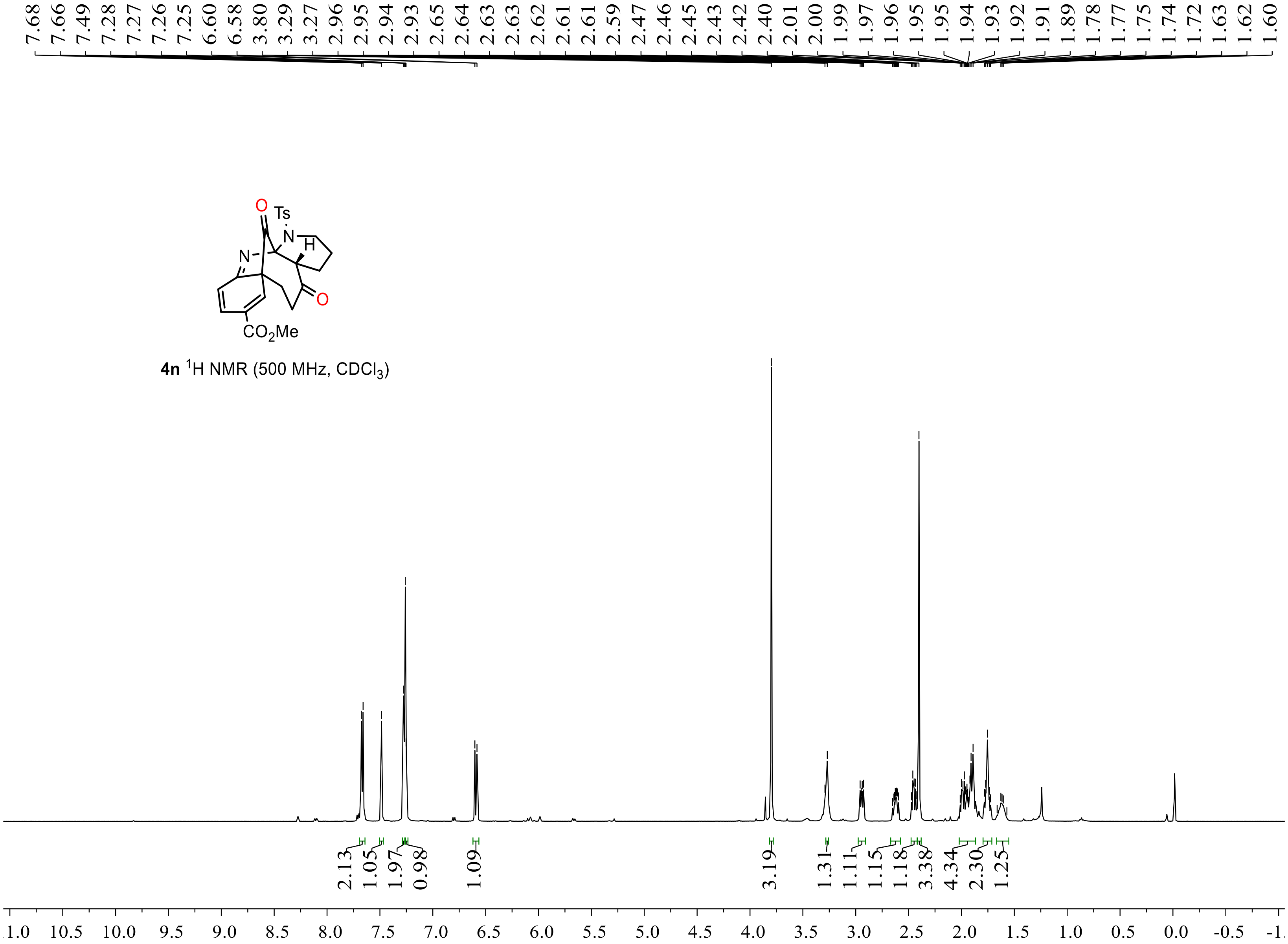

Supplementary Figure 203. ^{1}H NMR ($CDCl_3$, 500 MHz, 298 K) spectrum for **4n**

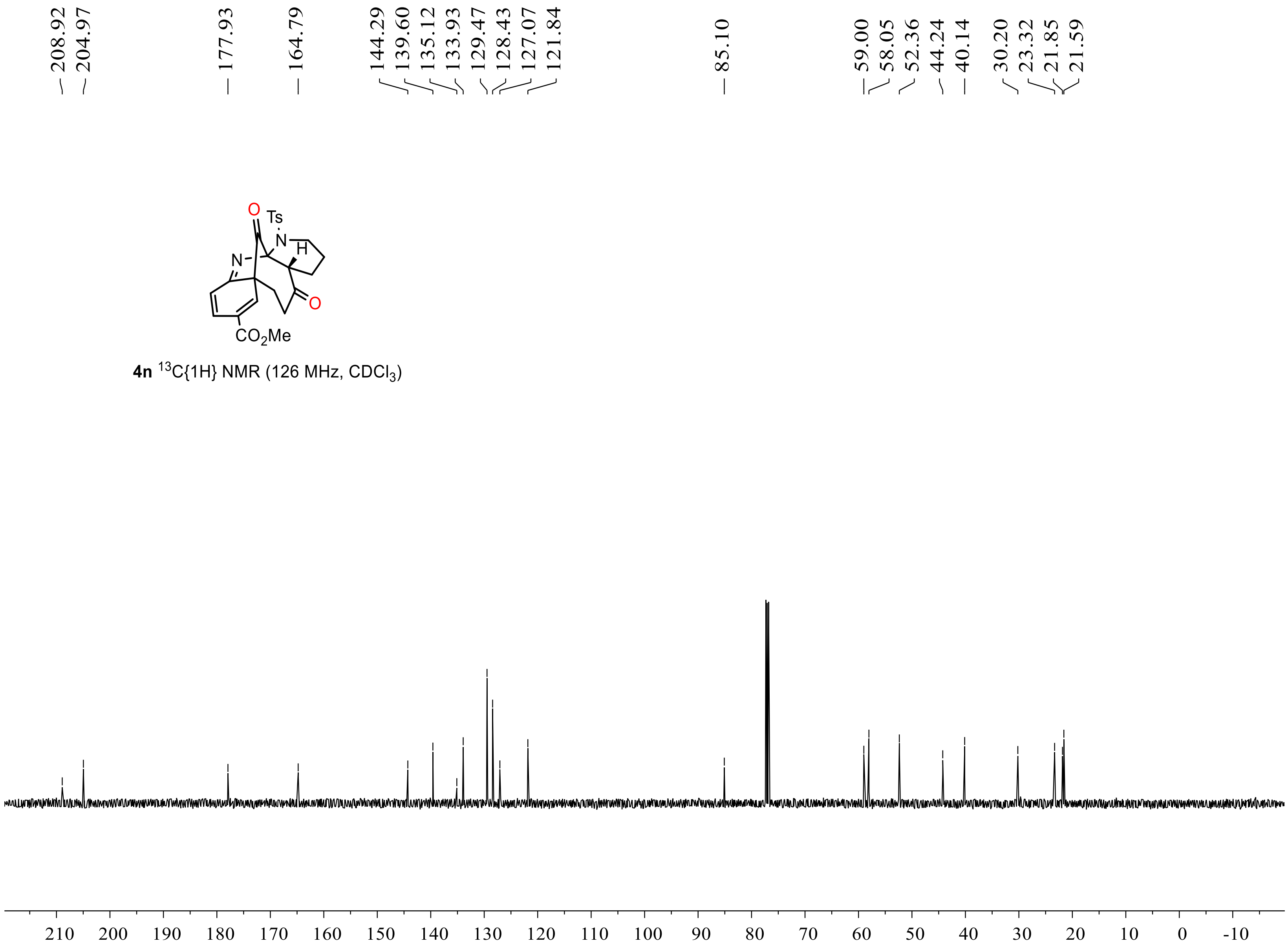

Supplementary Figure 204. ^{13}C NMR ($CDCl_3$, 126 MHz, 298 K) spectrum for **4n**

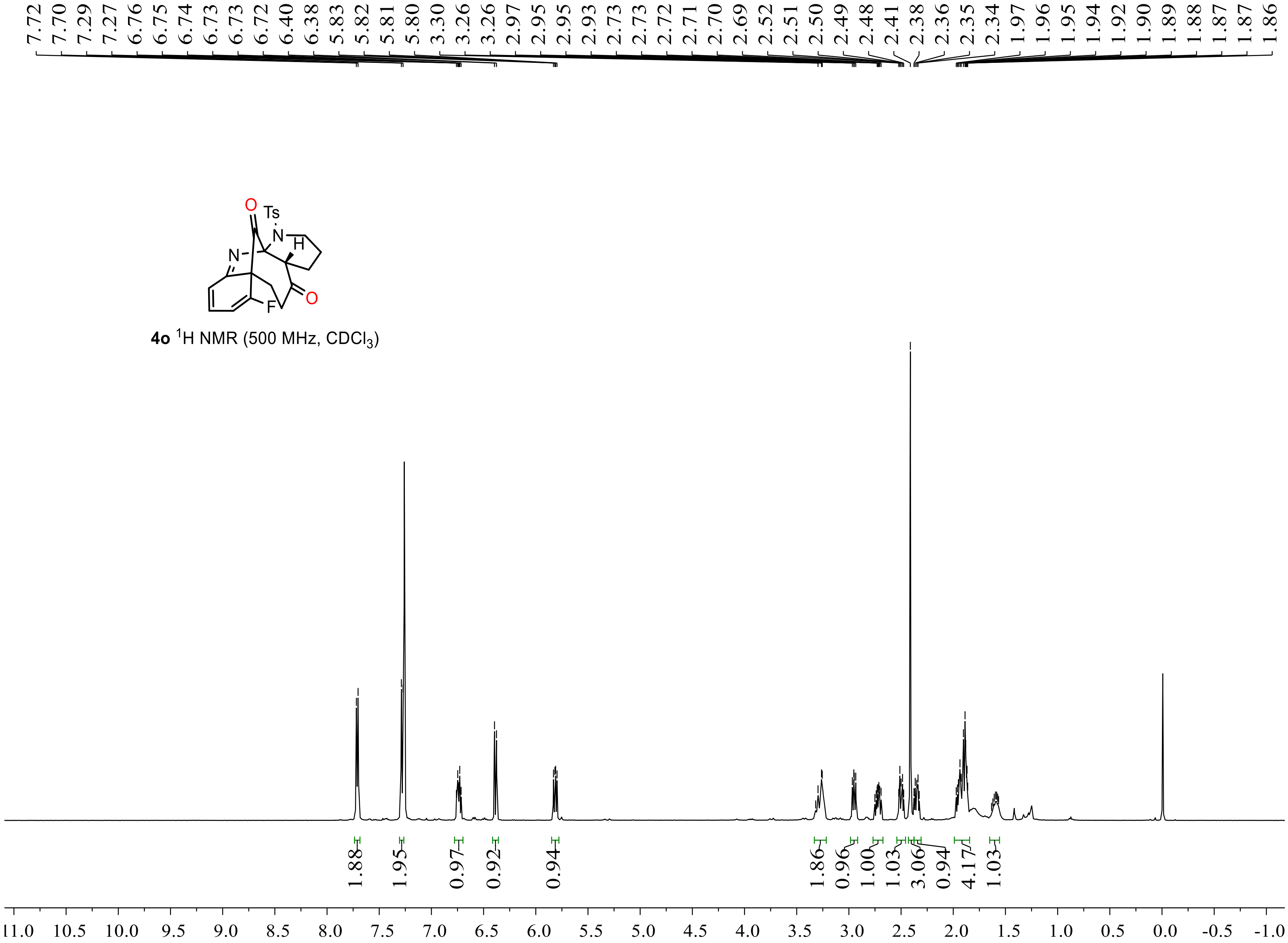

Supplementary Figure 205. ^{1}H NMR ($CDCl_3$, 500 MHz, 298 K) spectrum for **4o**

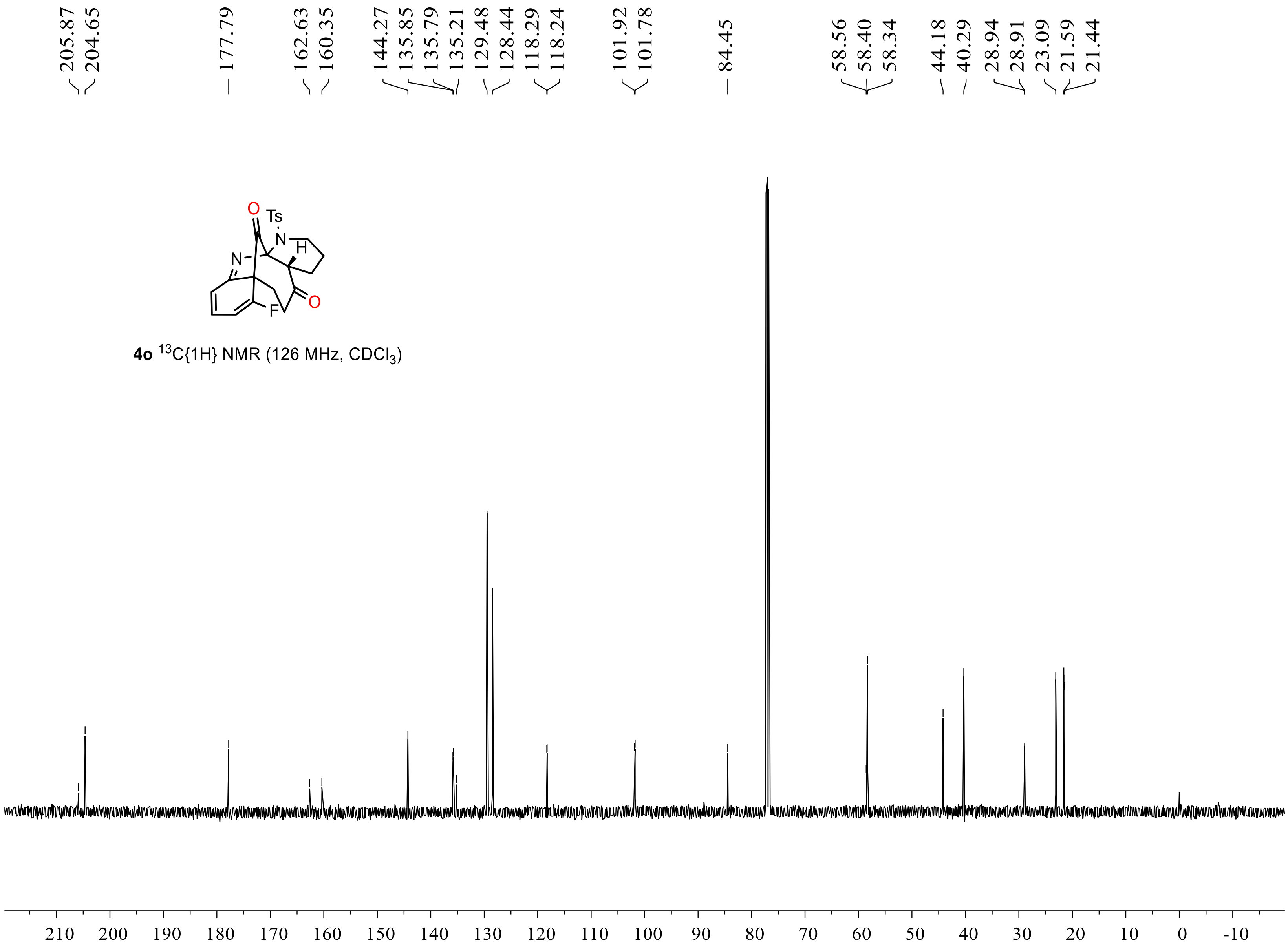

Supplementary Figure 206. ^{13}C NMR (CDCl$_3$, 126 MHz, 298 K) spectrum for **4o**

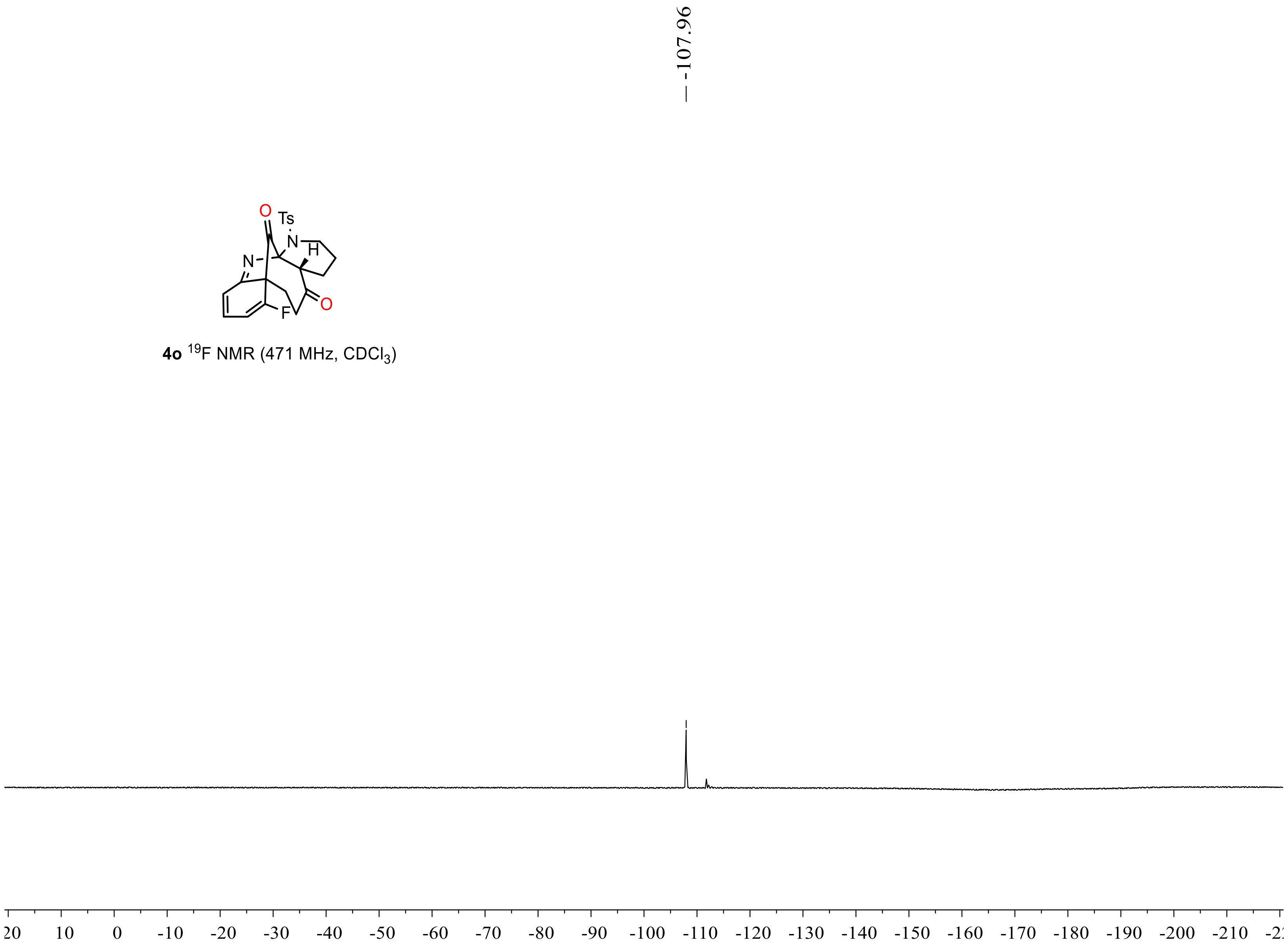

Supplementary Figure 207. ^{19}F NMR ($CDCl_3$, 471 MHz, 298 K) spectrum for **4o**

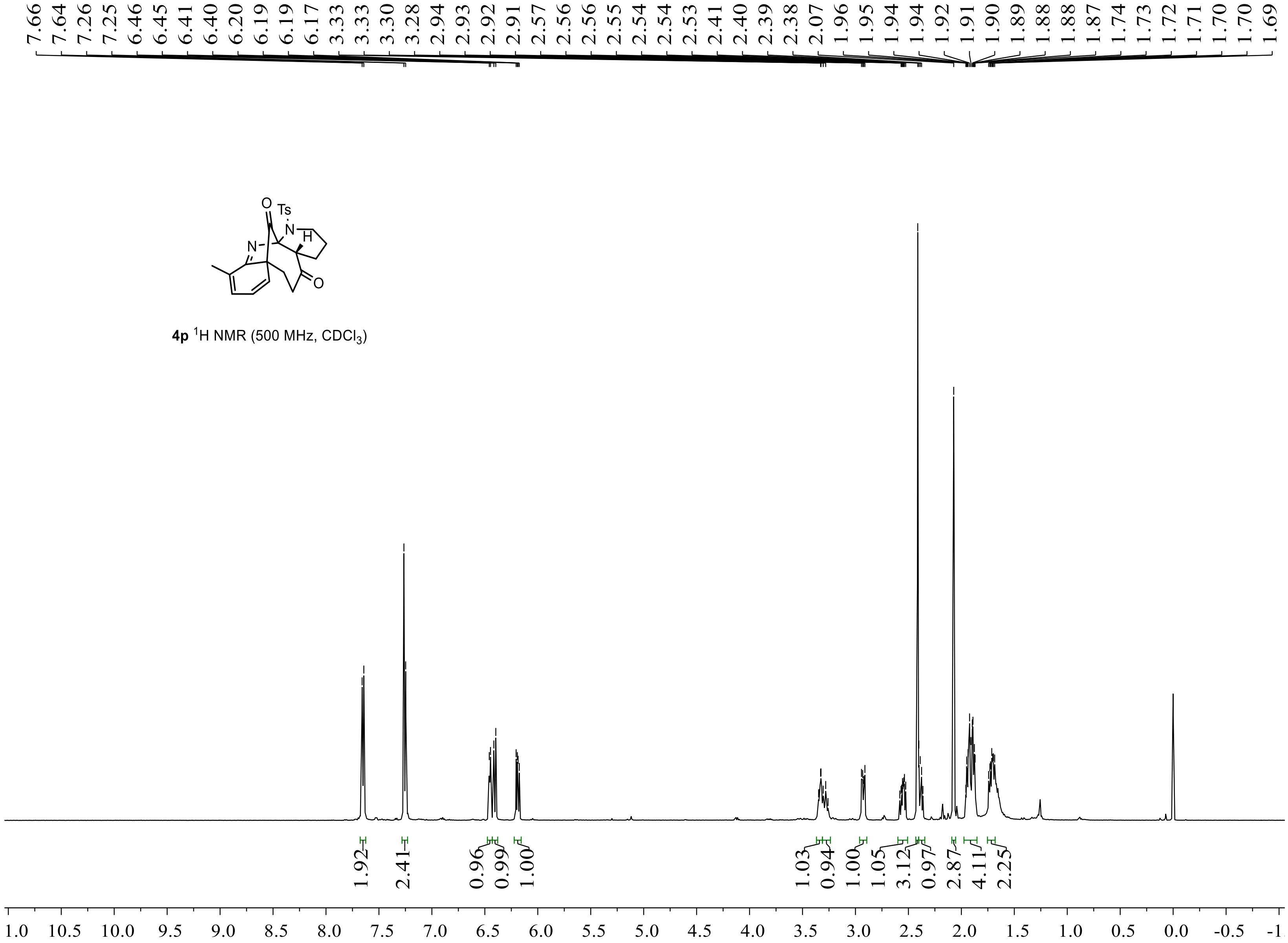

Supplementary Figure 208. ^{1}H NMR ($CDCl_3$, 500 MHz, 298 K) spectrum for **4p**

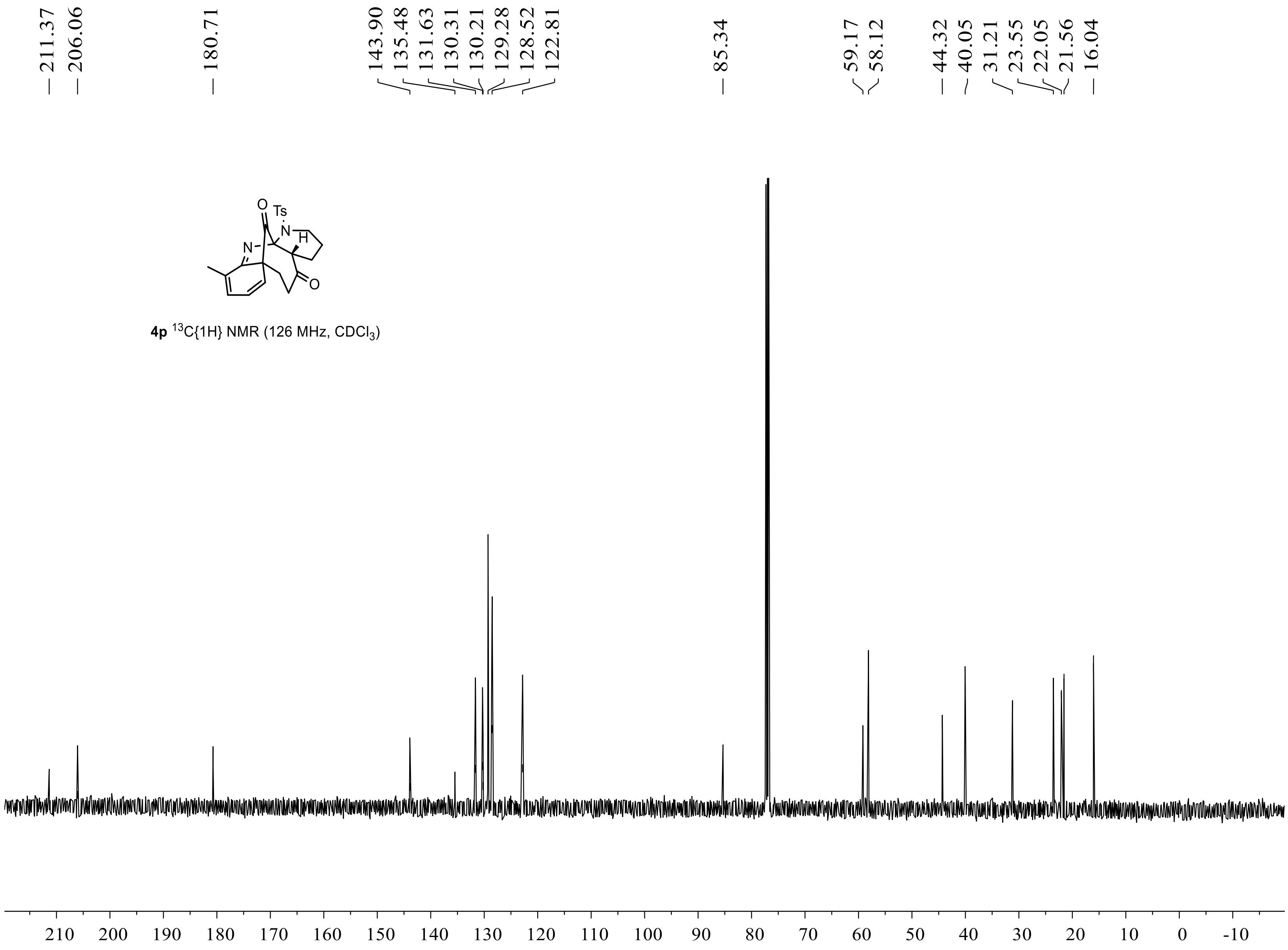

Supplementary Figure 209. ^{13}C NMR (CDCl$_3$, 126 MHz, 298 K) spectrum for **4p**

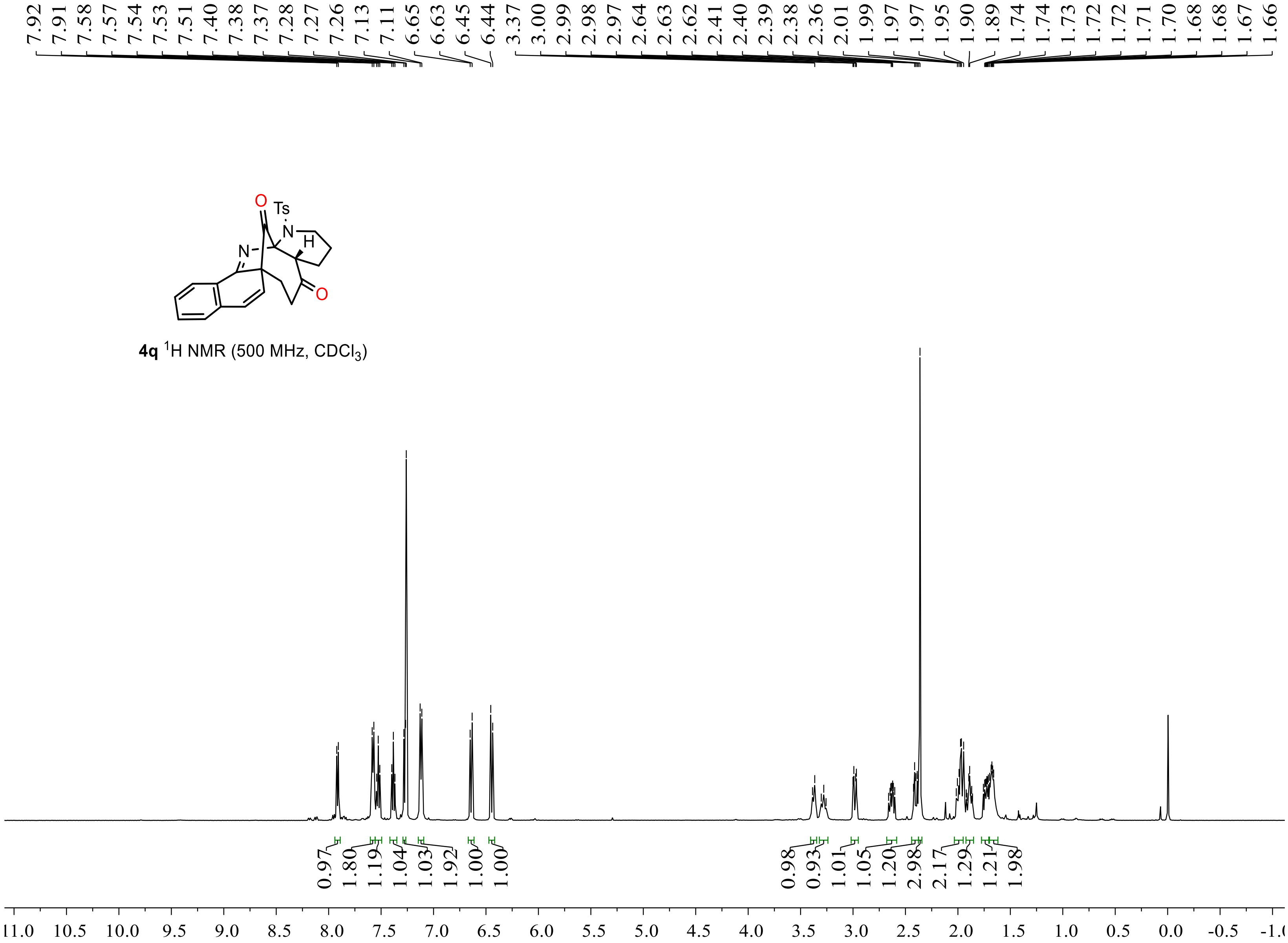

Supplementary Figure 210. ^{1}H NMR ($CDCl_3$, 500 MHz, 298 K) spectrum for **4q**

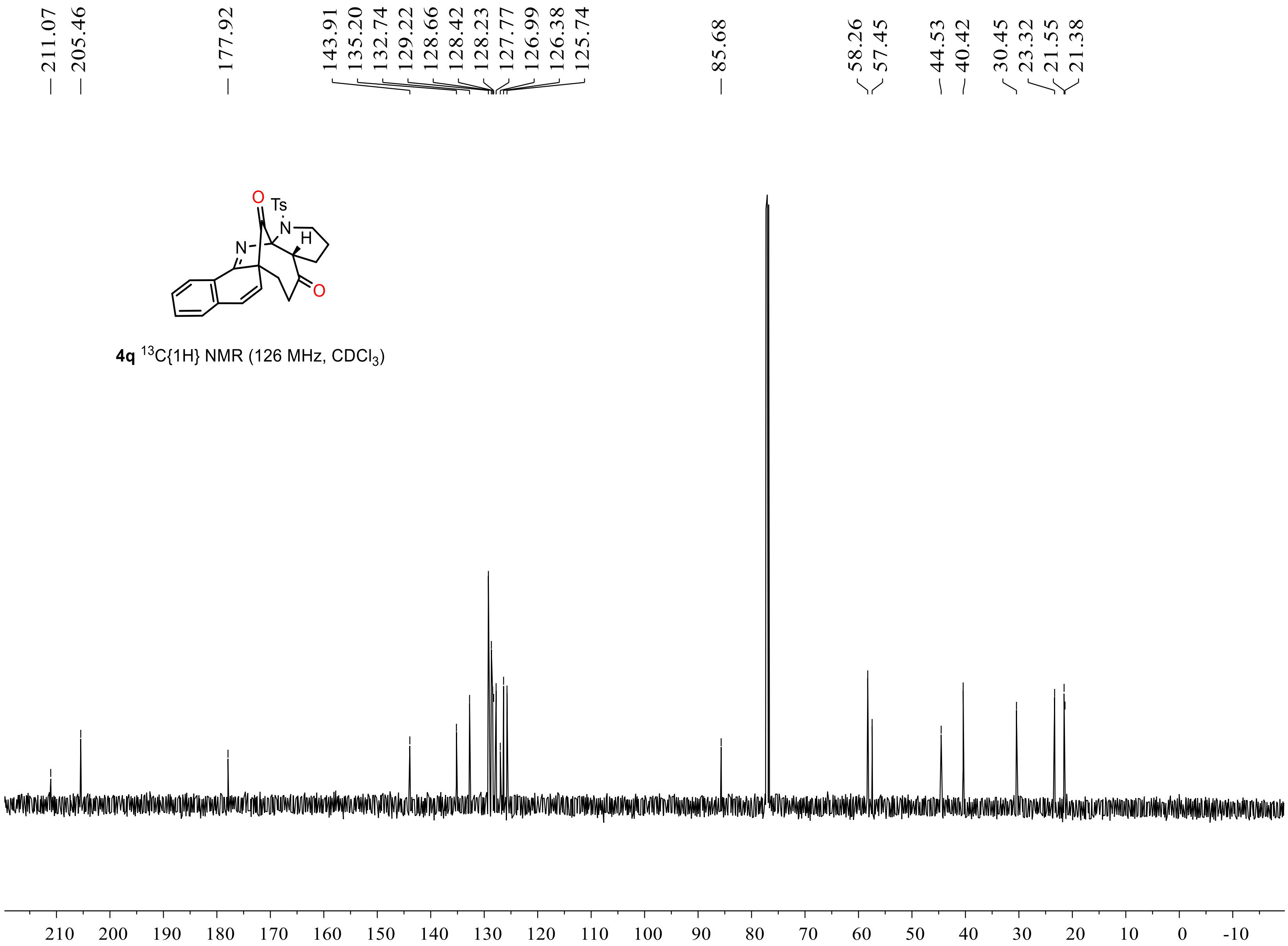

Supplementary Figure 211. ^{13}C NMR ($CDCl_3$, 126 MHz, 298 K) spectrum for **4q**

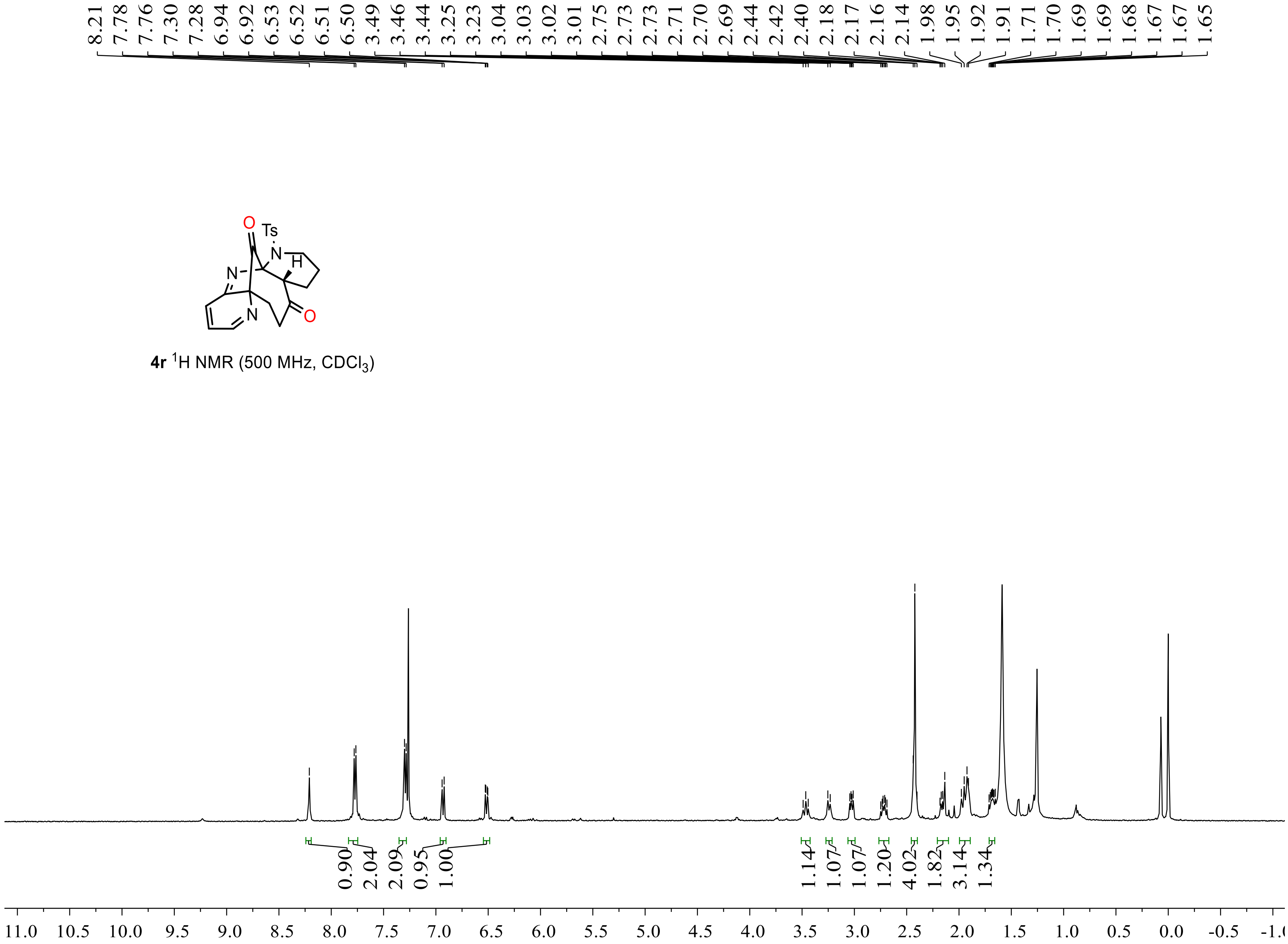

Supplementary Figure 212. ^{1}H NMR ($CDCl_3$, 500 MHz, 298 K) spectrum for **4r**

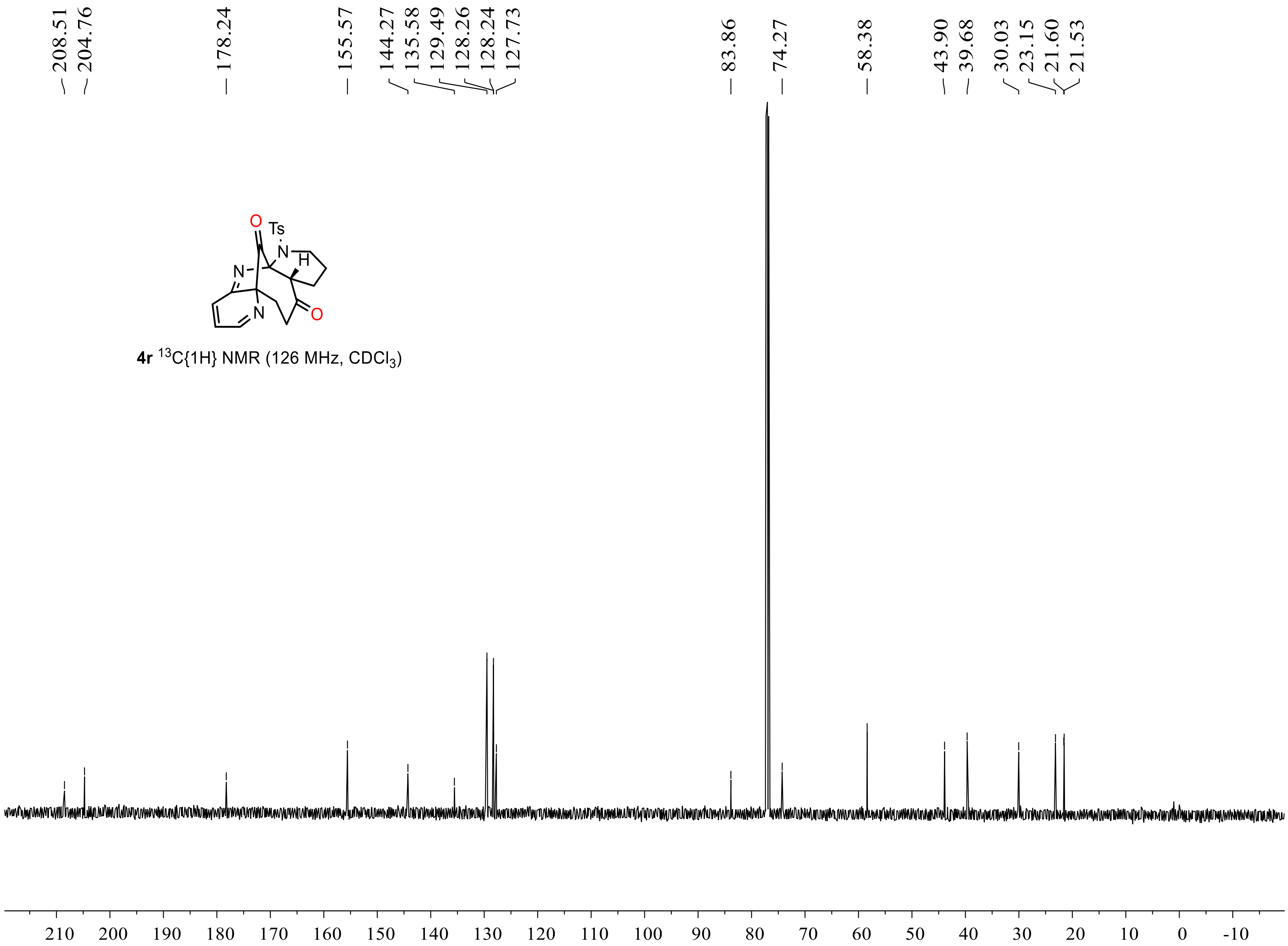

Supplementary Figure 213. ^{13}C NMR ($CDCl_3$, 126 MHz, 298 K) spectrum for **4r**

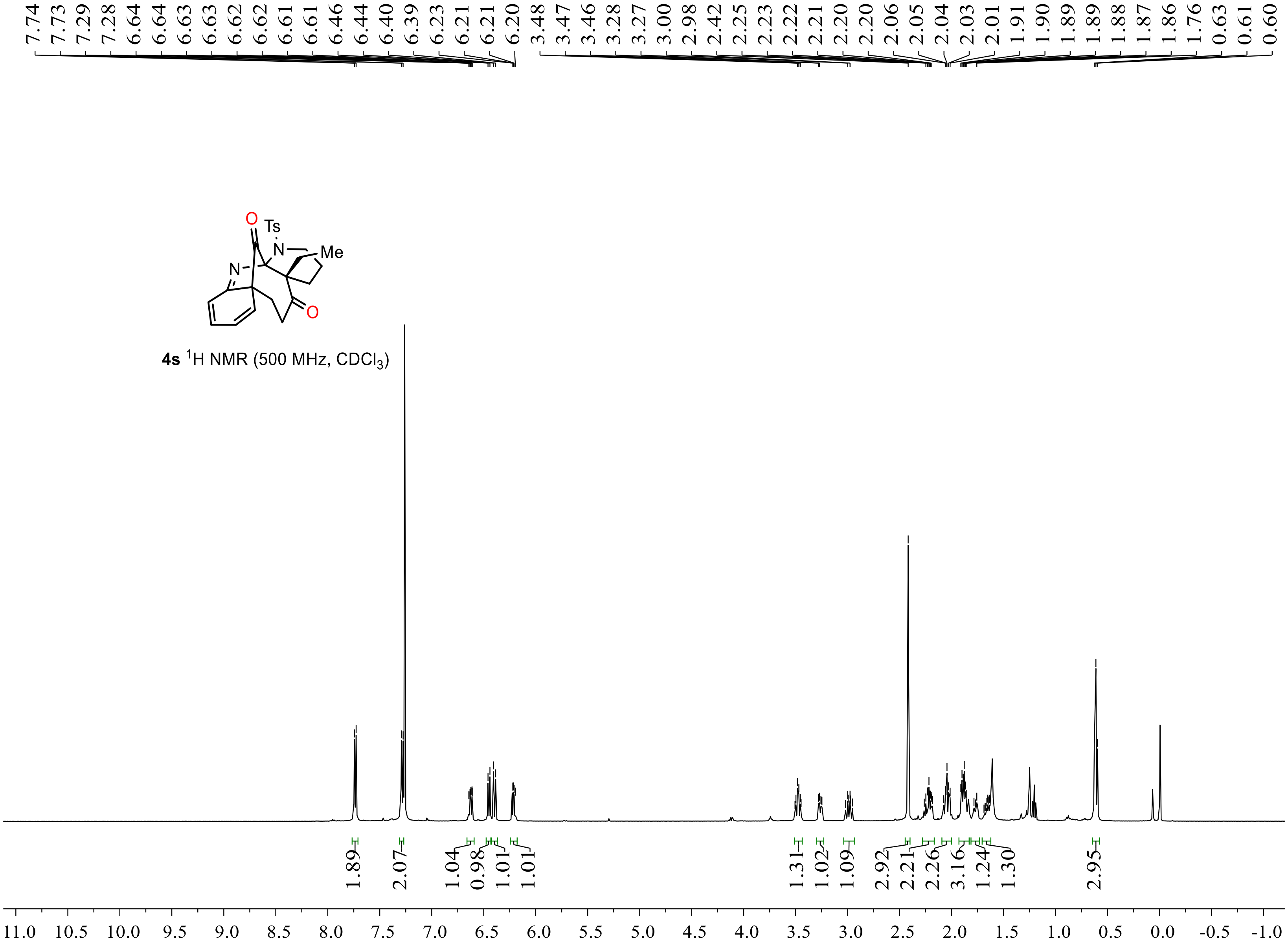

Supplementary Figure 214. ^{1}H NMR ($CDCl_3$, 500 MHz, 298 K) spectrum for **4s**

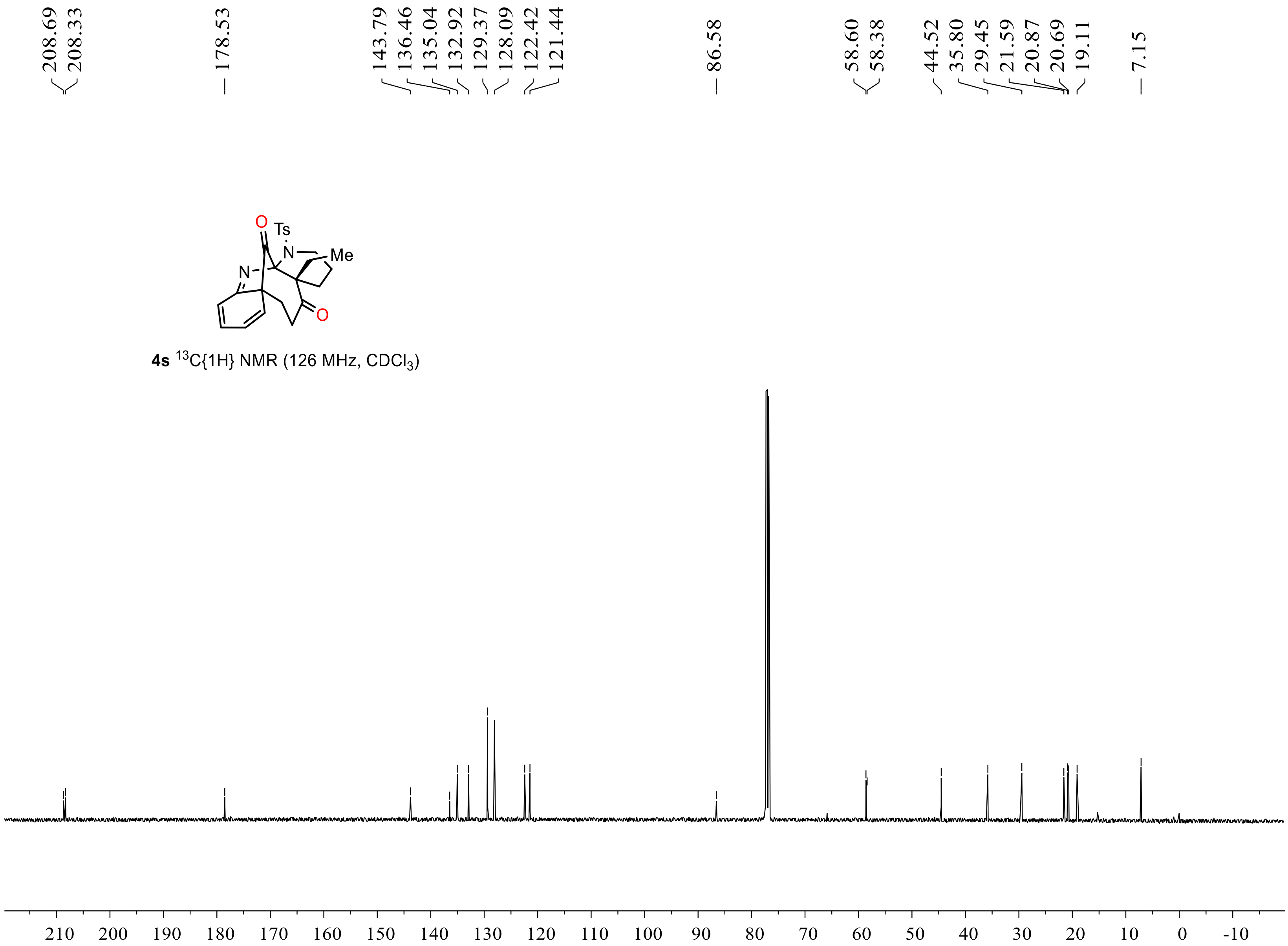

Supplementary Figure 215. ^{13}C NMR ($CDCl_3$, 126 MHz, 298 K) spectrum for **4s**

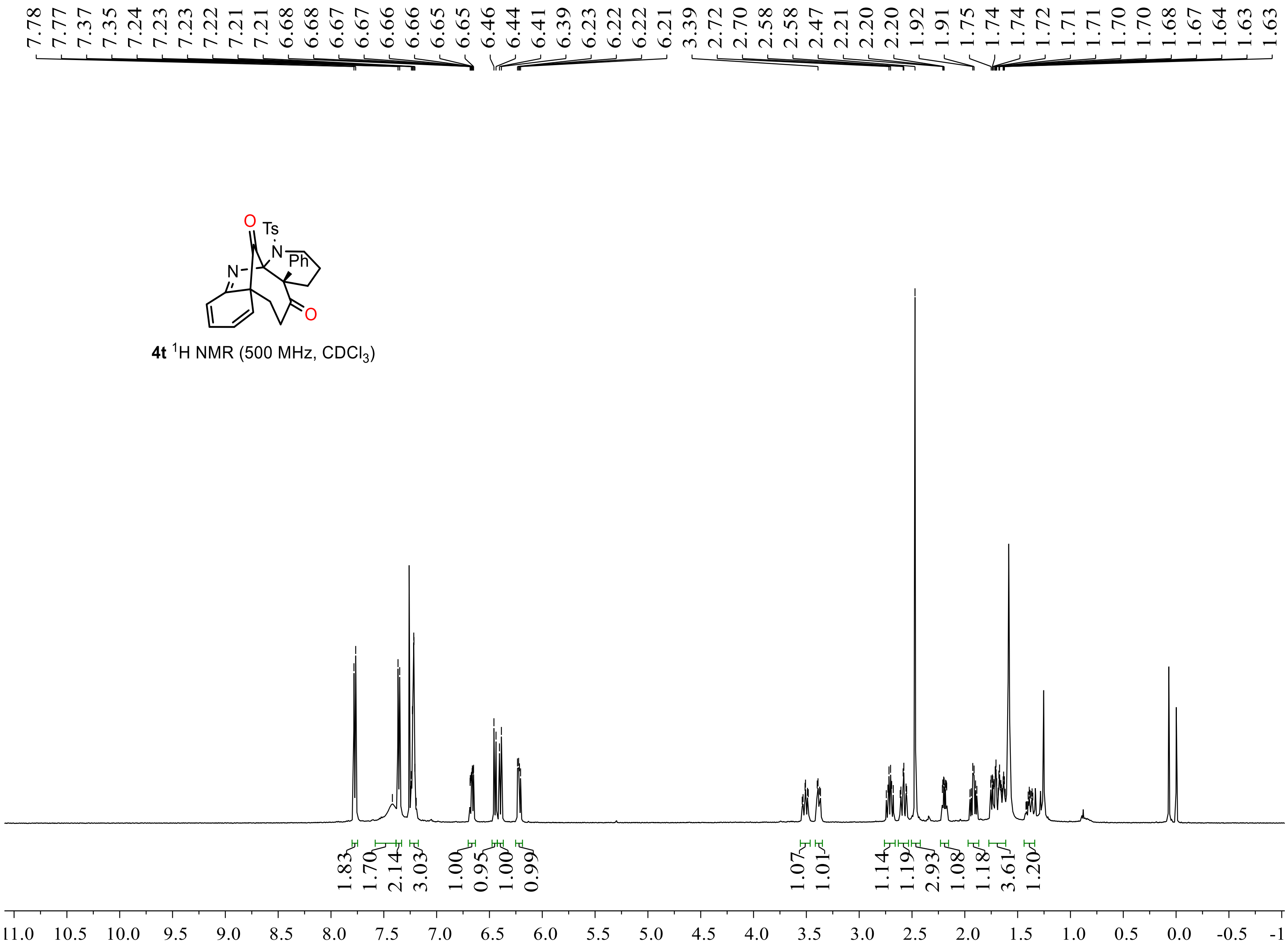

Supplementary Figure 216. ^{1}H NMR ($CDCl_3$, 500 MHz, 298 K) spectrum for **4t**

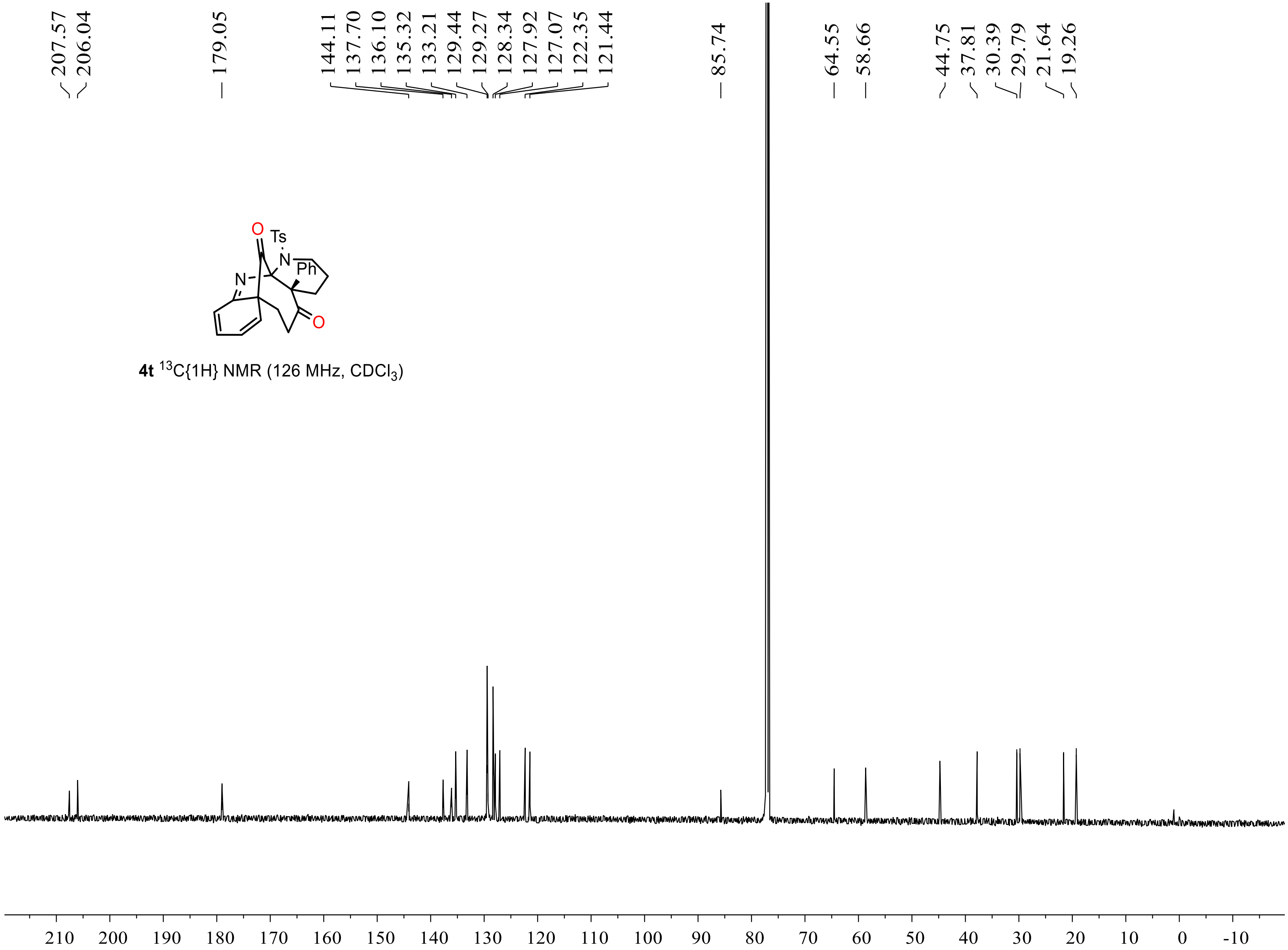

Supplementary Figure 217. ^{13}C NMR ($CDCl_3$, 126 MHz, 298 K) spectrum for **4t**

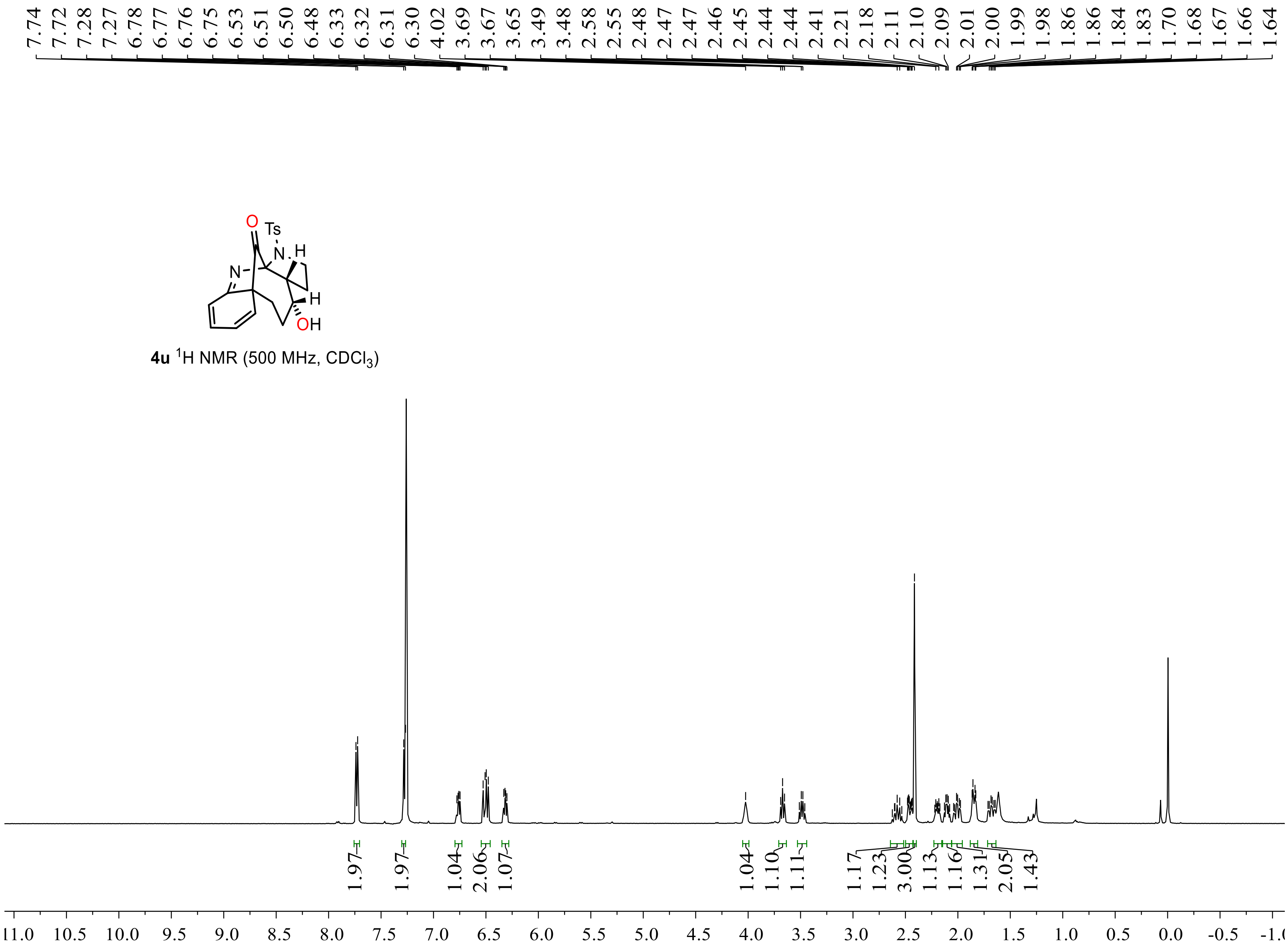

Supplementary Figure 218. ^{1}H NMR ($CDCl_3$, 500 MHz, 298 K) spectrum for **4u**

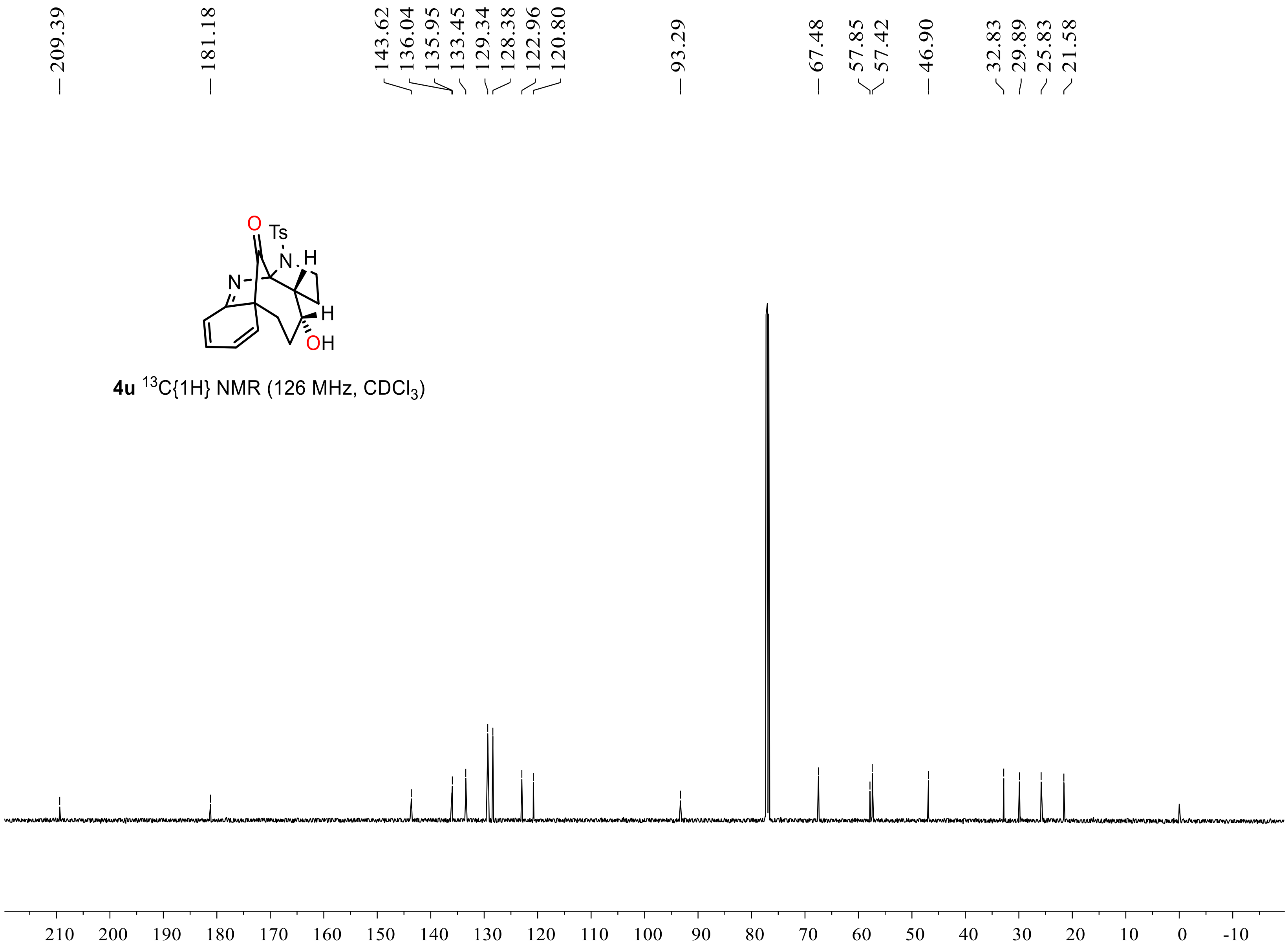

Supplementary Figure 219. ^{13}C NMR ($CDCl_3$, 126 MHz, 298 K) spectrum for **4u**

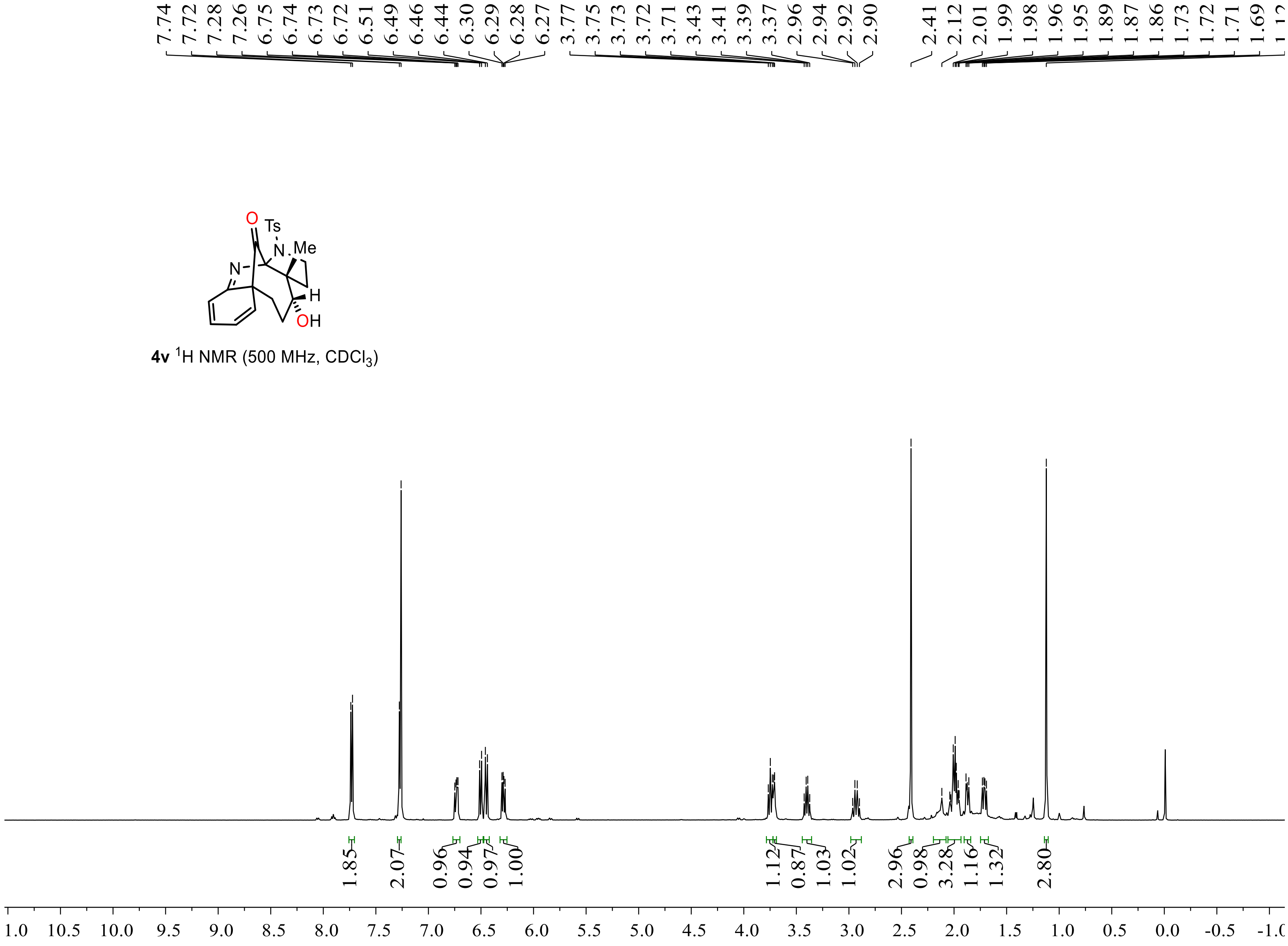

Supplementary Figure 220. ^{1}H NMR ($CDCl_3$, 500 MHz, 298 K) spectrum for **4v**

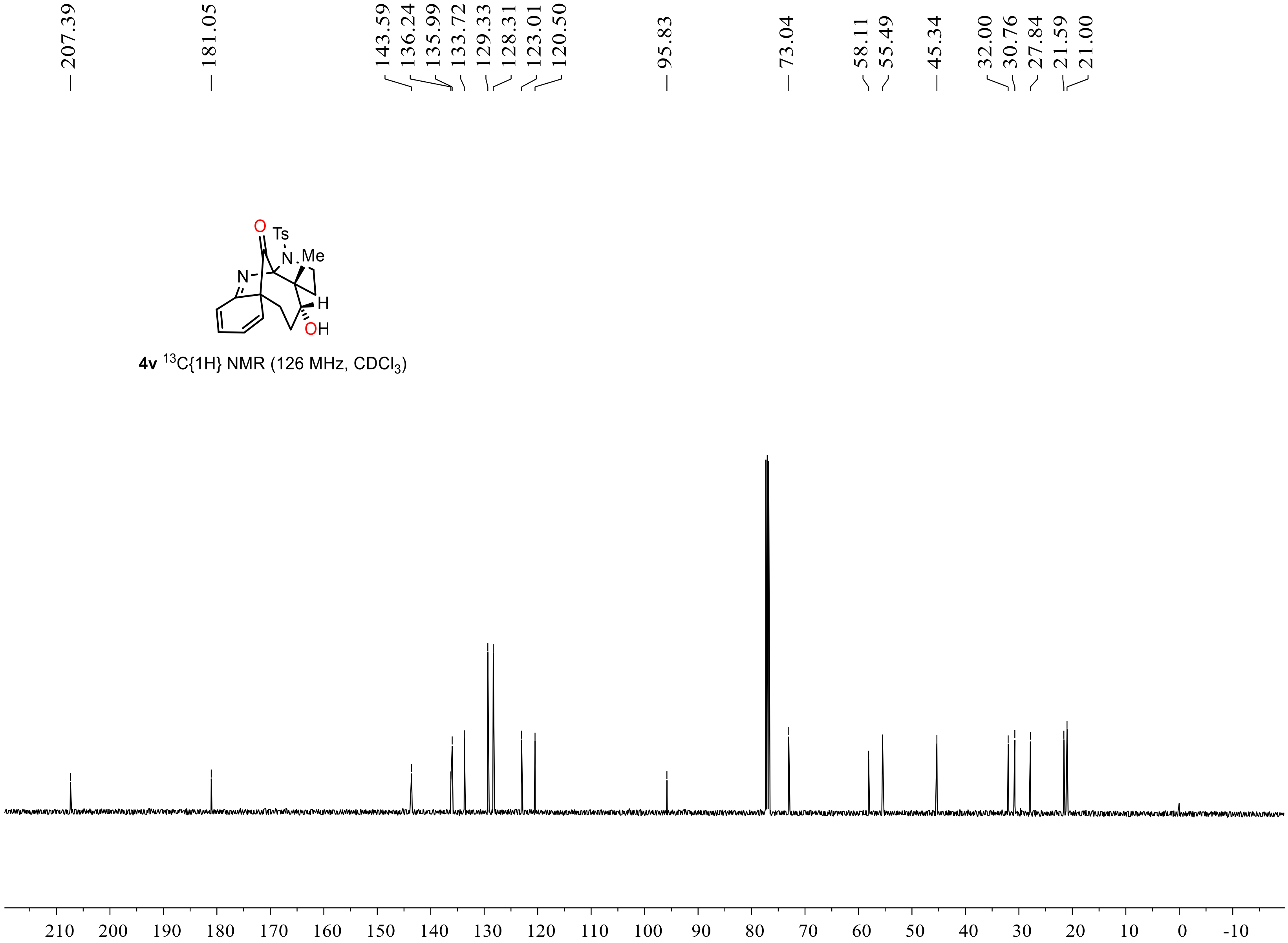

Supplementary Figure 221. ^{13}C NMR ($CDCl_3$, 126 MHz, 298 K) spectrum for **4v**

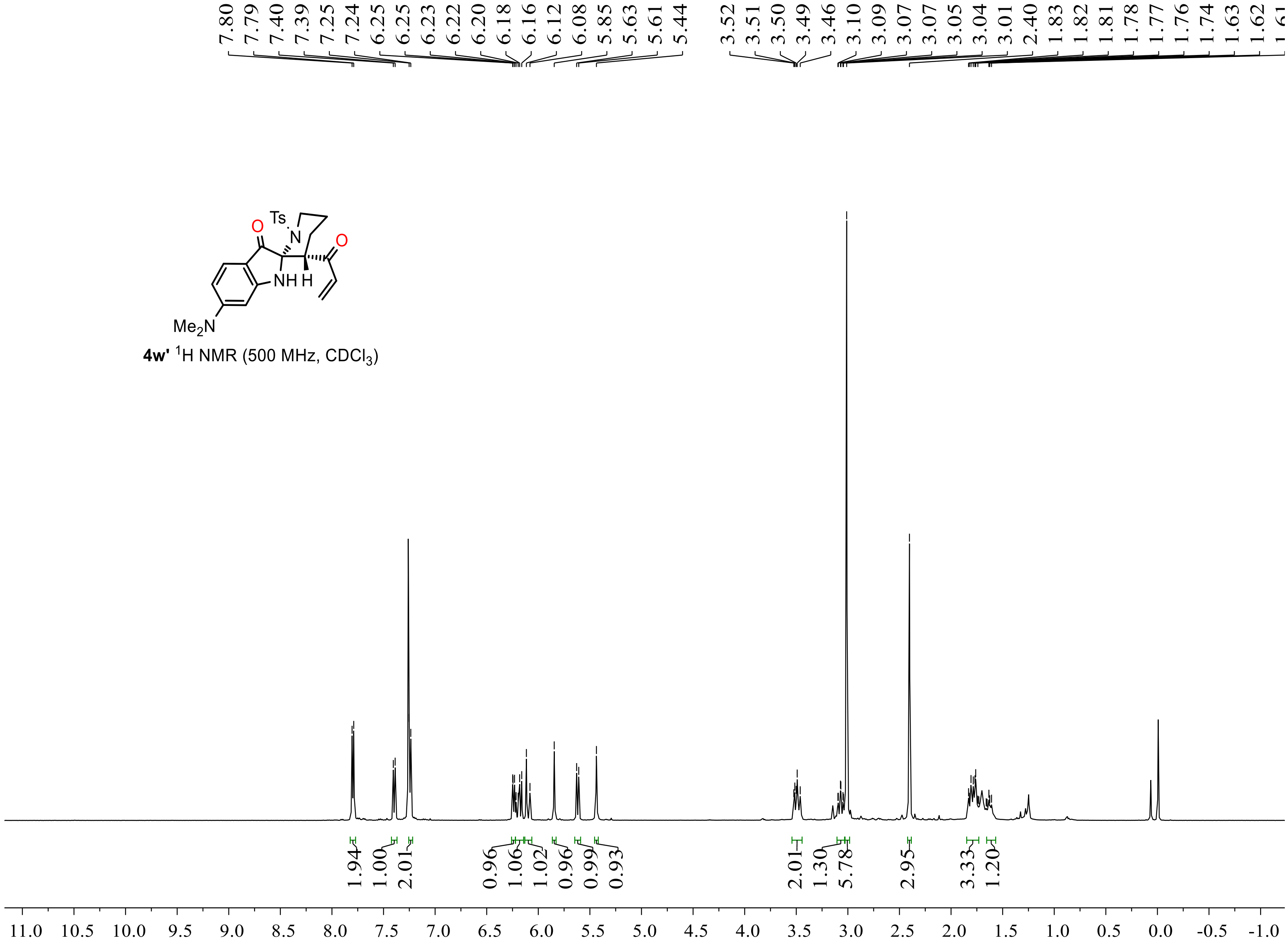

Supplementary Figure 222. ^{1}H NMR ($CDCl_3$, 500 MHz, 298 K) spectrum for **4w’**

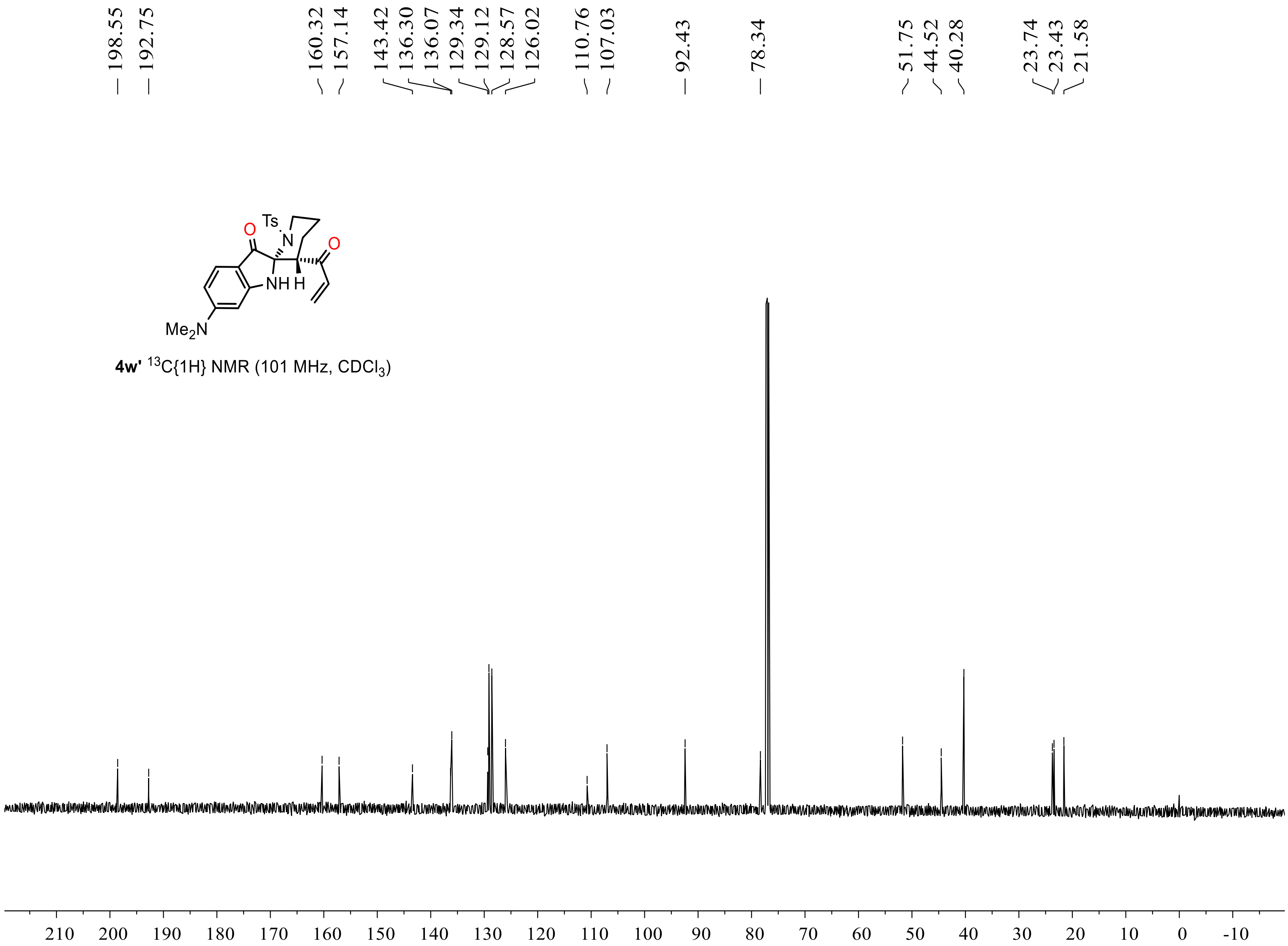

Supplementary Figure 223. ^{13}C NMR ($CDCl_3$, 126 MHz, 298 K) spectrum for **4w’**

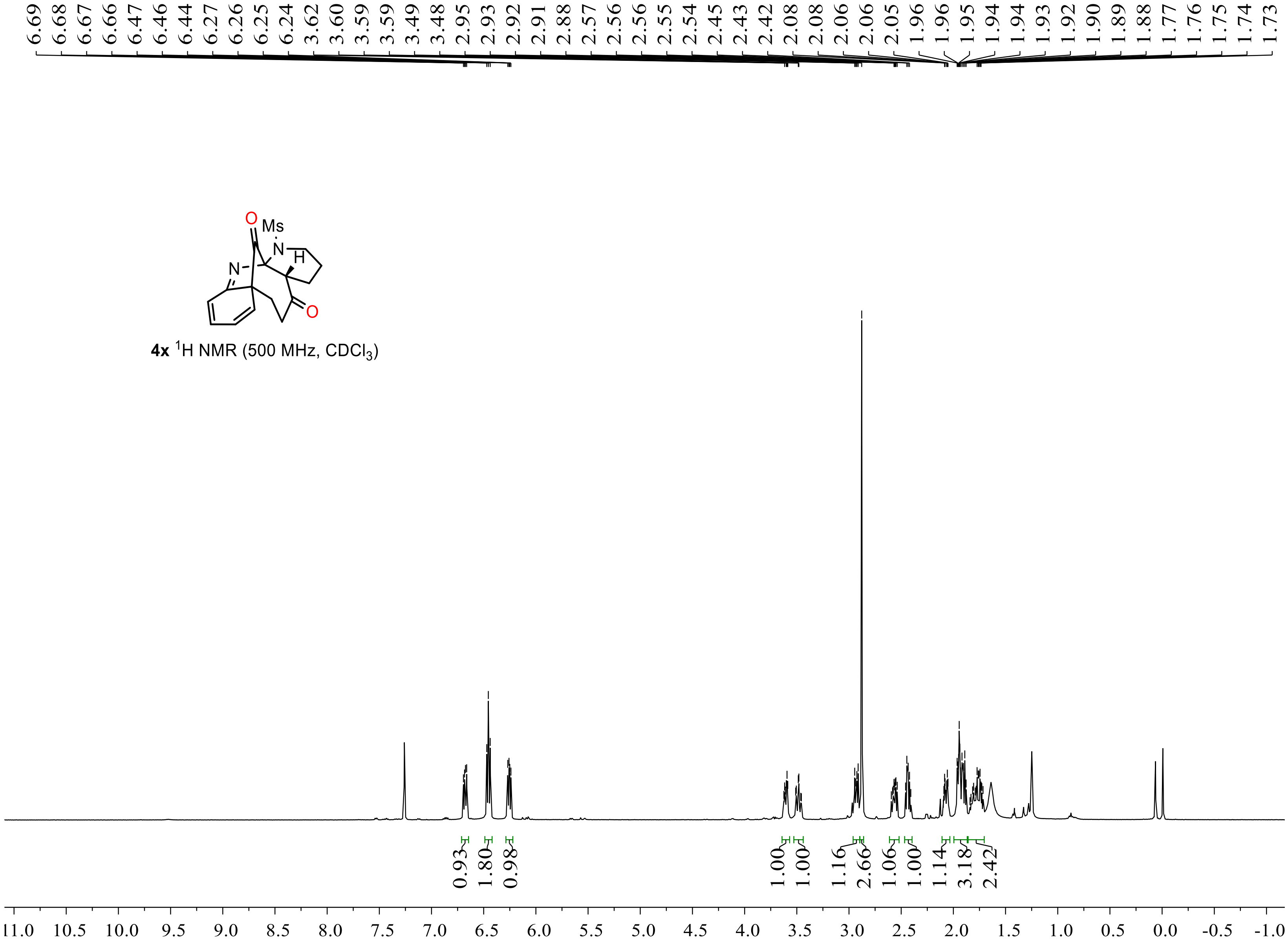

Supplementary Figure 224. ^{1}H NMR ($CDCl_3$, 500 MHz, 298 K) spectrum for **4x**

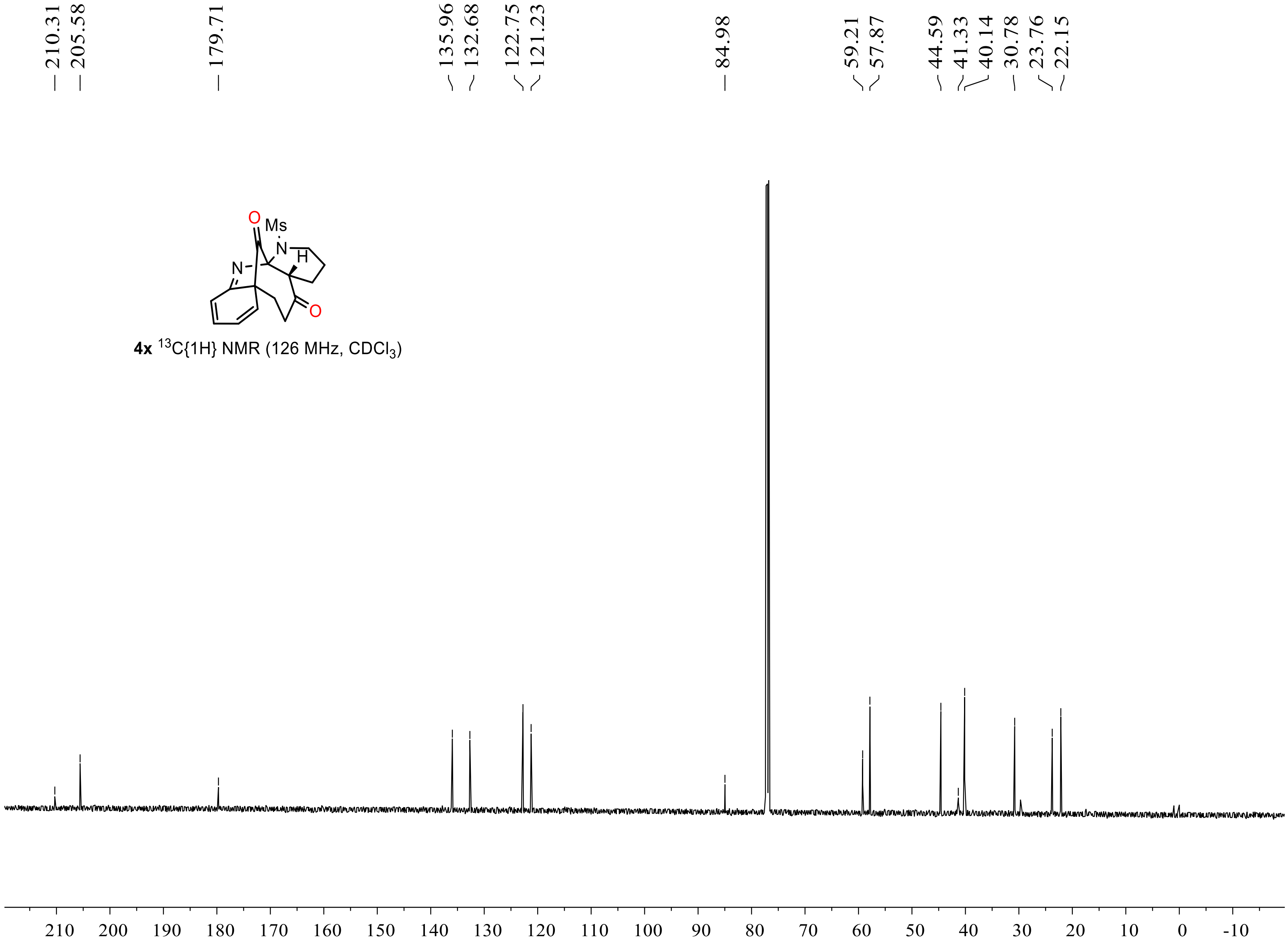

Supplementary Figure 225. ^{13}C NMR ($CDCl_3$, 126 MHz, 298 K) spectrum for **4x**

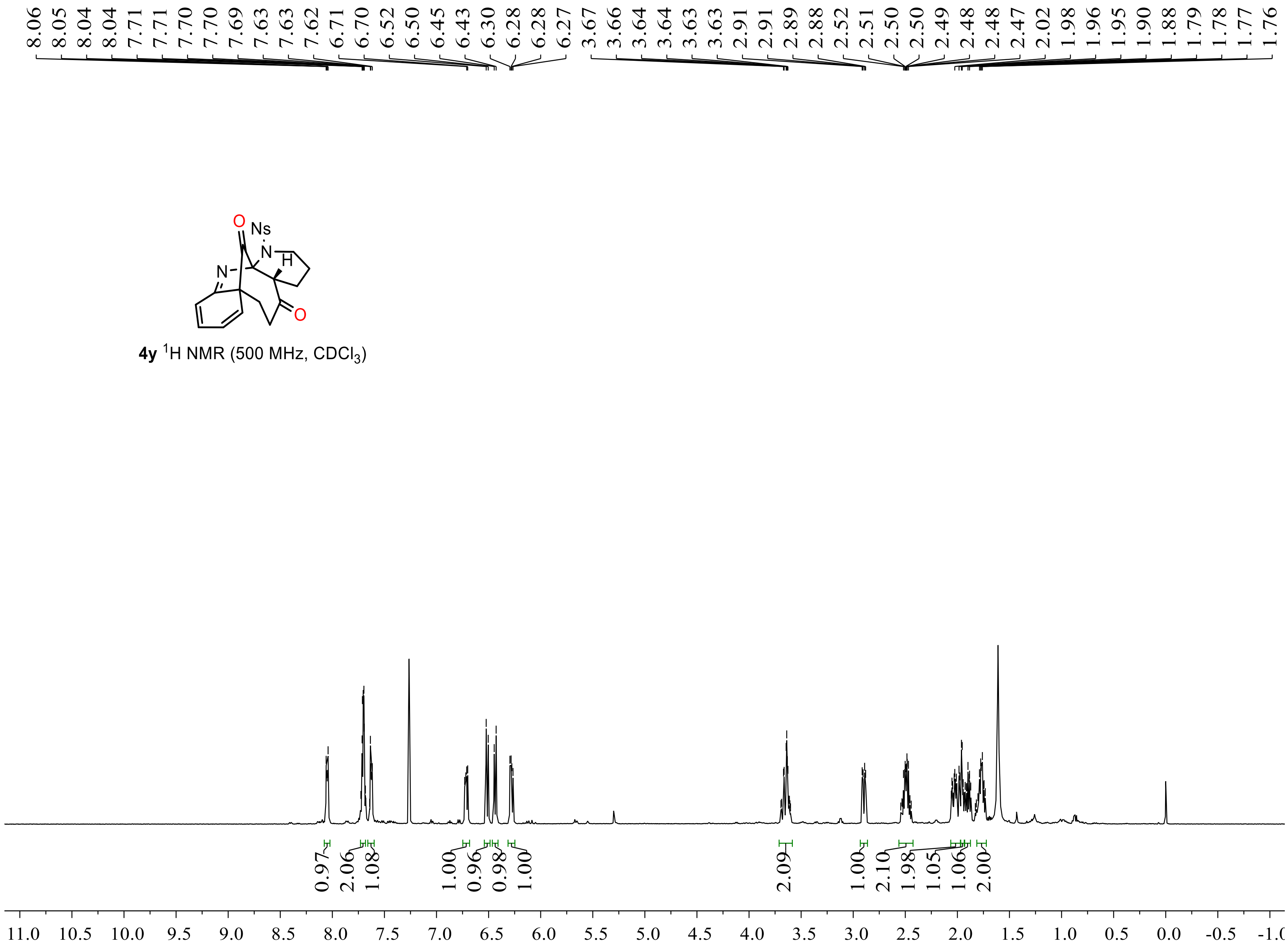

Supplementary Figure 226. ^{1}H NMR ($CDCl_3$, 500 MHz, 298 K) spectrum for **4y**

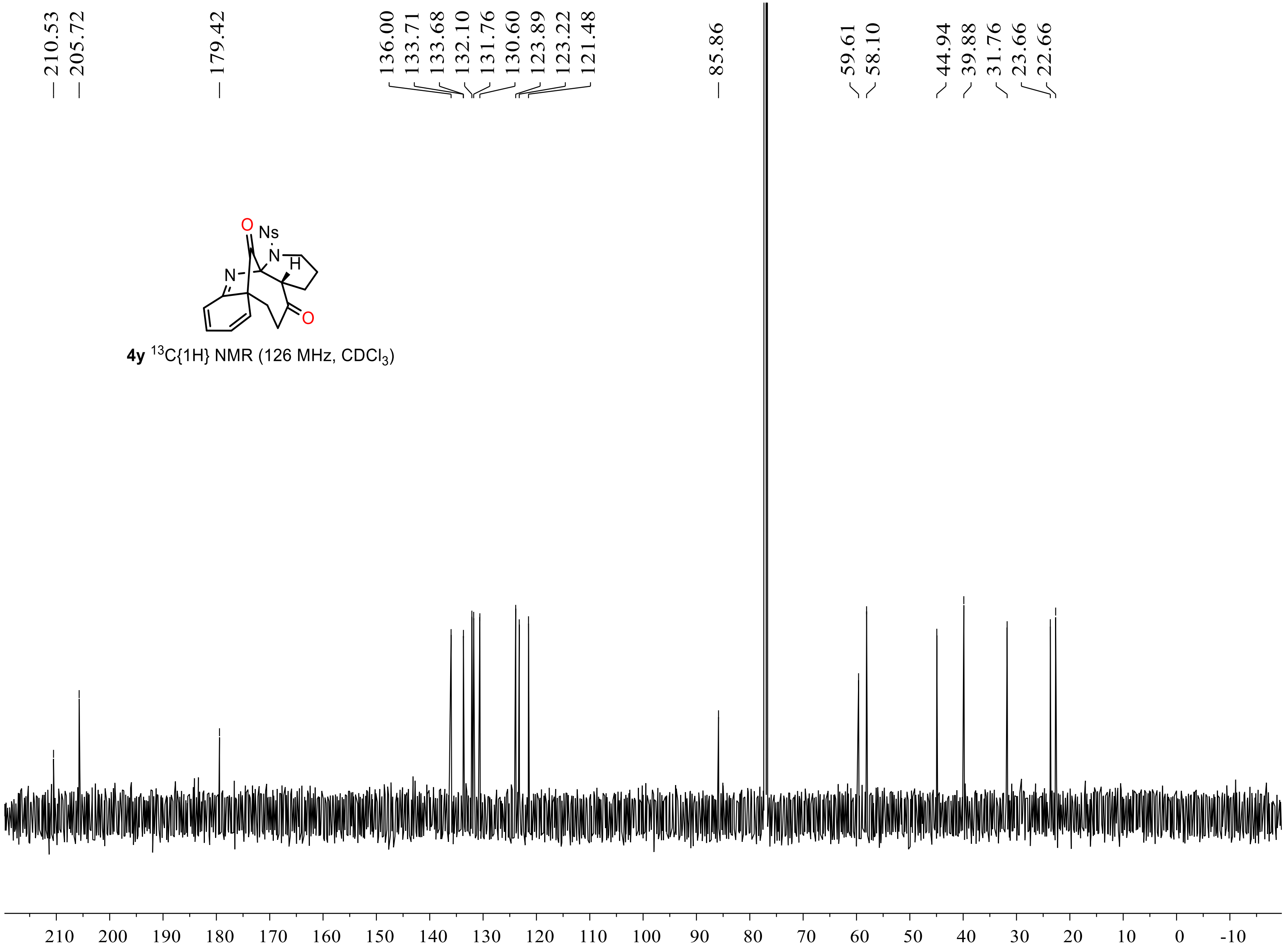

Supplementary Figure 227. ^{13}C NMR (CDCl$_3$, 126 MHz, 298 K) spectrum for **4y**

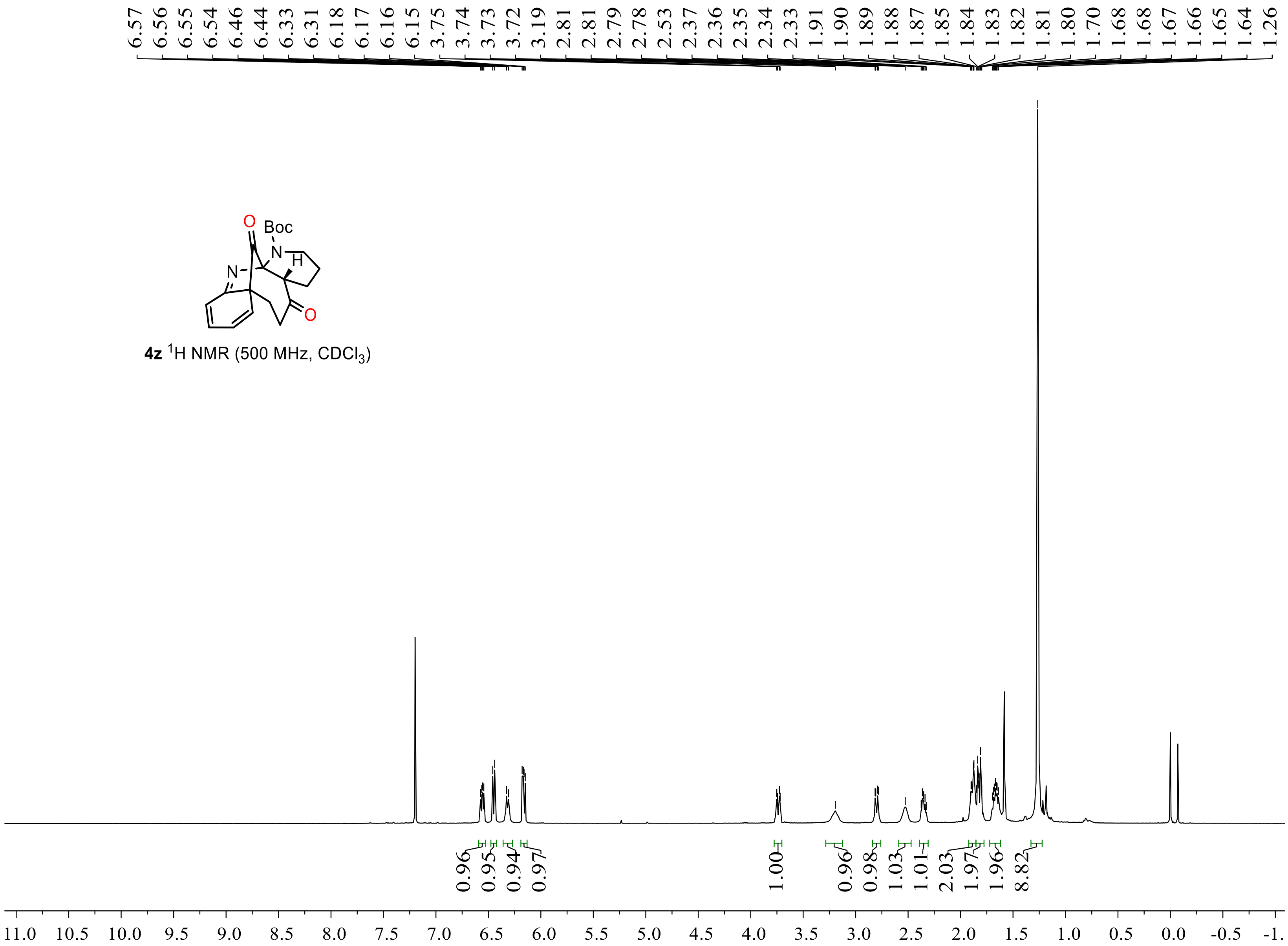

Supplementary Figure 228. ^{1}H NMR ($CDCl_3$, 500 MHz, 298 K) spectrum for **4z**

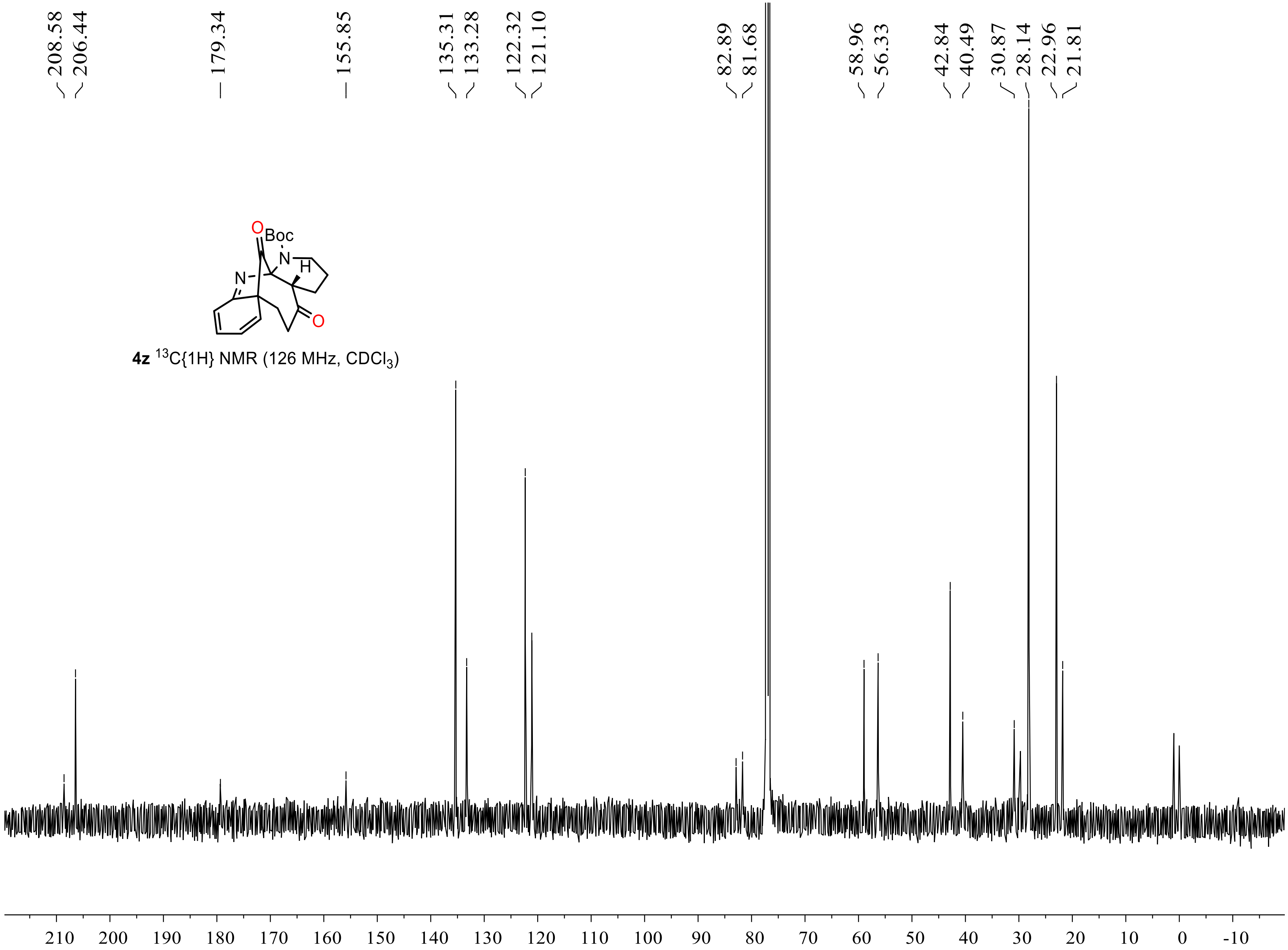

Supplementary Figure 229. ^{13}C NMR ($CDCl_3$, 126 MHz, 298 K) spectrum for **4z**

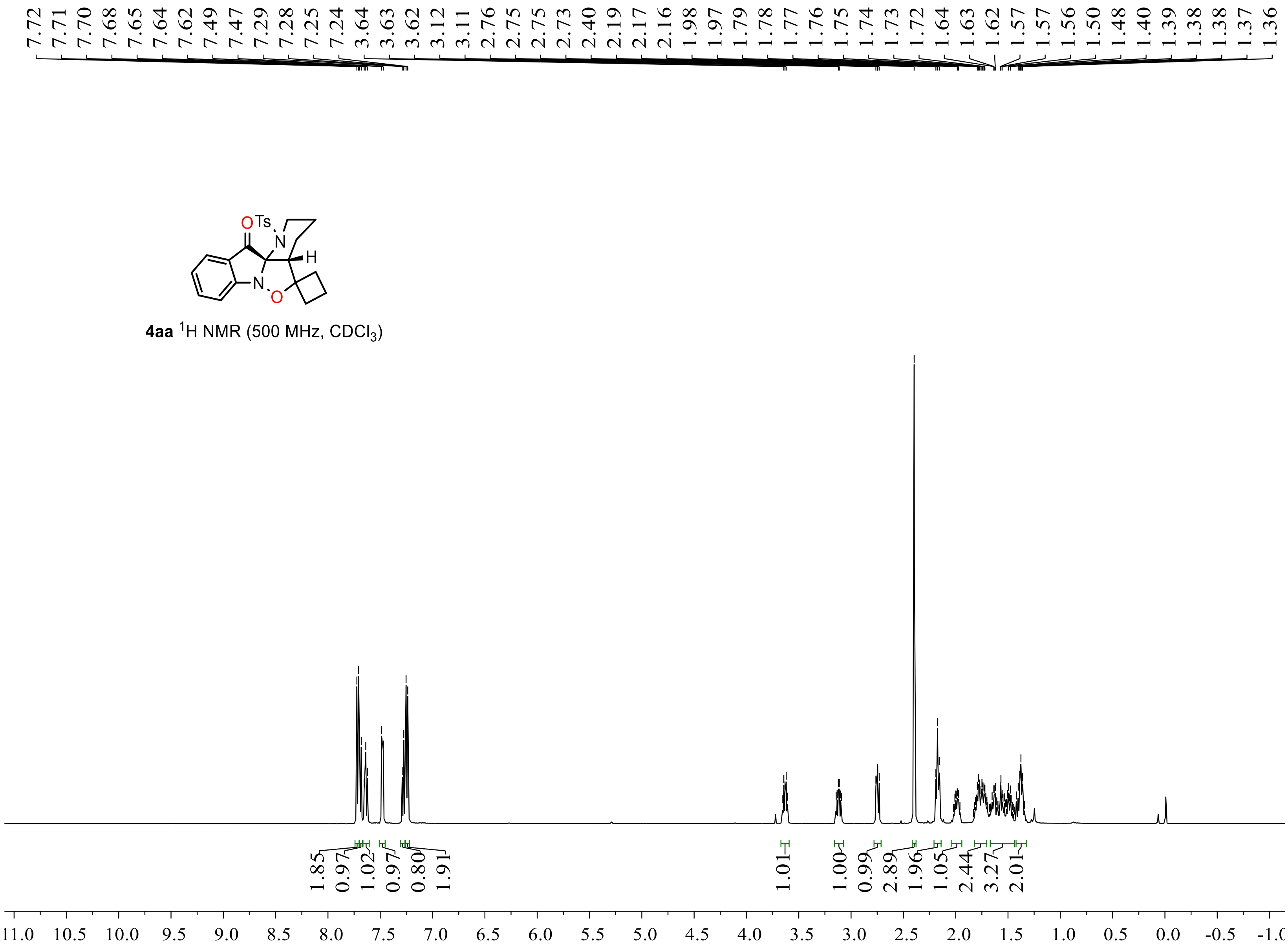

Supplementary Figure 230. ^{1}H NMR ($CDCl_3$, 500 MHz, 298 K) spectrum for **4aa**

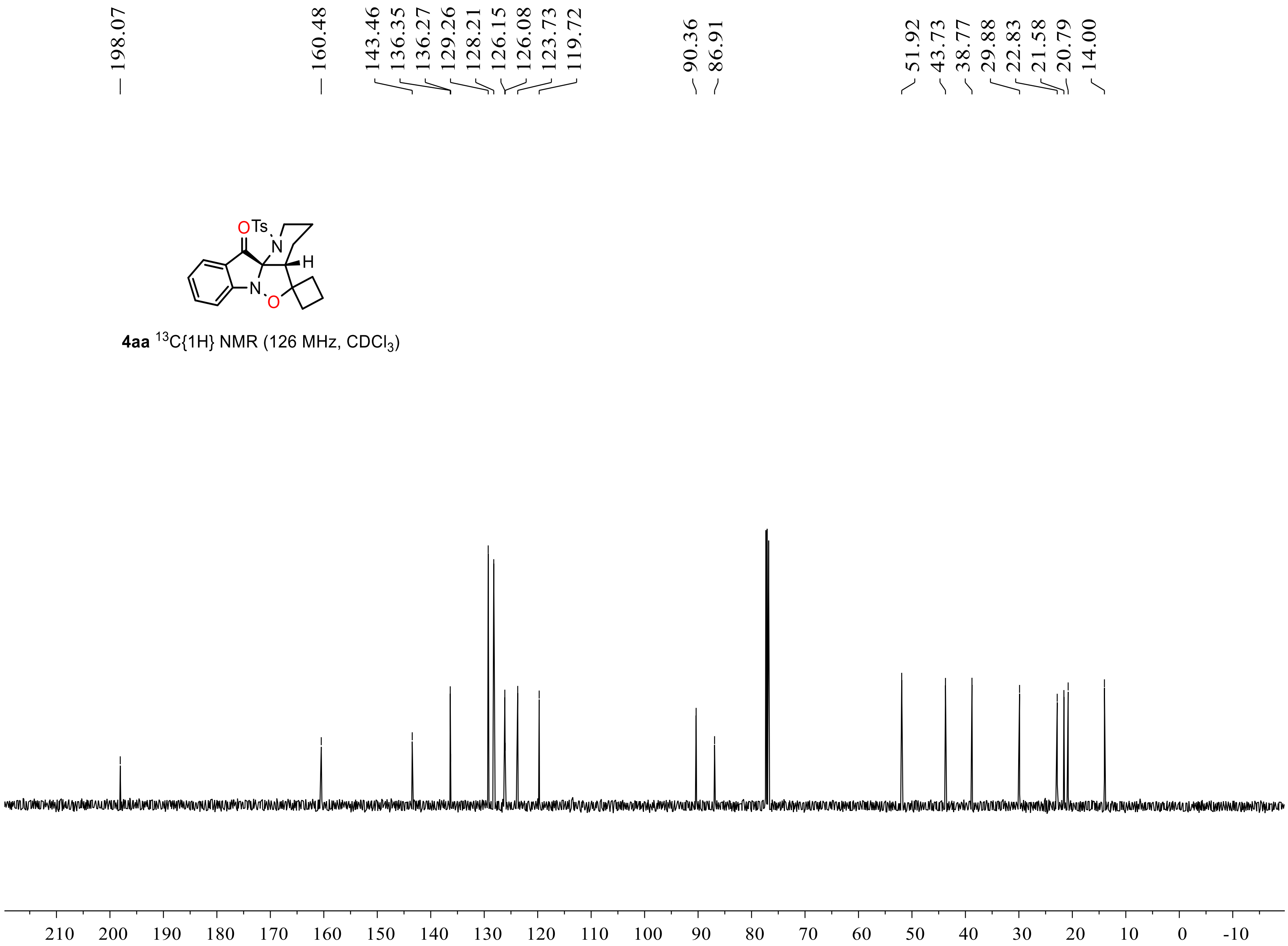

Supplementary Figure 231. ^{13}C NMR ($CDCl_3$, 126 MHz, 298 K) spectrum for **4aa**

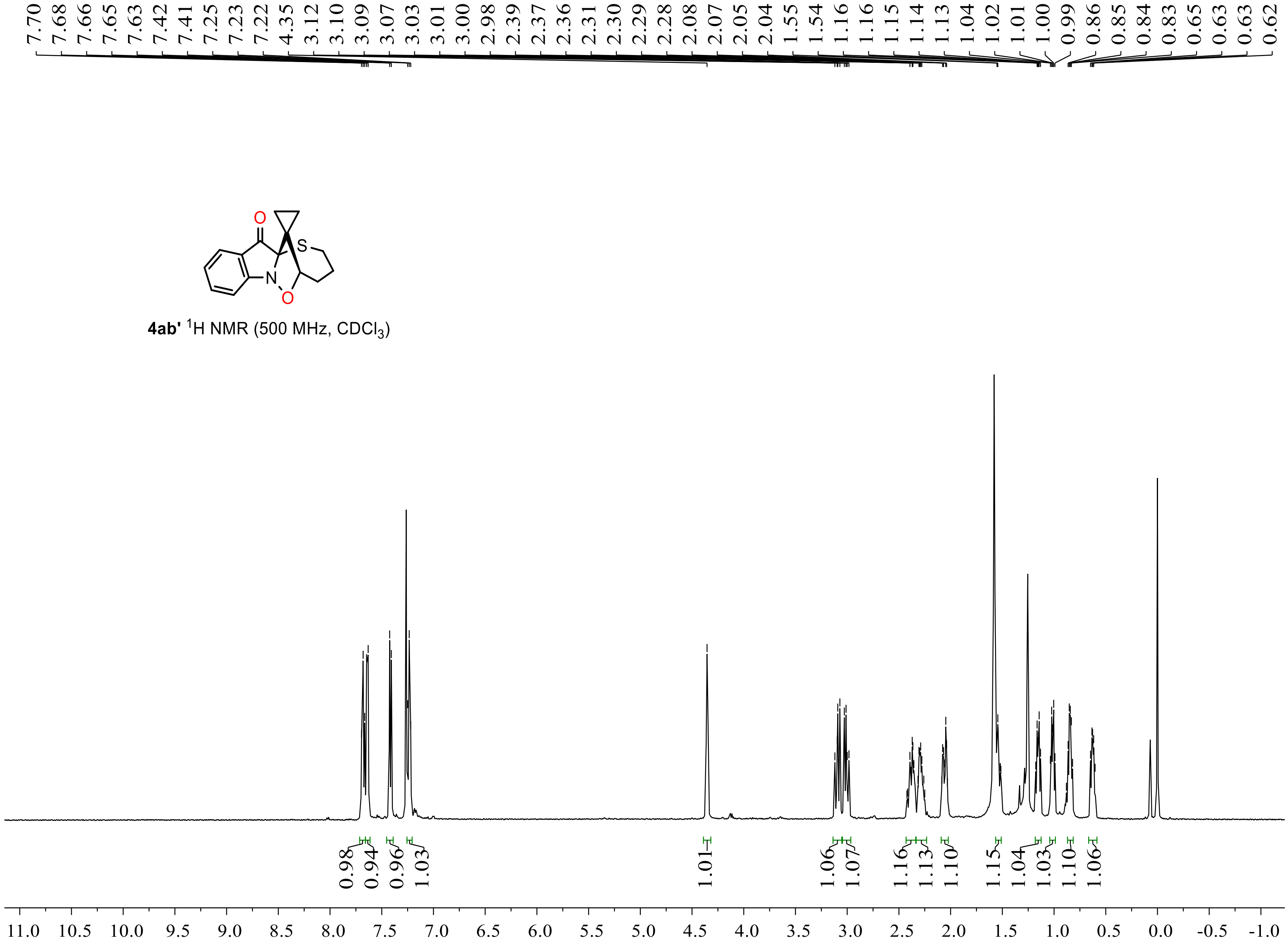

Supplementary Figure 232. ^{1}H NMR ($CDCl_3$, 500 MHz, 298 K) spectrum for **4ab’**

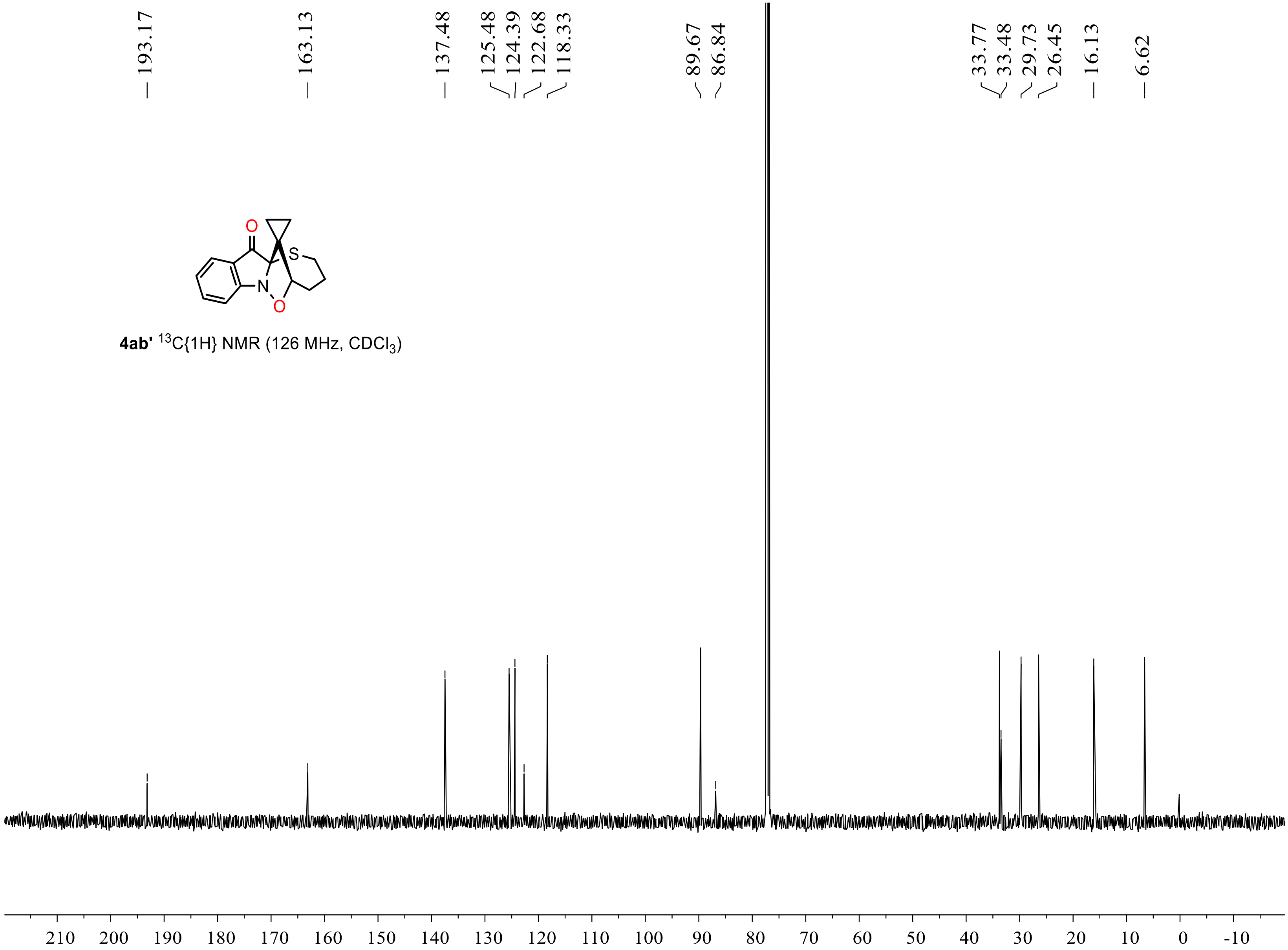

Supplementary Figure 233. ^{13}C NMR ($CDCl_3$, 126 MHz, 298 K) spectrum for **4ab'**

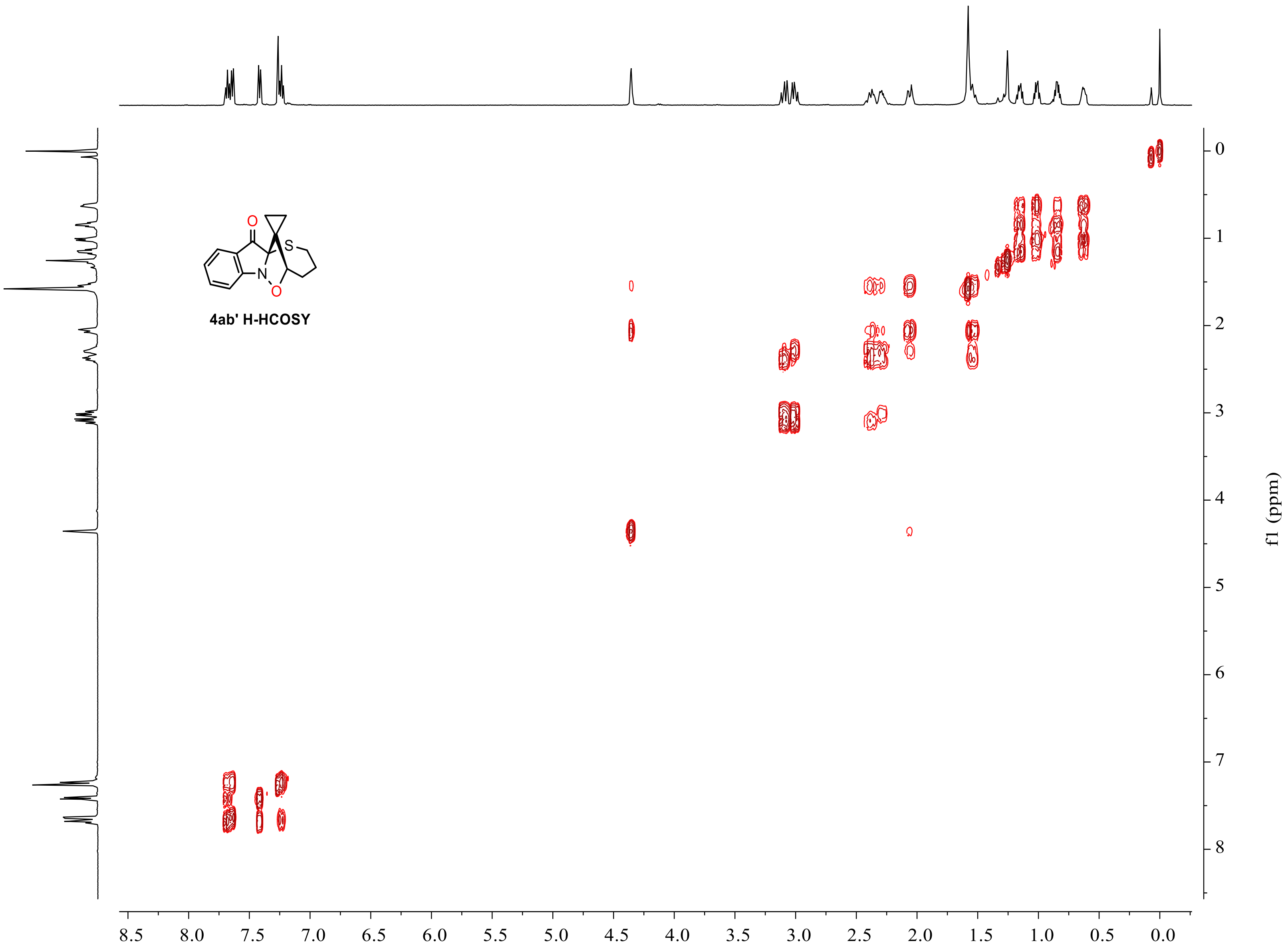

Supplementary Figure 234. H-H COSY ($CDCl_3$, 298 K) spectrum for **4ab'**

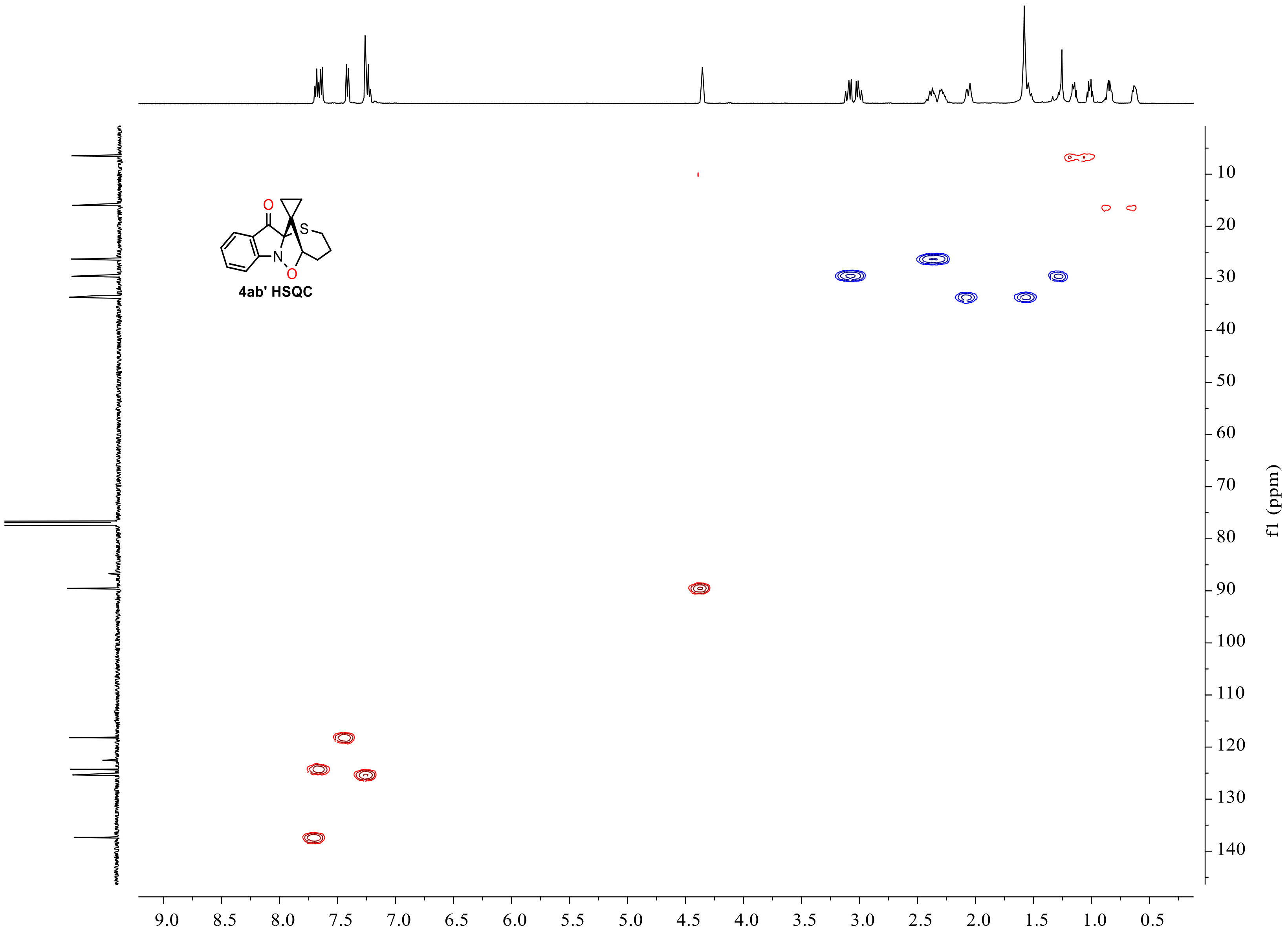

Supplementary Figure 235. HSQC ($CDCl_3$, 298 K) spectrum for **4ab’**

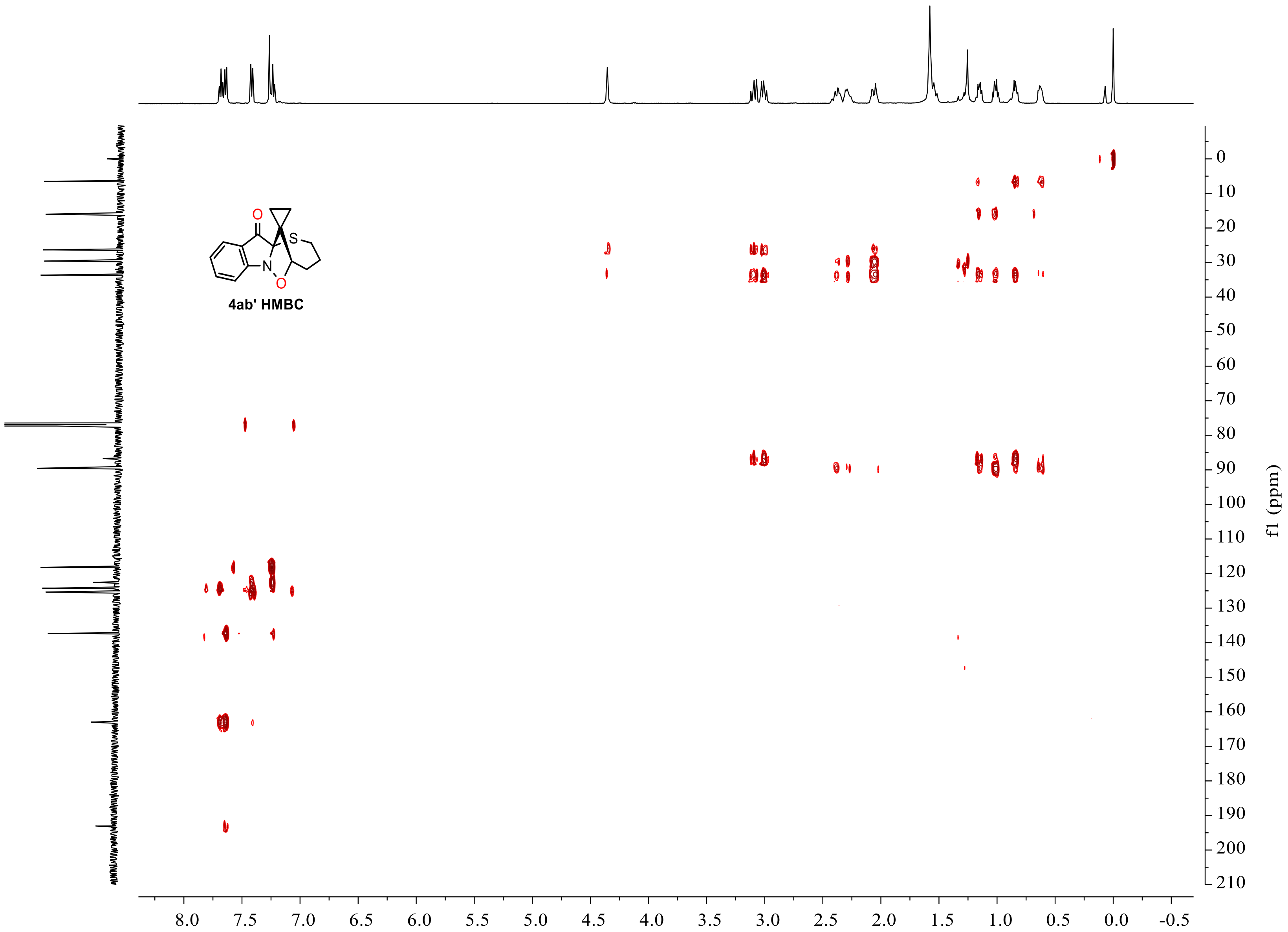

Supplementary Figure 236. HMBC ($CDCl_3$, 298 K) spectrum for **4ab'**

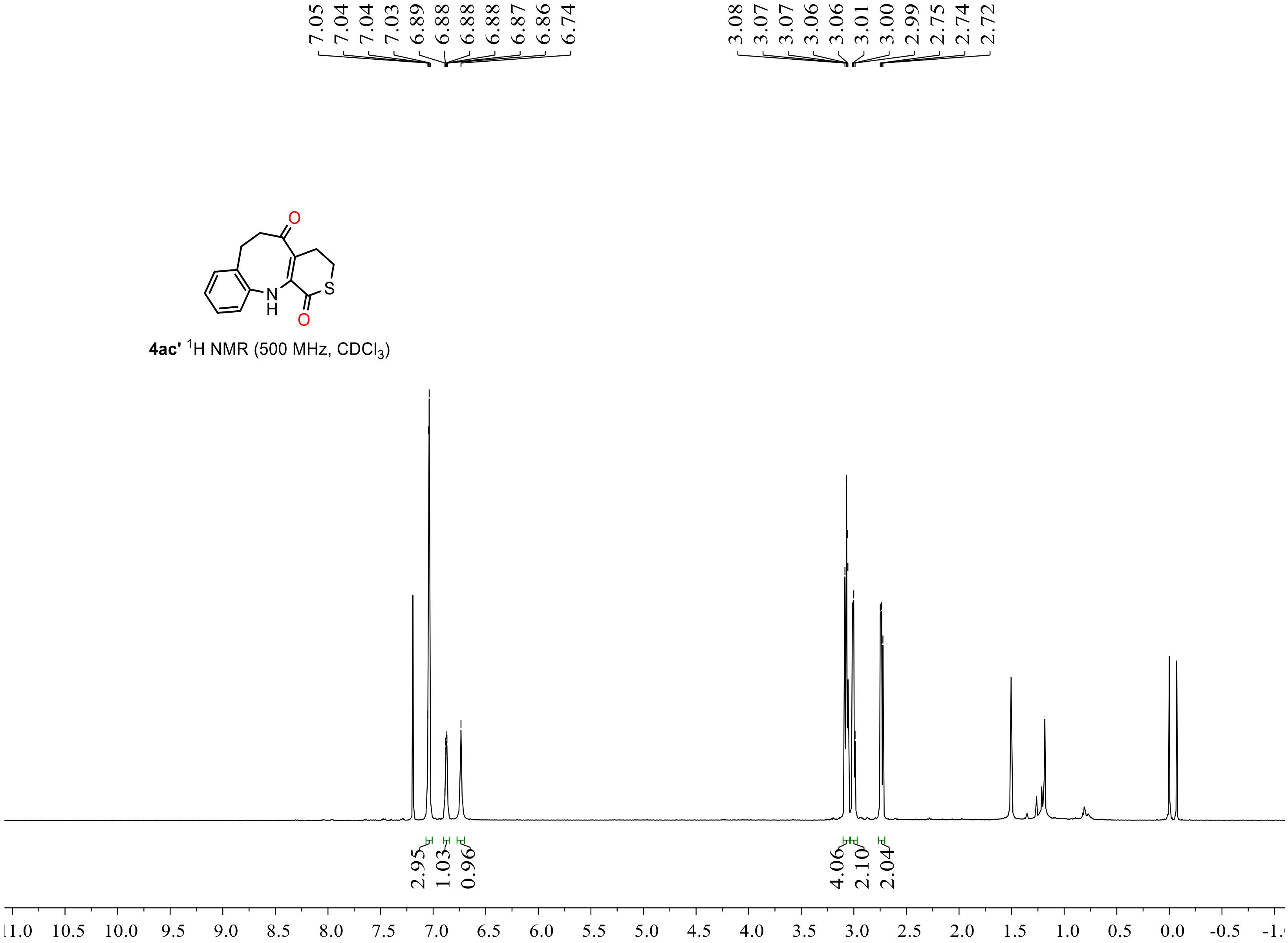

Supplementary Figure 237. ^{1}H NMR ($CDCl_3$, 500 MHz, 298 K) spectrum for **4ac’**

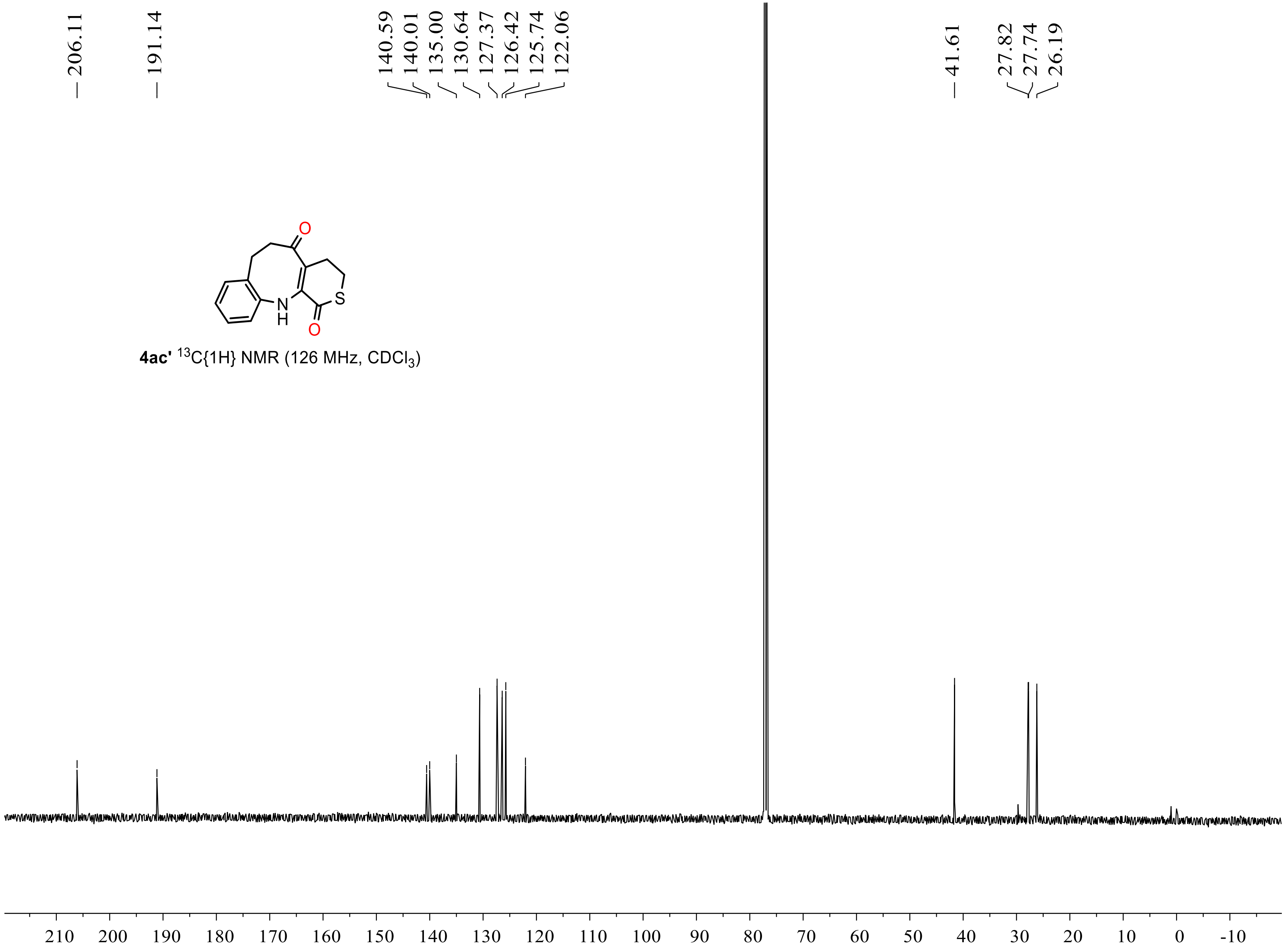

Supplementary Figure 238. ^{13}C NMR ($CDCl_3$, 126 MHz, 298 K) spectrum for **4ac’**

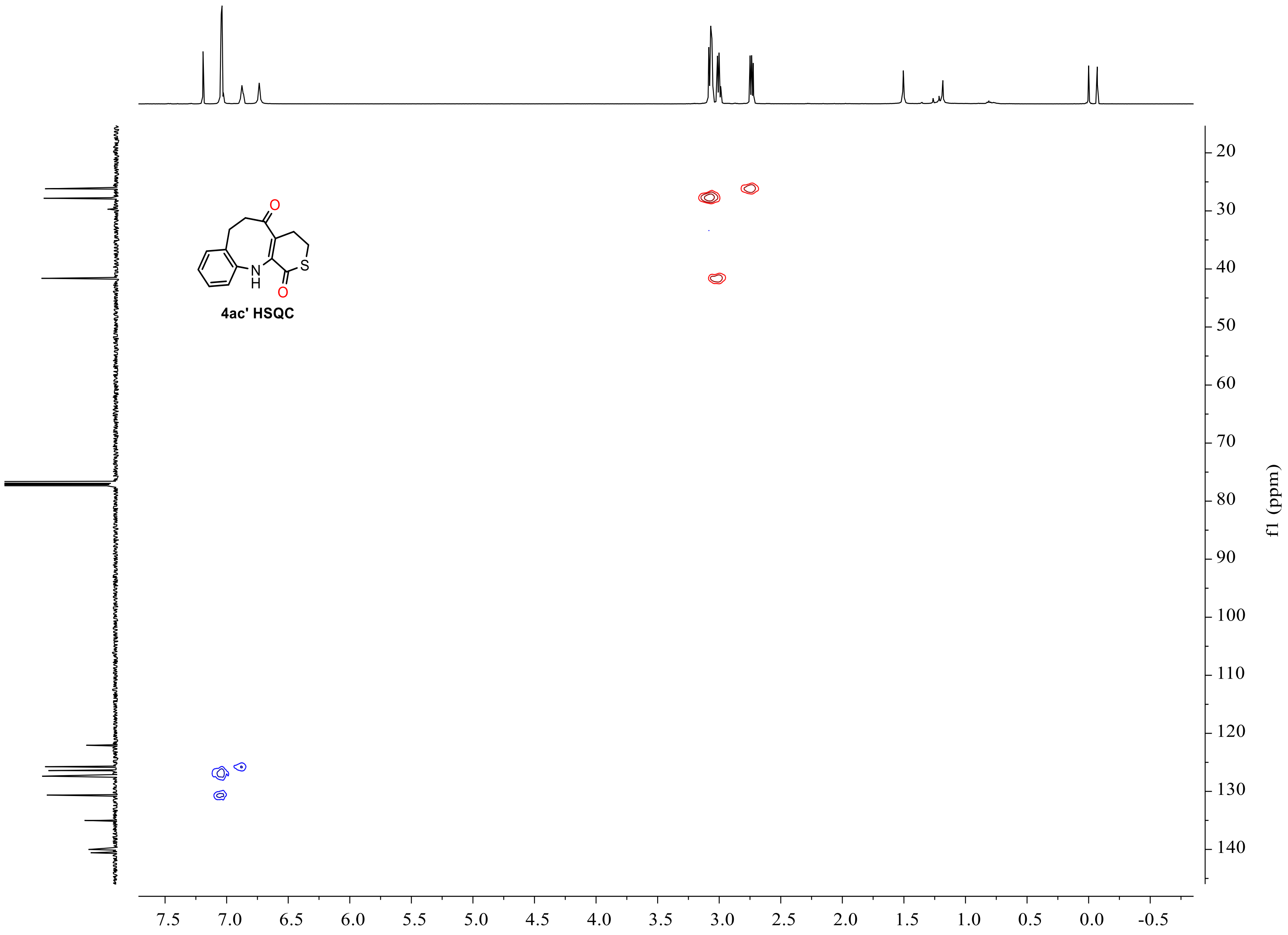

Supplementary Figure 239. HSQC ($CDCl_3$, 298 K) spectrum for **4ac'**

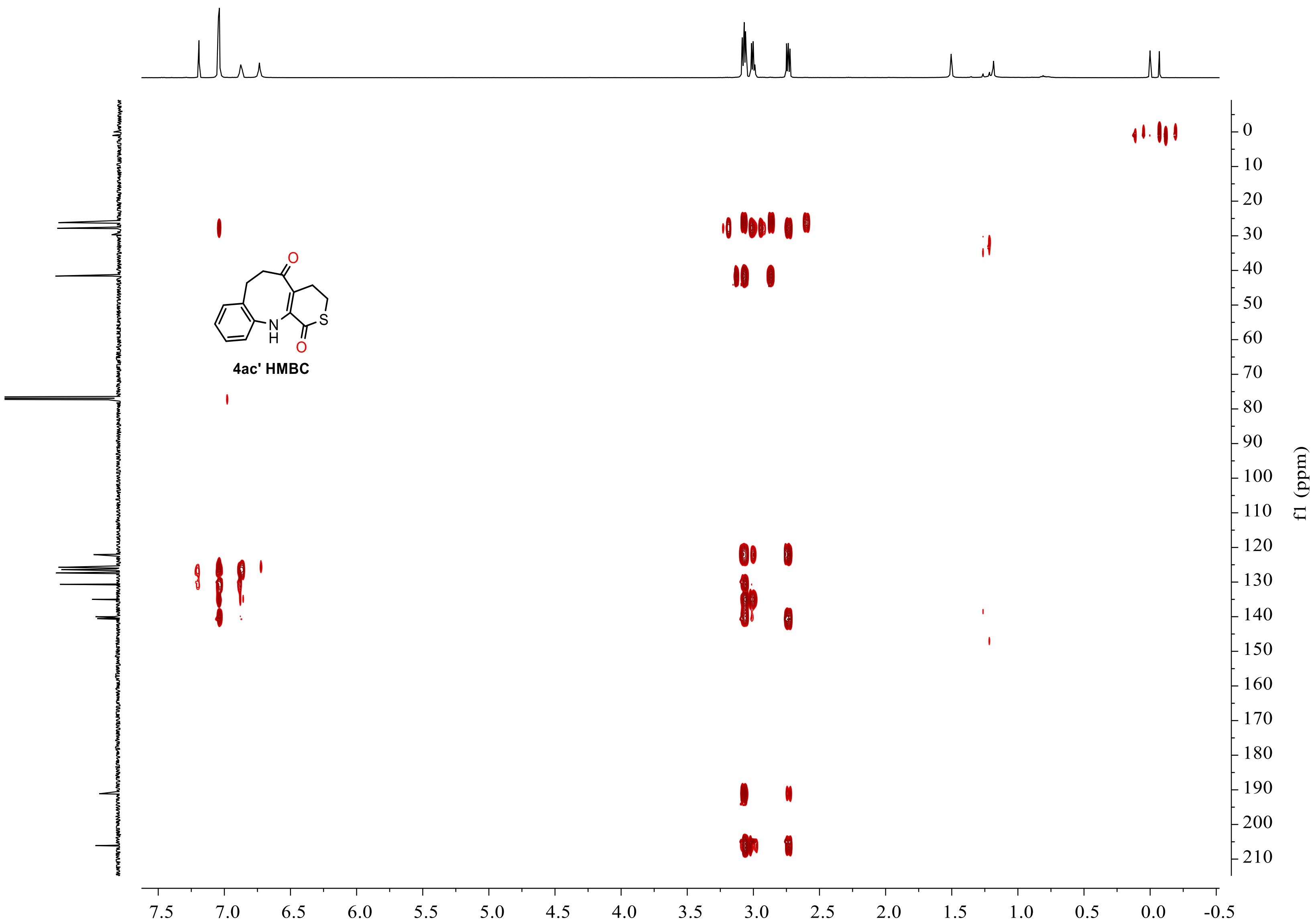

Supplementary Figure 240. HMBC ($CDCl_3$, 298 K) spectrum for **4aC'**

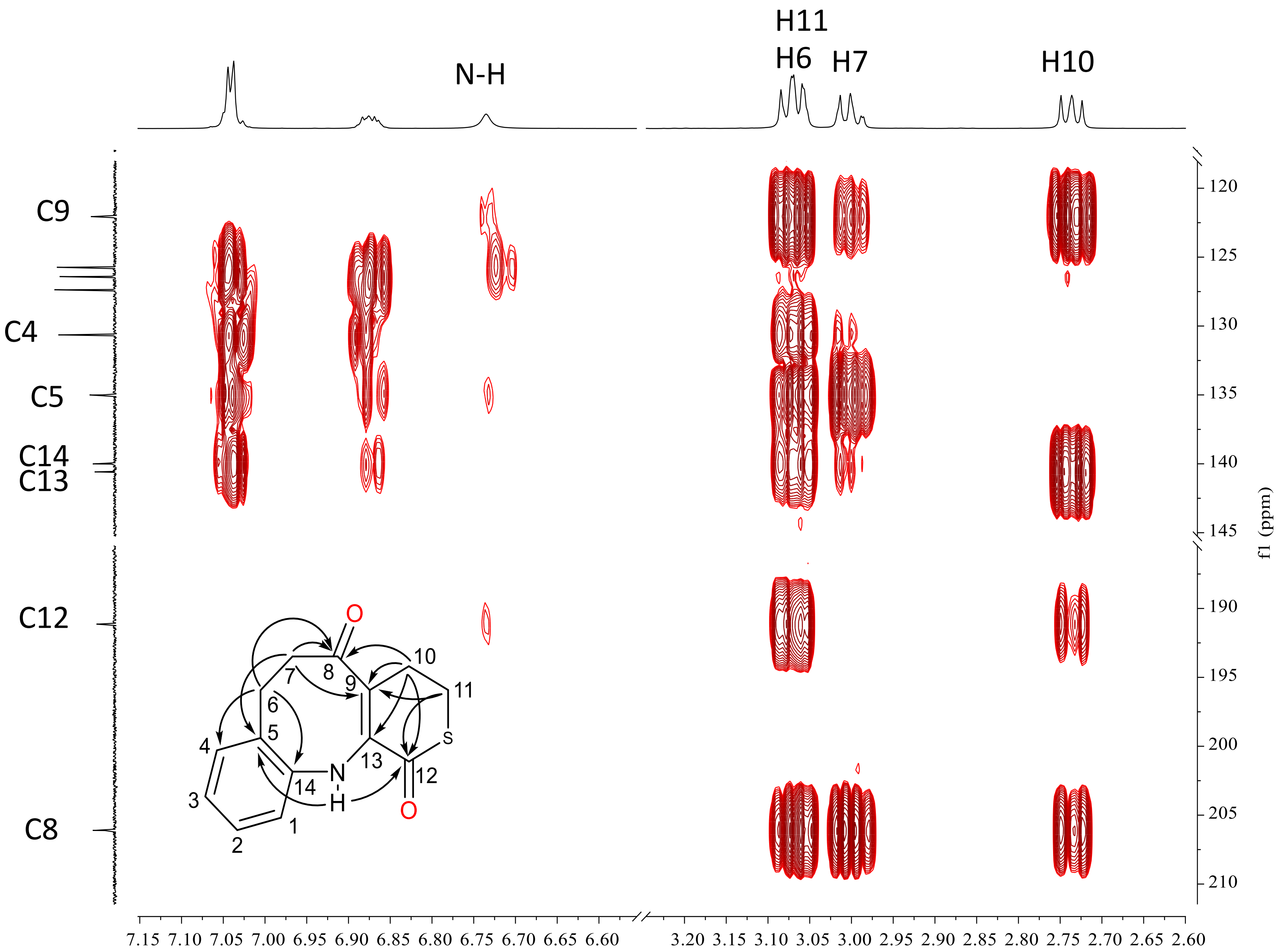

Supplementary Figure 241. HMBC ($CDCl_3$, 298 K) spectrum for **4ac'**

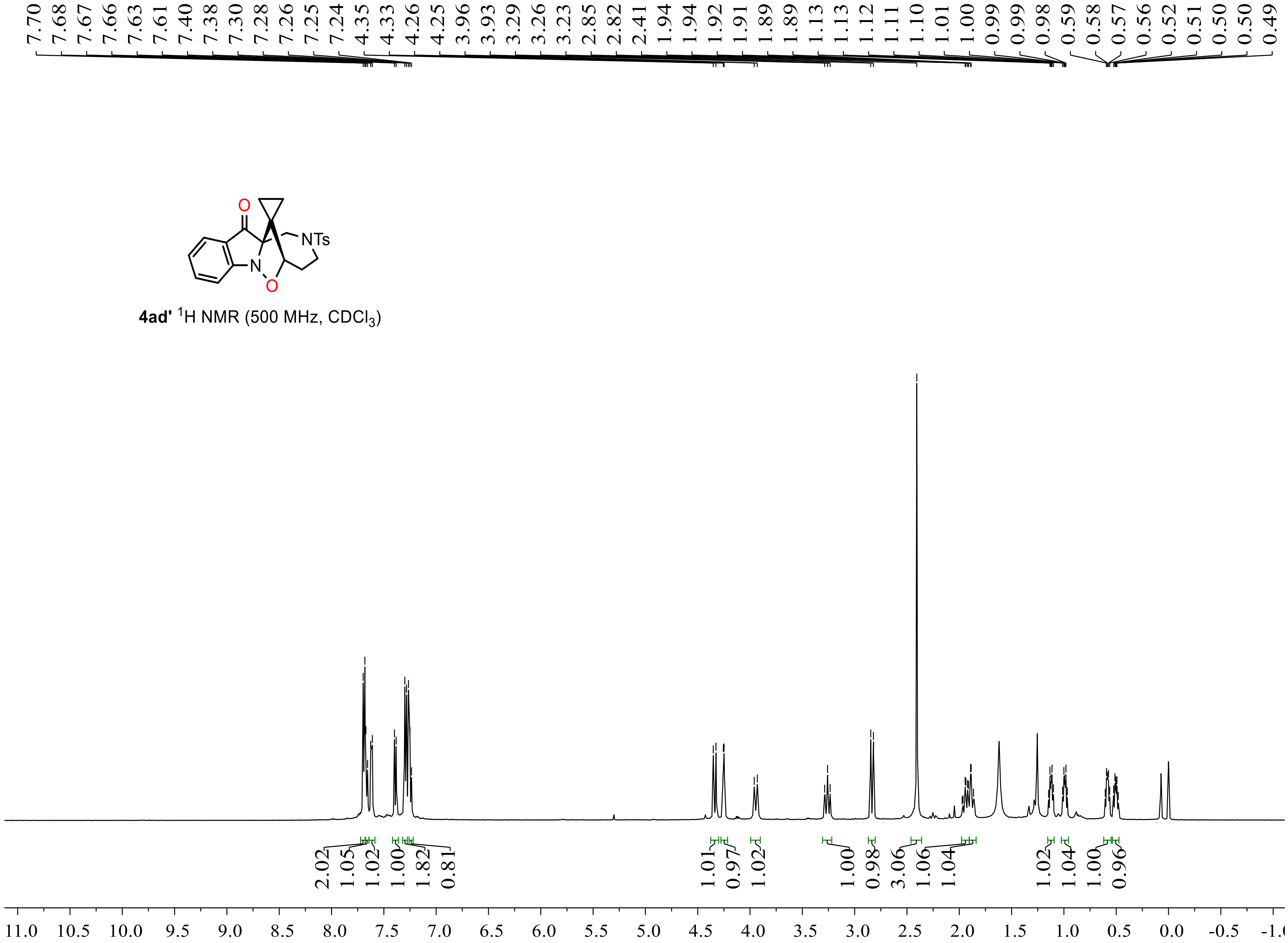

Supplementary Figure 242. ^{1}H NMR ($CDCl_3$, 500 MHz, 298 K) spectrum for **4ad’**

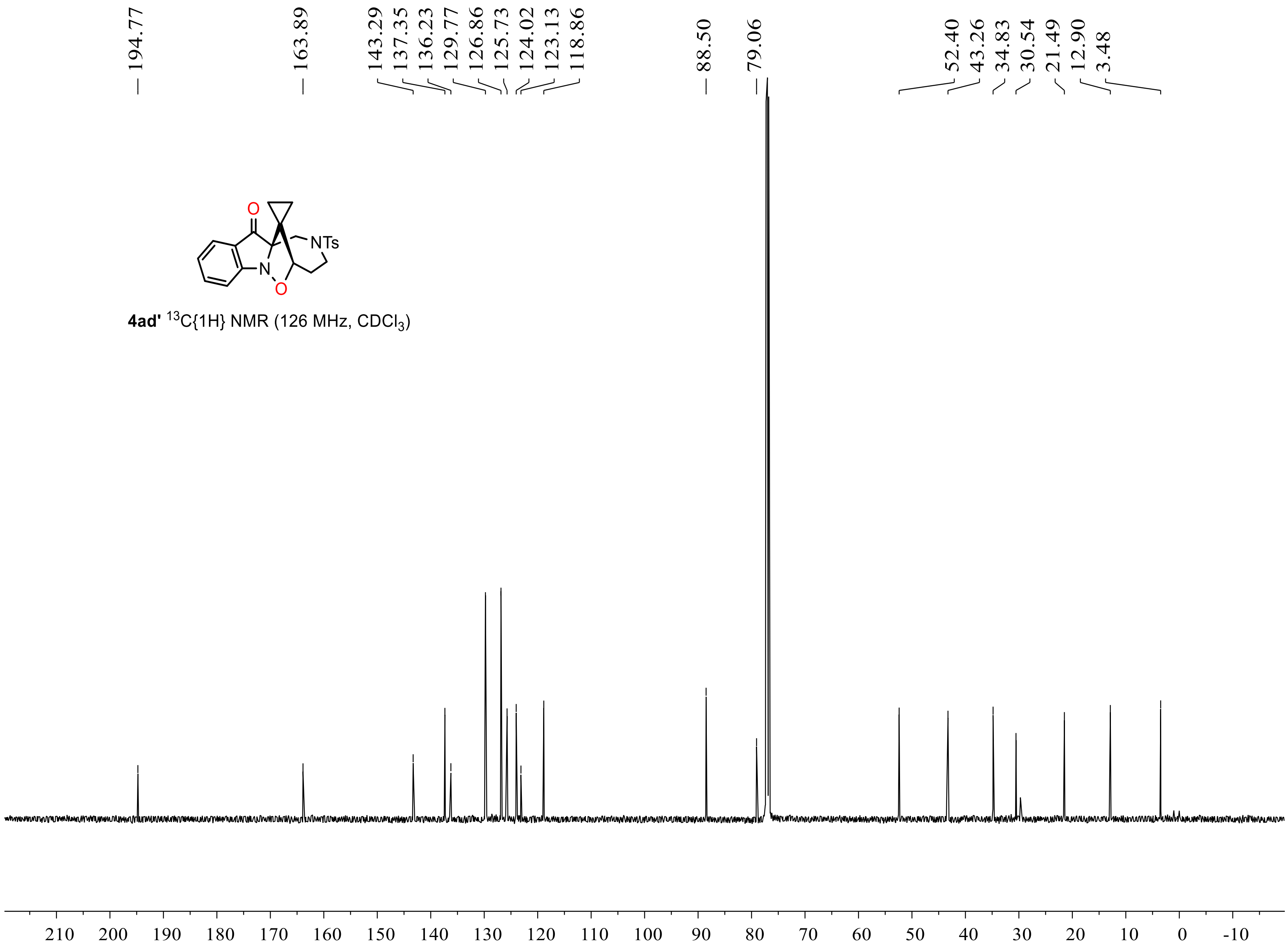

Supplementary Figure 243. ^{13}C NMR ($CDCl_3$, 126 MHz, 298 K) spectrum for **4ad’**

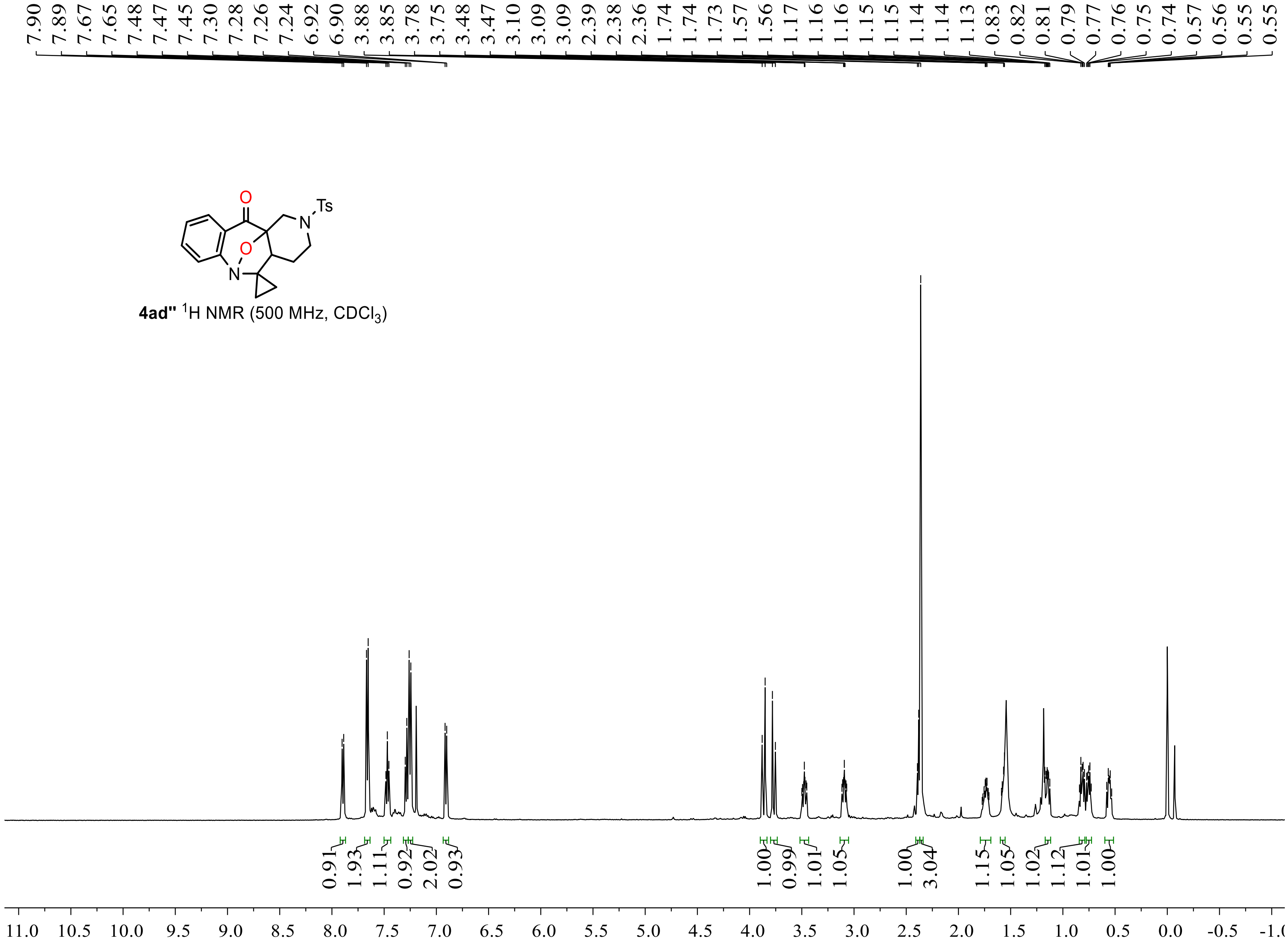

Supplementary Figure 244. ^{1}H NMR ($CDCl_3$, 500 MHz, 298 K) spectrum for **4ad''**

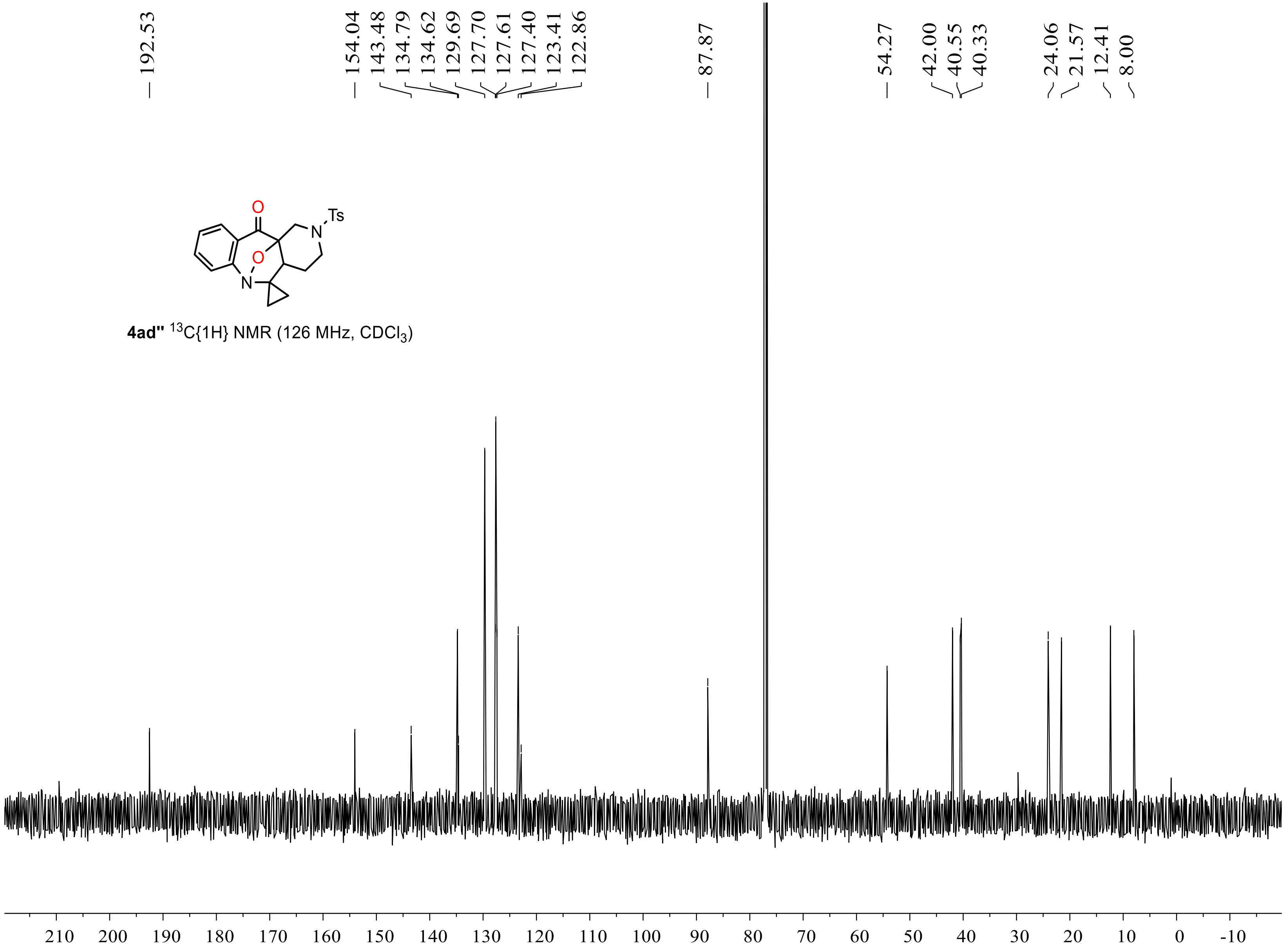

Supplementary Figure 245. ^{13}C NMR ($CDCl_3$, 126 MHz, 298 K) spectrum for **4ad''**

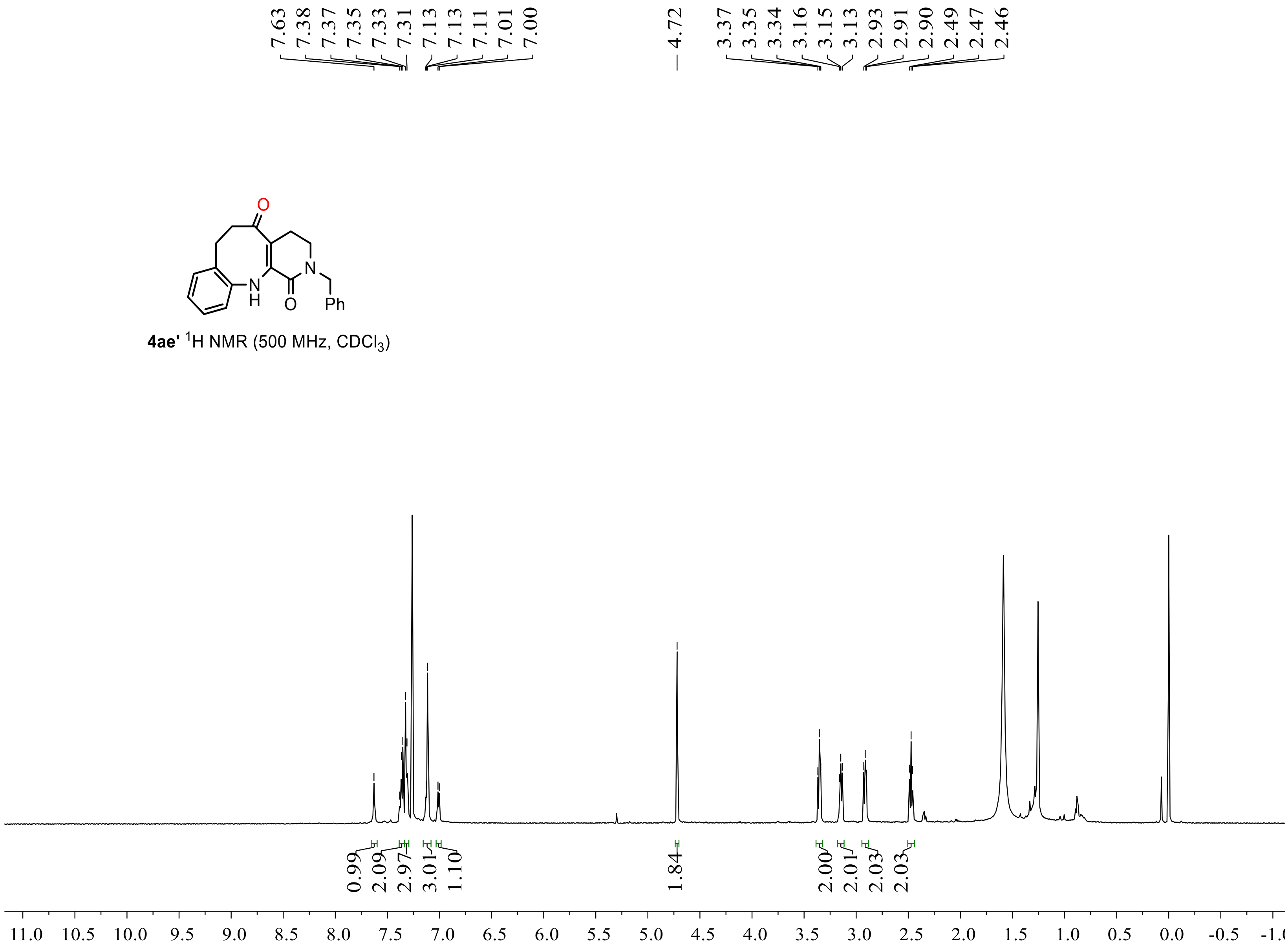

4ae' ^{1}H NMR (500 MHz, $CDCl_3$)

Supplementary Figure 246. ^{1}H NMR ($CDCl_3$, 500 MHz, 298 K) spectrum for **4ae'**

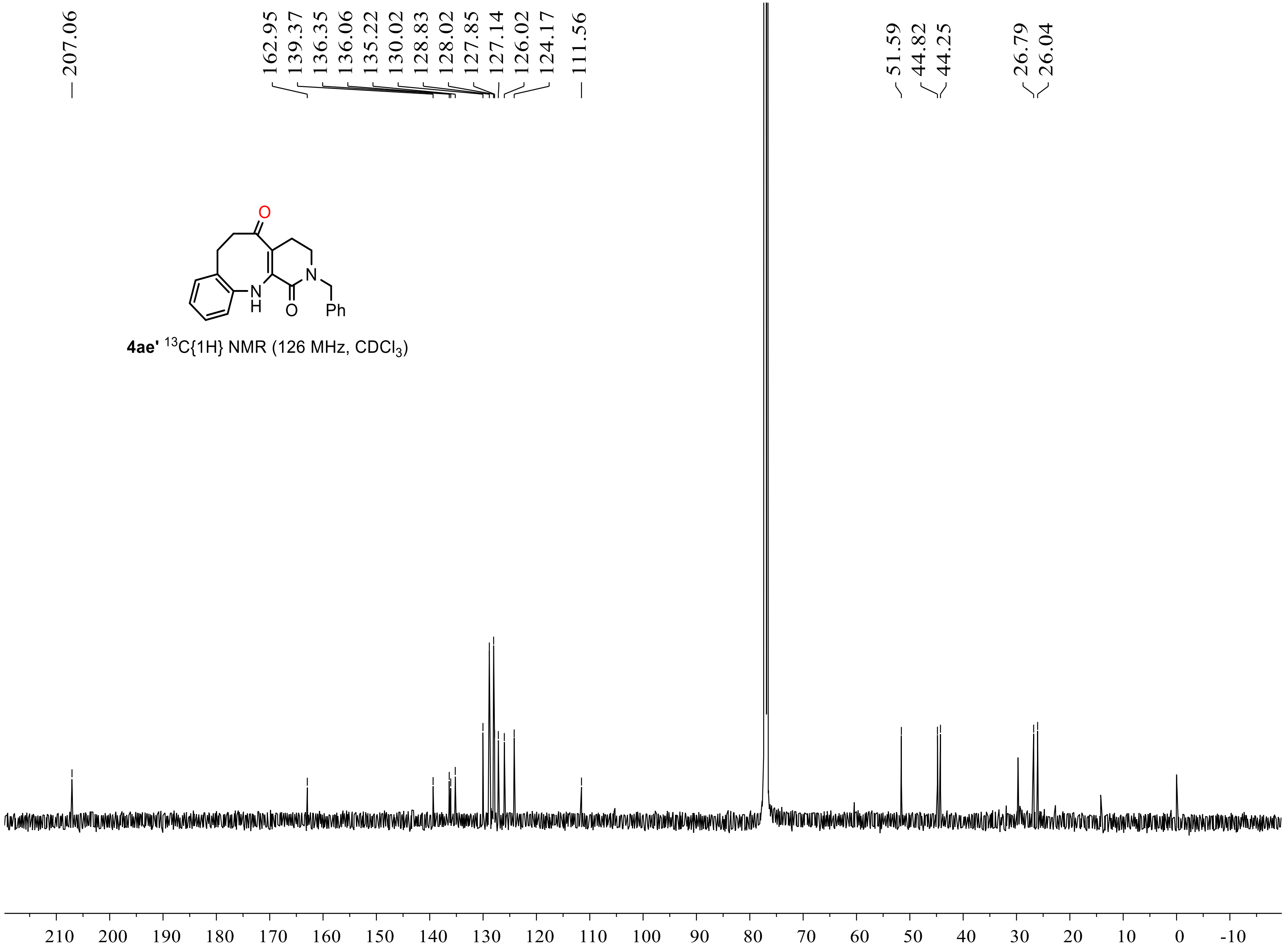

Supplementary Figure 247. ^{13}C NMR ($CDCl_3$, 126 MHz, 298 K) spectrum for **4ae'**

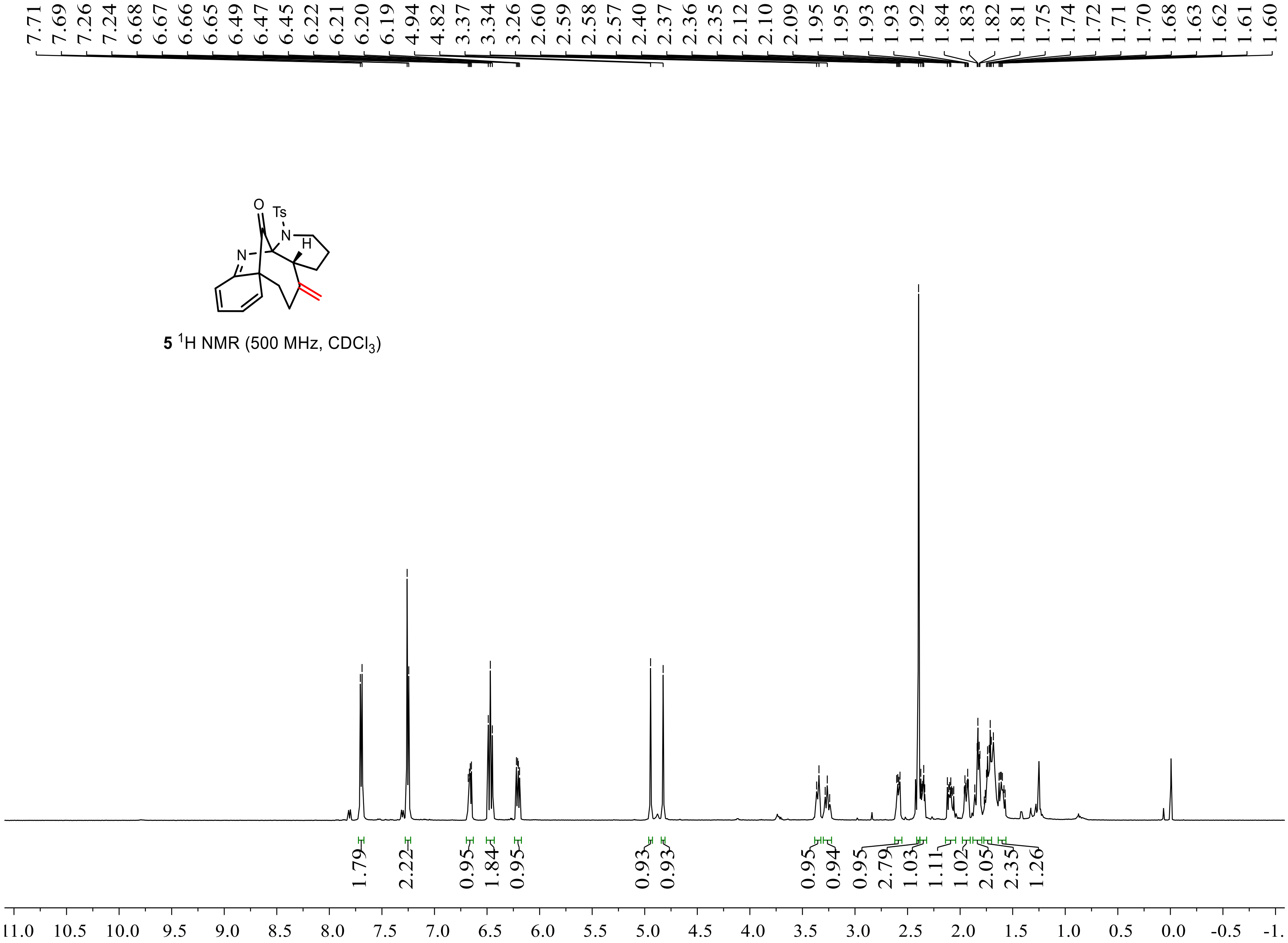

Supplementary Figure 248. ^{1}H NMR (CDCl$_3$, 500 MHz, 298 K) spectrum for **5**

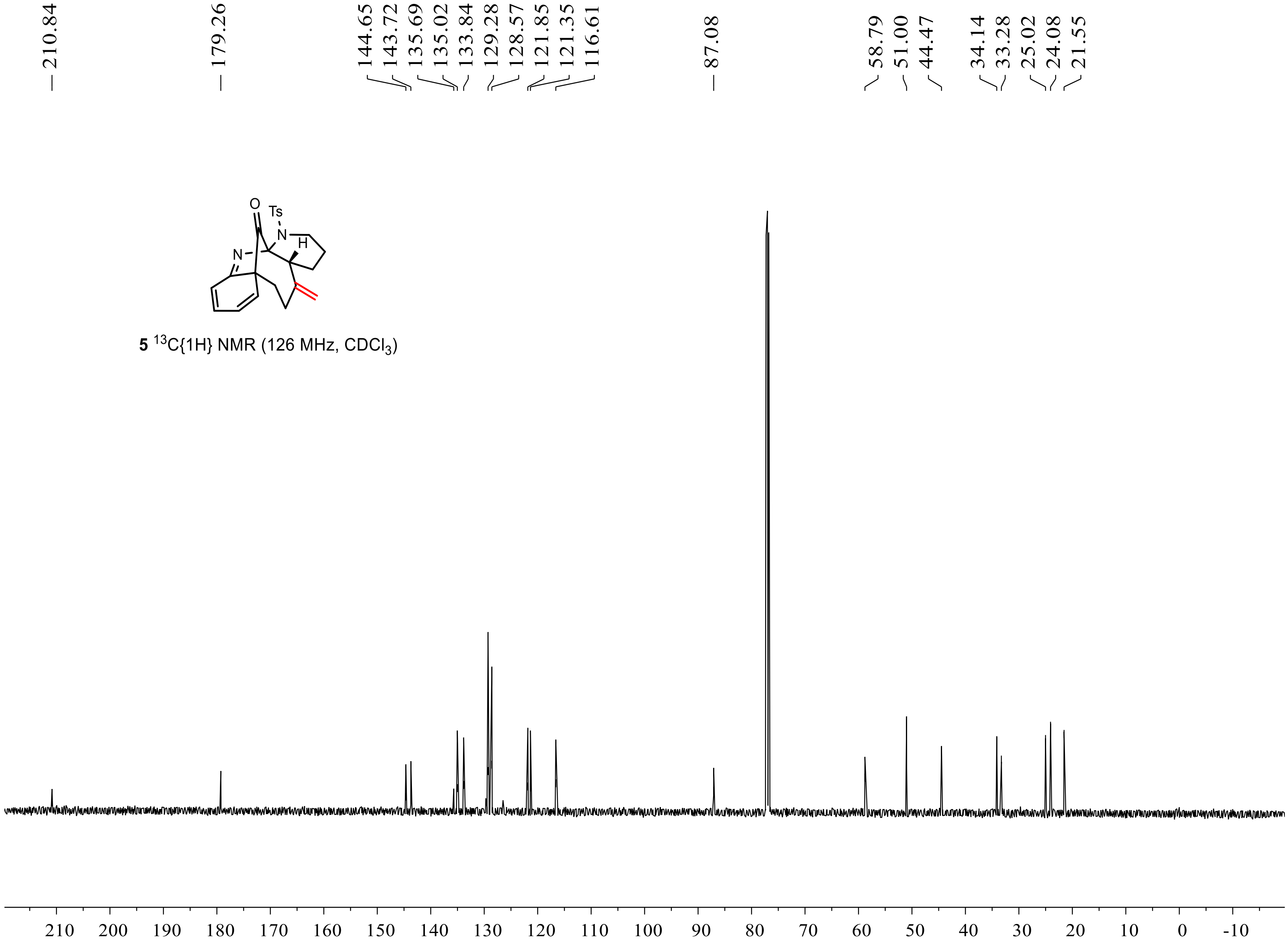

Supplementary Figure 249. ^{13}C NMR ($CDCl_3$, 126 MHz, 298 K) spectrum for **5**

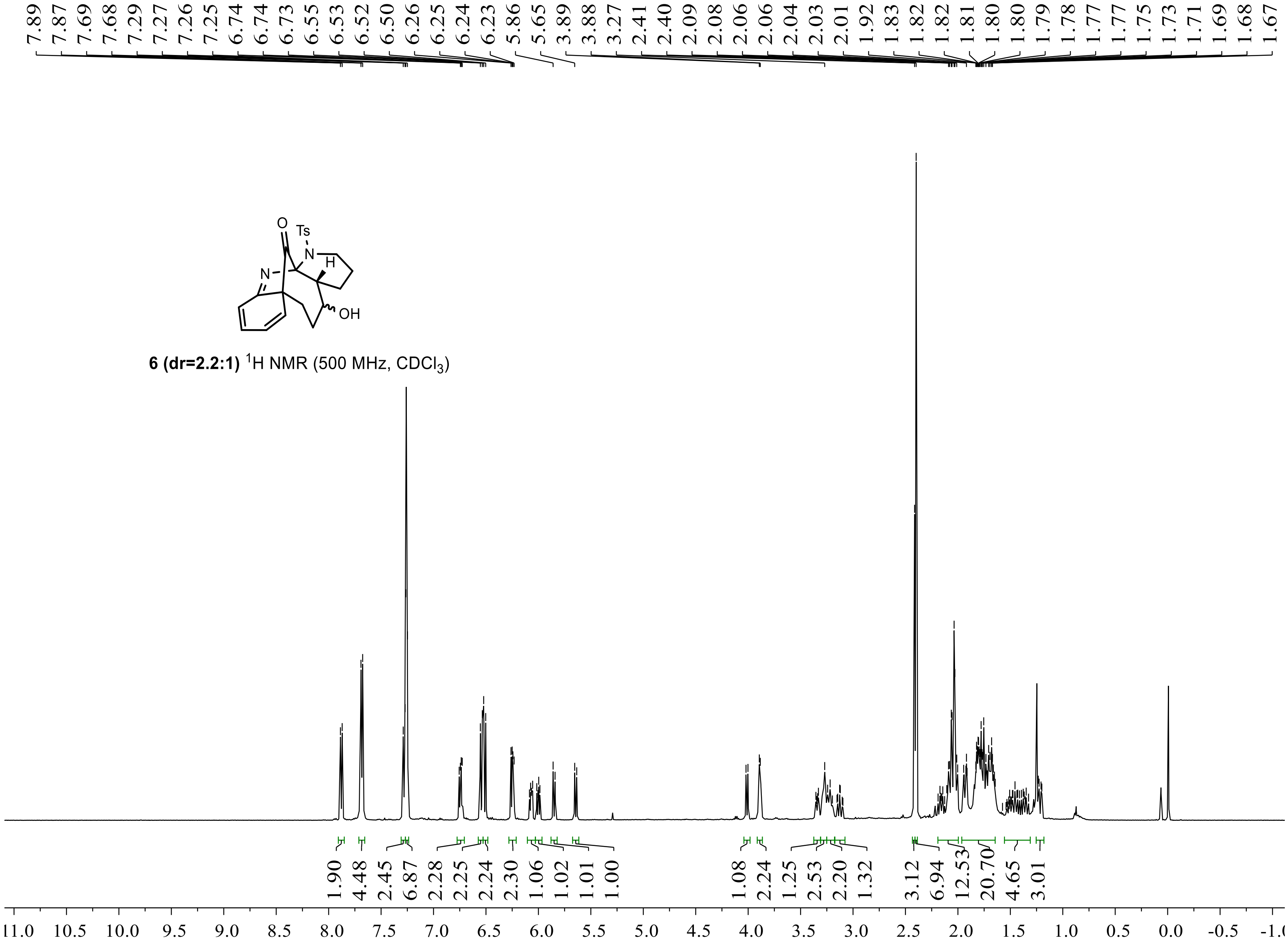

Supplementary Figure 250. ^{1}H NMR ($CDCl_3$, 500 MHz, 298 K) spectrum for **6**

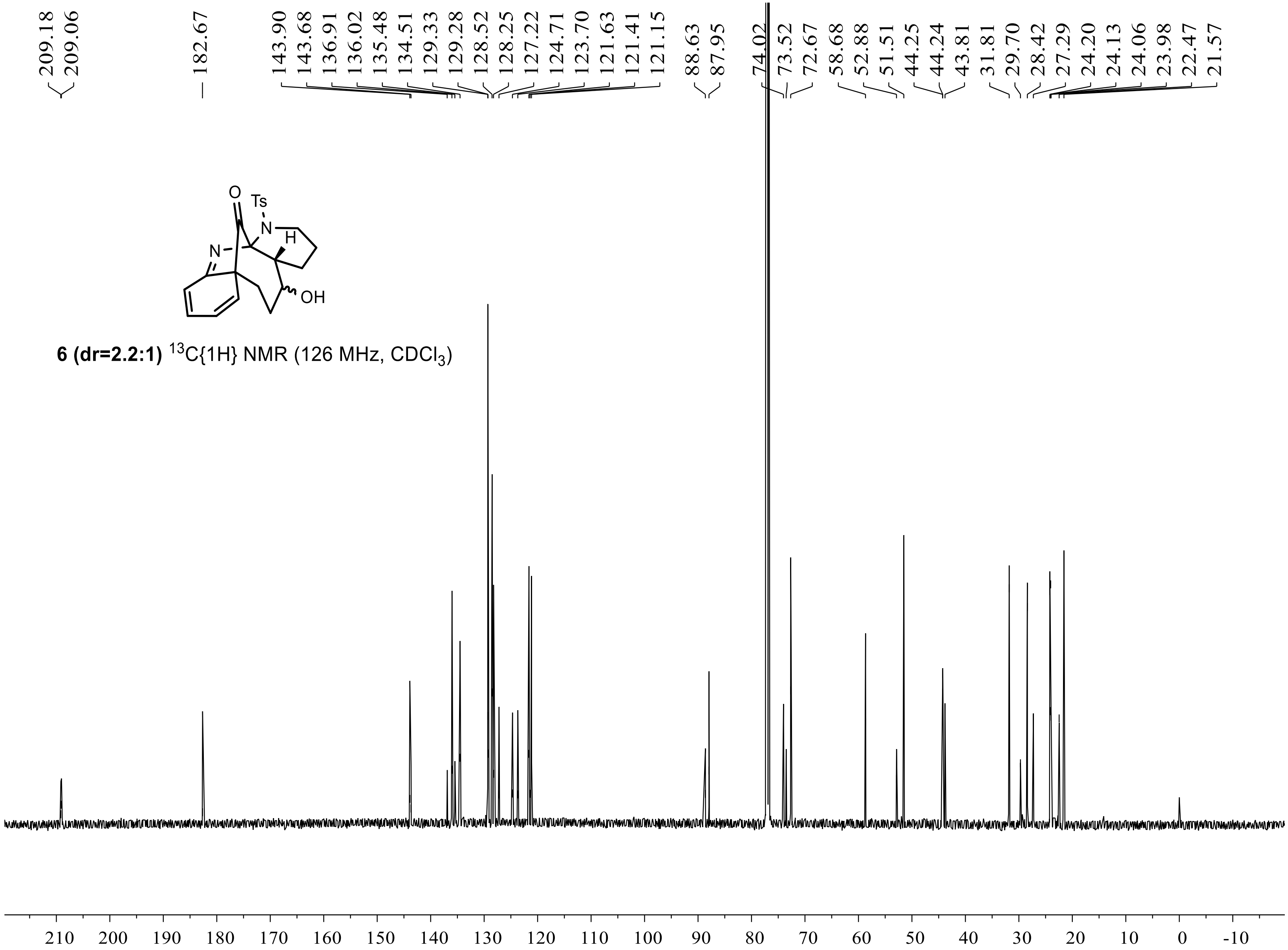

Supplementary Figure 251. ^{13}C NMR ($CDCl_3$, 126 MHz, 298 K) spectrum for **6**

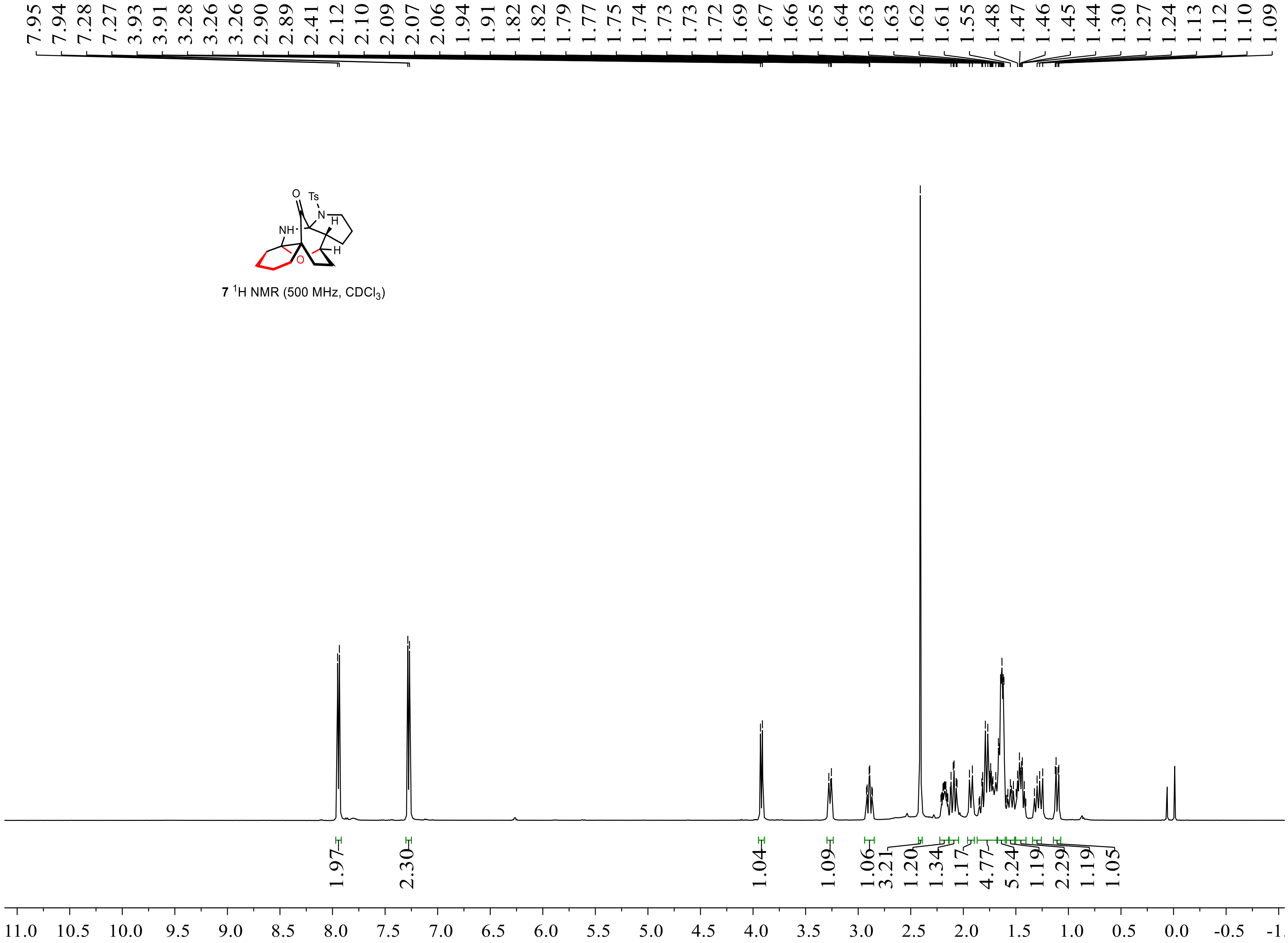

Supplementary Figure 252. ^{1}H NMR (CDCl$_3$, 500 MHz, 298 K) spectrum for **7**

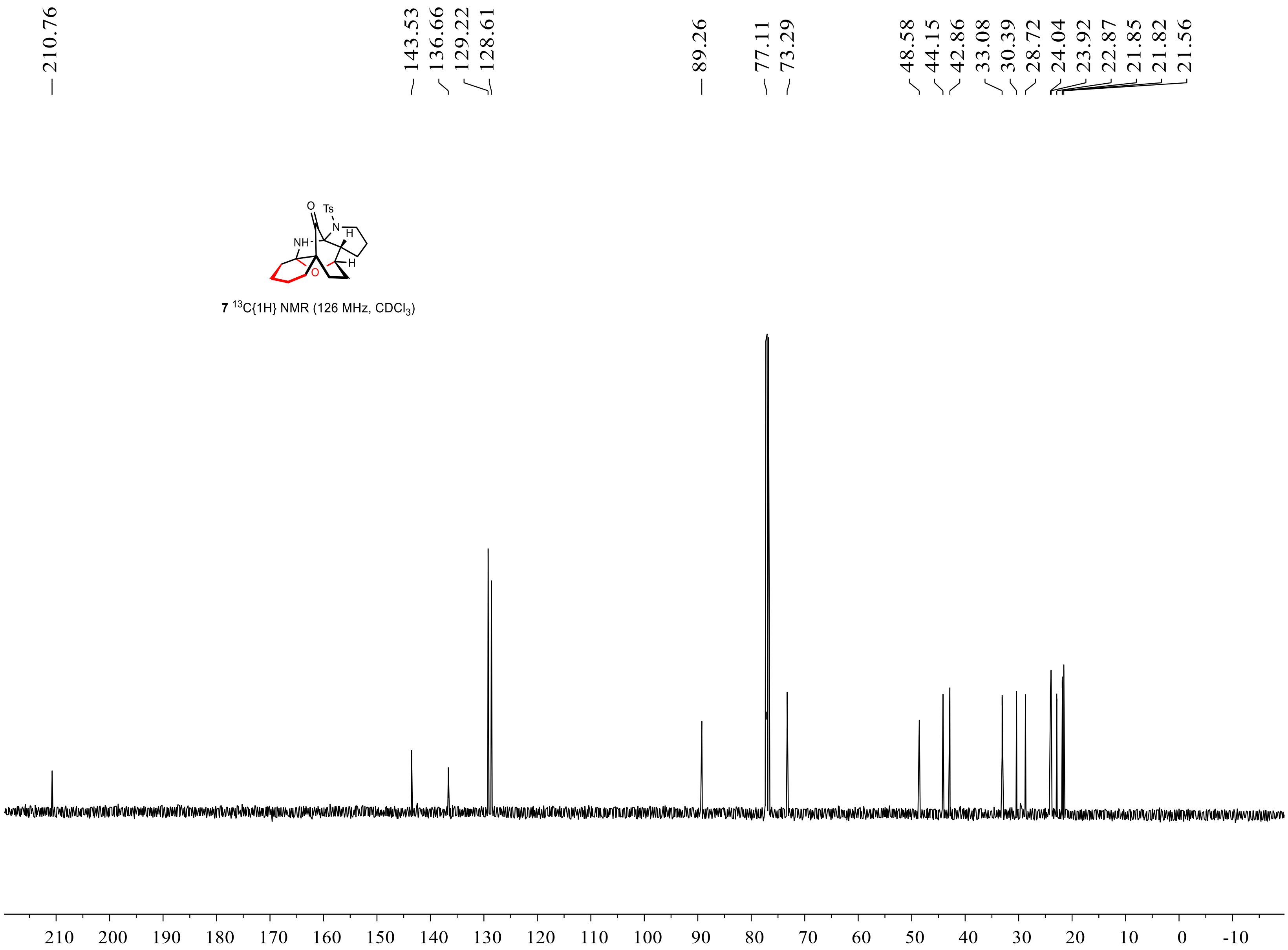

Supplementary Figure 253. ^{13}C NMR ($CDCl_3$, 126 MHz, 298 K) spectrum for **7**

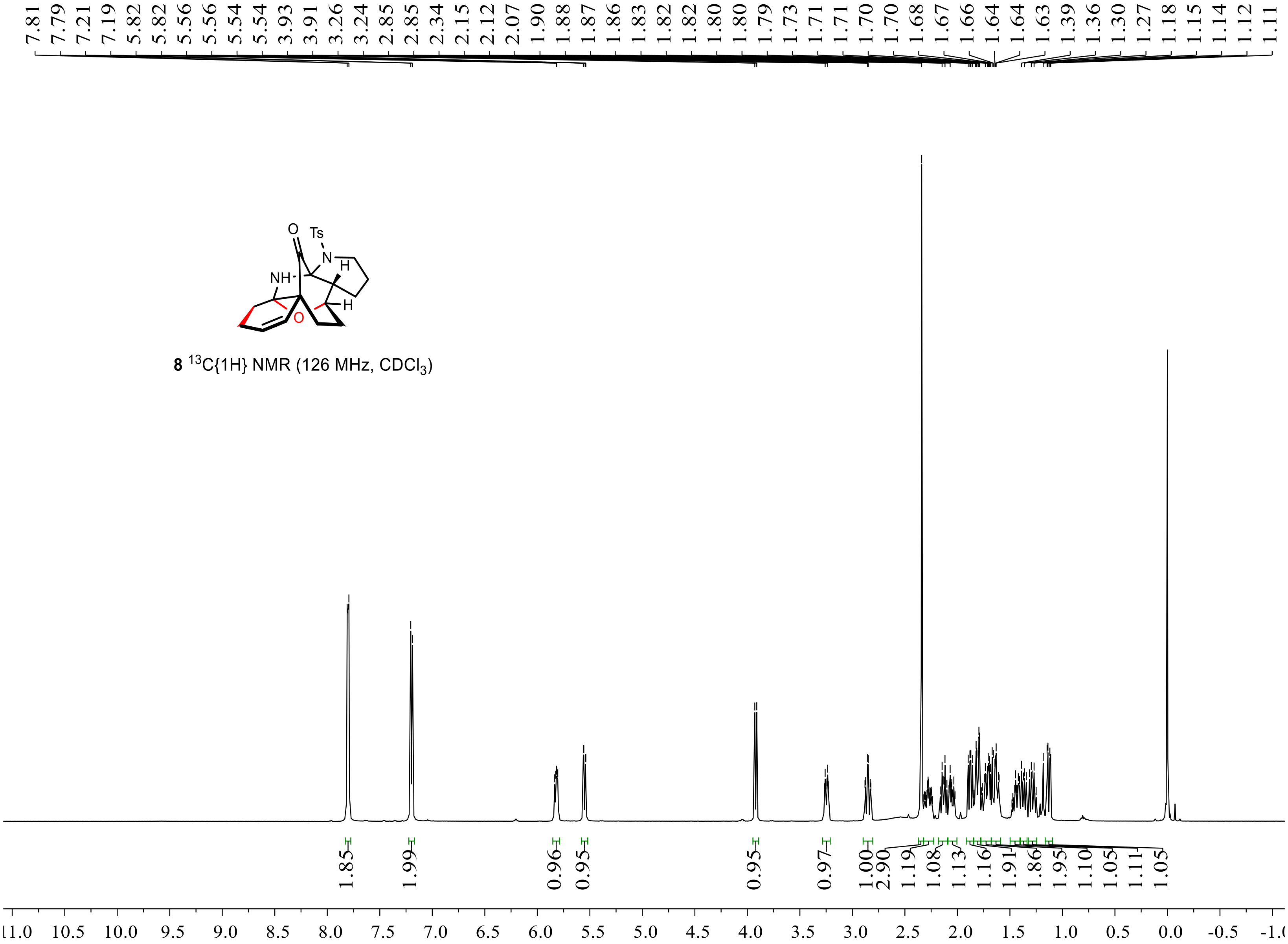

Supplementary Figure 254. ^{1}H NMR ($CDCl_3$, 500 MHz, 298 K) spectrum for **8**

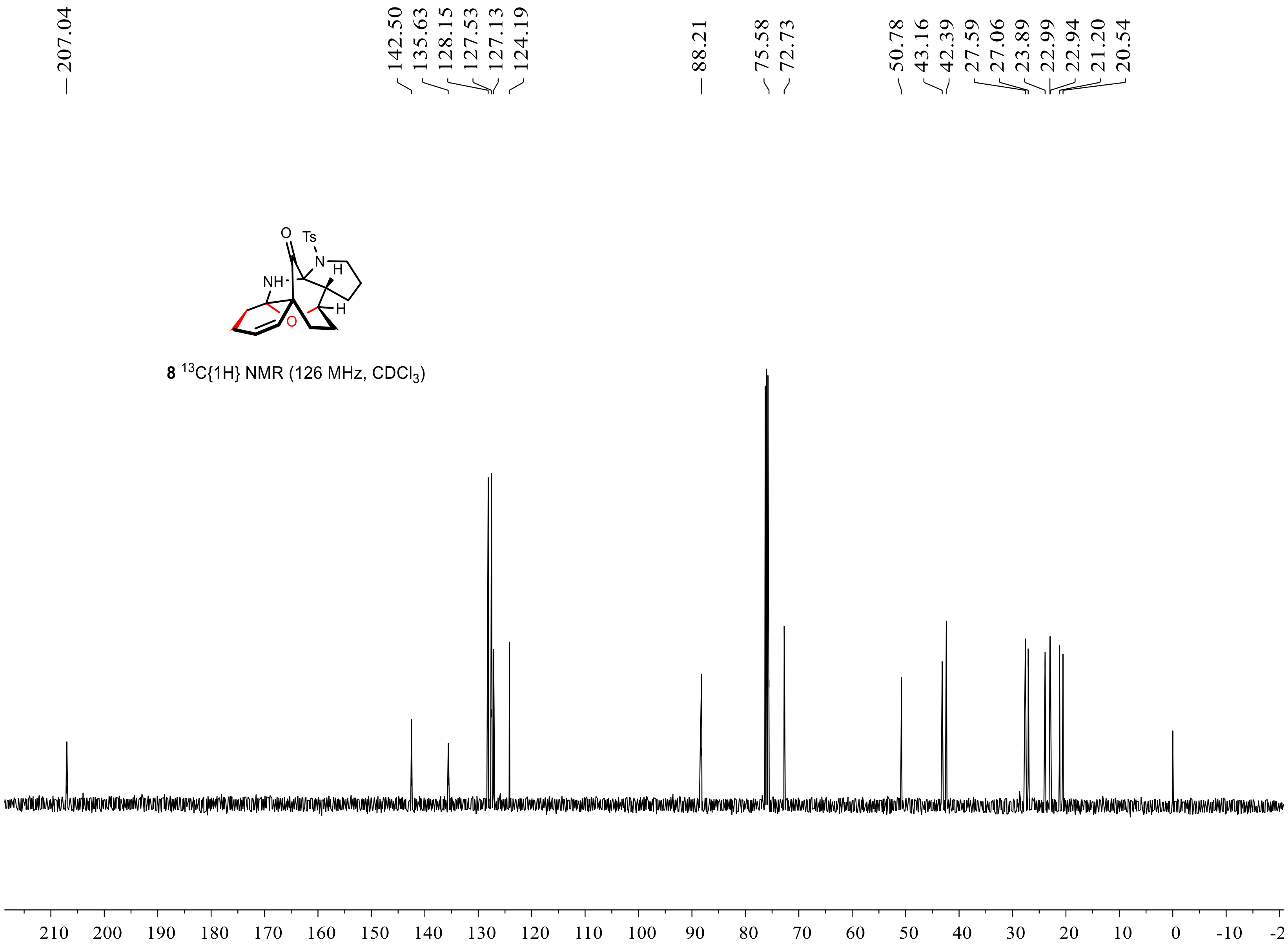

Supplementary Figure 255. ^{13}C NMR ($CDCl_3$, 126 MHz, 298 K) spectrum for **8**

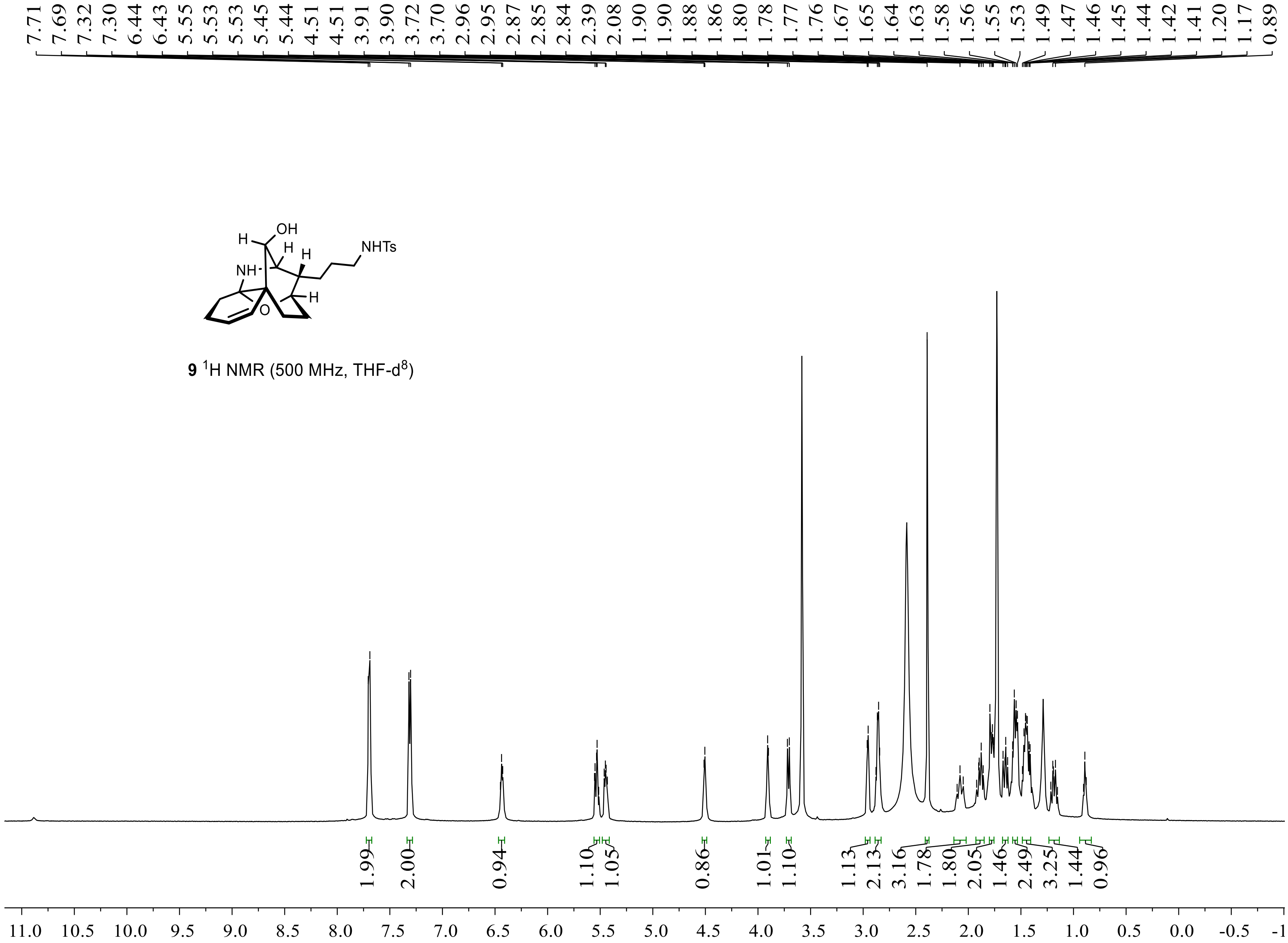

Supplementary Figure 256. ^1H NMR ($CDCl_3$, 500 MHz, 298 K) spectrum for **9**

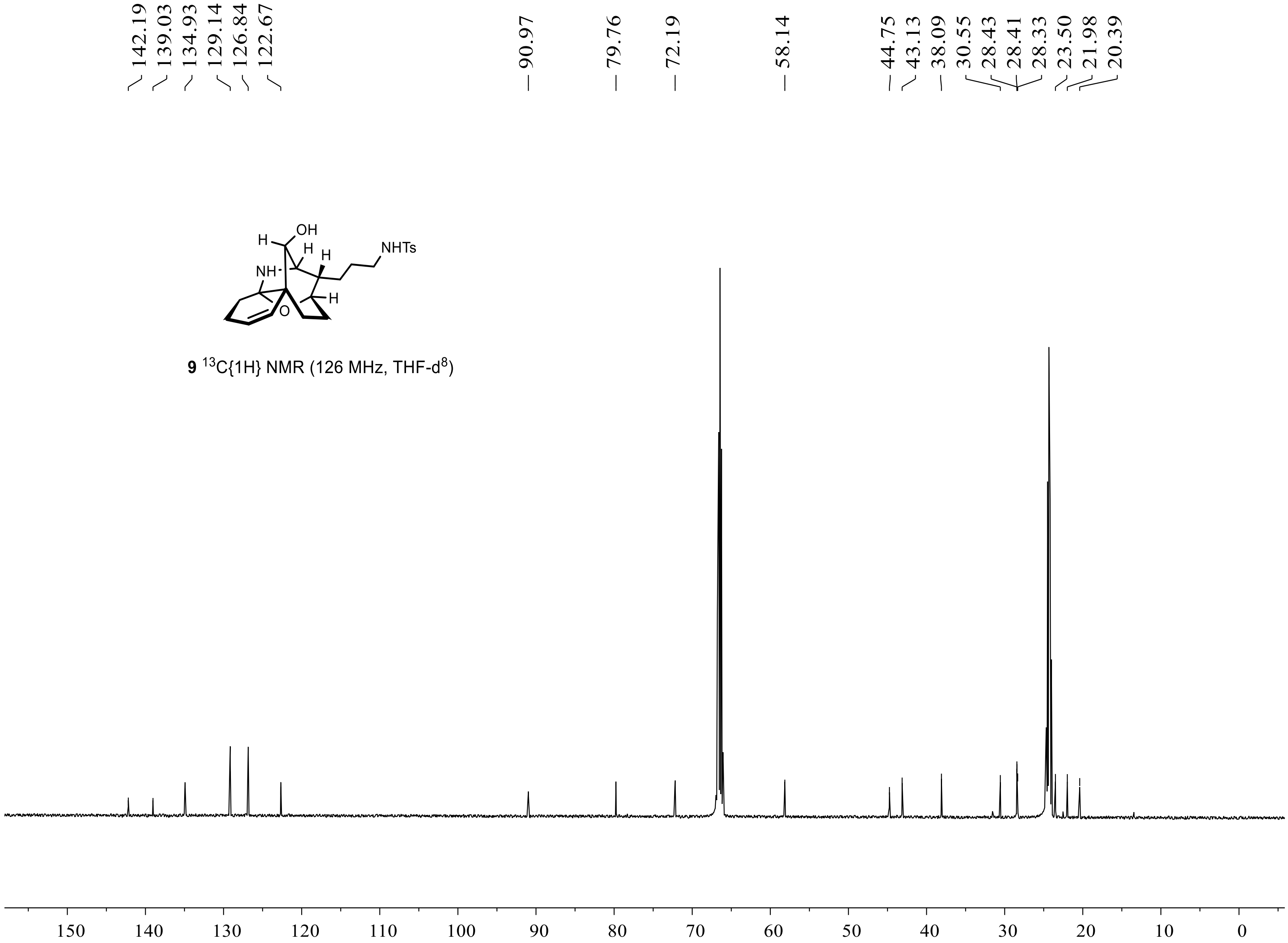

Supplementary Figure 257. ^{13}C NMR ($CDCl_3$, 126 MHz, 298 K) spectrum for **9**

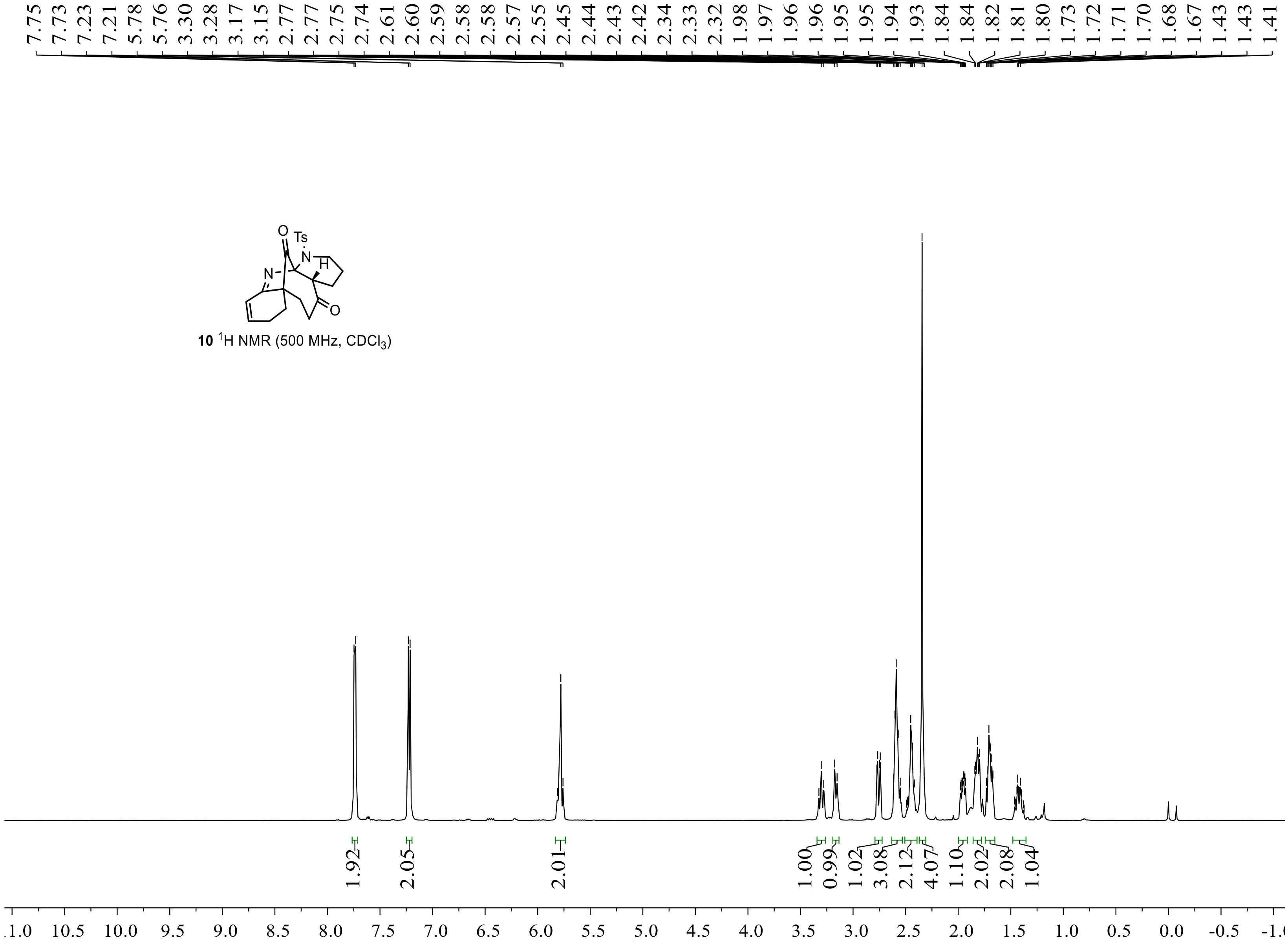

Supplementary Figure 258. ¹H NMR ($CDCl_3$, 500 MHz, 298 K) spectrum for **10**

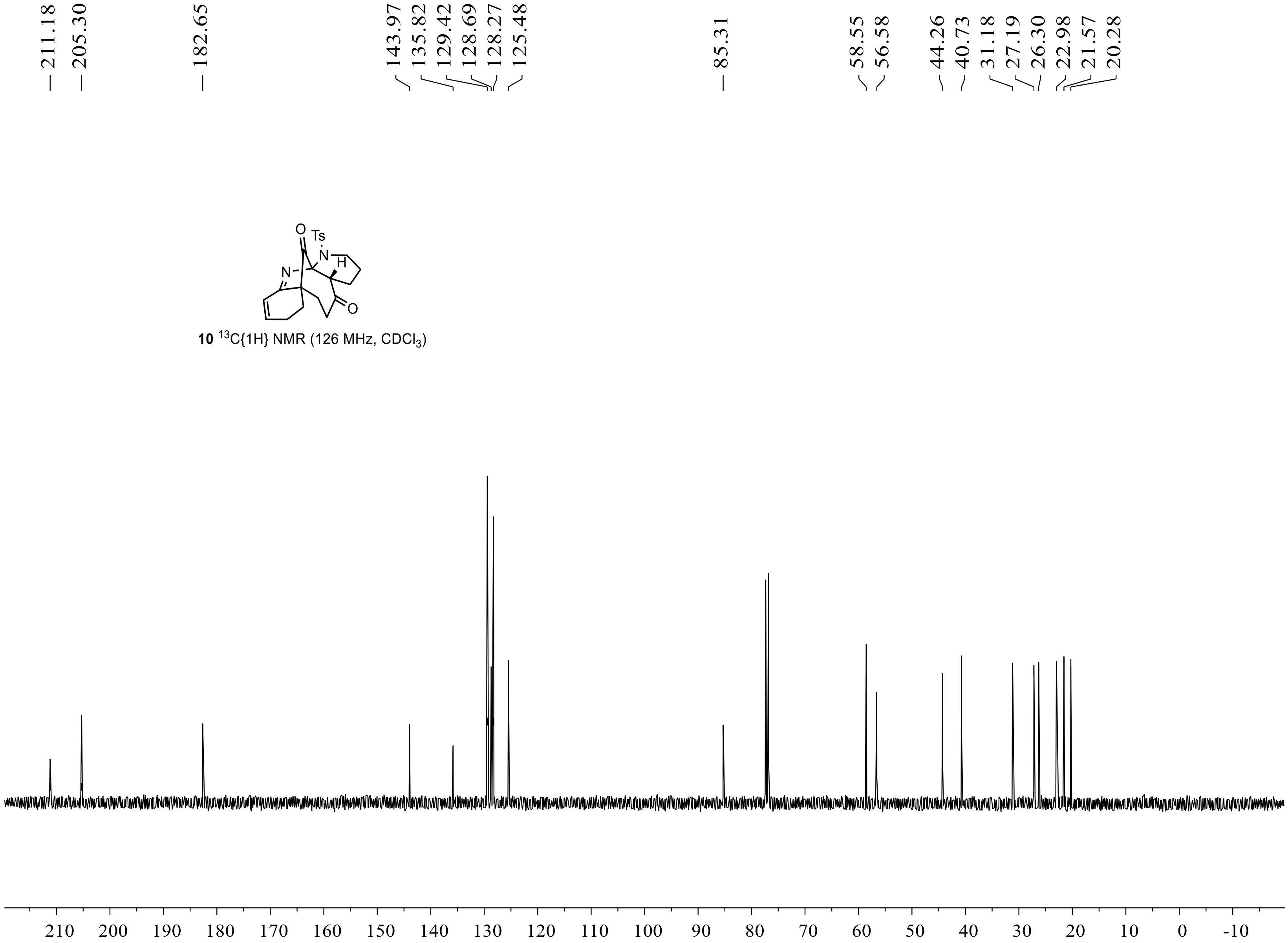

Supplementary Figure 259. ^{13}C NMR ($CDCl_3$, 126 MHz, 298 K) spectrum for **10**

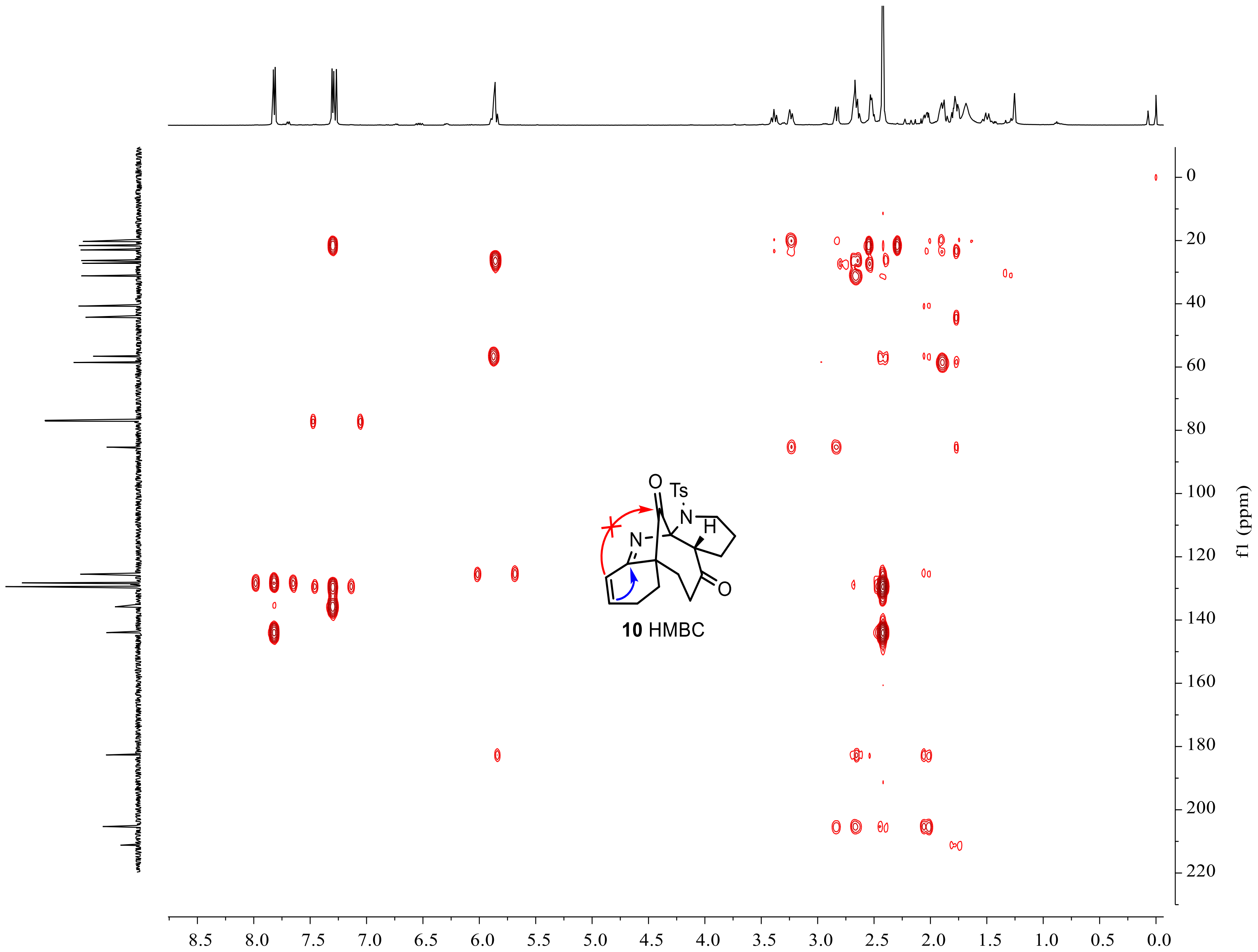

Supplementary Figure 260. HMBC ($CDCl_3$, 298 K) spectrum for **10**

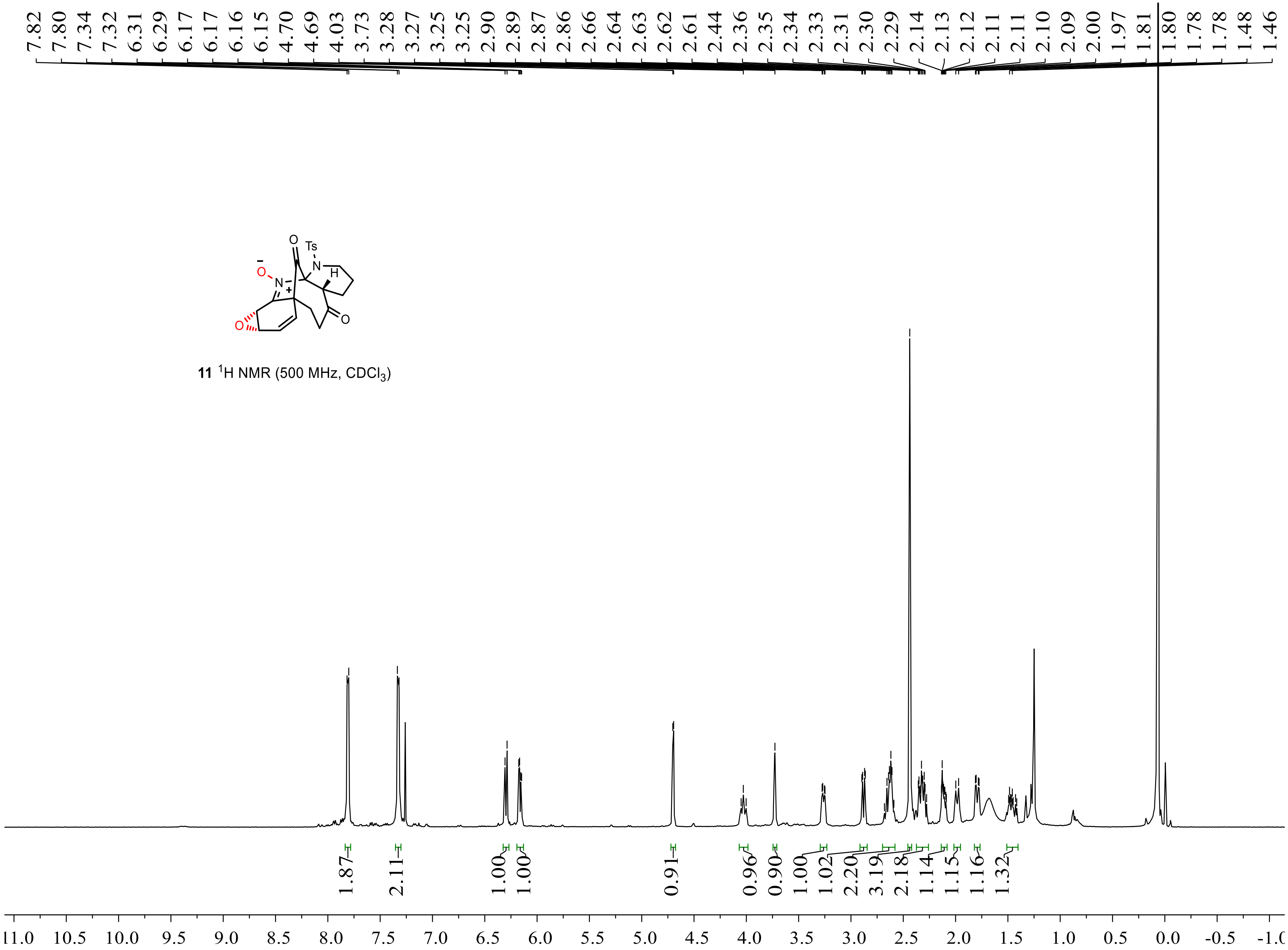

Supplementary Figure 261. ^{1}H NMR ($CDCl_3$, 500 MHz, 298 K) spectrum for **11**

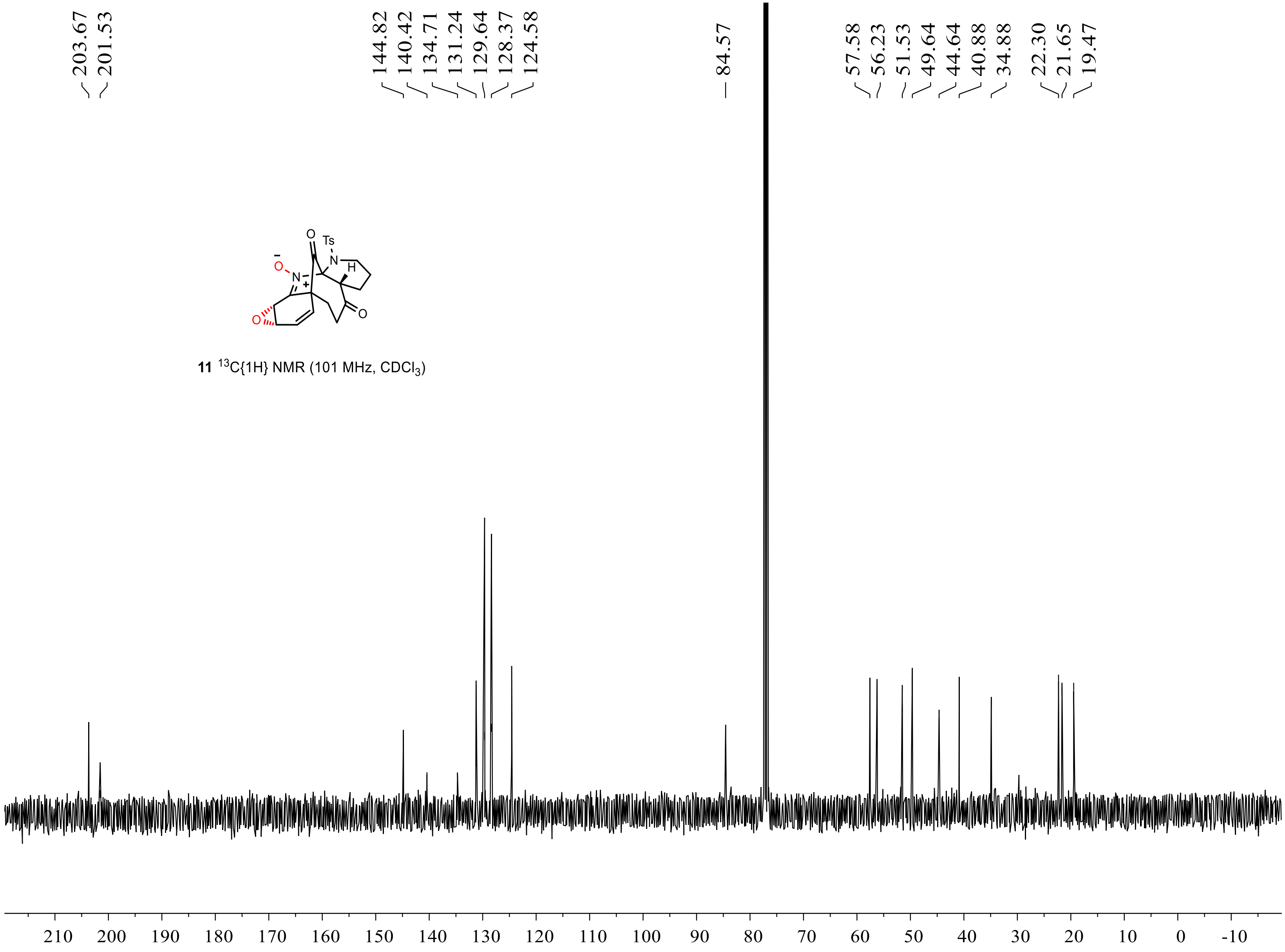

Supplementary Figure 262. ^{13}C NMR ($CDCl_3$, 126 MHz, 298 K) spectrum for **11**

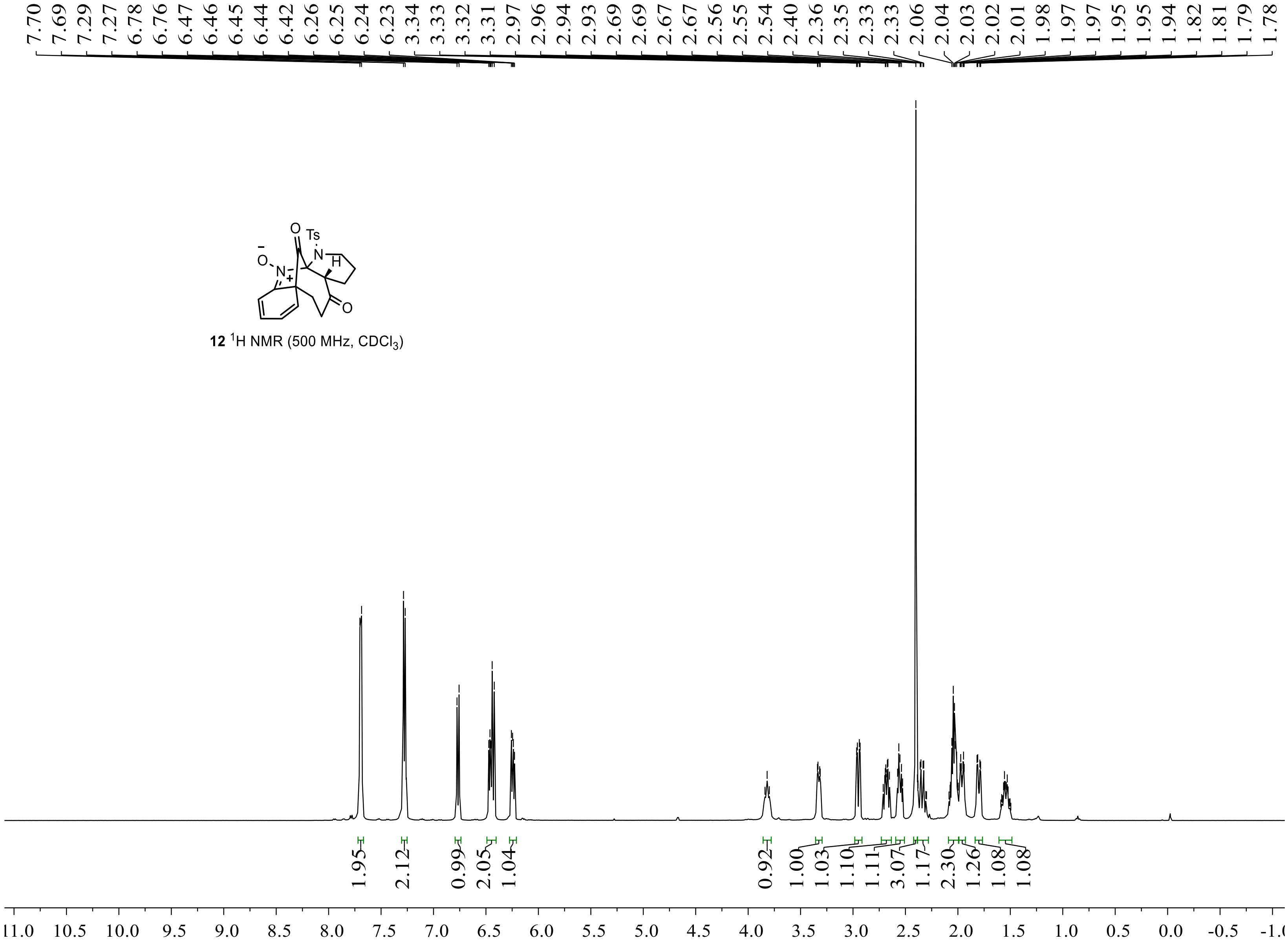

Supplementary Figure 263. ^{1}H NMR ($CDCl_3$, 500 MHz, 298 K) spectrum for **12**

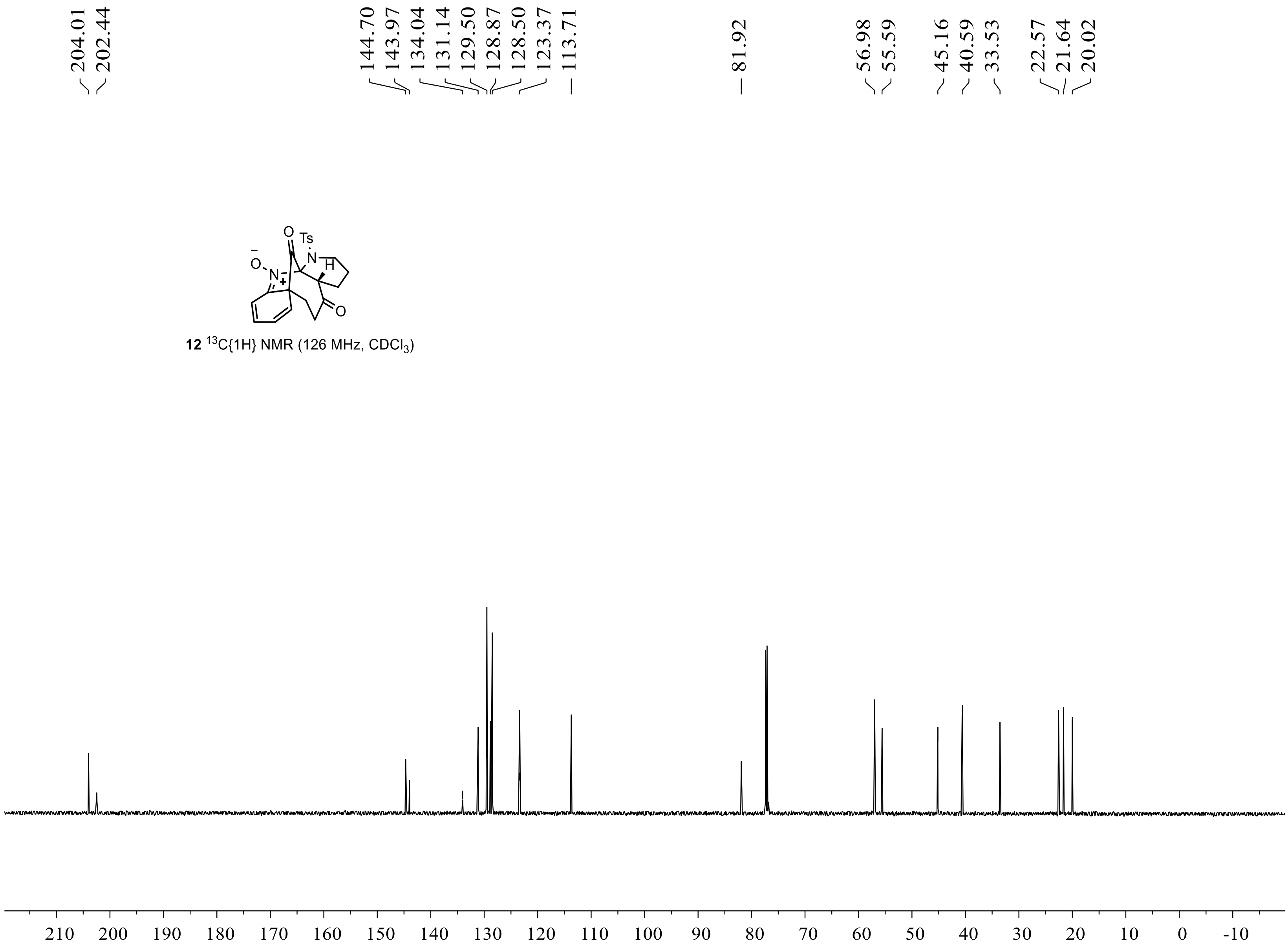

Supplementary Figure 264. ^{13}C NMR ($CDCl_3$, 126 MHz, 298 K) spectrum for **12**

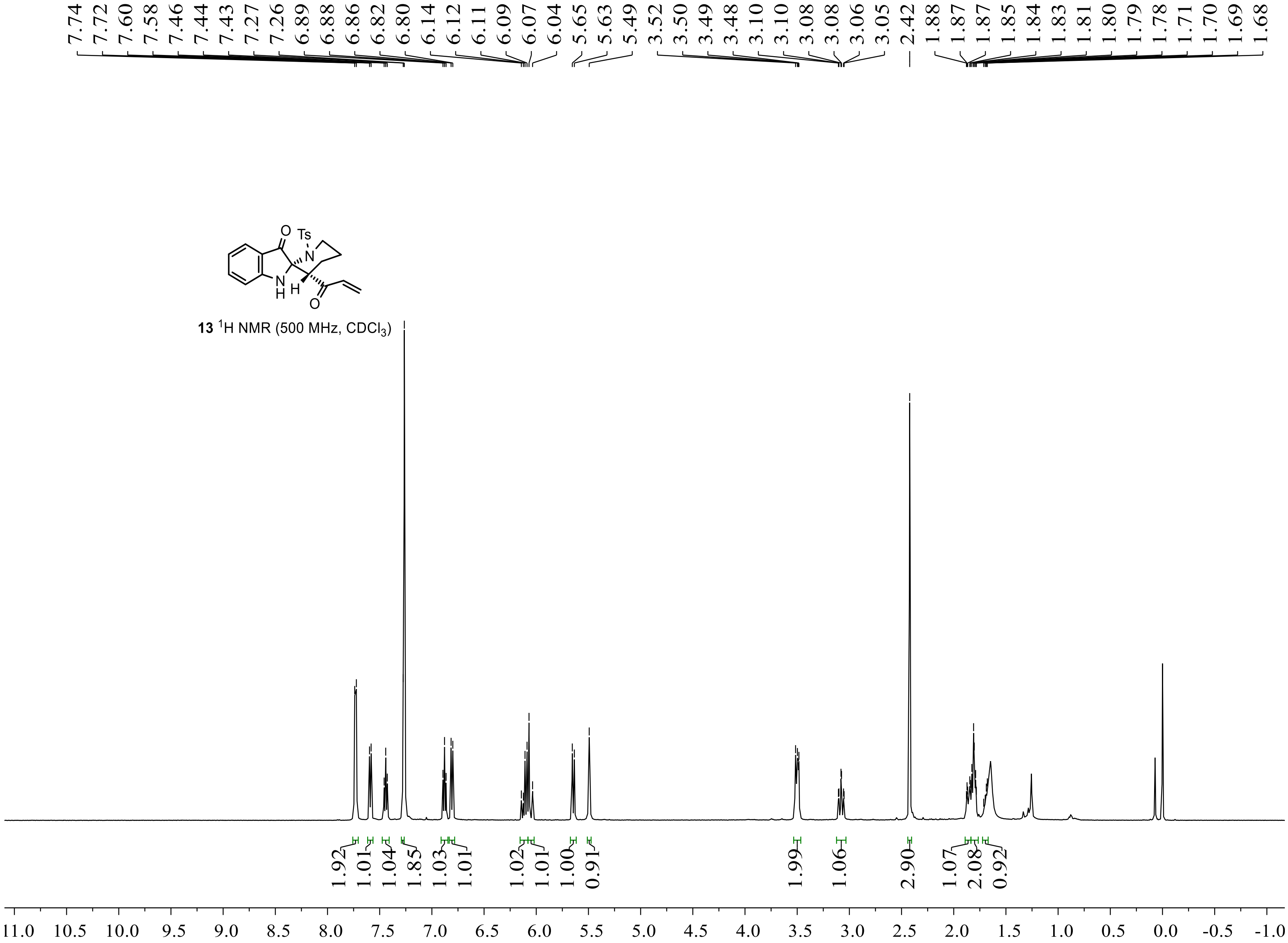

Supplementary Figure 265. ^{1}H NMR ($CDCl_3$, 500 MHz, 298 K) spectrum for **13**

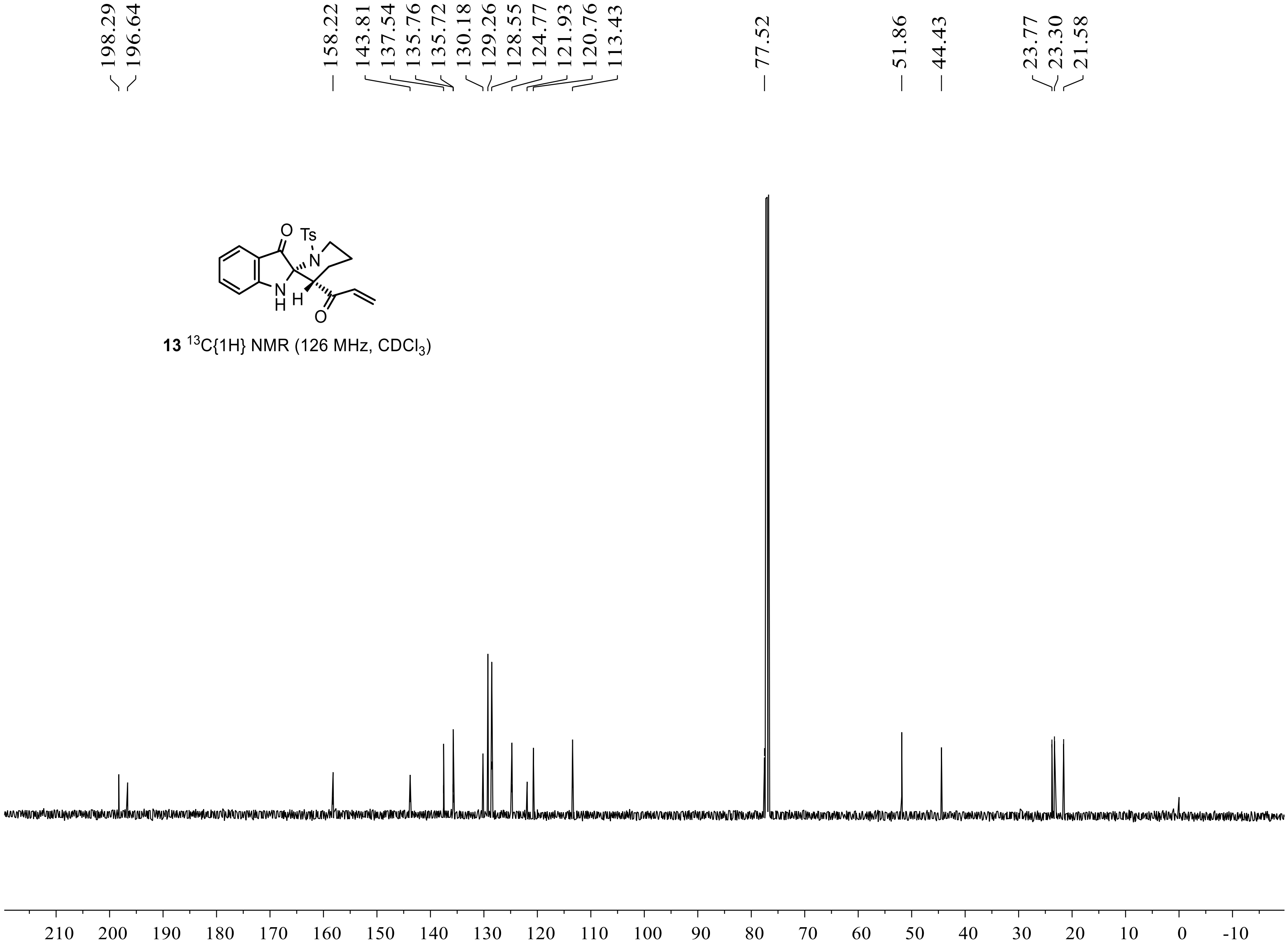

Supplementary Figure 266. ^{13}C NMR ($CDCl_3$, 126 MHz, 298 K) spectrum for **13**

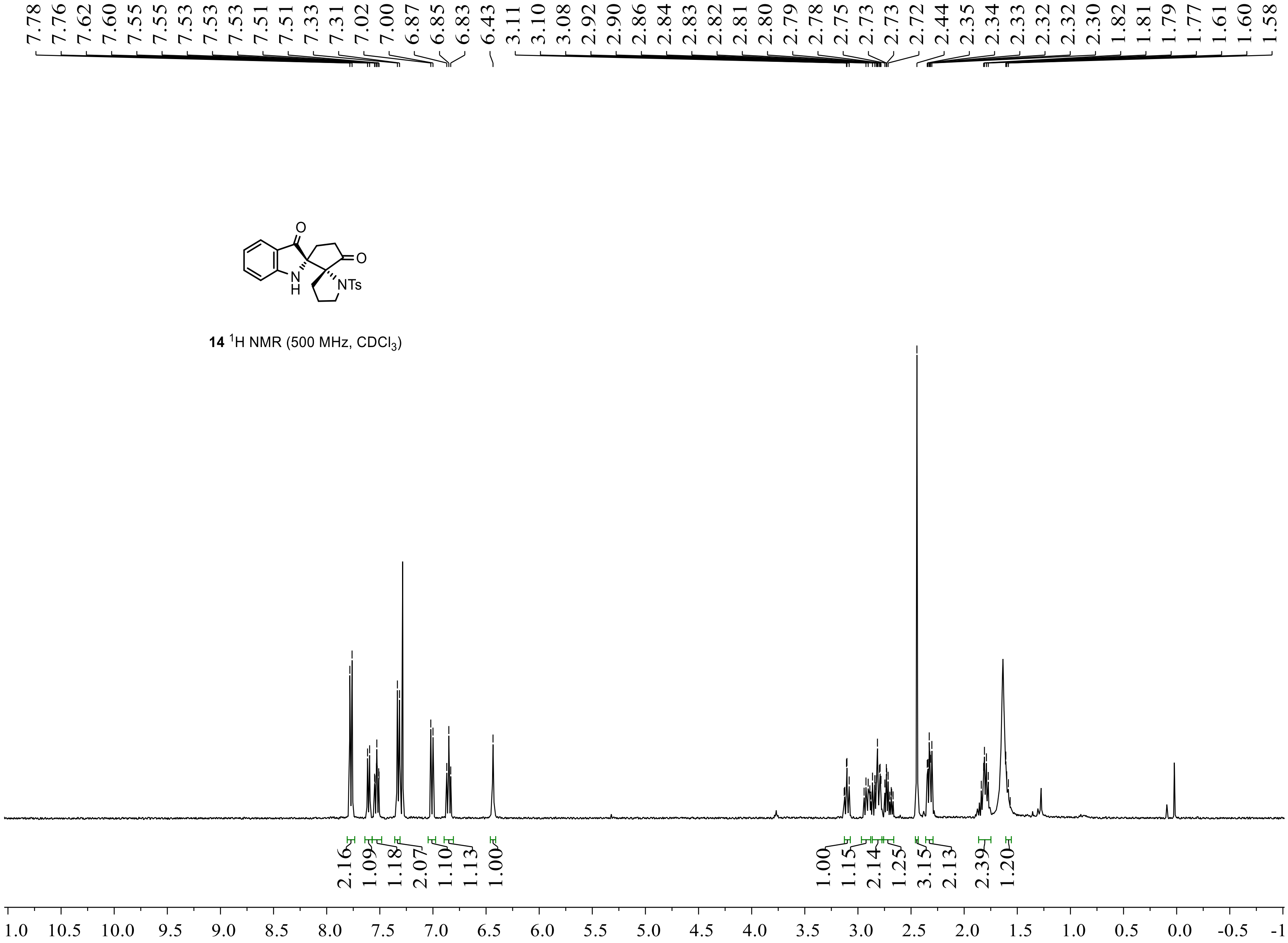

Supplementary Figure 267. ^{1}H NMR ($CDCl_3$, 500 MHz, 298 K) spectrum for **14**

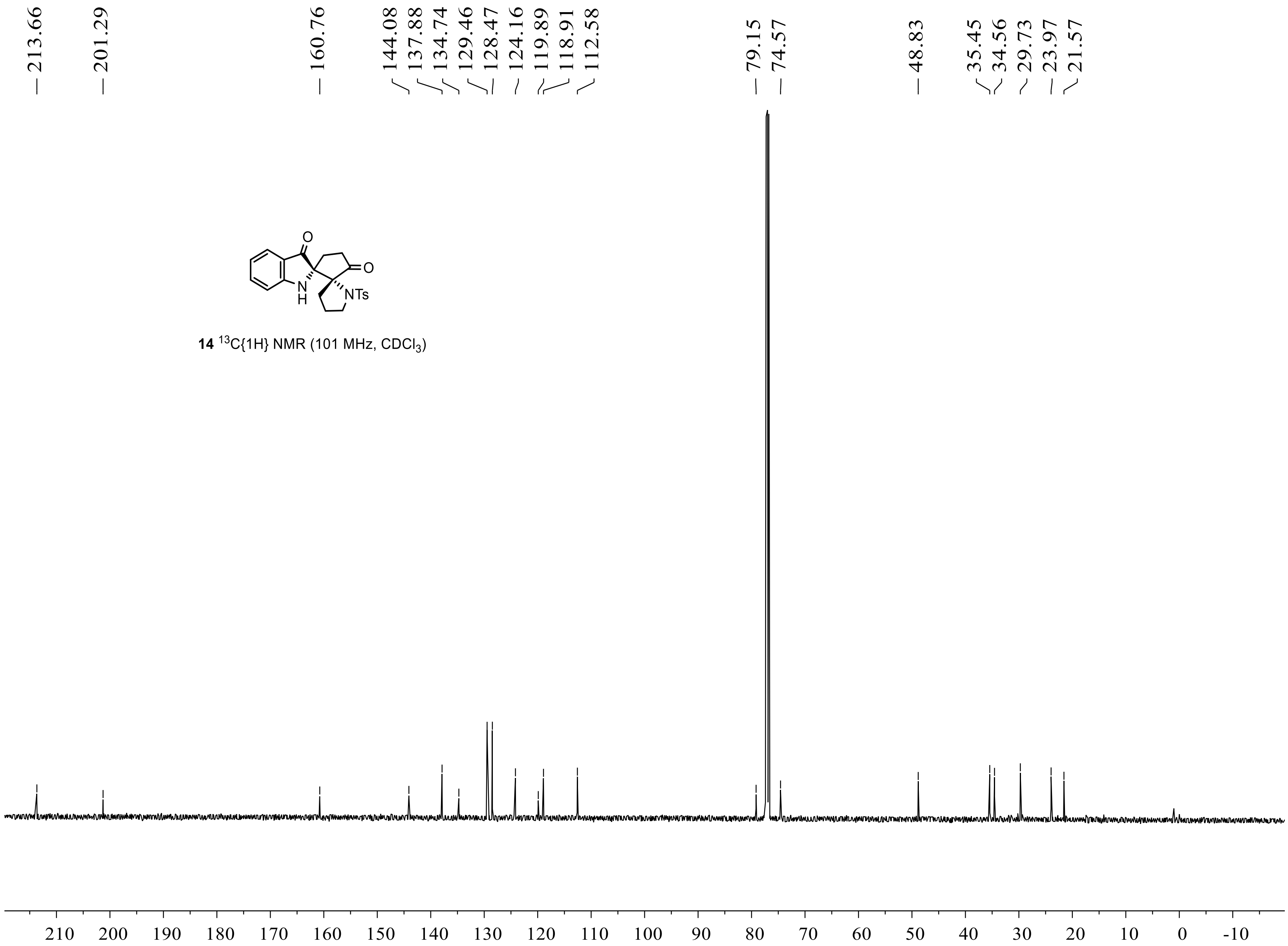

Supplementary Figure 268. ^{13}C NMR ($CDCl_3$, 126 MHz, 298 K) spectrum for **14**

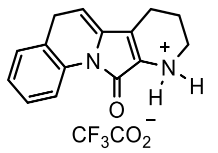

15 ^1H NMR (500 MHz, $CDCl_3$)

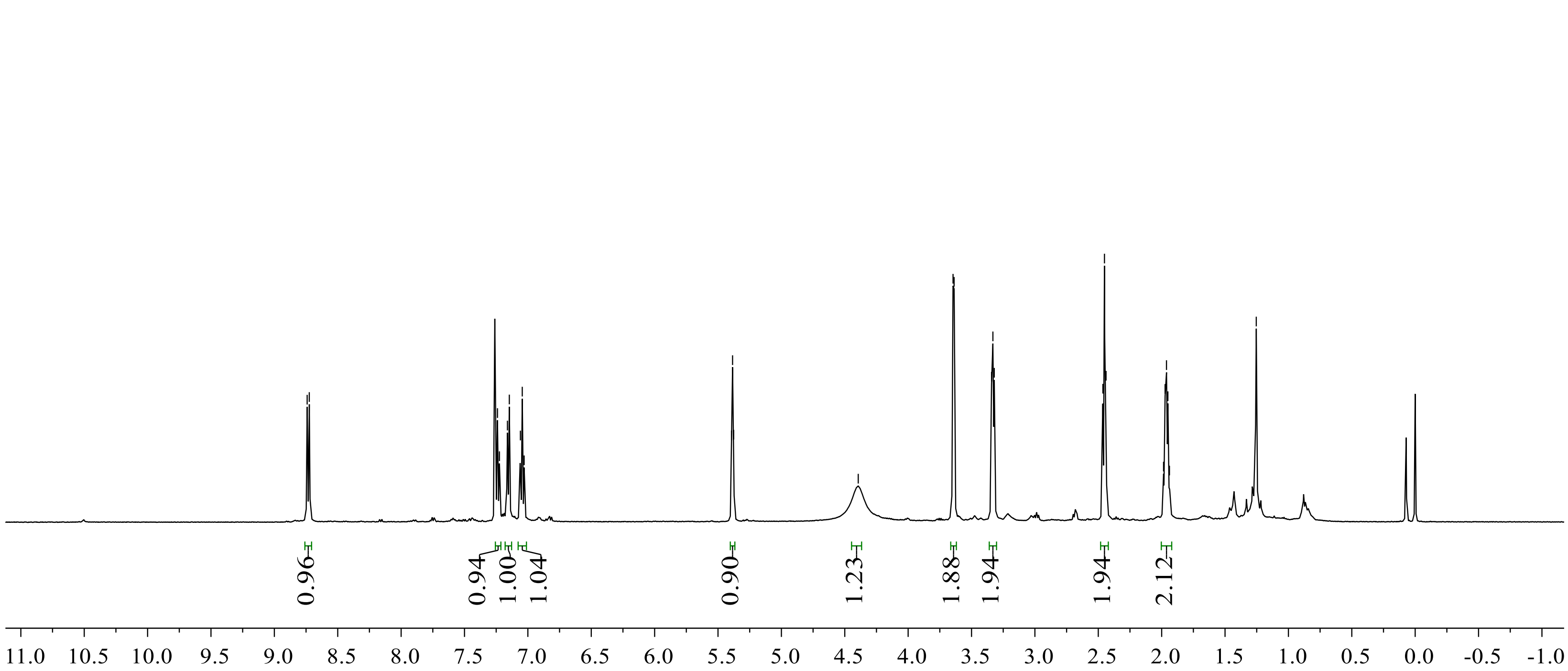

Supplementary Figure 269. ^1H NMR ($CDCl_3$, 500 MHz, 298 K) spectrum for **15**

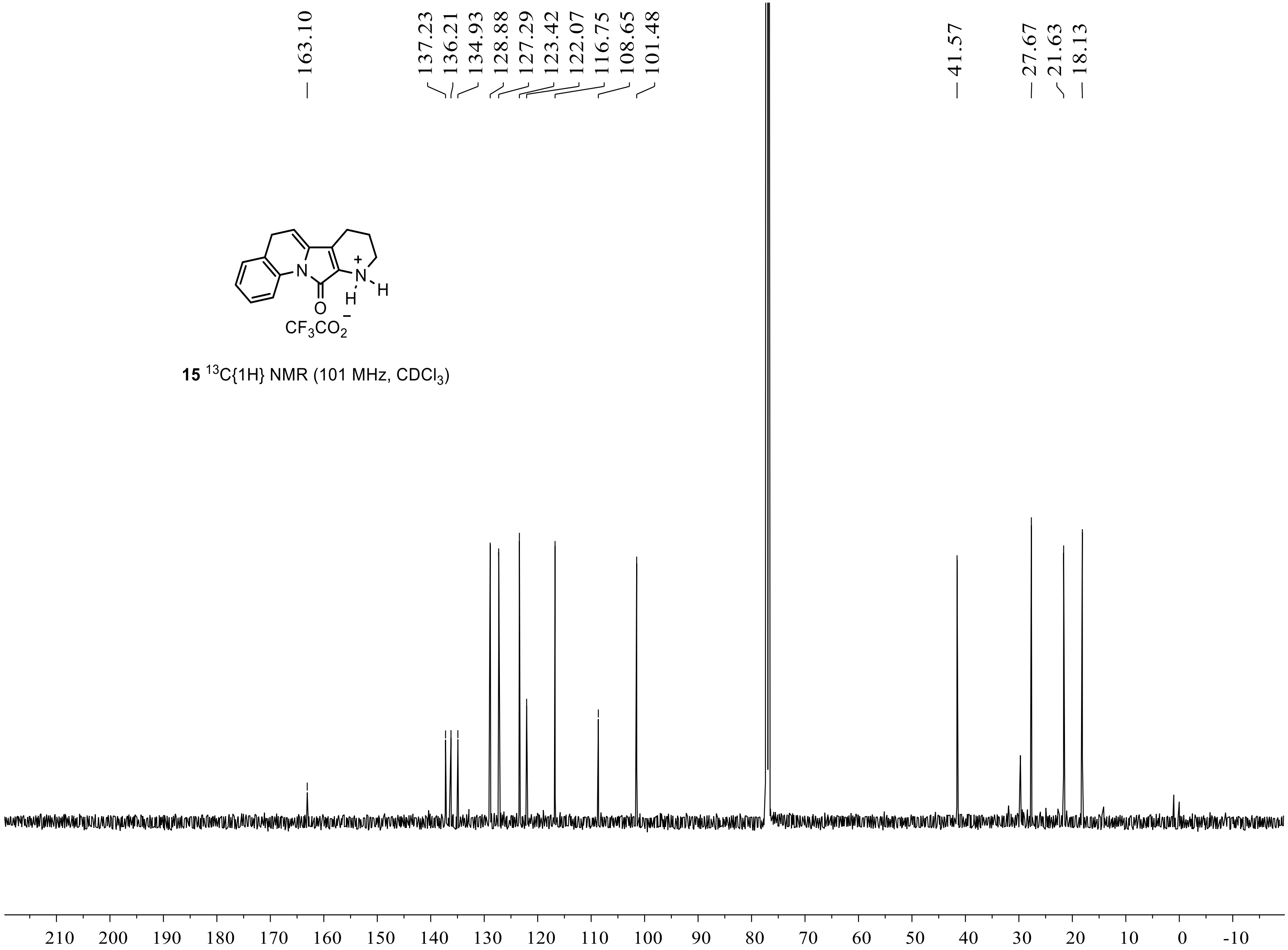

Supplementary Figure 270. ^{13}C NMR ($CDCl_3$, 126 MHz, 298 K) spectrum for **15**

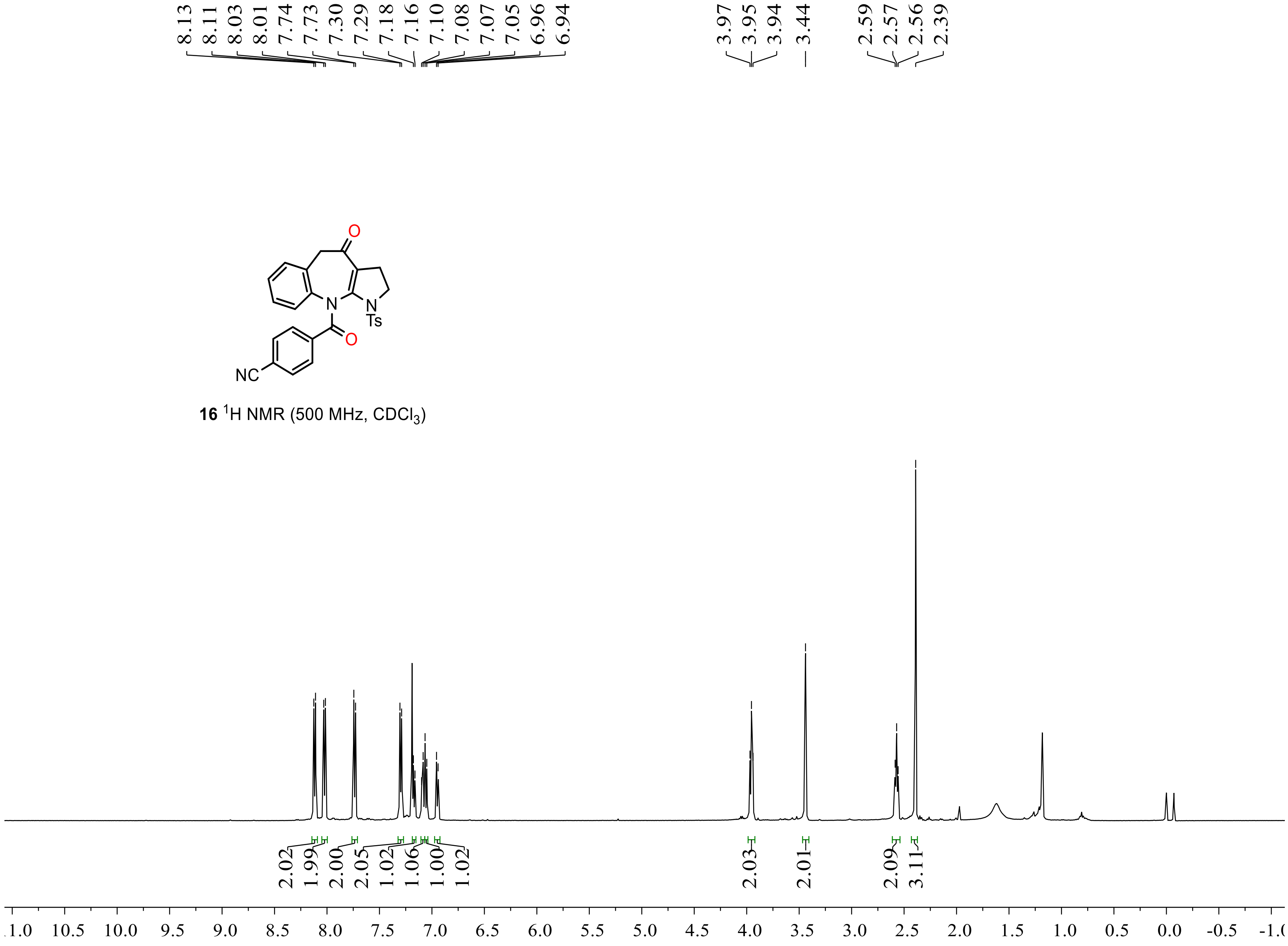

Supplementary Figure 271. ¹H NMR ($CDCl_3$, 500 MHz, 298 K) spectrum for **16**

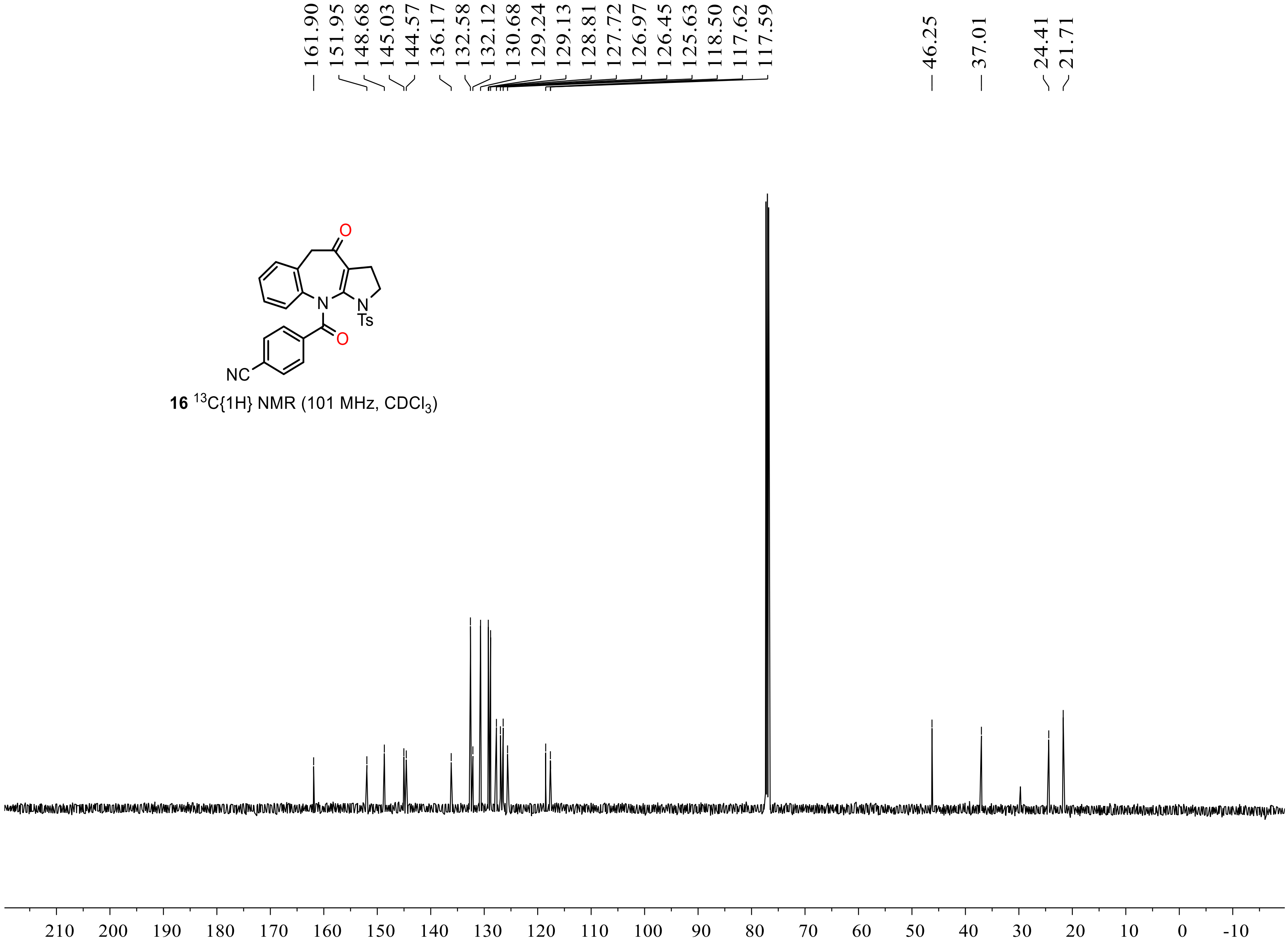

Supplementary Figure 272. ^{13}C NMR ($CDCl_3$, 126 MHz, 298 K) spectrum for **16**

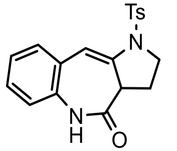

17 ^{1}H NMR (500 MHz, $CDCl_3$)

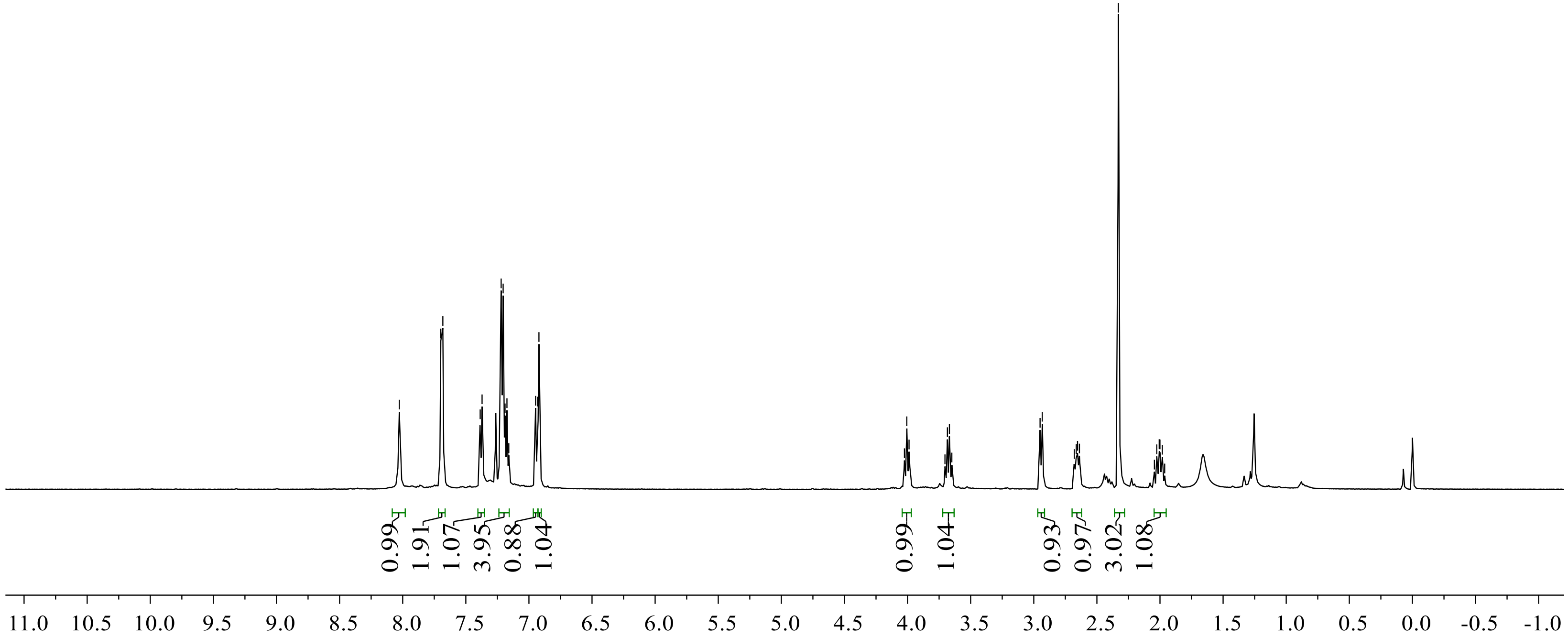

Supplementary Figure 273. ^{1}H NMR ($CDCl_3$, 500 MHz, 298 K) spectrum for **17**

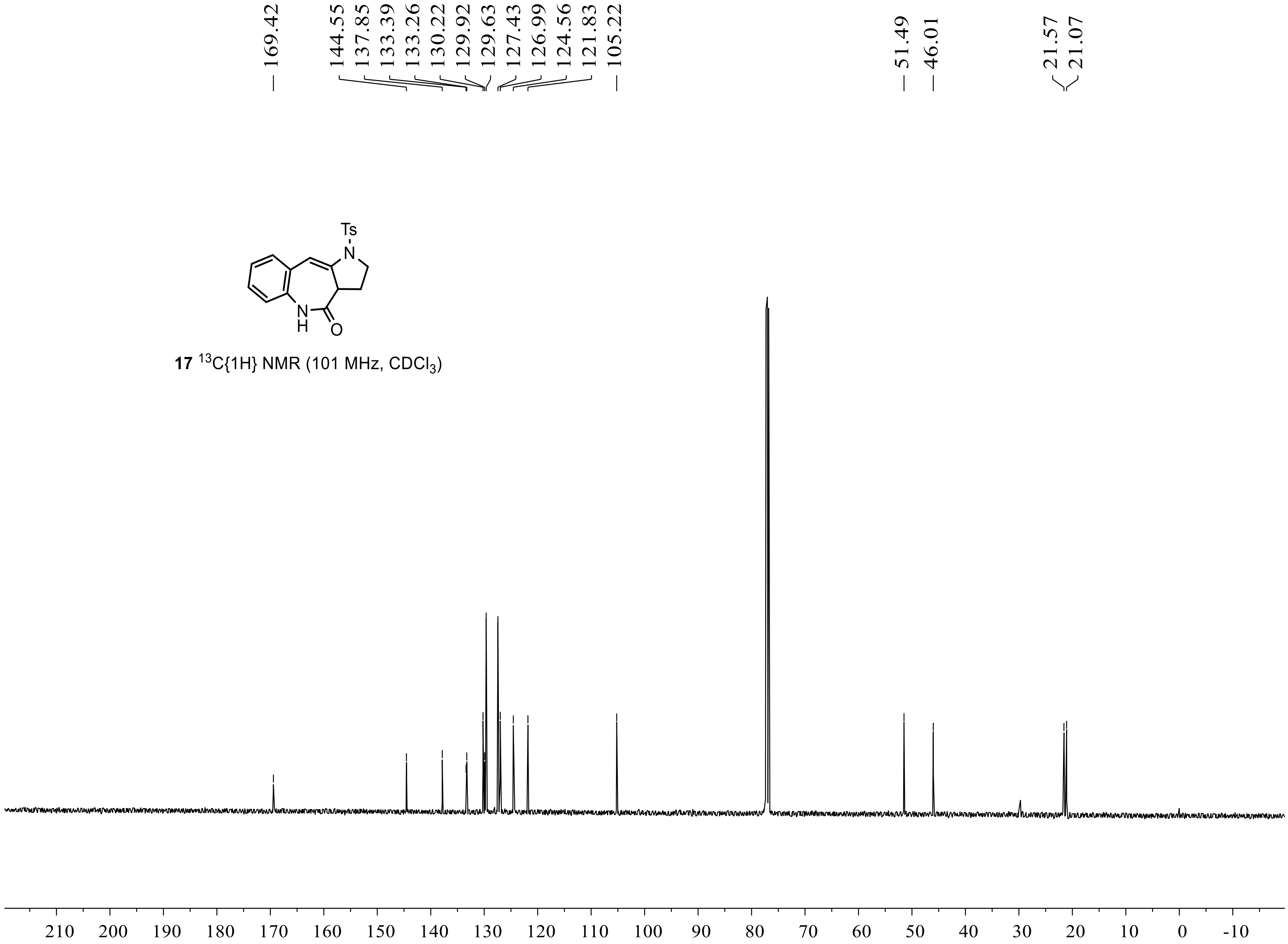

Supplementary Figure 274. ^{13}C NMR ($CDCl_3$, 126 MHz, 298 K) spectrum for **17**

IV. Supplementary References

1. Kunzer, A. R.; Wendt, M. D. Rapid, robust, clean, catalyst-free synthesis of 2-halo-3-carboxyindoles. *Tetrahedron Lett.*, **52**, 1815 (2011).

2. Berthold, D.; Geissler, A. G. A.; Giofré, S.; Breit, B. Rhodium-catalyzed asymmetric intramolecular hydroamination of allenes. *Angew. Chem. Int. Ed.*, **58**, 9994 (2019).

3. Serafino, A.; Balestri, D.; Marchiò, L.; Malacria, M.; Derat, E.; Maestri, G. Orthogonal syntheses of 3.2.0 bicycles from enallenes promoted by visible light. *Org. Lett.*, **22**, 6354 (2020).

4. Walters, J. C.; Tierno, A. F.; Dubin, A. H.; Wengryniuk, S. E. (poly)cationic λ^3-iodane-mediated oxidative ring expansion of secondary alcohols. *Eur. J. Org. Chem.*, **2018**, 1460 (2018).

5. Rabe, P.; Barra, L.; Rinkel, J.; Riclea, R.; Citron, C. A.; Klapschinski, T. A.; Janusko, A.; Dickschat, J. S. Conformational analysis, thermal rearrangement, and EI-MS fragmentation mechanism of (1(10)*E*,4*E*,6*S*,7*R*)-germacradien-6-ol by ^{13}C-labeling experiments. *Angew. Chem. Int. Ed.*, **54**, 13448 (2015).

6. Suneel Kumar C V, Ramana C V. Tuning the Regioselectivity of Gold-Catalyzed Internal Nitroalkyne Redox: A Cycloisomerization and [3 + 2]-Cycloaddition Cascade for the Construction of spiro-Pseudoindoxyl Skeleton. *Org. Lett.*, **16**, 4766 (2014).

7. Marien N, Brigou B, Pinter B, et al. Synthesis of 2-Spiropseudoindoxyls via an Intramolecular Nitroalkyne Redox–Dipolar Cycloaddition Cascade. *Org. Lett.*, **17**, 270 (2015).
